# Identification of Salty Dietary Patterns of the Japanese Macroregion

**DOI:** 10.1155/2021/6675418

**Published:** 2021-07-22

**Authors:** Masana Yokoya, Miyuki Fukuhara, Emiko Haga, Yuka Shimamura, Aki Terada

**Affiliations:** ^1^Shimonoseki Junior College, 1-1 Sakurayama-Cho, Shimonoseki 750-8508, Yamaguchi, Japan; ^2^University of Human Environments, 6-2 Kamisanbonmatsu Motojuku-Cho, Okazaki 444-3505, Aichi, Japan; ^3^Yamaguchi Prefectural University, 3-2-1 Sakurabata, Yamaguchi 753-8502, Japan

## Abstract

**Objective:**

The traditional Japanese dietary pattern, “Washoku,” is considered to provide an ideal nutritional balance. However, it tends to have a high salt intake. To reduce population-level salt intake, it is important to review the overall dietary patterns over a wide area.

**Methods:**

To identify dietary patterns with high salt intake, partial least squares regression analysis was conducted using population-based household survey data from 52 cities. Annual salt consumption was set as the target variable, and the annual purchase weight of 109 foods was set as the explanatory variable.

**Result:**

At least three dietary patterns (traditional, urban, and local) accounted for more than 90% of the variation in salt consumption and 29% of the explanatory variables (food purchases). Traditional patterns explained the salt consumption and regional differences in energy and protein consumption; however, their relationship to fat consumption was weak. The results reconfirmed that “Washoku” has an ideal nutritional balance but has high salt intake. The distribution of scores for traditional Japanese food patterns was high in northeast Japan and low in southwest Japan, with a geographical gradient. This pattern is thought to have formed over a long period of time because of the influence of environmental factors, such as local climate.

## 1. Introduction

In 2018, the traditional Japanese dietary pattern, “Washoku,” was registered as a UNESCO World Intangible Cultural Heritage. The Japanese eating style based on “Ichiju Sansai” (one soup and three side dishes) is considered to provide an ideal nutritional balance. By using “Umami” (dashi stock) skillfully, those who consume this diet ingest low levels of animal fats and oils, which is beneficial for longevity and for the prevention of obesity in Japanese people [1–8].

In contrast, Japanese dietary patterns tend to have high salt content [9, 10]. The Japanese Society for Hypertension Guidelines for the Management of Hypertension (JSH2014) recommends limiting salt intake to less than 6 g/day [11], and the Japanese Dietary Reference Level (2015) recommends a salt intake of less than 7.5 g/day [12]. However, according to the “Outline of National Health and Nutrition Survey” conducted by the Ministry of Health, Labor, and Welfare, the daily salt intake of Japanese people was 10.8 g for men and 9.1 g for women in 2017 [13]. These values are far from the World Health Organization guidelines of less than 5 g/day.

The levels of salt intake in Japanese people are high in the northeast region, which may be due to differences in eating habits, food culture, and food availability, depending on the region [13]. To reduce salt intake, it is important to not only reduce the use of salt and the intake of foods with high salt content, but also review the overall dietary pattern.

However, there are challenges in conducting a national survey to understand the differences in eating habits and food culture in different regions. For example, in Japan, there is an annual National Health and Nutrition Examination Survey [13]. However, this study aimed to investigate the current state of nutrient intake and the survey period is short; thus, it is not suitable for understanding eating habits and food culture.

However, household budget surveys are conducted in many countries to monitor the economic situation of the population, including the Family Income and Expenditure Survey (FIES) in Japan [14]. The FIES is used to understand the economic balance of each household, and the purchase amount of food is listed per household. Since the household budget survey is not a nutrition survey, the variety of foods listed is limited, and the resulting analysis does not assess intake but instead the purchase amount per household. However, this is a nationwide year-long survey. Therefore, from this survey, changes in purchase volume and purchase price over time as well as region-specific purchase trends can be observed [15, 16].

Since this survey is not nutritional, it is not possible to determine the exact amount of nutrient intake, but it is possible to identify an overall dietary pattern with a degree of validity. Previous studies that have derived dietary patterns from household survey data have been conducted in Europe and South America [17, 18].

Empirical methods frequently used to derive dietary patterns include principal component analysis (PCA), reduced rank regression (RRR), and partial least squares (PLS) regression analysis. The dietary patterns obtained using PCA tended to explain the high variation in dietary intake, thus the dietary patterns of real populations. However, these patterns may not be relevant to variables of interest, such as disease risk or nutrient intake [19, 20]. RRR, on the other hand, is used to build a linear function of the food that best describes the variation in the outcome variable. Therefore, compared to patterns derived by PCA, those obtained using RRR tend to be more associated with the outcome, despite being behaviorally irrelevant [20, 21]. The PLS regression analysis combines the advantages of PCA and RRR. It is used to identify patterns that maximize the variance explained by both dietary intake (food pattern) and intermediate response variables (e.g., nutrient intake) associated with health or disease outcomes. Such dietary patterns may allow directed data reduction of food intake variable groups via specific nutrients and biomarkers of interest [20, 22].

Therefore, in this study, we attempted to derive the dietary pattern of high salt intake using PLS regression analysis using population-level food purchase data based on a household survey in 52 prefectural offices and ordinance-designated cities in Japan. In addition, we compared the results of the analysis in two different statistical years and examined the validity and reproducibility of pattern extraction.

## 2. Materials and Methods

In this population-based study, we obtained annual mean food consumption data of households with two or more people in 47 prefectural capitals and five ordinance-designated cities from the results of the 2013 and 2018 Family Income and Expenditure Survey (FIES) as basic data. The data scale and sampling method are available on the homepage of the Statistics Bureau, Ministry of Internal Affairs and Communications [14]. From these datasets, basic data, such as average consumption expenditure, age of household heads, number of persons per household, and annual purchase weight of foods, were extracted.

Based on the annual food consumption of 47 prefectural capitals and five ordinance-designated cities (total 52 cities), we selected 109 foods with a record of purchase weight and calculated their basic nutritional value, including annual total calories, salt, protein, fat, and carbohydrates per household in each city. Nutritional values were calculated according to the 2015 Japanese Dietary Intake Standard [12]. The analysis of dietary patterns often involves the integration of several foods or food groups with similar nutritional values or culinary uses, but this was not the case in the present study. According to the FIES, the amount of “tofu” purchased was recorded in units of chops. In this study, the nutritional value was calculated assuming that the weight of one tofu chop was 350 g. Ground meat was calculated as beef:pork 6 : 4. Coffee was calculated as the weight of the infused beverage and not the weight of the beans. Details of the 109 foods and accompanying calculation units are shown in Supporting Files S1 (data for 2013) and S2 (data for 2018). The total salt value (sum of nutrient calculations) was used as a target variable (objective variable, response variable) for PLS regression, as described later in this study.

To identify the dietary patterns of high salt intake, PLS regression analysis was conducted using the annual total salt purchase weight from all foods (sum of nutrient calculations) in 52 prefectural capitals and ordinance-designated cities as the objective variable and the annual purchase weight of 109 foods as the explanatory variable. PLS calculations were performed using the PLS package in R. The calculation was based on a correlation matrix [23–25]. PLS regression analysis was used to identify patterns that maximized the variance explained by both food purchases and intermediate response variables (salt consumption). The dietary patterns derived by this method were expected to resemble the results of principal component analysis and explain the variance in salt consumption well [20].

We compared the 2013 and 2018 purchases for each of the 109 food products for each prefectural capital and ordinance-designated city and used Spearman's correlation coefficients to assess the reproducibility of the 109 food purchase patterns. In the comparisons, we did not adjust the amount purchased in each year by the total calories purchased, average income, or any other criteria.

The loading vectors of 109 foods and 52 city factor scores obtained for each dietary pattern in each year were also compared to the 2013 and 2018 values, and their reproducibility was assessed using Spearman's correlation coefficient. In addition, the factor scores of the prefectural capitals obtained for each dietary pattern were displayed on a map to investigate the continuity and validity of the distribution.

## 3. Results

Table 1 provides the basic statistics for the 52 cities in FIES 2013 and 2018. Detailed data regarding average annual total consumption expenditure, age of household heads, and number of persons per household in two-or-more-person households in 52 prefectural capitals and ordinance-designated cities are listed in Supporting Files S1 (data for 2013) and S2 (data for 2018). The total number of sample households in the 52 cities was greater than 5,000, ranging from 78 to 150 in 2015 and 83 to 161 in 2018 per city (except for Tokyo's 23 wards). The average annual total consumption expenditure of the 52 cities was approximately three to four million yen, and only Naha City had an outstandingly low expenditure. The total average age of the heads of households ranged from 53.8 to 61.5 years in 2015 and 54.6 to 63.5 y in 2018. The total average number of persons per household ranged from 2.82 to 3.37 in 2015 and 2.72 to 3.30 in 2018. There was no noticeable relationship between these variables, but the older the household head, the smaller the number of household members.

To compare food purchase trends in 2013 and 2018, we ranked the cities for each of the 109 foods in descending order based on purchase weight, and Spearman's correlation coefficients of the purchase rankings between 2015 and 2018 were calculated (Table 2). The higher the value is, the more the purchase ranking of the city does not change, indicating that the purchase pattern is region-specific. The correlation coefficients for purchases in 2013 and 2018 were higher for fresh fish, seafood, meats, and frequently used vegetables but lower for less purchased items, such as condiments, fruits, and dairy products.

PLS regression analysis was used to identify dietary patterns with high salt consumption from the 2013 and 2018 food purchase records. At least three factors (dietary patterns) accounted for more than 90% of variations in salt consumption and 29% of variations in explanatory variables (food purchases). In particular, the influence of the first factor was large, and more than half of the fluctuations in salt consumption were explained (Table 3).

**Figure 1:**
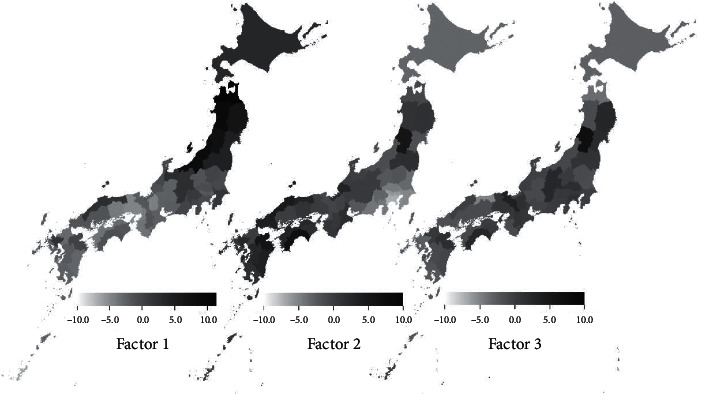
Distribution map of loading scores 1 to 3 in 2013 based on the Family Income and Expenditure Survey 2013. The values of 47 prefectural capitals of 52 cities are shown as different colors for each prefecture. See Table 5 and Supporting Figure S1.

Table 4 compares the 2013 and 2018 values for the factor loadings of the three factors (dietary patterns). The loading scores of the three factors for all cities are shown in Table 5, and the distribution of loading scores for the 47 prefectural capitals is shown in Figure 1. Furthermore, Table 6 shows the correlation between the scores of the three factors in each of the 52 cities, as well as the basic nutrient amount calculated from purchase weights.

In Table 4, for simplicity, factor loadings of less than ±0.10 are not listed. The loading factors for 2013 and 2018 were similar to each other, with the Spearman's correlation coefficient of the first loading vector being 0.84, second being 0.88, and third being 0.63.

The first loading factor showed that the purchase of vegetables, seafood, and seasonings, such as salt and miso, was high. This may represent the aforementioned typical Japanese food pattern; thus, it was named the traditional Japanese pattern. The score of the traditional Japanese pattern was higher in the northern region than in the southern region; thus, a geographical gradient exists (Table 5, Figure 1). The correlation between the scores of the traditional Japanese pattern in each city and the basic nutrient amount calculated from the purchased amounts was strong, not only in terms of the amount of salt but also in terms of the amount of protein and dietary fiber. However, the relationship with the amount of fat was weak (Table 6).

Contrary to the first loading factor, the second loading factor indicated that salt intake was suppressed when the purchase of vegetables, fruits, and dairy products increased (Table 4). These scores were low in the suburbs of Tokyo and large cities (Table 5, Figure 1) and did not show a strong correlation with nutrients other than salt (Table 6). This dietary pattern was characterized by the purchase of vegetables, dairy products, and fruits that do not take long to cook, and because this pattern was found in large cities, it was named the urban Japanese pattern.

The third factor showed that the purchase of seafood, such as shrimp and crab, resulted in a decrease in the amount of salt and the purchase of dairy products resulted in an increase in the amount of salt. These scores were low in coastal cities, such as Tottori and Akita, and high in inland cities, such as Nagano, Kofu, and Yamagata. Although it was not possible to associate a simple dietary pattern among the localities, local patterns were identified at a smaller scale than those captured by the first and second loading factors; thus, it was named the local Japanese pattern.

## 4. Discussion

We identified three major salt-rich dietary patterns (traditional, urban, and local Japanese) from food purchase records in 52 cities across Japan from the FIES using PLS regression analysis. Three dietary patterns accounted for more than 90% of the variation in salt consumption (Table 3).

The reproducibility of the dietary patterns from the FIES was acceptable. Spearman's correlation coefficient of the loadings of dietary patterns between 2013 and 2018 ranged from 0.88 to 0.63 (Table 4). In addition, Spearman's correlation coefficient of the scores between 2013 and 2018 surveys ranged from 0.77 to 0.38 (Table 5). The reproducibility of these results is acceptable, considering that we also analyzed foods with low purchase levels and large annual fluctuations in purchases.

The major dietary patterns identified may have been reported in previous Japanese studies. Most Japanese studies have identified dietary patterns that are similar to the traditional Japanese patterns in this study; namely, this dietary pattern is characterized by a large intake of typical Japanese foods, such as vegetables, seafood, soy products, seaweed, mushrooms, fruits, and green tea [4–7, 26, 27]. In this study, a pattern of large purchases of vegetables, seafood, seaweed, and fruit was identified as the traditional Japanese pattern. This pattern is the main pattern that causes regional differences in salt, energy, dietary fiber, and protein amounts. However, the relationship with the amount of fat was weak. “Washoku” is considered to have an ideal nutritional balance when it comes to a combination of staple foods and miso soup, but its salt intake is high. The results of this study confirmed this [3–6].

The distribution of the scores for the traditional Japanese food patterns was high in northeast Japan and low in southwest Japan, and a geographical gradient existed. Japanese food is characterized by seasonal ingredients, and this distribution may be closely related to climatic factors [16]. For example, from the factor loadings of food groups in the traditional Japanese pattern (Table 4), we can associate menus such as miso soup and hot pot dishes [10], and the purchase amounts of miso and soy sauce are high in northeast Japan and low in southwest Japan [14]. In addition, the purchase amounts of miso and soy sauce are seasonal, and the purchase amounts of miso and soy sauce are low in summer and high in winter [14]. Miso soup and hot pots are usually eaten while they are hot; thus, they are favored during the cold season. On the other hand, Japan is hot and humid in summer; thus, hot pot dishes are not desirable during this time. For the same reason, warm dishes may be preferred in cold regions, and cold dishes may be preferred in warm regions. This may explain why salt intake was high in northeastern Japan.

In recent years, it has become clear that salt intake plays an important role in maintaining body temperature. Animal studies have suggested that the physiological need for salt intake may be increased in cold environments [28–30]. It is unclear whether this north-south gradient of salt intake is a result of the physiological desires of the cold environment or cultural and social factors. However, the first pattern is thought to represent the pattern of traditional Japanese food, which is believed to have formed over a long period of time under the influence of environmental factors, such as the local climate. Therefore, when considering a low-salt diet, it would be beneficial to consider the historical and environmental formation processes of this dietary pattern [31].

A number of Japanese studies have also observed dietary patterns similar to those of the urban Japanese patterns observed in the present study, namely, patterns with high intake of meat, processed meat, dairy products, and raw vegetables. Other studies may have named these diets differently, for example, as westernized food patterns [6, 7, 15, 26, 27]. In this study, the factor loadings of the urban Japanese pattern showed that increasing purchases of vegetables and dairy products reduced salt consumption. The factor loading of meat did not show a particular westernized feature, but the score distribution was divided between large cities and rural areas. This food pattern may reflect differences in generational composition and lifestyles in urban and local areas.

Dietary patterns tend to vary widely between generations [27, 32–34]. The number of people living with elderly households is low in urban areas and high in rural areas. Particularly in rural areas, there are many cases of children living in the immediate vicinity of their parents' households, even if they do not live together [35]. These households are likely to inherit traditional food patterns. In the FIES, the sampling process is designed to avoid large differences in the average age of the heads of households in each city [14]. However, if households are independent of the budget record, they are treated as independent households, even if they live together. Therefore, living together with or near an elderly household may reduce urbanization and westernization of food patterns.

Urban households have high incomes and a high rate of food externalization [14]. Due to their busy schedules, these households are likely to eat out more often and prefer foods that do not take a lot of time to prepare at home [13, 17, 18].

In this study, bread was not detected as a representative ingredient in urban Japanese patterns. Unlike rice, bread can be eaten immediately, and no further preparation is required. In recent years, the consumption expenditure of bread has exceeded that of rice in Japan [14]. Bread should be universally popular not only in urban areas but also in local areas, not only for young people but also for the elderly. This is why it is no longer regarded as a representative food for urbanization or westernization. In addition, while westernized food patterns are usually characterized by an increase in the consumption of meats, in this study, the factor loading of meats did not indicate a westernized feature. This may be related to meat having different preference levels in Kanto (East Japan) and Kansai (West Japan). In western Japan, where cows are used extensively in farming, cows tend to be consumed. In eastern Japan, where horses are used for farming, pigs tend to be consumed [36].

Traditional and urban Japanese patterns have been observed in many other studies, but most of these are studies on individuals [26, 27]. The traditional and urban Japanese dietary patterns detected in this study were extracted to explain salt intake; however, they likely represent dietary patterns similar to those detected in individual-level studies. This means that while Japanese people inherit traditional dietary patterns, gradually westernized, externalized, or more hassle-free dietary patterns are becoming more prevalent over time, and this will be the biggest factor that changes dietary patterns, both individually and in groups.

The distribution of the score of the local Japanese pattern was dispersed, and there was no geographical continuity. It was not possible to associate the previously reported dietary pattern with the local Japanese pattern, but considering the distribution of scores for the first (traditional Japanese pattern) and second (urban Japanese pattern) factors showed a degree of geographical continuity, and the third factor is thought to reflect a more local dietary pattern.

The Japanese archipelago is made up of islands that stretch to the north and south. Japan is a volcanic country; thus, it is very mountainous with a wide range of altitudes, resulting in extreme variations in climate. In addition, the Japanese archipelago is influenced by the monsoon and has four distinct seasons. Such an environment becomes a factor that emphasizes regional characteristics, resulting in the development of various local dishes and unique food cultures in different regions. However, factor 3 was inferior to factors 1 and 2 in terms of reproducibility, and Spearman's correlation coefficient was 0.63, in the comparison between loading 2013 and 2018 (Table 4), and was 0.38, in the score comparison (Table 5). The third dietary pattern accounted for only approximately 10% of fluctuations in salt consumption and approximately 4%–5% of fluctuations in food purchases (Table 3). The purchase pattern shown by the local Japanese pattern was difficult to interpret as a purchase habit.

In this study, PLS regression analysis was performed using the average values of household surveys taken in 52 cities to identify dietary intake patterns with high salt intake. Some food purchase patterns were regionally specific, and the geospatial distribution of scores over a wide macroregion provided insights into the factors involved in the establishment of the food pattern. In this respect, it was considered to be sufficiently effective and thus suitable for extracting dietary patterns. In this study, to avoid becoming arbitrary, we directly used the food purchase weight from the FIES for analysis. However, with respect to foodstuffs purchased in small quantities, purchase weights tend to fluctuate year by year; thus, it may be possible to obtain a more stable pattern by excluding such foodstuffs.

On the other hand, the FIES includes a limited number of purchased food items, and there are discrepancies in the dietary survey items targeted at individuals. For example, regarding the intake of beans, which are characteristic of Japanese food, only “tofu” is subject to a weight survey. In addition, foods such as tea and alcohol are often purchased as gifts; thus, the values obtained may not reflect intake.

In addition, in the analysis using the average value for each city, the purchase pattern at the individual level was smoothed, and the food pattern phenomenon occurring at the microlevel was offset. Therefore, while household surveys can help develop data that are representative of dietary choices across a wide range of countries, the insights derived from them do not directly apply to individuals.

In conclusion, we used the FIES data to identify three Japanese dietary patterns with high salt intake (traditional, urban, and local) and confirmed that this method was effective and reproducible. While household survey data represent food availability rather than consumption, this method is useful for the long-term representation of dietary options across a wide region of the country. Therefore, FIES data could help to identify a variety of food choices and allow the appropriate planning of broad-based nutrition advisory activities [18].

## 5. Conclusions

The salt intake of Japanese people is decreasing annually. It is also considered that the dietary patterns of Japanese people are gradually changing. However, the traditional dietary pattern is still the main dietary pattern of the Japanese, and it is expected that it will be inherited in the future. This pattern is thought to have formed over a long period of time because of the influence of environmental factors, such as local climate. In the future, in order to achieve further salt reduction, it will be important to consider these characteristics and provide nutritional advice while taking advantage of traditional dietary patterns. The results of this study suggest that Japanese salt intake is high due to regionally specific factors. Therefore, FIES data could help to identify a variety of food choices and allow the appropriate planning of broad-based nutrition advisory activities.

## Figures and Tables

**Table 1:** Basic statistics for the 52 cities in FIES 2013 and 2018.

	No. of persons per household (persons)		Age of household heads (years)		Annual total consumption expenditure (yen)	
	2013	2018	2013	2018	2013	2018
All Japan	3.05	2.98	57.9	59.3	3,485,454	3,447,782
Maximum	3.37	3.30	61.5	63.5	4,036,707	4,070,101
Minimum	2.82	2.72	53.8	54.6	2,875,848	2,698,241

**Table 2:** Spearman's correlation coefficients for the purchase rankings of each city for each of the 109 foods in 2015 and 2018.

Food	*r*	*t*	*p*
Rice	0.39	2.96	0.0049
White bread	0.80	9.27	<0.0001
Spaghetti	0.38	2.95	0.0050
Wheat flour	0.29	2.12	0.0396
Tuna fish	0.94	18.75	<0.0001
Sardines	0.79	9.21	<0.0001
Flounder	0.93	18.08	<0.0001
Salmon	0.70	6.85	<0.0001
Mackerel	0.60	5.33	<0.0001
Saury	0.76	8.15	<0.0001
Cuttlefish	0.55	4.62	<0.0001
Shrimps and lobsters	0.66	6.17	<0.0001
Crabs	0.54	4.56	<0.0001
Scallops	0.66	6.27	<0.0001
Salted salmon	0.90	14.52	<0.0001
Salted pollock roe	0.84	11.01	<0.0001
Dried young sardines	0.88	13.34	<0.0001
Dried horse mackerel	0.89	13.91	<0.0001
Beef	0.91	15.64	<0.0001
Pork	0.73	7.54	<0.0001
Chicken	0.84	10.90	<0.0001
Mixed ground meat	0.84	11.06	<0.0001
Fresh milk	0.62	5.52	<0.0001
Butter	0.61	5.46	<0.0001
Cheese	0.83	10.69	<0.0001
Eggs	0.61	5.39	<0.0001
Spinach	0.79	8.99	<0.0001
Welsh onions	0.87	12.70	<0.0001
Lettuce	0.66	6.17	<0.0001
Broccoli	0.66	6.26	<0.0001
Bean sprouts	0.72	7.29	<0.0001
Radishes	0.70	6.95	<0.0001
Bamboo shoots	0.37	2.84	<0.0068
String beans	0.75	8.10	<0.0001
Tomatoes	0.54	4.53	<0.0001
Green peppers	0.60	5.34	<0.0001
“Wakame” seaweed	0.65	6.00	<0.0001
Dried tangle	0.51	4.14	<0.0001
“Tofu” bean curd	0.70	6.96	<0.0001
Pickled radishes	0.45	3.55	0.0009
Apples	0.58	5.04	<0.0001
Grapefruits	0.64	5.95	<0.0001
Melons	0.41	3.22	0.0024
Kiwi fruits	0.34	2.54	0.0145
Edible oil	0.55	4.68	<0.0001
Salt	0.29	2.16	0.0364
Soy sauce	0.31	2.32	0.0249
“Miso” bean paste	0.66	6.27	<0.0001
Sugar	0.45	3.53	0.0010
Dressing	0.58	5.07	<0.0001
Jam	0.51	4.23	0.0001
Green tea	0.66	6.22	<0.0001
Black tea	0.36	2.74	0.0089
“Sake”	0.56	4.80	<0.0001
Wine	0.58	5.07	<0.0001
Alcoholic beverages	0.52	4.33	0.0001

**Table 3:** Explained variations in response variables (total salt consumption) and food groups.

2013	Factor 1	Factor 2	Factor 3	Factor 4	Factor 5
Proportion (%) of explained variation in food groups	14.20	25.33	29.60	33.48	38.04

Proportion (%) of explained variation in response	73.42	86.45	95.05	97.53	98.71

2018					

Proportion (%) of explained variation in food groups	14.76	24.37	29.09	35.53	41.56

Proportion (%) of explained variation in response	57.33	78.80	92.41	95.47	97.55

Factors 1–5: number of components (loading factors).

**Table 4:** Factor loadings of food groups in dietary patterns identified using PLS regression analysis.

Foods	Factor 1 2013	Factor 1 2018	Factor 2 2013	Factor 2 2018	Factor 3 2013	Factor 3 2018
Rice						−0.11
White bread	−0.11					
Spaghetti		0.11	−0.17	−0.15		0.10
Wheat flour					0.12	0.20

Tuna fish			−0.21	−0.20	0.13	0.19
Sardines				0.14	−0.23	−0.18
Flounder	0.15	0.16			−0.18	−0.22
Salmon	0.20	0.18	−0.13	−0.10	0.03	
Mackerel			0.14	0.14	−0.17	−0.14
Saury	0.20	0.19			0.00	
Cuttlefish	0.22	0.18			−0.10	−0.17
Shrimps and lobsters					−0.18	−0.17
Crabs		0.10			−0.21	−0.13
Scallops	0.20	0.16				
Salted salmon	0.22	0.23				
Salted pollock roe	0.19	0.18				
Dried young sardines			−0.11	−0.11		0.19
Dried horse mackerel			−0.14			0.21

Beef	−0.12	−0.13	0.13			−0.11
Pork	0.14	0.14	−0.19	−0.15		
Chicken						−0.14
Mixed ground meat	−0.16	−0.15	0.15	0.11		

Fresh milk		0.10			0.13	0.15
Butter			−0.19	−0.20	0.15	0.10
Cheese			−0.25	−0.26	0.18	0.17

Eggs			0.17	0.12	−0.11	−0.20

Spinach	0.18	0.19		−0.17	0.13	
Welsh onions	0.14	0.19	−0.22	−0.20		
Lettuce			−0.24	−0.16		0.23
Broccoli	0.13	0.14	−0.21	−0.20		0.16
Bean sprouts	0.22	0.23				
Radishes	0.15	0.16	−0.19	−0.21		
Bamboo shoots				−0.11	0.28	
String beans	0.11	0.13	−0.14	−0.18		
Tomatoes			−0.19	−0.20		
Green peppers			−0.17	−0.22		

“Wakame,” seaweed	0.18	0.20				
Dried tangle	0.18	0.18			0.14	

“Tofu,” bean curd	0.14					

Pickled radishes	0.16	0.15				−0.17
Apples	0.20	0.21				
Grapefruits	0.12	0.14	−0.20	−0.19		
Melons	0.11	0.11			−0.22	
Kiwi fruits			−0.16	−0.19		

Edible oil				0.14	−0.22	−0.11

Salt	0.21	0.14	0.12	0.20	0.16	0.23
Soy sauce		0.13	0.16			
“Miso,” soybean paste	0.18	0.12		0.12		

Sugar			0.16	0.19	−0.14	
Dressing						0.20
Jam			−0.12	−0.10	0.18	0.16

Green tea				−0.10		
Black tea			−0.12	−0.19	0.20	0.15

“Sake”	0.14	0.18				
Wine		0.12	−0.18	−0.20	0.15	
Alcoholic beverages						−0.16

Spearman's correlation coefficient between 2013 and 2018	0.84^*∗∗∗*^		0.88^*∗∗∗*^		0.63^*∗∗∗*^	

Family Income and Expenditure Survey 2013/2018. Factor loadings less than ±0.10 are not listed. Spearman's correlation coefficient was calculated using the values of all 109 foods. ^*∗∗∗*^*p* < 0.0001.

**Table 5:** Loading scores of cities in dietary patterns (Factors 1 to 3) identified using partial least squares regression analysis.

No.	Name of a city	Factor 1 2015	Factor 1 2018	Factor 2 2013	Factor 2 2018	Factor 3 2013	Factor 3 2018
1	Sapporo-shi	3.62	1.14	−3.29	−4.46	−2.96	−4.29
2	Aomori-shi	11.36	11.78	−0.84	4.55	−3.83	−1.16
3	Morioka-shi	6.67	8.49	1.32	−0.50	2.61	0.08
4	Sendai-shi	4.59	3.05	−1.66	−2.63	2.12	0.11
5	Akita-shi	9.33	7.90	1.12	−1.89	−1.36	−4.79
6	Yamagata-shi	7.70	4.57	5.39	2.06	6.88	1.40
7	Fukushima-shi	4.45	1.60	1.61	−1.14	1.18	−2.49
8	Mito-shi	−0.55	2.24	−3.57	−0.66	−1.51	0.20
9	Utsunomiya-shi	−0.06	0.23	−2.18	−1.73	−1.30	1.30
10	Maebashi-shi	−1.49	1.20	−2.42	−0.35	0.22	2.00
11	Saitama-shi	−0.99	1.02	−4.53	−3.74	1.73	2.15
12	Chiba-shi	1.64	1.44	−5.23	−4.89	0.03	0.76
13	Ku-areas of Tokyo	−2.03	−1.60	−6.13	−5.15	0.61	1.32
14	Yokohama-shi	−0.64	−0.58	−7.02	−4.00	1.40	2.17
15	Niigata-shi	8.94	7.92	−1.62	−1.25	−1.07	−0.47
16	Toyama-shi	2.84	3.96	−0.16	−0.15	−0.37	−1.34
17	Kanazawa-shi	−0.28	1.05	0.20	1.89	−0.20	1.12
18	Fukui-shi	−1.26	−1.93	3.21	−0.63	−0.04	−2.91
19	Kofu-shi	1.01	−0.49	−3.31	0.61	0.40	3.98
20	Nagano-shi	2.39	5.35	0.29	5.73	2.51	7.45
21	Gifu-shi	−2.55	−1.66	0.39	−1.79	−1.18	−0.58
22	Shizuoka-shi	−0.16	−0.64	−4.25	−2.77	0.11	2.43
23	Nagoya-shi	−2.29	−1.61	−2.37	−1.97	−0.50	1.14
24	Tsu-shi	−2.40	−0.86	1.87	−0.44	0.75	−1.56
25	Otsu-shi	−4.52	1.31	−0.33	−1.13	1.51	0.57
26	Kyoto-shi	−1.80	−0.66	−0.88	−2.48	3.53	0.42
27	Osaka-shi	−4.26	−1.47	−0.71	−0.80	0.61	−0.74
28	Kobe-shi	−4.72	−4.75	0.11	−1.67	0.92	0.39
29	Nara-shi	−1.43	−0.24	0.54	−1.87	1.18	−0.17
30	Wakayama-shi	−3.76	−2.03	0.93	2.59	−1.32	0.42
31	Tottori-shi	2.29	2.43	3.26	4.22	−4.92	−3.03
32	Matsue-shi	2.55	0.94	4.62	−0.05	−0.08	−2.51
33	Okayama-shi	−4.19	−2.41	1.09	3.45	−1.38	3.09
34	Hiroshima-shi	−1.55	−1.08	−0.05	2.29	−1.41	0.10
35	Yamaguchi-shi	−1.55	−0.47	3.70	3.20	−1.81	−1.96
36	Tokushima-shi	−1.68	−4.09	1.75	1.76	0.35	−0.75
37	Takamatsu-shi	−3.18	−1.46	2.54	3.01	0.05	0.49
38	Matsuyama-shi	−3.64	−3.31	3.11	3.71	−1.13	−0.20
39	Kochi-shi	−1.22	−3.09	6.63	2.30	2.60	−0.75
40	Fukuoka-shi	−3.06	−3.77	0.16	−0.94	0.46	−0.48
41	Saga-shi	2.00	−0.63	2.54	1.77	−2.88	−1.48
42	Nagasaki-shi	−1.81	−2.07	1.32	1.26	−2.26	−1.19
43	Kumamoto-shi	−1.99	−4.39	2.78	3.41	−1.26	−1.43
44	Oita-shi	0.09	−1.22	5.65	3.57	1.62	−0.99
45	Miyazaki-shi	−3.01	−3.60	3.47	4.66	−0.87	0.05
46	Kagoshima-shi	−2.39	−3.68	1.84	3.77	−2.06	0.06
47	Naha-shi	−6.05	−8.74	0.10	−3.18	0.80	−1.15
48	Kawasaki-shi	−0.42	−0.41	−6.40	−4.47	1.45	2.40
49	Sagamihara-shi	1.09	2.02	−3.83	−3.84	1.07	1.60
50	Hamamatsu-shi	−1.62	−1.80	−1.77	−0.37	−1.61	1.44
51	Sakai-shi	−0.24	−2.77	0.16	−0.67	2.72	−0.19
52	Kitakyushu-shi	0.45	−1.92	1.53	1.76	−2.06	−2.60
Spearman's correlation coefficient between 2013 and 2018		0.77^*∗∗∗*^		0.71^*∗∗∗*^		0.38^*∗*^	

Family Income and Expenditure Survey in 2013/2018. ^*∗∗∗*^*p* < 0.0001; ^*∗*^*p* < 0.01. Numbers 1 to 47 are prefectural capitals and 48 to 52 are ordinance-designated cities. The numbers correspond to those in Supporting Figure S1 (distribution map of prefectures in Japan).

**Table 6:** Spearman's correlation coefficient between the loading scores of 52 cities and nutrient consumption calculated from purchase amount.

2013	Energy	Protein	Fat	Carbohydrates	Dietary fiber	Salt
Factor 1	0.36	0.47^*∗*^	0.13	0.35	0.68^*∗∗*^	0.81^*∗∗*^

Factor 2	0.01	−0.15	0.13	−0.03	−0.41^*∗*^	0.31

Factor 3	−0.09	0.01	−0.22	0.01	0.25	0.13

2018						

Factor 1	0.48^*∗*^	0.54^*∗∗*^	0.24	0.47^*∗*^	0.61^*∗∗*^	0.61^*∗∗*^

Factor 2	−0.03	−0.18	0.14	−0.14	−0.48^*∗*^	0.40^*∗*^

Factor 3	−0.08	−0.03	−0.17	0.03	0.21	0.27

^*∗∗*^ *p* < 0.0001; ^*∗*^*p* < 0.005.

## Data Availability

All the data used in this study are provided in the Supplementary Materials file.

## Supplementary Materials

Supplementary Materials Supporting File S1: details of 109 foods and calculation units used for nutritional value (data for 2013). Supporting File S2: details of 109 foods and calculation units used for nutritional value (data for 2018). Figure S1: the distribution map of prefectures in Japan.

## References

[1] Kumakura I., Receives W., Recognition O. (2013). Washoku Joins UNESCO’ s Intangible Cultural Heritage List. https://www.kikkoman.co.jp/kiifc/foodculture/pdf_24/e_003_005.pdf.

[2] Kohsaka R. (2017). The myth of washoku: a twisted discourse on the “uniqueness” of national food heritages. *Journal of Ethnic Foods*.

[3] Ministry of Agriculture Forestry and Fisheries WASHOKU Traditional Dietary Cultures of the Japanese. https://www.maff.go.jp/e/data/publish/attach/pdf/index-20.pdf.

[4] Suzuki N., Goto Y., Ota H. (2018). Characteristics of the Japanese diet described in epidemiologic publications: a qualitative systematic review. *Journal of Nutritional Science and Vitaminology*.

[5] Niu K., Momma H., Kobayashi Y. (2016). The traditional Japanese dietary pattern and longitudinal changes in cardiovascular disease risk factors in apparently healthy Japanese adults. *European Journal of Nutrition*.

[6] Gabriel A. S., Ninomiya K., Uneyama H. (2018). The role of the Japanese traditional diet in healthy and sustainable dietary patterns around the world. *Nutrients*.

[7] Tsugane S., Sawada N. (2014). The JPHC study: design and some findings on the typical Japanese diet. *Japanese Journal of Clinical Oncology*.

[8] Kawamura A., Kajiya K., Kishi H. (2018). The nutritional characteristics of the hypotensive WASHOKU-modified dash diet: a sub-analysis of the DASH-JUMP study. *Current Hypertension Reviews*.

[9] Sasaki N. (1962). High blood pressure and the salt intake of the Japanese. *Japanese Heart Journal*.

[10] Asakura K., Uechi K., Masayasu S., Sasaki S. (2016). Sodium sources in the Japanese diet: difference between generations and sexes. *Public Health Nutrition*.

[11] Shimamoto K. (2014). The Japanese Society of Hypertension guidelines for the management of hypertension (JSH 2014). *Hypertension Research*.

[12] Ministry of Health Labour and Welfare (2018). Dietary Reference Intakes for Japanese (2015). https://www.mhlw.go.jp/file/06-Seisakujouhou-10900000-Kenkoukyoku/Full_DRIs2015.pdf.

[13] Ministry of Health Labour and Welfare (2016). Summary of the National Health and Nutrition Survey 2015. http://www.mhlw.go.jp/stf/houdou/0000142359.html.

[14] Statistics Bureau of Japan Family Income and Expenditure Survey. https://www.stat.go.jp/data/kakei/rank/singleyear.html.

[15] Chern W. ., Ishibashi K., Taniguchi K., Yokoyama Y. (2002). Analysis of food consumption behavior by Japanese households. *ESA Working Paper*.

[16] Wessells C. R., Wilen J. E. (1994). Seasonal patterns and regional preferences in Japanese household demand for seafood. *Canadian Journal of Agricultural Economics/Revue Canadienne D’agroeconomie*.

[17] Naska A., Fouskakis D., Oikonomou E. (2006). Dietary patterns and their socio-demographic determinants in 10 European countries: data from the DAFNE databank. *European Journal of Clinical Nutrition*.

[18] Nascimento S., Barbosa F. S., Sichieri R., Pereira R. A. (2011). Dietary availability patterns of the brazilian macro-regions. *Nutrition Journal*.

[19] Hoffmann K., Schulze M. B., Schienkiewitz A., Nöthlings U., Boeing H. (2004). Application of a new statistical method to derive dietary patterns in nutritional epidemiology. *American Journal of Epidemiology*.

[20] Naja F., Itani L., Hwalla N., Sibai A. M., Kharroubi S. A. (2019). Identification of dietary patterns associated with elevated blood pressure among Lebanese men: a comparison of principal component analysis with reduced rank regression and partial least square methods. *PLoS One*.

[21] Batis C., Mendez M. A., Gordon-Larsen P., Sotres-Alvarez D., Adair L., Popkin B. (2016). Using both principal component analysis and reduced rank regression to study dietary patterns and diabetes in Chinese adults. *Public Health Nutrition*.

[22] Yang T. C., Aucott L. S., Duthie G. G., Macdonald H. M. (2017). An application of partial least squares for identifying dietary patterns in bone health. *Archives of Osteoporosis*.

[23] Mevik B.-H., Wehrens R. (2015). Introduction to the pls package. *Help Sect. “Pls” Packag. RStudio Softw.*.

[24] Wehrens R., Hovde K., Hiemstra P. (2020). Package ‘ pls. https://cran.r-project.org/web/packages/pls/pls.pdf.

[25] R Foundation for Statistical Computing. http://www.r-project.org.

[26] Nanri A., Shimazu T., Ishihara J. (2012). Reproducibility and validity of dietary patterns assessed by a food frequency questionnaire used in the 5 year follow-up survey of the Japan public health center-based prospective study. *Journal of Epidemiology*.

[27] Murakami K., Livingstone M. B. E., Sasaki S. (2018). Thirteen-year trends in dietary patterns among Japanese adults in the national health and nutrition survey 2003–2015: continuous westernization of the Japanese diet. *Nutrients*.

[28] Konishi M., Nagashima K., Kanosue K. (2002). Systemic salt loading decreases body temperature and increases heat‐escape/cold‐seeking behaviour via the central AT 1 and V 1 receptors in rats. *The Journal of Physiology*.

[29] Muller M. D., Ryan E. J., Bellar D. M. (2011). Effect of acute salt ingestion upon core temperature in healthy men. *Hypertension Research*.

[30] Gizowski C., Bourque C. W. (2020). Sodium regulates clock time and output via an excitatory GABAergic pathway. *Nature*.

[31] Fangfang S., Mi S. C. (2017). Geography of food consumption patterns between south and north China. *Foods*.

[32] Zarei N., Ahmadi A. (2015). Nutrition transition: an intergenerational comparison of dietary habits among women of Shiraz. *Iranian Journal Public Health*.

[33] Bezerra I. N., Bahamonde N. M. S. G., Marchioni D. M. L. (2018). Generational differences in dietary pattern among Brazilian adults born between 1934 and 1975: a latent class analysis. *Public Health Nutrition*.

[34] Ridoutt B., Baird D., Bastiaans K. (2016). Changes in food intake in Australia: comparing the 1995 and 2011 national nutrition survey results disaggregated into basic foods. *Foods*.

[35] Ministry of Health Labour and Welfare (2016). Graphical review of Japanese household. From comprehensive survey of living conditions. https://www.mhlw.go.jp/toukei/list/dl/20-21-h28_rev2.pdf.

[36] Yasuyuki S. (2001). Regional characteristics and intergenerational differences in meat consumption. *Journal of Rural Problems*.

