# Supplementary material for: Identification of Salty Dietary Patterns of the Japanese Macroregion
Source: J Nutr Metab. 2021 Jul 22;2021:6675418. doi: 10.1155/2021/6675418 (PMC8321765; doi:10.1155/2021/6675418)
Supplement: Supplementary Materials — Supporting File S1: details of 109 foods and calculation units used for nutritional value (data for 2013). Supporting File S2: details of 109 foods and calculation units used for nutritional value (data for 2018). Figure S1: the distribution map of prefectures in Japan. [file 6675418.f1.zip › 6675418.f1/Supporting file S1.pdf]

（品目分類） 第4表　都市階級・地方・都道府県庁所在市別１世帯当たり年間の品目別支出金額，購入数量（二人以上の世帯）

(Commodity Classification) Table 4 Yearly Amount of Expenditures and Quantities per Household by City Group,District,
Major Metropolitan Area and City with Prefectural Government (Two-or-more-person Households)

平成25年　2013

都市階級 地方 都道府県庁所在市	City group / District / Major metropolitan area / City with prefectural governmer	世帯数分布（抽出率調整） Distribution of households	集計世帯数 Num. of tabulated households	世帯人員（人） Num. of persons per household (persons)	有業人員（人） Num. of earners per household (persons)	世帯主の年齢（歳） Age of household heads (years old)	102～981 消費支出 Consumption expenditures 金額 Exp.	102～398・39A・39B・39X 食料 Food 金額 Exp.	1 米 Rice 1kg 数量 Quan.	2 食パン White bread 1g 数量 Quan.	3 生うどん・そ Non-dried "Udon"&"S 1g 数量 Quan.	4 乾うどん・そ Dried "Udon"&"S 1g 数量 Quan.	5 スパゲッティ Spaghetti 1g 数量 Quan.	6 中華麺 Chinese noo 1g 数量 Quan.	7 小麦粉 Wheat flour 1g 数量 Quan.	8 もち "Mochi", ric 1g 数量 Quan.	9 まぐろ Tuna fish 1g 数量 Quan.	10 あじ Horse mack 1g 数量 Quan.	11 いわし Sardines 1g 数量 Quan.	12 かつお Bonito 1g 数量 Quan.	13 かれい Flounder 1g 数量 Quan.	14 さけ Salmon 1g 数量 Quan.	15 さば Mackerel 1g 数量 Quan.	16 さんま Saury 1g 数量 Quan.	17 たい Sea bream 1g 数量 Quan.	18 ぶり Yellowtail 1g 数量 Quan.	19 いか Cuttlefish 1g 数量 Quan.	20 たこ Octopus 1g 数量 Quan.
全　国	All Japan	10,000	7,784	3.05	1.34	57.9	3,485,454	895,860	75.17	19,636	10,555	4,286	3,224	8,865	2,817	2,437	2,369	1,087	737	1,002	1,038	3,058	1,184	1,342	505	2,169	2,306	789
札幌市	Sapporo-shi	151	94	2.91	1.26	56.6	3,350,286	833,142	85.54	14,798	9,097	4,089	4,086	9,068	2,072	2,351	2,085	169	534	525	2,197	4,749	719	2,692	65	822	3,509	1,090
青森市	Aomori-shi	23	95	3.17	1.33	58.3	2,957,346	806,779	88.09	18,103	10,531	3,589	3,467	12,055	2,731	1,784	2,268	708	1,493	1,152	3,312	4,652	1,708	3,142	257	1,609	4,602	685
盛岡市	Morioka-shi	22	95	3.15	1.28	59.9	3,316,984	850,737	75.94	16,349	10,360	7,923	3,084	10,888	3,512	2,135	2,382	573	456	1,863	1,581	4,218	868	2,847	180	1,718	2,515	554
仙台市	Sendai-shi	76	93	2.97	1.12	59.8	3,473,145	928,604	64.94	16,378	9,541	6,662	2,881	8,574	2,154	2,760	2,979	448	579	2,305	1,545	3,581	694	2,205	105	1,429	2,731	875
秋田市	Akita-shi	25	93	2.95	1.22	59.9	3,274,371	847,368	80.17	12,701	10,730	5,818	2,951	9,902	2,640	1,242	2,269	731	992	1,074	2,087	3,832	1,449	2,926	979	2,028	3,532	524
山形市	Yamagata-shi	19	94	3.29	1.54	58.9	3,778,583	935,002	85.11	14,593	10,838	6,775	3,165	10,539	2,684	1,986	2,441	340	557	1,395	1,089	3,766	968	2,085	207	1,841	2,854	542
福島市	Fukushima-shi	22	94	2.98	1.42	59.4	3,290,341	844,919	80.83	13,044	10,296	4,639	3,354	7,297	2,370	2,158	2,834	299	343	2,306	1,244	3,811	819	1,672	229	1,381	2,686	510
水戸市	Mito-shi	21	94	2.92	1.21	57.2	3,482,624	869,195	55.66	16,528	8,607	4,762	3,337	7,680	2,261	1,922	2,754	804	290	2,395	900	3,190	715	1,663	304	1,834	1,579	791
宇都宮市	Utsunomiya-shi	40	94	3.06	1.39	56.3	3,704,386	907,846	62.22	18,551	10,377	4,780	3,290	8,658	2,584	2,171	3,215	505	528	1,174	678	3,248	1,011	1,434	111	1,510	1,910	580
前橋市	Maebashi-shi	27	92	3.00	1.38	56.7	3,428,762	855,697	72.72	15,278	11,265	5,977	3,245	8,825	3,085	2,164	3,591	942	432	676	429	3,780	860	1,045	85	1,066	2,057	507
さいたま市	Saitama-shi	100	94	3.05	1.34	55.5	3,704,840	966,689	71.01	19,781	10,796	4,744	4,829	9,424	3,249	2,997	3,093	893	435	838	655	3,180	925	1,225	240	1,666	1,700	783
千葉市	Chiba-shi	79	93	2.89	1.05	61.4	3,362,073	946,533	67.87	19,916	8,449	3,695	3,691	9,259	2,469	2,765	3,674	1,755	1,416	1,548	800	3,376	1,296	1,807	304	2,110	2,339	639
東京都区部	Ku-areas of Tokyo	671	386	2.97	1.36	57.2	3,999,967	1,055,450	62.58	19,190	8,627	2,875	4,102	8,219	2,102	2,678	2,665	1,000	620	1,006	653	2,959	753	1,137	289	1,841	1,780	773
横浜市	Yokohama-shi	300	129	3.04	1.26	58.1	3,714,437	998,500	73.68	21,480	9,956	4,033	4,016	9,364	2,965	3,094	3,800	1,237	690	873	752	3,055	1,040	1,319	287	2,152	2,619	814
新潟市	Niigata-shi	62	94	3.18	1.45	58.2	3,331,097	913,143	89.20	17,325	8,293	5,448	2,910	7,098	3,073	3,285	1,602	1,350	1,405	752	2,545	4,358	1,488	1,992	376	2,467	3,394	606
富山市	Toyama-shi	32	95	3.20	1.62	55.7	3,884,362	938,908	68.89	22,144	11,318	6,164	2,720	8,852	2,305	3,053	2,620	1,982	879	523	1,222	2,856	1,077	1,160	253	7,052	2,654	495
金沢市	Kanazawa-shi	34	96	3.24	1.57	53.8	3,893,213	943,651	69.76	22,039	15,277	2,724	3,222	8,479	3,204	2,342	1,230	1,174	1,188	431	2,165	2,639	1,112	1,103	406	3,903	2,044	556
福井市	Fukui-shi	20	95	3.37	1.68	57.6	3,445,098	852,421	93.82	17,321	9,829	2,902	2,524	7,433	2,112	2,528	920	618	556	689	1,877	2,652	847	668	139	2,505	2,243	743
甲府市	Kofu-shi	16	91	3.15	1.40	57.8	3,261,483	895,817	80.56	15,841	11,136	3,721	3,368	9,624	1,853	1,452	4,728	811	531	509	626	4,241	1,137	1,245	224	1,546	2,460	671
長野市	Nagano-shi	31	94	2.91	1.42	58.1	3,499,712	883,014	63.75	17,026	11,763	4,945	3,009	8,206	4,832	1,785	2,717	456	444	656	1,020	3,963	1,123	1,498	233	1,719	1,741	657
岐阜市	Gifu-shi	32	95	3.14	1.29	57.4	3,264,469	828,971	90.02	19,068	8,636	3,229	3,304	9,873	3,001	2,843	2,236	536	441	762	882	2,769	922	1,655	274	1,589	2,167	599
静岡市	Shizuoka-shi	56	96	3.08	1.35	60.8	3,682,165	957,839	95.63	21,400	8,551	3,110	2,752	8,314	2,561	2,494	5,405	1,176	889	1,905	356	3,050	1,381	1,244	138	1,608	2,235	630
名古屋市	Nagoya-shi	172	106	3.00	1.33	58.6	3,678,713	962,435	79.93	23,179	11,741	3,048	2,704	9,638	3,625	3,115	2,881	707	653	772	463	2,390	1,016	1,934	439	1,924	2,040	698
津市	Tsu-shi	23	83	3.13	1.36	58.6	3,475,233	871,740	84.66	20,390	11,989	3,019	3,057	9,454	2,991	2,148	2,903	1,113	981	1,202	531	2,488	1,244	1,728	261	2,268	1,820	855
大津市	Otsu-shi	27	92	3.04	1.10	56.2	3,255,439	872,073	71.82	24,680	11,157	3,367	3,410	9,419	3,912	2,224	1,347	578	878	661	1,318	2,699	889	1,112	633	2,466	1,892	809
京都市	Kyoto-shi	111	94	3.11	1.28	61.5	3,712,602	1,030,444	81.68	26,701	15,861	6,124	3,452	9,017	3,710	2,687	1,259	830	657	939	1,842	2,891	1,038	1,022	701	2,350	1,971	1,114
大阪市	Osaka-shi	197	128	3.10	1.39	56.0	3,273,759	917,699	69.71	23,397	13,465	3,302	3,128	9,519	2,690	2,494	1,612	953	562	793	1,185	2,577	1,286	944	453	2,156	1,935	1,171
神戸市	Kobe-shi	123	92	2.92	1.20	60.0	3,204,578	897,358	65.91	26,682	13,052	4,766	2,833	8,895	2,667	2,170	1,248	1,065	839	1,012	1,143	2,428	1,152	756	888	2,357	2,374	905
奈良市	Nara-shi	30	95	3.04	1.13	58.9	3,694,550	927,190	90.65	24,575	13,261	3,742	3,448	9,384	4,856	2,267	1,487	994	823	1,022	1,276	2,423	1,314	1,280	922	2,078	1,960	880
和歌山市	Wakayama-shi	30	93	2.99	1.18	58.1	3,101,569	846,596	68.88	21,073	14,430	3,579	2,637	9,236	3,392	1,953	2,108	1,031	765	1,512	1,028	2,705	1,795	856	803	2,775	2,039	834
鳥取市	Tottori-shi	15	93	3.29	1.60	56.7	3,193,385	854,373	66.54	22,274	11,645	4,019	3,139	7,960	3,319	2,079	867	1,481	2,264	633	4,454	2,278	1,678	1,143	288	3,145	3,319	706
松江市	Matsue-shi	16	92	3.11	1.35	58.4	3,559,562	858,481	74.97	24,958	10,392	3,583	3,633	7,513	2,339	3,089	524	3,273	1,753	569	2,756	2,892	2,059	1,257	1,118	3,706	2,762	461
岡山市	Okayama-shi	54	88	3.11	1.36	53.8	3,477,486	820,969	53.71	21,262	12,939	3,724	3,329	10,374	3,082	1,323	854	1,095	403	887	1,041	2,527	1,114	1,122	929	3,052	1,985	933
広島市	Hiroshima-shi	91	91	2.88	1.18	58.9	3,524,295	899,957	71.90	19,240	13,457	5,579	2,374	8,154	3,044	1,670	834	1,356	1,140	802	1,141	2,616	1,517	1,538	1,113	2,830	1,838	1,062
山口市	Yamaguchi-shi	15	94	3.11	1.39	57.3	3,416,268	830,530	66.15	19,868	11,042	2,412	2,537	7,006	3,311	1,648	571	2,321	1,069	737	1,642	2,994	1,370	1,184	1,503	3,286	2,007	660
徳島市	Tokushima-shi	20	93	3.14	1.42	56.8	3,702,603	879,252	76.27	20,885	11,342	3,979	2,824	6,868	2,333	2,400	1,754	987	406	1,214	397	2,156	922	1,091	532	2,960	2,076	548
高松市	Takamatsu-shi	32	94	2.93	1.33	57.5	3,828,604	867,157	66.12	23,156	20,243	7,838	2,306	7,285	2,807	1,518	722	1,475	483	1,123	953	2,496	1,306	1,143	1,062	3,053	1,688	1,211
松山市	Matsuyama-shi	40	94	3.11	1.10	54.2	3,317,862	825,158	78.01	21,096	13,497	3,398	3,700	8,155	2,789	1,684	720	1,257	610	975	486	2,420	1,550	1,697	817	2,052	1,363	701
高知市	Kochi-shi	26	92	2.99	1.47	55.7	3,432,229	857,310	67.53	21,534	10,596	2,641	2,678	5,335	3,707	1,686	2,543	1,226	938	4,957	336	1,541	1,818	1,047	366	2,840	2,186	601
福岡市	Fukuoka-shi	106	90	3.13	1.34	54.1	3,454,756	860,101	76.41	18,824	7,303	2,840	4,160	8,106	3,107	1,688	523	1,588	773	465	881	2,451	1,430	893	1,018	2,738	1,667	569
佐賀市	Saga-shi	18	95	3.10	1.31	59.5	3,535,017	906,231	106.97	19,708	9,404	4,310	3,163	8,432	1,982	1,933	782	2,671	1,368	641	1,778	3,099	1,351	990	2,065	3,203	2,317	513
長崎市	Nagasaki-shi	35	94	3.00	1.25	59.6	3,356,140	844,029	87.63	16,700	7,376	2,424	3,362	8,611	2,500	2,114	517	2,346	1,118	597	646	2,495	1,246	731	1,137	2,715	1,799	394
熊本市	Kumamoto-shi	57	95	3.09	1.33	55.6	3,245,645	803,448	81.45	17,044	6,784	3,345	3,392	6,550	3,105	1,896	1,064	1,242	695	630	583	2,006	1,164	706	1,187	2,031	1,662	569
大分市	Oita-shi	38	95	3.02	1.43	57.0	3,741,885	856,704	76.15	16,195	9,441	2,759	3,318	6,435	4,118	1,289	910	2,368	1,052	425	1,025	2,755	1,592	1,187	1,484	3,113	1,680	762
宮崎市	Miyazaki-shi	32	91	3.01	1.25	56.5	3,165,678	787,322	59.87	15,620	8,147	2,336	2,609	5,759	3,346	1,445	1,341	2,250	986	1,461	343	1,977	1,377	683	503	1,874	1,679	582
鹿児島市	Kagoshima-shi	48	94	3.14	1.34	55.0	3,609,197	850,180	63.57	15,623	8,640	3,701	3,055	7,104	3,216	1,226	1,073	1,593	873	823	319	2,537	1,887	695	698	2,238	1,716	615
那覇市	Naha-shi	25	161	3.34	1.33	55.8	2,875,848	747,992	82.78	16,620	4,531	3,930	3,163	10,772	2,813	1,789	3,436	154	103	678	443	2,873	885	1,003	430	700	830	283
令	(f)																											
川崎市	Kawasaki-shi	110	92	3.09	1.30	54.9	4,036,707	1,036,514	69.32	21,525	12,112	4,532	4,872	10,115	2,080	2,359	3,029	1,093	658	923	578	3,261	1,069	1,264	274	2,258	2,230	834
相模原市	Sagamihara-shi	58	90	3.09	1.32	56.5	3,541,139	900,686	80.63	23,341	11,255	4,563	4,508	9,902	3,347	2,784	3,158	1,318	637	718	926	3,732	1,140	1,486	318	1,957	1,687	637
浜松市	Hamamatsu-shi	62	93	3.36	1.56	55.6	3,668,486	922,973	94.92	18,989	10,821	3,071	3,188	9,567	2,828	1,753	3,911	837	668	2,032	234	3,127	1,511	1,433	384	1,655	1,976	647
堺市	Sakai-shi	68	93	3.01	1.14	59.0	3,661,131	960,592	82.55	24,418	14,906	3,101	2,656	8,652	2,536	2,654	1,721	808	750	1,083	1,091	2,886	1,323	1,239	891	2,377	2,479	1,145
北九州市	Kitakyushu-shi	77	93	2.82	1.14	60.9	3,310,036	845,204	77.79	15,390	9,166	3,763	2,443	6,761	2,207	2,243	674	1,962	1,370	431	1,715	2,717	1,914	745	2,187	3,168	2,001	617

(a) Cities with population of 50,000 or more
(b) Small cities B, towns & villages
(c) Major metropolitan area
(d) Kitakyushu & Fukuoka
(e) City with prefectural government
(f) Designated cities under article 252-19 of the Local Autonomy Act excluding cities with prefectural governments

（注）1. この表には川崎市，相模原市，浜松市，堺市及び北九州市も掲載されている。
2. 都道府県庁所在市及び政令指定都市の地域は，2012年4月1日現在の区域による。

Notes: 1.Kawasaki-shi, Sagamihara-shi, Hamamatsu-shi, Sakai-shi and Kitakyushu-shi are also shown in this tabl
2.The areas of cities with prefectural governments and designated cities under article 252-19 of the Loc

単位 円　In Yen

21 え び Shrimps & l 1g 数量 Quan.	22 か に Crabs 1g 数量 Quan.	23 あさり Short-necke 1g 数量 Quan.	24 しじみ Fresh water 1g 数量 Quan.	25 かき（貝 Oysters 1g 数量 Quan.	26 ほたて貝 Scallops 1g 数量 Quan.	27 塩さけ Salted salm 1g 数量 Quan.	28 たらこ Salted pollo 1g 数量 Quan.	29 しらす干し "Shirasu-boshi", dried young 1g 数量 Quan.	30 干しあじ Dried horse 1g 数量 Quan.	31 かつお節・削 Bonito fillets & fish flakes, d 1g 数量 Quan.	32 牛 肉 Beef 1g 数量 Quan.	33 豚 肉 Pork 1g 数量 Quan.	34 鶏 肉 Chicken 1g 数量 Quan.	35 合いびき肉 Mixed groun 1g 数量 Quan.	36 ハ ム Ham 1g 数量 Quan.	37 ソーセージ Sausages 1g 数量 Quan.	38 ベーコン Bacon 1g 数量 Quan.	39 牛 乳 Fresh milk 1l 数量 Quan.	40 粉ミルク Powdered m 1g 数量 Quan.	41 バター Butter 1g 数量 Quan.	42 チーズ Cheese 1g 数量 Quan.	43 卵 Eggs 1g 数量 Quan.	44 キャベツ Cabbage 1g 数量 Quan.	45 ほうれんそう Spinach 1g 数量 Quan.	46 はくさい Chinese cab 1g 数量 Quan.	47 ね ぎ Welsh onion 1g 数量 Quan.	48 レタス Lettuce 1g 数量 Quan.	49 ブロッコリー Broccoli 1g 数量 Quan.	50 もやし Bean sprout 1g 数量 Quan.	51 さつまいも Sweet potat 1g 数量 Quan.	52 じゃがいも White potato 1g 数量 Quan.
1,702	620	1,059	280	494	636	1,652	792	522	837	274	6,897	19,464	15,136	1,850	3,042	5,524	1,473	80.33	302	504	2,846	29,926	17,993	3,358	8,622	4,962	6,179	3,693	6,942	3,034	10,819
1,992	1,262	705	494	360	1,270	2,616	1,240	198	314	305	4,839	24,099	17,332	1,194	2,901	7,059	2,009	71.42	462	579	3,138	32,024	17,054	3,302	9,622	5,724	5,661	4,443	8,191	3,623	12,075
2,268	823	721	574	235	2,693	4,564	1,540	310	451	189	5,536	23,755	14,210	886	3,198	7,346	1,929	69.03	315	508	2,552	34,984	17,658	4,785	8,986	5,418	6,644	5,115	10,546	3,941	9,012
1,217	490	869	348	235	1,019	3,960	971	302	646	240	4,107	19,559	13,204	917	2,501	4,707	1,509	87.86	329	498	2,693	29,603	16,769	4,237	8,847	6,419	4,482	3,928	8,507	3,327	9,372
1,228	562	1,110	337	792	751	1,726	1,020	443	562	159	4,260	20,421	12,722	889	3,125	5,379	1,792	74.69	242	502	3,263	31,347	16,597	4,104	8,813	5,650	4,970	4,124	8,497	3,832	11,212
1,671	441	533	592	478	1,163	3,157	1,587	439	509	172	4,548	21,656	15,146	917	3,113	4,998	2,027	78.87	334	431	2,481	28,183	21,147	5,338	7,840	6,878	5,751	4,228	9,023	4,402	9,347
1,098	345	752	192	309	1,126	2,319	1,424	301	426	264	9,450	19,626	12,856	1,251	2,648	5,368	1,844	91.82	80	476	2,984	31,623	18,250	4,670	5,289	5,467	4,816	4,115	8,811	2,966	9,343
1,123	244	922	331	439	552	2,234	1,094	523	522	135	4,070	19,386	11,379	635	3,026	5,513	1,400	69.02	171	386	2,658	30,139	17,941	3,505	8,206	5,285	4,536	3,863	8,904	2,918	10,850
1,148	344	809	775	297	350	2,436	855	729	974	188	4,005	18,509	11,066	1,125	3,787	4,893	1,356	80.28	459	441	3,278	25,954	17,208	3,810	7,152	6,026	6,440	4,459	5,774	3,376	8,405
957	444	721	368	306	501	1,410	875	487	737	149	3,693	18,867	13,300	1,194	3,081	5,336	1,764	75.42	599	370	2,851	29,486	18,492	3,180	7,640	4,951	6,144	3,485	7,477	3,019	9,843
871	430	1,033	205	307	452	2,055	848	411	933	247	3,478	16,625	10,701	1,074	2,491	5,226	1,197	68.26	270	412	2,746	23,301	18,345	2,778	5,113	5,018	6,194	3,371	6,316	2,735	10,663
1,117	294	1,282	306	387	511	1,668	937	560	1,231	186	6,288	21,116	15,931	1,516	3,381	5,378	1,684	86.61	233	592	3,239	27,273	20,206	3,574	8,559	7,238	7,244	4,304	6,804	2,461	12,479
1,476	600	1,713	322	451	684	1,908	1,109	707	1,467	449	6,030	18,985	14,745	1,451	3,166	5,067	1,615	97.54	546	635	3,402	29,636	21,239	3,864	9,319	6,500	8,223	4,410	7,139	4,805	12,428
1,308	495	1,273	388	466	508	1,666	735	693	1,114	211	6,358	19,191	14,352	1,638	3,104	5,089	1,489	81.94	386	675	3,637	25,362	17,910	3,797	8,542	6,308	7,208	4,903	6,882	2,878	11,547
1,766	585	1,387	344	409	688	1,374	847	764	1,145	238	6,945	23,008	15,647	1,751	3,778	5,150	1,767	83.81	457	728	3,744	27,380	18,862	3,604	8,538	6,506	8,629	4,695	7,909	3,223	12,091
2,272	1,687	1,280	434	452	1,081	4,696	1,422	391	677	219	3,337	23,249	13,469	961	2,998	5,946	1,647	76.98	659	350	2,800	32,321	18,340	3,897	7,313	6,719	6,189	4,709	8,702	4,004	13,746
1,802	758	836	361	253	396	2,850	1,111	434	694	126	6,307	19,836	11,214	2,558	4,333	6,193	1,692	88.28	148	452	2,818	31,110	19,117	3,884	9,315	4,847	5,298	3,635	8,738	2,954	12,198
1,614	1,492	1,048	205	489	301	1,285	614	414	694	101	6,695	17,732	13,462	2,396	2,957	5,862	1,311	83.35	146	390	2,988	31,395	15,994	2,901	9,109	4,493	5,161	4,439	8,527	3,432	10,462
1,039	1,864	818	356	292	295	1,195	770	338	506	125	7,195	15,160	11,495	2,249	3,212	6,184	1,143	75.00	463	355	2,191	34,458	15,566	3,254	6,571	3,667	4,526	2,667	7,144	1,733	11,769
1,154	338	1,635	662	398	633	1,870	818	778	1,742	290	5,929	22,597	14,558	1,039	3,333	5,509	1,552	73.89	248	455	2,772	26,277	20,607	3,874	9,808	4,760	7,050	3,714	8,028	3,041	11,003
1,150	442	1,343	233	264	755	2,214	1,149	627	701	182	3,365	17,706	11,949	1,073	2,673	5,717	1,594	79.45	419	465	3,120	28,303	22,672	3,314	7,918	4,034	6,135	4,270	6,172	2,962	7,562
1,406	678	959	177	484	471	1,138	478	472	852	315	5,660	18,205	13,159	1,713	3,315	5,757	1,351	78.81	278	424	2,423	30,891	16,552	2,790	7,811	4,196	4,786	2,876	7,181	2,825	9,717
1,468	1,085	1,177	425	490	705	2,003	587	2,436	1,766	532	5,133	22,383	13,295	1,919	3,008	5,117	1,461	94.80	289	567	3,147	27,743	19,302	3,384	8,608	6,057	7,059	4,088	6,150	5,057	13,774
2,013	424	1,354	148	522	886	1,074	682	473	800	316	6,688	18,466	14,444	2,132	3,355	5,037	1,200	80.88	85	632	2,896	29,824	16,826	3,178	8,518	5,581	5,555	4,107	6,601	3,374	11,206
1,970	329	1,054	81	598	529	1,625	406	319	799	268	8,207	15,928	15,520	2,383	2,699	5,877	1,101	87.27	0	514	2,836	35,235	15,951	2,583	7,661	3,818	5,430	3,616	5,720	3,153	9,727
1,725	344	1,032	245	330	257	1,518	510	510	815	211	9,037	17,463	15,790	2,470	3,010	5,009	1,288	88.60	237	555	3,034	28,946	15,129	3,261	9,290	3,981	5,421	4,131	5,757	2,539	11,210
2,249	882	1,106	416	914	447	1,210	863	877	609	276	11,689	17,571	15,974	2,620	3,515	4,660	1,364	92.63	369	705	3,269	32,299	19,367	4,337	11,846	5,351	6,389	4,082	5,417	3,643	13,815
2,074	451	801	129	578	373	1,183	782	681	435	280	11,290	18,329	17,127	2,800	2,831	5,419	1,220	84.38	167	605	2,629	32,802	17,419	3,434	11,062	4,870	5,947	3,315	5,544	3,097	12,450
1,689	589	668	81	1,002	409	1,021	542	308	362	350	9,142	15,835	14,925	1,940	3,313	4,505	1,071	85.16	101	555	2,942	27,006	15,527	3,227	10,579	4,455	4,470	3,684	5,196	2,981	9,917
2,136	778	708	153	747	467	1,599	468	664	461	387	11,556	19,956	16,968	2,877	2,713	5,096	1,171	97.66	225	638	3,135	34,752	17,427	4,121	11,294	4,507	6,023	4,164	6,974	3,167	11,237
3,074	513	944	321	639	572	1,163	516	2,005	633	309	10,429	19,764	17,904	3,002	3,400	5,528	1,323	78.61	115	452	1,991	33,930	18,042	3,188	12,919	4,148	4,802	3,425	5,981	4,840	11,668
2,060	3,775	1,012	546	1,520	537	1,860	533	360	484	174	7,901	19,077	16,121	3,410	3,129	5,715	1,447	92.05	317	496	2,264	39,686	15,556	3,769	7,555	4,024	5,598	3,571	7,980	2,307	9,985
1,611	1,059	557	1,059	445	305	893	386	293	787	261	6,818	19,049	17,204	2,371	2,847	5,959	1,454	93.53	44	448	2,344	35,044	21,420	4,151	8,246	4,597	5,436	3,536	6,276	2,033	11,458
2,086	625	1,002	216	1,020	520	1,191	590	238	252	349	7,674	18,380	17,368	2,888	2,579	5,723	1,445	73.36	290	488	2,447	30,915	17,520	2,670	6,942	3,418	5,604	3,105	6,377	2,372	9,152
1,440	319	798	185	1,880	618	1,103	605	245	300	174	10,449	18,232	16,783	2,086	3,262	4,847	1,501	90.04	279	475	2,857	30,762	20,658	3,408	10,090	5,126	6,217	3,781	6,171	2,627	12,578
2,252	637	801	227	372	445	960	613	211	768	270	9,486	16,733	17,686	2,723	3,039	5,793	1,749	76.59	716	381	2,392	33,036	16,458	2,852	8,240	3,626	4,888	2,885	6,250	2,725	10,643
2,008	357	1,096	235	438	282	1,161	312	381	380	337	8,844	15,007	13,875	2,307	1,893	5,262	1,552	76.78	472	477	2,268	25,412	18,986	3,446	10,546	4,013	5,588	3,751	6,098	5,007	10,629
1,804	549	969	178	2,916	281	965	332	279	260	245	7,783	15,703	14,916	2,215	2,541	4,149	1,209	89.23	171	470	2,514	31,877	19,206	3,244	9,437	4,176	4,781	3,503	6,617	2,996	10,769
1,869	526	903	179	464	511	635	386	287	526	196	9,164	16,709	15,737	2,953	2,975	4,559	1,064	82.78	319	387	2,205	35,418	15,732	3,049	8,129	3,691	4,190	3,107	5,698	2,764	9,651
1,787	590	875	263	301	376	1,494	382	1,040	1,151	475	7,470	15,684	16,958	3,088	1,890	4,628	1,180	58.44	450	378	1,962	33,502	15,548	3,269	12,265	3,107	5,592	2,770	5,677	2,561	9,357
1,695	517	837	119	219	546	811	1,567	164	542	191	9,883	20,933	21,446	2,282	2,509	5,613	1,809	77.81	236	544	3,264	28,735	17,728	2,619	8,127	4,569	6,066	3,493	6,166	2,518	12,016
2,134	299	1,553	477	461	324	1,073	921	193	820	187	9,221	18,434	17,382	2,726	2,868	5,053	1,639	73.80	616	376	2,422	29,759	18,515	3,745	10,660	3,883	5,620	3,497	7,551	3,338	12,070
1,922	936	1,038	285	1,040	471	906	694	146	886	234	7,346	17,682	17,380	2,870	2,953	5,349	2,297	68.33	358	353	2,359	31,145	17,859	2,476	7,057	3,091	5,875	3,782	6,392	2,550	11,356
1,950	603	973	70	408	392	671	815	254	556	299	8,988	17,484	18,880	2,533	2,929	5,456	1,492	67.24	393	357	2,567	31,369	16,612	2,708	7,593	3,264	5,398	3,360	6,179	3,381	9,331
1,882	424	967	107	395	525	490	922	235	824	177	10,381	16,792	18,878	2,668	3,181	5,248	1,339	79.22	365	401	2,466	32,648	17,632	2,883	10,814	4,059	5,586	3,017	6,800	3,010	10,670
1,315	276	843	183	230	330	424	515	149	1,266	289	7,073	16,702	17,257	2,156	2,192	4,913	1,202	76.06	490	439	2,220	26,989	18,311	2,722	10,413	3,596	5,662	3,280	5,186	3,068	9,801
2,049	271	1,378	74	188	370	444	722	166	705	304	5,752	19,699	18,543	2,352	2,449	5,086	1,615	63.84	272	316	2,229	31,329	19,638	2,439	8,586	4,514	5,233	3,103	6,988	3,705	11,164
929	77	503	127	88	253	272	202	101	67	1,944	7,564	19,624	12,996	2,338	2,075	5,370	2,213	53.92	813	193	3,081	31,813	12,816	3,711	5,618	2,481	4,986	3,055	5,617	1,808	8,893
1,461	360	1,265	323	354	508	1,387	931	708	1,242	218	6,585	22,447	15,929	2,008	3,586	5,594	1,692	84.19	286	628	3,446	26,836	20,877	4,008	8,147	6,753	7,626	4,716	7,684	3,075	12,238
1,379	351	1,009	408	348	606	1,729	819	628	978	200	5,757	22,565	16,081	1,803	3,714	6,400	1,511	79.61	0	591	3,868	27,979	22,116	3,841	8,764	6,006	7,054	4,456	8,425	3,268	11,749
1,519	810	1,155	111	448	436	1,280	381	1,047	922	433	4,588	20,174	12,300	1,560	3,577	5,615	1,504	95.65	493	540	3,217	32,226	16,244	2,970	7,775	5,058	5,943	3,129	6,472	2,885	9,840
2,123	641	617	141	475	641	1,734	647	640	410	317	9,578	17,626	16,537	2,611	2,731	5,601	1,356	95.60	254	539	2,714	32,039	20,488	4,063	14,493	5,372	6,182	4,219	6,376	3,846	14,588
2,372	1,237	1,266	144	888	333	1,064	1,671	212	884	222	10,792	15,986	18,061	1,906	3,207	4,581	1,608	66.13	199	354	2,346	27,869	18,814	3,470	9,864	4,444	5,149	2,836	6,692	2,572	12,265

e.

al Autonomy Act are as of April 1, 2012.

53 さといも Taros 1g 数量 Quan.	54 だいこん Radishes 1g 数量 Quan.	55 にんじん Carrots 1g 数量 Quan.	56 ごぼう Burdocks 1g 数量 Quan.	57 たまねぎ Onions 1g 数量 Quan.	58 れんこん Lotus roots 1g 数量 Quan.	59 たけのこ Bamboo sho 1g 数量 Quan.	60 さやまめ String beans 1g 数量 Quan.	61 かぼちゃ Pumpkins 1g 数量 Quan.	62 きゅうり Cucumbers 1g 数量 Quan.	63 なす Eggplants 1g 数量 Quan.	64 トマト Tomatoes 1g 数量 Quan.	65 ピーマン Green peppe 1g 数量 Quan.	66 生しいたけ "Shiitake", Japanese m fresh 1g 数量 Quan.	67 干ししいたけ "Shiitake", Japanese mushrooms, dried 1g 数量 Quan.	68 わかめ "Wakame", 1g 数量 Quan.
2,086	13,534	8,630	2,037	16,095	1,560	913	2,224	4,461	8,358	4,505	12,185	2,667	1,611	66	874
714	15,148	8,355	2,300	21,180	859	712	1,495	4,604	7,391	4,360	10,318	3,121	2,032	68	571
633	16,005	10,368	2,681	22,724	891	742	2,489	4,294	7,990	6,475	15,084	2,836	1,902	43	1,626
2,180	19,666	10,299	2,914	17,280	948	494	1,844	4,396	7,416	4,313	10,704	2,712	1,871	116	1,680
2,328	19,821	9,495	2,463	17,213	1,516	1,076	2,496	5,026	9,368	5,285	13,385	3,014	1,564	61	1,581
1,768	15,421	8,792	2,184	17,580	921	775	3,754	4,272	8,808	6,230	14,187	2,344	2,516	61	1,474
3,345	13,269	8,480	2,432	15,846	871	2,498	2,184	4,783	11,657	4,953	10,971	2,247	1,453	52	994
2,456	15,896	10,656	2,424	15,138	1,001	934	2,147	3,895	10,699	4,566	9,818	2,273	1,509	26	1,229
2,508	15,685	8,297	1,929	15,576	2,677	341	1,740	4,185	8,573	3,549	11,268	2,565	1,398	28	884
2,303	17,194	9,428	2,198	16,104	1,165	717	1,560	4,680	8,597	4,874	10,445	2,460	1,589	23	1,077
1,945	11,414	8,051	1,526	11,966	1,114	499	1,956	4,395	10,364	3,740	14,041	2,507	1,394	23	631
2,265	15,301	9,363	1,864	18,207	1,515	796	2,430	5,643	9,619	5,006	15,726	3,349	1,580	39	957
2,610	17,347	9,184	1,995	17,353	1,905	1,099	3,624	5,483	9,092	5,362	16,627	3,053	1,581	82	987
2,175	15,337	8,720	1,753	15,555	1,648	1,153	3,803	4,534	9,424	5,166	15,041	2,968	1,420	68	806
2,125	15,838	9,447	1,927	18,006	1,851	1,929	3,508	6,016	11,171	6,460	15,847	3,431	1,545	74	722
3,844	17,860	11,179	1,937	17,917	2,771	1,602	7,182	5,280	10,171	6,660	17,306	2,478	1,530	73	833
2,689	14,149	8,136	1,868	15,173	1,306	1,402	1,816	5,016	8,064	5,302	12,840	2,520	2,201	48	843
1,837	15,385	9,061	1,481	15,123	2,066	1,280	2,006	4,797	8,345	3,538	12,823	2,543	1,772	52	852
2,704	12,222	7,338	1,291	14,527	751	838	1,692	3,443	6,413	3,664	9,975	1,952	1,462	44	1,064
1,757	18,549	9,259	2,397	14,644	1,793	814	1,895	5,146	11,267	3,533	12,772	2,758	1,518	71	1,200
1,888	13,198	8,446	1,985	14,951	1,864	1,438	1,911	3,944	10,299	3,789	13,864	2,517	1,300	41	1,145
2,809	12,052	7,660	1,810	13,856	1,639	511	2,049	4,072	5,762	4,080	8,986	2,175	1,612	87	798
3,106	15,678	9,747	1,918	16,310	2,430	911	3,098	6,373	8,988	4,756	13,074	3,258	1,904	71	1,100
3,447	13,538	8,293	1,921	16,089	1,817	595	2,426	4,610	7,870	5,124	12,774	2,572	1,281	80	470
2,081	12,220	8,406	1,575	14,882	1,461	839	1,709	3,626	7,796	3,743	12,298	2,591	1,581	58	488
1,950	11,601	8,389	1,292	16,559	1,000	696	1,937	4,808	6,480	5,149	11,250	2,424	1,601	56	490
2,508	16,373	8,048	1,775	19,581	1,312	1,476	2,454	5,197	7,938	6,747	13,829	3,102	1,986	65	587
1,875	13,124	8,037	1,798	19,618	1,818	764	2,165	4,533	6,909	4,946	10,293	2,792	1,719	56	486
1,659	11,512	7,628	1,811	14,732	1,389	763	1,867	4,524	6,129	4,693	11,329	2,750	1,870	34	939
2,120	13,776	8,968	2,122	18,099	1,519	1,004	1,926	4,919	7,140	5,333	11,671	2,802	1,750	69	620
1,747	11,943	7,402	1,848	14,610	1,034	486	2,144	4,640	6,905	4,673	9,349	2,094	1,756	68	819
1,442	11,820	7,530	1,589	14,041	629	473	1,432	4,482	5,425	4,185	8,711	2,588	2,108	35	967
1,896	10,604	9,292	2,272	17,199	692	423	1,436	4,579	6,747	4,135	8,969	2,725	1,687	69	1,379
1,028	9,726	7,221	2,095	14,402	1,671	799	1,353	3,949	6,135	3,010	8,916	2,346	1,327	41	483
2,361	12,819	9,263	2,545	14,996	2,566	578	2,250	5,965	8,114	4,865	10,766	2,505	1,737	53	530
1,757	11,357	8,654	2,745	13,621	1,612	930	1,766	5,209	7,311	4,426	9,103	2,516	1,588	37	622
1,605	11,769	7,303	1,456	15,536	1,426	1,220	2,469	4,721	7,440	5,121	10,278	2,199	2,267	78	1,056
1,445	10,603	8,878	2,100	15,917	1,794	792	1,617	5,296	6,863	4,339	10,149	2,351	1,818	51	745
2,478	9,436	8,191	2,211	14,641	2,103	992	2,195	4,839	7,737	4,537	9,419	2,030	1,756	44	496
1,190	11,050	6,693	1,716	13,352	750	1,354	1,628	4,430	9,265	4,542	11,570	2,395	1,437	26	911
2,361	11,726	9,565	2,253	18,677	1,458	704	2,105	4,111	8,161	4,036	11,242	2,997	1,503	81	998
2,826	12,792	9,283	3,060	17,833	2,729	1,025	2,139	4,710	9,506	5,476	12,068	2,470	1,637	98	1,329
2,153	11,771	8,609	1,954	16,263	1,662	864	1,787	4,851	8,667	4,706	10,820	2,411	1,576	111	1,167
3,505	10,130	8,011	2,220	15,946	1,680	746	1,821	4,264	9,078	4,131	12,376	2,875	1,485	120	840
3,443	11,665	8,020	2,948	16,233	1,228	885	1,720	4,560	9,208	4,674	11,479	2,677	2,033	350	1,171
2,425	11,250	9,498	1,834	14,047	717	707	1,342	3,644	8,349	3,510	12,366	2,411	1,732	183	424
2,120	13,360	9,449	1,920	16,249	721	656	1,125	4,619	8,796	3,843	13,853	2,482	1,843	92	525
290	12,806	11,474	1,519	14,975	198	410	1,612	2,999	5,465	2,095	10,249	3,476	708	108	319
2,812	15,286	10,027	1,930	16,077	1,771	1,012	3,106	5,581	11,901	5,361	18,061	3,064	1,689	69	750
2,156	16,163	10,100	2,145	19,560	1,455	998	2,677	4,850	8,754	4,682	11,408	2,793	1,688	51	1,228
2,274	12,342	8,655	1,833	13,461	1,614	723	1,843	4,275	6,706	4,278	12,189	2,454	1,600	40	774
3,057	14,046	10,231	1,670	21,314	1,611	1,602	2,270	4,874	7,354	7,003	13,197	3,264	1,913	80	693
3,243	14,177	8,787	3,016	18,531	2,307	1,114	1,879	6,456	10,299	6,038	12,810	3,298	1,554	147	1,149

69 こんぶ Dried tangle 1g 数量 Quan.	70 豆腐 Bean curd 1丁 each 数量 Quan.	71 梅干し "Umeboshi", pickled plum 1g 数量 Quan.	72 だいこん漬 Pickled radis 1g 数量 Quan.	73 はくさい漬 Pickled chinese cab 1g 数量 Quan.	74 こんぶつくだ Tangle prepared in soy sauce 1g 数量 Quan.	75 りんご Apples 1g 数量 Quan.	76 みかん Mandarin or 1g 数量 Quan.	77 グレープフル Grapefruits 1g 数量 Quan.	78 オレンジ Oranges 1g 数量 Quan.	79 梨 Pears 1g 数量 Quan.	80 ぶどう Grapes 1g 数量 Quan.	81 柿 Persimmons 1g 数量 Quan.	82 桃 Peaches 1g 数量 Quan.	83 すいか Watermelon 1g 数量 Quan.	84 メロン Melons 1g 数量 Quan.
337	79.82	754	1,521	837	623	13,315	12,290	1,741	1,758	4,008	2,506	2,747	1,634	4,132	2,174
381	60.87	1,062	1,763	394	496	11,039	12,030	2,550	1,393	1,895	2,273	3,589	1,032	4,055	4,874
736	99.54	1,128	2,124	1,125	681	35,056	10,426	1,417	1,980	2,222	1,760	2,997	1,031	4,272	4,067
504	93.66	990	1,698	1,037	672	28,605	12,604	2,000	1,817	3,426	2,882	2,291	1,743	3,472	2,452
579	84.29	687	1,756	1,137	564	14,830	14,457	3,077	1,903	6,117	2,362	3,456	2,828	3,214	2,123
476	80.91	864	2,368	439	503	21,591	14,444	3,097	1,772	6,339	3,294	3,513	1,648	5,088	3,884
688	90.17	665	1,401	635	877	20,719	9,825	2,011	2,164	11,246	3,212	2,556	1,828	6,179	1,677
605	78.68	1,307	2,052	989	797	18,988	11,251	1,002	1,342	7,353	2,053	2,229	7,564	2,840	1,144
212	69.36	706	1,499	765	521	12,103	14,011	2,228	2,694	6,859	2,831	2,275	1,953	4,683	6,094
294	86.09	1,283	1,683	819	929	8,323	13,564	2,888	1,696	5,073	2,010	2,065	1,458	3,215	2,165
254	86.40	859	1,334	703	617	16,152	11,718	3,416	1,511	4,980	2,323	1,021	1,251	4,324	1,322
237	80.70	671	952	764	693	10,034	12,173	1,905	1,508	4,130	1,365	2,002	1,256	4,852	1,302
453	80.17	1,434	1,902	1,150	789	11,394	14,237	3,684	2,819	4,485	3,492	3,603	2,727	7,906	4,102
277	76.46	984	1,518	1,077	567	10,898	11,347	2,497	1,641	3,680	2,065	2,513	2,475	5,783	2,436
233	82.28	797	1,470	786	592	11,779	9,785	3,058	1,663	3,061	2,390	2,616	1,700	4,761	2,175
394	96.51	521	1,767	938	923	18,119	15,050	3,406	1,817	11,844	1,892	2,902	1,972	7,883	3,399
561	89.56	470	2,071	1,602	868	13,539	13,096	1,975	3,507	6,225	1,927	1,934	987	5,257	1,994
371	85.10	566	1,776	928	838	10,710	12,448	1,226	1,443	5,190	2,514	1,714	1,235	5,078	1,585
456	65.75	554	1,487	722	1,006	9,178	12,206	517	2,250	2,335	1,547	1,199	655	4,466	1,749
271	84.97	664	1,195	870	607	10,890	13,286	1,377	2,439	2,850	5,572	2,385	4,647	3,182	1,303
416	65.49	396	1,800	719	706	30,103	13,315	1,478	1,639	3,998	5,511	1,225	7,279	4,265	1,922
244	84.34	662	1,692	584	748	9,753	8,593	943	1,924	2,620	2,010	6,145	686	3,020	1,050
302	94.42	703	1,829	1,165	548	12,021	23,798	897	1,544	4,613	2,441	3,062	1,256	4,191	1,811
187	85.58	645	1,536	495	531	11,179	9,670	1,311	3,076	2,790	2,691	2,846	1,214	7,540	2,420
259	73.27	581	1,863	708	568	9,779	13,385	1,361	2,201	5,986	1,907	2,616	1,089	4,087	1,420
181	71.77	858	1,236	841	1,029	10,069	8,624	1,309	1,641	3,804	1,959	2,091	1,232	2,259	1,617
410	76.54	823	1,535	804	716	9,887	13,578	1,287	1,221	2,218	2,816	2,616	1,093	4,515	1,268
298	74.63	751	1,078	633	718	9,053	8,426	1,329	1,835	2,224	1,863	2,823	1,420	4,458	1,659
179	71.41	714	1,485	655	679	12,811	11,177	1,659	1,573	3,446	2,446	3,620	1,063	2,909	859
358	75.54	631	2,125	720	803	13,589	12,338	1,824	1,511	3,330	2,350	5,922	1,972	3,604	1,783
362	72.85	1,867	1,087	601	649	11,206	11,718	660	2,021	2,775	2,380	3,394	3,687	4,187	1,564
295	84.14	763	1,652	2,261	829	15,245	13,779	2,038	2,612	13,137	2,106	6,482	887	7,261	3,123
450	83.98	814	1,909	677	607	10,551	14,800	618	2,227	7,722	2,892	2,436	749	4,024	1,329
280	77.15	834	1,263	647	364	8,857	11,220	1,060	1,856	2,998	4,381	2,909	4,836	4,591	1,188
300	83.88	806	1,389	1,473	463	13,032	14,270	798	2,623	3,214	2,777	2,050	1,022	4,642	1,284
309	83.18	696	1,230	1,013	544	13,138	12,705	835	1,764	4,738	2,249	880	1,049	3,270	2,943
177	80.12	744	1,719	1,100	542	12,654	15,813	686	1,268	3,058	2,295	3,664	1,408	4,796	659
270	74.02	846	1,229	800	329	14,581	14,788	172	1,960	3,248	2,745	3,669	3,180	4,295	1,869
282	76.71	525	1,387	765	364	11,505	14,885	212	449	3,627	2,588	3,793	1,379	4,340	1,529
421	72.20	468	2,220	2,204	472	11,425	13,108	570	1,063	6,055	2,107	4,129	1,661	3,387	1,281
268	71.29	549	1,001	467	309	8,265	10,950	593	980	2,831	1,217	2,218	492	2,810	825
266	87.51	821	2,074	1,147	471	10,672	14,945	674	1,229	7,458	2,231	2,720	737	4,689	1,164
338	75.40	912	1,239	626	515	8,397	14,884	175	1,329	3,013	2,674	3,201	1,097	4,326	2,706
189	70.55	566	1,419	648	485	9,286	13,969	461	796	5,138	1,927	2,155	654	6,719	3,900
267	81.86	421	1,361	602	559	10,216	17,868	249	1,432	5,050	2,588	1,879	879	4,960	1,800
190	69.24	663	1,596	638	503	11,991	12,182	892	548	4,952	2,476	3,813	590	3,151	3,769
200	77.20	513	1,203	382	711	8,681	13,029	784	1,343	2,752	2,190	2,846	867	5,483	1,460
455	68.40	922	662	96	200	7,776	8,029	1,277	2,337	1,226	1,171	1,396	208	2,310	758
677	72.88	734	1,047	1,241	491	12,995	9,913	2,487	1,613	3,154	1,854	3,686	2,321	5,599	1,687
319	82.53	709	1,573	673	512	11,877	11,089	3,701	2,312	3,716	2,639	2,419	1,209	5,198	2,126
244	95.14	570	1,908	761	428	10,903	19,853	471	2,125	4,940	2,750	3,332	1,173	4,052	3,559
413	86.31	892	1,520	983	1,064	12,607	14,271	1,701	2,008	3,058	2,542	3,592	2,678	5,880	1,254
343	83.50	738	1,574	1,094	403	11,225	12,011	1,254	1,711	4,224	2,850	2,899	1,447	7,357	2,797

Notes: 1.Kawasaki-shi, Sagamihara-shi, Hamamats 1. この表には川崎市，相模原市，浜松市，堺市及び北九州市も掲載されている。
2.The areas of cities with prefectural gove 2. 都道府県庁所在市及び政令指定都市の地域は，2012年4月1日現在の区域による。

85 いちご Strawberries 1g 数量 Quan.	86 バナナ Bananas 1g 数量 Quan.	87 キウイフルーツ Kiwi fruits 1g 数量 Quan.	88 食用油 Edible oil 1g 数量 Quan.	89 マーガリン Margarine 1g 数量 Quan.	90 食塩 Salt 1g 数量 Quan.	91 しょう油 Soy sauce 1ml 数量 Quan.	92 みそ "Miso", soy 1g 数量 Quan.	93 砂糖 Sugar 1g 数量 Quan.	94 酢 Vinegar 1ml 数量 Quan.	95 ソース Worcester s 1ml 数量 Quan.	96 ケチャッ Tomato ket 1g 数量 Quan.	97 マヨネーズ・マヨネーズ風調味料 Mayonnaise & mayonnaise flavor seasoning 1g 数量 Quan.	98 ドレッシング Dressing 1ml 数量 Quan.	99 ジャム Jam 数量 Quan.	100 カレールウ Instant curr 1g 数量 Quan.	101 緑茶 Green tea 1g 数量 Quan.	102 紅茶 Black tea 1g 数量 Quan.	103 コーヒー Coffee 1g 数量 Quan.	104 清酒 "Sake" 1ml 数量 Quan.	105 焼酎 "Shochu", distilled spir 1ml 数量 Quan.	106 ビール Beer 1l 数量 Quan.	107 ウイスキー Whisky 1ml 数量 Quan.	108 ワイン Wine 1ml 数量 Quan.	109 発泡酒・ビール風アルコール飲料 Low-malt beer & beer-flavored alcoholic beverages 1l 数量 Quan.
2,794	18,235	1,588	8,324	1,231	2,196	5,934	5,868	5,959	2,439	1,522	1,619	2,584	2,294	1,283	1,566	874	227	2,318	7,443	10,115	22.55	1,033	3,315	30.98
2,275	15,435	1,981	10,858	978	2,610	5,465	6,411	6,264	3,005	1,210	1,838	2,931	1,982	959	1,543	892	217	2,943	7,373	16,017	29.69	2,991	5,089	35.24
1,915	22,231	1,926	10,177	1,294	3,280	5,928	7,608	5,951	3,114	1,693	1,763	2,953	2,260	1,450	1,900	542	202	2,579	6,441	15,904	19.36	3,165	2,854	38.92
3,440	19,691	1,365	6,862	1,163	4,073	5,695	7,408	3,665	2,028	954	1,467	2,117	2,116	1,264	1,674	594	247	2,695	8,014	14,667	18.03	1,948	3,179	39.15
3,608	18,496	2,115	7,237	982	3,405	4,456	7,157	4,815	2,077	1,000	1,373	2,087	2,236	1,276	1,385	1,135	279	2,247	9,812	9,066	27.67	1,309	3,409	41.57
2,550	16,992	1,673	9,989	919	3,751	6,329	9,487	7,336	2,426	1,297	1,096	2,606	2,055	838	1,705	800	208	2,199	12,163	14,629	25.56	1,295	2,932	33.85
2,363	16,976	1,232	8,561	791	5,117	9,705	6,686	6,838	2,725	1,588	1,373	2,675	2,296	1,256	1,668	554	241	2,703	7,347	10,824	20.64	1,245	3,426	44.79
2,637	15,519	1,246	8,572	812	2,988	5,437	6,067	4,519	2,364	1,326	1,273	2,837	2,210	1,022	1,493	538	181	1,876	11,419	10,317	15.57	1,048	2,565	36.80
2,938	21,660	1,535	8,697	740	1,702	5,788	5,589	5,305	1,943	920	1,604	2,252	2,469	1,632	1,526	839	184	2,226	6,337	6,142	19.73	725	2,369	27.41
4,590	16,757	1,029	7,954	1,074	919	6,041	4,423	4,820	1,623	1,309	1,552	2,381	2,468	1,250	1,524	1,062	120	2,483	7,866	11,922	16.10	1,214	3,587	37.18
3,021	19,052	1,708	7,207	892	1,677	5,494	3,974	4,045	2,355	1,260	1,570	2,222	2,364	1,353	1,593	852	159	2,193	9,979	6,529	18.53	1,153	4,862	30.53
2,754	15,238	1,706	8,243	1,341	1,526	6,855	5,998	4,166	1,936	1,213	1,568	2,302	2,771	1,179	1,406	765	175	2,760	7,569	8,285	26.52	1,690	2,221	36.18
2,918	18,906	1,737	7,960	1,307	1,929	6,948	5,599	6,571	2,296	1,432	1,572	2,411	2,350	1,521	1,364	1,048	285	2,498	7,372	8,689	21.56	1,123	3,577	26.09
3,307	16,439	1,724	7,039	1,083	1,344	4,835	4,971	3,959	2,732	1,230	1,510	2,067	2,409	1,521	1,421	843	290	2,439	6,784	7,813	22.35	1,299	8,486	29.76
3,851	16,663	1,389	7,880	1,515	1,427	5,402	5,109	4,454	2,433	1,234	2,009	2,404	2,515	1,778	1,663	1,227	487	2,986	6,567	10,732	19.57	1,932	6,064	28.64
3,269	19,892	1,649	9,329	1,090	3,810	6,230	7,550	6,350	2,023	1,161	1,375	2,812	2,124	1,234	1,752	996	327	2,407	13,707	11,343	24.48	1,573	2,463	41.81
2,848	20,441	1,455	5,679	1,449	2,134	5,666	7,811	3,891	2,437	1,255	1,434	2,235	2,490	1,340	1,606	1,064	121	2,306	7,271	9,989	20.05	1,854	2,207	29.47
2,386	19,000	1,999	6,795	1,548	2,290	5,115	6,176	3,654	1,856	1,082	1,669	2,212	2,074	964	1,897	985	167	2,043	9,843	6,847	24.30	648	2,681	25.59
1,959	15,730	1,244	6,783	1,261	2,600	5,019	6,741	4,840	1,724	1,374	1,507	2,749	2,052	876	1,598	1,313	147	1,830	7,773	6,637	19.05	407	1,874	24.64
2,331	15,239	1,785	9,313	992	2,374	5,896	6,369	4,461	2,524	1,363	1,372	2,255	2,689	843	1,496	1,019	201	2,058	7,156	10,287	20.15	1,363	4,073	16.23
2,648	17,653	1,505	8,604	1,113	2,646	5,358	8,638	6,107	2,628	1,095	1,212	2,693	2,750	1,409	1,276	527	308	2,233	11,633	9,603	26.78	717	4,512	25.38
1,698	17,490	1,285	8,007	1,263	1,698	5,073	5,351	6,080	2,570	1,608	1,747	2,654	1,990	1,090	1,331	752	174	2,136	5,700	5,967	19.26	780	2,579	34.50
3,303	19,456	1,197	7,494	1,268	1,705	4,930	7,214	6,333	2,745	1,570	1,502	2,224	2,144	1,335	1,496	2,352	231	1,618	6,072	9,282	24.41	833	2,968	24.90
2,946	20,478	1,614	7,198	1,339	1,224	4,111	6,172	6,572	1,482	1,519	1,531	2,437	2,054	1,311	1,533	892	212	2,412	8,663	7,137	23.27	288	2,869	32.41
3,162	21,253	1,349	7,955	1,302	2,160	7,775	4,505	6,015	2,785	1,426	1,613	2,687	2,287	1,163	1,570	1,161	119	2,010	7,713	8,724	12.09	0	2,691	28.70
2,702	15,857	1,262	7,769	1,724	1,280	6,202	3,515	6,078	1,834	2,057	1,750	2,207	2,422	1,916	1,528	1,179	261	3,005	6,235	7,006	18.26	809	3,346	36.49
3,023	19,434	1,268	8,308	1,762	1,764	6,325	4,391	5,269	2,289	2,013	1,861	2,600	2,396	2,096	1,343	1,384	218	3,693	9,585	8,962	22.71	324	4,004	40.73
2,559	17,390	1,237	7,297	1,373	1,462	4,887	3,390	4,883	2,817	1,839	2,019	2,260	1,791	1,474	1,594	671	261	2,685	5,910	8,439	27.33	1,162	2,213	37.03
3,049	16,243	1,527	5,757	1,565	1,106	6,003	3,706	7,447	2,551	2,112	1,926	2,263	1,998	1,778	1,389	884	421	3,213	7,090	8,409	20.75	709	3,993	24.11
2,496	22,147	1,418	8,648	1,501	1,908	4,931	4,265	5,836	3,265	2,229	1,614	2,780	2,626	1,760	1,843	2,301	326	2,899	8,171	9,067	19.19	613	3,733	30.14
2,587	22,179	1,047	7,625	1,617	1,340	5,234	3,593	7,701	3,237	1,935	2,098	2,467	1,771	1,553	1,370	776	175	2,246	6,247	5,738	16.48	683	1,652	32.02
2,514	20,636	1,713	10,743	1,124	2,216	5,635	5,495	6,887	2,165	1,635	1,752	3,316	2,521	1,153	1,824	692	114	3,553	7,507	10,346	19.18	100	2,024	40.69
2,815	19,816	1,033	8,372	1,455	2,482	11,228	5,543	6,963	2,188	1,311	1,418	2,968	2,324	1,205	1,876	1,213	296	2,691	11,087	12,702	20.21	501	2,564	33.56
2,326	15,663	1,222	8,019	1,225	1,363	5,315	3,271	5,980	2,583	1,846	1,514	2,525	2,355	1,225	1,644	435	260	2,681	9,551	8,179	26.30	783	1,996	18.60
2,655	19,343	1,140	10,013	1,298	1,465	5,245	5,316	5,123	3,233	2,457	1,370	2,273	1,985	1,338	1,654	1,097	262	2,812	8,993	9,385	28.81	1,163	3,403	40.74
3,418	18,247	920	8,740	1,529	2,257	7,486	4,809	6,507	2,708	1,715	1,439	2,591	1,714	1,165	1,441	457	125	2,431	5,784	13,954	21.39	356	1,525	42.00
2,284	16,547	2,504	6,450	1,001	1,981	6,279	6,089	5,296	1,725	1,963	1,711	2,272	2,041	844	1,639	513	187	2,781	7,762	12,366	20.37	798	1,845	31.05
2,495	19,485	1,320	7,648	1,040	1,254	7,558	3,939	5,548	2,657	1,541	1,414	2,264	2,064	1,185	1,576	505	221	2,515	5,616	7,545	17.42	367	2,606	34.41
2,195	18,362	771	9,767	1,186	1,616	5,249	4,233	7,110	3,443	1,794	1,565	2,257	1,763	1,143	1,576	591	149	2,725	5,354	9,509	21.37	542	2,693	29.30
2,184	17,640	1,166	8,299	1,301	3,138	6,636	4,937	6,314	1,540	1,633	1,749	2,631	2,471	991	1,513	666	192	2,435	7,555	12,337	16.83	512	2,634	59.17
1,981	17,896	1,666	7,589	1,257	2,257	4,182	4,975	4,002	1,734	1,449	2,127	2,913	2,171	929	1,618	671	215	2,778	4,936	9,720	24.25	705	4,415	26.09
2,374	17,952	1,203	12,095	1,245	1,860	8,246	6,816	7,906	2,577	1,269	1,557	3,174	2,743	815	1,809	1,032	151	2,191	6,157	12,955	25.43	1,374	1,696	28.14
3,039	15,684	1,034	10,605	1,097	1,991	6,054	6,934	5,798	2,109	1,429	1,863	2,930	2,232	1,205	1,444	850	165	2,307	6,961	10,755	24.51	533	2,620	28.07
2,209	14,735	1,317	10,139	1,261	1,904	6,794	7,122	6,612	2,424	1,427	1,532	3,106	3,013	892	1,784	1,374	165	1,868	3,732	11,891	17.58	774	3,214	49.32
2,534	19,593	931	10,057	1,325	3,005	8,094	7,332	7,168	1,522	1,314	1,437	2,821	2,513	1,268	1,596	1,010	262	2,100	4,873	14,491	20.50	491	3,580	32.38
2,531	15,262	1,030	8,038	1,026	1,755	7,219	6,603	7,461	3,541	1,142	1,426	2,475	2,672	769	1,421	816	263	1,836	2,361	21,825	18.94	135	2,236	42.14
2,138	17,361	1,504	11,775	1,047	1,923	6,018	6,993	5,915	2,612	1,173	2,125	2,594	2,712	786	1,660	1,002	180	1,768	1,048	17,201	19.65	313	1,255	27.21
1,671	13,542	1,345	10,663	990	1,783	4,039	4,590	4,704	2,205	880	1,832	1,460	2,558	1,004	1,447	307	202	2,366	2,503	8,627	18.55	210	3,828	29.88
3,462	16,887	2,337	6,774	1,096	1,633	4,313	5,017	4,261	2,573	1,434	1,911	1,887	2,486	1,231	1,589	737	485	2,091	5,834	7,049	22.39	1,773	4,345	36.83
2,241	17,159	1,639	8,756	1,138	2,019	6,023	4,798	5,168	2,716	1,281	1,830	3,050	3,161	1,451	1,815	785	413	2,513	9,683	5,795	17.66	912	3,168	30.72
2,854	15,534	1,636	8,361	1,014	1,598	5,423	6,707	5,469	2,249	1,323	1,554	2,381	2,230	1,100	1,607	1,430	90	1,911	5,741	8,060	22.46	664	4,049	20.17
2,276	21,385	1,535	7,347	1,636	2,297	6,079	3,573	5,647	2,395	2,280	1,966	2,692	2,173	1,821	1,742	1,523	371	2,686	4,998	9,894	25.08	749	2,674	40.91
2,635	16,867	1,941	7,466	882	1,802	7,343	6,562	7,117	2,497	1,626	1,706	2,485	2,143	707	1,587	1,262	290	2,027	8,449	12,352	18.44	464	1,970	19.05

Nutrient calculations

		朝昼夕 間食	献立名	食品 番号	食品名	Weight 重量 (g)	食品群	廃棄率 (%)	Erergy エネルギー (kcal)	Erergy エネルギー (kJ)	Water 水分 (g)
**全国**	All Japan	総 合 計	0.0	0.0	0.0	720885.0	0.0	0.0	1088588.9	4553837.2	499562.3
札幌市	Sapporo-shi	総 合 計	0.0	0.0	0.0	755578.5	0.0	0.0	1170481.1	4897132.6	521080.6
青森市	Aomori-shi	総 合 計	0.0	0.0	0.0	818241.0	0.0	0.0	1236876.8	5174862.4	565739.4
盛岡市	Morioka-shi	総 合 計	0.0	0.0	0.0	769966.0	0.0	0.0	1094410.4	4578589.9	541576.9
仙台市	Sendai-shi	総 合 計	0.0	0.0	0.0	742183.5	0.0	0.0	1031855.7	4316736.4	529325.5
秋田市	Akita-shi	総 合 計	0.0	0.0	0.0	777974.5	0.0	0.0	1148948.1	4807078.6	541902.2
山形市	Yamagata-shi	総 合 計	0.0	0.0	0.0	788120.5	0.0	0.0	1167070.4	4882784.3	546489.7
福島市	Fukushima-shi	総 合 計	0.0	0.0	0.0	702777.0	0.0	0.0	1049523.3	4390996.4	486672.6
水戸市	Mito-shi	総 合 計	0.0	0.0	0.0	670797.0	0.0	0.0	952198.4	3983478.8	477398.1
宇都宮市	Utsunomiya-shi	総 合 計	0.0	0.0	0.0	691219.5	0.0	0.0	997029.3	4170944.3	489872.5
前橋市	Maebashi-shi	総 合 計	0.0	0.0	0.0	662809.0	0.0	0.0	977760.4	4090426.8	460732.4
さいたま市	Saitama-shi	総 合 計	0.0	0.0	0.0	745048.0	0.0	0.0	1086041.0	4543262.9	525629.9
千葉市	Chiba-shi	総 合 計	0.0	0.0	0.0	771966.5	0.0	0.0	1086323.2	4544543.3	549858.5
東京都区部	Ku-areas of Tokyo	総 合 計	0.0	0.0	0.0	703354.0	0.0	0.0	992828.2	4153264.9	502858.3
横浜市	Yokohama-shi	総 合 計	0.0	0.0	0.0	759305.0	0.0	0.0	1116811.9	4671964.2	534162.6
新潟市	Niigata-shi	総 合 計	0.0	0.0	0.0	829189.5	0.0	0.0	1197689.0	5010690.5	581616.9
富山市	Toyama-shi	総 合 計	0.0	0.0	0.0	741980.0	0.0	0.0	1075132.3	4497290.4	521682.3
金沢市	Kanazawa-shi	総 合 計	0.0	0.0	0.0	707205.0	0.0	0.0	1048856.1	4387444.5	492847.3
福井市	Fukui-shi	総 合 計	0.0	0.0	0.0	654763.5	0.0	0.0	1058655.0	4428687.1	435740.6
甲府市	Kofu-shi	総 合 計	0.0	0.0	0.0	707889.5	0.0	0.0	1089280.6	4556998.5	486733.5
長野市	Nagano-shi	総 合 計	0.0	0.0	0.0	719806.5	0.0	0.0	1032583.7	4319619.4	507516.3
岐阜市	Gifu-shi	総 合 計	0.0	0.0	0.0	683658.0	0.0	0.0	1088985.9	4555496.5	460621.1
静岡市	Shizuoka-shi	総 合 計	0.0	0.0	0.0	775410.0	0.0	0.0	1179474.6	4933937.5	531012.6
名古屋市	Nagoya-shi	総 合 計	0.0	0.0	0.0	726195.0	0.0	0.0	1098126.0	4593612.3	501705.1
津市	Tsu-shi	総 合 計	0.0	0.0	0.0	705462.5	0.0	0.0	1113649.6	4658727.8	478197.7
大津市	Otsu-shi	総 合 計	0.0	0.0	0.0	704236.5	0.0	0.0	1076057.3	4501149.8	487269.4
京都市	Kyoto-shi	総 合 計	0.0	0.0	0.0	806922.0	0.0	0.0	1200654.5	5022404.3	562693.7
大阪市	Osaka-shi	総 合 計	0.0	0.0	0.0	722335.5	0.0	0.0	1082255.0	4527165.0	505537.6
神戸市	Kobe-shi	総 合 計	0.0	0.0	0.0	692359.5	0.0	0.0	1028708.5	4302833.2	481821.4
奈良市	Nara-shi	総 合 計	0.0	0.0	0.0	789402.0	0.0	0.0	1237066.8	5174826.4	538427.7
和歌山市	Wakayama-shi	総 合 計	0.0	0.0	0.0	707323.5	0.0	0.0	1095713.1	4583624.6	486574.0
鳥取市	Tottori-shi	総 合 計	0.0	0.0	0.0	779150.0	0.0	0.0	1143329.6	4782625.7	551147.0
松江市	Matsue-shi	総 合 計	0.0	0.0	0.0	763634.0	0.0	0.0	1145538.0	4791787.4	530684.5
岡山市	Okayama-shi	総 合 計	0.0	0.0	0.0	654993.5	0.0	0.0	994408.1	4159625.7	457833.7
広島市	Hiroshima-shi	総 合 計	0.0	0.0	0.0	763644.0	0.0	0.0	1128704.9	4721700.0	538309.6
山口市	Yamaguchi-shi	総 合 計	0.0	0.0	0.0	705806.0	0.0	0.0	1064542.9	4453158.8	493490.7
徳島市	Tokushima-shi	総 合 計	0.0	0.0	0.0	700164.0	0.0	0.0	1037468.9	4339964.6	486814.6
高松市	Takamatsu-shi	総 合 計	0.0	0.0	0.0	715807.0	0.0	0.0	1057884.1	4425232.8	499333.8
松山市	Matsuyama-shi	総 合 計	0.0	0.0	0.0	703011.5	0.0	0.0	1102368.0	4611385.4	481233.8
高知市	Kochi-shi	総 合 計	0.0	0.0	0.0	696895.0	0.0	0.0	1036658.8	4336322.0	486998.9
福岡市	Fukuoka-shi	総 合 計	0.0	0.0	0.0	692352.5	0.0	0.0	1078572.2	4511986.4	477288.2
佐賀市	Saga-shi	総 合 計	0.0	0.0	0.0	770454.5	0.0	0.0	1268574.8	5307098.2	513007.1
長崎市	Nagasaki-shi	総 合 計	0.0	0.0	0.0	697280.0	0.0	0.0	1116933.8	4672739.1	472364.5
熊本市	Kumamoto-shi	総 合 計	0.0	0.0	0.0	694315.5	0.0	0.0	1107586.5	4633531.9	473009.6
大分市	Oita-shi	総 合 計	0.0	0.0	0.0	723637.0	0.0	0.0	1122961.0	4697947.4	497871.8
宮崎市	Miyazaki-shi	総 合 計	0.0	0.0	0.0	665158.0	0.0	0.0	978181.4	4092194.9	469871.6
鹿児島市	Kagoshima-shi	総 合 計	0.0	0.0	0.0	658789.0	0.0	0.0	1033163.7	4322281.7	455038.1
那覇市	Naha-shi	総 合 計	0.0	0.0	0.0	607174.0	0.0	0.0	1032435.7	4319143.1	401846.9
川崎市	Kawasaki-shi	総 合 計	0.0	0.0	0.0	742606.0	0.0	0.0	1076159.9	4501664.8	523564.6
相模原市	Sagamihara-shi	総 合 計	0.0	0.0	0.0	750285.5	0.0	0.0	1150544.2	4812949.7	516237.3
浜松市	Hamamatsu-shi	総 合 計	0.0	0.0	0.0	729741.0	0.0	0.0	1145122.7	4790292.0	494642.7
堺市	Sakai-shi	総 合 計	0.0	0.0	0.0	803962.5	0.0	0.0	1169672.6	4893027.9	564059.7
北九州市	Kitakyushu-shi	総 合 計	0.0	0.0	0.0	700680.0	0.0	0.0	1066939.7	4463844.5	483081.1

		Protein たんぱく質 (g)	アミノ酸組成 たんぱく質 (g)	Fat 脂質 (g)	TG当量 (g)	Fatty acids 飽和脂肪酸 (g)	一価不飽和 脂肪酸 (g)	多価不飽和 脂肪酸 (g)	Cholesterol コレステロール (mg)	Carbohydrate 炭水化物 (g)	利用可能 炭水化物 (g)	低分子量 水溶性 食物繊維 (g)
**全国**	All Japan	35251.0	29597.2	41061.0	38135.7	12021.8	16304.8	8284.5	191037.5	129389.9	121282.1	579.4
札幌市	Sapporo-shi	36740.2	30772.8	45018.2	42079.2	12813.9	18006.3	9613.9	207571.1	134325.8	126210.2	521.7
青森市	Aomori-shi	40301.8	33915.9	46208.4	42905.6	13010.7	18285.5	9945.1	224172.4	146379.4	136052.8	591.7
盛岡市	Morioka-shi	36146.4	30461.0	38095.8	35271.5	11415.0	14704.5	7783.7	187104.8	134650.9	124282.8	557.4
仙台市	Sendai-shi	34521.5	28873.6	37951.0	35197.4	11288.3	14793.1	7761.7	192880.1	122681.4	111595.9	554.5
秋田市	Akita-shi	36809.2	30835.5	42372.7	39425.0	12002.1	16646.2	9217.8	191011.6	137253.4	126201.1	493.1
山形市	Yamagata-shi	36912.2	31123.6	42892.5	39791.5	12592.5	17060.3	8570.2	200694.7	141539.2	132092.7	532.0
福島市	Fukushima-shi	33171.3	27907.0	37986.1	35319.5	10759.1	14957.6	8240.5	183830.8	128299.4	119841.3	513.2
水戸市	Mito-shi	31663.1	26518.1	37376.4	34786.7	10841.9	14600.8	7983.9	168026.5	111052.4	102156.3	495.8
宇都宮市	Utsunomiya-shi	32660.3	27393.3	37848.4	35197.5	11009.9	14832.8	8005.5	179739.2	116145.2	107600.9	569.7
前橋市	Maebashi-shi	30910.5	25954.8	34028.8	31655.9	9869.8	13261.8	7317.6	151039.4	123100.5	115793.0	547.5
さいたま市	Saitama-shi	35507.9	29831.1	41569.3	38713.1	12377.3	16443.2	8380.4	181303.8	127043.7	118196.2	628.0
千葉市	Chiba-shi	36939.7	30856.2	41290.3	38249.5	12378.8	16094.1	8291.4	195688.0	128308.5	118705.5	609.1
東京都区部	Ku-areas of Tokyo	33065.8	27655.8	38284.3	35578.5	11639.5	15077.1	7468.0	171221.4	114817.5	106191.1	589.2
横浜市	Yokohama-shi	36954.2	30828.0	42896.7	39904.4	12978.3	16970.9	8403.3	188811.3	129835.6	121274.9	633.0
新潟市	Niigata-shi	38929.1	32560.1	42736.7	39701.0	12241.0	16652.2	9250.0	207362.5	146336.0	136027.6	626.5
富山市	Toyama-shi	37013.4	31140.1	39795.6	36652.1	12205.9	15456.3	7571.7	199444.5	127522.4	117827.6	636.7
金沢市	Kanazawa-shi	34968.8	29417.5	39242.1	36272.7	11790.8	15414.4	7650.2	192989.7	125307.1	116915.1	614.2
福井市	Fukui-shi	33267.9	27940.8	37304.9	34469.6	10903.1	14836.8	7397.4	197614.3	134401.5	128222.6	528.3
甲府市	Kofu-shi	35471.1	29724.4	41717.4	38894.7	12033.1	16546.4	8823.2	178575.0	128663.3	120868.2	556.2
長野市	Nagano-shi	32825.5	27588.5	38204.5	35512.1	10929.7	14918.8	8275.6	176321.4	124945.3	114565.0	473.7
岐阜市	Gifu-shi	34077.1	28756.8	39089.6	36308.6	11365.9	15435.3	8108.4	186320.8	136594.0	130516.2	567.2
静岡市	Shizuoka-shi	39069.8	32431.7	41054.4	38083.3	12497.5	15882.2	8269.8	190027.5	149166.3	140552.5	658.5
名古屋市	Nagoya-shi	35222.3	29591.3	39496.8	36616.9	11783.1	15565.0	7860.0	187671.9	136328.7	128769.9	622.1
津市	Tsu-shi	35982.8	30238.9	41326.1	38220.1	12083.7	16438.3	8231.8	208510.7	135577.4	129253.7	555.9
大津市	Otsu-shi	34631.7	29045.7	41274.0	38290.3	12333.8	16490.7	7954.8	186621.2	127314.6	120712.5	620.0
京都市	Kyoto-shi	38322.1	32059.2	44539.3	41218.3	13232.1	17856.1	8507.6	207952.6	145099.5	136955.8	683.7
大阪市	Osaka-shi	35432.4	29802.9	42145.8	39015.6	12710.6	17006.9	7770.5	206569.1	124959.1	117940.2	625.9
神戸市	Kobe-shi	33587.3	28123.4	37465.8	34616.8	11483.2	14898.4	6877.4	177063.1	125866.8	118875.7	612.6
奈良市	Nara-shi	39228.5	32688.2	46516.3	43089.1	13835.7	18677.0	8880.1	217308.0	149728.5	142119.3	632.2
和歌山市	Wakayama-shi	36662.8	30780.4	42936.8	39723.5	12704.2	17362.1	8107.6	216404.2	127950.4	121009.2	578.7
鳥取市	Tottori-shi	38080.1	32068.9	46847.3	43411.3	13208.5	18657.2	9824.6	237461.5	127349.6	118367.7	606.8
松江市	Matsue-shi	38227.3	32038.6	43739.6	40453.5	12813.1	17262.3	8778.3	215671.3	133098.5	124309.1	660.7
岡山市	Okayama-shi	33147.6	27956.2	40800.3	37894.0	11894.6	16412.6	8084.5	193883.3	109981.0	103190.6	570.0
広島市	Hiroshima-shi	35752.5	29961.1	44632.2	41415.3	12904.3	17863.5	8988.2	196934.0	129180.0	120298.6	594.2
山口市	Yamaguchi-shi	34924.0	29442.8	42763.2	39623.8	12315.3	17220.5	8511.3	205474.3	118666.1	110999.6	573.7
徳島市	Tokushima-shi	32712.9	27475.7	37062.4	34294.7	11039.1	14777.8	7144.7	166619.4	128172.4	120499.1	585.9
高松市	Takamatsu-shi	34407.6	28935.1	38920.4	35953.4	11498.4	15336.1	7698.5	193078.5	129305.0	121759.7	587.5
松山市	Matsuyama-shi	34402.8	29039.8	42345.7	39252.7	12081.2	16956.0	8669.5	205705.0	131417.8	125275.8	579.9
高知市	Kochi-shi	34314.3	28690.9	39568.4	36612.5	11042.7	15945.1	8217.9	201816.7	118780.4	110457.0	553.8
福岡市	Fukuoka-shi	35245.9	29651.8	43528.6	40476.1	13009.7	17668.5	8175.6	194998.2	121872.3	115195.7	584.4
佐賀市	Saga-shi	38319.6	32155.8	47220.8	43938.4	12841.3	19012.2	10336.3	195960.3	155452.5	148255.3	614.9
長崎市	Nagasaki-shi	34778.1	29211.7	43209.2	40204.1	11936.9	17385.4	9284.3	192967.9	132022.4	124824.2	548.7
熊本市	Kumamoto-shi	34124.4	28528.6	43797.1	40786.0	12116.4	17849.9	9195.1	194487.1	127925.8	119866.9	519.6
大分市	Oita-shi	35914.3	30114.4	44874.6	41618.4	12589.4	18134.9	9233.0	204881.2	127966.3	119264.8	550.3
宮崎市	Miyazaki-shi	31106.7	26025.9	38427.5	35733.4	11090.5	15413.9	7805.0	172727.6	109524.9	101072.1	491.9
鹿児島市	Kagoshima-shi	32772.4	27484.6	43392.6	40511.2	11760.4	17464.0	9671.1	191376.8	112937.5	104883.8	536.7
那覇市	Naha-shi	32126.7	27262.6	40946.7	38279.0	11452.7	16579.2	8779.4	183349.2	120160.8	115341.2	495.6
川崎市	Kawasaki-shi	35986.9	30130.0	40388.9	37521.4	12484.2	15922.5	7648.7	183999.8	127852.9	118833.2	629.2
相模原市	Sagamihara-shi	37272.5	31275.6	43753.0	40761.4	12815.7	17369.3	8993.4	186646.2	138004.9	129944.9	658.3
浜松市	Hamamatsu-shi	37169.4	31243.9	40903.5	37979.1	12210.9	15816.1	8505.2	197237.6	143196.3	136382.1	592.1
堺市	Sakai-shi	37842.2	31544.8	42829.3	39595.3	12841.0	17053.7	8158.7	204870.3	142826.1	134468.9	683.4
北九州市	Kitakyushu-shi	35133.2	29316.6	40033.6	36985.3	11510.3	16172.6	7826.0	189046.3	127320.2	119046.5	551.4

Dietary fibers			Ash	Salt	Na	K	Ca	Mg	P	Fe	Zn	Cu	Mn	I	Se	Cr	Mo	Vitamin A				
高分子量 水溶性 食物繊維 (g)	不溶性 食物繊維 (g)	食物繊維 総量 (g)	灰分 (g)	食塩 相当量 (g)	ナトリウム (mg)	カリウム (mg)	カルシウム (mg)	マグネシウム (mg)	リン (mg)	鉄 (mg)	亜鉛 (mg)	銅 (mg)	マンガン (mg)	ヨウ素 (μg)	セレン (μg)	クロム (μg)	モリブデン (μg)	レチノール (μg)	α-カロテン (μg)	β-カロテン (μg)	β-クリプト キサンチン (μg)	β-カロテン 当量 (μg)
1822.5	5333.0	8207.3	9562.0	5905.1	2336014.0	1234859.2	244767.7	125563.1	507105.9	3828.6	4180.5	524.5	1923.3	859081.7	45807.7	3816.1	102691.1	123059.9	292476.4	1339527.9	243165.1	1732175.6
1834.0	5359.1	8083.2	9910.1	6211.7	2455747.1	1251669.1	232938.5	125034.7	526592.5	3822.7	4351.3	542.8	1984.1	903096.2	48060.7	3864.4	104901.4	144905.2	282641.7	1302104.0	243289.0	1693119.8
1989.9	5986.5	9463.8	11721.2	7596.9	3002840.1	1413855.4	259412.5	147685.6	573455.3	4335.1	4667.2	609.4	1923.7	1740900.0	55791.4	4389.3	120057.0	148710.6	351175.3	1582198.5	217592.6	1948246.6
1932.0	5674.6	9004.0	12074.9	8171.0	3227959.1	1314533.0	266474.1	136662.6	526589.8	3903.7	4136.3	543.3	1817.3	1280920.0	47810.1	3972.5	108786.8	143394.8	347752.6	1464003.2	248204.3	1850805.6
1930.0	5741.5	9048.8	10946.4	7129.8	2818181.4	1293863.1	255784.9	131768.2	506240.9	3881.0	4053.7	523.6	2016.6	1408160.2	45664.5	3883.8	97407.3	147275.7	321336.6	1466671.3	285439.4	1930085.9
1875.3	5816.8	8930.1	11840.2	7928.7	3132051.6	1334743.1	256382.3	136412.7	533025.9	4057.1	4309.5	561.4	1959.4	1185606.3	47856.0	4027.9	108368.5	137879.2	297608.1	1478800.2	284807.9	1883195.5
1802.9	5409.0	8389.1	13510.2	9557.1	3770031.5	1336761.3	264718.2	137580.9	544009.2	3999.0	4484.6	562.0	1887.4	1603882.5	47248.2	3940.4	115358.6	148531.5	287146.6	1394442.2	202666.6	1723341.0
1813.0	5177.1	8209.7	10289.3	6731.7	2660312.1	1202565.6	231398.2	124182.3	487592.9	3642.2	3985.1	519.5	1737.6	1448088.7	43075.6	3873.3	105256.3	138091.2	358165.5	1415707.8	223170.4	1787574.6
1709.9	5024.1	7662.0	8711.3	5211.5	2063419.2	1193565.7	239739.5	116223.2	470213.4	3484.1	3620.5	459.5	1713.6	598584.5	42404.9	3451.2	84723.8	136418.9	282349.0	1335971.5	269782.1	1732111.5
1785.1	5119.6	8029.6	8185.2	4671.8	1853086.3	1184375.0	240399.1	120587.5	475549.5	3606.5	3758.9	477.6	1893.6	826248.4	43190.7	3895.4	94524.8	146001.3	318192.2	1378275.9	260721.2	1816599.7
1734.8	4955.4	7581.6	8414.4	5071.0	2007869.2	1127471.2	217299.1	116099.1	452089.9	3402.7	3633.3	489.0	1847.3	664294.9	40529.9	3588.4	99684.1	129027.2	273262.6	1276302.5	222030.4	1645053.5
1956.0	5509.0	8569.7	9142.3	5379.8	2131326.2	1265826.8	255613.8	127827.0	515756.9	3791.5	4157.1	514.6	1863.7	676320.8	45923.3	3830.3	102336.9	147058.3	316048.8	1505745.6	237140.1	1894353.8
2047.7	5899.4	9120.6	10019.8	5951.9	2355834.3	1391139.5	282087.1	136019.7	545808.9	4058.0	4277.6	538.2	2049.4	1126163.2	50161.5	4298.0	100814.4	163424.8	311636.1	1578230.0	283277.0	2025261.0
1864.6	5361.3	8235.6	8317.0	4697.7	1862325.4	1225522.4	248286.9	120987.1	486597.2	3632.2	3916.4	489.8	1816.6	725934.5	42599.4	3839.5	92946.5	138765.3	295014.0	1439391.8	224690.4	1822764.6
2018.0	6008.9	9036.5	8978.0	5048.9	2001081.7	1330700.5	262083.1	131637.9	534319.7	4060.4	4372.1	554.3	2225.5	634522.9	48001.9	4061.6	104779.1	156089.5	319470.9	1571285.9	200670.8	2008478.5
2110.5	6367.4	9596.8	11892.0	7696.4	3039787.7	1433743.1	263839.9	144426.8	567631.3	4320.5	4621.0	620.0	2246.1	1005614.2	52259.7	3973.9	120390.2	141779.8	377406.1	1686517.4	293449.1	2164354.0
1930.9	5722.7	8835.4	10015.3	6130.5	2425382.1	1322141.0	262057.4	131776.8	530630.0	3982.8	4257.1	535.2	1994.5	1335210.7	50848.6	3922.0	103651.2	149096.6	277010.0	1348056.1	256324.7	1762702.8
1830.1	5378.4	8309.3	9598.2	5947.8	2352787.6	1229245.2	251203.6	124633.9	502218.6	3775.9	4143.3	518.2	1937.5	950087.7	47567.4	3719.1	101444.2	135470.1	306751.3	1355018.4	240713.8	1767998.6
1630.0	5002.0	7775.7	9471.7	6093.3	2407237.5	1128608.7	228845.6	118939.5	482958.8	3814.3	4212.3	526.6	2184.2	1157540.4	43558.2	3525.1	109576.7	133123.0	248611.1	1132328.0	234060.8	1552311.5
1858.6	5445.7	8435.2	9812.0	6100.2	2414231.9	1256565.5	243914.5	128840.3	510169.4	3935.5	4190.7	536.9	2046.1	757758.8	45615.5	3865.8	106806.3	151927.3	312609.2	1456067.2	259050.9	1885336.3
1822.6	5381.5	8291.9	10001.1	6422.4	2538614.2	1220062.5	245265.8	121123.1	485180.8	3588.0	3781.0	488.6	1638.7	1053498.0	44357.3	3570.2	91344.2	140649.1	285888.4	1329823.5	253937.6	1680154.5
1632.3	4923.1	7540.6	8503.8	5067.9	2005755.0	1140840.9	231072.1	120824.1	491995.7	3680.3	4173.5	528.2	1919.9	678290.6	44141.5	3535.6	112838.7	139273.2	260606.8	1148560.7	196451.4	1485255.1
2107.4	6611.9	9919.3	9571.1	5417.0	2146143.8	1402617.8	287603.3	144228.3	574582.4	4504.3	4688.7	616.8	2953.2	807849.2	50694.5	4124.5	120969.3	172609.4	330134.6	1553494.0	441022.0	2254599.8
1859.8	5475.9	8217.0	8193.9	4543.5	1801224.8	1230464.2	240624.3	124669.4	505449.0	3805.4	4213.0	539.9	1971.7	508716.1	45475.5	3664.2	107699.2	145572.7	282223.5	1320846.0	200550.4	1689815.3
1716.5	5134.7	7701.7	9521.5	5910.0	2335877.8	1211435.7	243334.9	123246.1	516705.3	3830.6	4326.0	531.0	2119.0	659710.1	46893.6	3538.6	107408.5	159034.3	285795.8	1226107.1	261500.5	1659924.9
1803.5	5205.8	7930.9	8413.3	4883.2	1933527.1	1179670.2	243724.5	118874.8	495071.7	3658.2	4093.6	499.0	2044.2	553457.1	45000.4	3741.2	98007.4	135780.6	283826.4	1271221.2	176937.0	1667958.7
2119.3	6068.8	9265.0	9838.0	5783.1	2290234.7	1361961.2	272446.0	136520.3	551092.1	4172.2	4699.7	576.0	2366.3	990236.9	49994.4	4254.9	109185.4	146969.8	273863.1	1395949.8	265393.5	1857122.6
1853.6	5107.3	7906.8	8458.8	4852.9	1922621.6	1210237.0	240709.6	120505.0	501510.8	3641.0	4237.6	501.8	1767.7	753333.5	45980.4	3737.7	96431.7	144858.5	272657.5	1270475.1	178605.4	1595178.6
1788.3	5157.4	8010.4	8282.4	4818.8	1908625.3	1159320.6	240604.1	118438.9	479954.8	3528.9	4024.2	494.8	1878.3	555261.4	44032.0	3785.3	93100.5	127390.2	259012.8	1209043.9	227407.9	1580091.0
2019.0	6350.4	9397.1	9624.3	5559.3	2201080.2	1365488.3	276350.3	137035.9	560226.2	4376.7	4841.2	600.2	2909.8	898812.7	50105.5	4043.8	114443.9	154975.1	305372.4	1408577.6	262096.5	2004498.8
1842.4	5107.0	7988.1	8756.4	5129.0	2031700.5	1231743.7	238679.8	124265.5	509807.7	3738.1	4283.2	510.6	1803.4	907043.0	48482.0	4285.1	95446.7	150484.4	253168.6	1205663.5	238647.7	1560766.1
1782.1	5143.7	8037.3	9937.1	6110.0	2415893.0	1282918.9	264023.5	131135.2	537169.3	3936.6	4588.7	540.8	1754.7	813717.7	56532.1	3886.6	98742.3	155810.6	257477.7	1273726.7	291304.5	1646047.8
1880.1	5545.4	8789.7	10981.9	7111.5	2810054.1	1307338.6	271792.3	137340.0	544085.6	4166.0	4451.7	543.5	2215.2	1137109.9	51774.6	4186.8	106941.5	142733.1	314755.1	1392835.0	286352.3	1861047.6
1658.9	4458.8	6970.5	8012.7	4757.6	1883999.6	1098803.5	217995.1	110466.1	457386.7	3330.2	3838.5	450.9	1468.8	675919.8	44672.5	3565.3	84174.3	129864.1	245067.9	1127343.6	224906.6	1429045.7
1930.9	5578.8	8416.9	8934.7	5130.3	2032273.3	1278747.7	257749.6	127860.2	515020.3	3906.2	4464.0	539.8	2052.9	735435.5	44339.3	3989.7	100562.4	136450.2	313962.4	1434429.2	276104.8	1883924.9
1707.7	4707.7	7346.3	9395.5	5912.5	2336468.4	1174499.5	231512.4	119874.7	491213.4	3603.0	4072.6	485.8	1555.6	769194.8	45494.1	3623.9	95439.3	135234.6	292979.7	1270595.4	241980.8	1607370.9
1784.5	5156.0	8014.2	9169.6	5700.0	2254030.6	1167995.6	232923.5	121431.1	471421.6	3644.7	3999.7	508.5	1721.7	545538.9	40127.8	3671.4	102325.5	124540.9	248241.6	1248661.0	305927.5	1600458.8
1865.9	5138.3	7974.8	8969.6	5364.8	2124634.5	1202184.3	247171.6	122594.5	491745.1	3606.3	4336.0	517.2	1698.5	681765.2	45009.4	3933.7	95352.5	134498.5	301208.3	1331344.5	291980.5	1703557.0
1702.9	4840.4	7412.6	8392.1	4926.2	1949690.7	1158914.5	231377.0	118369.3	488697.3	3627.4	4174.4	505.3	1741.3	682365.9	43821.4	3476.8	101882.0	138889.1	277932.6	1234057.9	292079.0	1604250.7
1652.2	4779.2	7490.4	10150.0	6743.6	2662352.2	1147133.4	212782.0	119658.8	473218.3	3678.2	3896.0	481.1	1690.3	1010748.5	46075.5	3449.5	92411.8	143973.2	228501.6	1169291.4	264075.2	1509873.4
1762.8	4982.0	7811.2	8962.2	5441.8	2151238.7	1178718.7	234067.1	119557.0	496169.8	3616.8	4210.8	501.5	1747.4	698351.0	43670.3	3523.6	98688.2	129169.3	322934.0	1331120.6	214978.2	1700169.3
1905.8	5754.7	8892.3	9942.1	6068.1	2400768.8	1306851.5	248071.4	139803.2	545082.4	4324.6	4782.5	612.6	2300.3	745916.6	46978.9	4044.3	127602.0	128847.0	313908.2	1413734.4	287667.9	1858035.6
1723.4	5096.7	7948.8	9105.1	5607.4	2217298.5	1184235.0	226994.3	123795.2	493387.2	3863.0	4361.0	536.5	1946.7	877210.2	43526.2	3797.9	108210.3	124874.2	291065.6	1302492.2	288509.5	1711824.2
1664.7	5195.5	7786.6	8960.1	5534.8	2187691.8	1159214.1	221667.6	119525.7	483293.1	3853.4	4178.4	518.7	2153.4	541548.1	41656.9	3516.5	102620.3	130742.1	270789.2	1252881.7	266760.4	1709489.4
1791.9	5384.9	8289.6	10576.9	6898.3	2723970.8	1247306.6	245501.7	128767.2	510357.8	3980.7	4318.3	529.5	1993.8	741966.4	44675.0	3742.6	103745.7	137378.9	272493.3	1273761.6	333660.8	1716953.8
1629.3	4836.5	7201.7	8415.4	5147.8	2035900.8	1113635.7	219233.4	109247.3	446324.0	3404.5	3637.0	447.5	1674.1	503621.8	38391.5	3315.5	85840.6	125498.0	320069.4	1294362.7	243019.7	1692378.9
1733.9	5084.0	7655.1	8671.2	5331.6	2108419.8	1142210.9	212895.5	113891.6	457965.3	3597.7	3782.4	473.0	1808.8	556156.8	41222.0	3449.6	91362.3	127636.3	319221.9	1340193.7	255174.7	1767393.1
1470.8	4191.1	6424.1	7620.2	4549.1	1800604.9	1021947.4	190037.8	104537.6	448340.6	3403.9	3843.5	458.1	1487.9	986919.9	44654.5	3079.5	98895.5	143374.3	384432.7	1389348.4	162698.0	1716636.1
2033.9	5808.7	8994.9	9044.2	5155.1	2042603.8	1327961.9	257760.0	129211.5	519750.7	3840.9	4212.2	531.1	1898.9	1513414.2	46970.7	4022.1	98765.4	146411.4	338810.6	1614743.9	207933.8	2000607.9
2011.6	5761.4	9029.5	9786.8	5929.5	2347080.7	1295734.5	259064.1	132963.4	535066.7	3966.6	4383.5	551.3	2027.0	846802.1	48486.2	4090.5	110040.9	147610.7	340736.8	1526384.1	221947.8	1926186.8
1774.4	5447.3	8197.1	8995.6	5197.7	2059095.9	1257059.1	268783.4	131386.9	546351.9	4102.8	4464.7	569.5	2336.4	645221.2	48392.9	3729.4	119407.3	167138.4	292567.9	1301444.7	374959.5	1829758.4
2147.8	6243.0	9532.9	10236.9	6125.8	2423478.3	1395127.6	275148.5	138485.5	549234.1	4160.6	4563.2	580.3	2461.9	1044284.1	47393.7	4278.0	111059.2	148631.7	346612.4	1537380.3	283523.4	2062857.5
1866.6	5623.5	8610.7	9339.9	5675.3	2245030.5	1261052.4	231700.8	128553.0	496768.8	3978.5	4327.2	548.5	2166.7	874171.3	44825.1	3956.2	103938.2	118707.7	297669.8	1445678.9	240235.2	1890173.3

	Vitamin D	Vitamin E				Vitamin K	Vitamin B									Vitamin C						
レチノール活性当量 (μg)	ビタミンD (μg)	α－トコフェロール (mg)	β－トコフェロール (mg)	γ－トコフェロール (mg)	δ－トコフェロール (mg)	ビタミンK (μg)	ビタミンB_1 (mg)	ビタミンB_2 (mg)	ナイアシン (mg)	ナイアシン当量 (mg)	ビタミンB_6 (mg)	ビタミンB_{12} (μg)	葉酸 (μg)	パントテン酸 (mg)	ビオチン (μg)	ビタミンC (mg)	アルコール (g)	硝酸イオン (g)	テオブロミン (g)	カフェイン (g)	タンニン (g)	ポリフェノール (g)
267065.6	4746.1	6019.7	260.8	7840.9	1512.7	126107.3	513.8	542.3	8526.6	15681.7	709.3	3449.8	153750.5	2906.4	21112.8	58151.7	5902.5	62.8	0.0	46.2	219.7	0.8
285629.1	5546.0	6548.0	283.0	9109.5	1713.3	125796.2	541.7	555.8	9264.7	16702.5	743.3	3580.5	157258.6	3013.7	22113.6	60545.8	8356.0	63.8	0.0	51.5	242.8	1.1
310713.2	6868.6	6869.2	266.2	9277.2	1829.3	147200.3	588.4	596.9	9879.8	18028.0	823.3	4338.4	171154.2	3199.9	24944.5	64078.5	7850.5	70.3	0.0	40.0	182.9	1.1
297227.3	5700.2	5672.2	231.7	6918.7	1406.8	131251.5	522.9	556.4	8705.1	16079.1	738.9	3643.6	156924.7	2974.7	22008.6	58479.2	7369.7	67.3	0.0	43.5	198.7	1.0
307848.7	4944.4	6153.7	258.8	7052.6	1398.4	139265.2	517.4	546.9	8550.6	15578.1	714.9	3646.6	165475.9	2855.8	21884.0	64545.9	6683.0	67.6	0.0	53.1	256.9	0.7
294522.7	5635.2	6331.2	273.1	8724.6	1728.6	145412.7	551.5	562.1	9317.6	16766.7	762.5	3878.4	167184.9	3033.1	22230.7	65004.1	7700.5	71.1	0.0	42.9	203.8	0.9
291611.7	5058.5	6024.1	244.1	7925.8	1560.1	126919.0	533.0	576.4	8928.2	16437.0	733.2	3371.5	156681.4	3104.7	22700.8	56905.5	6705.7	59.6	0.0	42.4	193.1	0.7
286739.2	4818.9	5736.0	230.5	7822.0	1511.8	123948.4	497.6	502.3	8157.0	14928.2	681.4	3280.8	146662.9	2760.3	20481.0	55752.6	6344.0	62.6	0.0	33.4	155.5	1.3
280532.3	4551.4	5700.6	249.9	7652.8	1473.6	122513.4	483.7	509.1	7858.0	14278.1	673.5	3506.4	150454.0	2647.2	19404.7	60539.6	4503.8	64.0	0.0	43.3	207.2	0.7
297125.1	4451.5	5917.8	264.2	7552.2	1459.7	127530.2	492.2	515.7	7892.3	14535.4	659.4	3009.7	153989.6	2706.5	20285.4	58322.8	6434.7	65.9	0.0	48.8	238.2	1.3
265929.3	4223.2	5285.5	241.9	6902.9	1350.6	110477.3	467.4	461.1	7519.4	13809.1	647.2	2895.2	140564.1	2583.6	18257.2	54825.2	5486.4	55.7	0.0	42.6	205.0	0.9
304604.7	4416.0	5911.9	261.3	7835.7	1517.7	131867.3	528.9	544.5	8708.7	15907.3	714.0	3370.8	159599.5	2940.6	20845.7	61337.7	6045.2	68.5	0.0	45.9	215.3	0.7
331853.6	5360.9	6418.5	263.9	7677.8	1471.2	142114.9	545.9	596.4	9264.7	16749.3	767.1	4258.5	175521.7	3104.9	22643.8	70011.2	5435.2	76.0	0.0	53.4	255.0	1.4
290408.9	4268.8	5578.3	232.7	6846.1	1325.9	128062.1	498.1	522.9	8093.0	14795.0	679.3	3365.8	157430.1	2770.3	19789.1	62455.1	5837.6	67.1	0.0	48.3	226.9	1.0
323224.7	4601.5	6433.3	282.8	7757.3	1483.4	141921.6	555.4	567.9	9125.2	16592.0	741.4	3618.5	172435.8	3038.4	21565.9	66057.6	6257.5	71.1	0.0	67.4	317.6	0.8
321828.7	6477.7	6853.5	275.3	8646.9	1701.6	138706.5	597.2	606.6	9681.9	17569.9	820.1	4301.2	179607.9	3278.9	24498.3	71403.2	7558.3	67.4	0.0	52.6	249.7	0.5
295528.6	5471.2	6076.7	249.9	6509.3	1304.6	123353.0	555.5	588.9	8930.0	16453.2	737.9	3599.9	163196.2	3023.1	22482.5	62071.9	5865.1	64.9	0.0	47.4	232.3	0.5
282418.8	4865.2	6013.4	259.6	7013.0	1382.9	120980.1	510.0	560.7	7970.6	15092.7	691.1	3284.6	154103.4	2904.4	21738.8	57956.6	5161.4	59.9	0.0	44.7	217.9	0.6
262111.0	4452.0	6069.8	257.9	6913.3	1307.9	120922.8	478.7	534.2	7479.3	14336.2	642.7	2930.2	145239.5	2816.5	21230.7	50372.0	4472.0	52.7	0.0	49.8	250.9	0.6
308878.5	4802.5	6097.4	276.8	8321.4	1629.2	140072.7	533.4	521.7	8758.7	15949.6	716.3	3899.9	159343.3	2863.1	20466.6	60923.9	5374.1	74.4	0.0	46.6	226.6	0.7
280281.9	4833.4	5854.9	248.5	7974.7	1534.6	129878.3	487.7	518.2	7932.6	14579.4	672.3	3366.6	149995.8	2732.7	20557.4	59276.9	6227.9	66.5	0.0	39.8	180.5	0.4
262589.5	4315.9	5694.4	242.7	7613.4	1480.2	112113.9	493.3	515.0	7798.7	14813.5	664.3	2974.5	140148.1	2839.5	20515.4	52471.3	4692.9	55.1	0.0	40.3	191.7	0.7
360437.8	5031.5	7037.5	338.9	7534.0	1486.3	152848.3	588.9	598.4	9564.1	17532.2	799.9	3859.2	188579.3	3238.3	22622.7	71348.8	5256.4	68.2	0.0	74.4	387.8	0.7
285982.7	4263.6	5875.3	253.5	7216.6	1416.3	114941.2	512.0	533.0	8339.2	15539.3	710.0	3322.2	154865.6	2933.2	20852.9	58556.5	5200.2	59.8	0.0	46.9	223.7	0.6
296980.0	4835.1	6324.6	274.2	7631.3	1433.4	115666.1	498.2	560.1	8219.7	15570.8	712.5	3299.3	152330.9	3004.1	22067.6	55942.2	4803.4	55.3	0.0	47.0	234.4	0.6
274362.2	4515.2	6052.6	281.9	7499.4	1414.3	121582.9	486.4	543.1	8012.3	15036.0	667.0	3083.0	149746.0	2889.5	20713.5	53566.2	5123.0	55.9	0.0	59.9	287.2	0.9
301292.0	4777.9	6791.4	319.4	8072.9	1514.9	137841.9	544.1	604.5	9030.8	16792.2	756.2	3599.8	176573.4	3237.6	23193.3	63974.0	6180.5	72.3	0.0	69.2	333.2	0.8
277260.1	4453.4	5801.7	240.5	7017.7	1336.2	116429.6	499.4	548.7	8466.0	15675.4	695.5	3024.2	149458.8	2944.0	21138.3	54876.2	5719.9	63.2	0.0	45.6	209.9	0.8
258636.9	4115.7	5472.2	241.8	6204.8	1179.7	119439.4	471.4	520.6	7891.3	14671.1	647.3	2885.6	144329.5	2773.3	19964.6	54602.0	5110.5	56.3	0.0	59.5	273.7	0.7
321713.2	4854.6	7500.1	371.2	8338.5	1543.4	151237.1	555.4	623.8	8993.1	16995.7	774.2	3228.7	183565.0	3324.2	23801.7	65700.0	5495.1	67.0	0.0	86.7	436.5	0.6
280028.9	4908.1	6108.6	253.4	7361.6	1385.8	122150.8	517.1	550.7	8783.1	16194.9	725.4	3442.1	150689.6	2963.4	21727.8	56658.5	4390.9	61.8	0.0	41.8	198.7	1.9
292340.8	5943.6	6974.8	284.1	9527.0	1806.9	127941.9	532.5	636.6	8796.4	16529.1	725.7	4312.8	153474.4	3096.1	24870.7	58811.3	5802.9	56.9	0.0	49.1	226.9	0.8
297429.6	5596.5	6668.7	287.1	8182.5	1558.5	144782.0	535.3	618.1	8693.7	16406.4	748.1	4040.6	164369.8	3113.5	24099.7	58475.2	6766.5	63.6	0.0	59.1	284.4	0.8
248462.0	4287.9	5554.2	235.1	7521.9	1429.7	106118.5	469.6	506.6	7879.9	14583.2	633.2	3164.5	130889.6	2638.8	19797.1	49958.3	5164.5	54.1	0.0	40.1	179.0	0.8
293024.0	4684.8	6499.8	301.6	8722.3	1692.5	131800.8	518.5	572.4	8494.5	15774.6	727.7	3535.9	162286.0	3035.2	21615.0	61106.5	6622.2	66.8	0.0	56.4	269.9	0.8
268622.0	5044.7	5937.2	235.1	8057.4	1557.4	109909.2	491.8	543.8	8299.8	15384.2	687.9	3262.3	139928.2	2848.0	21198.0	52067.1	6545.4	55.5	0.0	34.6	158.2	0.7
257526.1	3781.2	5152.7	212.7	6497.9	1286.4	118735.4	476.1	497.5	7795.8	14435.7	668.3	2918.4	145525.2	2755.7	19381.8	57707.0	6117.5	62.5	0.0	40.6	184.6	0.7
275893.8	4339.7	5704.6	252.6	7125.8	1366.6	113507.4	487.0	547.2	7748.2	14709.1	678.3	3571.9	146682.6	2896.5	21076.8	55996.6	4850.9	58.6	0.0	39.2	178.0	0.8
271991.0	4403.5	6056.6	262.6	8358.4	1611.3	108857.3	487.8	541.3	7835.1	14896.3	673.5	3061.5	140111.4	2897.4	21335.7	51923.0	5170.9	51.4	0.0	40.8	188.6	0.5
269378.8	4544.0	5949.0	244.2	7800.9	1476.5	120805.9	465.4	508.1	8921.8	15858.6	693.6	3433.2	143755.1	2705.8	20602.3	52521.3	7121.5	60.5	0.0	41.3	192.9	0.5
270576.6	4177.6	5727.4	229.9	7393.0	1393.1	122751.0	509.7	528.3	8993.5	16175.0	701.9	3056.0	142301.8	2910.8	20148.7	53746.2	5326.6	58.2	0.0	45.0	208.1	0.5
283397.9	5049.0	6862.9	315.2	10353.2	1992.7	144963.4	552.5	561.2	9160.0	16957.8	773.8	3913.2	163335.8	3205.2	22525.7	59889.1	6325.7	65.8	0.0	46.5	227.5	0.8
267235.3	4411.5	6291.9	273.6	9173.1	1756.4	130215.5	504.7	524.7	8314.4	15413.1	693.1	3358.3	147103.3	2885.6	20978.5	56079.5	5695.6	56.6	0.0	43.7	209.4	0.9
272960.1	3958.0	6620.2	305.1	9168.6	1725.9	131183.7	490.4	519.3	8236.0	15182.7	673.3	2823.0	150377.0	2848.4	20704.3	55014.1	6318.7	54.2	0.0	52.1	262.2	0.6
280120.2	4752.0	6531.0	284.6	9037.3	1744.3	136278.3	511.5	562.7	8643.2	15945.7	723.1	3267.5	154719.5	2999.3	22040.1	56653.5	6355.4	61.4	0.0	48.5	233.6	0.4
266253.5	3814.9	5496.7	236.5	7508.1	1440.6	114651.8	450.0	494.0	7680.7	13988.5	634.4	2843.3	139721.5	2605.4	18690.4	53223.2	7639.8	60.2	0.0	41.8	199.3	0.7
274649.2	4104.3	6445.0	301.0	9804.8	1891.5	124486.5	483.1	502.1	8028.0	14689.7	666.7	3064.2	145450.2	2677.6	20173.2	55164.3	5872.6	60.1	0.0	43.1	211.9	0.5
286129.6	3851.9	5447.4	238.0	8534.5	1668.8	98552.6	460.0	457.2	7973.7	14624.4	604.3	2385.3	122290.1	2507.1	18568.2	43804.5	4528.7	50.8	0.0	32.8	144.9	0.9
312811.0	4499.4	5786.7	240.6	6668.3	1288.7	133681.5	544.8	551.8	8917.8	16199.1	730.2	3480.0	166161.1	2996.2	20715.2	66024.9	5624.8	70.6	0.0	48.6	222.3	0.7
307890.1	4809.6	6162.2	273.0	8364.9	1583.7	143271.5	556.3	551.3	8950.1	16496.5	745.6	3454.8	164507.4	3032.5	21376.0	63026.7	5048.4	72.6	0.0	51.1	235.4	0.7
319308.1	4793.3	6407.4	292.7	7894.2	1564.3	123245.2	549.6	575.3	8616.5	16275.8	719.5	3405.2	159567.6	3057.1	22124.1	59166.4	4748.8	57.9	0.0	51.6	262.7	0.6
320175.2	4853.6	6731.2	300.6	7538.1	1423.3	145247.6	548.7	603.2	8990.9	16680.1	782.1	3083.5	180693.1	3260.6	22998.0	67285.5	5915.7	72.7	0.0	68.4	332.8	0.9
275959.8	4644.8	6192.5	261.2	7244.1	1411.8	135342.3	507.8	533.4	8976.3	16080.4	725.4	3695.1	158186.4	2923.0	21096.1	61030.2	5546.2	64.7	0.0	54.5	266.9	0.7

酢酸 (g)	調理油 (g)	有機酸 (g)	重量変化率 (%)	備考欄 (メモ欄)	イソロイシン(mg)	ロイシン (mg)	リシン (リジン) (mg)	含硫アミノ酸			芳香族アミノ酸			トレオニン (スレオニン) (mg)	トリプトファン (mg)	バリン (mg)	ヒスチジン (mg)	アルギニン (mg)	アラニン (mg)	アスパラギン酸 (mg)	グルタミン酸 (mg)	グリシン (mg)
								メチオニン (mg)	シスチン (mg)	含硫 アミノ酸合計 (mg)	フェニル アラニン (mg)	チロシン (mg)	芳香族 アミノ酸合計 (mg)									
189.0	0.0	1013.9	0.0	0.0	1482786.3	2672752.9	2188814.5	792643.5	531828.9	1326372.2	1570107.3	1236112.4	2828113.3	1385713.0	418329.5	1787814.4	1134833.2	2067107.6	1724552.5	3104193.3	6461237.5	1536070.0
206.1	0.0	981.1	0.0	0.0	1547678.9	2789008.4	2315600.4	840699.8	553298.0	1395159.6	1626275.6	1292183.4	2942337.9	1454692.3	436059.6	1872493.4	1172810.2	2209575.3	1839730.7	3260572.8	6595328.4	1655162.4
222.7	0.0	1171.3	0.0	0.0	1707556.5	3073072.3	2553127.0	924436.4	612644.0	1538243.4	1801382.2	1417143.3	3244173.2	1608150.0	481339.2	2054638.9	1290016.5	2440373.9	2033985.3	3640391.0	7286522.6	1850889.1
157.6	0.0	1094.7	0.0	0.0	1532622.8	2765403.9	2254607.8	816432.4	549239.5	1367663.2	1629217.1	1277947.7	2928858.5	1430669.5	434604.3	1850517.3	1174774.6	2128710.8	1774320.3	3212870.6	6707211.6	1587774.8
159.2	0.0	1046.1	0.0	0.0	1455562.3	2616088.3	2163612.1	775935.0	517952.4	1295396.8	1535601.4	1217095.6	2774097.4	1364982.5	412406.1	1757804.4	1131882.8	2027013.3	1690527.9	3076819.3	6263070.9	1504397.6
178.1	0.0	1078.1	0.0	0.0	1555107.4	2798929.0	2324181.9	833206.8	547925.7	1382867.7	1636940.1	1289644.6	2947510.6	1460055.1	438284.5	1874455.9	1191324.6	2193866.9	1838897.9	3305638.8	6652567.1	1642066.0
206.1	0.0	1111.1	0.0	0.0	1568608.8	2830698.6	2298341.6	830164.9	562071.2	1394562.4	1663881.7	1306990.2	2994338.0	1460522.5	440907.4	1898592.5	1185353.7	2178342.3	1816663.1	3298893.9	6794577.7	1612070.5
181.2	0.0	1007.3	0.0	0.0	1405947.0	2536842.3	2070290.9	755659.9	509755.4	1266606.0	1491962.0	1176141.1	2691358.7	1319100.3	399866.2	1709956.3	1087391.7	1989647.4	1651870.4	3007155.8	6017540.1	1458598.5
158.9	0.0	1004.9	0.0	0.0	1334978.6	2400326.9	1998467.8	710263.4	465736.5	1177910.0	1401741.2	1110040.5	2531016.8	1249054.0	375775.9	1609694.5	1052433.6	1823213.0	1541296.7	2791990.4	5799757.2	1366441.9
154.3	0.0	984.7	0.0	0.0	1378169.4	2476265.4	2029047.9	728698.5	493867.2	1224176.0	1458386.4	1148218.3	2626271.5	1286009.4	389018.9	1662775.0	1064252.2	1897500.9	1583823.5	2873477.8	6035446.0	1404718.7
179.8	0.0	987.8	0.0	0.0	1295267.4	2342599.4	1880875.4	685535.9	473064.3	1160338.7	1387688.3	1083528.6	2489296.8	1205226.0	367969.8	1571364.5	1000168.1	1809735.2	1496612.2	2717815.7	5797692.9	1337938.3
169.0	0.0	1038.7	0.0	0.0	1493401.3	2692935.9	2199936.8	788833.5	532107.9	1323351.0	1583664.9	1245503.5	2848863.2	1391258.8	421372.0	1802236.3	1150021.8	2060806.2	1722013.4	3101601.5	6625433.1	1538472.0
183.2	0.0	1163.6	0.0	0.0	1557396.3	2796901.6	2338086.0	830944.6	540522.9	1374026.8	1630565.5	1294566.8	2944161.9	1457178.7	437374.2	1876237.7	1227020.9	2127734.6	1797121.2	3263879.5	6703277.5	1585281.5
193.7	0.0	1028.6	0.0	0.0	1385659.0	2495261.5	2066962.6	734955.5	485487.1	1222919.5	1460468.3	1158189.8	2636974.0	1293854.8	391004.1	1671579.1	1071801.4	1910025.6	1598856.9	2903670.8	6056865.0	1425635.4
188.4	0.0	1076.9	0.0	0.0	1541118.1	2778409.3	2296459.5	820444.9	544530.8	1367471.6	1627595.9	1288598.5	2936631.7	1440115.4	434778.9	1858638.0	1204475.1	2143303.3	1787716.6	3227654.3	6765998.5	1606060.0
165.4	0.0	1125.1	0.0	0.0	1638799.2	2950264.3	2446073.3	882855.4	586321.7	1470532.5	1730489.6	1366591.8	3123082.4	1541414.4	462761.7	1981420.0	1224395.3	2338609.8	1945921.0	3542650.2	6966151.2	1746087.6
185.6	0.0	1052.5	0.0	0.0	1560321.0	2805629.1	2322589.3	831248.6	550876.1	1384414.6	1645443.3	1298096.4	2967827.9	1455239.2	438024.4	1875098.8	1213170.8	2148564.1	1796489.5	3262815.3	6767148.7	1605202.7
154.0	0.0	997.1	0.0	0.0	1469281.4	2647579.2	2151486.7	779909.0	530974.2	1312732.5	1563209.8	1228196.2	2813136.1	1366445.3	414846.7	1771192.8	1102008.7	2031681.3	1683508.2	3061918.3	6480181.5	1499258.6
155.0	0.0	878.6	0.0	0.0	1399448.2	2537008.9	2012048.7	754388.8	523239.3	1278805.9	1499803.8	1179109.5	2704357.6	1305901.0	399944.2	1709419.5	1021159.2	1990415.0	1630243.0	2940734.7	6013095.3	1427178.4
191.8	0.0	1004.5	0.0	0.0	1493091.2	2690209.4	2230537.7	799630.1	528534.3	1330077.9	1576172.2	1242414.1	2838747.6	1402025.6	422064.6	1805723.0	1176488.6	2109863.7	1758268.8	3161381.5	6436747.6	1575024.9
194.1	0.0	1071.4	0.0	0.0	1385787.4	2496456.9	2028335.8	734282.0	497308.4	1233193.7	1469443.5	1156979.7	2646128.4	1290201.1	390949.8	1675107.7	1051044.0	1900880.4	1594996.1	2897597.7	6121463.2	1418275.8
193.0	0.0	916.6	0.0	0.0	1438783.7	2606923.5	2079879.9	769992.4	532346.0	1304033.6	1543213.8	1210496.3	2777437.3	1341068.8	410890.9	1750339.4	1088014.6	2035251.3	1666462.1	3008398.4	6282007.4	1479874.1
196.6	0.0	1088.8	0.0	0.0	1622527.8	2936035.5	2413101.2	872070.6	576710.6	1451315.3	1720755.1	1362590.0	3105958.7	1522344.3	462694.5	1970894.7	1295561.0	2293074.2	1896390.9	3437915.3	6964601.0	1686679.5
141.9	0.0	1015.7	0.0	0.0	1473362.9	2663110.3	2144450.0	783830.2	540404.8	1326166.2	1575476.1	1239714.5	2836966.1	1371412.8	419694.6	1784140.4	1134983.0	2068606.6	1698345.4	3070630.4	6522624.1	1522607.1
204.0	0.0	990.4	0.0	0.0	1515711.4	2734402.6	2216288.5	815426.8	553451.9	1370510.5	1609171.6	1266848.4	2899168.4	1412307.8	428246.7	1838135.6	1171487.0	2104535.7	1746354.9	3142075.3	6564186.4	1538719.2
175.0	0.0	988.2	0.0	0.0	1446980.0	2615017.3	2116163.5	769677.2	523220.0	1295312.6	1539620.9	1209802.7	2769514.7	1343684.8	407934.5	1745771.0	1091149.4	1984699.9	1654525.0	2968110.2	6467414.1	1474381.9
194.1	0.0	1107.0	0.0	0.0	1593434.0	2883678.4	2314431.3	846208.2	584810.7	1433404.2	1701842.9	1336098.0	3059931.6	1481045.2	451101.5	1926154.3	1189906.7	2202696.4	1826817.4	3280664.8	7169202.2	1627461.0
203.8	0.0	983.4	0.0	0.0	1484800.6	2675145.2	2192635.8	794164.5	537505.4	1333762.0	1571295.0	1242761.3	2834822.4	1386321.6	419546.9	1788898.6	1127866.7	2059095.4	1711997.7	3065485.4	6536382.2	1525714.6
199.7	0.0	982.6	0.0	0.0	1402718.2	2534599.8	2045153.4	742588.9	507457.0	1252313.0	1493664.7	1171102.1	2682209.6	1302099.2	395329.9	1688545.9	1065517.2	1916537.3	1599348.2	2871259.5	6321187.7	1427770.4
239.7	0.0	1079.6	0.0	0.0	1626539.7	2946260.2	2373400.0	873290.2	596548.3	1472233.4	1735783.8	1366628.4	3127276.4	1514468.3	462453.3	1972057.4	1227897.9	2266561.6	1874380.4	3358315.5	7188152.5	1668856.4
224.2	0.0	1043.9	0.0	0.0	1534540.2	2757820.9	2302535.8	831031.2	550735.7	1383300.0	1612944.6	1274090.6	2907968.9	1442260.5	432523.0	1844209.8	1199569.5	2140565.1	1794088.6	3196327.6	6624629.6	1605474.6
187.7	0.0	1020.1	0.0	0.0	1610233.8	2887478.2	2407686.2	868360.1	573717.0	1443449.1	1688011.6	1337345.4	3048361.1	1508093.8	450812.4	1927737.7	1193152.9	2224479.5	1860619.1	3353969.6	6859758.1	1655615.6
181.4	0.0	1060.2	0.0	0.0	1613919.3	2898864.3	2395173.3	863384.5	573582.3	1438723.3	1699048.8	1331311.4	3051348.4	1509149.0	450271.7	1939487.3	1215439.9	2221551.8	1867947.5	3356731.4	6949472.0	1644877.3
197.5	0.0	912.4	0.0	0.0	1392102.9	2496282.6	2069528.8	742207.6	499138.7	1242778.5	1466428.5	1156461.8	2641688.9	1299670.4	390180.6	1664919.5	1068673.9	1913677.9	1602812.3	2871631.5	6136028.0	1441003.7
227.4	0.0	1054.2	0.0	0.0	1500380.8	2703312.9	2224456.2	798800.7	536677.8	1338004.8	1586827.4	1251933.6	2859240.2	1400811.1	423561.1	1807703.4	1138648.0	2080878.2	1730329.8	3129335.4	6556820.9	1538197.2
195.5	0.0	972.3	0.0	0.0	1479784.5	2652234.2	2220811.0	794301.3	524555.9	1320549.2	1549841.8	1222929.6	2792221.1	1386048.1	413688.0	1774040.7	1126441.4	2053337.8	1720697.3	3096581.7	6297120.0	1523164.0
161.3	0.0	996.9	0.0	0.0	1366685.2	2469477.4	1989833.7	723327.5	497311.1	1222900.8	1460259.7	1138991.4	2617962.0	1273863.9	388036.0	1653411.2	1055333.8	1918827.4	1581872.0	2868018.1	6035832.1	1409338.3
192.3	0.0	1023.2	0.0	0.0	1443400.6	2601100.9	2093741.1	763679.1	529588.5	1294795.5	1539595.4	1201160.9	2761077.9	1340381.3	406954.0	1739115.5	1086076.4	1968667.5	1640109.7	2968351.5	6491747.7	1456074.9
224.1	0.0	930.8	0.0	0.0	1446919.2	2612285.2	2103455.3	775216.4	536664.5	1313302.3	1543789.4	1213430.8	2780781.1	1348721.6	411307.7	1752656.0	1087086.6	2017114.2	1664819.7	2998477.9	6325998.0	1469599.3
156.2	0.0	880.2	0.0	0.0	1431091.1	2565361.2	2141388.7	779082.8	518815.0	1298259.9	1503594.7	1185176.0	2708429.3	1350887.0	405096.7	1723827.3	1197997.3	1998382.9	1685449.6	3000999.7	6076739.8	1480694.0
158.3	0.0	944.4	0.0	0.0	1485808.6	2679765.3	2221582.0	798143.0	526352.6	1326913.5	1562345.9	1241030.2	2821746.1	1389507.7	419663.1	1790028.8	1136697.1	2083833.6	1739979.5	3089367.0	6396087.4	1548227.7
200.8	0.0	1023.7	0.0	0.0	1602856.5	2901796.0	2346281.2	862235.5	586808.9	1450961.7	1710881.8	1335024.4	3067813.0	1500878.2	455320.1	1946390.2	1207461.0	2304270.5	1897062.6	3414420.5	6913267.1	1677173.9
175.9	0.0	940.9	0.0	0.0	1457068.7	2629309.4	2146222.1	785017.4	530740.9	1317144.9	1548229.7	1215999.8	2785382.1	1365885.6	413958.3	1766361.3	1100051.3	2081823.7	1721594.7	3085294.7	6228546.1	1522276.0
198.3	0.0	909.0	0.0	0.0	1427922.3	2572236.0	2104553.1	765706.4	518023.9	1285070.9	1511533.7	1189477.6	2721309.6	1336468.0	404253.1	1727040.8	1083796.3	2027426.5	1678768.8	3004230.3	6103058.0	1479965.5
151.9	0.0	993.4	0.0	0.0	1514724.5	2720654.4	2257893.9	809936.5	538719.6	1350504.5	1592565.1	1252277.0	2864027.8	1418115.8	425791.3	1823063.2	1151314.0	2120657.5	1767833.8	3182810.1	6422419.9	1554893.6
233.2	0.0	914.6	0.0	0.0	1308663.9	2348143.5	1961960.6	696724.5	459285.2	1157832.2	1370835.4	1082302.3	2468914.1	1226387.3	367035.4	1572485.0	1023719.0	1808678.9	1520900.9	2736776.9	5596088.4	1336929.5
200.7	0.0	923.5	0.0	0.0	1377639.2	2467757.4	2057217.8	736640.7	492806.4	1230377.2	1449236.7	1142294.7	2610585.4	1292472.9	387756.3	1656360.1	1066190.7	1937178.8	1612095.6	2894873.2	5897081.4	1431645.5
164.4	0.0	780.2	0.0	0.0	1363092.5	2457959.9	1997563.5	738825.1	500862.5	1239868.9	1448396.5	1145199.0	2617259.0	1276074.3	388683.4	1661868.6	1100715.3	1923936.0	1591966.9	2827667.0	5839330.6	1405474.8
192.0	0.0	1085.7	0.0	0.0	1501165.7	2707667.6	2228725.9	799059.1	533015.8	1334530.7	1587745.4	1254540.3	2862290.9	1402346.8	424607.6	1810670.4	1171048.8	2074011.0	1737781.3	3126169.0	6669718.4	1559498.6
211.5	0.0	1033.4	0.0	0.0	1558702.0	2815516.0	2291674.9	830345.0	560487.0	1393018.7	1659488.9	1303496.0	2983940.8	1453715.4	441616.8	1886610.0	1205993.0	2174029.0	1810858.4	3244954.1	6936529.4	1625686.2
174.1	0.0	992.8	0.0	0.0	1570242.8	2842767.1	2295057.8	840902.7	568812.6	1411847.6	1674226.8	1321957.5	3021371.7	1463577.9	447488.9	1910217.4	1225402.7	2198911.7	1809466.1	3281496.0	6778168.7	1596531.9
202.7	0.0	1158.6	0.0	0.0	1572076.6	2837836.4	2310572.0	838737.1	569997.8	1411172.5	1670162.8	1315431.7	3007538.1	1467183.6	445885.6	1900609.0	1193032.9	2192169.1	1813257.7	3287065.6	6925033.1	1612251.9
191.3	0.0	1020.2	0.0	0.0	1474874.8	2648652.6	2214779.5	791346.9	521736.7	1314912.3	1548360.7	1219989.5	2785084.2	1385062.3	414703.2	1773431.1	1121228.4	2103174.1	1749468.8	3145681.3	6246183.0	1547130.2

プロリン (mg)	セリン (mg)	ヒドロキシプロリン (mg)	アミノ酸合計 (mg)	アンモニア (mg)	脂肪酸総量 (g)	n-3系 多価不飽和 脂肪酸(g)	n-6系 多価不飽和 脂肪酸(g)	飽和脂肪酸														
								4:0 酪酸 (mg)	6:0 ヘキサン酸 (mg)	7:0 ヘプタン酸 (mg)	8:0 オクタン酸 (mg)	10:0 デカン酸 (mg)	12:0 ラウリン酸 (mg)	13:0 トリデカン酸 (mg)	14:0 ミリスチン酸 (mg)	15:0 ペンタ デカン酸 (mg)	15:0 ant ペンタ デカン酸 (mg)	16:0 パルミチン酸 (mg)	16:0 iso パルミチン酸 (mg)	17:0 ヘプタ デカン酸 (mg)	17:0 ant ヘプタ デカン酸 (mg)	18:0 ステアリン酸 (mg)
2028451.3	1714161.2	123988.4	33979600.1	690457.9	36254.0	1404.2	6732.9	135901.9	88994.2	803.3	57039.9	130865.2	224694.9	2409.9	829912.4	78135.8	20303.5	7078864.2	9710.8	103478.2	19303.7	2915132.5
2055895.9	1787031.1	142549.8	35514890.8	702867.0	40188.8	1660.1	7772.5	130013.3	84917.4	714.2	54198.2	126402.1	219075.7	2142.6	847192.4	76103.1	19402.5	7617629.1	9214.1	102815.2	18445.3	3198682.8
2245273.3	1977143.7	149558.2	39208234.4	787988.4	41057.1	1837.9	7907.7	119816.1	78495.5	690.3	51201.3	119973.5	220466.5	2070.9	866513.9	77737.5	17874.3	7771051.0	8546.2	109863.6	16984.3	3254561.9
2111575.6	1774502.6	120308.6	35168742.1	734023.3	33721.1	1444.1	6169.8	143424.3	93962.2	878.6	59770.5	135784.7	227554.0	2635.8	846548.6	77427.9	21427.2	6676339.3	10297.3	96664.5	20349.8	2754468.3
1972818.0	1692660.0	119468.9	33338266.2	686525.9	33625.0	1372.3	6206.5	132776.5	86732.0	746.9	54621.3	126446.9	209754.9	2240.7	794985.6	73008.9	19855.9	6641598.0	9417.4	94554.5	18922.1	2778012.6
2070125.2	1782433.1	132128.1	35628106.0	721438.1	37690.6	1718.8	7334.3	128924.0	84416.7	788.7	53358.2	123524.4	207942.6	2366.1	832965.5	76010.6	19271.7	7104357.4	9248.8	100820.2	18303.6	2960778.3
2142358.0	1821834.2	125876.8	35860597.0	738087.6	37955.6	1504.7	6888.5	149967.2	98026.1	918.2	60018.9	139734.8	215041.5	2754.6	906785.9	86740.9	22430.8	7414854.0	10753.7	114217.7	21357.8	3059546.0
1873894.9	1642076.8	114367.0	32258044.7	665348.7	33785.1	1446.1	6623.2	117328.7	76706.4	690.2	47991.5	112112.4	184921.5	2070.6	725022.9	66753.0	17560.1	6381265.5	8362.5	89096.1	16728.4	2673544.9
1837969.8	1536229.9	110776.6	30555648.2	630611.5	33178.4	1438.4	6378.9	138176.5	90268.8	802.8	56472.3	129687.4	208991.5	2408.4	781087.6	73171.1	20696.1	6307266.0	9823.4	90952.7	19692.1	2645533.9
1901611.8	1604471.8	111249.8	31569737.5	656546.2	33553.2	1376.3	6442.5	126716.1	83036.1	754.2	53938.6	122705.9	215301.0	2262.6	753450.8	69639.1	18988.9	6472777.3	9031.8	90116.3	18033.8	2700304.6
1816424.3	1504411.1	101888.1	29917226.2	636101.9	30184.6	1275.4	5870.0	118003.8	77160.6	682.6	48840.4	112285.8	187409.2	2047.8	686156.6	63826.4	17654.9	5789429.5	8395.1	81826.0	16808.2	2411697.3
2090730.9	1720904.3	125948.7	34339092.5	711541.3	36855.5	1420.3	6774.8	149286.0	97682.1	866.1	62257.9	142465.4	241270.6	2598.3	873580.0	81849.6	22290.6	7252535.8	10648.0	105363.4	21211.9	3007352.5
2114129.5	1784445.2	184220.8	35526465.2	722500.1	70214.3	1515.7	6573.8	199107.2	141993.2	34758.4	102982.3	155284.3	259395.3	36709.2	927388.1	88215.7	58470.9	7188801.1	45598.5	107477.6	57223.1	2944431.5
1915814.9	1590838.5	117877.6	31779531.9	649841.5	33828.1	1285.4	6008.9	149648.8	97687.5	819.4	61749.2	140895.5	232244.0	2458.2	849180.8	80275.8	22328.9	6771230.1	10604.0	101356.9	21249.1	2804604.2
2129255.1	1766744.7	134266.8	35457640.2	724818.9	37957.1	1447.0	6755.1	154353.6	100908.9	838.1	65404.6	148692.3	261388.4	2514.3	924988.7	86249.3	23016.8	7580018.6	10933.3	112928.9	21891.7	3159477.2
2162555.5	1899992.7	137947.5	37601527.1	769410.7	37952.9	1704.6	7372.4	127645.5	83691.7	769.8	54568.3	125483.7	225580.7	2309.4	832165.8	74593.6	19135.3	7244197.7	9110.2	101337.2	18161.6	3037382.8
2134955.6	1799922.1	128580.9	35669140.1	737399.6	34715.7	1464.3	5903.1	143641.1	94198.8	882.8	60416.5	138666.8	237701.7	2648.4	895194.4	84155.7	21485.0	7086005.3	10304.9	115567.7	20444.4	2937526.0
2043995.4	1713198.1	115385.9	33666755.9	700744.9	34370.0	1334.2	6136.6	137579.2	90258.1	833.5	58493.6	132994.2	232488.1	2500.5	834995.5	79411.7	20612.1	6887940.2	9833.4	105653.0	19639.7	2828061.8
1873251.0	1662826.0	103345.0	31976774.2	655893.2	32669.0	1230.0	5996.0	119739.2	78656.5	750.0	51676.7	116389.2	207020.9	2250.0	737955.8	70503.8	17914.9	6430042.8	8608.9	95889.6	16988.9	2615168.9
2004600.5	1708959.5	132504.0	34332360.1	692941.2	37125.0	1547.2	7076.4	126134.1	82511.1	738.9	52241.8	122258.8	208294.7	2216.7	810220.4	74878.3	18856.2	7130053.9	8996.2	103297.3	17943.6	2995140.0
1940369.7	1613178.8	109360.5	31800215.3	672228.3	33878.5	1467.1	6633.6	136368.9	89241.8	794.5	57114.2	129324.5	218846.3	2383.5	772523.4	72039.7	20405.9	6391879.0	9708.6	89007.2	19409.0	2649978.7
1967480.8	1688155.8	112105.8	33043504.6	681740.7	34535.4	1361.6	6564.6	128088.3	84016.5	788.1	54217.6	123953.8	215204.5	2364.3	778995.2	72780.2	19146.1	6701076.1	9196.6	95451.6	18189.1	2750507.6
2186091.4	1863026.3	129351.5	37266643.6	759257.2	36194.8	1472.0	6567.0	157663.7	103217.1	948.0	65393.0	149924.0	249934.6	2844.0	904550.9	84316.9	23554.4	7266024.7	11291.7	107014.5	22392.9	3016579.8
2048567.1	1718991.2	115496.7	33931498.0	701943.8	34756.2	1350.4	6312.5	140263.9	91762.5	808.8	58348.4	134304.5	227135.5	2426.4	840007.8	79492.0	20900.8	6906919.1	10019.1	102094.7	19889.6	2813210.3
2062944.6	1776997.5	115636.8	34622331.5	700086.6	36248.8	1437.3	6582.5	144126.0	94380.3	872.7	59515.2	136062.2	223245.2	2618.1	858780.4	83531.1	21529.4	7105620.0	10334.0	107055.1	20502.5	2853531.4
2047702.0	1678520.6	117627.7	33221293.2	683091.8	36281.6	1338.7	6440.3	148833.8	97616.9	886.0	63649.3	143295.3	252765.5	2658.0	886981.8	85263.1	22229.2	7205809.1	10648.4	109704.9	21141.1	2917735.7
2265992.5	1859033.1	123030.9	36675842.7	765250.1	39068.0	1418.0	6904.7	159958.9	104809.9	926.3	68328.8	153408.9	269532.3	2778.9	955333.6	93317.3	23843.4	7753645.4	11427.2	120266.7	22650.2	3102195.4
2057898.6	1729817.5	125301.0	34049912.3	684951.5	36912.1	1266.6	6311.9	141409.0	92625.4	843.8	59237.7	136329.1	232756.3	2531.4	887722.4	86327.0	21072.1	7488883.4	10145.4	116834.6	20021.7	3033298.7
2007933.4	1625174.3	110178.3	32245616.7	671101.8	32852.6	1153.2	5560.1	143749.9	94202.2	851.6	60667.2	137780.3	236753.3	2554.8	843404.7	82189.7	21460.8	6723541.8	10283.2	105286.7	20427.5	2697673.7
2270797.7	1898469.3	130803.2	37360387.7	762883.0	40816.8	1492.8	7190.5	162844.5	106660.1	976.6	68035.3	155302.2	262260.3	2929.8	984436.9	95578.4	24298.4	8119058.0	11674.4	124952.5	23096.6	3279741.5
2070420.6	1774243.2	134123.2	35152583.2	690682.2	37546.8	1363.1	6539.6	124563.1	81888.4	786.1	53778.9	123767.8	225630.5	2358.3	850455.8	81719.4	18581.7	7529793.0	8994.7	116171.7	17642.7	3064653.7
2156722.8	1877882.0	127050.6	36568415.4	726840.3	41027.9	1716.1	7903.2	144526.0	94767.6	920.5	60068.9	138531.4	232041.0	2761.5	889477.1	86382.3	21571.8	7755645.0	10442.7	114357.6	20454.9	3167346.3
2180655.1	1868623.9	128432.5	36733906.5	741286.0	38375.8	1558.5	7035.8	145469.4	95496.8	935.3	60888.3	140303.0	238389.6	2805.9	890974.9	85483.9	21736.4	7543184.6	10513.4	112577.7	20660.1	3069089.5
1942970.9	1612754.4	121649.8	31914703.3	641490.5	35825.3	1357.6	6551.8	123503.6	80929.7	733.6	52298.0	120774.5	212378.8	2200.8	796433.0	75813.0	18430.9	7014002.2	8841.0	104350.2	17507.4	2875921.3
2069285.8	1735401.4	124886.1	34353638.5	693864.1	39346.7	1546.0	7279.7	146848.3	96244.1	900.4	61445.2	139884.7	235964.2	2701.2	909896.0	88530.2	21958.9	7602815.3	10534.3	117050.5	20874.1	3089531.7
1972627.7	1707275.7	125054.8	33660386.1	655905.0	37513.3	1461.8	6875.4	124396.0	81810.0	765.9	55251.7	123195.2	232054.1	2297.7	831933.9	79928.0	18614.0	7285959.2	8920.6	110978.9	17626.4	2950149.7
1893273.2	1583167.8	107846.3	31432468.3	648150.6	32491.7	1220.4	5756.2	125870.7	82476.1	767.8	52938.3	121005.9	205629.1	2303.4	782424.7	75482.0	18783.6	6489360.9	9042.1	101962.5	17801.9	2624207.7
2064823.6	1691388.5	108256.7	33136978.3	698742.1	34092.1	1318.6	6215.8	142552.7	93373.0	892.3	58508.5	134357.9	216888.2	2676.9	825411.4	80866.0	21298.7	6745400.0	10264.7	102900.5	20239.7	2706131.0
1995613.2	1708045.8	112694.6	33139428.9	672938.5	37126.6	1437.2	7053.1	129929.9	85282.0	827.8	54812.4	125351.0	215473.0	2483.4	819355.5	79617.5	19428.5	7132792.9	9370.1	105513.9	18436.7	2872355.6
1879019.6	1661237.4	113417.9	32702173.3	641169.2	34572.9	1440.0	6571.6	98415.0	64665.9	584.4	43634.7	99610.3	192055.2	1753.2	705351.8	67951.7	14694.3	6585328.3	7040.2	97137.6	13929.0	2641974.3
2007505.9	1701204.0	135279.8	33993678.8	657075.6	38405.0	1354.2	6665.9	137657.8	90024.5	778.1	57514.5	132915.2	227575.9	2334.3	885335.1	84464.6	20569.2	7718153.2	9778.2	117185.3	19592.5	3153595.4
2134295.6	1852017.7	128802.9	36822067.4	739084.9	41664.4	1796.9	8370.6	121089.0	79496.5	738.0	52561.7	119649.3	220080.3	2214.0	832377.5	79790.0	18120.8	7650107.8	8670.9	111980.6	17177.6	3094703.9
1929778.4	1692614.8	120556.5	33376586.8	658724.5	38048.6	1567.6	7553.5	113094.5	74160.6	683.3	48136.6	110922.0	197109.8	2049.9	755552.4	71981.2	16926.5	7104651.2	8088.6	101427.6	16083.6	2898920.7
1894442.0	1659777.4	119460.3	32637619.9	636299.2	38648.9	1517.8	7508.7	113799.4	74648.0	672.4	49122.1	112218.3	204726.0	2017.2	767458.0	73963.5	17041.2	7235171.5	8110.1	104608.5	16202.7	2943272.3
2003603.3	1748283.2	124372.4	34450279.8	671799.9	39433.1	1595.3	7466.5	128428.1	84288.6	792.2	54873.6	124089.2	218448.5	2376.6	843875.1	82624.8	19210.4	7473653.4	9216.9	113630.6	18243.1	3011683.9
1749571.0	1502881.3	110766.7	29800341.2	583412.0	33863.7	1308.4	6341.4	123565.6	81012.2	760.6	52244.4	118476.0	203649.3	2281.8	749633.5	72261.5	18452.7	6539674.2	8880.6	96133.8	17486.9	2670939.4
1828102.2	1595939.3	120488.0	31486563.6	617906.3	38412.1	1603.4	7904.1	105456.7	69155.1	638.4	44791.0	104545.4	188234.5	1915.2	714101.4	67504.0	15790.2	7028433.9	7541.0	95872.3	15016.2	2911878.1
1815967.9	1587985.9	111887.7	31227718.6	625724.8	36286.0	1375.8	7202.4	98408.2	64487.7	539.2	43529.8	100116.4	192902.5	1617.6	700776.2	65043.8	14835.8	6830678.5	6893.1	95600.8	14116.7	2857311.6
2101798.8	1724163.1	132557.6	34611060.7	713703.5	35621.0	1309.0	6147.5	149266.8	97503.6	841.9	61403.4	142602.3	236348.2	2525.7	890829.9	83174.2	22282.3	7296994.8	10611.0	109690.9	21209.1	3038746.8
2180110.1	1797678.9	134135.0	35975393.9	747529.9	38786.8	1557.5	7234.8	146301.0	95453.1	796.1	59496.8	140006.3	230409.5	2388.3	884424.7	82595.3	21872.6	7537117.5	10337.8	108616.3	20899.2	3137695.4
2142324.3	1828259.5	119014.5	35948752.8	739505.5	36156.5	1460.9	6833.2	158776.4	103885.6	956.5	65479.2	149092.0	242414.9	2869.5	878645.5	82730.5	23738.8	7109011.4	11364.2	102253.1	22546.3	2945738.7
2173848.4	1828669.8	124121.9	36092088.9	740415.3	37522.1	1390.9	6573.0	153937.8	101028.2	956.0	65376.0	147756.9	255564.9	2868.0	922210.8	88979.4	22986.1	7518746.0	11079.2	115288.1	21830.3	3032933.3
1919804.4	1684861.2	123170.4	33669008.8	651970.0	35131.3	1401.5	6277.3	110215.1	72151.7	661.3	45696.3	106580.8	179527.3	1983.9	774603.7	76095.7	16492.1	6903930.2	7877.6	110043.8	15694.7	2772987.1

			一価不飽和脂肪酸																			
20:0 アラキジン酸 (mg)	22:0 ベヘン酸 (mg)	24:0 リグノセリン酸 (mg)	10:1 デセン酸 (mg)	14:1 ミリストレイン酸 (mg)	15:1 ペンタデセン酸 (mg)	16:1 パルミトレイン酸 (mg)	17:1 ヘプタデセン酸 (mg)	18:1 計 (mg)	18:1 *n*−9 オレイン酸 (mg)	18:1 *n*−7 シス-バクセン酸 (mg)	20:1 イコセン酸 (mg)	22:1 ドコセン酸 (mg)	24:1 テトラコセン酸 (mg)	16:2 ヘキサデカジエン酸 (mg)	16:3 ヘキサデカトリエン酸 (mg)	16:4 ヘキサデカテトラエン酸 (mg)	18:2 n−6 リノール酸 (mg)	18:3 *n*−3 α-リノレン酸 (mg)	18:3 *n*−6 γ-リノレン酸 (mg)	18:4 *n*−3 オクタデカテトラエン酸 (mg)	20:2 *n*−6 イコサジエン酸 (mg)	20:3 *n*−3 イコサトリエン酸 (mg)
104547.5	49767.5	22924.8	11535.0	100990.6	26.8	796620.0	84335.2	14666608.1	5942992.9	363555.8	310776.6	115140.8	22091.3	3090.6	2866.9	3257.4	6474936.0	1044269.4	1341.9	30189.9	47514.5	528.9
119331.7	57010.1	26361.2	11011.0	85428.2	53.8	822733.5	81206.2	16220382.5	6835971.7	439113.7	412363.0	196364.0	25064.1	4098.8	3196.8	4440.6	7484373.7	1226880.0	1621.9	42917.7	57788.1	992.9
123016.4	58987.7	26383.3	10211.7	87545.4	62.8	850011.0	85624.0	16400216.3	7137117.8	452121.9	458265.3	243876.8	30290.8	6003.4	4876.1	6326.0	7597231.2	1233506.9	2606.8	59924.9	58785.0	1143.6
96468.3	45072.0	20347.8	12112.6	81827.7	56.9	713501.0	72120.4	13094136.3	5692976.8	359583.0	380462.3	215925.1	24758.6	4575.1	3592.6	4777.3	5909240.3	930392.0	2110.2	53764.3	47159.1	1016.1
96173.7	44628.5	20126.1	11243.6	79701.9	44.1	706635.3	72758.1	13280830.6	5819692.6	370054.3	341203.7	166642.7	21768.2	3577.1	2993.9	3649.6	5941595.1	952367.0	1873.5	44019.1	48631.5	814.6
113464.8	55331.9	24848.4	10888.3	81745.8	58.5	776133.5	76957.2	14920353.4	5978806.8	385596.3	415882.3	219538.1	28599.7	5342.2	4418.9	5541.6	7059179.2	1170190.1	2331.6	55189.9	51561.7	1025.0
107048.1	50953.2	23163.1	12661.5	122883.8	41.7	864173.4	96726.8	15272880.9	5854268.9	370882.7	366177.0	167187.0	23934.1	3671.6	3100.1	3751.2	6618268.4	1070968.1	1707.0	41085.4	48706.4	770.2
102170.4	49632.9	22591.7	9948.5	73125.0	33.4	666416.2	68896.3	13547693.3	5839149.1	369781.3	331437.8	146602.7	22452.4	3259.1	2643.5	3186.5	6371064.8	1052572.9	1610.5	37278.8	46194.2	668.5
100007.5	48741.3	21959.2	11701.1	78449.5	33.3	656099.6	69445.3	13153032.3	5303821.2	338485.7	325711.4	148110.2	23315.9	3237.0	2684.2	3129.6	6142434.9	1032898.6	1416.4	36267.8	43785.5	635.2
99924.5	47826.7	21551.9	10788.9	74050.8	28.7	673555.8	68471.0	13396164.1	5705463.2	355043.2	312283.1	130035.4	21566.7	2961.0	2528.8	2819.6	6191108.4	1009244.5	1492.3	33798.2	45918.6	556.7
91623.1	44253.1	20159.9	10015.9	68522.9	20.9	593250.1	62025.3	11973531.3	5038932.7	317388.7	284301.6	118518.2	20078.2	2835.6	2285.1	2551.6	5653610.9	924257.4	1239.0	28299.0	40557.0	459.1
106127.2	50087.1	23198.7	12630.7	100237.5	24.5	799123.6	84179.5	14806747.2	6018841.8	375184.8	324275.4	123944.0	22604.2	2891.4	2548.0	2725.5	6513672.5	1045626.7	1378.2	31784.2	49828.7	491.7
104694.9	49525.0	23029.4	13953.7	102518.7	36.1	792302.6	83918.8	14400131.1	5828753.4	360178.1	351316.9	162480.9	26094.9	4241.6	3653.1	4120.5	6307818.4	1025739.1	1927.4	42209.3	47516.6	672.5
95288.8	43989.1	20397.4	12632.7	100247.0	22.7	757260.0	81823.4	13514412.0	5469574.1	342903.0	297427.7	115746.6	20831.0	2802.5	2567.6	2701.7	5768140.7	923229.4	1285.9	29892.6	45825.6	459.8
107870.2	49407.0	23119.1	13036.8	107189.4	26.4	843376.5	90875.4	15236001.9	6365135.2	397260.8	338015.9	131535.5	23911.3	3202.4	2815.4	2983.8	6486799.3	1034581.4	1458.9	33751.2	52367.2	515.3
114845.1	54758.6	24826.3	10870.9	72576.9	39.8	757428.4	74723.9	15001312.9	6656222.3	422549.1	393693.6	188057.9	28434.8	5064.2	4166.1	5188.1	7085111.5	1153575.2	1917.4	45307.5	54335.2	790.5
95811.4	42237.1	19528.4	12230.8	97627.4	23.2	808516.8	89350.4	13676044.4	6138306.2	376287.3	333687.0	142602.9	30310.0	3521.2	3142.4	3327.0	5630752.9	877910.9	1548.9	43014.3	49335.3	468.7
96555.3	44728.9	20615.4	11735.2	100118.4	22.1	777946.0	84365.4	13765601.6	5819207.4	348261.8	294837.1	110264.3	23463.7	3100.1	2914.0	3161.2	5881147.5	921813.9	1392.7	32497.4	45356.1	462.0
92448.0	43556.4	20876.5	10209.1	97137.7	13.4	722769.2	79595.8	13337086.4	5651398.1	334166.7	259168.6	78906.4	19232.6	2123.0	2104.0	2115.2	5750958.3	922123.6	1118.3	24162.6	41358.6	337.6
110177.6	52664.5	24015.6	10694.2	90530.4	24.9	786317.9	82030.6	14935390.4	6019819.2	388653.9	347959.6	140450.9	24841.4	3337.8	2805.8	2970.7	6809242.4	1115480.7	1640.8	36647.6	51662.6	535.9
103478.1	50821.1	23058.3	11564.3	73361.9	30.0	649782.6	66871.4	13489613.4	5687680.0	351600.5	325938.0	141821.8	23545.8	3300.9	2740.3	3175.2	6387603.3	1067480.2	1559.1	36111.8	44496.0	604.9
101152.7	48594.8	22706.8	10898.5	88426.0	33.1	720759.4	75822.2	13884545.7	5880939.1	358846.1	314160.5	131169.9	21417.4	2901.7	2479.5	2908.8	6313654.2	1010587.5	1367.3	32848.6	45486.8	599.4
104533.8	48272.7	22838.9	13345.2	93144.1	24.9	766151.0	81103.2	14217308.7	6155139.4	389679.8	335865.9	141800.5	24265.0	3633.9	3032.9	3152.8	6298129.8	1011540.0	1775.1	37943.6	50855.4	496.5
98590.1	46338.7	21684.8	11892.3	100455.8	38.7	769330.6	82005.7	13886153.0	5793002.3	353204.9	326083.9	148151.0	22425.9	3324.8	2884.4	3332.1	6061246.4	958915.6	1446.0	35909.4	45386.0	654.1
102160.0	48961.9	23155.4	12231.8	113370.5	34.6	825964.3	88695.2	14651795.1	5803507.0	347408.4	333282.6	146089.5	24164.0	3541.2	2978.6	3492.6	6317427.5	1017594.0	1512.5	36103.0	44464.5	606.0
101508.9	47925.7	22681.9	12641.8	120767.4	22.2	842471.4	92336.9	14744137.0	5700281.4	337115.6	300760.6	108678.8	21985.5	2855.7	2748.6	2954.4	6194242.1	984544.4	1169.1	27693.5	44392.3	452.3
107841.4	51372.7	24525.8	13547.2	143544.9	20.4	930423.2	105136.6	15977246.9	5966141.7	349234.2	309388.1	102957.5	22344.9	2888.1	2926.3	2872.6	6643793.9	1064544.1	1228.5	27169.0	45091.3	448.0
98016.5	45060.9	21217.6	12001.8	135854.7	18.9	926567.6	102302.6	15148194.3	5857852.7	352624.6	294815.6	97662.6	20136.4	2731.5	2639.6	2626.4	6050866.7	930405.3	1158.6	25413.2	46971.4	399.0
88613.9	40490.6	19367.5	12226.9	119445.8	15.1	801925.1	89212.6	13324655.9	5330937.7	312543.4	259495.8	85543.9	19043.5	2757.7	2768.8	2689.2	5330976.5	825390.8	1267.1	25865.4	40621.8	341.8
112636.5	53079.2	25279.9	13790.3	144418.6	25.6	971354.4	107950.5	16683487.9	6361396.0	382197.6	335048.8	118854.7	23179.6	3360.9	3230.0	3347.2	6910047.9	1118666.3	1412.1	30413.6	49353.4	491.1
101480.4	46784.5	21980.6	10608.7	124682.5	17.1	932637.2	99930.2	15465371.3	6241188.7	375344.8	307598.4	101947.2	22800.3	3225.1	2993.6	2959.6	6264371.2	969914.3	1457.9	29720.2	49706.7	383.6
121849.9	60427.0	27341.8	12280.0	111844.2	22.9	882962.2	92479.8	16720869.9	6516774.1	393875.8	345032.6	118125.8	27117.6	4140.4	3977.6	4317.4	7601105.1	1262446.1	1752.4	34787.5	49641.7	506.6
110856.5	52726.8	24490.9	12402.7	104650.0	25.1	861117.5	88684.1	15472925.6	6396159.8	381888.6	328469.1	120445.6	26994.8	4444.3	4230.6	4375.7	6747416.1	1088541.1	1963.4	38113.5	49638.4	528.5
101061.4	47806.9	21628.3	10522.0	104264.6	22.4	829939.4	86773.5	14658290.6	5847450.0	355106.1	302093.4	104891.5	22352.3	2842.4	2563.1	2828.4	6294179.5	999849.6	1136.8	27935.6	46910.6	444.9
112285.3	55038.5	25100.8	12437.0	131067.0	30.8	916554.2	100223.5	16006624.6	5628947.4	342824.0	336766.1	128238.7	24740.0	3798.4	3480.2	3842.5	7014645.9	1142011.7	1536.5	33912.9	46505.6	566.1
105750.7	50853.2	23170.8	10610.7	118051.8	23.7	892926.6	95009.6	15379719.5	5832476.5	344749.6	315012.9	112234.0	24881.7	3715.2	3368.9	3721.8	6609033.1	1050414.6	1443.7	31170.8	46347.1	491.5
90053.4	41999.1	19522.2	10707.5	111682.3	21.8	779086.7	86695.3	13157637.0	4986625.3	302804.1	277244.9	103485.9	20423.3	2509.8	2415.0	2429.5	5530407.6	874806.9	1236.5	30175.5	40437.4	411.8
95857.3	46046.3	21349.8	12101.9	109140.6	22.9	783520.2	84025.6	13716676.2	5192594.5	312282.2	284583.6	104537.8	21747.3	3106.4	2898.8	2965.3	5969647.4	949138.8	1332.1	30382.9	40687.9	450.2
106654.4	52900.6	24398.3	11055.5	116334.6	33.9	839413.1	89555.7	15142135.8	5519336.0	330098.9	318321.7	126463.7	22587.1	3294.0	2977.1	3318.8	6792006.6	1089175.5	1369.8	31307.6	43263.6	591.4
100640.5	49108.4	22546.9	8435.0	95555.6	20.9	790631.5	80980.0	14222645.0	5538911.3	328104.7	301083.0	112742.9	24563.3	3225.9	2994.9	2947.6	6313588.6	1028312.6	1527.7	32540.3	42008.3	411.1
104249.2	47311.7	22199.7	11669.0	126184.5	17.9	958473.6	101532.2	15824013.3	6206601.9	380417.3	305545.4	91266.0	21996.9	2972.1	2757.8	2970.5	6396152.9	1003794.9	1316.3	27300.3	51614.6	370.4
128010.5	64987.5	30053.8	10320.1	115392.2	19.8	896790.7	94935.2	17129894.8	5963888.7	360034.6	345143.2	110567.6	28679.2	4078.8	3842.3	4036.0	8097660.9	1364478.9	1731.0	33924.2	47049.5	443.1
114477.9	57087.8	26278.5	9634.5	98579.0	14.6	822713.4	84803.0	15654002.8	5754031.8	348364.8	303579.3	87183.1	24319.2	3098.0	2865.1	3041.4	7288388.9	1209965.6	1429.3	27500.0	46407.1	323.4
114164.1	56864.0	26127.0	9699.9	112052.7	14.1	863785.0	90827.4	16110857.3	5876799.5	351758.7	299601.3	80848.1	22570.8	2616.6	2495.9	2564.4	7245674.3	1214485.3	1178.9	23167.5	45666.8	295.9
113796.5	56664.3	25819.8	10894.3	126097.9	23.7	923958.7	98650.7	16247755.5	5718287.9	341197.8	325743.8	111204.3	26129.3	3681.9	3343.2	3613.5	7193103.8	1189162.1	1645.9	33411.5	45522.2	467.3
97987.6	47622.4	21687.0	10464.9	98679.3	13.7	768156.5	79660.8	13867625.2	5217304.7	318030.0	266230.1	78548.5	20581.3	2643.7	2547.9	2486.4	6101827.2	1003373.6	1100.6	21226.4	42364.5	291.8
118729.9	60525.0	26935.9	8989.8	85240.9	13.9	789255.1	77546.9	15835319.3	5829526.7	358694.5	305504.1	84344.8	23927.5	2835.5	2642.6	2644.8	7634686.4	1282997.4	1201.1	22805.7	47698.6	310.7
108060.6	52910.7	24208.9	8458.2	95007.2	20.1	763120.9	82046.5	14956989.4	5465142.6	344231.5	301280.8	96968.6	18988.6	2126.8	1986.2	1873.3	6947045.1	1114307.0	1010.7	21992.4	47190.4	411.4
97866.8	43252.1	20352.6	12644.0	102872.1	25.3	825534.9	87683.5	14213457.6	5973394.0	380119.9	318470.3	125408.0	22068.0	3069.9	2798.2	2906.8	5884720.5	909116.8	1463.4	32683.7	51985.6	505.2
114577.7	53919.4	25018.3	12444.1	96851.1	29.7	818371.1	86036.3	15630095.0	6603492.0	414500.4	356057.6	141835.6	25175.7	3458.6	3031.5	3289.1	6959836.3	1138928.1	1656.8	37358.9	53342.8	591.1
106495.8	50748.4	23620.3	13431.6	89312.3	28.7	728548.1	77207.6	14240966.2	5987269.3	375277.2	329183.3	136138.4	23093.0	3404.5	2849.2	3065.2	6563312.5	1056976.6	1575.5	34710.1	48478.7	551.3
103590.1	48556.7	22928.2	13077.2	126808.1	24.8	887010.7	96869.7	15211305.3	6049776.8	358508.2	317854.2	120358.7	22634.3	3341.6	3206.4	3301.3	6310434.1	1002323.7	1437.9	31406.0	46572.9	495.0
96772.5	45811.7	21004.0	9365.4	123498.7	14.9	903058.8	97313.8	14444370.6	5073305.9	310279.7	290781.7	95599.1	23847.7	4089.0	3821.7	3944.8	6025514.7	974946.9	1714.2	31225.1	43242.5	355.9

多価不飽和脂肪酸										未同定物質 (mg)	利用可能炭水化物									糖アルコール	
20:3 *n*−6 イコサトリエン酸 (mg)	20:4 *n*−3 イコサテトラエン酸 (mg)	20:4 *n*−6 アラキドン酸 (mg)	20:5 *n*−3 イコサペンタエン酸 (mg)	21:5 *n*−3 ヘンイコサペンタエン酸(mg)	22:2 ドコサジエン酸 (mg)	22:4 *n*−6 ドコサテトラエン酸 (mg)	22:5 *n*−3 ドコサペンタエン酸 (mg)	22:5 *n*−6 ドコサペンタエン酸 (mg)	22:6 *n*−3 ドコサヘキサエン酸 (mg)		でん粉 (g)	ぶどう糖 (g)	果糖 (g)	ガラクトース (g)	しょ糖 (g)	麦芽糖 (g)	乳糖 (g)	トレハロース (g)	利用可能炭水化物計 (g)	ソルビトール (g)	マンニトール (g)
22273.1	11148.1	101579.3	97110.4	2934.2	199.6	13341.9	35756.1	16253.0	181629.3	186755.0	85688.2	4553.3	4799.6	38.5	12174.9	719.6	3759.4	59.9	111807.5	158.4	225.6
23719.0	14666.0	109733.0	115193.5	4476.5	353.2	16276.8	40169.5	18179.9	214081.8	237868.6	90994.7	4521.0	4563.7	40.5	11953.4	660.2	3436.5	57.5	116253.7	112.6	244.4
24418.8	19855.7	121708.6	166031.1	6191.1	463.5	17052.0	53616.9	21126.2	296799.1	270504.2	95711.6	5295.0	6483.4	41.9	13893.5	733.9	3267.8	66.6	125534.7	243.4	318.0
21387.8	17434.1	103018.9	136212.8	4902.2	399.2	13626.8	45323.9	17529.7	253317.1	229853.3	88194.7	4814.3	5735.2	35.9	10886.5	746.9	4094.3	69.1	114597.3	233.8	266.2
21192.6	13246.3	104761.0	111913.0	4027.5	264.1	14256.9	37944.0	17775.8	206924.5	213674.1	77361.4	4795.8	5080.8	32.6	11500.5	687.0	3472.7	55.0	102993.1	202.0	297.2
22215.2	17884.1	106477.5	151180.9	5502.7	387.5	14540.2	49014.1	17415.2	267285.3	223998.1	87471.9	5038.8	5564.2	38.8	13914.1	662.2	3695.7	59.1	116476.3	235.7	263.2
23529.1	14097.9	105707.5	115455.2	3975.3	270.9	13911.6	39854.5	17841.3	216675.8	213657.7	93172.8	4717.7	5473.9	52.8	13429.9	721.8	4137.0	57.7	121785.4	312.7	315.9
19943.9	12552.3	98608.6	103274.2	3613.9	254.3	13621.0	36403.2	17039.5	198170.8	203522.1	86211.2	4472.6	4921.9	35.9	10921.8	625.7	3178.4	43.8	110417.9	258.6	271.9
19472.6	13036.0	90943.8	106109.6	3541.4	291.1	12505.7	38029.2	15136.3	205495.3	196468.2	67982.9	4557.3	4882.0	37.6	12427.5	646.7	3831.2	48.2	94426.2	193.4	185.6
20154.6	11368.3	98519.3	96022.9	3283.9	204.4	13320.4	34724.7	16612.2	185345.7	195919.8	75180.2	4387.3	4404.2	39.5	10770.8	712.2	3690.2	53.4	99240.9	156.4	222.6
17566.3	11106.1	82236.0	90862.6	3191.0	199.1	11643.1	33199.9	14049.7	181498.5	180300.7	83420.4	4281.6	4667.0	36.0	10323.0	696.1	3197.3	49.8	106676.5	187.9	201.7
22638.2	11561.5	100047.8	97840.9	3181.3	196.5	13846.0	36640.7	15601.1	190727.7	194337.0	84953.5	4532.3	4639.2	42.1	9872.3	774.6	4004.0	58.6	108889.3	144.5	235.2
23021.2	14951.8	106931.5	132230.2	4486.1	244.3	13696.3	44997.4	17981.1	247812.9	214986.5	80135.8	5171.6	5368.3	43.0	13513.4	701.9	4642.5	63.0	109657.6	164.5	281.7
21269.1	11187.4	93159.9	95397.5	3041.8	195.5	12739.2	35451.4	14562.0	184745.9	185454.2	74110.4	4484.4	4677.2	34.3	10054.6	668.0	3871.9	56.9	97970.3	141.8	211.1
23491.6	12376.8	103435.3	109080.4	3548.8	192.2	14637.4	40267.0	16197.4	210867.8	203511.8	86392.0	4767.6	4938.3	37.0	10809.4	756.0	4002.5	63.8	111778.7	136.6	243.9
22601.3	17943.4	112127.1	151059.0	5091.1	385.7	15692.4	51598.4	19125.5	278184.6	237590.6	95736.4	5422.5	5878.4	41.6	13857.8	667.2	3786.8	52.7	125465.9	304.5	262.1
22935.7	17498.3	112897.3	159925.1	3665.0	241.0	14119.0	57380.8	17785.8	302316.1	219775.0	83460.5	4635.6	5000.7	38.3	10578.3	789.6	4045.3	62.7	108623.8	195.7	301.6
21931.2	12274.5	104827.7	114003.7	3055.7	160.4	13247.7	41295.6	16912.6	207604.5	194385.3	84085.9	4527.1	4687.2	35.6	9702.6	786.2	3819.8	60.2	107707.5	162.8	245.7
20695.1	9071.4	102138.1	82776.8	2156.6	142.9	12563.6	31474.2	17515.8	157945.6	180572.4	95706.3	3897.6	3928.5	36.0	10027.4	629.5	3602.5	50.3	117886.7	112.5	224.7
22196.6	13093.3	100836.3	112374.4	3796.5	244.9	14558.9	40929.9	16150.4	221306.1	199020.0	87528.6	4637.8	4721.1	38.1	10228.4	671.7	3440.8	58.5	111326.2	137.5	228.1
19654.8	12793.9	95995.9	105943.6	3541.7	236.7	12835.8	37395.7	16194.4	201399.1	202936.4	77016.2	4995.9	5954.2	37.2	13337.8	706.5	3771.0	51.3	105897.8	259.1	226.2
21063.6	11036.7	99444.6	91814.9	3130.5	186.7	13280.5	33822.3	16869.3	176805.1	196113.9	95633.3	4174.0	4223.4	35.1	11594.0	679.6	3680.2	64.6	120095.2	118.6	193.4
22772.5	13694.0	104505.5	120484.0	4116.2	219.3	14650.7	42890.6	17431.2	238977.7	209870.1	100695.5	4911.5	5159.8	34.0	13526.3	673.0	4393.3	61.7	129467.4	156.6	274.2
21828.2	12256.5	99555.1	103583.0	3604.9	194.7	13196.0	36955.2	16881.5	197182.0	204050.4	91683.3	4674.6	4883.0	29.7	12878.2	756.1	3683.9	65.9	118666.5	125.7	190.3
23172.9	13136.8	109272.6	110447.1	3740.7	200.0	13391.7	39490.6	19527.5	214485.3	215738.1	93041.0	4427.4	4549.2	45.7	12258.4	726.8	3911.7	61.4	119028.8	166.4	183.4
22677.5	11135.9	98162.0	94785.8	2849.0	174.0	12548.2	35909.1	15640.7	180098.2	187428.6	85638.7	4251.7	4369.4	38.6	11956.3	787.3	4103.9	65.4	111227.9	144.8	200.3
24404.6	10758.9	105400.8	96082.3	2915.6	171.9	12994.3	35621.1	17198.7	179732.5	196057.1	98658.8	5057.7	5032.3	39.0	11989.6	930.3	4355.8	68.8	126141.2	116.6	268.2
24414.6	10152.6	106223.0	89449.2	2754.0	140.1	13534.7	34122.1	17487.7	172758.6	187672.3	84260.4	4459.6	4495.7	33.5	10674.8	793.7	3877.0	64.9	108671.2	110.9	220.2
21457.2	9816.6	93602.9	89779.4	2707.8	117.0	11567.9	33334.0	14840.1	165342.7	174279.5	82173.3	4490.5	4733.1	36.3	13426.8	841.2	3888.0	64.6	109671.6	147.0	193.7
25889.0	11748.6	112269.1	100011.0	3314.1	177.2	14325.7	37748.3	18737.0	189948.0	205428.6	102230.3	5013.9	5226.3	33.7	12994.0	828.6	4493.9	67.2	130898.0	153.1	245.7
24868.2	11431.8	113248.2	105665.2	3163.7	150.6	14392.1	39258.0	18623.1	202425.8	187619.5	83440.5	4622.0	4717.3	34.3	14391.4	774.6	3596.0	63.2	111649.5	140.0	238.3
25147.5	13168.2	124455.0	129755.0	4033.8	209.4	15301.7	44529.1	21421.8	225429.1	216672.8	79959.9	4868.7	5490.6	37.2	13850.7	769.4	4288.2	58.4	109338.4	305.1	223.1
24528.6	13602.0	119933.8	132847.1	3970.0	224.8	14626.0	47123.2	19503.3	232824.9	203998.5	86586.3	4786.8	4877.3	59.3	13264.1	772.2	4220.8	61.8	114651.6	195.5	256.2
22514.2	10933.0	102970.7	96334.4	2798.2	157.5	13294.5	36513.8	16472.5	181674.1	186049.8	70754.3	4301.1	4321.4	36.0	11548.6	775.6	3447.2	62.2	95252.8	127.4	193.5
24073.1	12545.1	105365.3	112543.5	3696.4	177.4	13135.7	39824.8	16979.3	199739.0	188671.8	84705.0	4703.2	4869.2	34.4	11613.2	748.2	4176.0	56.4	110917.7	141.3	259.9
23570.1	12336.5	109473.3	113616.9	3368.8	157.5	13236.0	41759.1	17909.7	207924.6	187307.6	76592.4	4195.4	4470.8	46.1	12572.6	663.1	3806.4	51.3	102411.8	161.4	239.4
20424.6	10525.7	90038.5	91740.6	2590.4	152.3	11205.6	33955.6	14112.6	175117.6	174050.3	86020.1	4583.0	4782.6	39.3	11153.9	709.4	3683.4	59.1	111051.1	136.4	203.0
21766.5	11208.2	101835.2	101812.2	3132.6	146.4	12016.5	36454.9	17027.1	184717.7	183695.4	85457.7	4618.3	4984.3	41.6	12101.3	916.6	4087.2	57.8	112275.7	155.3	234.6
22951.7	10916.1	106622.8	93112.4	3122.2	165.7	12912.7	34314.3	18551.4	174355.3	185146.0	88725.4	4308.2	4537.5	33.3	13137.9	758.1	3877.5	57.4	115443.0	135.9	224.0
21410.1	11624.2	106969.5	109286.0	3232.0	159.0	12690.6	38397.2	18759.1	215438.1	188845.1	77310.3	4361.9	4565.7	39.1	12020.1	664.2	2867.9	46.1	101886.4	170.8	241.7
24806.8	10173.2	106586.7	97320.0	2726.8	120.1	13917.6	36319.6	15450.6	175267.1	175617.2	83961.0	4173.1	4187.8	31.9	9365.4	652.0	3610.7	58.0	106050.3	103.7	197.1
23255.3	12691.7	106977.7	121543.0	3667.4	171.5	13267.1	43778.1	16797.8	215502.0	183949.6	108651.6	4593.4	4821.4	47.4	13895.3	701.6	3630.3	62.2	136422.0	191.5	215.7
22075.6	10251.3	105703.9	98923.4	2843.2	127.9	12941.0	36585.4	16696.8	180674.9	171091.9	90756.7	4315.7	4310.2	38.3	11563.8	608.6	3251.5	60.9	114923.1	113.9	235.9
22615.5	8817.0	102872.3	81650.0	2455.5	94.9	12961.4	31767.3	16532.5	154988.9	170488.9	85896.8	4111.9	4358.6	41.8	12110.7	605.6	3223.7	55.7	110425.8	145.9	183.4
24099.4	11840.2	110547.5	112884.1	3352.9	126.9	13066.9	40555.2	17772.1	202927.1	179002.5	82974.9	4411.2	4596.7	46.4	13518.6	615.2	3736.7	81.8	109996.2	149.8	216.8
21059.1	8625.8	93691.6	81990.8	2470.9	92.4	11961.9	31434.7	14850.4	157743.8	151290.5	67806.1	4067.3	4391.6	42.4	12779.7	547.2	3651.7	59.8	93369.0	155.3	169.3
21794.7	9087.8	104099.6	86441.8	2565.5	90.8	13607.6	33388.5	16795.8	164602.4	161114.0	72983.8	4247.5	4411.9	37.3	11405.3	613.8	3005.0	54.3	96770.6	112.2	201.1
20517.2	7924.5	96696.2	62249.0	2404.4	110.0	13677.9	26342.2	17511.5	138526.3	173618.6	86423.4	3428.8	3477.5	32.4	9173.0	584.7	2841.2	63.5	106032.5	79.7	199.7
23190.7	12104.6	102193.6	106059.8	3305.8	189.8	14337.6	39358.1	15572.3	203677.8	195483.4	84421.2	4807.1	5100.6	32.7	10379.7	814.6	3927.9	66.1	109556.3	145.2	336.9
23153.3	12948.5	104774.2	110755.6	3684.9	228.3	14864.9	40653.6	16370.0	210124.6	220245.9	93883.0	4885.4	5005.9	40.8	11380.5	831.8	3586.4	65.4	119696.8	144.8	232.8
22271.8	12458.2	106419.2	105087.7	3675.0	177.3	14307.1	38744.5	18568.7	207336.5	206811.9	99446.3	4393.5	4631.1	37.6	11709.9	695.1	4531.0	58.5	125514.5	154.3	197.7
24041.7	12223.8	106805.5	104200.4	3324.2	183.8	13360.8	38498.0	17633.4	197533.9	203637.7	94980.9	5232.6	5469.0	39.0	12937.2	817.2	4418.0	66.7	123978.2	150.3	260.3
22540.3	12169.1	102426.8	123324.4	3650.3	126.0	12066.0	42321.0	15955.6	213094.0	162462.5	83479.2	4731.9	4884.7	41.5	12930.1	596.1	3071.8	57.9	109810.0	144.2	281.3

Summary

Data:	X	dimension:	53	109											
	Y	dimension:	53	1											
Fit	method:	kernelpls													
Number	of	componen	considerec	52											
TRAINING	%	variance	explained												
	1comps	2comps	3comps	4comps	5comps	6comps	7comps	8comps	9comps	10comps	11comps	12comps	13comps	14comps	15comps
X	14.20	25.33	29.6	33.48	38.04	45.41	49.75	53.38	56.9	59.56	61.55	63.32	65.56	67.16	68.45
sio251	73.42	86.45	95.05	97.53	98.71	99.05	99.41	99.67	99.81	99.89	99.93	99.95	99.96	99.97	99.97
	16comps	17comps	18comps	19comps	20comps	21comps	22comps	23comps	24comps	25comps	26comps	27comps	28comps	29comps	
X	69.59	70.9	72.41	73.82	75.28	76.79	78.38	79.39	80.88	81.95	83.47	84.32	85.23	86	
sio251	99.98	99.98	99.98	99.99	99.99	99.99	100	100	100	100	100	100	100	100	
	30comps	31comps	32comps	33comps	34comps	35comps	36comps	37comps	38comps	39comps	40comps	41comps	42comps	43comps	
X	86.86	87.87	88.71	89.73	90.47	91.07	92.06	92.71	93.35	93.81	94.25	94.77	95.46	96.24	
sio251	100	100	100	100	100	100	100	100	100	100	100	100	100	100	
	44comps	45comps	46comps	47comps	48comps	49comps	50comps	51comps	52comps						
X	96.72	97.27	97.71	98.18	98.62	98.96	99.24	99.65	100						
sio251	100	100	100	100	100	100	100	100	100						

>

Loadings

No.	intake	和名	英名	Factor1	Factor2	Factor3	Factor4	Factor5	Factor6	Factor7	Factor8	Factor9	Factor10	Factor11	Factor12	Factor13	Factor14	Factor15	Factor16
1	intake11	米	Rice	0.067597	0.021552	-0.02329	0.216039	-0.07041	-0.07691	-0.13952	0.059045	0.181868	0.122445	-0.15054	-0.0715	-0.08635	0.137029	-0.01376	-0.14946
2	intake12	食 パ ン	White bread	-0.11322	-0.02746	0.069424	0.004481	-0.11223	0.206589	-0.32811	0.157644	0.013649	-0.0362	-0.07723	-0.07137	0.065885	0.007508	-0.10218	-0.06367
3	intake13	生うどん・そば	“Udon”&“Soba	-0.03685	0.028877	0.091464	-0.22435	0.01662	0.283272	-0.1629	0.024934	-0.10823	0.104762	-0.17683	-0.10752	0.165044	0.034399	-0.09011	-0.02605
4	intake14	乾うどん・そば	“Udon”&“Soba	0.115276	-0.02863	0.158729	-0.23516	0.071754	0.068234	0.052961	-0.01202	-0.07368	0.110794	0.053239	-0.19471	0.11013	-0.06682	-0.04346	0.192132
5	intake15	スパゲッティ	Spaghetti	0.008069	-0.17044	0.06009	0.209131	-0.05032	-0.11008	-0.00296	0.0857	-0.09927	-0.01794	-0.01222	-0.00958	0.18905	-0.05294	-0.02707	-0.19616
6	intake16	中 華 め ん	Chinese noodl	0.054523	-0.14106	0.059702	0.004744	0.122976	-0.06782	-0.17617	0.188974	-0.11085	0.209384	-0.19721	0.075876	0.116967	-0.1674	-0.02184	0.139945
7	intake17	小 麦 粉	Wheat flour	-0.04462	0.079752	0.117052	-0.08905	-0.11102	0.025757	-0.03354	0.11466	0.000692	-0.01615	-0.12918	0.215493	-0.00422	-0.12756	-0.24129	0.140083
8	intake18	も ち	“Mochi”, rice-	0.032802	-0.15059	0.100379	0.042585	-0.07383	0.056008	-0.22984	0.096244	0.086206	-0.22226	0.247382	-0.02355	0.05993	-0.02613	0.089173	-0.15595
9	intake19	ま ぐ ろ	Tuna fish	0.042124	-0.20543	0.131629	-0.03512	0.147102	-0.11201	-0.05706	-0.13463	0.164052	-0.03367	-0.15807	0.182701	-0.16464	0.091555	-0.03222	0.178084
10	intake110	あ じ	Horse mackere	-0.02493	0.105847	-0.15704	0.226957	-0.20819	0.119284	0.088498	-0.09003	0.159636	-0.08456	-0.02376	-0.03054	0.163823	-0.15515	0.019846	0.060049
11	intake111	い わ し	Sardines	0.083836	0.083291	-0.22777	0.190536	-0.14973	0.155119	-0.10738	0.056271	0.110774	-0.11651	0.045265	-0.06526	0.015645	-0.05691	-0.10545	0.090936
12	intake112	か つ お	Bonito	0.025905	0.03951	0.127878	-0.14329	0.119019	0.022814	0.023463	-0.05237	0.095859	-0.05139	0.022992	0.282769	-0.36386	0.263131	-0.22639	0.116072
13	intake113	か れ い	Flounder	0.15108	0.064422	-0.17559	0.051971	0.010896	0.092874	-0.19808	0.183826	-0.09733	-0.03591	0.077425	-0.22534	0.110869	-0.0014	0.088511	-0.0473
14	intake114	さ け	Salmon	0.196489	-0.13232	0.02522	-0.01691	0.10384	-0.06267	0.084353	0.035749	-0.05873	0.100964	-0.17836	-0.00689	0.077168	-0.04452	0.106	-0.15172
15	intake115	さ ば	Mackerel	0.013637	0.142576	-0.175	0.17859	-0.16323	0.122005	-0.05493	-0.07415	0.040258	0.019727	-0.15024	0.215624	0.022841	-0.00824	-0.27036	0.171088
16	intake116	さ ん ま	Saury	0.20178	-0.05581	0.001467	-0.08374	0.121728	-0.00733	-0.01677	0.063026	-0.15131	0.078983	-0.1111	0.168488	-0.06282	-0.02323	-0.09156	########
17	intake117	た い	Sea bream	-0.05045	0.145803	-0.13005	0.130652	-0.26405	0.102981	0.125707	-0.08092	-0.02331	0.173631	-0.02585	-0.0904	0.140839	-0.05029	0.096929	0.014923
18	intake118	ぶ り	Yellowtail	-0.00799	0.107402	-0.06794	0.029544	-0.10399	0.174354	-0.10872	-0.00311	0.116077	-0.12373	0.120071	-0.20149	0.159963	-0.21169	0.071457	0.256471
19	intake119	い か	Cuttlefish	0.219048	-0.01426	-0.1023	0.010357	0.023206	0.070462	-0.13723	0.114158	-0.07784	0.014228	0.030899	-0.01659	-0.09284	0.082455	0.075768	-0.02504
20	intake120	た こ	Octopus	-0.06226	-0.06319	0.060781	-0.05107	-0.00893	0.260139	-0.07814	0.077264	-0.24147	0.153769	-0.02924	-0.04555	-0.04335	0.043923	0.068024	-0.07796
21	intake121	え び	Shrimps & lobs	-0.01611	0.077872	-0.18091	0.144838	-0.15961	0.234501	-0.06274	0.112534	-0.1448	0.019148	-0.07143	0.152713	-0.0593	0.091487	0.048449	0.065427
22	intake122	か に	Crabs	0.056928	0.043238	-0.20529	0.114733	0.082269	0.104446	-0.24011	0.070373	0.047949	-0.1755	0.250401	-0.23072	0.032793	0.126623	-0.0307	-0.02263
23	intake123	あ さ り	Short-necked	-0.00782	-0.13501	-0.01349	0.096108	-0.05627	0.084117	0.162723	-0.21319	0.178421	-0.08018	-0.01118	-0.09976	0.044352	0.059862	-0.05761	-0.01006
24	intake124	し じ み	Fresh water cl	0.135129	-0.05674	-0.10695	0.080198	0.098829	0.024263	-0.12672	-0.04892	0.066646	-0.01339	-0.08119	-0.08707	0.050948	-0.02594	0.225463	-0.1583
25	intake125	か き（貝）	Oysters	-0.04717	0.043124	-0.08626	-0.12572	0.025767	0.239918	-0.04253	-0.0527	-0.06696	0.203379	0.066466	-0.28563	0.094026	-0.01212	-0.21449	0.241847
26	intake126	ほ た て 貝	Scallops	0.197305	-0.0531	-0.05644	0.005037	0.009654	-0.0148	-0.02954	0.114074	-0.2004	0.074744	-0.18857	0.197153	-0.07639	-0.01155	-0.08338	0.098926
27	intake127	塩 さ け	Salted salmon	0.221112	-0.07989	-0.00123	-0.12524	0.074856	0.042166	-0.00643	0.141568	0.008931	-0.0454	-0.0528	0.039175	-0.05033	0.0425	-0.01252	0.084048
28	intake128	た ら こ	Salted pollock	0.186095	-0.05796	0.009398	0.064958	-0.10205	-0.0212	0.147777	-0.05652	-0.15168	-0.01581	0.091701	-0.08813	-0.00243	-0.21487	0.09396	-0.02057
29	intake129	しらす干し	dried young sa	-0.0229	-0.1125	0.075192	0.039161	0.078245	0.128269	-0.17067	-0.13814	0.158349	-0.02159	-0.20933	0.325496	-0.27534	0.106942	-0.07465	0.076553
30	intake130	干しあじ	Dried horse ma	-0.00389	-0.13857	0.031258	0.148991	-0.03002	0.025023	0.10409	-0.20849	0.309803	-0.14007	-0.10409	0.160143	-0.13656	-0.04708	0.026174	0.036692
31	intake131	かつお節・削り節	fish flakes, drie	-0.08409	-0.00372	0.042649	0.126483	0.088766	-0.22896	-0.06432	0.050029	-0.0301	0.109658	-0.04784	0.01957	-0.14382	0.180739	-0.12123	0.402832
32	intake132	牛 肉	Beef	-0.12285	0.126572	0.063182	0.178114	-0.18659	0.152434	-0.08602	0.094099	-0.13366	0.117708	-0.0112	-0.08936	-0.00792	-0.02284	0.111944	1.30E-01
33	intake133	豚 肉	Pork	0.137619	-0.18875	-0.04901	0.193347	0.000672	-0.07281	-0.0574	0.053862	-0.05822	0.084104	-0.13363	0.077988	-0.00919	-0.03987	-0.11997	0.218254
34	intake134	鶏 肉	Chicken	-0.0779	0.085778	-0.09814	0.326677	-0.23213	0.093233	0.099152	0.055658	-0.17629	0.117444	-0.0191	0.106905	0.012063	-0.06866	-0.1182	-0.03895
35	intake135	合いびき肉	Mixed ground r	-0.16191	0.145246	-0.07475	0.169644	-0.10815	0.108423	-0.16928	0.078752	-0.00348	-0.04234	0.039217	-0.03204	0.006639	0.018168	-0.06223	0.173814
36	intake136	ハ ム	Ham	0.038539	-0.15872	-0.072	-0.00137	-0.03988	0.098942	-0.15225	-0.03565	0.100116	-0.02122	0.093402	0.07115	0.123409	-0.3327	0.367739	0.159077
37	intake137	ソーセージ	Sausages	0.104486	-0.04068	-0.13406	0.150388	0.090664	-0.1137	-0.15227	0.202683	-0.03166	-0.03135	-0.05408	0.026314	0.101351	-0.12603	0.039012	-0.21024
38	intake138	ベ ー コ ン	Bacon	0.111542	-0.06689	-0.07658	0.21481	-0.00051	-0.17245	0.105169	-0.00166	-0.12591	0.139404	0.150298	-0.04461	0.00706	-0.07915	-0.09739	0.169507
39	intake139	牛 乳	Fresh milk	0.010096	-0.06039	0.13107	0.034787	-0.00504	0.255146	-0.26027	-0.01	0.08908	0.009261	-0.05714	0.043969	0.092913	-0.10625	-0.0006	-0.12374
40	intake140	粉ミルク	Powdered milk	0.003182	-0.0144	-0.12617	0.021227	-0.02662	-0.14144	0.178664	0.004022	0.007893	-0.06658	0.112586	-0.05318	-0.23078	0.234895	-0.0051	-0.02955
41	intake141	バ タ ー	Butter	-0.00871	-0.18948	0.148483	0.059616	-0.06189	0.181576	-0.17209	0.049083	-0.10491	0.059551	-0.00985	0.074003	-0.02483	-0.01915	0.008406	-0.18624
42	intake142	チ ー ズ	Cheese	0.011337	-0.25217	0.184819	0.067746	-0.02029	-0.03209	-0.0159	0.057378	0.014981	0.018747	-0.00542	-0.13012	0.073033	-0.07372	-0.02891	-0.01813
43	intake143	卵	Eggs	0.025517	0.172956	-0.10851	0.114723	0.048976	0.060114	-0.27307	0.177672	-0.05273	-0.03774	-0.02057	0.113828	0.022374	0.005707	-0.11851	0.084514
44	intake144	キャベツ	Cabbage	0.082914	-0.09543	0.069191	0.037254	-0.16292	0.107237	0.028313	-0.24976	0.04975	0.190278	-0.03483	0.034488	0.142356	-0.1864	-0.0735	-0.13728
45	intake145	ほうれんそう	Spinach	0.183325	-0.05768	0.131076	0.00071	-0.03389	0.031717	-0.18287	0.027744	-0.05718	0.156116	0.036814	-0.07033	-0.05233	0.056038	0.107492	0.131884
46	intake146	はくさい	Chinese cabba	-0.0394	0.028849	0.053483	0.036731	-0.20862	0.216221	-0.09933	0.02272	-0.06118	0.14845	-0.10564	0.137132	-0.24945	0.173056	0.106763	-0.05355
47	intake147	ね ぎ	Welsh onions	0.137819	-0.22264	0.09879	0.011559	-0.02891	0.094672	0.003702	-0.04096	-0.04089	-0.04608	0.074383	0.144295	0.04429	-0.01724	-0.02689	0.01752
48	intake148	レ タ ス	Lettuce	0.022453	-0.24157	0.026802	0.162782	-0.13292	0.034402	0.01374	-0.04924	0.066168	-0.01027	-0.03162	0.004783	-0.03238	0.007461	-0.09407	0.069837
49	intake149	ブロッコリー	Broccoli	0.129505	-0.20859	0.084486	0.001535	-0.0596	0.071741	-0.02801	0.104435	-0.07385	-0.00419	-0.06064	-0.05826	0.005533	0.048538	0.001707	0.009751
50	intake150	も や し	Bean sprouts	0.223636	-0.05524	-0.04555	-0.01476	0.012413	-0.05035	########	0.08722	0.011865	0.012021	-0.00167	-0.11992	0.07854	-0.04976	-0.00444	0.121258
51	intake151	さつまいも	Sweet potatoe	0.082341	-0.0903	-0.01299	0.037257	-0.01832	0.172802	0.043838	-0.07761	-0.00627	0.01994	-0.15507	0.251824	-0.32527	0.185346	-0.00939	-0.09481
52	intake152	じゃがいも	White potatoes	-0.00893	-0.11453	0.037676	0.224352	-0.20396	0.176592	-0.09626	0.015831	0.030883	-0.0387	0.09876	-0.07172	-0.02739	0.017042	-0.04932	-0.05915
53	intake153	さといも	Taros	0.031225	-0.00691	0.107016	0.064906	-0.21172	0.082019	0.052759	-0.20096	0.181426	-0.22879	0.140693	0.081991	0.01176	-0.16605	0.115364	-0.06366
54	intake154	だいこん	Radishes	0.152399	-0.19043	0.095483	-0.00871	0.015936	2.22E-05	0.041815	0.009872	0.048833	-0.00666	-0.01105	0.010461	-0.07762	0.048014	0.036771	0.020807
55	intake155	にんじん	Carrots	0.098456	-0.10783	0.03497	0.062167	-0.14131	-0.15149	0.075472	0.07419	0.02162	0.029851	-0.19885	0.00444	0.100655	0.054478	-0.2269	0.210951
56	intake156	ご ぼ う	Burdocks	0.120904	0.060691	-0.0569	0.064767	-0.14949	0.016875	0.186075	-0.13647	-0.08445	0.17576	-0.20035	0.06093	0.133651	-0.12041	0.064449	0.072549
57	intake157	たまねぎ	Onions	0.105568	-0.08772	0.017692	0.219533	-0.21187	0.041497	-0.08622	0.145403	-0.23777	0.115678	-0.10303	-0.02219	-0.03917	0.005994	0.019558	-0.2687
58	intake158	れんこん	Lotus roots	-0.01163	-0.11302	-0.06946	-0.02395	-0.11641	0.196533	0.139591	-0.17503	0.125169	-0.01629	-0.08134	-0.19191	0.101507	0.04175	-0.06207	0.145073
59	intake159	たけのこ	Bamboo shoot	0.081912	-0.00244	0.282301	0.062259	-0.14308	0.085587	0.008072	-0.03735	0.006114	-0.07503	0.103143	-0.2073	-0.03262	0.148835	0.076397	0.089416
60	intake160	さやまめ	String beans	0.110773	-0.14456	0.040868	0.033193	-0.10566	0.098613	0.081497	0.030733	0.046071	-0.12663	0.12375	-0.02807	-0.07701	0.100106	-0.16428	0.134993
61	intake161	かぼちゃ	Pumpkins	0.027679	-0.11931	0.020149	0.104643	-0.19289	0.244001	0.027178	-0.13723	0.046442	0.016249	0.025587	-0.07972	0.017846	-0.00055	-0.15594	0.190241
62	intake162	きゅうり	Cucumbers	0.105514	-0.09443	0.164594	0.029733	-0.1454	-0.02196	0.265904	-0.22227	0.072659	-0.08953	-0.02051	-0.07218	0.031822	0.020776	0.019126	0.143314
63	intake163	な す	Eggplants	0.115542	-0.07623	0.057576	0.00787	-0.30711	0.188313	-0.09385	0.039167	-0.0793	-0.00433	0.106636	0.0598	-0.12743	0.081121	0.043983	-0.06224
64	intake164	ト マ ト	Tomatoes	0.083942	-0.19252	0.088443	0.061955	-0.18785	0.0207	0.1303	-0.03325	0.066826	-0.09638	0.081058	0.034644	-0.04808	0.081569	-0.10908	0.172554
65	intake165	ピーマン	Green peppers	-0.00091	-0.17386	0.08659	0.27028	-0.15565	-0.05402	-0.03841	0.020812	-0.10039	0.046396	0.027742	0.003968	0.033855	0.167082	0.007961	0.198333
66	intake166	生しいたけ	fresh	0.086105	0.040524	-0.09194	0.031147	-0.07154	0.210032	-0.1231	-0.01327	-0.0558	0.080259	0.019768	0.04051	-0.12685	0.060869	0.090339	-0.12349
67	intake167	干ししいたけ	dried	-0.0136	0.084321	0.015111	0.269675	-0.17339	-0.03943	0.164382	-0.12154	-0.02503	-0.02869	-0.07712	0.07279	-0.10557	-0.10779	0.186423	0.07478
68	intake168	わかめ	“Wakame”, sea	0.184494	0.021023	0.009438	0.033039	0.018813	-0.0119	0.018016	-0.08855	0.120417	0.08609	-0.03551	0.007203	0.048334	-0.01834	0.019652	-0.20807
69	intake169	こんぶ	Dried tangle	0.17856	0.012441	0.137896	-0.0098	0.042431	-0.06124	-0.11996	0.066655	-0.07393	0.074324	0.041061	0.013425	-0.03058	-0.08661	-0.09766	0.169563
70	intake170	豆 腐	Bean curd	0.142527	-0.03033	-0.03184	-0.01101	-0.07031	0.063553	-0.09947	-0.03543	0.165084	-0.11994	-0.15914	0.129815	0.176941	0.0779	-0.20655	0.178811
71	intake171	梅 干 し	pickled plums	0.024194	-0.07626	-0.0831	-0.09418	0.058579	0.038145	-0.02968	0.089529	-0.14046	0.106119	-0.11808	0.249204	-0.14446	-0.05289	0.14547	-0.20791
72	intake172	だいこん漬	Pickled radishe	0.156165	0.042057	-0.05972	-0.14221	-0.0636	0.04459	-0.15854	-0.04725	0.207506	0.016359	-0.02222	0.013696	-0.20839	0.141438	0.016563	-0.24128
73	intake173	はくさい漬	chinese cabba	0.060628	0.027021	-0.04779	-0.10055	-0.04402	0.152416	-0.13005	-0.01946	0.147349	-0.12866	0.194483	-0.13069	-0.10962	0.18411	-0.22776	0.319477
74	intake174	こんぶつくだ煮	in soy sauce	0.075322	-0.00693	0.111182	-0.11736	-0.0442	0.07488	-0.19275	0.187716	0.127388	-0.19152	0.106896	0.009691	-0.05984	-0.09158	0.258065	-0.27431
75	intake175	りんご	Apples	0.197989	0.017508	0.050839	-0.2267	0.020524	-0.00257	0.021137	0.049085	-0.07301	0.09929	-0.1463	0.116508	0.039303	0.038842	-0.08738	0.118523
76	intake176	みかん	Mandarin oran	0.036695	0.030098	-0.07971	0.118708	0.015811	0.130001	-0.0106	-0.2998	0.238128	-0.02406	-0.128	0.098198	-0.06658	0.23837	-0.16121	-0.1601
77	intake177	グレープフルーツ	Grapefruits	0.116155	-0.20129	0.070607	-0.07614	0.005448	0.001061	0.030974	0.10916	-0.0055	-0.07705	0.167924	-0.07551	-0.0387	-0.0626	0.069893	0.023473
78	intake178	オレンジ	Oranges	0.048658	-0.07755	-0.02856	-0.03217	0.178256	0.054744	-0.22418	0.006511	0.112261	0.003158	0.099761	-0.02502	0.116765	-0.33691	0.107611	0.382695
79	intake179	な し	Pears	0.134031	0.093757	-0.01369	0.001962	0.030364	0.060765	-0.03382	-0.07284	0.142364	-0.29471	0.141725	-0.16004	0.054154	0.040897	0.021584	0.185435
80	intake180	ぶどう	Grapes	0.0419	-0.00504	0.067889	-0.06151	0.10503	0.096795	0.069233	-0.29622	0.06018	0.277781	-0.154	-0.05781	0.130502	-0.06485	0.118654	-0.14074
81	intake181	かき(果物)	Persimmons	0.001695	0.023559	-0.11001	0.024183	-0.01399	0.114217	-0.21939	0.014185	-0.02668	0.132296	0.126517	0.011412	-0.29753	0.193347	-0.09563	0.182491
82	intake182	も も	Peaches	0.044301	-0.03542	0.121153	-0.25364	0.083577	0.032138	0.042983	-0.20509	-0.02695	0.166039	-0.10899	0.011707	0.092553	0.058844	0.061589	-0.0417
83	intake183	すいか	Watermelons	0.074064	-0.04398	-0.0747	0.131777	-0.11783	0.143872	-0.01854	-0.13938	-0.00477	-0.18101	0.299521	-0.01165	0.122052	0.002448	-0.02272	0.058793
84	intake184	メロン	Melons	0.105029	-0.06637	-0.21962	0.033986	0.061823	0.024905	0.128183	-0.00694	-0.02836	-0.05846	0.122288	0.169232	-0.18467	-0.06565	0.191534	-0.18453
85	intake185	いちご	Strawberries	0.028074	-0.13535	0.057457	-0.14998	-0.05078	0.110634	0.085192	-0.06091	0.062433	-0.23534	0.024632	0.12854	0.031988	-0.04955	0.0257	-0.1328
86	intake186	バナナ	Bananas	0.064991	0.020468	-0.03571	-0.05816	-0.02552	0.240782	-0.17027	0.101239	0.026349	-0.14322	-0.25897	0.185493	0.021929	-0.04286	0.003619	0.384503
87	intake187	キウイフルーツ	Kiwi fruits	0.074399	-0.15571	-0.01744	0.044	0.060984	0.007851	0.016886	0.009079	-0.06526	0.024552	0.201115	-0.25326	0.010769	0.237696	-0.13745	-0.07239
88	intake188	食用油	Edible oil	0.0528	0.074743	-0.21923	0.200801	-0.06841	-0.11932	0.090796	-0.00901	-0.04895	0.159367	-0.10298	-0.00414	-0.00235	0.072825	-0.02318	0.174168
89	intake189	マーガリン	Margarine	-0.08408	0.008863	0.063873	0.071846	-0.17429	0.172175	-0.22317	0.254675	0.028903	-0.02533	-0.08594	0.171006	0.022015	-0.09212	0.062962	-0.02569
90	intake190	食 塩	Salt	0.213955	0.117424	0.16099	0.101756	0.097588	-0.03678	0.080147	0.052632	-0.00181	-0.00133	0.06112	0.025293	-0.02764	0.050124	-0.03846	0.089953
91	intake191	しょう油	Soy sauce	0.065333	0.161714	0.059307	0.159767	-0.07802	0.084151	0.069587	-0.0687	0.084229	-0.04663	-0.09393	-0.01908	0.121615	-0.09803	0.22747	-0.26305
92	intake192	み そ	“Miso”, soybe	0.180022	0.027541	-0.06748	0.089635	-0.0089	-0.09953	0.098987	-0.21713	0.128995	0.015286	0.122243	0.047544	-0.05568	-0.04121	0.003748	0.044958
93	intake193	砂 糖	Sugar	0.011607	0.155269	-0.14141	0.104722	-0.09061	0.101667	-0.02133	-0.13174	-0.03856	0.070317	-0.07275	0.134978	-0.24648	-0.06397	0.189545	-0.03654
94	intake194	酢	Vinegar	-0.00456	-0.00919	-0.09074	-0.05267	-0.06937	0.060551	0.007255	0.031339	-0.06981	0.327845	-0.22996	0.133819	-0.043	0.015052	0.117793	0.198897
95	intake195	ソース	Worcester sau	-0.07981	0.05957	0.021446	-0.07705	-0.14378	0.223437	-0.20548	0.106264	-0.09324	0.215267	-0.01508	0.03274	-0.08924	0.032322	-0.06733	-0.01177
96	intake196	ケチャップ	Tomato ketchu	-0.11886	-0.05937	-0.03941	0.219619	-0.06668	0.021156	-0.06132	0.268633	-0.1671	-0.03123	-0.01404	0.076984	-0.0913	0.086106	-0.00768	0.042828
97	intake197	マヨネーズ・	seasoning	0.089199	0.125936	-0.14837	0.164663	-0.13237	0.041496	-0.07867	0.058413	0.043076	0.012992	0.019585	0.009955	0.050635	-0.0636	0.10419	-0.31235
98	intake198	ドレッシング	Dressing	0.00805	-0.04683	0.066976	0.09182	-0.16957	-0.17929	0.027324	-0.03963	0.199439	0.021159	-0.0165	-0.12767	0.06065	-0.04474	0.060865	0.026107
99	intake199	ジャム	Jam	-0.02875	-0.12065	0.183768	-0.10677	-0.07745	0.170356	-0.18487	0.204879	-0.04939	0.00288	-0.09861	0.137324	-0.01683	-0.08917	0.19785	0.025316
100	intake1100	カレールウ	Instant curry n	0.097557	0.064485	-0.11726	0.108858	-0.13344	0.018664	-0.1172	0.185175	0.066343	-0.03833	-0.14305	-0.20034	0.257783	0.128256	-0.1781	-0.08748
101	intake1101	緑 茶	Green tea	-0.01468	-0.0627	0.0223	0.134428	-0.17137	0.092521	-0.188	-0.03474	0.302971	0.028909	-0.08204	0.042988	-0.15768	0.091633	0.068759	-0.2232
102	intake1102	紅 茶	Black tea	0.012722	-0.12334	0.198826	0.057741	-0.20487	0.074533	0.028755	0.064366	-0.07256	0.144874	0.031785	-0.11117	-0.00112	-0.03412	-0.00082	0.058844
103	intake1103	コーヒー	Coffee	-0.0077	-0.01493	0.071233	0.000165	-0.04649	0.141421	-0.20204	0.223452	-0.2818	0.052701	0.108328	-0.18517	0.075636	0.056874	0.023262	-0.01619
104	intake1104	清 酒	“Sake”	0.136386	-0.03245	0.06499	-0.19264	0.049336	0.101609	-0.07965	-0.04624	0.063362	-0.00113	0.108535	-0.18157	0.182521	-0.05274	-0.11573	-0.22097
105	intake1105	焼ちゅう	distilled spirits	0.100031	0.122685	-0.11287	0.103834	-0.16592	-0.07788	0.177774	-0.0508	-0.11245	0.016938	-0.05561	0.143795	-0.16342	0.126057	0.065908	-0.09905
106	intake1106	ビール	Beer	0.035184	-0.08465	-0.02689	0.137342	-0.01722	0.074215	0.000355	-0.14195	-0.19319	0.165767	0.179846	-0.12104	0.079511	0.024938	-0.21064	-0.03094
107	intake1107	ウイスキー	Whisky	0.161791	-0.15276	-0.02756	-0.0106	-0.0255	-0.02867	0.043345	0.127028	-0.15026	0.078248	-0.0676	-0.01806	-0.03515	0.009174	0.044809	-0.0501
108	intake1108	ワイン	Wine	-0.00448	-0.17661	0.149675	0.005538	-0.01932	-0.06849	0.051203	0.028115	-0.06766	0.051353	0.004027	-0.08449	0.019531	0.108762	0.072246	-0.16642
109	intake1109	発泡酒・	alcoholic bever	0.064095	0.102257	0.109568	-0.07321	-0.10621	0.014086	0.002002	0.170093	-0.12272	-0.13352	0.224672	0.216498	-0.21536	0.073354	-0.23097	0.072472

No.	intake	Factor17	Factor18	Factor19	Factor20	Factor21	Factor22	Factor23	Factor24	Factor25	Factor26	Factor27	Factor28	Factor29	Factor30	Factor31	Factor32	Factor33	Factor34	Factor35	Factor36
1	intake11	0.07575	0.131906	-0.16134	0.209023	-0.2623	0.251649	-0.19022	0.203707	-0.31393	0.096829	0.069785	-0.07266	0.076474	-0.04218	-0.09883	0.234078	-0.11813	0.060472	-0.04312	0.039085
2	intake12	-0.03524	0.021554	0.018949	-0.02096	0.150549	-0.04025	0.008212	-0.11177	0.034575	0.003491	-0.00555	-0.04186	0.087685	-0.06992	0.172296	-0.02783	-0.0119	0.025334	-0.05114	0.076778
3	intake13	0.006972	-0.03781	0.13569	0.097289	-0.06647	-0.03273	-0.0523	-0.05637	0.103487	-0.02875	-0.02595	-0.01475	-0.1592	0.114669	-0.01347	0.064445	0.052092	-0.10635	0.064375	0.162344
4	intake14	-0.17631	0.121839	-0.21931	0.151289	0.132201	-0.02949	0.100074	-0.19964	-0.17885	0.119463	0.07346	0.145746	-0.03009	0.075054	-0.23037	0.174447	0.082763	-0.18316	0.10911	0.011427
5	intake15	0.222429	-0.24161	0.121736	-0.04801	0.239373	-0.19752	-0.08417	-0.02962	0.173167	-0.01954	-0.04589	-0.12886	0.108265	-0.03898	-0.18651	0.284888	-0.1097	0.109228	-0.15129	-0.01605
6	intake16	-0.15872	0.06626	0.098003	0.022551	-0.13507	0.078396	-0.04141	-0.02143	-0.02783	0.138522	0.026378	-0.31807	0.123712	-0.07118	-0.08359	0.031699	-0.05231	0.09603	0.011365	0.007618
7	intake17	0.184516	-0.32903	0.208476	-0.19945	0.188766	-0.03257	-0.1101	0.052323	-0.27835	0.254616	0.027256	-0.0872	0.005892	-0.11618	0.032423	-0.15806	0.300287	-0.30765	0.190834	-0.10893
8	intake18	0.052151	0.169969	-0.17938	0.006763	-0.00196	0.190702	-0.05178	-0.01425	-0.03694	-0.01359	0.096584	0.114439	-0.0365	-0.14173	0.013612	0.105202	-0.0215	0.050234	-0.12311	0.071478
9	intake19	-0.07721	0.017188	6.30E-02	-0.09175	-0.05085	-0.01272	0.08722	-0.02713	-0.03457	-0.01439	0.182448	-0.00588	-0.02696	0.003161	0.006678	0.112484	-0.01572	-0.0301	0.042529	0.093211
10	intake110	-0.03199	0.097121	-0.10002	-0.10874	0.140521	-0.14697	0.11803	-0.12663	0.157169	-0.07841	-0.0256	0.101832	0.095212	-0.17924	0.079783	0.004515	0.059386	0.18401	-0.07411	0.130065
11	intake111	-0.08191	0.062046	0.084603	-0.27266	0.127893	-0.07479	0.1275	-0.00743	0.023186	-0.02634	-0.03543	-0.05868	0.123911	0.034034	0.06267	-0.11192	-0.00219	0.060841	0.058365	-0.02904
12	intake112	-0.03456	0.023941	0.036193	-0.0328	0.138317	-0.22749	0.070818	0.014271	0.113754	-0.17889	0.009384	0.03588	-0.02844	-0.01731	0.060177	0.04705	-0.24234	0.166186	0.156323	-0.18679
13	intake113	-0.01461	0.006365	-0.06088	-0.07669	0.160306	-0.07944	-0.07749	0.019279	0.036418	0.027345	0.062024	-0.0094	-0.02914	0.04246	0.024408	-0.1645	0.064458	-0.08597	0.073013	-0.15839
14	intake114	-0.0314	0.017431	-0.1044	0.015465	0.054451	0.082162	-0.07815	0.075277	-0.1186	-0.02839	0.132509	-0.00183	-0.08695	0.034836	-0.03049	-0.03709	0.089666	-0.04025	-0.02672	0.028453
15	intake115	-0.14562	-0.02301	0.074073	-0.16484	0.146552	0.065233	0.011142	-0.09608	0.099377	-0.08229	0.017377	0.011254	-0.05999	0.066686	0.142399	-0.03807	-0.10381	0.006254	0.191614	-0.01067
16	intake116	0.035774	0.031895	-0.01624	0.045175	-0.06437	-0.05863	0.052723	0.042291	-0.14633	0.195895	-0.07986	-0.04472	0.07324	-0.01199	0.059508	-0.00801	-0.1492	-0.00761	-0.17772	-0.00349
17	intake117	-0.13011	0.231192	-0.12755	0.024347	-0.02201	0.024108	-0.05706	0.017704	0.034508	0.051839	-0.02025	-0.06667	0.065395	-0.15038	0.076895	-0.02098	-0.11149	-0.01276	0.110148	-0.16571
18	intake118	-0.11017	-0.10371	-0.02839	0.213978	-0.09538	-0.02401	0.095631	-0.15586	0.281764	-0.19251	-0.02493	0.062983	0.121453	-0.26775	0.166994	-0.12375	0.123947	-0.06693	-0.07227	0.117753
19	intake119	0.087348	-0.02656	0.02693	-0.07362	-0.06012	0.020333	0.040321	-0.03007	0.019419	-0.07951	0.158886	-0.0429	0.064251	0.05921	0.112793	0.05025	-0.07443	0.017679	-0.03169	0.076315
20	intake120	0.071161	-0.0884	0.058734	0.126739	-0.06538	-0.13407	0.050207	0.183696	-0.11425	0.026501	0.03398	0.173091	-0.34201	0.184561	-0.04144	-0.11368	-0.02322	-0.03105	0.037566	-0.00881
21	intake121	-0.12358	-0.10402	0.062079	-0.04334	-0.07008	0.245429	-0.12583	0.044928	0.074488	-0.05421	0.017852	-0.04741	0.009274	-0.18447	0.093616	0.086075	0.009072	-0.23583	0.086729	-0.00411
22	intake122	0.020273	-0.07708	0.114694	0.004928	0.113686	-0.06668	-0.13685	0.067255	-0.1107	0.030032	0.074903	-0.0116	-0.07975	0.009139	-0.09945	-0.01005	0.143908	-0.15222	0.117737	-0.15625
23	intake123	-0.10243	-0.19062	0.270257	-0.15953	-0.03574	0.168174	-0.06365	0.044187	-0.0399	-0.06559	0.215359	-0.21273	0.231523	0.127648	-0.18398	-0.09817	0.080041	-0.08692	0.012889	-0.01255
24	intake124	0.053795	0.094026	-0.24611	-0.18938	0.4267	-0.18122	0.017575	-0.11048	0.201633	-0.13081	0.008691	-0.00471	-0.08682	0.068993	0.169343	-0.08196	-0.09672	0.04083	-0.17444	-0.02463
25	intake125	-0.05162	0.20709	-0.03904	-0.08594	-0.01749	-0.14236	0.142306	0.094538	-0.10427	0.054221	-0.07269	0.088271	-0.10682	0.063	-0.21978	0.228236	0.037561	-0.15066	0.090456	0.016726
26	intake126	0.040843	-0.05102	0.171119	-0.02454	-0.13465	0.052771	0.042531	0.068416	-0.21524	-0.00875	0.036329	-0.10413	0.019574	-0.05041	0.119348	-0.0049	-0.04322	0.053366	-0.05086	0.017828
27	intake127	0.011401	-0.08296	-0.02525	0.00117	0.009538	0.081592	-0.02523	0.005634	-0.0811	0.028719	0.050271	-0.03376	-0.14245	-0.12774	0.059283	-0.05889	0.079168	0.076887	-0.04954	-0.06447
28	intake128	0.052771	-0.06141	0.038714	0.070598	-0.14666	0.112267	-0.0567	0.039606	-0.00975	-0.05463	0.109253	0.009925	0.003688	-0.04254	0.041071	0.023856	-0.03425	0.040281	0.243607	-0.28168
29	intake129	-0.02391	-0.05702	-0.0713	0.042933	0.12247	0.059034	-0.21296	0.040828	0.109628	-0.13011	0.147422	0.040718	-0.04001	-0.12492	-0.06423	0.008128	-0.0376	-0.11187	0.169027	-0.04323
30	intake130	0.074984	-0.11113	0.097602	-0.09427	0.128371	-0.12849	0.073065	-0.06094	0.134891	-0.08947	0.187902	-0.23369	-0.0713	0.048872	0.023582	0.010637	-0.04869	0.140099	-0.13869	0.011265
31	intake131	-0.38834	0.352231	-0.07148	-0.16028	0.085467	0.088461	-0.07162	-0.03813	-0.1009	0.114707	-0.01252	0.097207	-0.00965	-0.09991	0.1562	-0.05701	0.121156	-0.05501	-0.02221	0.033906
32	intake132	-0.15562	0.043218	0.027413	0.073616	-0.10764	0.019779	-0.01836	0.065193	-0.02267	-0.01306	0.031196	-0.08506	0.06017	-0.02021	0.004395	0.103065	-0.03293	0.098335	0.006506	-0.05031
33	intake133	0.047847	-0.17108	-0.08881	0.069363	0.161813	0.001421	-0.10423	0.03388	0.021133	-0.05979	-0.00903	0.015232	-0.01571	0.10069	-0.04311	0.115316	-0.02732	-0.09154	0.038876	0.093088
34	intake134	0.129073	-0.07438	0.058713	-0.08569	0.114199	-0.07423	0.031936	-0.02698	0.060289	-0.07984	0.010581	-0.03236	-0.01098	0.039571	-0.03682	0.06343	-0.06511	-0.05205	0.056234	-0.0996
35	intake135	-0.08855	-0.0989	0.082449	0.019963	0.040341	-0.05591	-0.08167	0.022686	0.132895	-0.0455	-0.00078	-0.02797	0.04806	-0.12801	-0.036	-0.04588	0.068933	-0.01877	0.005922	0.073304
36	intake136	-0.10207	-0.17276	-0.07548	0.296802	-0.01408	-0.09173	-0.07496	0.099824	0.001719	-0.03999	-0.00622	-0.09208	0.052029	0.047097	0.076513	0.169722	-0.1031	-0.12097	0.08113	-0.02372
37	intake137	0.162878	-0.20618	0.159431	0.023347	-0.15186	0.103566	-0.0491	0.129826	0.031092	-0.22989	0.071826	0.166619	-0.17056	-0.17169	0.099723	-0.02642	0.101582	-0.04739	-0.23925	0.133204
38	intake138	-0.11811	0.251214	-0.13671	-0.04397	-0.03839	-0.05144	0.072945	0.112232	0.117632	-0.11255	-0.0358	-0.16404	0.155258	-0.16441	-0.00056	-0.01782	0.129529	-0.23461	0.012697	0.125486
39	intake139	0.012728	0.053945	0.024716	0.097287	0.022983	-0.18505	0.069843	0.033331	-0.12204	0.232939	-0.21854	0.035575	0.13262	0.065009	-0.14833	-0.06118	0.071032	0.162356	-0.20398	0.089815
40	intake140	-0.19828	0.295592	-0.03055	0.062312	0.176377	-0.1866	-0.24088	0.182028	-0.16431	0.056954	0.088124	-0.08617	0.140062	-0.09271	0.108748	-0.32818	0.294709	-0.01657	-0.21879	0.072561
41	intake141	0.144826	-0.17664	0.159436	-0.05898	0.01467	-0.16752	0.163039	-0.00995	-0.09872	0.074628	0.017195	-0.00727	0.153958	-0.08929	-0.04061	0.035847	-0.08303	0.095608	-0.02431	0.022212
42	intake142	-0.01126	-0.04567	0.042746	0.010431	0.06656	-0.14929	0.103947	0.122514	-0.15912	0.096678	-0.17828	0.134769	0.119862	-0.09127	0.014105	0.00771	-0.05173	-0.06749	0.084308	-0.05563
43	intake143	-0.15203	-0.02254	0.092242	0.05269	0.01652	-0.104	-0.14061	0.302788	-0.03781	-0.01348	-0.06346	0.077784	-0.04269	0.120035	-0.04051	0.061064	0.056579	-0.12825	0.096717	0.031396
44	intake144	0.151	0.011183	0.066058	-0.24208	0.096185	0.058476	0.030282	-0.17339	0.161931	-0.12067	0.031539	0.185225	-0.13401	0.077172	-0.07202	-0.11171	0.11807	-0.02173	-0.201	0.136653
45	intake145	-0.24759	0.138443	-0.09053	-0.0349	0.158339	-0.061	-0.01839	-0.13242	0.129314	0.088028	-0.08437	-0.06755	-0.04465	0.043817	0.080894	-0.07393	-0.08546	0.113634	0.008564	0.013061
46	intake146	0.015145	-0.08013	-0.05336	0.103321	-0.00864	0.028842	0.094902	-0.15521	0.140018	-0.21994	-0.00884	0.006933	-0.09734	0.056531	0.047872	-0.18746	0.17867	0.07513	0.090072	-0.08584
47	intake147	0.109285	0.059066	-0.08877	0.084608	0.028347	-0.04843	-0.02721	-0.12995	-0.01544	0.100651	-0.06361	0.039205	-0.057	0.125604	-0.11117	-0.02787	-0.1389	0.080281	0.022068	0.02079
48	intake148	0.085073	0.004831	0.24548	-0.29367	0.091774	-0.17937	0.097147	0.042427	0.071185	-0.0215	0.119727	-0.01905	-0.02297	-0.02085	0.056027	-0.06677	0.080164	0.108988	-0.11625	0.036626
49	intake149	0.098035	-0.0753	0.101481	-0.17076	0.06006	-0.02839	0.028364	0.046416	0.031141	0.013443	########	0.023111	0.066081	-0.03171	0.039473	-0.06941	0.015359	-0.01053	-0.08135	0.078386
50	intake150	-0.00963	-0.08774	0.115665	0.168572	-0.15732	-0.00317	-0.05071	-0.03647	0.174763	-0.07003	0.011565	-0.06532	0.077079	0.077029	-0.20145	0.106346	-0.01681	-0.11195	0.068755	0.089139
51	intake151	0.005735	0.038345	0.005916	-0.04062	-0.16513	0.241467	-0.07561	-0.17692	0.106873	0.076614	-0.12002	0.151622	0.182488	-0.01487	-0.04582	0.029569	0.147296	-0.15222	-0.04994	0.078914
52	intake152	0.049236	0.2134	-0.33133	0.157736	-0.11221	0.182499	-0.07124	0.055554	0.012002	-0.19377	0.101578	0.15878	-0.17636	0.141328	-0.14894	-0.04581	0.093418	0.010013	-0.03397	0.051438
53	intake153	-0.02975	-0.03004	-0.08366	0.247697	-0.16197	0.191325	-0.17432	0.141648	-0.23181	0.139032	-0.13611	0.055145	-0.00162	0.155659	-0.17818	0.154158	-0.0748	0.105846	-0.15418	-0.17713
54	intake154	-0.0946	0.106783	-0.12422	0.15118	-0.00494	-0.03453	0.018759	-0.04016	0.082414	-0.05397	-0.06751	-0.10175	-0.08073	0.211608	-0.05755	-0.08817	0.148921	-0.13774	0.165424	-0.18932
55	intake155	-0.15847	0.312326	-0.21886	0.02536	0.043021	0.022947	-0.05005	0.039032	0.120165	-0.00218	-0.28773	0.167762	-0.11734	0.23498	-0.08839	-0.0755	0.107977	-0.00167	-0.10687	-0.03823
56	intake156	-0.15015	0.120316	-0.11673	0.259579	0.020807	-0.18499	0.043131	0.050787	-0.13866	-0.05411	0.145105	-0.03047	0.108866	-0.03725	-0.08083	0.12897	-0.09483	0.06596	0.048643	-0.2111
57	intake157	0.046577	0.104291	-0.06232	0.0548	-0.11076	0.03401	0.050366	0.005005	0.089886	0.040234	-0.02357	0.087588	-0.2058	0.037626	0.011138	-0.08816	0.019616	0.040326	0.143797	-0.14263
58	intake158	-0.01855	0.004568	-0.08841	0.157448	-0.10248	0.087422	-0.22881	0.270696	-0.12173	0.096158	-0.04553	0.015629	0.017428	-0.07477	0.115592	-0.02284	-0.08737	0.239399	-0.13152	-0.16045
59	intake159	-0.10198	-0.06846	0.245623	0.003017	-0.13071	0.068582	-0.16226	0.224367	0.004388	-0.11121	0.020455	-0.06228	0.089654	-0.16016	0.023802	-0.01315	-0.02804	-0.07692	-0.03565	0.264999
60	intake160	-0.01603	0.077317	-0.16053	-0.12266	0.09223	0.288066	-0.34495	0.124084	-0.01593	0.122852	-0.13189	-0.02076	0.063621	-0.12591	0.071934	0.029936	-0.02533	0.056778	-0.11684	0.141501
61	intake161	-0.14254	0.148713	-0.1292	0.159037	-0.03377	-0.09115	-0.00464	-0.01563	-0.00859	-0.05684	0.250718	-0.13502	-0.0632	0.07989	-0.02236	0.130673	-0.07576	-0.17273	-0.03894	0.098128
62	intake162	0.015462	-0.14992	0.141283	-0.08415	-0.02166	0.002888	-0.04411	0.096211	0.089829	-0.19338	0.104094	-0.01374	-0.00453	0.107759	-0.0145	0.230211	-0.11978	-0.01289	0.059565	0.049473
63	intake163	-0.07045	0.112639	-0.1124	0.014731	-0.1241	0.093334	0.001112	0.073253	0.059363	0.012714	0.02526	-0.18173	0.035451	-0.04444	0.01423	0.042567	-0.08545	-0.04363	0.046357	0.044718
64	intake164	-0.04819	-0.11308	0.218015	-0.0967	-0.08994	0.063891	-0.01807	-0.05496	0.019456	0.028518	-0.09139	-0.03302	-0.05659	0.037071	0.183335	-0.10231	0.045573	-0.02169	0.06717	0.064864
65	intake165	-0.14007	0.177167	-0.04714	-0.05061	-0.01795	-0.2006	0.176564	-0.04114	-0.03985	0.040749	0.123817	0.149338	-0.16752	-0.05872	0.140463	-0.04618	0.072481	-0.10692	0.124083	-0.24866
66	intake166	0.235934	-0.18405	-0.13746	0.244596	-0.11163	-0.08321	0.198595	-0.322	0.220612	0.119336	-0.0652	-0.02124	0.081457	0.015153	-0.12085	0.033998	0.134179	-0.1751	-0.0784	0.214671
67	intake167	-0.05526	-0.05034	-0.03801	0.114323	-0.10024	0.001992	0.109264	-0.10675	-0.0268	0.218106	-0.11901	-0.0053	-0.00754	-0.13587	-0.12982	-0.03101	0.273383	-0.07747	-0.08396	-0.1425
68	intake168	0.103866	0.116815	-0.12387	0.077683	0.020458	-0.0871	0.07312	-0.20382	0.230442	-0.10294	0.055444	-0.14286	0.199103	-0.13465	-0.07247	0.030901	0.005113	0.045846	0.264	-0.27327
69	intake169	-0.25741	0.094501	0.079197	0.163411	-0.04704	-0.03755	-0.12466	0.084852	0.305394	-0.24187	-0.05311	0.130438	0.008499	-0.06602	0.050256	0.066536	0.002271	0.032274	-0.06836	-0.05451
70	intake170	-0.22249	0.159528	-0.1618	0.138076	-0.18544	0.099598	0.14556	-0.04247	-0.10886	0.075384	0.147131	-0.30491	0.063975	-0.01783	-0.12221	0.027585	0.013236	0.13335	-0.08289	0.031822
71	intake171	-0.18849	0.542567	-0.01383	-0.22544	0.098702	0.04865	-0.08666	-0.03696	0.227361	-0.25558	0.019937	0.028941	0.156811	-0.02051	-0.36381	0.145735	0.085692	-0.00152	0.048544	-0.07566
72	intake172	0.161719	-0.05019	0.093518	-0.05043	-0.0429	-0.09063	0.109081	-0.00594	0.007755	0.037706	-0.07842	0.112743	0.14643	-0.22665	0.146554	-0.02629	-0.13099	0.013827	0.070163	-0.01927
73	intake173	-0.17037	-0.12831	0.108005	0.032111	0.113462	-0.26171	0.218957	-0.00091	0.095499	-0.27524	0.253121	-0.06928	0.070085	-0.05114	-0.09121	-0.10566	-0.00257	0.118196	-0.15364	-0.12811
74	intake174	0.095621	-0.00319	0.077043	-0.03034	-0.10643	0.133226	-0.10352	0.088545	0.072216	-0.05089	0.03758	-0.13092	-0.07721	0.350012	-0.18071	-0.27385	0.246394	0.002757	0.027832	0.075292
75	intake175	-0.04794	-0.15608	0.14398	-0.06088	0.008742	0.009541	0.006213	-0.04268	-0.06203	0.072424	0.060812	0.022174	0.10102	-0.13677	0.056588	-0.15909	0.103008	0.041335	-0.03215	-0.04404
76	intake176	0.082801	0.120032	-0.16985	0.204131	6.65E-05	-0.15285	0.085877	0.072811	-0.16417	0.070951	-0.15038	0.098758	-0.00148	-0.10621	-0.00659	-0.01824	0.186798	-0.13522	0.026968	0.026945
77	intake177	-0.04849	0.075983	0.078083	-0.25166	0.160629	-0.01145	0.003244	-0.12424	-0.03424	0.131495	0.028735	0.088456	-0.09757	-0.03592	-0.08112	0.051662	-0.00617	0.00349	0.169088	0.039539
78	intake178	-0.35066	0.191324	0.054138	-0.13819	-0.15225	0.065083	0.181556	-0.04796	-0.1435	-0.02178	-0.00507	0.120759	-0.05644	0.078794	0.095784	-0.20274	0.026687	0.021349	-0.01415	0.016821
79	intake179	-0.06735	-0.03216	-0.04346	-0.28534	0.24712	-0.0683	-0.02022	0.121972	-0.08407	0.063745	-0.10914	0.032376	0.007212	0.063981	-0.18851	0.163045	-0.08967	0.10553	0.053846	0.034182
80	intake180	-0.07206	-0.16038	0.269869	-0.27079	0.152727	0.029632	-0.03806	0.05042	-0.07008	0.01913	0.061988	-0.17288	0.029113	0.048325	0.226575	-0.1771	0.195254	-0.0619	-0.06619	-0.07111
81	intake181	0.07092	-0.21377	0.116529	-0.16411	0.092183	-0.02134	0.00393	-0.09107	0.05831	0.167255	-0.17089	-0.15279	0.043129	0.050642	-0.31397	0.355637	-0.08836	0.144038	-0.13483	-0.08903
82	intake182	0.026602	-0.13247	0.180102	-0.29641	0.073327	0.082734	-0.26325	0.242754	0.299596	-0.24333	0.010311	0.100789	0.028535	0.021659	-0.07214	-0.02952	0.187369	-0.1029	0.024119	-0.15771
83	intake183	-0.27842	-0.00517	0.395021	-0.16774	-0.11374	0.205787	-0.15039	-0.03814	-0.18625	0.131172	-0.06566	0.130222	-0.08063	0.028562	-0.11129	0.013072	-0.00538	0.040196	0.003779	0.012347
84	intake184	-0.15067	-0.06222	0.215183	0.007306	0.102999	-0.2347	0.05943	0.125442	-0.11446	0.221023	-0.19073	0.147135	-0.18829	-0.02676	0.18792	0.053734	0.018997	0.08143	0.024353	-0.06952
85	intake185	-0.07926	0.322963	-0.16479	0.042789	0.143107	-0.28583	0.136674	0.093144	-0.01671	-0.01842	-0.05143	-0.1259	0.091469	-0.09879	0.010678	0.110084	0.015998	-0.27444	0.183745	0.104719
86	intake186	-0.03093	-0.00707	-0.00465	-0.06426	-0.06257	0.041124	0.103098	-0.03231	-0.09744	0.04307	-0.16993	0.240038	-0.02912	-0.06311	0.01063	-0.08195	0.058969	0.022082	0.009759	-0.24009
87	intake187	0.001918	-0.1724	0.230883	0.00061	-0.20874	0.208923	0.211998	-0.31874	0.131348	-0.01737	-0.09585	-0.00151	-0.01137	-0.02164	0.11946	0.04387	-0.00058	-0.07366	-0.03997	-0.13755
88	intake188	-0.01033	-0.06767	-0.01225	-0.22902	0.206072	-0.06345	-0.04485	0.284343	-0.19143	0.082092	-0.00317	-0.04276	0.020939	0.294279	-0.19027	-0.05942	0.020607	-0.11839	0.069989	0.104255
89	intake189	0.092877	0.024373	-0.02667	0.087023	-0.00376	-0.03755	-0.11728	0.01184	0.020389	-0.06052	0.128414	-0.10937	0.181667	-0.0381	0.255188	-0.25405	0.151871	-0.13682	-0.02635	0.029366
90	intake190	0.028481	-0.07981	-0.00733	0.043209	-0.02574	0.048011	-0.04196	0.05788	-0.01224	0.015606	-0.02393	0.015606	0.004325	0.008201	0.030018	-0.03182	0.005671	0.018249	-0.00187	-0.04428
91	intake191	-0.06248	0.30556	0.040688	-0.29581	0.137582	-0.14288	0.185812	-0.09054	-0.03012	0.020096	0.065435	-0.01901	-0.15321	0.002989	-0.00946	0.094096	-0.14325	0.062273	-0.19209	0.250018
92	intake192	0.10213	-0.19278	0.003161	0.212992	-0.12646	0.040887	0.037695	-0.03378	-0.07243	0.093925	-0.08499	0.003333	0.107954	-0.05068	0.127057	-0.19029	0.133321	-0.16388	0.081166	0.073676
93	intake193	-0.04659	0.094361	0.13037	-0.2829	0.219371	0.137277	-0.17833	0.029855	-0.23663	0.166271	-0.03315	-0.05021	0.053878	-0.04103	0.05944	0.036022	-0.19802	0.03319	0.032463	0.110991
94	intake194	-0.1496	-0.24817	0.079317	0.005597	0.087631	0.000946	-0.1088	0.121888	-0.06812	-0.02194	-0.06099	0.258757	-0.17369	-0.06701	-0.17162	0.145743	-0.11603	0.204273	-0.21928	0.333396
95	intake195	0.021303	0.081661	0.035617	-0.03663	-0.10381	0.132201	0.0173	0.111907	-0.11638	-0.05931	0.107618	-0.17799	3.88E-02	0.086987	0.048156	0.082362	-0.06704	0.105887	-0.23682	-0.02436
96	intake196	-0.03935	-0.11334	0.046698	-0.03195	-0.05505	0.150879	0.009819	-0.16557	0.158487	-0.16249	0.075774	-0.00914	0.101556	0.085148	-0.12481	0.047173	-0.07261	-0.04267	0.204807	-0.17387
97	intake197	0.384244	-0.15199	0.197449	-0.26627	0.0648	0.01719	-0.05082	0.186265	-0.17819	-0.06916	0.088547	0.117949	0.031783	0.008493	-0.18387	0.179541	-0.01842	-0.01277	0.074965	-0.13208
98	intake198	-0.01414	-0.29986	0.321514	-0.27145	0.268292	-0.17965	0.1671	-0.11906	-0.05983	-0.00068	-0.01068	0.027594	-0.19033	0.072082	-0.17766	0.019992	0.141499	-0.11943	0.107491	-0.06174
99	intake199	-0.09986	0.016387	-0.0647	-0.17664	0.16229	-0.0749	0.048474	0.179926	-0.10194	0.090601	-0.0344	-0.01872	0.086339	-0.08989	0.059616	-0.09219	0.107932	-0.0091	0.091655	-0.0876
100	intake1100	-0.03102	-0.0607	0.167243	0.091306	-0.064	-0.02482	0.00706	-0.15187	0.077767	0.140637	-0.09463	0.173062	-0.07762	0.104254	0.002652	0.065003	-0.15855	0.089218	0.04761	0.021937
101	intake1101	0.074037	0.051196	-0.08362	0.192919	-0.12441	-0.0648	0.030871	0.125626	-0.21738	0.118298	-0.0875	0.130342	-0.17884	0.097019	0.063863	0.039147	-0.01879	-0.18765	0.207005	-0.12619
102	intake1102	-0.033	-0.04504	0.083094	-0.16117	0.203233	-0.00088	-0.16498	-0.15617	0.096314	0.058555	-0.09031	0.188789	-0.01177	-0.05098	0.133875	-0.052	-0.13761	0.017786	-0.05232	-0.03703
103	intake1103	0.019558	0.057999	-0.08072	-0.19942	0.291667	-0.16698	0.112002	-0.0497	-0.26375	0.049137	0.100845	-0.23699	0.217487	0.003306	0.001145	0.035744	0.107621	0.001047	-0.04109	-0.03239
104	intake1104	0.256189	0.11949	-0.1153	-0.24087	0.152267	0.149065	-0.18357	0.084103	-0.02294	-0.02596	0.043609	-0.03582	-0.06097	-0.22307	0.232985	0.087224	-0.01225	-0.13903	0.134018	-0.10594
105	intake1105	0.058544	-0.01262	0.013621	0.032239	0.054688	-0.18148	0.239123	-0.15589	-0.03006	-0.00808	0.003835	-0.00504	-0.04223	0.0973	0.070274	-0.1605	0.138177	-0.05803	-0.11816	0.207815
106	intake1106	0.23815	-0.07613	-0.21668	0.195676	-0.01956	0.029557	-0.12077	0.22236	-0.1489	0.032082	-0.01377	-0.01594	0.038969	-0.04837	0.183496	-0.45758	0.15739	0.27952	0.073654	0.09507
107	intake1107	0.031072	-0.10534	-0.03585	0.265798	-0.06682	-0.08669	0.033685	-0.05223	-0.02551	-0.15301	0.255992	-0.02462	0.041539	-0.09096	-0.00078	0.044368	-0.00971	0.166965	-0.10315	0.162552
108	intake1108	-0.00953	-0.16926	0.042832	-0.07336	0.145885	-0.14932	0.250863	0.162932	-0.2636	-0.00998	-0.09098	0.050549	0.288509	-0.25187	0.010005	0.038286	-0.07957	-0.05113	0.07504	0.035779
109	intake1109	-0.03239	-0.03126	-0.10486	-0.05095	0.187225	-0.19327	0.103795	0.101734	-0.06522	-0.04985	0.112915	0.096149	-0.16323	0.156789	-0.06901	0.148998	-0.0186	0.019809	-0.25562	0.107836

Factor37	Factor38	Factor39	Factor40	Factor41	Factor42	Factor43	Factor44	Factor45	Factor46	Factor47	Factor48	Factor49	Factor50	Factor51	Factor52
0.078857	−0.06615	0.001858	0.050767	−0.2017	0.185083	0.060048	−0.08897	−0.017	−0.0957	−0.07654	−0.03181	0.225009	0.203506	−0.00531	−0.02705
0.06917	−0.08672	0.19448	0.074108	0.051565	0.052429	−0.13588	0.007585	0.026074	−0.14407	0.134004	−0.01111	0.047053	−0.02461	−0.00799	0.07602
−0.062	−0.11412	−0.00466	0.092745	−0.06574	0.132026	−0.0148	−0.13756	−0.11949	−0.06259	−0.01032	0.076407	0.052434	0.131233	−0.0724	−0.1206
−0.2048	−0.03516	0.120667	0.011374	−0.20132	0.151585	−0.09254	0.058296	0.103006	−0.06438	0.065606	−0.05001	0.073501	−0.06572	0.026828	−0.05215
0.025808	0.04618	0.139053	0.007699	−0.17793	0.147955	−0.02085	0.019668	0.005576	0.049312	0.003189	0.041176	−0.24682	0.133076	0.050997	0.004483
−0.18566	−0.05939	0.10603	−0.00149	−0.01234	0.011972	−0.02887	0.075474	0.071897	−0.07579	−0.07113	0.097755	0.141894	0.024985	0.143393	−0.01127
0.132372	0.055192	−0.05914	−0.11625	0.062536	0.087378	0.009944	−0.02313	0.029355	0.060263	−0.12807	−0.03531	−0.0821	0.078194	−0.16863	−0.13472
0.210088	−0.25905	0.117925	−0.24555	0.106214	0.036703	−0.03098	−0.04614	0.182149	0.027817	0.091982	−0.12963	0.01521	−0.02582	0.086547	−0.11963
−0.01734	−0.00977	−0.026	−0.06003	0.024954	−0.03419	−0.03549	0.041219	−0.08154	0.008631	−0.00775	0.120688	0.041127	0.104075	−0.02622	0.079559
−0.02658	−0.07083	0.146852	−0.04106	0.001547	−0.01889	0.060877	−0.07499	−0.04005	−0.09337	−0.02869	−0.11779	0.002035	−0.06741	0.035483	−0.0738
−0.03631	−0.04176	−0.04352	−0.02067	0.076392	−0.12571	0.228451	−0.16289	0.101081	−0.0457	−0.02366	0.119984	−0.10557	0.105827	0.042714	0.045706
0.026096	−0.12279	0.079419	−0.04365	−0.02445	0.142611	−0.14423	0.175352	−0.07375	−0.0374	−0.01025	−0.20667	−0.08113	0.014781	−0.05875	0.03387
0.070529	−0.0504	−0.07618	0.050942	0.075664	−0.03065	0.170724	−0.13587	0.099434	−0.08582	−0.05025	0.11973	−0.04236	−0.05759	−0.08846	−0.01141
0.008773	0.045179	0.106996	−0.02316	−0.02072	−0.01949	0.068392	−0.0575	−0.10688	−0.09347	0.015643	0.030828	−0.09429	0.180645	−0.03366	−0.05113
−0.11799	0.081893	−0.03987	−0.05521	−0.06724	−0.04546	0.248266	−0.03894	−0.10454	0.145502	0.207244	−0.04989	−0.05126	0.057043	−0.07652	−0.00239
0.103327	−0.13728	0.138894	−0.1644	−0.03282	0.023139	−0.09604	−0.02845	−0.06737	0.136223	−0.0506	0.033073	−0.06846	0.131481	0.116362	0.01465
0.058868	0.052925	0.038954	0.070239	−0.06849	0.011568	−0.07914	0.008872	−0.01534	−0.05453	−0.07877	0.094655	−0.02813	0.094863	−0.15046	−0.08332
−0.15925	0.014386	0.131816	−0.00814	−0.04191	0.035719	−0.14941	0.105071	0.004125	0.147242	−0.03619	−0.08162	0.018123	0.069256	−0.09709	0.064745
−0.09036	−0.07695	0.059554	0.111112	−0.00047	−0.24266	0.114399	0.052642	0.001394	−0.08619	−0.02548	−0.0282	0.051978	−0.04734	−0.07501	−0.03263
0.066333	−0.16206	0.109834	0.048498	−0.1387	−0.02329	0.078679	0.144898	−0.09425	−0.06182	0.068855	0.077367	0.009778	−0.09256	−0.00806	0.041579
0.018688	−0.03637	0.172119	−0.0692	−0.04639	0.060063	−0.0987	0.126108	0.028551	−0.00769	−0.10553	−0.03159	0.113612	−0.0327	−0.08463	0.016669
0.023241	0.064589	−0.13498	0.047022	0.136696	−0.20055	0.233902	−0.21067	0.0279	−0.02882	0.046734	−0.08452	0.091693	0.017691	0.024335	0.010464
0.15499	−0.28358	0.278194	−0.148	−0.15304	0.108941	0.01403	−0.05635	0.141795	−0.06341	−0.07848	−0.03704	−0.01026	−0.12794	−0.0105	0.04
0.075172	9.44E−05	−0.09991	0.134431	−0.14613	0.134943	−0.07458	−0.00267	0.039804	0.083304	−0.07694	−0.08302	0.132613	−0.18441	0.092625	−0.14227
0.093553	−0.22248	−0.06966	0.263309	−0.0519	−0.04173	0.079092	−0.06279	0.078562	−0.01553	0.131234	−0.12987	0.061546	−0.06284	−0.06719	0.024647
0.035897	−0.03724	−0.10613	0.162676	−0.02929	−0.07496	0.030013	−0.07158	0.032381	−0.03701	0.087936	−0.08174	−0.02227	−0.16154	0.084734	0.075649
−0.03691	0.114708	0.113302	0.077481	−0.113	−0.00739	−0.02267	0.065627	0.064382	0.103216	0.000464	−0.12306	0.110769	0.138769	−0.02068	0.18595
−0.16381	0.211153	0.046681	0.00078	−0.04243	0.035421	0.04692	−0.1166	0.016027	0.080749	0.01783	0.021209	0.023792	−0.02598	−0.13932	0.110057
−0.04537	0.139268	−0.26843	0.205015	0.02714	0.006581	−0.01454	0.029027	0.086545	−0.0045	−0.01779	0.046181	0.072245	−0.0121	−0.12723	−0.01437
0.06553	−0.03564	0.064041	0.032934	0.044491	−0.05546	0.076762	−0.24351	0.031499	−0.01155	−0.06303	−0.01573	0.069983	0.051262	0.003008	−0.01527
0.000667	−0.0172	0.10451	0.056748	0.047486	0.015656	0.006486	0.035123	0.012148	0.043594	−0.02173	0.00351	−0.07212	0.106251	−0.0511	0.049198
0.057869	0.141273	−0.21082	−0.03909	0.005985	0.125938	0.058288	0.005914	0.062588	0.093132	−0.18731	0.174253	0.002397	−0.13546	−0.03579	0.099847
−0.07264	0.014961	0.032789	−0.03476	0.072128	−0.14626	−0.06353	0.152453	−0.02671	0.0856	−0.04863	−0.03722	0.080528	−0.06956	−0.18623	0.008612
0.018699	0.011724	0.086597	−0.03954	0.033405	−0.11937	−0.0695	0.042977	−0.01227	0.147283	−0.09314	0.147355	0.113003	−0.03443	−0.03833	0.08771
−0.01482	0.028381	0.029072	0.03051	−0.05075	0.035518	−0.14456	0.026846	−0.01526	−0.00532	−0.16325	0.023248	0.118067	−0.00507	0.199217	0.004624
0.00598	−0.10708	−0.14708	0.06012	−0.06224	0.145186	−0.00235	0.02203	−0.14685	−0.12307	0.10969	0.000192	−0.12359	0.091431	−0.0931	0.102478
0.032148	1.56E−06	−0.03127	−0.08414	0.09628	−0.04171	−0.00645	0.130953	−0.104	−0.15525	0.091633	0.163527	−0.10685	−0.05607	−0.04683	0.003955
−0.14394	0.177453	−0.08119	−0.05762	0.049332	0.041566	−0.16458	0.081514	0.060513	−0.0645	0.112637	−0.05844	0.115745	0.028478	−0.01081	−0.1285
−0.11821	0.117139	0.01294	−0.15956	−0.02811	−0.05721	0.101724	−0.03308	0.087449	0.015908	0.019408	0.027197	0.063342	0.110918	−0.21206	0.043625
−0.02094	0.088612	0.034088	−0.03911	−0.15397	0.159997	−0.00344	0.091736	−0.23851	−0.01314	0.065844	0.028661	0.074435	−0.11698	−0.13071	−0.11844
0.063832	−0.0683	0.083693	−0.07219	0.005912	0.009391	0.077125	−0.02847	0.024946	−0.0551	−0.20203	−0.07002	0.163874	−0.16887	−0.14633	0.106963
0.003763	0.033675	−0.00472	−0.0699	0.144726	0.096894	−0.06827	−0.02084	−0.03994	−0.07568	0.015955	0.048623	−0.12735	0.037918	−0.16985	0.10788
0.080653	−0.14734	0.19835	−0.18371	−0.11336	0.041788	0.078744	−0.01911	−0.02718	0.11762	0.109176	0.035987	−0.05754	−0.07903	0.126898	0.013425
−0.11518	0.152496	−0.06548	0.001538	−0.0528	0.168559	−0.00875	−0.01036	−0.10941	0.023416	0.133465	−0.00487	0.108238	−0.02287	0.04368	−0.09353
0.050706	0.096611	−0.10542	0.087574	−0.1724	0.158716	−0.03614	−0.05382	−0.01481	0.064039	−0.12082	0.107853	0.080862	−0.15867	−0.02865	−0.00852
0.173363	−0.21874	0.012376	−0.10002	0.042308	0.071734	−0.07946	0.04023	−0.12529	0.098965	−0.01453	0.136296	−0.0087	0.227111	−0.11438	−0.02519
−0.01749	0.028316	0.034943	0.039569	−0.02344	0.064276	0.054534	0.022467	−0.04968	0.067765	−0.0888	−0.03797	−0.03515	0.05889	−0.13056	0.032137
−0.06216	0.064428	−0.20047	0.08757	−0.11695	0.118751	0.040856	0.104841	−0.13828	0.087541	########	−0.01377	0.093809	−0.1227	0.017863	−0.01273
0.097701	−0.00976	−0.18717	0.152947	0.024294	0.053208	−0.12694	−0.02525	0.243111	−0.05908	−0.06188	−0.0491	−0.0231	0.10145	0.041988	−0.00301
0.079722	−0.12556	0.026065	0.051672	0.125412	−0.095	−0.06933	−0.00213	−0.11671	0.094834	−0.05439	−0.01648	−0.09036	−0.07231	−0.07136	−0.06188
−0.06951	0.052239	−0.06972	0.027862	0.126165	0.127487	−0.2425	0.07917	0.064024	−0.0472	−0.00122	0.153752	−0.10483	−0.16115	0.059425	0.039165
−0.04135	−0.01945	0.102734	0.002251	−0.072	−0.0521	0.062969	−0.11597	−0.09372	0.00361	−0.21551	−0.099	−0.01053	0.008282	−0.00831	0.048167
0.042132	0.009197	−0.01434	0.068954	−0.0006	−0.05833	−0.02569	0.043357	−0.00745	−0.03917	0.02219	0.021897	0.113492	−0.0175	0.035665	−0.02159
0.143758	−0.11284	0.136802	−0.1679	0.248912	0.111535	0.001366	−0.03244	−0.03084	0.120478	−0.04054	0.072625	0.034937	−0.13041	0.104791	0.085353
0.058084	−0.06811	0.052977	−0.13873	0.114986	−0.12143	0.068741	−0.13159	0.009648	−0.11307	0.037742	−0.07037	0.063525	0.112991	−0.14993	0.174109
0.268558	−0.03427	0.18956	−0.13243	0.181757	−0.03291	−0.03915	0.072501	−0.05938	0.018594	−0.04494	0.085192	−0.07473	−0.06832	−0.02846	−0.02195
0.102055	−0.04098	0.065264	−0.07217	−0.0255	0.160124	−0.16636	−0.01729	0.058844	0.025402	0.143757	−0.15512	0.050253	−0.02343	0.038482	0.109176
0.102681	−0.02495	−0.00092	−0.07855	0.125629	0.164015	−0.18051	0.143234	0.09016	0.016382	0.188998	0.063742	0.05333	0.072051	0.069982	−0.01619
−0.2346	0.030624	−0.06726	−0.15823	0.192854	0.030458	−0.10589	−0.02847	−0.10122	0.032268	0.169336	−0.16936	0.032783	−0.06651	−0.03452	−0.01811
0.028119	−0.06742	−0.02523	0.14738	−0.12932	−0.03524	0.098177	0.008711	−0.12656	0.188129	0.054566	0.052919	0.009588	−0.15902	−0.08741	0.226384
0.063881	0.165305	−0.12066	−0.10131	0.150062	−0.08618	−0.03056	−0.09206	0.144496	0.052684	0.040132	−0.14986	−0.22676	0.123038	0.034281	−0.12039
−0.03782	0.083771	−0.132	0.022547	−0.03356	0.042185	0.047075	−0.02134	0.024361	−0.0127	−0.10812	0.05473	0.025637	0.076175	0.127544	−0.14298
−0.01587	0.000427	−0.0511	0.002256	−0.20423	0.07358	−0.02489	−0.0363	−0.12082	−0.04918	0.06057	−0.07671	0.044787	−0.01376	−0.02993	−0.00869
−0.18277	−0.08935	0.158714	0.156362	−0.33887	0.048545	0.1671	−0.11104	0.093888	−0.09344	0.0242	0.097057	−0.06364	−0.02726	0.015922	−0.05742
0.041712	−0.02351	0.106796	−0.04209	0.109805	−0.11151	0.035118	−0.12459	0.172094	−0.03589	0.067867	0.109234	−0.04259	0.115845	−0.04426	0.082453
−0.1736	0.157901	−0.04882	0.074177	−0.01687	0.068235	0.037578	−0.01631	−0.13583	−0.00594	−0.01512	0.068543	−0.0592	0.163706	0.064854	0.160172
0.256754	−0.30483	−0.12879	0.144531	−0.00719	−0.1913	0.235808	0.122803	−0.15055	0.084568	−0.10866	0.008826	−0.12394	0.080761	0.100584	−0.05831
0.289948	−0.0384	−0.18857	0.097164	−0.07078	−0.03355	−0.03461	−0.02106	0.060558	−0.12978	0.28786	0.062998	0.017914	−0.16419	0.09706	0.055959
−0.0538	0.033138	0.132698	0.076904	−0.08917	0.025925	0.179401	−0.10808	0.043814	−0.15209	−0.06817	0.004996	−0.096	−0.01712	0.171835	−0.14544
−0.11664	−0.06433	−0.06567	0.050397	0.241329	−0.10561	−0.03463	0.083938	−0.16829	−0.05265	0.022906	−0.04836	−0.0866	−0.16292	−0.12316	−0.05501
−0.04354	−0.07109	−0.19769	0.09987	−0.02433	−0.0284	0.013055	−0.01796	0.091895	0.137598	−0.20149	0.050823	−0.08539	0.101009	−0.04147	−0.05337
−0.06108	−0.1418	0.287676	−0.1547	0.109314	0.150356	−0.02969	0.004652	−0.05534	0.153143	−0.07823	0.008714	−0.07841	0.099171	0.003456	−0.02177
0.123216	0.044754	−0.05593	−0.01168	−0.0292	−0.00371	−0.01375	−0.00039	−0.00114	−0.03234	−0.05525	0.099129	−0.04613	0.145431	0.095397	0.150981
−0.05666	0.180951	0.019463	0.016748	0.09207	−0.14561	0.061254	0.066372	0.088336	0.050721	0.117322	0.002856	−0.09662	0.088099	0.007192	−0.0917
0.106702	0.109695	−0.16016	0.048612	−0.0796	−0.01499	0.14206	−0.14674	0.174901	−0.21534	0.116185	−0.09513	0.132849	0.051164	−0.03165	0.029272
−0.10987	0.135839	−0.0672	0.103927	−0.17858	−0.01677	−0.04896	0.080078	−0.09024	−0.11727	0.135539	−0.0284	−0.18171	0.027981	0.09535	0.138336
−0.13697	0.083292	0.109471	−0.22776	0.128173	−0.10998	−0.08127	−0.02809	−0.2588	0.036228	−0.0607	0.224591	−0.12199	−0.0116	0.169626	−0.02866
0.191227	−0.02959	0.033181	−0.05127	−0.02415	0.032534	−0.09179	0.20069	−0.16402	0.094688	−0.04734	−0.01321	0.09456	0.036708	−0.06648	0.043253
−0.16184	0.08124	−0.01398	−0.08729	0.081325	−0.08422	−0.05984	0.170225	0.054529	−0.10845	−0.0464	0.191106	−0.03423	0.105674	−0.19639	0.084888
−0.06885	−0.0368	0.026607	−0.05253	0.21468	−0.1388	−0.07533	0.00575	−0.10536	0.110227	−0.00579	−0.07103	0.136032	0.020782	0.153273	−0.07498
0.08666	−0.08622	0.317828	−0.19603	0.127851	−0.15893	0.067683	0.033096	−0.02816	0.042687	0.170017	−0.09078	0.071849	−0.03855	−0.01048	−0.26592
0.157651	−0.03302	0.161191	−0.05685	−0.02798	−0.19075	−0.00057	0.123097	0.030876	0.021342	−0.03989	−0.06016	0.004008	0.080989	−0.16696	0.115861
0.041127	0.002171	−0.04384	0.200002	−0.01566	0.081657	−0.09364	0.012002	0.001478	0.147957	−0.07697	−0.04131	−0.00689	0.067706	0.157997	0.044542
0.025253	0.049818	−0.14159	0.02236	0.030822	0.087587	−0.14584	−0.05712	0.030876	−0.02562	−0.03126	−0.18755	0.058782	0.108563	−0.1378	−0.02854
−0.03718	0.062651	0.151241	−0.1883	0.126123	−0.18456	0.148095	0.168673	0.02041	−0.164	−0.03558	0.122911	0.298605	−0.05061	0.213414	−0.03212
0.122294	0.115364	0.066543	−0.08968	−0.14835	0.145763	−0.05642	−0.0773	−0.05329	0.030274	−0.00647	0.050016	0.024694	−0.12625	0.191121	−0.35185
0.161872	0.077878	−0.00049	−0.13173	−0.04392	0.024119	0.035663	0.033915	0.065654	−0.20617	−0.04726	0.209654	−0.15549	0.019634	−0.15104	−0.1435
−0.01189	−0.01511	−0.00961	0.077664	−0.12908	0.270915	−0.14753	0.089419	−0.05148	0.021801	−0.04474	0.004545	−0.14376	0.130464	0.183966	−0.0585
−0.05351	−0.19377	0.057126	0.085215	−0.01685	−0.0491	−0.06348	−0.08343	0.113465	0.040781	0.020477	0.156221	−0.04709	0.012581	−0.03837	0.023791
0.020342	0.029348	0.002887	−0.03783	0.039559	−0.05815	0.016238	−0.01624	−0.0651	0.031141	−0.06163	−0.0102	0.014246	−0.02128	0.03867	0.004319
−0.10281	−0.06603	0.098758	0.010568	−0.04846	0.124299	−0.00939	0.067317	0.136293	−0.02838	0.145445	0.008384	−0.13545	0.090283	−0.15391	−0.04791
0.052611	−0.07132	−0.10244	0.034404	−0.044	0.089288	0.035572	−0.02397	0.210732	−0.02993	0.029341	−0.06325	0.101922	0.000208	0.001834	0.026956
−0.00689	−0.06441	0.072364	−0.02919	0.011698	−0.13911	−0.04479	0.011442	0.071625	−0.25707	0.092475	0.106619	−0.1371	0.098183	0.000963	0.0338
−0.25769	0.087428	−0.00593	−0.24924	0.224224	−0.05967	0.216118	−0.2743	0.193457	−0.02505	−0.02601	0.083239	−0.02857	−0.15578	0.101152	0.069796
−0.01871	0.3136	−0.1866	0.095326	0.023382	−0.09427	0.041441	0.12484	−0.03815	−0.00435	−0.07235	0.039396	−0.14905	−0.03587	0.030259	0.11628
−0.11992	0.09866	−0.05011	−0.06424	0.09996	0.124648	0.022014	0.078621	−0.06694	−0.17136	0.259191	−0.21964	0.134341	0.146513	0.118313	−0.13217
0.051158	−0.00324	0.025631	0.032522	0.036403	0.109941	−0.13671	0.02979	−0.12087	−0.11124	0.083186	−0.06314	0.165172	−0.04902	−0.07239	0.065131
−0.0322	−0.06529	0.007764	0.094727	−0.13473	0.140876	−0.27394	0.186983	0.069483	−0.10583	0.015337	0.057936	−0.03507	−0.13369	0.010051	0.102875
−0.03327	0.081946	−0.09069	0.163283	−0.06936	0.006191	−0.04349	0.074038	−0.02739	−0.16872	0.080572	−0.19311	−0.14658	−0.02429	0.119585	−0.05499
−0.0554	0.084685	−0.29349	0.10239	0.03817	−0.07798	−0.1725	0.272455	−0.10663	0.160885	−0.1955	−0.01798	0.101449	0.01415	0.052965	−0.06948
−0.11915	0.172635	0.017848	−0.03044	−0.00258	−0.21342	0.059826	0.04205	0.150868	0.111341	−0.13238	−0.00301	−0.0501	−0.13715	0.093425	−0.17462
0.174017	−0.10496	−0.07932	0.003591	0.188829	−0.41399	0.211075	0.175976	−0.12791	−0.18986	0.151868	−0.16525	0.049453	0.055758	0.134806	−0.01114
−0.04415	0.091444	−0.04039	−0.15636	−0.00371	0.103387	−0.07331	6.10E−05	−0.03202	0.084959	0.010747	−0.14348	−0.02191	−0.00043	0.015875	0.112362
0.026254	−0.09831	−0.00575	−0.11292	0.076489	0.088792	0.085039	0.047259	−0.05743	0.131734	−0.24649	0.095333	−0.09984	0.067683	0.115435	0.03277
−0.1274	−0.01081	0.137908	−0.18494	0.195621	−0.11281	0.128162	0.009035	0.027866	0.023325	−0.10408	−0.13704	0.14869	−0.0803	−0.00248	0.099337
−0.16117	−0.07726	0.034518	−0.01392	0.12746	−0.06025	−0.12302	0.115238	0.078515	−0.14527	−0.08913	0.114581	−0.09387	0.01856	0.091215	−0.22859
−0.05322	−0.0639	0.015081	0.054153	−0.1307	−0.08417	−0.04701	0.22375	0.125293	0.139221	0.038816	−0.02379	−0.07591	0.140393	0.036282	−0.08882
0.002712	−0.00205	−0.09838	0.310967	−0.05492	−0.2279	0.258271	−0.1918	−0.15305	0.342833	−0.00389	0.101493	0.046402	0.050925	−0.00677	−0.1682
0.085533	−0.14196	−0.14785	0.220682	0.034161	0.104619	−0.20728	0.010889	0.062483	−0.0627	−0.02801	0.230337	0.090087	0.024062	0.000588	−0.12566

Scores

No.	和名	英名	Factor1	Factor2	Factor3	Factor4	Factor5	Factor6	Factor7	Factor8	Factor9	Factor10	Factor11	Factor12	Factor13	Factor14	Factor15	Factor16	Factor17	Factor18	Factor19	Factor20	Factor21	Factor22	Factor23	Factor24	Factor25	Factor26
	全国	All Japan	-0.21098	-0.67678	-0.04726	0.073236	0.453313	0.14313	0.282368	0.269588	0.163602	0.185976	0.160958	0.340466	0.40971	0.362418	0.714238	0.903378	1.706086	1.443713	2.107047	2.117731	2.643731	2.670214	2.286933	1.590914	1.027947	1.159244
1	札幌市	Sapporo-shi	3.619055	-3.29274	-2.96245	2.609029	2.790251	-0.83559	0.34045	1.047592	-4.71462	1.454342	0.257051	-0.87044	-2.02277	0.59796	1.007841	-1.28768	1.212378	-0.47348	-0.06958	0.735814	0.204683	-0.56493	0.230033	0.388029	-1.47703	-0.97676
2	青森市	Aomori-shi	11.35639	-0.84286	-3.82804	-0.73989	-1.44821	-1.94883	-1.5247	3.38589	-2.48219	0.616346	-2.96086	1.000558	-0.47591	0.177934	-0.46977	0.252832	-0.51205	-0.33107	1.198541	-0.18768	-1.39944	-0.1944	0.840367	-0.03279	0.167103	-0.86744
3	盛岡市	Morioka-shi	6.672104	1.31627	2.60607	-1.3364	2.0743	0.169041	2.261267	2.476585	0.389921	0.579206	-0.58772	1.874998	1.228189	0.505825	-0.50677	-0.34186	-0.84229	0.321153	-0.63944	1.742617	0.571003	-0.98932	0.19905	-2.29273	-0.46878	1.253974
4	仙台市	Sendai-shi	4.592346	-1.66187	2.115666	-0.54924	1.968129	0.389693	0.694257	-0.67544	-0.40211	0.468506	1.727302	-0.24348	-0.88688	0.468391	-0.73036	-0.01029	-0.55087	-0.38108	-1.72073	0.440287	-0.45904	-0.56943	0.925202	0.762444	0.461728	-0.31577
5	秋田市	Akita-shi	9.325999	1.11752	-1.36108	-0.75505	0.373552	-0.27353	0.286654	-1.6711	-0.76465	2.949188	1.504421	0.682732	-1.13242	-0.52195	0.017817	0.194955	1.264653	0.389622	-0.71429	-0.60539	-0.20692	-0.03555	-0.53009	-1.01882	1.403752	2.895439
6	山形市	Yamagata-shi	7.70434	5.387739	6.88186	1.672768	2.194653	-0.37417	0.172934	-0.62029	-1.4868	-0.43377	0.169703	-0.7468	0.080491	-1.17008	0.585942	0.389977	-1.13416	0.220069	1.125665	-0.19849	-0.48703	0.44795	-0.09946	0.866973	-1.15709	-0.0012
7	福島市	Fukushima-shi	4.44791	1.607546	1.180907	-3.81035	-0.37922	-3.56046	-0.42533	-1.32396	0.089418	0.053027	-0.18579	0.468373	0.53388	0.967886	0.741486	-0.14683	1.001649	0.337429	-0.77807	-0.53381	-0.459	0.178597	-0.68515	1.730397	1.586992	-1.88712
8	水戸市	Mito-shi	-0.5483	-3.572	-1.51247	-1.16907	3.623693	0.972353	1.912044	-0.7601	0.437162	-1.73952	-0.35185	-0.06622	-0.47027	-0.20636	2.370683	0.666739	-0.12522	-0.24602	-0.43918	-0.24419	0.814668	-1.42332	0.061691	0.128043	0.153248	1.098034
9	宇都宮市	Utsunomiya-sh	-0.05849	-2.17732	-1.29993	-3.56941	-1.55457	-3.22261	-0.75547	-0.97294	-0.82859	-2.87437	-1.07578	-0.14711	-1.07361	-1.31414	-0.29998	-1.33917	0.000942	2.131714	0.126166	0.451191	0.527894	-1.34726	0.45409	0.072471	-0.13754	-1.07683
10	前橋市	Maebashi-shi	-1.48811	-2.42461	0.217974	-2.94853	1.169668	-1.4745	2.132145	0.805567	1.144434	-1.70874	-0.55789	-0.16977	1.544751	0.578849	-0.21278	-0.21517	0.365263	-0.13262	-0.57513	-1.28414	-0.51325	0.897934	0.711176	-1.03093	-0.95558	0.567453
11	さいたま市	Saitama-shi	-0.99408	-4.5333	1.733753	1.204974	-0.77256	-1.20181	-0.19903	-0.90152	-0.88537	-0.64394	1.483411	1.229172	2.212843	0.083753	-0.36976	0.687757	2.149367	0.539764	-0.18408	0.088842	-0.12934	-1.30139	-0.65091	-1.10051	-0.34839	-0.28969
12	千葉市	Chiba-shi	1.642702	-5.23078	0.027946	1.655038	0.70053	2.853796	0.931978	-0.63182	1.553187	0.243068	1.134199	0.674555	-1.94477	-1.44328	-0.28445	-0.53367	-1.22467	1.792582	2.034591	-1.41698	-0.26159	-0.42195	-0.38326	-0.27218	0.305508	0.46744
13	東京都区部	Ku-areas of T	-2.02684	-6.12983	0.613402	-0.59197	-0.49644	-0.28227	1.015052	-0.15432	-0.26685	-0.63227	1.380277	0.24027	0.835518	1.669725	0.999025	-0.48745	-0.73565	-0.73625	-0.13408	-0.38561	0.691335	-0.16168	1.151078	0.932664	-0.02505	-1.13014
14	横浜市	Yokohama-shi	-0.63933	-7.01889	1.40199	0.79037	-2.90897	0.278638	0.647005	1.268403	-0.06736	-0.81097	-0.16319	-0.19464	0.457731	1.056682	1.263701	0.80449	0.229598	0.014598	0.380344	-0.68428	-0.35643	-1.89819	-0.71466	0.237452	0.394889	0.64037
15	新潟市	Niigata-shi	8.938286	-1.61605	-1.06959	0.193473	-1.43888	2.343814	2.397455	1.826114	1.116156	-3.39959	0.810234	-0.99283	-0.21781	0.791997	-1.15563	0.387815	-0.18347	0.062203	-1.28983	-0.46624	1.25676	2.665434	-1.92098	1.102665	-1.04414	0.555773
16	富山市	Toyama-shi	2.836594	-0.16306	-0.36844	-1.50384	-0.96656	-0.11718	-3.07978	-0.35354	1.919891	-1.33965	1.323191	-0.62595	0.271891	-2.32935	1.35705	1.301437	-1.09779	-1.31582	-0.74768	1.551575	-1.08538	0.025159	1.219782	-0.94774	0.471315	-1.55386
17	金沢市	Kanazawa-shi	-0.27801	0.196139	-0.2049	0.176312	1.518636	1.402161	0.08002	1.594423	1.371509	-2.26268	-0.83921	-1.74358	1.801703	0.931705	-0.7998	-0.0892	0.668454	-0.2881	1.046502	1.065007	-1.16295	-0.02238	-0.86543	-0.41592	1.349065	-0.04576
18	福井市	Fukui-shi	-1.26493	3.205443	-0.04361	0.079538	2.758771	-1.94249	-1.48056	1.352752	2.550431	-0.44808	2.039156	-1.24099	-0.53927	-0.98413	1.022218	-1.11676	0.863173	0.699649	0.819127	1.603312	-1.25233	0.002467	-2.04184	-0.0241	-0.43151	-0.91821
19	甲府市	Kofu-shi	1.014598	-3.30724	0.397431	1.491813	2.801623	-0.38756	1.263474	-2.16664	2.485768	2.581563	-2.04963	-1.24777	0.645037	0.61145	1.305932	0.284113	-0.39514	-1.0486	-0.0811	-0.36995	0.091295	0.480443	0.550019	-0.22586	0.055313	-1.81922
20	長野市	Nagano-shi	2.386468	0.289742	2.506415	-2.67574	0.100471	-1.75036	0.408087	-3.2888	-0.3038	1.470484	-0.53134	-0.25604	0.843373	-0.82858	-0.35331	-0.45801	0.54005	-1.67903	1.250631	-1.21979	0.907145	0.480524	-0.79651	1.240944	-0.55162	-0.09252
21	岐阜市	Gifu-shi	-2.55375	0.389831	-1.18212	-1.00013	1.241985	-1.98252	-1.54321	0.765945	1.4241	1.062162	1.064092	0.652785	-1.20531	-1.20145	0.502256	0.425969	1.064584	-0.86401	-1.07355	-0.18036	-0.99179	1.118482	-0.5206	0.096658	0.049393	1.578574
22	静岡市	Shizuoka-shi	-0.15556	-4.254	0.107129	2.194183	0.736199	1.248816	-2.28935	-3.72323	2.959507	-0.8015	-2.59961	0.661726	-2.53546	1.053943	-1.29851	0.484335	0.755819	0.586519	-1.28057	0.738722	-0.36452	-0.33251	-0.48569	0.347146	-0.23554	0.723371
23	名古屋市	Nagoya-shi	-2.2856	-2.3681	-0.4978	-1.16025	-0.62839	-0.28532	-2.48115	-2.50637	-1.91992	-2.45301	0.265823	1.216939	0.400014	-1.47625	-0.6412	0.256546	0.098014	0.276519	0.68252	-0.37439	-1.19336	1.764207	0.155359	-0.44387	-1.77134	0.759505
24	津市	Tsu-shi	-2.40142	1.869195	0.751809	0.512897	2.236339	1.261532	0.419061	1.908747	1.826388	-1.42542	-1.78763	0.477134	0.348487	0.014887	0.389267	0.287096	0.689735	0.194132	0.810918	-1.3685	-1.98163	-0.10532	1.202202	0.946381	-0.32558	0.307608
25	大津市	Otsu-shi	-4.51902	-0.32665	1.513911	-0.99135	-0.73608	0.55907	-0.18574	3.48386	0.577673	-0.71401	-0.75316	-1.25748	-1.86191	-2.11374	-0.06037	-1.2365	-0.51792	-0.63501	-0.72309	-0.46716	0.737162	0.594332	0.874686	0.987804	0.784378	1.281144
26	京都市	Kyoto-shi	-1.80188	-0.87944	3.52853	0.627942	-1.86609	3.10763	-2.24293	0.181169	-1.74047	0.352388	0.305966	-0.16824	-0.4186	0.645873	0.835955	-0.62321	-0.33136	-0.0213	-0.8385	0.180565	1.03576	-0.06956	0.160052	0.665503	-0.62518	0.017597
27	大阪市	Osaka-shi	-4.26218	-0.71034	0.610485	0.464992	0.27414	1.836389	-0.92233	0.750947	-3.1884	-0.36725	-0.0084	0.18541	-0.21157	-0.53822	-0.52967	-0.71953	-0.87728	-0.97123	-1.23165	-0.07874	-0.94083	0.301764	-0.16504	0.98797	1.644129	1.423387
28	神戸市	Kobe-shi	-4.72229	0.108554	0.919488	-1.92021	-0.20776	2.22174	-0.21665	1.581401	-0.46525	1.473943	0.662381	-0.51489	-0.20507	0.505047	1.74464	0.21445	0.649158	0.366894	-0.41517	-0.07799	0.451106	0.110599	-0.11364	-2.1651	-1.30774	-0.24255
29	奈良市	Nara-shi	-1.42644	0.54441	1.176982	-0.77914	-3.38152	0.581792	-2.75735	2.623983	2.124724	2.846407	-1.0495	-0.34577	-0.84445	0.709579	-0.05232	0.34781	0.679098	-0.40777	0.760079	0.024799	0.105895	-0.82782	-0.67499	0.358622	-0.47632	1.451153
30	和歌山市	Wakayama-shi	-3.76415	0.930059	-1.31926	0.066557	2.268001	4.301324	-0.44194	0.751764	-1.01065	0.512003	-1.47591	2.974178	-1.36213	-1.04054	0.964286	0.449852	-0.79791	0.049423	-0.18558	-0.17677	0.939837	2.065496	-1.71748	-0.87379	0.399062	-2.73048
31	鳥取市	Tottori-shi	2.293442	3.2617	-4.91539	0.760101	2.037574	1.943184	-4.56146	0.340579	-0.59237	-2.00254	2.18013	-1.22965	0.589535	1.105569	-0.21132	0.953148	-0.34951	-0.94021	0.829964	-1.34502	1.448302	-1.357	0.560633	-0.10644	-0.25317	0.770922
32	松江市	Matsue-shi	2.54514	4.623437	-0.08326	2.564117	-0.16226	-0.01578	-3.38479	-0.80921	1.166223	-0.43352	-0.65473	0.517559	3.165342	-0.87503	0.076346	-1.39191	0.07121	1.0703	-1.51296	-1.23256	2.198416	-0.54635	0.667133	-1.22064	0.689539	-0.55786
33	岡山市	Okayama-shi	-4.18937	1.094026	-1.37857	-1.03277	1.992622	0.521619	-0.16781	-0.94495	-2.06585	1.641012	-0.36052	-0.63492	2.086744	-0.37975	-0.50552	-0.03782	0.239242	0.056643	1.406503	-0.54809	0.064738	-0.39723	-1.3705	0.285572	0.307509	-0.1139
34	広島市	Hiroshima-shi	-1.5519	-0.04676	-1.41146	-2.02345	-1.72575	3.080765	0.592866	-0.07194	0.24258	3.274404	0.866496	-0.3702	0.731912	-1.39206	-1.93894	1.085311	0.087622	0.583657	-1.0085	0.469376	-0.16358	-0.5613	0.950125	1.04602	-1.7522	-1.10429
35	山口市	Yamaguchi-shi	-1.55354	3.704595	-1.80687	-0.00233	0.207356	1.932872	2.84965	1.759347	0.086812	-0.45339	0.513395	1.682667	1.393881	-0.31362	0.385179	0.312585	-0.54694	0.934545	-0.55491	0.219722	-0.17101	-1.65076	-0.67615	1.339304	0.071537	-0.50213
36	徳島市	Tokushima-shi	-1.67531	1.747877	0.354781	-0.46187	0.778489	1.145098	0.259706	-1.17091	-0.77626	-0.16975	0.82484	-1.17898	-0.94908	2.957847	-0.41378	-1.00246	0.536335	0.36263	-0.09615	-0.30448	-1.226	1.646972	0.80264	-2.50067	0.601218	0.094165
37	高松市	Takamatsu-sh	-3.18071	2.544832	0.05317	-2.24016	1.623033	4.58449	1.32161	-0.81918	-1.33009	1.377537	-1.34776	-2.15151	0.360967	-0.18834	-1.25156	0.310142	-0.54841	0.986908	0.186942	0.077397	-0.42636	-0.97782	0.539488	-0.38547	0.384039	0.88716
38	松山市	Matsuyama-sh	-3.64014	3.11191	-1.12695	-1.22135	-0.3408	0.366856	-0.48393	-1.69625	-2.17132	-0.68718	-1.43893	0.398041	0.96721	1.288639	-0.1522	0.013685	-0.50463	-0.98257	-0.28433	1.380031	1.05631	0.055012	-1.47298	0.885322	0.03956	1.049103
39	高知市	Kochi-shi	-1.21932	6.633028	2.598967	-0.47252	0.196924	-0.02381	0.240485	0.366521	1.363824	-1.10729	1.128718	0.586508	-3.34865	1.826949	-2.09979	0.648455	0.325657	-0.62927	0.765396	-0.56232	1.206774	-1.1842	0.357657	-0.28621	0.21278	-2.466
40	福岡市	Fukuoka-shi	-3.06371	0.15702	0.455114	3.139284	-0.47823	-1.4969	1.395777	0.954714	-2.42622	-0.90849	-0.45956	-0.8928	0.559557	-1.30484	-1.72124	0.100291	1.89411	-0.46597	-0.77868	0.282327	-0.47637	0.341635	0.487956	-0.23366	-0.14491	-1.2964
41	佐賀市	Saga-shi	1.999929	2.543552	-2.88091	1.116561	-3.81354	-0.44912	0.963273	-0.80751	1.922966	0.742566	-1.87594	-2.36034	-0.14137	0.838026	0.908613	-0.11186	-0.24851	0.57693	-0.61326	0.204525	0.227197	0.046388	-0.80159	-0.00898	-0.52226	-0.66432
42	長崎市	Nagasaki-shi	-1.80939	1.323944	-2.26305	2.081729	0.164046	-0.41001	1.860911	0.324592	1.247633	1.394409	1.57861	0.232288	0.270606	-1.00482	-0.33546	-0.04986	0.775549	0.693321	-0.72419	-0.72951	-0.5961	0.045224	0.513684	1.996191	0.74027	-1.01425
43	熊本市	Kumamoto-shi	-1.98949	2.782011	-1.26182	0.781123	-2.80422	-2.45751	1.81947	0.340647	1.253407	0.254081	1.825547	1.277015	0.565433	0.742099	0.354682	-1.02517	-0.76842	-0.92444	0.966628	0.400697	-0.18706	-0.36031	-0.24143	0.60772	-0.60119	1.103065
44	大分市	Oita-shi	0.094815	5.647708	1.624941	3.032423	-1.86476	0.327138	1.468144	-2.1034	-0.90612	-1.77113	-1.56385	0.392937	-0.12504	-1.35139	1.207779	0.413827	0.543151	-0.58269	-0.19449	0.475306	-0.61868	-1.04272	0.456171	0.079147	-0.18043	1.497062
45	宮崎市	Miyazaki-shi	-3.00975	3.465034	-0.87275	-0.75125	-2.35459	-2.35321	2.314738	-1.30126	-0.01512	-0.70437	-0.28373	0.503543	-2.94508	-0.60605	0.544144	-0.10581	0.519909	-0.62114	0.528731	-0.23953	0.948891	-0.95079	0.471775	-1.23755	-0.05402	0.243162
46	鹿児島市	Kagoshima-shi	-2.39464	1.839979	-2.06372	1.321471	-1.25574	-1.64074	2.437896	-0.03974	0.230915	0.043384	0.069095	1.48244	0.594199	0.529996	-0.47095	0.267405	-0.1869	-1.21581	0.321319	-0.24162	-0.42887	1.240303	0.557323	-1.34333	0.333833	-0.24444
47	那覇市	Naha-shi	-6.05395	0.096738	0.801267	1.454176	1.94582	-10.061	-0.916	1.488294	-1.39144	1.041539	-0.20759	-0.82379	-0.40601	0.928842	-0.37271	1.620295	-2.2782	1.693677	-0.83086	-0.47812	0.527283	0.531366	-0.45273	-0.12079	-0.06532	0.851182
48	川崎市	Kawasaki-shi	-0.424	-6.39739	1.449084	0.700735	-1.37786	-0.17254	0.431863	-0.41303	-1.08809	-0.22193	0.827945	-0.55666	-0.10898	-0.81789	-1.40733	0.426178	-0.86588	-0.76028	1.087393	0.996263	0.483943	-0.73328	-1.01056	-0.9735	1.49534	-0.97387
49	相模原市	Sagamihara-sh	1.094441	-3.83187	1.074353	1.14242	-1.0522	-1.10339	-0.5475	1.818119	2.094172	1.664583	-0.5209	-0.34703	1.115857	-2.00257	-0.84648	-0.59703	0.134926	-0.79657	0.431884	-0.37089	1.387106	0.677924	-0.14297	-1.35133	0.087878	0.063226
50	浜松市	Hamamatsu-sh	-1.62285	-1.77412	-1.60566	0.601024	2.504718	-0.93481	-1.41084	-1.5169	2.854467	0.886262	0.001221	1.60826	0.658002	1.242865	-1.37093	-1.30698	-1.4461	-0.61661	0.321188	1.137448	-0.45179	-0.36359	0.673892	1.106843	-0.90848	0.762725
51	堺市	Sakai-shi	-0.23736	0.162495	2.72412	0.524816	-3.17318	2.118813	-2.68943	0.910839	-0.84365	0.878592	0.419956	1.225376	0.489343	2.385676	0.870206	-0.14867	-0.04582	0.926947	0.12337	-0.29884	-1.57866	0.769388	0.199214	0.52182	0.300988	-0.61888
52	北九州市	Kitakyushu-sh	0.4522	1.533668	-2.06021	0.673164	-3.54643	1.066279	1.506642	-2.93403	-0.46332	0.467411	1.166885	-1.20807	0.270202	-0.17601	0.693599	-0.10799	-1.95063	0.755431	0.408184	0.28788	-0.95793	1.232563	0.275256	-0.69811	0.30207	0.00205

Factor27	Factor28	Factor29	Factor30	Factor31	Factor32	Factor33	Factor34	Factor35	Factor36	Factor37	Factor38	Factor39	Factor40	Factor41	Factor42	Factor43	Factor44	Factor45	Factor46	Factor47	Factor48	Factor49	Factor50	Factor51	Factor52
1.036234	0.402844	0.154534	-0.03118	0.206796	0.012433	-0.02234	0.300142	0.139297	0.165307	0.003216	-0.19226	0.069979	-0.14294	-0.05993	-0.07065	0.201779	0.236415	0.019638	-0.28057	0.050795	0.219073	0.122446	-0.26172	-0.05926	-0.14297
0.511653	1.353254	-0.69075	-0.25708	0.014497	0.229348	-0.08766	-0.7838	-0.25519	0.893675	0.03202	-0.1915	0.688483	-0.3959	0.405486	-0.26093	-0.30601	-0.00543	0.068337	0.423944	-0.56213	-0.21958	-0.35605	1.266914	0.118446	-0.52612
0.342927	-0.76891	-0.13371	-0.35255	1.058635	-0.13358	-0.16884	0.716671	-0.26485	-0.24654	0.352259	-0.14917	-0.54017	0.793941	-0.50083	0.442896	0.542847	-0.32698	0.574428	-0.62447	0.797432	-0.48968	-0.57703	-0.84751	0.005731	0.151352
0.440715	-0.48283	0.596485	-0.31791	-1.20053	-0.67791	0.942541	0.045135	-0.2203	-1.54628	0.593248	-0.40901	0.387186	-0.27268	0.26433	-0.30895	0.225317	0.034427	0.622737	0.541028	-0.53241	-0.67292	1.251918	0.552267	0.69061	0.414711
-0.57102	0.321769	0.028795	0.190456	0.08539	0.166524	-0.12909	-0.51579	1.02355	-0.78752	0.496897	-1.46244	0.118626	-1.29138	-0.10768	-1.24355	-0.8746	-0.09906	0.290319	-1.23676	0.254117	1.004427	-0.95355	-1.55858	0.677403	-0.62331
-0.83358	-0.62178	0.178062	-0.13146	0.869291	-0.34884	-1.18593	-0.65022	0.784597	0.758669	-1.23362	0.476062	-0.23289	0.600977	-0.6507	0.135491	-0.31777	-0.53773	-0.91918	0.700829	-0.72555	1.088565	0.536265	0.204483	-0.1757	0.305321
-0.23233	-0.6344	0.286927	0.415965	-1.22488	0.698626	-1.52252	0.803581	-0.13858	1.312373	-1.01646	1.08359	-0.54565	0.15082	0.92155	-0.01623	-0.36757	0.308093	0.62219	-0.1291	0.014534	-0.28656	-0.01335	-0.23663	-0.33105	-0.48531
-0.01457	1.268754	0.831331	1.141903	-1.05688	1.206252	-0.12779	-0.10766	-0.04672	-0.77884	0.66747	-0.29914	0.245121	0.115268	0.420104	0.204977	0.725775	0.208821	0.035852	-0.25909	-1.20886	-0.7798	-0.61539	0.667095	-0.93655	1.103006
-1.4548	0.791106	-0.84509	0.545368	0.914585	-0.44066	-0.98711	0.906835	-0.18144	-2.00441	0.322006	1.069998	-0.52738	0.0662	-0.17741	1.593167	-1.36367	1.043091	0.078211	-0.3284	0.291458	-0.89111	-0.03095	0.04555	-0.0138	-0.50143
-0.27351	-1.28444	-0.29445	0.649611	-0.84466	0.43768	0.512955	-0.86856	0.57027	0.1892	-0.48341	0.891169	0.096019	-0.16133	0.675085	-0.04363	-0.3599	0.833227	-0.82292	0.857139	1.253863	0.979106	0.402111	-0.21259	0.503884	-0.28209
2.116568	0.8227	-0.44259	-0.52097	-0.27424	0.937426	0.44391	0.749684	0.433373	-0.06917	-1.43707	0.83235	-0.34263	0.497569	-0.77393	-0.43368	0.535904	-0.92136	-0.76753	-0.58251	-1.13753	0.252758	-0.53901	0.15384	0.706094	-1.42015
0.710624	-1.77356	-1.80394	0.28251	-0.78809	-0.04076	0.566449	0.691097	0.29276	0.78721	0.227312	-0.24964	-0.01173	-0.477	-1.1096	0.832643	-0.4929	-0.00292	1.768744	-0.00036	0.323336	0.303132	-0.9193	0.262766	-0.94494	0.390085
0.307556	0.849309	1.775699	0.584375	-0.05861	-0.12965	1.504367	1.340356	-0.35836	-0.43538	-0.1999	-0.23075	0.832734	-0.35435	-0.24457	0.267479	-0.02417	-0.72032	-0.04952	0.747332	0.102742	-0.06861	-1.25103	0.076029	0.164466	0.327304
-1.58118	-0.25675	0.277719	-1.99013	-1.41374	-1.25832	-1.2609	0.672498	0.080568	0.843861	0.937208	0.142359	0.179204	1.366695	0.076136	-0.31412	0.742545	-0.84745	0.070622	1.430761	-0.33798	0.374157	-0.04593	-0.71146	-0.18192	0.188766
1.540428	0.336032	1.519748	-0.07003	0.36225	0.141225	-1.07451	-1.31219	0.250658	0.933082	-0.51834	-1.16012	-1.17809	-0.96131	0.016311	-0.66837	0.346481	0.487834	-0.52745	0.538768	0.435577	-1.34778	0.892117	-0.27399	-0.14555	-0.29218
-0.82655	-0.03819	-1.09674	0.010344	0.647827	0.284241	0.971092	-0.05791	-0.35655	0.430667	-0.04065	-0.32989	0.494168	0.032229	-0.19428	-0.41109	0.572475	0.869141	-0.39769	0.434653	0.560434	-0.32921	0.108695	-0.11796	-0.45166	0.459832
0.211734	0.894421	0.19177	-1.37263	0.625959	0.118559	1.449409	-0.07845	-0.20838	1.217018	-1.17472	0.325892	0.7884	-0.26899	-0.41316	0.053134	-1.08455	0.147425	0.084681	0.587573	-0.05525	-0.45941	0.00321	0.257355	-0.12318	0.442544
-1.33519	-0.25135	0.325102	0.188866	0.907478	-0.65979	0.671486	-0.38812	0.505401	-0.11617	-0.65805	-1.08103	-0.6924	0.384646	1.920607	0.459986	-0.2418	-0.83161	0.725139	0.591542	-0.34107	0.769518	-0.79054	0.739937	-0.12363	-0.90693
0.429382	0.624284	-0.64735	0.661289	0.136967	-1.13485	-0.42689	0.09445	0.370148	0.095638	0.667286	0.1106	-0.60391	0.278466	-1.28181	-0.06657	1.288103	-0.54415	-0.49269	-0.09479	-0.06527	-0.54126	0.38401	-0.98609	0.541526	0.321425
0.83194	-1.95473	-0.55911	2.330739	1.118153	0.653705	1.158825	0.204907	-0.40541	0.050119	0.676662	-0.01576	0.059551	0.135324	0.039295	-0.76836	-0.0977	-0.32344	-0.6358	0.995281	-0.17525	0.646305	0.680283	-0.28527	-0.07443	0.308264
0.326056	0.758047	0.50319	-1.12763	0.796729	-1.85883	1.686242	-0.81926	0.258986	-0.12776	0.462028	0.554051	-0.09793	-0.59672	-0.16629	0.346606	0.41888	-0.33112	0.887591	-0.50356	1.432121	0.343301	0.537008	0.527177	0.114781	-0.33201
1.008485	-0.91304	0.34448	-0.21923	-1.34274	0.279274	-0.94494	0.208383	-1.93052	-0.83662	0.243189	-1.03592	0.678546	0.195219	0.98833	0.322464	0.469304	-0.19959	-0.26213	0.474541	1.697698	0.199876	-0.12084	-0.10939	-0.42724	-1.35015
0.910433	0.843297	-0.23643	-0.69778	-0.3447	-0.42384	-0.38025	-0.48163	-0.15143	0.042827	-0.28088	1.210601	-0.12897	0.936595	0.893339	-0.92299	-0.06711	-0.38416	1.236367	-0.5647	-0.00225	-0.35839	-0.01031	-0.22049	0.417214	0.7415
-0.53524	-0.44029	0.701949	0.748568	1.264147	-0.00187	-0.76888	-0.07566	0.344667	0.129355	1.856401	-1.19927	0.35321	0.224182	0.281408	-0.10145	-1.20679	-0.34336	-0.05896	-0.21651	-1.5738	-0.47777	0.912031	0.012195	0.074513	-0.33787
-1.04424	0.44962	-0.79329	-0.61815	-0.07699	0.48578	-1.49485	-0.98143	-0.40923	0.117101	-0.37431	-0.50918	0.862357	-1.09878	-0.58995	0.682654	0.943008	0.142478	0.460316	0.096335	0.044608	1.412848	0.624602	0.308632	0.93628	1.326736
0.475642	-1.70581	0.226696	0.744664	0.027229	-1.18299	-0.69788	-0.80623	-0.4354	-0.02821	0.066598	0.322423	-0.39006	0.035267	-1.01536	-1.22455	-0.86532	0.063135	1.164006	-0.27025	-0.79431	-0.35038	-0.68518	0.787302	-0.69415	0.438426
0.259503	-0.81771	-0.05806	0.349633	0.446973	0.291162	1.206575	-1.62313	-0.3298	-1.18829	-1.47875	-0.56666	0.384981	0.728293	0.027581	2.684348	0.652469	-0.99589	-0.24472	-0.45787	-0.64117	-0.25336	0.073334	-0.82852	0.192636	0.15608
1.720355	0.765516	-0.7619	0.554687	0.270492	-0.15594	0.593086	2.348666	1.311693	0.910708	0.343961	0.071833	0.086125	-0.08701	0.843605	0.808277	0.858724	0.983403	0.186648	0.35137	0.090972	0.50025	0.899342	-0.09761	0.339298	0.438052
-0.62547	-0.46501	1.343086	0.163334	1.445949	0.543114	-0.57871	0.66336	1.255392	0.20595	-0.17808	0.110044	0.557301	0.740646	0.839461	-1.01826	1.035195	0.36905	0.35183	-1.21352	1.428898	0.205244	-0.51692	0.499407	0.047293	1.060578
-1.3206	0.118228	-0.46694	-0.29019	0.018451	1.066933	0.808425	0.21563	0.848697	-0.15342	1.085186	1.69451	0.741656	-0.85841	0.079665	-0.1861	0.284947	0.409308	0.401855	0.911145	-1.13859	-0.19394	-0.22238	-0.61538	-0.20935	-1.25383
-0.28535	0.20766	0.159579	-0.73955	-1.09688	0.506479	0.317401	-0.77333	0.788351	-0.04995	-0.16295	-0.25609	-1.12118	-0.21945	-0.57217	-0.104	-0.05476	0.239615	0.486742	0.305011	-0.2246	0.788108	0.113033	-0.0458	-0.47559	-0.60544
0.463403	-0.37852	0.458386	0.829632	-1.9114	0.044052	0.921904	-0.32201	0.259392	-0.7171	0.309646	0.53838	-0.49164	-0.14044	0.17948	-0.55266	0.440108	-0.35825	-0.06978	-0.53536	-0.16201	0.498049	0.426034	0.17127	-0.01408	0.478895
-0.27821	1.168513	-0.06871	0.097681	1.479668	0.013763	-1.07844	0.242946	-0.72239	0.430625	0.127403	-0.43968	0.184098	-0.0761	-0.13128	-0.46187	0.760268	0.261093	0.738679	0.444776	0.232539	-0.31846	-0.09919	-0.18799	0.091357	-0.95187
0.489333	-0.7676	-0.69521	-1.50784	0.029271	-0.5344	-0.01227	0.005034	-0.58771	-1.03448	-1.5405	-0.60782	0.851548	0.362467	0.404257	-1.28109	-0.88039	1.874539	-0.34	0.078142	-0.95528	-0.62657	-0.37828	-1.03499	0.266336	0.632389
-0.10978	0.844668	0.179188	1.165377	0.047491	-0.91592	-0.48049	0.258946	-0.94897	0.090848	-0.21124	-0.11872	-1.48732	-0.05123	0.281386	0.510574	0.888494	0.86632	0.026185	0.835709	-0.52198	0.475613	-0.51571	0.249759	0.628412	0.181481
1.19974	-0.37068	0.252835	-0.43369	1.222571	-0.57904	0.130909	-0.61934	-0.8693	1.319158	1.009233	1.599728	0.595787	-0.3561	1.066283	0.050534	-0.51181	-1.03201	-0.87223	-1.1629	-0.47697	0.753295	0.015602	-0.37529	-1.07897	-0.07043
-0.06912	-0.40892	1.092843	-0.97408	0.117147	0.239125	0.9111	-0.33714	-1.5631	1.035197	1.041941	1.035596	-0.91288	-0.72714	-0.12079	0.969844	-0.4223	1.3022	0.020287	-0.14075	-0.10043	-0.15639	-0.18951	-0.5211	0.008987	0.254908
-0.30273	1.326485	-0.88387	0.23679	-0.99134	0.849085	0.290099	-0.69641	0.161749	1.105258	0.745565	-0.8726	0.449482	1.004538	-0.97835	-0.02393	-0.50057	-0.95107	0.093436	0.077241	1.158865	-0.94755	0.514831	-0.11305	-1.2418	-0.31615
-0.54359	-0.65617	0.83009	0.386128	0.122203	0.835221	-1.04042	0.928094	-0.65688	0.756603	-0.2827	0.289161	-0.04233	-1.03816	-0.89097	-0.14748	-0.55961	-1.68157	-0.451	0.684652	0.597577	-0.45488	-0.87861	0.20727	1.613508	0.559302
1.45266	-0.47984	-0.45785	-0.4913	1.292862	-0.14362	-1.42328	0.399818	0.592996	-0.36505	0.290846	-0.53822	0.395625	0.403815	-0.17231	0.556438	-0.75527	0.191662	-0.77018	0.786542	0.114675	-0.34029	-0.01802	0.557931	0.204966	-0.27213
-0.65207	0.645595	1.696472	-0.17615	0.156836	0.267638	-0.11528	0.665295	0.217395	-2.42709	-0.66397	1.251489	0.031691	-0.36931	-0.4307	-0.33374	-0.26885	-0.76734	-0.48361	0.550406	0.401192	0.093835	0.767537	-0.54648	-1.33138	0.394321
0.597483	-0.22514	1.367668	-0.32102	-1.43735	-0.98544	-0.86893	1.353419	0.499937	0.210961	-0.19684	-1.08034	0.685406	-0.13279	-0.63512	1.127111	-1.26402	0.4952	0.374181	-0.97983	-0.54737	1.045875	0.594382	0.139183	-0.0284	-0.45582
-1.16683	-1.76376	0.348671	-1.01102	-0.2128	1.064227	1.453809	0.19194	0.590271	0.400583	0.04546	0.068953	-0.31574	0.433335	0.099487	0.090743	-0.25216	-0.18412	0.572197	-0.69843	0.195137	-1.77365	0.750647	0.579456	1.706417	-0.47162
0.529591	1.45448	0.097438	0.48361	0.535289	1.797796	0.299975	-0.59297	-0.35658	-0.59235	-0.10638	-0.17179	-0.60483	1.293372	-0.17886	-0.30593	-1.77208	0.465676	1.640922	0.459888	0.444817	1.160518	0.271688	-0.26216	0.023414	0.463476
-0.08135	-0.16668	0.25471	-0.67746	-0.40879	-0.16471	1.54145	-0.5011	0.308921	-0.35022	1.355087	-0.58623	-0.31122	0.910167	-0.57318	-0.42847	1.083928	1.121619	-1.81313	0.137638	-0.07141	0.397043	-1.33218	0.350981	0.263918	-0.09806
-1.41761	0.312093	-1.32996	0.171013	-0.06229	-0.13751	1.154546	1.323846	-0.94662	0.789698	-1.07696	-1.28233	-0.58548	-0.7493	0.872	-0.66414	0.988193	-0.93291	0.315541	-0.71258	-0.77887	-0.30608	0.604903	-0.54971	-0.61403	0.267988
-0.38255	0.567579	0.083747	2.677924	-1.13364	-1.52234	-0.50899	-1.49227	0.87576	0.939293	-0.72857	0.849996	1.130123	0.048959	0.256326	0.425246	0.191825	0.468009	0.139256	-0.04095	0.328526	-1.15705	-0.55723	-0.55223	0.436441	-0.30161
-0.47468	0.501183	-0.08846	-0.38328	0.562763	-0.45491	0.901229	-0.47532	0.052117	0.12048	0.134498	-0.22936	-0.1408	0.049502	-0.10904	0.289595	0.099583	-0.10971	0.012178	0.151414	-0.04614	0.306699	-0.20399	0.266312	-0.02242	0.191577
-0.81088	0.039012	0.036298	0.418862	0.038689	0.849223	-1.02159	-0.21327	-1.4167	0.001306	0.280288	0.607989	0.569813	0.593822	-0.74599	-0.20579	1.526566	0.667842	-0.06644	-1.55616	-0.40183	0.902231	0.601386	0.639112	0.633224	-0.7846
-0.04565	0.779124	-1.0773	-0.74468	-1.07971	0.599093	-0.8376	-0.18593	0.352112	0.352702	0.947232	-0.21878	-0.67654	-0.21607	1.140605	0.911523	-1.59566	-0.49289	-2.00544	-0.7782	0.263717	-0.18313	-0.41662	0.02299	0.370126	1.227368
-0.80059	-0.79745	0.388603	-0.35132	0.283116	0.339927	-0.27112	0.329111	0.662418	0.284835	-1.0668	-0.401	-0.20078	-0.46775	-0.49368	0.33344	0.905097	0.879754	-1.17902	-0.70656	0.205862	-0.43697	-0.0546	0.584436	-1.9311	-0.13408
-0.87157	0.632025	-1.39339	0.796155	-0.39536	-1.37085	0.738039	0.726348	-0.72998	-0.86505	-0.57778	0.196955	-0.18374	0.063624	-0.25617	-1.36207	-1.46748	-0.28018	-1.42225	-0.49936	0.772042	0.141638	0.489195	0.90343	0.143831	-0.37289
1.028369	-0.65403	-1.70697	-1.01717	0.249908	0.298702	-1.65831	-0.62708	0.658932	-2.2004	0.368808	0.550972	-0.15093	-0.93531	0.581991	-0.1685	1.246979	-0.76479	0.582531	0.379882	0.084768	-0.44069	-0.28494	0.518908	-0.29093	0.063343

Coefficients

	sio251.1.fa	sio251.2.fa	sio251.3.fa	sio251.4.fa	sio251.5.fa	sio251.6.fa	sio251.7.fa	sio251.8.fa	sio251.9.fa	sio251.10.f	sio251.11.f	sio251.12.f	sio251.13.f	sio251.14.f	sio251.15.f	sio251.16.f	sio251.17.f	sio251.18.f	sio251.19.f	sio251.20.f
intake11	18.99084	26.84376	36.68424	52.13968	52.9491	51.51715	50.6719	56.22609	63.1902	66.97669	65.46668	63.26278	62.03852	62.04878	61.63759	60.6113	60.03769	59.42829	57.89175	56.42662
intake12	-27.1756	-32.8095	-30.5644	-36.8701	-45.2435	-46.7709	-56.6929	-60.1913	-63.1791	-66.7376	-70.4036	-73.2916	-74.39	-75.3816	-76.3279	-77.0135	-77.1931	-76.56	-75.2441	-73.2397
intake13	-5.00729	-0.61351	6.765859	-5.4888	-5.4345	-2.0237	-5.86161	-10.3257	-14.9444	-17.3133	-21.2387	-23.2713	-22.8364	-22.6061	-22.9546	-23.0832	-22.9491	-22.5877	-21.7416	-21.3282
intake14	29.19433	37.40663	58.46526	51.72972	56.35376	60.16293	65.40554	68.58784	69.88073	72.30931	73.00242	71.50634	71.48235	71.14545	71.2865	72.48416	73.259	74.82539	76.79505	80.80174
intake15	-7.40334	-22.1598	-15.2793	-1.60562	-1.81854	-4.47953	-6.72698	-7.70435	-10.2405	-11.3349	-10.9647	-9.24239	-7.22688	-6.61048	-6.6857	-7.09496	-6.69537	-7.05992	-6.79436	-6.58798
intake16	4.768062	-6.01879	-0.61333	6.396588	13.35115	12.82438	10.91611	14.94604	16.30291	19.37796	18.54605	19.13935	19.40923	18.2875	17.82288	17.68004	17.03366	17.02884	17.1435	16.66979
intake17	-1.67131	10.77745	26.89027	16.47166	7.069751	5.843278	6.018365	9.041256	9.753779	9.085101	7.628209	8.111331	7.028759	4.807754	3.062376	2.746531	2.569603	1.206678	0.331823	-1.34534
intake18	-1.02707	-13.839	-9.43718	-11.6755	-18.3816	-21.1839	-28.4096	-29.4046	-28.6925	-29.2572	-24.7312	-21.077	-18.4179	-16.3884	-14.7024	-12.6036	-9.61801	-6.19091	-2.32384	3.130262
intake19	-1.65491	-18.6529	-11.0561	-10.4448	-8.73759	-13.2612	-21.7799	-29.9724	-31.658	-34.1968	-36.953	-37.0435	-37.8926	-37.6156	-37.3569	-36.4648	-36.1492	-35.9093	-35.7473	-36.1928
intake110	-1.1199	5.532137	-4.03616	8.2097	8.411293	13.05182	20.55419	25.5255	30.30364	31.15026	31.34513	31.65441	32.26837	31.44002	31.07572	30.48506	29.50154	28.37147	26.26339	23.74724
intake111	19.21414	21.91218	-2.1069	4.150729	2.815389	5.54754	6.648265	9.784376	11.84541	10.3634	9.006721	6.445386	4.655679	2.747285	1.433504	0.494021	-0.62802	-1.29902	-2.17119	-3.88225
intake112	14.45896	29.12262	56.75767	62.68432	74.47484	79.18548	85.43804	90.60261	95.37836	97.93979	100.6265	103.5056	102.761	103.6017	102.8959	102.841	102.5843	102.3728	102.0013	101.1657
intake113	34.41267	38.92892	19.43099	19.96939	20.89846	21.18437	17.05424	16.97069	14.01057	11.51966	9.95185	6.978662	6.500531	6.208278	6.109568	5.025327	3.677604	2.213455	0.186347	-1.96552
intake114	37.60346	31.86977	36.20866	44.13914	53.99762	56.78092	62.61606	66.80693	67.64875	68.18249	65.73743	64.57893	64.33581	63.7521	63.37938	61.54482	59.73845	58.27464	56.53193	55.36978
intake115	8.786165	18.41192	3.143696	6.087619	-0.1853	-0.63128	-4.48223	-8.8495	-10.1729	-10.9311	-11.9259	-10.0409	-9.63877	-10.2762	-11.74	-12.222	-12.9652	-12.8956	-12.0707	-10.8227
intake116	42.82753	43.77398	44.37767	44.17743	49.90304	50.60136	49.95347	48.64027	45.16652	44.09438	42.5516	42.75387	41.87416	40.8231	39.91271	39.25413	38.77866	38.26252	37.3871	36.21621
intake117	-5.17227	3.574411	-9.32812	-11.6183	-21.1879	-19.024	-12.7253	-8.61188	-4.52047	0.920185	3.596462	5.54639	7.683732	8.906243	9.825149	10.30228	10.42448	11.10514	11.15332	11.26882
intake118	4.091471	13.44508	9.525086	8.619816	6.199099	8.368054	7.495238	7.718963	8.781426	7.206357	6.673692	4.123831	3.443364	1.999562	2.288505	3.475779	3.830511	4.071056	4.86197	6.568489
intake119	46.55998	47.69675	35.26992	35.15276	36.84791	37.59617	35.47012	36.13216	35.0329	34.86863	35.03101	34.78188	34.58748	35.38158	36.118	36.67733	37.22678	37.10378	36.58395	35.3495
intake120	-16.5858	-22.348	-14.7338	-9.05148	-0.09252	7.143983	10.739	12.18731	9.275367	10.05502	9.655106	8.993045	8.859472	9.478031	10.26334	11.20032	12.55597	13.78353	15.76521	18.16436
intake121	-2.86576	-2.04162	-20.7696	-13.8137	-12.0876	-6.1952	-1.43236	3.062188	2.695633	3.334863	4.369256	7.460775	9.095198	10.87357	11.69657	12.00794	11.68319	11.58575	12.19453	13.27062
intake122	11.14998	9.903034	-11.2484	-0.94417	7.501925	9.003486	3.863148	2.211695	1.260551	-0.78756	0.814572	-0.16675	0.414129	1.708198	2.183495	2.833405	3.656996	4.482776	5.977754	7.394298
intake123	-11.432	-27.3284	-32.471	-22.5427	-16.5294	-11.6385	-4.77922	-4.17889	-1.50069	-2.19481	-3.49727	-5.26715	-5.75711	-6.07298	-6.89255	-8.30243	-9.91584	-10.9287	-10.7609	-10.9905
intake124	24.5576	18.49985	5.611549	13.06951	18.88508	18.08224	11.55259	5.518931	2.869573	0.017507	-3.15259	-5.49084	-5.97706	-6.01765	-5.36291	-6.10949	-6.82521	-7.86652	-9.9765	-11.5109
intake125	-11.3548	-13.755	-28.7395	-33.6739	-27.3361	-20.5751	-15.9984	-13.6192	-11.7424	-7.93634	-7.56261	-10.8995	-12.0278	-13.1092	-13.9762	-13.0353	-12.3198	-11.5052	-11.4959	-12.335
intake126	39.31085	36.07082	24.47672	19.66879	15.93911	13.48893	9.115149	5.062509	-1.38572	-4.66573	-8.08771	-8.2795	-9.11142	-9.69367	-9.90126	-9.02929	-8.08424	-7.40128	-6.43184	-6.45938
intake127	45.22737	43.50043	41.77564	40.66241	48.79515	54.05149	62.8644	73.25827	77.7702	79.01338	79.11696	79.61168	79.74272	80.22895	80.54337	81.18902	81.56766	81.55693	81.70184	82.24501
intake128	38.78557	38.50176	37.67006	34.24584	25.57716	22.39885	18.83417	10.11943	1.59216	-3.86923	-7.42315	-12.4673	-16.0349	-19.5137	-20.3857	-21.1034	-21.5879	-22.1824	-22.7048	-23.3649
intake129	-9.92741	-18.2575	-7.18953	4.088654	12.48975	13.7906	6.95175	-0.29898	-1.69659	-4.15301	-7.31619	-7.12623	-8.98708	-9.5333	-10.0725	-10.285	-10.679	-11.0769	-11.2877	-10.6679
intake130	-8.09657	-19.915	-12.8716	4.818717	13.7754	17.91504	23.90859	26.08031	31.5638	31.14236	29.79387	29.76935	28.61982	28.00576	28.42455	29.82851	31.52951	32.92706	35.12196	37.47755
intake131	-16.8447	-15.6108	-3.46894	7.601096	9.175192	3.453349	-2.81931	-5.27338	-5.94689	-4.7224	-4.80883	-4.7722	-4.89951	-3.60422	-3.09342	-1.27483	-0.8201	0.967891	2.380175	3.655261
intake132	-14.1836	4.537633	31.87827	48.19414	49.15031	52.30821	53.55817	55.6468	54.84887	55.92834	55.42774	53.97584	53.40378	53.31121	53.91151	54.39096	54.12849	54.20946	54.33745	54.31016
intake133	17.3089	-1.359	-11.91	-0.79803	0.690635	-1.54206	-5.24358	-7.22124	-9.1747	-9.7594	-11.7027	-12.1095	-12.5772	-13.0896	-13.1781	-11.5908	-10.2257	-9.51242	-8.14331	-5.30915
intake134	-11.3254	-3.2367	1.828891	25.72328	29.62211	35.01082	43.97405	50.34736	51.5218	54.65543	56.66379	58.7704	59.27798	58.817	58.26586	58.46177	59.26656	59.75242	60.54971	61.25926
intake135	-27.6026	-17.3886	-15.9358	-6.28188	-7.32267	-7.11391	-11.1783	-12.1855	-13.2283	-14.3692	-14.6147	-15.1553	-15.3554	-15.4696	-15.7061	-15.556	-16.0582	-16.5977	-16.7902	-17.0973
intake136	-7.45623	-32.6512	-61.3694	-71.5543	-77.713	-78.7473	-83.2411	-84.2953	-81.468	-78.5684	-74.4779	-70.6792	-68.6682	-68.5929	-66.5379	-65.0401	-64.7406	-65.0075	-64.8406	-63.5002
intake137	18.31515	12.53787	-1.43671	10.05823	15.84609	14.01955	11.19265	14.07678	13.96816	12.83744	11.54915	11.03788	10.70856	9.320154	8.434211	6.586176	5.343623	3.626556	2.156757	-0.02199
intake138	20.47679	15.81013	11.90962	27.35459	31.10421	29.69981	31.98106	33.60276	34.40309	38.45038	41.96767	42.64841	42.39365	41.4455	40.78987	40.84554	40.46143	40.47988	39.44074	38.04811
intake139	4.490128	8.346851	35.77855	51.61317	62.39955	67.91892	65.85708	66.07363	68.20589	69.35166	69.65317	70.68715	71.32955	70.88439	70.66272	70.4039	70.75451	71.65545	72.94725	74.47135
intake140	-6.00785	-16.8392	-43.5388	-52.0579	-58.2787	-60.4617	-56.6717	-54.3018	-53.6676	-54.2675	-54.1108	-55.8485	-57.4432	-56.8318	-56.8812	-57.4716	-58.189	-57.566	-57.3565	-57.5241
intake141	-10.5863	-24.8087	-9.4028	-1.23017	1.045884	3.096258	-0.93205	-3.84498	-6.79485	-7.23392	-7.39345	-7.18353	-7.65274	-8.36777	-9.06648	-10.5762	-11.5336	-12.9047	-14.0391	-15.9327
intake142	-9.32959	-28.3224	-10.0709	-0.2773	2.762753	3.331703	4.91301	7.780121	9.059379	9.103288	7.631514	4.907532	3.667361	2.241716	1.522854	0.986547	0.676513	0.707598	1.251885	2.143133
intake143	17.83686	38.11454	43.06117	56.71688	63.75397	64.13242	58.63418	58.8936	57.62825	56.69588	56.91716	58.4254	58.92666	58.70045	57.75995	56.64671	55.08064	54.20463	53.70448	53.08837
intake144	13.3052	6.726768	8.261911	1.672372	-9.2787	-11.1647	-15.9881	-23.7707	-23.8764	-20.5156	-19.06	-17.8231	-17.344	-18.7417	-19.6987	-20.4155	-20.27	-20.2986	-20.5271	-21.5188
intake145	42.79879	49.96912	70.34642	69.86512	64.98864	61.76734	53.54332	49.35576	48.23675	50.54906	51.69012	51.4865	51.49053	52.18345	52.84753	52.9092	51.96726	51.78708	51.37145	51.35388
intake146	-5.50179	-1.04427	6.75296	4.045492	-3.50605	-1.49186	-2.1265	-1.66184	-1.23437	1.009181	0.97787	1.678993	1.069087	2.224029	2.994811	2.987026	2.80115	2.320845	1.91051	2.048727
intake147	18.82309	2.544854	8.515216	13.0589	16.43541	19.41174	21.64453	22.20944	22.7266	24.48192	28.85509	34.3906	37.58719	39.64352	40.7939	42.70228	44.84331	46.34101	47.48676	48.68856
intake148	-12.1038	-39.3758	-48.559	-43.8179	-48.0484	-48.3269	-48.5585	-49.2058	-48.4347	-48.5363	-49.0743	-49.3452	-49.4853	-49.2307	-48.7112	-46.3225	-43.3549	-40.5178	-36.9501	-34.5857
intake149	16.68181	-0.24691	0.578807	-0.31534	-1.67383	-0.27883	0.997287	2.849085	1.353747	-0.40576	-2.58793	-4.23364	-4.62992	-4.43051	-4.24367	-3.89344	-3.51913	-3.85034	-4.40106	-5.9788
intake150	45.76581	44.05503	35.02302	30.81734	28.7179	27.54815	27.70807	30.74642	32.36309	32.99789	32.62004	31.49993	31.60098	31.60834	32.08901	33.51483	34.79303	35.9228	37.75837	39.52456
intake151	13.13194	6.46707	6.326559	15.89251	24.70964	30.91126	35.80329	35.99941	35.20503	33.82275	31.14997	30.36172	27.89415	27.36176	26.51744	24.64783	22.7749	20.94293	18.38141	15.0057
intake152	-7.64949	-17.0306	-9.60219	4.812808	4.269425	7.296779	7.79805	9.07008	10.0881	10.22242	10.75161	9.523388	8.577191	7.877255	7.232066	6.544253	6.167029	5.784423	4.123038	3.340586
intake153	9.724642	14.89754	28.68048	23.74973	10.22859	7.274953	1.944138	-6.51713	-8.80779	-12.7712	-12.4121	-12.0716	-12.698	-14.1725	-14.5021	-15.5821	-16.7103	-17.6218	-18.4821	-18.543
intake154	24.84065	13.3745	22.30395	28.13425	33.91563	36.98985	42.79005	48.25844	51.83793	53.39858	53.80353	53.74884	53.392	53.6091	53.79927	53.73075	53.41504	53.53576	53.43983	53.99177
intake155	14.26287	3.957818	-2.84432	-12.9485	-28.1995	-34.0047	-35.4013	-34.3509	-34.1361	-35.0457	-37.8694	-38.3177	-37.6586	-37.2714	-37.754	-36.7849	-36.0929	-34.5779	-33.6864	-32.1078
intake156	30.55836	39.07242	34.65891	31.13443	23.10557	22.51571	24.22667	20.71021	18.59128	19.45169	17.95564	18.86459	19.96842	20.00199	20.41757	20.82216	20.8357	21.51965	22.25848	23.88689
intake157	18.89648	13.69492	14.84583	18.14178	5.891831	1.476527	-6.77675	-13.4625	-21.9911	-26.2679	-31.1428	-35.2876	-37.8939	-39.7579	-40.9558	-43.0742	-44.2568	-44.7117	-45.325	-45.6247
intake158	-15.085	-35.6163	-59.8547	-67.1733	-69.177	-63.9842	-57.4485	-56.8717	-55.204	-56.1728	-58.9962	-61.9584	-62.2268	-62.0417	-61.921	-60.9963	-60.3887	-60.0168	-59.7343	-58.9354
intake159	30.68764	52.65959	107.9983	121.2981	121.2236	122.5972	122.4718	120.3731	118.5413	116.395	115.6984	113.5945	113.5863	115.1798	116.4985	118.0569	119.215	120.6299	123.0354	124.7274
intake160	16.63246	5.988131	7.732996	12.27241	14.95035	20.37101	29.80893	37.70558	41.58204	42.20304	43.8558	43.8975	43.51854	43.63114	43.06135	43.27013	43.24008	43.06985	42.32318	42.05757
intake161	-1.51967	-13.3902	-14.9095	-10.2785	-12.0087	-7.36952	-4.85062	-6.56517	-6.57733	-6.84211	-7.8155	-10.0501	-11.4602	-12.894	-14.2954	-15.2854	-16.9277	-18.1611	-20.2677	-22.4113
intake162	22.8424	24.06328	45.28735	44.62132	37.23485	36.16439	38.02294	32.18485	29.24253	25.56112	23.10731	21.38588	21.16738	21.45355	21.95345	22.92453	23.39455	23.1882	23.22726	22.53531
intake163	20.05874	13.35456	6.043506	-13.9322	-36.9926	-40.4196	-47.209	-52.0407	-55.5157	-56.4151	-55.2782	-54.8089	-55.3858	-55.3472	-55.6632	-57.1212	-58.8355	-60.2348	-62.4315	-64.676
intake164	7.129426	-9.82103	-9.59329	-12.4832	-20.512	-19.7489	-14.3323	-10.4253	-7.51199	-6.6759	-4.66322	-3.153	-2.59224	-1.95176	-2.17564	-2.06428	-2.62601	-3.53883	-4.37543	-6.67621
intake165	-8.91921	-23.0842	-12.733	0.528658	-5.35163	-9.11987	-14.2682	-17.896	-20.1959	-19.1308	-16.4481	-12.6554	-9.31006	-5.82857	-4.21424	-2.57069	-1.99422	-1.57807	-2.11935	-3.60566
intake166	20.20105	23.72969	14.21123	14.57324	14.72575	17.66781	15.95097	14.32502	13.58287	14.89488	15.86215	16.38199	16.14757	16.77572	17.48005	17.91342	18.78029	18.35707	17.83925	18.28764
intake167	6.502975	21.67838	41.36917	58.47218	55.30713	53.64637	52.42979	45.63285	40.30525	35.7449	31.76227	28.95283	26.54484	24.96119	25.38488	25.71915	25.61063	25.4308	25.41687	25.92955
intake168	46.62757	59.6144	73.55639	83.67276	90.52389	93.11399	97.5333	101.9632	108.1277	112.9637	115.2113	116.9665	118.0127	118.355	118.2549	117.4514	117.3283	117.19	116.364	115.5901
intake169	48.17438	65.68776	95.42254	99.95865	99.92768	96.67673	90.28988	87.01881	84.69101	84.7121	84.55338	83.34298	81.80999	80.06176	79.14874	79.13443	78.79127	79.98372	82.17307	84.94739
intake170	28.45763	26.21564	15.79762	6.399878	-1.92481	-4.09304	-9.00814	-11.6215	-10.8046	-12.4211	-12.9441	-9.37648	-6.23491	-4.53304	-5.06352	-5.56522	-6.90981	-7.48309	-8.51765	-8.86778
intake171	-4.68051	-20.4968	-45.2813	-55.236	-55.1852	-54.2433	-53.3952	-51.7471	-52.761	-51.5936	-51.4196	-49.3191	-49.5332	-49.6223	-49.0021	-48.8638	-47.9752	-44.9427	-42.3373	-40.5057
intake172	33.80825	35.61597	14.47819	-12.214	-29.5472	-35.0873	-44.0522	-47.9261	-45.6651	-45.086	-46.2174	-48.2921	-50.4404	-50.8806	-51.3485	-52.5207	-52.8257	-53.3572	-53.9457	-55.2659
intake173	12.74524	12.82998	-0.20893	-13.0726	-18.5026	-17.7748	-20.1559	-20.2179	-17.9667	-18.1108	-16.6131	-17.8816	-18.6724	-18.1784	-18.6046	-17.5521	-17.3059	-16.7019	-14.9505	-12.574
intake174	19.67152	26.0046	39.09161	30.73096	26.4927	26.90812	26.72993	33.50296	37.70732	37.29223	38.43207	38.56965	38.12832	37.88465	38.85526	38.7996	39.5249	40.36484	41.56112	42.55225
intake175	46.59691	54.94884	55.60664	36.45198	29.78421	28.74718	29.39231	30.91211	31.13805	32.68223	32.79667	35.13907	36.66448	37.62868	37.61758	37.81444	37.52907	37.08453	37.23477	37.11834
intake176	10.38347	14.76707	15.13195	29.8933	38.92419	42.23468	40.75957	34.74143	36.40809	36.40145	35.71501	37.19117	38.22208	39.94118	39.55105	38.90312	38.94247	39.12015	38.80286	39.10855
intake177	13.241	-4.73569	-7.77458	-11.4528	-9.70104	-7.00231	-0.21461	7.90608	11.548	13.09125	15.38337	15.05465	14.45497	13.97273	14.31505	14.87421	15.40734	16.24104	17.13838	17.43795
intake178	5.399693	-2.36941	-6.27251	1.148607	13.22306	15.30728	12.32489	13.3276	16.76769	19.10635	20.90027	20.55725	19.61946	16.85279	16.30426	15.7618	13.3521	11.58863	8.738775	4.230649
intake179	38.92903	56.56756	67.16861	71.80076	75.01687	75.39458	71.87021	66.33904	63.01385	56.4284	54.24241	51.49347	50.85914	50.75868	50.68548	50.30436	48.84787	46.786	43.8239	40.2228
intake180	13.25522	20.53074	38.74374	48.90374	61.79622	66.75815	69.59839	66.45431	69.13505	74.26332	74.53892	74.90277	75.83897	75.92582	75.96935	74.62247	73.261	72.47152	72.74437	72.44363
intake181	-2.23386	-6.43518	-25.2565	-29.8579	-33.2262	-34.4289	-41.6074	-44.7346	-44.6114	-41.6124	-39.4388	-40.0903	-41.9579	-41.7747	-41.9125	-41.3323	-41.2839	-42.3295	-43.3489	-45.1117
intake182	8.949978	8.423	13.1784	-5.78247	-11.1117	-13.4731	-19.0073	-28.1253	-31.1103	-29.8873	-29.6879	-27.7747	-25.6816	-23.9976	-23.3997	-23.811	-24.5671	-25.855	-27.2099	-29.8897
intake183	11.7013	5.526958	-5.86993	-3.07009	-6.91703	-6.79628	-11.3434	-19.0506	-22.7461	-24.3544	-19.5027	-15.6451	-13.0121	-11.8213	-12.0014	-13.241	-15.3046	-16.1357	-16.2653	-18.3919
intake184	12.41882	-3.11195	-37.2406	-32.8218	-22.8712	-17.1313	-7.05344	-0.18286	2.905326	4.908639	8.044855	10.00596	9.172353	8.467919	8.640266	7.461585	6.498564	6.911005	8.816832	11.00741
intake185	-4.51004	-21.3679	-32.3651	-48.7138	-55.162	-54.0056	-52.611	-54.718	-56.9169	-61.3497	-61.8173	-60.2113	-59.4456	-59.3506	-59.3093	-59.5732	-59.3353	-57.9881	-57.1585	-55.8961
intake186	15.20632	17.80284	13.35465	10.86642	13.03401	17.00752	15.71951	15.56208	13.0517	7.848035	3.514291	4.428295	5.457091	6.554425	8.158106	11.80528	14.4915	16.23973	17.84995	19.13131
intake187	5.874853	-9.86917	-16.0137	-7.15617	1.569981	4.687651	8.689453	11.80537	13.23034	15.69727	18.77645	18.59732	19.73453	21.80314	21.97838	22.38211	23.25238	24.5732	27.56644	30.86571
intake188	11.43459	12.05203	-12.5702	-7.28368	-10.4075	-12.4117	-10.7975	-9.15088	-7.59692	-4.42574	-3.8239	-2.60615	-1.35949	0.216076	1.219604	3.062706	4.410009	5.23412	6.281967	7.631597
intake189	-15.8852	-13.0989	-0.2127	5.695261	4.884366	9.243277	13.92568	26.30574	33.16543	36.80684	39.23246	43.24194	45.17268	46.06792	47.03058	48.30615	49.81249	50.82229	51.63166	52.27475
intake190	70.9369	113.3654	179.8923	221.0917	249.3481	259.389	275.1803	289.5019	297.5919	303.1025	307.5881	311.0559	312.9847	314.735	315.7034	317.4806	319.167	320.47	322.3117	324.7825
intake191	34.05805	67.13291	107.8603	134.9809	147.4697	153.9063	161.8058	166.2141	169.194	169.3264	168.488	168.8378	169.8343	170.1353	170.8973	170.1509	169.8949	170.6365	170.6892	169.4475
intake192	42.48001	50.25511	48.00528	51.66624	50.01426	47.55915	46.15224	42.47356	44.93282	48.01438	51.74264	54.09756	54.78083	55.22249	55.89333	57.4071	59.08902	60.13116	62.05509	65.00705
intake193	9.918106	22.07051	10.4486	9.67731	3.552996	1.5185	-6.00004	-16.5841	-22.6277	-25.6647	-28.8053	-31.1441	-34.0227	-35.2289	-34.165	-32.3444	-30.0177	-26.9116	-22.7981	-18.6752
intake194	-6.50866	-15.5222	-37.8968	-48.5928	-52.955	-50.8671	-45.483	-38.3022	-32.5663	-24.7726	-23.0544	-20.1082	-18.7484	-17.5606	-16.7367	-16.7495	-18.2285	-20.1355	-21.5892	-22.8511
intake195	-15.0344	-12.3181	-14.6708	-26.7504	-35.1741	-33.5654	-35.2543	-32.0877	-29.5449	-24.2789	-21.8658	-20.533	-20.3426	-19.8293	-19.4036	-17.8149	-15.5975	-12.9575	-9.64895	-6.00724
intake196	-30.8705	-40.5863	-40.1301	-20.2039	-12.3254	-8.48178	-2.5871	6.233409	6.501008	6.158745	6.134965	6.629142	6.410751	6.700411	6.473778	5.673028	4.378852	2.897331	1.444849	-0.1702
intake197	26.5435	39.31416	30.91747	37.0517	34.19494	34.77611	35.95326	40.53328	44.60899	47.80362	50.51495	53.08175	54.87224	56.06459	57.38995	58.97305	62.16256	64.14303	66.63966	68.10573
intake198	-0.92325	-5.15671	-4.29791	-14.0039	-32.5299	-40.5119	-45.1102	-45.5514	-41.6144	-39.9701	-40.4667	-42.1823	-42.6637	-43.172	-43.1858	-43.5203	-44.1482	-44.9613	-44.529	-44.8199
intake199	-10.6372	-18.1335	-1.82932	-7.08943	-9.04207	-6.06322	-4.66131	1.7881	3.823577	4.799354	5.018957	6.91436	7.631428	7.990698	9.014885	9.321765	9.074734	8.96751	8.843749	9.202041
intake1100	23.64116	28.86168	16.1607	14.85821	8.01936	7.206173	7.563903	13.73756	16.74656	16.31656	14.10092	13.27267	15.39874	16.99999	16.63451	16.35449	16.5749	17.55253	19.72212	22.08559
intake1101	-7.21878	-13.9472	-13.4919	-10.9872	-17.8515	-18.3988	-20.8132	-17.2194	-9.04603	-5.31382	-5.28885	-6.01265	-7.3568	-7.68106	-8.1004	-9.84523	-11.1339	-12.4091	-14.3367	-16.3532
intake1102	0.67317	-2.55697	22.82662	26.35466	21.10506	23.16043	28.24512	34.07224	36.97323	40.58408	41.37981	39.73681	38.59549	37.50378	36.87604	35.99217	34.72184	33.33245	31.67992	29.19344
intake1103	-0.44792	1.440996	11.78321	12.22819	10.59593	10.74888	5.248682	2.970419	-3.50301	-5.61401	-6.06708	-7.84266	-7.79958	-7.47531	-7.54563	-8.29154	-9.29868	-10.6845	-13.0867	-16.0679
intake1104	29.82955	31.90048	34.82383	22.93447	22.04906	23.0906	21.66635	21.01678	22.55384	23.92096	25.43609	25.03486	25.82708	25.54735	24.89936	24.63733	25.71015	26.31079	26.0892	25.60021
intake1105	28.01224	39.48596	27.39091	19.79894	5.639246	1.07405	-0.86885	-6.23374	-11.1579	-13.3802	-14.3054	-13.3424	-13.3131	-12.0739	-11.1962	-10.4851	-9.41894	-8.4444	-7.17637	-5.7274
intake1106	2.133763	-6.35785	-9.03282	0.988536	3.599644	3.549894	-1.53206	-10.7644	-16.2488	-14.672	-11.739	-11.1256	-10.3848	-10.2545	-10.9604	-10.6103	-9.52361	-9.42565	-9.62569	-8.57863
intake1107	23.893	7.697633	-8.45707	-15.1358	-19.0263	-19.5864	-17.7745	-15.5365	-17.565	-18.3285	-20.4036	-22.3754	-23.6427	-24.4015	-24.7782	-25.7569	-26.7722	-28.0068	-29.2053	-29.9544
intake1108	-9.63695	-23.7539	-12.1515	-11.3378	-13.1803	-14.4597	-13.8783	-13.3812	-13.9585	-13.3664	-13.1582	-13.2532	-12.5546	-11.432	-11.1835	-12.2563	-13.1926	-13.8689	-13.6343	-12.4558
intake1109	25.88973	46.12892	67.92169	61.71307	55.03514	54.88545	57.28625	61.75835	61.24521	60.99559	64.65364	66.92805	65.73537	64.51842	62.49702	60.43213	58.10464	55.83637	53.09903	50.77935

	sio251.21.f	sio251.22.f	sio251.23.f	sio251.24.f	sio251.25.f	sio251.26.f	sio251.27.f	sio251.28.f	sio251.29.f	sio251.30.f	sio251.31.f	sio251.32.f	sio251.33.f	sio251.34.f	sio251.35.f	sio251.36.f	sio251.37.f	sio251.38.f	sio251.39.f
intake11	54.02151	52.99048	51.2065	50.76915	49.70195	49.43401	49.36815	49.32838	49.3413	49.33822	49.34032	49.36163	49.36504	49.36919	49.3709	49.37228	49.37361	49.37356	49.37353
intake12	-70.7156	-69.3638	-68.3791	-68.2751	-68.0468	-67.9396	-67.8793	-67.8553	-67.8011	-67.7829	-67.7568	-67.7407	-67.7335	-67.7285	-67.7253	-67.7227	-67.7201	-67.7192	-67.7175
intake13	-21.8029	-22.5502	-23.6053	-24.0114	-24.2358	-24.4294	-24.6158	-24.7005	-24.7713	-24.757	-24.7456	-24.7267	-24.7171	-24.7151	-24.7102	-24.7078	-24.7076	-24.7081	-24.7081
intake14	85.3588	88.22096	90.68339	90.97303	91.26383	91.70143	92.13784	92.32525	92.38783	92.4077	92.40095	92.41675	92.42291	92.41764	92.41648	92.41448	92.4109	92.40997	92.40997
intake15	-6.19075	-7.06526	-8.13667	-8.34854	-8.17775	-8.20901	-8.35002	-8.43793	-8.4353	-8.44689	-8.45417	-8.43375	-8.43095	-8.42814	-8.43133	-8.43168	-8.431	-8.43023	-8.42943
intake16	15.63986	15.18656	14.59373	14.43557	14.44607	14.57719	14.49677	14.28339	14.23087	14.1923	14.17069	14.15726	14.1505	14.15058	14.14844	14.14627	14.14275	14.14177	14.14191
intake17	-2.4957	-3.84594	-5.65099	-6.21609	-6.92742	-6.86096	-6.86243	-6.94294	-6.99535	-7.03111	-7.03883	-7.04476	-7.03543	-7.04131	-7.03767	-7.03696	-7.03493	-7.03435	-7.03459
intake18	9.433213	14.48459	18.85708	20.30412	21.72769	22.44524	23.02037	23.18938	23.21541	23.20132	23.2079	23.22437	23.23056	23.23416	23.23411	23.23576	23.23762	23.23653	23.23607
intake19	-36.6099	-36.5892	-36.0556	-35.8534	-35.4996	-35.2044	-34.7774	-34.6894	-34.6553	-34.6349	-34.6174	-34.5921	-34.5822	-34.5775	-34.5728	-34.5707	-34.5699	-34.5699	-34.5703
intake110	21.65517	19.83813	18.62698	18.09273	17.93487	17.77372	17.739	17.82457	17.88186	17.87848	17.89969	17.92998	17.9517	17.9686	17.97523	17.97837	17.97983	17.98012	17.9808
intake111	-4.70782	-5.18404	-4.99081	-4.97618	-5.0113	-5.09016	-5.16882	-5.16863	-5.10355	-5.07938	-5.07178	-5.07671	-5.0767	-5.07358	-5.07193	-5.0725	-5.07361	-5.07425	-5.07465
intake112	100.1906	98.64402	97.5332	97.11201	96.56326	96.05494	95.80448	95.76107	95.72807	95.71665	95.71392	95.70347	95.69021	95.69338	95.69561	95.69347	95.69196	95.691	95.69092
intake113	-3.6611	-5.2885	-6.89655	-7.19867	-7.18598	-7.09313	-6.97029	-6.96395	-6.97664	-6.98151	-6.9943	-7.02144	-7.03145	-7.03959	-7.04318	-7.04583	-7.04687	-7.04746	-7.0477
intake114	54.67316	54.31444	53.59592	53.38815	52.90623	52.75111	52.83388	52.82038	52.78162	52.77763	52.77447	52.77478	52.77935	52.77964	52.78021	52.78121	52.78241	52.78324	52.78395
intake115	-8.45615	-6.65706	-5.19819	-4.9042	-4.51243	-4.4502	-4.38371	-4.36215	-4.35457	-4.32772	-4.30907	-4.30617	-4.30858	-4.30555	-4.29953	-4.29901	-4.29985	-4.29981	-4.3002
intake116	34.65375	33.59658	33.04114	32.95103	32.8332	33.01117	32.97701	32.95774	32.97197	32.96449	32.95358	32.92554	32.90181	32.88784	32.87667	32.87407	32.87293	32.87181	32.87138
intake117	11.30181	11.37175	11.33922	11.48509	11.80362	11.97596	11.98802	11.93818	11.91253	11.87211	11.85902	11.83722	11.82196	11.81657	11.81596	11.81447	11.81476	11.8153	11.81577
intake118	7.760296	8.636734	9.687058	9.829866	10.32023	10.23936	10.20781	10.25168	10.27593	10.23716	10.23989	10.23406	10.23621	10.23265	10.22949	10.22912	10.22742	10.22737	10.22786
intake119	34.01805	33.35653	32.96258	32.82816	32.82954	32.86144	33.13649	33.21795	33.30768	33.35739	33.38688	33.40663	33.41019	33.41169	33.41138	33.41138	33.41016	33.40968	33.40999
intake120	20.06045	21.26141	23.13221	24.10428	24.78368	25.15618	25.41078	25.46851	25.35865	25.35373	25.33387	25.30343	25.28926	25.28317	25.28191	25.28155	25.28144	25.28074	25.28097
intake121	14.8729	16.81504	18.11993	18.70056	19.29289	19.45037	19.50729	19.46324	19.42044	19.38157	19.38268	19.38552	19.38142	19.3714	19.37057	19.37055	19.37088	19.37108	19.37171
intake122	8.62186	8.66431	8.037151	7.891464	7.556768	7.497354	7.531378	7.492139	7.434075	7.413364	7.39878	7.39448	7.397283	7.392259	7.391385	7.389399	7.388677	7.388663	7.388298
intake123	-10.6817	-9.79676	-9.17935	-8.86557	-8.58615	-8.40439	-8.03878	-8.01437	-7.89936	-7.87061	-7.891	-7.91314	-7.91961	-7.92556	-7.92715	-7.92733	-7.9269	-7.9281	-7.92789
intake124	-11.4786	-12.4064	-13.3619	-13.8203	-13.9939	-14.2802	-14.4413	-14.4959	-14.5355	-14.5258	-14.5182	-14.5346	-14.5487	-14.5566	-14.5653	-14.5672	-14.5676	-14.5678	-14.5681
intake125	-13.3411	-14.0777	-13.858	-13.7007	-13.8348	-13.8594	-13.9568	-13.9532	-13.9933	-13.9966	-14.0048	-13.9842	-13.9765	-13.9778	-13.9749	-13.9739	-13.9733	-13.9742	-13.9742
intake126	-7.15378	-7.4796	-7.92548	-8.34738	-9.50626	-10.0336	-10.3262	-10.4528	-10.4911	-10.4995	-10.4894	-10.4854	-10.4853	-10.4836	-10.4841	-10.4839	-10.4837	-10.4839	-10.4841
intake127	83.06239	83.80963	84.20597	84.22573	84.03376	83.98067	83.92881	83.81684	83.68753	83.6352	83.62797	83.62537	83.63039	83.63416	83.6334	83.63281	83.63308	83.6342	83.63524
intake128	-24.4604	-24.7872	-25.2529	-25.3007	-25.3458	-25.3492	-25.1824	-25.14	-25.1243	-25.1224	-25.1143	-25.1057	-25.1023	-25.0979	-25.0947	-25.0981	-25.1006	-25.0999	-25.0994
intake129	-9.65301	-9.28415	-9.80094	-9.74542	-9.49886	-9.47794	-9.27275	-9.25461	-9.30855	-9.36186	-9.39053	-9.41504	-9.42839	-9.43518	-9.43324	-9.43334	-9.43343	-9.43294	-9.4333
intake130	40.29156	41.90776	43.8232	44.4745	45.38803	45.71218	45.99285	45.87437	45.81156	45.81	45.8131	45.81606	45.81693	45.82111	45.81938	45.81971	45.82072	45.82101	45.82155
intake131	5.574361	6.982662	7.80818	8.023955	8.301669	8.630595	8.851278	8.962196	8.999722	9.008576	9.033233	9.053022	9.066972	9.070285	9.072079	9.073299	9.074412	9.075091	9.076171
intake132	53.77283	53.54494	53.39827	53.4686	53.44098	53.42008	53.42823	53.40226	53.42778	53.44165	53.45805	53.48402	53.49589	53.50533	53.50946	53.51057	53.51218	53.51288	53.51218
intake133	-1.86807	0.122973	1.428139	1.89062	2.224578	2.284861	2.330488	2.367567	2.400382	2.432668	2.444336	2.461173	2.464705	2.463705	2.465673	2.46686	2.466816	2.466917	2.466994
intake134	62.29966	62.67426	63.04664	63.06251	63.0194	62.87003	62.78183	62.73893	62.72101	62.71981	62.71392	62.70872	62.7011	62.6959	62.69406	62.69256	62.69208	62.69215	62.69237
intake135	-17.5796	-18.2848	-19.0783	-19.12	-18.8931	-18.8839	-18.9099	-18.9461	-18.9723	-19.0109	-19.0265	-19.0358	-19.035	-19.0342	-19.032	-19.0304	-19.0294	-19.0289	-19.0287
intake136	-62.8783	-63.0169	-63.3142	-63.2372	-63.3193	-63.4356	-63.5266	-63.5657	-63.5306	-63.4972	-63.4725	-63.4497	-63.45	-63.4548	-63.4556	-63.4568	-63.4583	-63.4597	-63.4607
intake137	-2.73702	-4.22622	-5.77398	-6.21752	-7.1006	-7.77321	-8.00889	-8.0381	-8.15554	-8.21143	-8.21655	-8.22004	-8.21938	-8.22508	-8.23227	-8.23216	-8.23187	-8.23195	-8.23225
intake138	36.70262	36.04045	36.14332	36.40355	36.52263	36.31402	36.06405	35.92371	35.90129	35.85877	35.8452	35.83582	35.83346	35.82321	35.82101	35.82141	35.8207	35.82127	35.82096
intake139	75.56122	75.78998	76.52593	76.87176	77.21791	77.58544	77.53426	77.6006	77.68477	77.70415	77.69344	77.68841	77.69262	77.69705	77.69273	77.69206	77.69066	77.6907	77.69019
intake140	-58.3097	-60.3619	-63.1015	-63.6639	-64.4337	-64.6152	-64.6037	-64.6231	-64.5878	-64.5988	-64.6015	-64.6199	-64.6128	-64.615	-64.6214	-64.6219	-64.6219	-64.6214	-64.6213
intake141	-17.9893	-19.6133	-20.2725	-20.5324	-20.8016	-20.7659	-20.687	-20.635	-20.5701	-20.5776	-20.5841	-20.5874	-20.5906	-20.5873	-20.5857	-20.5847	-20.5836	-20.5836	-20.5833
intake142	3.19412	3.68534	4.824823	5.285551	5.309862	5.383511	5.27607	5.374223	5.442151	5.436351	5.431714	5.422661	5.414714	5.409992	5.410057	5.409541	5.409517	5.409749	5.409895
intake143	52.14555	51.12324	50.36143	50.79892	51.00361	51.05347	51.04953	51.11404	51.15107	51.19003	51.20511	51.22555	51.23544	51.23661	51.24122	51.24298	51.24421	51.24378	51.24383
intake144	-21.7762	-21.8266	-22.0917	-22.4579	-22.4903	-22.6207	-22.5693	-22.481	-22.5055	-22.5091	-22.5249	-22.543	-22.5463	-22.5521	-22.5589	-22.5593	-22.5601	-22.5596	-22.5596
intake145	51.83998	51.74898	51.48469	51.33977	51.70011	51.88907	51.81336	51.74315	51.70871	51.71209	51.71558	51.70806	51.7043	51.70935	51.71308	51.71493	51.71694	51.7178	51.7177
intake146	2.072151	2.033457	1.726546	1.051513	0.156944	-0.60075	-1.03991	-1.15163	-1.21921	-1.21775	-1.21111	-1.20636	-1.19007	-1.17868	-1.17213	-1.17112	-1.1699	-1.17101	-1.1716
intake147	49.40461	49.30393	48.71203	48.2632	48.00158	47.99321	47.90275	47.89217	47.86737	47.86611	47.84244	47.81426	47.7976	47.79582	47.79649	47.79709	47.79762	47.79815	47.79871
intake148	-31.8053	-30.0487	-27.4322	-26.231	-24.734	-24.0137	-23.4651	-23.366	-23.3342	-23.3223	-23.31	-23.2995	-23.289	-23.283	-23.2846	-23.2851	-23.2862	-23.2865	-23.2874
intake149	-7.36773	-8.47403	-9.51382	-9.98506	-10.847	-11.4868	-12.2974	-12.4364	-12.4681	-12.4842	-12.4901	-12.5017	-12.5058	-12.5079	-12.5087	-12.5072	-12.5055	-12.5053	-12.5056
intake150	40.22245	40.61085	40.86328	41.07194	41.6393	41.77974	41.8585	41.86074	41.89622	41.90456	41.88907	41.88764	41.88496	41.88235	41.88599	41.88845	41.89031	41.89026	41.89063
intake151	11.43099	9.730018	7.583668	6.755557	6.572404	6.610461	6.627176	6.810497	6.963317	7.00086	7.014616	7.033871	7.04553	7.042635	7.040856	7.041071	7.040689	7.040901	7.040977
intake152	2.486393	2.457886	1.977177	1.772466	1.255766	0.846821	0.829054	0.878915	0.833949	0.838057	0.82319	0.816253	0.819163	0.820347	0.820275	0.820627	0.820415	0.820428	0.820742
intake153	-19.2642	-19.4523	-20.4898	-20.7654	-21.545	-21.7572	-22.0186	-22.0176	-21.9964	-21.9709	-21.9777	-21.9727	-21.9762	-21.979	-21.9883	-21.9926	-21.9945	-21.9949	-21.995
intake154	54.22712	54.13274	53.92198	53.68813	53.37488	53.0522	52.71325	52.57413	52.52625	52.55376	52.55487	52.55517	52.56172	52.56043	52.56314	52.56237	52.56334	52.56331	52.56397
intake155	-30.2244	-28.909	-27.8218	-27.3538	-26.9086	-26.9131	-27.2389	-27.2015	-27.1982	-27.1576	-27.1576	-27.1599	-27.1565	-27.1584	-27.1633	-27.1649	-27.1656	-27.1663	-27.1667
intake156	24.74512	24.52963	24.4582	24.35518	23.89921	23.75289	23.90292	23.94895	24.00584	24.01031	24.00997	24.0205	24.02179	24.02634	24.02947	24.03006	24.03383	24.03508	24.03634
intake157	-45.9408	-45.5621	-44.6121	-44.0631	-43.1792	-42.759	-42.5835	-42.5627	-42.6422	-42.656	-42.6623	-42.6709	-42.6703	-42.6659	-42.6611	-42.661	-42.66	-42.6599	-42.6598
intake158	-58.6088	-58.244	-58.3026	-57.6982	-57.3028	-57.0113	-56.9092	-56.8746	-56.8594	-56.8613	-56.8507	-56.8479	-56.8478	-56.8418	-56.8458	-56.8481	-56.8484	-56.8486	-56.8486
intake159	125.7125	126.4716	126.8225	127.3312	127.4591	127.3324	127.2515	127.1901	127.1659	127.121	127.1039	127.0844	127.0728	127.0653	127.0632	127.064	127.0616	127.0608	127.06
intake160	42.84181	43.80217	43.32888	43.56031	43.86869	44.07607	43.9878	43.96444	43.96681	43.95086	43.9575	43.96546	43.96849	43.97163	43.97203	43.97405	43.97526	43.97526	43.97541
intake161	-25.2705	-27.6655	-29.8574	-30.5528	-31.0863	-31.2455	-31.0605	-31.1254	-31.1617	-31.1547	-31.1539	-31.1517	-31.1596	-31.1689	-31.1704	-31.1684	-31.166	-31.1648	-31.1651
intake162	21.7503	21.25273	20.83285	20.89734	20.86626	20.6556	20.71937	20.75661	20.80793	20.85732	20.88534	20.92283	20.93181	20.93684	20.94158	20.94325	20.94395	20.94431	20.94389
intake163	-66.8776	-67.6551	-67.9773	-67.8027	-67.4985	-67.3949	-67.4213	-67.5503	-67.5958	-67.6185	-67.627	-67.6367	-67.6462	-67.6506	-67.6505	-67.6503	-67.6507	-67.6512	-67.6519
intake164	-9.47696	-11.3321	-13.3773	-14.2246	-15.0588	-15.4758	-15.8587	-15.9634	-16.0029	-15.988	-15.9664	-15.9594	-15.9539	-15.9521	-15.9504	-15.9508	-15.9533	-15.954	-15.9535
intake165	-5.47677	-6.99265	-7.35568	-7.42916	-7.23657	-6.95412	-6.58161	-6.46452	-6.49501	-6.50158	-6.48917	-6.48469	-6.48205	-6.48623	-6.48829	-6.49169	-6.49306	-6.49328	-6.49291
intake166	18.18963	18.25007	19.04611	19.05583	20.0826	20.67087	20.90522	20.96954	21.02638	21.03594	21.03053	21.03546	21.04043	21.03481	21.03319	21.03485	21.03477	21.03573	21.03616
intake167	26.22543	26.78425	27.78155	28.02613	28.59111	29.05152	29.08375	29.04995	28.99557	28.94649	28.92603	28.92655	28.93859	28.9363	28.93087	28.92841	28.92805	28.92602	28.92508
intake168	114.5037	113.3747	112.3891	111.8123	111.7645	111.6035	111.5532	111.4953	111.532	111.5114	111.5049	111.5121	111.5202	111.5304	111.5408	111.5419	111.5452	111.5454	111.5447
intake169	87.13307	88.35412	89.34195	90.0646	91.02278	91.06842	91.10348	91.20302	91.24214	91.24921	91.26343	91.28045	91.28676	91.28815	91.28535	91.28395	91.28319	91.2837	91.28473
intake170	-9.37241	-8.83472	-7.77891	-7.60282	-7.53581	-7.37264	-7.26001	-7.43573	-7.4884	-7.51517	-7.5331	-7.53787	-7.53693	-7.53356	-7.53629	-7.53783	-7.54039	-7.54134	-7.54151
intake171	-38.2359	-36.8448	-36.1261	-35.9517	-35.7282	-35.9829	-36.038	-35.9954	-35.9409	-35.9583	-35.9911	-35.9897	-35.9849	-35.9843	-35.9857	-35.9881	-35.9909	-35.9924	-35.9937
intake172	-56.8128	-57.8306	-58.0936	-58.1257	-58.0377	-57.9531	-57.9295	-57.8272	-57.7673	-57.7955	-57.7971	-57.8126	-57.826	-57.8299	-57.8306	-57.8317	-57.8332	-57.8337	-57.8327
intake173	-10.0344	-8.78539	-6.55753	-5.91174	-5.45302	-5.487	-5.17852	-5.16303	-5.15981	-5.19143	-5.22273	-5.25685	-5.27035	-5.27488	-5.28274	-5.28533	-5.28537	-5.28533	-5.2857
intake174	43.46881	44.61013	45.47531	46.03778	46.62993	46.79961	46.87471	46.81632	46.81289	46.86265	46.85054	46.83567	46.84648	46.85334	46.85933	46.86231	46.86464	46.86649	46.86745
intake175	37.1173	37.1712	37.23257	37.23664	37.40427	37.6641	37.923	38.00769	38.05528	38.03825	38.03423	38.0241	38.02748	38.03056	38.03159	38.03241	38.03437	38.03511	38.03468
intake176	38.81625	38.08541	37.70546	37.45096	36.67199	36.33663	35.98231	35.95795	35.92937	35.90489	35.90375	35.91248	35.92348	35.92207	35.92215	35.922	35.92123	35.92164	35.92146
intake177	18.56313	19.18038	19.60937	19.63712	20.02865	20.4408	20.7176	20.78893	20.76034	20.74255	20.73442	20.73869	20.74284	20.74848	20.75576	20.75722	20.75685	20.75715	20.75707
intake178	-0.55815	-3.22797	-5.26345	-6.35057	-7.8458	-8.49645	-8.78184	-8.76745	-8.77159	-8.75757	-8.75568	-8.77308	-8.77658	-8.77514	-8.77218	-8.76961	-8.76603	-8.76529	-8.76503
intake179	37.86773	36.01744	34.44973	34.1112	33.54765	33.31934	33.06207	33.03583	33.03299	33.03595	33.02615	33.03604	33.03827	33.04449	33.0476	33.04765	33.04609	33.04601	33.04571
intake180	73.1311	73.63868	73.95545	74.1022	74.0844	74.11652	74.14993	74.07879	74.09905	74.13609	74.16917	74.18277	74.19568	74.19416	74.1887	74.18562	74.18287	74.18202	74.18186
intake181	-46.4805	-47.5671	-48.6283	-49.038	-49.1133	-49.0893	-49.4162	-49.5751	-49.6155	-49.625	-49.6386	-49.6109	-49.6032	-49.5974	-49.6	-49.6011	-49.6006	-49.6004	-49.5993
intake182	-31.8794	-33.0213	-34.3189	-33.8791	-33.1049	-33.0891	-32.9825	-32.8744	-32.8165	-32.7988	-32.7951	-32.7834	-32.7706	-32.7705	-32.7707	-32.7714	-32.77	-32.7695	-32.769
intake183	-21.0833	-22.791	-25.5581	-26.739	-28.1196	-28.5465	-28.8226	-28.8283	-28.8722	-28.8879	-28.9037	-28.9107	-28.9119	-28.9095	-28.9072	-28.9059	-28.9043	-28.9036	-28.9029
intake184	13.23244	14.2105	15.92977	16.85409	17.75306	18.35454	18.44626	18.51782	18.4882	18.50781	18.54759	18.58767	18.60757	18.61889	18.6231	18.62344	18.6239	18.62397	18.62342
intake185	-54.602	-54.4797	-53.5048	-53.1018	-53.0057	-53.077	-53.2547	-53.3574	-53.3705	-53.3883	-53.3862	-53.3766	-53.376	-53.3818	-53.3757	-53.3726	-53.3707	-53.3696	-53.3689
intake186	20.39715	21.58764	22.85433	22.98613	22.77624	22.68109	22.51298	22.61671	22.62532	22.61081	22.60355	22.59344	22.5924	22.59123	22.58845	22.58606	22.58668	22.58744	22.58755
intake187	34.17328	37.57584	40.87607	41.24381	41.80917	41.94588	41.88132	41.8716	41.87372	41.88456	41.9033	41.91658	41.91868	41.9148	41.91151	41.91043	41.9119	41.91247	41.91223
intake188	10.33342	12.24144	14.50082	15.78883	16.62685	17.12939	17.40937	17.48324	17.56026	17.61611	17.6039	17.58789	17.5812	17.57649	17.57898	17.58093	17.58195	17.58224	17.58252
intake189	52.28093	51.73944	50.73316	50.49944	50.39207	50.36699	50.54676	50.58794	50.69207	50.72317	50.74621	50.73784	50.73821	50.73041	50.72512	50.72298	50.72017	50.71852	50.7183
intake190	327.3543	329.3343	331.1319	331.8735	332.5556	332.8675	333.016	333.064	333.0882	333.0978	333.1024	333.1029	333.1037	333.1044	333.1041	333.1037	333.1038	333.104	333.104
intake191	168.8155	168.1724	168.2537	168.0299	167.8072	167.7542	167.747	167.6673	167.5643	167.5362	167.5247	167.5151	167.503	167.499	167.4948	167.4961	167.4956	167.4955	167.496
intake192	67.45135	69.35632	71.15041	71.62172	72.01194	72.27832	72.33493	72.3873	72.44974	72.45892	72.46642	72.45701	72.45837	72.45463	72.45738	72.45907	72.46005	72.45967	72.45918
intake193	-13.3468	-9.85614	-7.80378	-7.25917	-7.10484	-6.81818	-6.73456	-6.73917	-6.72694	-6.7338	-6.73679	-6.74949	-6.76412	-6.76609	-6.76393	-6.76219	-6.76157	-6.76173	-6.76168
intake194	-23.8996	-24.9039	-26.2319	-26.5298	-27.1193	-27.418	-27.5878	-27.5398	-27.6292	-27.6782	-27.705	-27.7102	-27.7149	-27.7101	-27.7117	-27.7098	-27.7114	-27.7116	-27.7121
intake195	-2.16362	1.199475	4.413105	5.489729	6.022909	6.21249	6.395559	6.353293	6.384922	6.420978	6.44106	6.458232	6.460868	6.46153	6.454978	6.453986	6.454725	6.456469	6.456572
intake196	-1.63285	-2.18964	-3.09736	-3.72215	-4.10871	-4.44417	-4.47653	-4.4462	-4.38571	-4.36823	-4.3799	-4.38983	-4.39857	-4.40238	-4.40185	-4.40474	-4.40733	-4.40747	-4.40764
intake197	70.04537	71.42957	72.6611	73.23843	73.23056	73.25121	73.48148	73.62111	73.68325	73.6933	73.68867	73.70512	73.71063	73.71271	73.71419	73.71338	73.71369	73.71394	73.71436
intake198	-44.2396	-44.5435	-44.5893	-45.0992	-45.8691	-46.2513	-46.5287	-46.645	-46.7746	-46.8053	-46.8289	-46.8343	-46.8308	-46.8339	-46.8337	-46.835	-46.8365	-46.8372	-46.8373
intake199	10.85111	12.22909	14.34676	15.46795	16.41655	16.95089	17.19839	17.26194	17.31248	17.31278	17.32043	17.32577	17.33494	17.33915	17.34215	17.3418	17.34169	17.34213	17.34228
intake1100	23.95614	25.14116	26.26139	26.62412	27.63081	28.27437	28.58881	28.76712	28.82402	28.86068	28.87113	28.87642	28.87231	28.87563	28.87804	28.87834	28.8777	28.87737	28.87613
intake1101	-19.3142	-21.4843	-23.236	-23.7581	-24.7149	-25.0017	-25.2442	-25.2526	-25.3089	-25.2962	-25.2867	-25.2835	-25.2874	-25.2947	-25.2941	-25.2959	-25.2975	-25.297	-25.297
intake1102	27.06411	24.81791	21.66154	20.4873	19.86634	19.67405	19.55044	19.64174	19.66103	19.65621	19.65635	19.637	19.62075	19.6144	19.61084	19.61041	19.61142	19.6109	19.61044
intake1103	-18.2513	-20.6281	-22.9128	-24.1819	-26.0056	-26.6768	-26.9537	-27.0795	-27.0164	-26.9895	-26.9708	-26.945	-26.9292	-26.9249	-26.9256	-26.9265	-26.9275	-26.9275	-26.9283
intake1104	26.2003	26.74069	26.41026	26.43104	26.29101	26.18836	26.13251	26.05334	25.97587	25.93845	25.95918	25.97761	25.98089	25.97717	25.97748	25.97586	25.97457	25.97358	25.97309
intake1105	-4.40591	-3.92098	-2.83774	-2.96943	-3.21175	-3.32671	-3.38193	-3.39259	-3.39139	-3.36814	-3.35955	-3.36337	-3.35895	-3.36077	-3.36266	-3.36137	-3.36194	-3.36192	-3.36152
intake1106	-7.63789	-6.98536	-6.57502	-6.09757	-6.07465	-6.02738	-6.02386	-6.02469	-6.01431	-6.01835	-6.01546	-6.03771	-6.02727	-6.00887	-5.99964	-5.99769	-5.99875	-5.99933	-5.9993
intake1107	-31.6906	-33.382	-35.0502	-35.815	-36.6541	-37.039	-36.8771	-36.8532	-36.837	-36.8441	-36.8372	-36.8206	-36.8098	-36.7987	-36.7955	-36.7933	-36.793	-36.7933	-36.7935
intake1108	-10.2189	-8.55765	-5.84439	-5.08856	-5.34322	-5.49463	-5.61567	-5.54289	-5.44395	-5.47587	-5.48692	-5.49737	-5.50624	-5.50937	-5.50711	-5.50589	-5.50495	-5.50452	-5.50403
intake1109	49.22653	47.82453	47.37084	47.41505	47.3251	47.32956	47.53366	47.61241	47.60643	47.64077	47.65251	47.67284	47.67741	47.67549	47.6687	47.66856	47.66868	47.66789	47.66765

sio251.40.f	sio251.41.f	sio251.42.f	sio251.43.f	sio251.44.f	sio251.45.f	sio251.46.f	sio251.47.f	sio251.48.f	sio251.49.f	sio251.50.f	sio251.51.f	sio251.52.factors
49.37351	49.37336	49.37344	49.37348	49.37346	49.37346	49.37346	49.37346	49.37346	49.37346	49.37346	49.37346	49.37346
−67.7165	−67.7162	−67.7162	−67.7162	−67.7162	−67.7162	−67.7162	−67.7162	−67.7162	−67.7162	−67.7162	−67.7162	−67.7162
−24.7078	−24.7078	−24.7078	−24.7078	−24.7078	−24.7078	−24.7078	−24.7078	−24.7078	−24.7078	−24.7078	−24.7078	−24.7078
92.40977	92.40952	92.40953	92.40952	92.40954	92.40954	92.40954	92.40954	92.40954	92.40954	92.40954	92.40954	92.40954
−8.42923	−8.42932	−8.42925	−8.42923	−8.42922	−8.42922	−8.42921	−8.42921	−8.42921	−8.42921	−8.42921	−8.42921	−8.42921
14.14196	14.14198	14.14199	14.14201	14.14203	14.14203	14.14203	14.14203	14.14203	14.14203	14.14203	14.14203	14.14203
−7.03487	−7.03478	−7.0347	−7.03467	−7.03467	−7.03467	−7.03466	−7.03467	−7.03467	−7.03467	−7.03467	−7.03467	−7.03467
23.23531	23.23527	23.23527	23.23527	23.23526	23.23527	23.23527	23.23527	23.23527	23.23527	23.23527	23.23527	23.23527
−34.5706	−34.5707	−34.5708	−34.5708	−34.5708	−34.5708	−34.5708	−34.5708	−34.5708	−34.5708	−34.5708	−34.5708	−34.5708
17.98096	17.981	17.98101	17.98101	17.98099	17.98099	17.98099	17.98099	17.98099	17.98099	17.98099	17.98099	17.98099
−5.07479	−5.07473	−5.07474	−5.07468	−5.0747	−5.0747	−5.0747	−5.0747	−5.0747	−5.0747	−5.0747	−5.0747	−5.0747
95.69081	95.69082	95.69089	95.69089	95.69093	95.69092	95.69092	95.69092	95.69092	95.69092	95.69092	95.69092	95.69092
−7.04748	−7.04727	−7.0472	−7.04714	−7.04714	−7.04714	−7.04714	−7.04714	−7.04714	−7.04714	−7.04714	−7.04714	−7.04714
52.78416	52.78418	52.78418	52.78419	52.78417	52.78416	52.78416	52.78416	52.78416	52.78416	52.78416	52.78416	52.78416
−4.30057	−4.30069	−4.30066	−4.30057	−4.30056	−4.30056	−4.30056	−4.30055	−4.30055	−4.30055	−4.30055	−4.30055	−4.30055
32.87058	32.87027	32.87017	32.87009	32.87006	32.87006	32.87006	32.87006	32.87006	32.87006	32.87006	32.87006	32.87006
11.81604	11.81598	11.81594	11.8159	11.81588	11.81588	11.81588	11.81588	11.81588	11.81588	11.81588	11.81588	11.81588
10.22795	10.2279	10.22787	10.22783	10.22784	10.22784	10.22785	10.22785	10.22785	10.22785	10.22785	10.22785	10.22785
33.41033	33.4103	33.4102	33.41021	33.41022	33.41022	33.41022	33.41022	33.41022	33.41022	33.41022	33.41022	33.41022
25.28105	25.28092	25.28093	25.28098	25.28103	25.28103	25.28102	25.28102	25.28103	25.28103	25.28103	25.28103	25.28103
19.37173	19.3717	19.37172	19.37172	19.37175	19.37175	19.37175	19.37175	19.37175	19.37175	19.37175	19.37175	19.37175
7.388409	7.388547	7.388503	7.388532	7.388494	7.388494	7.388493	7.388493	7.388493	7.388493	7.388493	7.388493	7.388493
−7.92841	−7.92869	−7.92869	−7.92869	−7.92869	−7.92869	−7.92869	−7.92869	−7.92869	−7.92869	−7.92869	−7.92869	−7.92869
−14.568	−14.5682	−14.5681	−14.5681	−14.5681	−14.5681	−14.5681	−14.5681	−14.5681	−14.5681	−14.5681	−14.5681	−14.5681
−13.9734	−13.9733	−13.9733	−13.9732	−13.9732	−13.9732	−13.9732	−13.9732	−13.9732	−13.9732	−13.9732	−13.9732	−13.9732
−10.4838	−10.4838	−10.4838	−10.4839	−10.4839	−10.4839	−10.4839	−10.4839	−10.4839	−10.4839	−10.4839	−10.4839	−10.4839
83.63575	83.63572	83.63572	83.63573	83.63575	83.63576	83.63576	83.63576	83.63576	83.63576	83.63576	83.63576	83.63576
−25.0993	−25.0993	−25.0993	−25.0992	−25.0993	−25.0993	−25.0993	−25.0993	−25.0993	−25.0993	−25.0993	−25.0993	−25.0993
−9.43276	−9.43257	−9.43251	−9.43249	−9.43247	−9.43246	−9.43246	−9.43246	−9.43246	−9.43246	−9.43246	−9.43246	−9.43246
45.82188	45.82197	45.82193	45.82191	45.82184	45.82184	45.82184	45.82184	45.82184	45.82184	45.82184	45.82184	45.82184
9.076939	9.077242	9.077335	9.077375	9.077399	9.077401	9.077402	9.077402	9.077402	9.077402	9.077402	9.077402	9.077402
53.51192	53.512	53.51213	53.51219	53.51222	53.51223	53.51223	53.51223	53.51223	53.51223	53.51223	53.51223	53.51223
2.466916	2.466892	2.466797	2.46677	2.466795	2.466796	2.466797	2.466797	2.466797	2.466797	2.466797	2.466797	2.466797
62.69227	62.69217	62.69205	62.69199	62.69198	62.69198	62.69199	62.69199	62.69199	62.69199	62.69199	62.69199	62.69199
−19.0286	−19.0287	−19.0287	−19.0288	−19.0288	−19.0288	−19.0288	−19.0288	−19.0288	−19.0288	−19.0288	−19.0288	−19.0288
−63.4609	−63.461	−63.4609	−63.4609	−63.4609	−63.4609	−63.4609	−63.4609	−63.4609	−63.4609	−63.4609	−63.4609	−63.4609
−8.23248	−8.23241	−8.23241	−8.23239	−8.23236	−8.23236	−8.23236	−8.23236	−8.23236	−8.23236	−8.23236	−8.23236	−8.23236
35.8207	35.82068	35.82065	35.8206	35.8206	35.82061	35.82061	35.82061	35.82061	35.82061	35.82061	35.82061	35.82061
77.68943	77.68918	77.68913	77.68914	77.68915	77.68915	77.68915	77.68915	77.68915	77.68915	77.68915	77.68915	77.68915
−64.6215	−64.6217	−64.6216	−64.6216	−64.6215	−64.6216	−64.6216	−64.6216	−64.6216	−64.6216	−64.6216	−64.6216	−64.6216
−20.5834	−20.5834	−20.5833	−20.5833	−20.5833	−20.5833	−20.5833	−20.5833	−20.5833	−20.5833	−20.5833	−20.5833	−20.5833
5.410034	5.410319	5.410413	5.410416	5.410409	5.410406	5.410405	5.410404	5.410405	5.410404	5.410404	5.410404	5.410404
51.24317	51.24289	51.24288	51.2429	51.24291	51.24291	51.24291	51.24291	51.24291	51.24291	51.24291	51.24291	51.24291
−22.5596	−22.5595	−22.5594	−22.5594	−22.5594	−22.5594	−22.5594	−22.5594	−22.5594	−22.5594	−22.5594	−22.5594	−22.5594
51.71775	51.7176	51.71764	51.71763	51.71761	51.71761	51.71761	51.71761	51.71761	51.71761	51.71761	51.71761	51.71761
−1.17208	−1.17212	−1.17212	−1.17214	−1.17214	−1.17215	−1.17215	−1.17215	−1.17215	−1.17215	−1.17215	−1.17215	−1.17215
47.79914	47.79931	47.79941	47.79947	47.79949	47.79949	47.79949	47.79949	47.79949	47.79949	47.79949	47.79949	47.79949
−23.2875	−23.2876	−23.2875	−23.2874	−23.2874	−23.2874	−23.2874	−23.2874	−23.2874	−23.2874	−23.2874	−23.2874	−23.2874
−12.5052	−12.5051	−12.5051	−12.5051	−12.5051	−12.5051	−12.5051	−12.5051	−12.5051	−12.5051	−12.5051	−12.5051	−12.5051
41.89103	41.8912	41.89115	41.8911	41.89108	41.89107	41.89107	41.89107	41.89107	41.89107	41.89107	41.89107	41.89107
7.041318	7.041598	7.041665	7.041622	7.041628	7.041632	7.041631	7.041631	7.041632	7.041631	7.041631	7.041631	7.041631
0.820707	0.820544	0.820468	0.820438	0.820394	0.820387	0.820385	0.820384	0.820384	0.820384	0.820384	0.820384	0.820384
−21.9948	−21.9948	−21.9949	−21.9949	−21.9949	−21.9949	−21.9949	−21.9949	−21.9949	−21.9949	−21.9949	−21.9949	−21.9949
52.56427	52.5648	52.56499	52.56505	52.56506	52.56506	52.56506	52.56506	52.56506	52.56506	52.56506	52.56506	52.56506
−27.1672	−27.1673	−27.1674	−27.1674	−27.1674	−27.1674	−27.1674	−27.1674	−27.1674	−27.1674	−27.1674	−27.1674	−27.1674
24.03676	24.03711	24.03717	24.03719	24.03721	24.03721	24.03721	24.03721	24.03721	24.03721	24.03721	24.03721	24.03721
−42.66	−42.66	−42.66	−42.66	−42.6601	−42.6601	−42.6601	−42.6601	−42.6601	−42.6601	−42.6601	−42.6601	−42.6601
−56.8485	−56.8482	−56.8481	−56.848	−56.848	−56.848	−56.848	−56.848	−56.848	−56.848	−56.848	−56.848	−56.848
127.0594	127.0595	127.0595	127.0594	127.0594	127.0594	127.0594	127.0594	127.0594	127.0594	127.0594	127.0594	127.0594
43.97573	43.97565	43.97564	43.97567	43.97568	43.97568	43.97568	43.97568	43.97568	43.97568	43.97568	43.97568	43.97568
−31.1654	−31.1654	−31.1655	−31.1655	−31.1655	−31.1655	−31.1655	−31.1655	−31.1655	−31.1655	−31.1655	−31.1655	−31.1655
20.94377	20.94375	20.94378	20.94381	20.94381	20.94381	20.94381	20.94381	20.94381	20.94381	20.94381	20.94381	20.94381
−67.6526	−67.653	−67.6531	−67.6532	−67.6532	−67.6532	−67.6532	−67.6532	−67.6532	−67.6532	−67.6532	−67.6532	−67.6532
−15.9532	−15.9536	−15.9536	−15.9535	−15.9535	−15.9535	−15.9535	−15.9535	−15.9535	−15.9535	−15.9535	−15.9535	−15.9535
−6.49279	−6.49269	−6.49274	−6.49276	−6.49278	−6.49278	−6.49278	−6.49278	−6.49278	−6.49278	−6.49278	−6.49278	−6.49278
21.03663	21.03679	21.03687	21.03691	21.03691	21.0369	21.0369	21.0369	21.0369	21.0369	21.0369	21.0369	21.0369
28.92508	28.92507	28.92506	28.92515	28.9252	28.9252	28.9252	28.9252	28.9252	28.9252	28.9252	28.9252	28.9252
111.5445	111.5443	111.5442	111.5442	111.5442	111.5442	111.5442	111.5442	111.5442	111.5442	111.5442	111.5442	111.5442
91.28538	91.2855	91.2856	91.28568	91.28568	91.28568	91.28568	91.28567	91.28567	91.28567	91.28567	91.28567	91.28567
−7.54112	−7.54075	−7.54074	−7.54074	−7.54073	−7.54074	−7.54074	−7.54074	−7.54074	−7.54074	−7.54074	−7.54074	−7.54074
−35.994	−35.9942	−35.9942	−35.9942	−35.9942	−35.9942	−35.9942	−35.9942	−35.9942	−35.9942	−35.9942	−35.9942	−35.9942
−57.8324	−57.8321	−57.8319	−57.8319	−57.8319	−57.8319	−57.8319	−57.8319	−57.8319	−57.8319	−57.8319	−57.8319	−57.8319
−5.28596	−5.28608	−5.28612	−5.28614	−5.28614	−5.28615	−5.28615	−5.28615	−5.28615	−5.28615	−5.28615	−5.28615	−5.28615
46.86801	46.86824	46.86825	46.86829	46.86832	46.86833	46.86833	46.86833	46.86833	46.86833	46.86833	46.86833	46.86833
38.03453	38.03441	38.0344	38.03443	38.03441	38.03442	38.03442	38.03442	38.03442	38.03442	38.03442	38.03442	38.03442
35.92136	35.92105	35.92095	35.92091	35.92091	35.92091	35.92091	35.92091	35.92091	35.92091	35.92091	35.92091	35.92091
20.75638	20.75623	20.75607	20.75597	20.75591	20.7559	20.7559	20.7559	20.7559	20.7559	20.7559	20.7559	20.7559
−8.76509	−8.76512	−8.76511	−8.76511	−8.76507	−8.76508	−8.76507	−8.76507	−8.76507	−8.76507	−8.76507	−8.76507	−8.76507
33.04539	33.04537	33.04532	33.04532	33.04536	33.04536	33.04536	33.04536	33.04536	33.04536	33.04536	33.04536	33.04536
74.18181	74.18196	74.18187	74.18182	74.18179	74.18179	74.18179	74.18179	74.18179	74.18179	74.18179	74.18179	74.18179
−49.5993	−49.5992	−49.5992	−49.5992	−49.5992	−49.5992	−49.5992	−49.5992	−49.5992	−49.5992	−49.5992	−49.5992	−49.5992
−32.7691	−32.7693	−32.7694	−32.7695	−32.7694	−32.7694	−32.7694	−32.7694	−32.7694	−32.7694	−32.7694	−32.7694	−32.7694
−28.902	−28.9017	−28.9016	−28.9016	−28.9016	−28.9016	−28.9016	−28.9016	−28.9016	−28.9016	−28.9016	−28.9016	−28.9016
18.62327	18.62326	18.62324	18.62318	18.62314	18.62314	18.62314	18.62314	18.62314	18.62314	18.62314	18.62314	18.62314
−53.369	−53.3688	−53.3688	−53.3687	−53.3687	−53.3687	−53.3687	−53.3687	−53.3687	−53.3687	−53.3687	−53.3687	−53.3687
22.58714	22.58688	22.58687	22.58683	22.58679	22.58679	22.58679	22.58679	22.58679	22.58679	22.58679	22.58679	22.58679
41.91169	41.91153	41.91153	41.91155	41.91157	41.91157	41.91157	41.91157	41.91157	41.91157	41.91157	41.91157	41.91157
17.58285	17.58291	17.58306	17.58307	17.58309	17.58309	17.58309	17.58309	17.58309	17.58309	17.58309	17.58309	17.58309
50.71834	50.71824	50.71814	50.71808	50.71805	50.71805	50.71805	50.71805	50.71805	50.71805	50.71805	50.71805	50.71805
333.1038	333.1038	333.1038	333.1038	333.1037	333.1037	333.1037	333.1037	333.1037	333.1037	333.1037	333.1037	333.1037
167.4963	167.4964	167.4965	167.4966	167.4966	167.4967	167.4967	167.4967	167.4967	167.4967	167.4967	167.4967	167.4967
72.45911	72.45913	72.45921	72.45925	72.45927	72.45928	72.45928	72.45928	72.45928	72.45928	72.45928	72.45928	72.45928
−6.76188	−6.76205	−6.7622	−6.76226	−6.76227	−6.76227	−6.76228	−6.76228	−6.76228	−6.76228	−6.76228	−6.76228	−6.76228
−27.7127	−27.7125	−27.7125	−27.7124	−27.7125	−27.7125	−27.7125	−27.7125	−27.7125	−27.7125	−27.7125	−27.7125	−27.7125
6.456926	6.457032	6.457038	6.457074	6.457113	6.457113	6.457113	6.457113	6.457112	6.457112	6.457112	6.457112	6.457112
−4.40758	−4.4073	−4.40713	−4.40705	−4.40701	−4.40701	−4.40702	−4.40701	−4.40701	−4.40701	−4.40701	−4.40701	−4.40701
73.71473	73.7149	73.71495	73.71492	73.71491	73.7149	73.7149	73.7149	73.7149	73.7149	73.7149	73.7149	73.7149
−46.8373	−46.8374	−46.8375	−46.8375	−46.8375	−46.8375	−46.8375	−46.8375	−46.8375	−46.8375	−46.8375	−46.8375	−46.8375
17.34273	17.34275	17.34275	17.34275	17.34276	17.34276	17.34275	17.34275	17.34275	17.34275	17.34275	17.34275	17.34275
28.87587	28.87577	28.87568	28.87563	28.87568	28.87567	28.87568	28.87567	28.87567	28.87567	28.87567	28.87567	28.87567
−25.2972	−25.2974	−25.2975	−25.2975	−25.2975	−25.2975	−25.2975	−25.2975	−25.2975	−25.2975	−25.2975	−25.2975	−25.2975
19.61036	19.61044	19.6103	19.61035	19.6104	19.6104	19.6104	19.6104	19.6104	19.6104	19.6104	19.6104	19.6104
−26.929	−26.9292	−26.9292	−26.9292	−26.9292	−26.9292	−26.9292	−26.9292	−26.9292	−26.9292	−26.9292	−26.9292	−26.9292
25.97281	25.97297	25.97311	25.97319	25.97323	25.97323	25.97323	25.97323	25.97323	25.97323	25.97323	25.97323	25.97323
−3.36161	−3.36136	−3.36131	−3.36126	−3.36123	−3.36123	−3.36123	−3.36123	−3.36123	−3.36123	−3.36123	−3.36123	−3.36123
−5.9992	−5.99906	−5.99909	−5.99912	−5.99911	−5.9991	−5.9991	−5.9991	−5.9991	−5.9991	−5.9991	−5.9991	−5.9991
−36.7936	−36.7939	−36.7939	−36.7939	−36.7939	−36.7939	−36.7939	−36.7939	−36.7939	−36.7939	−36.7939	−36.7939	−36.7939
−5.50306	−5.50299	−5.50306	−5.50303	−5.50307	−5.50308	−5.50307	−5.50307	−5.50307	−5.50307	−5.50307	−5.50307	−5.50307
47.66826	47.66846	47.6685	47.66844	47.66843	47.66843	47.66843	47.66843	47.66843	47.66843	47.66843	47.66843	47.66843

Loadingweights

	Factor1	Factor2	Factor3	Factor4	Factor5	Factor6	Factor7	Factor8	Factor9	Factor10	Factor11	Factor12	Factor13	Factor14	Factor15	Factor16	Factor17	Factor18	Factor19	Factor20	Factor21	Factor22	Factor23	Factor24	Factor25	Factor26	Factor27	Factor28	Factor29	Factor30	Factor31	Factor32	Factor33	Factor34	Factor35	Factor36	Factor37	Factor38	Factor39
intake11	0.080793	0.037666	0.030906	0.125561	-0.1113	-0.06326	0.01802	0.189266	0.179589	-0.00343	-0.2085	-0.0831	-0.0138	0.09998	-0.06719	-0.07517	0.059687	-0.01881	-0.12979	0.032896	-0.14895	0.128365	-0.15153	0.057159	-0.19466	0.127243	0.053075	-0.03652	0.061488	-0.02852	0.024243	0.164961	-0.08628	0.060983	0.000915	0.069279	0.044859	-0.06671	-0.00115
intake12	-0.11561	-0.00682	0.039575	-0.06916	-0.09059	0.033484	-0.22846	0.119723	-0.0523	-0.09939	-0.10466	-0.03934	0.038107	-0.03828	-0.08304	0.026935	0.072795	0.126524	0.090396	0.074499	0.080728	-0.07907	-0.04772	-0.08262	0.038717	0.004418	0.001618	0.015667	0.097876	0.019392	0.158521	-0.01846	0.011693	0.045172	0.035533	0.136346	0.088499	0.037925	0.258858
intake13	-0.0213	0.044375	0.029725	-0.14304	0.100019	0.129013	-0.20358	-0.04888	-0.10179	0.009699	-0.15745	0.027779	0.160973	-0.00561	-0.07256	0.024692	0.040768	0.039579	0.066647	-0.07199	-0.14316	-0.08685	-0.06652	-0.021	0.046979	-0.06029	-0.05504	-0.06357	-0.08305	0.144905	0.053663	0.089985	0.031882	-0.0387	0.121179	0.089359	-0.10843	-0.0911	0.047809
intake14	0.124202	0.025477	0.103776	-0.12732	0.132651	0.094204	0.034275	-0.02245	-0.01439	0.089361	-0.0355	-0.12724	0.080268	-0.04115	0.046557	0.126643	-0.05261	0.144861	0.019826	0.249349	0.082929	-0.0558	-0.03234	-0.19561	0.005351	0.196522	0.134472	0.133339	-0.02111	0.017089	-0.10288	0.170894	-0.00444	-0.16697	0.028996	-0.12603	-0.20421	0.001151	0.075418
intake15	-0.0315	-0.11293	0.110292	0.116314	-0.11418	-0.09879	0.014906	0.021461	-0.08859	0.016086	0.056352	0.098322	0.128373	-0.08361	-0.05562	-0.04017	0.125321	-0.11372	0.110131	-0.0121	0.030366	-0.2367	-0.04816	0.053205	0.110014	-0.06738	-0.08347	-0.08214	0.079497	########	-0.02797	0.212502	-0.09036	0.037025	-0.12932	0.034557	0.075189	0.096889	0.105309
intake16	0.020285	-0.09773	0.083117	0.05425	0.060899	-0.09603	-0.03723	0.166922	-0.03041	0.121232	-0.14601	0.073425	-0.00292	-0.1652	0.003993	0.036346	-0.08323	0.088405	0.01907	-0.0823	-0.08868	0.052539	-0.03178	0.014229	0.04737	0.080225	-0.10173	-0.27998	0.064813	-0.11208	-0.07259	0.01475	-0.02116	0.059651	-0.06516	-0.12038	-0.19016	-0.00883	0.10501
intake17	-0.00711	0.107072	0.0524	-0.14979	-0.07471	0.056157	0.04012	0.088493	-0.03609	-0.05543	-0.06507	0.091925	-0.14701	-0.19678	-0.12552	0.162866	0.018304	-0.19465	0.115719	-0.09672	0.086877	-0.11539	-0.10179	0.012272	-0.0532	0.240209	-0.02514	-0.11451	-0.04648	-0.09966	0.029331	-0.00414	0.19214	-0.20708	0.180121	-0.01685	0.136797	0.008684	-0.09658
intake18	-0.00437	-0.1061	0.085327	-0.03487	-0.09528	-0.03319	-0.11772	0.134702	0.053038	-0.04999	0.285328	0.05441	0.092757	0.045236	0.129421	0.056624	0.170781	0.138928	-0.02673	0.159171	0.128892	0.148198	-0.05224	-0.00068	0.018019	0.058639	0.126036	0.064365	-0.0852	-0.09266	0.087084	0.09848	-0.00839	0.025101	-0.04502	0.122851	0.076323	-0.26246	-0.00708
intake19	-0.00704	-0.14033	0.124862	-0.01568	0.023922	-0.19055	-0.10366	-0.05598	0.108468	-0.08378	-0.08299	0.107659	-0.08928	0.103846	0.02229	0.076686	-0.08146	-0.00498	-0.01909	-0.08565	0.005161	0.06343	0.093594	0.009416	0.048548	0.088672	0.179851	-0.00568	0.000353	0.051971	0.086629	0.107166	-0.00664	0.017391	0.085065	0.066914	-0.03907	-0.04264	-0.06826
intake110	-0.00476	0.057552	-0.09263	0.149224	-0.09562	0.174132	0.072386	-0.01936	0.097469	-0.0937	-0.01514	0.012363	0.051043	-0.15541	-0.00048	-0.02859	-0.07122	-0.04594	-0.1232	-0.02417	0.071521	-0.07814	0.084588	-0.0494	0.102587	-0.05823	0.035205	0.132893	0.052846	-0.08062	0.175038	0.127678	0.153745	0.18068	-0.00596	0.107208	-0.03396	-0.01448	0.117009
intake111	0.081743	-0.00597	-0.17121	0.131044	-0.07318	0.118414	-0.04844	0.070802	0.020039	-0.13676	-0.03353	-0.11299	-0.05678	-0.09981	-0.0778	0.038893	-0.04181	0.046954	-0.013	-0.10177	0.144522	0.018833	0.115072	-0.01836	-0.01452	-0.04023	-0.02424	0.024471	0.141469	0.033411	-0.0011	-0.08548	0.033008	0.067404	0.011755	-0.07332	-0.0658	-0.05786	-0.03343
intake112	0.061513	0.101637	0.119161	-0.0202	0.151425	0.05013	0.03605	0.015122	0.093077	-0.00419	0.078168	0.079116	-0.24229	0.167526	-0.17339	0.074567	-0.03335	0.001419	-0.0194	-0.05796	-0.02128	-0.18074	0.057459	-0.01973	-0.04517	-0.16955	0.016286	0.015085	-0.03538	-0.01322	0.007259	-0.07093	-0.14727	0.182029	0.028375	-0.20128	-0.02152	-0.09343	0.060986
intake113	0.146403	-0.01335	-0.14917	0.061202	0.011355	0.00071	-0.12164	0.091839	-0.12686	-0.04451	-0.01425	-0.13145	0.111711	0.001161	0.004652	-0.11798	-0.05678	-0.04939	-0.04801	0.013413	0.076199	-0.09525	-0.01944	0.085753	0.088297	0.05535	0.04887	-0.02875	-0.03293	-0.00721	-0.08816	-0.15089	0.016994	-0.09088	-0.0088	-0.12871	0.044099	-0.05186	-0.00303
intake114	0.159977	-0.10422	0.053906	0.066463	0.102554	-0.00199	0.080081	-0.00513	-0.05638	0.00355	-0.16135	0.024397	0.037218	-0.05505	-0.01909	-0.17599	-0.0195	0.013944	-0.003	0.105731	0.076338	0.024788	-0.07052	0.011266	-0.08503	0.035815	0.112036	-0.04474	-0.07302	0.026515	-0.01477	0.021075	0.072605	-0.03267	0.01358	0.063399	0.051919	0.084657	0.081984
intake115	0.037379	0.067769	-0.14348	0.073016	-0.12987	0.051616	-0.0929	-0.04562	0.039352	-0.00136	-0.03493	0.165333	-0.05983	-0.11393	-0.19167	0.110699	-0.04852	0.113718	0.117747	0.045539	0.177921	0.035526	-0.03651	-0.0704	0.034121	-0.06962	0.0221	0.010322	-0.00159	0.111141	0.078899	-0.08511	-0.05872	0.086332	0.143427	-0.0758	-0.09677	0.041624	-0.08363
intake116	0.182202	-0.05588	-0.00014	-0.00372	0.098427	-0.03605	-0.0379	-0.02537	-0.12191	0.044302	-0.05744	0.076943	-0.10891	-0.06351	-0.07305	0.02605	0.020951	-0.01736	-0.04242	-0.0273	-0.06129	0.003493	0.07636	0.034917	-0.00979	0.145669	-0.08765	-0.01701	0.047145	-0.04966	-0.06686	-0.16938	-0.20143	-0.10001	-0.1655	0.019231	0.033761	-0.1365	0.001625
intake117	-0.022	0.081189	-0.12393	0.014174	-0.14328	0.186818	0.110645	-0.01809	0.086641	0.165716	-0.01311	0.018265	0.129285	-0.01592	0.062335	-0.04867	-0.05109	0.09254	-0.1194	0.008494	-0.01341	0.009746	-0.01765	0.058216	0.053813	0.020596	-0.05452	-0.07489	-0.01398	-0.15104	-0.00117	-0.10464	-0.10443	0.013522	0.047069	-0.09923	0.098774	0.078299	0.052696
intake118	0.017406	0.07248	-0.06698	0.002222	-0.03361	0.108876	-0.08641	0.026794	0.041235	-0.1128	0.018108	-0.14621	0.065773	-0.12979	0.148515	0.108413	-0.11895	-0.01028	0.08046	0.113498	-0.08498	0.011787	0.043999	-0.07627	0.105714	-0.18782	0.008174	0.072358	0.01595	-0.20076	0.118902	-0.06446	0.074013	-0.09561	-0.05138	0.03285	-0.12614	0.064963	0.105035
intake119	0.198081	-0.05985	-0.08744	0.034427	0.029609	0.009905	-0.07992	0.068848	-0.06249	0.023146	0.014772	-0.02312	-0.00778	0.117218	0.063046	-0.0179	0.005736	-0.09558	-0.05943	-0.09005	-0.0139	0.052344	0.039345	-0.00144	0.038021	0.019845	0.173385	0.031687	0.126898	0.119211	0.106492	-0.00845	-0.07327	0.002215	-0.0277	0.006286	-0.10404	-0.02685	0.10405
intake120	-0.07056	-0.02369	0.075766	0.034718	0.105525	0.177059	-0.10965	-0.03785	-0.15875	0.124673	-0.04819	-0.02718	0.021865	0.089868	0.083326	0.021529	0.079929	0.010269	0.084972	0.027359	-0.08405	-0.02114	0.138806	0.130881	-0.07015	0.047047	0.035855	0.004097	-0.28753	0.103674	-0.14356	-0.13688	-0.02897	-0.011	0.035907	-0.00261	0.009219	-0.11206	0.103831
intake121	-0.01219	0.011195	-0.12789	0.122836	-0.02707	0.20504	-0.03888	0.028669	-0.11566	0.043914	0.04102	0.161243	0.010149	0.095697	0.007635	-0.05742	-0.0987	0.029143	0.114674	0.054841	0.083034	0.1734	-0.08853	0.055093	0.013503	-0.06506	-0.01895	-0.08042	-0.05616	-0.12452	0.106415	0.017155	-0.08603	-0.18211	0.096223	0.014935	0.028298	0.018855	0.114682
intake122	0.047436	-0.02709	-0.1349	0.163085	0.059478	-0.03526	-0.18437	0.066953	-0.00472	-0.07938	0.159203	-0.13077	0.118911	0.118673	-0.01442	0.022907	0.036582	0.019099	0.082821	-0.03324	-0.03227	-0.1653	-0.12122	0.023091	-0.05866	0.055517	0.044472	-0.0665	-0.09342	-0.02602	-0.06241	0.049645	0.074511	-0.13288	0.034644	-0.13071	0.037944	0.028848	-0.07422
intake123	-0.04864	-0.1165	0.035499	0.113493	0.021385	0.120123	0.04752	-0.1384	0.103136	-0.11347	-0.05514	-0.06303	0.043694	-0.00091	-0.11021	-0.074	-0.05137	0.059794	0.215646	-0.05694	0.08676	0.138738	-0.03618	0.040582	-0.00479	0.037461	0.179826	-0.07766	0.229814	-0.00325	-0.23232	-0.0648	0.041654	-0.0735	0.024034	0.017533	0.044687	-0.21643	0.139467
intake124	0.104476	-0.08749	-0.05899	0.111104	0.038018	-0.09407	-0.15617	-0.03538	0.018667	-0.07231	-0.09759	-0.02352	0.075617	0.033995	0.108699	-0.16427	-0.0048	-0.06862	-0.14007	0.110576	0.253673	-0.19596	-0.01811	-0.05272	0.076716	-0.13327	-0.00429	-0.02837	-0.04026	0.088599	0.034797	-0.18034	-0.12282	-0.04996	-0.16261	0.018611	0.064237	-0.02145	-0.04475
intake125	-0.04831	-0.00324	-0.08893	-0.00617	0.147063	0.187637	-0.069	-0.0318	0.028821	0.144358	-0.09776	-0.23548	0.059663	-0.04735	-0.0639	0.211853	-0.0241	0.032226	-0.15059	-0.11631	-0.02569	-0.00928	0.163571	0.031415	-0.08385	0.021784	-0.0566	0.035151	-0.09038	0.031295	-0.05627	0.219163	-0.01133	-0.09361	0.102176	0.018437	0.002542	-0.17857	0.091176
intake126	0.167241	-0.08581	-0.06275	-0.01462	-0.02418	-0.05234	-0.04955	-0.02404	-0.19047	0.014967	-0.09901	0.128429	-0.08176	-0.00741	0.007501	0.127854	0.02324	-0.02061	0.026182	-0.15113	-0.10705	0.031259	-0.02644	-0.10189	-0.22622	-0.01171	-0.00517	-0.09071	0.022839	0.006212	0.100502	-0.02526	-0.02541	0.034091	-0.03452	0.025698	0.011693	-0.04749	-0.02128
intake127	0.192412	-0.08192	-0.00388	-0.00613	0.14651	0.110845	0.09064	0.116611	-0.03442	-0.06533	-0.03302	0.028357	-0.01287	0.051619	0.016539	0.040884	-0.03468	-0.05396	0.02497	0.052364	0.043294	0.038229	-0.0533	-0.04146	-0.06256	0.019786	-0.01559	-0.14393	-0.18744	-0.08561	0.074774	0.020764	0.099426	0.03879	-0.06823	-0.02941	0.052087	0.174628	0.124437
intake128	0.165006	-0.06019	-0.00428	-0.03168	-0.11888	-0.02602	-0.00637	-0.18518	-0.17744	-0.03882	-0.03812	-0.18615	-0.11661	-0.15734	0.104337	0.0146	0.028258	-0.02871	0.028161	-0.011	-0.06901	0.087939	-0.0299	0.039592	########	0.010383	0.113448	0.009169	-0.00129	-0.00947	0.05871	0.023643	-0.00027	0.065065	0.04439	-0.31339	-0.04712	0.228971	0.037004
intake129	-0.04223	-0.05518	0.109933	0.080491	0.050841	-0.04239	-0.22523	-0.06555	0.1001	-0.08779	-0.10964	0.142935	-0.2172	0.080123	-0.04864	0.036596	-0.0321	-0.00959	0.040845	0.116939	0.062587	-0.06782	-0.15591	0.084276	0.057712	-0.05539	0.130389	-0.03722	-0.13261	-0.17622	-0.09105	-0.03596	-0.05503	-0.03337	0.140606	-0.04471	-0.00219	0.084713	-0.1133
intake130	-0.03445	-0.08722	0.098505	0.155804	0.008381	0.059407	0.045378	-0.07053	0.190254	-0.18018	-0.06644	0.053976	-0.12631	0.014119	0.110997	0.119338	0.066398	-0.01005	0.087038	-0.01102	0.070409	-0.06564	0.07724	0.006168	0.089146	-0.0488	0.070961	-0.25557	-0.03723	0.064835	0.028332	0.006366	-0.00533	0.083027	-0.10222	0.057377	0.068507	0.005842	0.086139
intake131	-0.07166	0.035473	0.075169	0.075345	-0.06291	-0.23463	-0.00749	0.068274	0.025162	0.083294	-0.04367	0.005979	-0.01617	0.175905	-0.00877	0.158223	-0.19652	0.224642	-0.10987	-0.04004	0.101684	0.018365	-0.08615	-0.02147	0.022126	0.131258	0.028882	0.090487	-0.01143	-0.0034	0.171287	0.020222	0.096405	-0.04739	0.013644	0.056394	0.033411	0.064246	0.169138
intake132	-0.06034	0.178415	0.099436	0.083997	-0.11578	0.109546	-0.0566	0.035339	-0.08104	0.07932	-0.06358	-0.07512	0.01695	0.034266	0.103566	-0.01179	-0.11359	0.049225	0.005173	-0.02319	-0.08187	0.029185	0.011562	0.044201	-0.02788	-0.00556	0.013077	-0.0396	0.077351	0.032694	0.093892	0.11996	0.02109	0.103445	0.009153	0.004164	0.080926	0.04524	-0.19943
intake133	0.073638	-0.18263	0.011748	0.140759	-0.06469	-0.10111	-0.03735	0.024087	-0.04106	0.025863	-0.09647	0.053285	-0.02939	-0.02783	0.021824	0.199485	-0.01508	-0.07369	0.083865	0.180049	0.093607	-0.07724	-0.09669	0.01115	-0.03019	-0.05476	0.008777	0.038911	0.040288	0.106554	0.010407	0.071735	-0.0544	-0.05187	0.071056	0.050622	-0.06309	0.018727	0.007822
intake134	-0.04818	0.084833	-0.00181	0.223185	-0.12731	0.162152	0.090956	-0.00985	-0.09034	0.12955	0.020051	0.056135	-0.0604	-0.09986	-0.05659	0.086692	0.100945	-0.03294	0.035684	-0.02401	0.052164	-0.07026	0.004883	-0.03996	-0.01724	-0.08272	-0.00503	-0.03412	-0.003	0.015172	-0.0433	-0.00869	-0.09002	-0.0477	0.007778	-0.07623	0.034721	0.031438	0.040941
intake135	-0.11743	0.126948	-0.0351	0.091874	-0.09567	0.019308	-0.11761	0.062075	-0.023	-0.02941	0.02141	-0.02553	0.007745	0.001524	-0.03018	0.04508	-0.10342	-0.01742	0.07017	-0.0128	-0.02771	-0.07707	-0.02601	0.082226	0.079087	-0.05741	-0.02077	-0.04368	-0.02673	-0.14232	-0.0254	0.01421	0.075005	0.011625	0.054438	0.076321	0.004483	0.037865	0.019697
intake136	-0.03172	-0.20055	-0.08022	-0.01906	-0.02177	0.028012	-0.09361	0.070452	0.146331	0.069654	0.15051	0.081887	0.012774	-0.15246	0.32689	-0.05747	-0.17397	-0.08421	0.076251	0.158217	-0.1172	-0.11679	-0.03081	0.06523	-0.04595	-0.05086	-0.01897	-0.02784	0.10929	0.108962	0.1098	0.044617	-0.15617	-0.10161	0.034688	-0.07306	-0.07331	-0.15557	-0.1007
intake137	0.077919	-0.07583	-0.06743	0.154374	0.004904	-0.13267	-0.02503	0.152859	-0.06871	-0.05584	-0.04055	0.019393	-0.00823	-0.15101	-0.04531	-0.11863	0.073602	-0.10455	0.087522	-0.07498	-0.08316	0.077805	-0.03166	0.025763	-0.13823	-0.18065	0.085926	0.030815	-0.23106	-0.11513	0.100391	0.000895	0.034097	-0.12922	-0.14656	0.145806	0.018723	-0.02634	-0.05471
intake138	0.087115	-0.06972	-0.00544	0.164814	-0.0615	-0.09435	0.103074	-0.00252	-0.00118	0.187995	0.080481	-0.10011	-0.06603	-0.10072	-0.03912	0.08197	-0.07033	0.055961	-0.16814	-0.03278	0.009465	0.054199	0.129839	0.084047	-0.03744	-0.16544	-0.0923	-0.12348	0.068998	-0.16414	0.000469	0.001373	0.023958	-0.20215	0.058142	0.07149	-0.08022	0.125022	-0.10889
intake139	0.019102	0.025707	0.165146	0.078948	0.054324	0.091837	-0.21553	0.053751	0.087912	-0.00176	-0.01825	0.055761	0.01403	-0.1087	-0.00444	-0.0054	0.095073	0.096434	0.03659	0.01238	-0.07181	-0.10735	0.095497	0.037898	0.006066	0.136679	-0.16798	0.110498	0.127474	-0.00979	-0.13276	0.020864	0.102411	0.060084	-0.18318	0.032728	-0.08481	0.065525	-0.10719
intake140	-0.02556	-0.08204	-0.12974	-0.00827	-0.03629	-0.01496	0.16692	-0.01411	-0.025	-0.04958	0.028153	-0.12107	-0.08077	0.206712	-0.05111	-0.06473	-0.02826	0.19911	-0.08309	-0.05478	-0.09903	-0.3119	-0.154	0.128346	-0.07131	0.099221	0.073759	-0.03139	0.093198	-0.08918	0.006278	-0.13735	0.238213	-0.10818	-0.16409	0.086054	0.020046	0.080417	-0.01702
intake141	-0.04504	-0.1037	0.164527	0.037174	-0.02761	0.053035	-0.16964	0.002938	-0.06364	0.062205	0.004396	0.020421	-0.06375	-0.05362	-0.06253	-0.09979	0.069448	-0.08827	0.076094	-0.0869	-0.02361	-0.04335	0.152613	-0.0154	-0.00725	0.097592	0.040073	0.049999	0.09744	-0.10755	-0.0324	0.010995	-0.03102	0.099581	0.007116	0.04943	0.040437	-0.0459	0.046514
intake142	-0.03969	-0.14565	0.204306	0.045148	-0.0278	-0.01161	0.027038	0.051587	-0.00799	-0.03462	-0.08839	-0.11897	0.013264	-0.08236	-0.01567	0.018627	0.029529	0.04777	0.080464	0.039329	0.024439	-0.0477	0.124866	0.030902	-0.12169	0.039935	-0.09902	0.173229	0.065438	-0.10356	-0.02182	-0.04815	-0.06973	-0.03447	0.059138	-0.0396	0.023823	0.039358	0.011803
intake143	0.075884	0.143765	-0.05599	0.121675	0.008552	-0.06253	-0.16187	0.133602	-0.06078	-0.01212	0.04244	0.090347	-0.02794	-0.06933	-0.13608	-0.02472	-0.08776	0.075267	0.084228	-0.00836	-0.05163	-0.07718	0.032969	0.256413	-0.0616	-0.02538	-0.02076	0.093323	0.026439	0.131538	0.020417	0.081661	0.025711	-0.05911	0.12385	0.042684	0.01677	-0.12535	0.045671
intake144	0.056605	-0.0751	0.039	-0.06995	-0.13187	0.048029	-0.07814	-0.12789	0.168072	0.178334	-0.01978	0.021571	-0.01537	-0.21735	-0.05612	0.024448	0.12993	-0.02468	-0.03088	-0.10108	0.119249	0.026121	-0.03977	-0.10348	0.092863	-0.07369	0.081982	0.110243	-0.12758	0.012246	-0.11523	-0.05792	0.067147	-0.09751	-0.13572	0.102694	-0.05045	0.127	-0.05295
intake145	0.18208	-0.00355	0.103819	-0.06315	-0.07856	-0.06911	-0.13306	0.059831	0.044252	0.152881	-0.00536	-0.06047	0.011734	0.088288	0.058487	-0.06894	-0.16135	0.100996	-0.03225	0.060771	0.080906	-0.0877	-0.03281	-0.02131	0.14759	0.019499	-0.11959	-0.07696	-0.01602	0.054479	0.018923	-0.08307	-0.0114	0.141814	0.050475	0.06593	0.078546	0.054626	-0.08719
intake146	-0.02341	0.045647	0.032219	-0.04927	-0.10579	0.159075	-0.07542	0.028723	0.008278	0.104679	-0.0725	0.04753	-0.1066	0.196852	0.043156	-0.08949	-0.02888	-0.05155	0.024609	0.081301	-0.01862	-0.0113	-0.04934	-0.21308	-0.07687	-0.2314	-0.02	-0.0244	-0.0533	0.083805	0.048407	0.000717	0.234907	0.107683	0.058306	-0.04997	0.053296	-0.23558	-0.03497
intake147	0.08008	-0.16481	0.110917	0.028099	0.020346	0.076192	-0.02439	-0.03375	0.009954	0.076628	0.203248	0.184779	0.048166	0.00534	0.040956	0.095446	0.062606	-0.05467	-0.09794	-0.00956	-0.07964	-0.1223	-0.09079	-0.09392	0.047864	0.067535	-0.05779	0.012718	-0.04507	0.022706	-0.18263	-0.09578	-0.08478	0.103649	0.041853	0.031123	0.015351	0.064436	0.075011
intake148	-0.05149	-0.21107	0.05851	0.073465	-0.10987	0.035658	0.001658	-0.01451	0.047893	-0.02754	-0.02861	0.004319	-0.00055	0.043862	0.066015	0.225229	0.124842	0.046573	0.035947	-0.21848	0.063585	-0.03192	0.181224	0.124201	0.108621	0.03994	0.107221	-0.02733	-0.01409	0.016913	0.067018	0.014732	0.101743	0.04132	-0.1212	-0.00779	-0.06599	-0.00752	-0.14941
intake149	0.07097	-0.16708	0.079627	-0.01126	-0.01574	0.067857	-0.00513	0.027496	-0.10611	-0.04862	-0.0736	-0.01857	0.047215	0.057439	0.016141	0.020307	0.008481	-0.10488	-0.02547	-0.13237	0.032468	-0.03125	-0.00351	-0.04708	-0.1242	-0.16573	-0.31266	0.051046	0.047528	-0.0353	-0.00639	-0.06147	0.00991	-0.01043	0.000163	0.128234	0.074057	-0.04639	-0.07607
intake150	0.194703	-0.08259	-0.05244	-0.01597	-0.00149	-0.0215	0.038071	0.089313	0.002885	-0.01353	-0.04233	-0.05829	0.073314	-0.0072	0.07718	0.114836	-0.00516	0.005238	0.080065	-0.03712	-0.17395	-0.01884	-0.01926	0.046461	0.110164	-0.06892	0.001935	-0.02105	0.07533	-0.00333	-0.14262	0.078852	-0.03432	-0.03353	0.140459	0.112798	0.035149	-0.08746	0.079137
intake151	0.055868	-0.07556	0.028259	0.095562	0.071721	0.139285	-0.04424	-0.10581	-0.03889	-0.04917	-0.11447	0.058204	-0.23036	0.13079	-0.09894	-0.12599	-0.02505	-0.03605	-0.06406	-0.07297	-0.02736	0.15602	-0.10502	-0.04344	0.177293	0.075131	-0.00259	0.256648	0.178693	-0.00722	0.013579	0.079618	0.062477	-0.16241	-0.01825	0.049851	-0.04318	0.051668	-0.00118
intake152	-0.03254	-0.06741	0.090384	0.12212	-0.12576	0.120975	-0.0734	0.027465	0.016044	-0.02237	0.027054	-0.10282	-0.037	-0.01325	-0.05494	-0.00791	0.041165	-0.00945	-0.19191	0.145372	-0.01046	0.115239	-0.08267	-0.01688	-0.09622	-0.11546	0.13666	0.076671	-0.1397	0.069754	-0.12703	0.029364	0.09384	0.000807	-0.01649	0.027493	-0.03557	0.01134	0.063935
intake153	0.041372	0.028962	0.068807	-0.08853	-0.18874	0.035549	-0.06133	-0.13706	0.088126	-0.14062	0.146029	0.007651	-0.08845	-0.13808	0.050709	-0.09096	-0.02193	0.009158	0.033751	0.122429	-0.10594	0.063454	-0.15717	0.025343	-0.15449	0.082491	-0.09867	0.08183	0.045403	0.089475	-0.11747	0.081379	-0.09085	-0.03074	-0.24463	-0.14228	0.051762	0.018895	0.02014
intake154	0.10568	-0.13335	0.109477	0.032422	0.050603	0.053627	0.070746	0.034757	0.03432	-0.02188	-0.0252	-0.0203	-0.0366	0.056523	0.015432	-0.03002	-0.04084	0.062961	-0.03774	0.090175	-0.05159	-0.05284	-0.0225	-0.06096	-0.02763	-0.1174	-0.11069	-0.09436	0.012566	0.177525	-0.06049	-0.00394	0.105136	-0.08384	0.096534	-0.10837	0.120257	-0.04611	0.13857
intake155	0.060679	-0.10783	########	-0.08103	-0.17615	-0.0539	0.128796	0.064062	-0.01397	-0.05364	-0.13828	0.086849	0.098045	-0.0036	-0.10532	0.171041	-0.03206	0.148039	-0.14148	0.080691	0.046793	0.004272	-0.02295	0.040024	0.001318	-0.1268	-0.21745	0.153575	-0.02414	0.177361	-0.10226	-0.01859	0.071041	-0.07073	-0.12369	-0.02646	0.017486	-0.07966	-0.02398
intake156	0.130005	0.025979	-0.06658	-0.02242	-0.10725	0.06534	0.063962	-0.1467	-0.01412	0.106007	-0.11553	0.121623	0.072208	-0.08467	0.06481	0.000508	-0.05788	0.108062	-0.01055	0.110717	-0.12589	-0.16614	0.023169	-0.02949	-0.10663	0.034177	0.154063	0.019577	0.085158	-0.04511	-0.01395	0.089646	-0.04909	0.087582	0.038727	-0.0156	0.290457	0.042968	0.160397
intake157	0.080392	-0.07186	0.03042	0.029491	-0.23377	-0.03389	-0.09949	-0.01594	-0.22251	0.022997	-0.15351	-0.07237	-0.05971	-0.02831	-0.06221	-0.11504	0.123448	0.090024	-0.01229	0.052171	-0.00222	0.122925	0.109286	0.087038	0.108965	0.020355	-0.03469	-0.02431	-0.19038	0.029339	-0.01471	-0.03464	0.066801	0.090351	0.089599	-0.08526	0.08523	-0.03301	0.016546
intake158	-0.06418	-0.15	-0.07092	-0.00338	0.025306	0.219221	0.029942	-0.13173	0.059722	-0.09864	-0.13641	-0.07895	0.134388	4.53E-02	0.006459	0.096413	-0.03909	-0.02405	-0.02465	0.066481	-0.07693	0.028939	-0.07188	0.231825	-0.05163	0.074781	-0.0373	0.017979	0.003997	-0.02556	0.08735	-0.03786	-0.01874	0.131416	-0.19341	-0.09735	0.093738	-0.01755	0.015383
intake159	0.130555	0.138844	0.270993	-0.0262	-0.10881	0.05301	-0.04299	-0.06135	-0.03309	-0.05909	0.026405	-0.11004	0.115724	0.204425	0.100815	0.034354	-0.04424	0.067622	0.117191	-0.13392	-0.1158	0.016883	-0.06354	0.145829	-0.10432	-0.11598	-0.00832	-0.06289	-0.00104	-0.17258	-0.02204	-0.06144	-0.06028	-0.06173	0.027205	0.098883	-0.24679	-0.02392	-0.11328
intake160	0.07076	-0.11421	0.058201	0.040159	0.008569	0.17671	0.10307	0.025916	-0.00664	-0.07945	0.078149	-0.06539	-0.0444	0.044941	-0.10005	0.090369	-0.03585	-0.02321	-0.08657	0.077118	0.16895	0.086887	-0.24727	0.1443	0.026853	0.045647	-0.13473	-0.0062	0.024775	-0.07392	0.092279	0.027269	-0.00333	0.042132	-0.02623	0.14253	0.001529	-0.05217	0.031665
intake161	-0.00647	-0.09746	0.04191	0.050419	-0.0667	0.195196	-0.06441	-0.11003	0.037512	-0.01346	-0.0492	-0.10724	-0.03274	-0.06971	-0.12543	0.042927	-0.11835	0.028327	-0.10367	0.026617	-0.11199	-0.08859	0.00315	0.011511	0.036045	0.047619	0.182289	-0.14955	-0.02473	0.07322	-0.01184	0.014109	-0.14551	-0.13356	0.070167	0.171633	0.109204	0.088929	-0.15861
intake162	0.097179	-0.02379	0.135496	-0.06742	-0.1195	0.040057	0.081853	-0.22111	0.001589	-0.10712	-0.02913	-0.01237	0.071158	0.054206	0.060628	0.058389	-0.06823	-0.09801	0.044703	-0.1007	-0.014	0.008676	0.007114	0.075677	-0.02728	-0.12494	0.119443	0.033545	0.080446	0.161709	0.095752	0.147777	-0.1029	0.032325	0.080982	0.033692	-0.02345	0.028206	-0.11539
intake163	0.085336	-0.08622	-0.01915	-0.17777	-0.22836	0.121825	-0.08775	0.007332	-0.0439	0.05335	0.095531	-0.01592	-0.09009	0.051448	-0.05381	-0.13759	-0.06053	0.011618	-0.087	0.026493	0.009947	0.151809	0.071871	0.104529	0.041544	-0.01901	-0.05536	-0.17619	0.009418	-0.04954	-0.00906	-0.03122	-0.0921	-0.01273	0.055337	0.014128	-0.04545	-0.05803	-0.1214
intake164	0.030331	-0.15302	0.075754	-0.0294	-0.11238	0.116748	0.126759	-0.00425	0.039993	-0.04044	0.092652	0.016625	-0.02144	0.036712	-0.08135	0.039006	-0.10729	-0.06922	0.037774	-0.18794	-0.07716	0.014471	-0.06074	-0.06303	-0.01072	-0.03219	-0.10594	-0.03181	0.002067	0.111615	0.132303	-0.0684	0.042325	-0.00622	0.02771	-0.06208	-0.18859	-0.01143	0.161812
intake165	-0.03795	-0.1057	0.130733	0.102275	-0.20667	-0.07892	-0.03286	0.006662	-0.01951	0.121897	0.125053	0.139536	0.161289	0.175606	0.01546	0.010552	-0.15086	-0.01265	-0.16346	-0.12128	-0.05977	-0.04736	0.188344	0.017402	0.077768	0.12549	0.147876	0.052581	-0.16462	0.005515	0.114005	-0.03546	0.013372	-0.11318	-0.11121	-0.21283	0.05323	0.022599	0.095765
intake166	0.085942	-0.00047	-0.07862	0.030864	-0.00035	0.110128	-0.13185	-0.01051	0.003805	0.089838	0.015867	-0.00559	-0.05485	0.099223	0.069559	-0.02924	0.075723	-0.18762	-0.00308	0.140121	-0.08835	0.026363	0.134677	-0.09442	0.302298	0.087149	-0.05617	0.019735	0.069713	-0.02235	-0.06656	0.07278	0.048412	-0.16423	0.01947	0.15396	-0.0902	0.163639	0.011914
intake167	0.027666	0.117794	0.064201	0.113737	-0.19182	-0.02851	0.014413	-0.18017	-0.08086	-0.08414	-0.09183	-0.02109	-0.1117	-0.00845	0.180159	-0.00881	-0.06716	-0.01393	0.031357	0.072332	-0.03551	0.073309	0.087656	-0.03192	0.099403	0.13465	-0.14564	-0.05818	-0.08997	-0.15684	-0.03722	0.124127	0.193654	-0.15267	-0.13469	-0.0798	0.093156	-0.321	-0.03358
intake168	0.198369	0.039603	0.035638	0.060702	0.034029	0.023539	0.046769	0.034542	0.169762	0.074372	-0.01941	0.023091	0.018903	-0.04056	-0.0403	-0.08435	0.099395	-0.00524	-0.1051	0.01957	-0.04915	-0.07883	0.010166	-0.093	0.147207	-0.0888	0.024664	-0.06727	0.128609	-0.13414	0.000889	0.098336	0.084179	0.151396	0.18905	-0.11791	0.230823	-0.11601	-0.16118
intake169	0.20495	0.075325	0.120613	-0.04004	-0.03721	-0.12319	-0.08177	0.045885	-0.02864	0.068258	-0.01005	-0.07328	-0.10316	-0.10002	-0.02431	0.103195	-0.05332	0.239004	0.124439	0.047175	-0.0983	-0.05806	-0.0252	0.146922	0.082448	-0.23786	0.006995	0.131365	0.001577	-0.01317	0.093799	0.058365	-0.0102	-0.02388	-0.10058	-0.05067	0.005701	0.11675	0.173639
intake170	0.121068	-0.06125	-0.05931	-0.06365	-0.06476	0.008597	-0.07253	0.03237	0.093507	-0.10788	0.019984	0.256852	0.15114	-0.03555	-0.20576	0.001112	-0.14276	0.093366	-0.05698	0.109303	-0.02433	0.182459	0.101844	-0.06458	-0.02936	0.084812	1.65E-02	-0.28559	0.032857	-0.05921	-0.07345	0.065356	0.04715	0.064938	-0.12253	-0.06236	-0.13993	-0.0457	0.038691
intake171	-0.01991	-0.12589	-0.0952	-0.02804	0.081359	0.035238	-0.00384	0.031047	-0.08065	0.090135	-0.02647	0.131353	-0.14021	0.005861	0.106541	-0.05477	0.123037	0.364824	-0.15306	-0.14518	0.067879	-0.03491	-0.1027	-0.0237	0.017617	-0.22377	0.055507	0.077736	0.08302	-0.14042	-0.21281	0.202399	0.070734	-0.02864	-0.04857	-0.15277	-0.11456	-0.13933	-0.14173
intake172	0.143831	-0.0352	-0.14819	-0.20498	-0.07721	-0.02106	-0.08665	0.086366	0.184264	-0.03503	-0.08512	-0.09018	-0.12359	0.116849	-0.04459	-0.08604	0.124721	-0.04333	0.00591	-0.09135	-0.0346	0.009399	0.122947	0.020484	0.035101	0.029175	-0.01489	0.138844	0.044409	-0.19414	0.057702	-0.1191	-0.11584	0.028997	0.027171	-0.06763	-0.07185	-0.02114	0.250596
intake173	0.054222	-0.01828	-0.0869	-0.09062	0.012217	0.087001	-0.08633	0.052524	0.099267	-0.07247	0.093076	-0.14541	-0.01751	0.126917	-0.10372	0.174499	-0.11648	0.063113	0.164845	0.059269	0.022967	-0.10249	0.195703	-0.03435	-0.04442	-0.14928	0.219807	-0.07281	-0.006	-0.14478	-0.16619	-0.1005	0.00644	0.01725	-0.1808	-0.04273	0.126838	0.007107	-0.07818
intake174	0.083689	0.023883	0.059107	-0.12065	-0.00405	0.062109	-0.01685	0.211315	0.032545	-0.14295	0.080452	-0.03792	-0.05664	0.004409	0.174079	-0.11816	0.125452	0.034935	0.032835	-0.0461	-0.01332	0.105452	-0.03414	0.102496	0.018531	-0.05728	-0.01115	-0.1065	0.041545	0.225986	-0.22012	-0.05283	0.275901	0.0565	0.096259	0.107643	0.048063	0.20548	0.050941
intake175	0.198238	0.000712	-0.03222	-0.19243	0.042161	0.033472	0.047572	0.031759	-0.0239	0.074017	-0.04186	0.149765	0.039567	0.000364	-0.06978	0.024756	-0.07533	-0.03208	0.106784	-0.03878	0.018689	0.011265	0.00212	-0.00605	0.04866	0.118093	0.079695	0.041266	0.032484	-0.13042	0.011275	-0.06074	0.122776	0.03785	-0.00624	0.040752	0.125977	0.037819	-0.14927
intake176	0.044175	0.02135	-0.01678	0.145796	0.033322	0.027089	-0.13582	-0.15043	0.205995	-0.04843	-0.04037	0.125654	0.032665	0.136756	-0.18428	-0.03246	0.10255	0.023128	-0.08345	0.090093	-0.09644	-0.1093	0.053524	-0.04779	-0.1602	0.004238	-0.11642	0.074234	-0.04173	-0.07658	0.052589	0.079329	0.121795	-0.12447	0.019251	-0.01214	-0.05807	0.101646	-0.07101
intake177	0.056332	-0.17076	0.058571	-0.02789	0.059363	0.083403	0.108671	0.093343	-0.02181	-0.02458	0.086903	-0.11618	-0.04838	-0.01334	0.089341	0.027361	0.003124	0.060446	-0.01338	-0.09537	0.132172	-0.03223	-0.02554	-0.04252	0.108549	0.152339	0.036374	0.016695	-0.1221	-0.04668	-0.01909	0.083151	0.039308	0.087079	0.149715	-0.03048	-0.10402	0.06466	-0.03869
intake178	0.022972	-0.07332	0.008127	0.085002	0.144141	-0.05277	-0.1419	0.098845	0.127338	0.022723	0.032407	-0.09658	-0.08514	-0.27823	0.106419	-0.00168	-0.3088	0.049018	-0.12255	-0.18423	-0.03894	0.128332	0.077738	-0.15336	-0.14001	0.003727	0.044515	0.108371	-0.02108	0.067283	-0.02043	-0.15577	0.058633	0.06117	0.071322	0.134452	0.17476	-0.03231	-0.00566
intake179	0.165617	0.090156	-0.00691	0.015716	0.016918	-0.0208	-0.10765	-0.08869	-0.02187	-0.24753	0.078141	-0.09117	0.081932	0.038278	-0.00475	-0.03705	-0.17874	-0.13045	-0.08465	-0.04295	0.20499	-0.04771	0.025305	0.067248	-0.07269	0.012138	-0.09006	0.041696	0.015857	0.01645	-0.08436	0.139598	-0.02927	0.115664	0.01815	-0.05615	-0.13421	0.05421	-0.05613
intake180	0.056392	0.041364	0.088996	0.048903	0.135823	0.047635	-0.06488	-0.16112	0.186324	0.190122	-0.14519	0.012634	0.083806	-0.06435	0.000912	-0.16565	-0.02001	0.060945	0.190594	-0.08266	0.159099	0.007216	-0.02755	0.015528	-0.04635	0.025319	0.0108	-0.11187	0.103805	0.142131	0.166489	-0.08054	0.120534	-0.14307	-0.1454	-0.12461	-0.07949	-0.02086	0.033108
intake181	-0.0095	-0.03197	-0.1065	0.008135	-0.01974	-0.00889	-0.16247	0.068378	0.074739	0.152859	0.034059	-0.13257	-0.17131	0.173943	-0.03519	0.085029	-0.0783	-0.17475	0.033599	-0.08647	0.065655	-0.03004	-0.01069	-0.0216	0.092272	0.036233	-0.22864	-0.12622	0.045217	0.003973	-0.08283	0.309813	-0.0572	0.059671	-0.15111	-0.02561	0.094222	0.014839	0.209881
intake182	0.038076	-0.01777	0.033865	-0.20224	0.063226	-0.03148	-0.08396	-0.15251	0.072506	0.149903	-0.02673	0.117958	0.126411	0.046656	-0.0221	-0.11775	-0.06109	-0.1027	0.025637	-0.16106	0.114464	0.046589	-0.04443	0.323256	0.106932	-0.20555	0.065922	0.121536	0.035299	0.01287	-0.0156	0.075786	0.13145	-0.10707	-0.00748	-0.04971	0.160457	0.005506	0.08001
intake183	0.049781	-0.06931	-0.04859	0.060495	-0.08769	0.046635	-0.12833	-0.1319	0.01031	0.022732	0.337455	0.054394	0.078576	-0.05991	-0.11309	-0.12715	-0.14939	0.151116	0.134587	-0.27156	-0.0878	0.029383	-0.21682	-0.09814	-0.07969	0.113683	-0.03052	0.076806	-0.09088	-0.0195	-0.08531	0.034821	0.027148	0.062293	0.039577	0.056314	0.06532	0.04747	0.094073
intake184	0.052834	-0.14898	-0.15846	0.141708	0.132511	0.109351	0.111448	-0.0201	-0.01815	0.015386	0.122316	4.08E-05	-0.20129	-0.0229	0.077532	-0.16039	0.019393	0.199155	0.225084	0.010324	0.002553	-0.11376	0.148645	0.131793	0.008435	0.131115	-0.15689	0.073959	-0.1245	0.121377	0.262914	0.100521	0.058405	0.075459	-0.01069	-0.05513	0.021371	-0.00762	-0.11928
intake185	-0.01919	-0.1349	0.000858	-0.13113	0.023182	0.11442	0.004997	-0.09634	-0.04887	-0.16775	0.111942	0.125194	-0.00398	-0.04957	########	-0.03621	0.077604	0.183698	-0.11993	0.046777	0.003373	-0.15825	0.156809	0.029744	-0.08421	-0.07202	-0.09354	-0.09202	0.057644	-0.06437	0.06109	0.067572	-0.05307	-0.13225	0.254678	0.111586	0.010201	0.092962	0.062949
intake186	0.064693	-0.00085	-0.04089	-0.01201	0.056771	0.127298	-0.14977	0.024621	-0.10567	-0.19897	-0.09233	0.238945	0.063593	0.057414	0.181854	0.250757	-0.10745	-0.08962	-0.07108	-0.06927	-0.00423	0.066068	0.030658	-0.10701	-0.09923	-0.0019	-0.07848	0.199873	-0.06834	-0.07462	-0.02043	-0.04163	0.050329	-0.01654	-0.06918	-0.12418	0.172203	0.097927	-0.03621
intake187	0.024994	-0.14102	0.028183	0.105711	0.075912	0.023092	0.020115	0.003879	-0.00717	0.087551	0.104346	-0.13876	0.136229	0.172886	-0.11754	0.028019	0.080671	0.092227	0.227889	-0.00312	-0.00316	0.232833	0.029388	-0.26976	0.065058	-0.07072	-0.0931	0.005995	0.012769	0.045927	0.119921	0.000618	-0.05399	-0.10226	-0.05124	-0.01772	0.178025	0.031696	-0.09591
intake188	0.048646	-0.01186	-0.1661	0.123111	-0.09557	-0.04202	0.102027	0.013493	0.031036	0.120559	-0.06428	0.0555	0.070952	0.101012	0.051119	0.104533	-0.05595	-0.05342	0.012264	0.025565	0.215304	0.010455	0.090839	0.20045	-0.11143	0.085342	0.005673	0.019323	0.105625	0.161149	-0.23628	-0.06168	-0.00282	-0.04486	0.131696	0.097073	-0.01067	0.002399	0.036363
intake189	-0.06758	0.047084	0.073309	0.021862	-0.06149	0.174497	0.003065	0.271789	0.023603	-0.00799	0.028728	0.164427	-0.00783	-0.04112	0.092485	0.041536	0.054007	-0.04552	-0.06019	-0.03495	-0.10315	-0.11256	-0.0922	0.03706	0.0335	0.013988	0.130014	0.003497	0.192028	0.019717	0.102612	-0.20451	0.061841	-0.17239	-0.06371	-0.05879	-0.13097	-0.15197	0.086808
intake190	0.301789	0.250707	0.25564	0.219294	0.144586	0.072703	0.144492	0.077301	0.034022	0.054003	0.091648	0.043773	0.021986	0.068387	0.033121	0.100701	0.008635	-0.02324	0.048715	0.058435	0.012876	0.043735	-0.00526	0.054227	-0.00485	0.007884	-0.01348	0.022838	0.012305	0.015184	0.012395	-0.02362	0.010236	0.008743	-0.01703	-0.02385	0.030352	0.019642	-0.02016
intake191	0.144894	0.227095	0.125402	0.153136	-0.00816	0.108079	0.03158	-0.04566	0.031769	-0.07907	-0.05373	0.057636	0.091274	-0.04181	0.101953	-0.17659	0.069463	0.154515	-0.13007	-0.17806	0.099586	-0.04303	0.122723	-0.0932	-0.00354	0.028363	0.014426	-0.11148	-0.15733	-0.00784	-0.01921	-0.01307	-0.13377	0.018155	-0.07902	0.177867	-0.10719	-0.00861	0.119259
intake192	0.180724	0.002002	-0.04898	0.042862	-0.05754	-0.07524	0.032058	-0.08041	0.188555	0.089767	0.123362	0.001605	-0.05466	0.001412	0.077297	0.103476	0.047013	-0.06455	0.110426	0.111847	-0.08554	0.046341	0.006703	-0.04578	-0.01595	0.060267	-0.05874	0.057369	0.091938	-0.03048	0.03585	-0.12225	0.084936	-0.09265	0.127577	0.07301	-0.00099	-0.10517	-0.07029
intake193	0.042195	0.087308	-0.13035	0.025615	-0.09731	-0.01037	-0.14786	-0.15201	-0.02796	0.015964	-0.09002	-0.02477	-0.19005	0.077751	0.257018	0.094928	0.105618	0.178247	0.072238	-0.06062	0.187987	-0.03554	-0.21241	-0.05035	-0.10653	0.138804	-0.04793	-0.03231	0.03046	-0.04456	-0.00627	-0.08808	-0.15491	0.082542	0.088393	0.087985	-0.03418	-0.05354	0.022574
intake194	-0.02769	-0.06602	-0.109	-0.04232	0.012741	0.127016	0.087718	0.096665	0.090092	0.240997	-0.14385	0.123476	-0.0123	0.042305	0.049425	-0.09619	-0.23707	-0.10244	0.125496	0.048151	0.035987	-0.05849	-0.07305	0.052808	-0.09176	-0.02522	-0.00572	0.120783	-0.23475	-0.1162	-0.08731	0.113014	-0.04086	0.143954	-0.10804	0.174994	-0.23533	0.043865	-0.09047
intake195	-0.06396	0.045228	-0.02751	-0.11343	-0.04474	0.153202	-0.09269	0.135493	0.04031	0.201283	-0.02316	-0.01159	-0.05274	0.0503	0.032605	0.14059	0.122406	0.118402	0.03164	-0.00415	0.02747	0.148682	0.020257	0.004368	-0.14285	-0.02824	0.054224	-0.11669	0.104304	0.124729	0.066985	0.025239	-0.07131	-0.00817	-0.20429	0.051171	0.11221	0.256868	-0.11782
intake196	-0.13133	-0.03561	0.045583	0.196913	-0.02793	0.059942	0.051188	0.135167	-0.18406	-0.02557	0.009372	0.033572	-0.05165	0.054644	-0.05706	-0.06947	-0.09022	-0.05957	0.046304	-0.00041	0.026671	0.092546	-0.0717	-0.12042	0.059979	-0.1051	0.100148	0.053269	0.106181	0.008801	-0.1355	-0.01433	-0.07678	-0.00798	0.06212	-0.22446	-0.07516	0.087827	-0.0225
intake197	0.112925	0.067722	-0.11166	0.085071	-0.09791	0.053308	0.015589	0.113236	0.075606	0.049028	0.059687	0.057502	0.056568	0.008177	0.130164	0.036543	0.280303	-0.12173	0.026063	-0.17871	0.074055	0.010482	-0.00825	0.062898	-0.16387	0.015284	0.147361	0.128537	0.018016	-0.0262	-0.06157	0.163923	-0.0195	-0.00207	0.019171	-0.08777	0.065825	0.028778	0.066488
intake198	-0.00393	-0.03419	0.024241	-0.09901	-0.23475	-0.10082	0.103565	0.091594	0.180978	-0.02782	-0.08113	-0.09267	0.041637	-0.0262	0.033621	-0.03833	-0.05177	-0.04407	0.22028	-0.10556	0.140299	-0.14495	0.042646	-0.18385	-0.08606	-0.02798	-0.04764	-0.08079	-0.18441	0.011265	-0.10794	0.093448	0.091695	-0.09536	0.043097	-0.1013	-0.05877	-0.05214	0.027309
intake199	-0.04525	-0.04712	0.141028	-0.09902	0.009522	0.134535	-0.04727	0.165299	-0.05458	-0.00783	-0.01774	0.11595	-0.02543	-0.01185	0.140224	-0.08107	-0.08547	0.016848	0.000397	0.067882	0.206791	0.050398	0.15401	0.155903	-0.03191	0.074714	-0.02772	0.014582	0.056662	-0.05647	0.059307	-0.00041	0.114569	0.012708	0.03906	-0.08274	0.007221	0.079442	-0.0052
intake1100	0.100577	0.00862	-0.10715	0.023421	-0.1051	0.043848	0.033236	0.180727	-0.00614	-0.10924	-0.11744	0.03672	0.282036	0.033422	-0.17199	0.008594	0.077184	0.126723	0.161401	-0.00609	-0.08237	-0.0208	0.004931	-0.00314	0.197558	0.127804	-0.02239	0.157872	-0.02585	0.098531	-0.01016	-0.01717	-0.10258	0.10717	0.032154	-0.02431	-0.06871	-0.02612	-0.23011
intake1101	-0.03071	-0.04577	0.032491	0.023612	-0.13632	0.054229	-0.05054	0.165146	0.275666	-0.04115	-0.11605	-0.04876	-0.10916	0.066864	-0.04492	-0.15993	0.05083	-0.02718	-0.06749	0.01682	-0.14893	-0.02777	0.045521	0.021641	-0.13812	0.084563	-0.05887	0.062571	-0.11531	0.120906	0.042396	-0.02877	-0.08479	-0.12637	0.109754	-0.15299	-0.03981	0.155683	-0.03521
intake1102	0.002864	-0.02814	0.182594	-0.03761	-0.11729	0.13548	0.080435	0.062087	-0.00314	0.104627	-0.06666	-0.14116	-0.03568	-0.04763	-0.02449	-0.0333	-0.07403	-0.04805	-0.0026	-0.08935	0.060735	-0.16138	-0.19727	-0.0477	0.144081	0.050961	-0.01325	0.168419	-0.03466	-0.04354	0.013201	-0.16175	-0.137	0.001159	-0.02978	0.035452	0.107679	-0.13016	-0.05234
intake1103	-0.00191	0.016536	0.060358	-0.0252	-0.0312	0.023653	-0.15543	0.055998	-0.23094	0.076663	0.039689	-0.09842	0.10321	0.037997	-0.03423	-0.08089	-0.05198	-0.08378	-0.1221	-0.04315	0.132161	-0.18064	-0.0168	-0.19026	-0.18671	0.082191	0.057681	-0.09433	0.242722	0.04802	0.079361	0.104839	0.086251	-0.04092	-0.07517	-0.0536	-0.03151	0.024801	-0.1384
intake1104	0.126905	-0.02706	0.010329	-0.12664	0.081176	0.049255	-0.06909	0.012676	0.081248	0.026957	0.046519	-0.08893	0.110227	-0.09962	-0.08502	0.043199	0.212237	-0.05147	-0.14722	-0.03329	0.175555	0.026374	-0.1508	0.048413	-0.04741	-0.02611	-0.00026	-0.09587	-0.10216	-0.07838	0.256795	0.031915	-0.06904	-0.10875	0.054228	-0.12552	-0.02908	-0.10858	-0.02132
intake1105	0.119173	0.054638	-0.13052	-0.04088	-0.17802	-0.01871	0.078095	-0.11975	-0.09509	0.026164	0.015281	0.101655	-0.05013	0.156112	0.054508	-0.01604	0.066692	0.009542	0.019088	0.005701	-0.02244	-0.08735	0.115687	-0.18234	-0.03514	-0.00543	0.00464	0.00176	0.011569	0.10237	0.008998	-0.08213	0.097824	-0.07727	-0.03446	0.131681	-0.11311	0.028044	0.080688
intake1106	0.009078	-0.07452	0.019442	0.107351	-0.03689	-0.03043	-0.13811	-0.16634	-0.03364	0.24047	0.123731	-0.08046	0.048272	-0.04305	-0.1233	0.122878	0.123579	-0.13417	-0.04999	0.173806	-0.0185	0.001201	-0.03485	0.126914	-0.12678	0.023596	-0.01481	-0.00227	0.023267	-0.02988	0.032824	-0.20196	0.319091	0.309627	0.053923	-0.03104	-0.18736	-0.05139	0.05374
intake1107	0.101649	-0.17167	-0.03627	-0.02017	-0.01177	0.021242	0.065875	0.027067	-0.13786	0.018701	-0.09863	-0.04449	-0.03145	0.005098	-0.00739	-0.07344	-0.01875	-0.05835	0.040463	0.079575	-0.15749	-0.10268	-0.01965	-0.07879	-0.03529	-0.01044	0.248792	-0.01573	0.015118	-0.05028	0.072198	0.097815	0.066719	0.146342	-0.03694	0.104166	-0.08674	-0.06578	-0.00389
intake1108	-0.041	-0.10423	0.138826	-0.02514	-0.03773	-0.02848	0.052801	0.001919	-0.03613	0.047531	-0.00633	-0.01485	0.082855	0.087261	-0.03899	-0.1565	0.007968	0.020496	0.163411	0.125729	0.168371	0.025466	0.214828	-0.05323	-0.28713	-0.0251	-0.02638	0.141185	0.15421	-0.25556	-0.00654	-0.02218	-0.07548	0.00783	0.105603	0.048078	0.018273	0.030532	0.067668
intake1109	0.110143	0.131439	0.055971	-0.12418	-0.0627	0.067301	0.07023	0.081967	-0.12154	0.001785	0.224106	-0.00081	-0.25854	-0.0595	-0.24094	-0.01404	-0.0695	-0.04346	-0.0105	0.098388	0.126299	-0.069	0.15274	0.072304	-0.03909	0.027875	0.135635	0.049655	-0.07911	0.160083	0.005845	0.100334	-0.06075	-0.0807	-0.18003	0.11892	0.016467	-0.13551	0.013389

Factor40	Factor41	Factor42	Factor43	Factor44	Factor45	Factor46	Factor47	Factor48	Factor49	Factor50	Factor51	Factor52
-0.00435	-0.09821	0.190757	0.008772	-0.09929	-0.04272	-0.11918	-0.06939	0.025571	0.266159	0.202359	-0.01168	-0.0268
0.093137	0.033909	-0.03255	-0.13138	0.008727	0.004727	-0.09892	0.133445	-0.002	0.04225	-0.02362	0.010067	0.075996
0.075906	-0.03001	0.065864	-0.10229	-0.1694	-0.13185	-0.05728	0.015703	0.093031	0.077102	0.121309	-0.10106	-0.1206
-0.06546	-0.13692	0.118719	-0.05081	0.080789	0.093148	-0.04568	0.055261	-0.03698	0.060425	-0.0643	0.014384	-0.05236
-0.04882	-0.10071	0.142353	-0.00866	0.023611	0.016328	0.049825	0.001516	-0.00598	-0.21872	0.138196	0.05214	0.00481
-0.00148	2.20E-05	0.022787	0.01672	0.088273	0.053004	-0.08755	-0.03475	0.130049	0.149786	0.03881	0.140775	-0.01102
-0.05417	0.110636	0.088664	0.001989	-0.0154	0.031979	0.012159	-0.14218	-0.05044	-0.07021	0.058483	-0.20072	-0.13503
-0.18085	0.115304	0.016757	-0.03084	0.000283	0.19227	0.046897	0.056392	-0.12723	0.011121	-0.02011	0.058201	-0.11928
-0.06113	-0.00197	-0.04963	-0.02387	0.022504	-0.0775	0.018701	0.029761	0.134087	0.062145	0.103362	-0.00726	0.079794
-0.04318	-0.00376	-0.00978	0.014072	-0.09063	-0.06475	-0.11445	-0.06232	-0.12023	-0.01132	-0.06565	0.017902	-0.07398
0.0146	0.062847	-0.02497	0.155749	-0.14078	0.091577	-0.04404	0.004895	0.102092	-0.08299	0.111086	0.05356	0.045642
-0.02667	0.030269	0.100866	-0.06454	0.154309	-0.08714	-0.06205	-0.07283	-0.22374	-0.07913	0.009801	-0.05071	0.033834
0.105824	0.097797	0.0408	0.110462	-0.11669	0.079443	-0.09263	-0.02013	0.107682	-0.05588	-0.06653	-0.09104	-0.01088
-0.03618	-0.02321	-0.00459	0.023041	-0.08781	-0.12554	-0.08646	0.02074	0.018222	-0.05847	0.176159	-0.04568	-0.05057
-0.06331	-0.01443	0.097339	0.220772	-0.05324	-0.05921	0.210059	0.190804	-0.05877	-0.0412	0.049472	-0.07709	-0.00243
-0.19859	-0.06092	-0.0518	-0.11586	-0.03839	-0.04116	0.121432	-0.04372	0.024593	-0.03933	0.14325	0.119839	0.014634
0.01988	-0.08974	-0.03916	-0.07843	0.00139	-0.03098	-0.07247	-0.05303	0.092015	-0.01224	0.078135	-0.17034	-0.08366
-0.03874	-0.05453	-0.02326	-0.09119	0.112732	0.031726	0.127898	-0.05717	-0.07503	0.030574	0.061229	-0.08173	0.064626
0.064373	-0.08329	-0.15265	0.139159	0.047942	-0.01946	-0.09665	-0.03092	-0.01942	0.040697	-0.05547	-0.08282	-0.03289
-0.00868	-0.1019	0.067849	0.140913	0.120504	-0.10102	-0.03139	0.089954	0.075425	-0.00901	-0.09238	0.001846	0.041672
-0.0831	-0.02476	0.039881	-0.0312	0.130697	0.019004	-0.04424	-0.10801	-0.00887	0.105352	-0.04062	-0.08067	0.016672
0.087897	0.072838	-0.11771	0.128068	-0.20493	0.02379	-0.01905	0.028871	-0.06385	0.095826	0.020326	0.026839	0.01054
-0.2007	-0.09391	0.10899	7.69E-05	-0.02702	0.121464	-0.09421	-0.09103	-0.04486	-0.0363	-0.12804	-0.00093	0.040292
0.079799	-0.09735	0.089916	-0.06961	0.009622	0.050892	0.051379	-0.09436	-0.06226	0.096289	-0.17863	0.058823	-0.14224
0.232691	-0.05456	-0.00491	0.056933	-0.04291	0.082349	0.01755	0.097771	-0.11962	0.047542	-0.06887	-0.06131	0.024744
0.122755	-0.07114	-0.07713	-0.00335	-0.0646	0.028911	-0.01608	0.06187	-0.09318	-0.05306	-0.15145	0.102737	0.075755
0.01611	-0.10936	0.006707	0.0218	0.086102	0.084791	0.094572	-0.02555	-0.09299	0.139458	0.141083	0.023561	0.186161
-0.014	-0.02634	0.029663	-0.0089	-0.10809	0.03526	0.08912	0.024743	0.024713	0.016251	-0.03708	-0.11309	0.110415
0.224437	0.034611	0.01377	0.011115	0.049677	0.085515	-0.00478	-0.00082	0.060664	0.067177	-0.02492	-0.13059	-0.01417
0.031971	-0.00172	-0.08518	-0.04595	-0.23761	0.024437	-0.03272	-0.06259	0.001604	0.080396	0.051206	-0.00057	-0.01505
0.093498	0.065487	0.033182	0.027096	0.039908	0.019815	0.035528	-0.02384	-0.00755	-0.0513	0.102378	-0.03944	0.049061
0.016471	0.099	0.171459	0.070378	0.02341	0.072457	0.045732	-0.14009	0.168779	-0.02539	-0.13665	-0.01208	0.099753
-0.03612	-0.00242	-0.13742	0.013676	0.14949	-0.01227	0.066904	-0.05526	-0.0237	0.062707	-0.08764	-0.18408	0.00904
-0.06605	-0.04724	-0.14866	-0.04528	0.046886	0.016188	0.131872	-0.04555	1.70E-01	0.105652	-0.03615	-0.01747	0.087796
-0.01356	-0.07853	-0.05122	-0.13411	0.02025	-0.02732	-0.05588	-0.14945	0.049343	0.121035	0.014598	0.200292	0.004521
0.067097	0.012432	0.137646	-0.01166	-0.01802	-0.16586	-0.08811	0.103328	-0.02274	-0.10637	0.084685	-0.0687	0.102691
-0.0339	0.089516	-0.01247	0.045213	0.100045	-0.128	-0.1112	0.13019	0.137835	-0.11916	-0.06058	-0.04588	0.004023
-0.04007	0.031272	-0.03329	-0.11573	0.094591	0.054155	-0.02946	0.10357	-0.03241	0.120713	0.024431	-0.04122	-0.12797
-0.17379	-0.02537	0.005052	0.096263	-0.01057	0.093216	0.02672	0.031954	0.044848	0.081869	0.091111	-0.20169	0.04363
-0.07394	-0.06207	0.169401	0.014538	0.03481	-0.23575	0.012815	0.076725	0.038897	0.047475	-0.13258	-0.15886	-0.11846
-0.05379	0.032798	0.049559	0.062102	-0.02909	-0.00257	-0.12749	-0.21395	-0.04261	0.127121	-0.18074	-0.12081	0.107383
0.023907	0.167165	0.041363	-0.08585	-0.03404	-0.05466	-0.06818	0.022167	0.022207	-0.12252	0.023758	-0.1442	0.107965
-0.22088	-0.06623	0.086869	0.069697	-0.01752	0.006594	0.156515	0.114959	0.020676	-0.07101	-0.06626	0.130082	0.013396
0.018131	0.029568	0.151837	-0.02585	-0.03312	-0.09425	0.070235	0.138378	0.017561	0.104016	-0.02076	0.021494	-0.09336
0.026376	-0.10905	0.116762	-0.06486	-0.05561	-0.00739	0.034346	-0.08776	0.118199	0.047987	-0.16167	-0.03059	-0.00818
-0.0685	0.056165	0.025543	-0.07141	0.015586	-0.10206	0.107666	0.025716	0.143858	0.035076	0.215286	-0.12041	-0.02536
0.057967	0.032784	0.103631	0.060845	0.012221	-0.04243	0.033606	-0.10096	-0.04349	-0.02562	0.046834	-0.12277	0.032789
0.073869	-0.02442	0.17057	0.080114	0.076017	-0.11937	0.087643	0.000301	0.001137	0.069156	-0.12123	0.014928	-0.01235
0.160736	0.01388	-0.0192	-0.11194	0.029037	0.224832	-0.0847	-0.07572	-0.04945	-0.00164	0.105512	0.041364	-0.00263
0.07678	0.044742	-0.1487	-0.08303	-0.02653	-0.10103	0.072694	-0.06544	-0.0395	-0.10679	-0.08076	-0.08602	-0.0617
0.099157	0.127046	0.001622	-0.19459	0.092763	0.056291	-0.03584	0.033574	0.12438	-0.13623	-0.15439	0.068785	0.039385
-0.05613	-0.10403	-0.05904	-0.01073	-0.1427	-0.11074	-0.07885	-0.24373	-0.1009	-0.00878	0.008593	0.003171	0.048332
0.049879	-0.03399	-0.06155	-0.00497	0.040133	-0.01335	-0.02733	0.034982	0.045731	0.110542	-0.0145	0.03053	-0.02161
0.002558	0.303746	0.101078	-0.01617	-0.03395	-0.00627	0.11386	-0.01956	0.074978	0.010916	-0.11813	0.125087	0.085407
-0.11133	0.048817	-0.12197	-0.00084	-0.13474	-0.01306	-0.10524	0.023145	-0.05218	0.084337	0.102344	-0.10841	0.174696
-0.04219	0.160795	-0.03864	-0.00885	0.058666	-0.0573	0.009629	-0.0265	0.065941	-0.08929	-0.07162	-0.03366	-0.02188
-0.07048	0.003006	0.052545	-0.16632	6.42E-05	0.07186	0.060314	0.103186	-0.14503	0.046776	-0.0171	0.064487	0.109428
0.023582	0.181998	0.103907	-0.09293	0.169579	0.109102	0.087775	0.211009	0.078684	0.069303	0.078546	0.066144	-0.01615
-0.06657	0.16333	-0.05442	-0.13123	-0.04906	-0.08527	0.07391	0.123076	-0.16537	0.018484	-0.07032	-0.03881	-0.01806
0.082308	-0.11596	0.024637	0.092576	-0.01085	-0.081	0.211122	0.067957	0.047871	-0.02342	-0.16231	-0.03353	0.226737
-0.05491	0.082676	-0.12422	-0.0588	-0.05467	0.154847	0.047965	-0.01395	-0.19333	-0.20163	0.123601	0.005728	-0.12015
0.024027	0.002638	0.066717	0.037929	-0.01771	0.015033	-0.04322	-0.09022	0.064	0.042996	0.085366	0.093594	-0.14286
-0.1017	-0.18525	0.034998	-0.05965	-0.0673	-0.12839	-0.0351	0.041606	-0.06779	0.04135	-0.0169	-0.03198	-0.00862
0.004483	-0.27064	0.125759	0.119377	-0.09241	0.077169	-0.07747	0.04718	0.082151	-0.06914	-0.02702	0.002353	-0.0571
-0.01596	0.04656	-0.11658	-0.00784	-0.08319	0.171449	-0.00299	0.097246	0.105026	-0.01952	0.113429	-0.0246	0.082726
0.087864	0.024389	0.07605	0.012081	-0.04937	-0.13692	-0.00505	0.00262	0.063401	-0.02385	0.173816	0.102948	0.160303
0.137729	-0.01212	-0.0091	0.281705	0.088871	-0.14052	0.04648	-0.11257	-0.01398	-0.10579	0.089279	0.08674	-0.05825
0.039617	-0.10255	-0.05855	-0.03865	-0.00783	0.054798	-0.02669	0.304682	0.060137	-0.01327	-0.15334	0.110431	0.056267
0.05923	-0.03149	0.10632	0.124296	-0.1067	0.005691	-0.17665	-0.07261	-0.01586	-0.09674	-0.00364	0.137304	-0.1453
0.150981	0.179237	-0.11446	-0.01367	0.040592	-0.17951	-0.05197	0.001992	-0.07476	-0.12244	-0.17629	-0.13617	-0.05477
0.080957	-0.0337	-0.01727	0.0172	0.008024	0.107626	0.072891	-0.19125	0.036607	-0.06594	0.09569	-0.05416	-0.0534
-0.05364	0.180078	0.130442	-0.03079	-0.00213	-0.02808	0.126309	-0.07931	-0.00385	-0.05828	0.099008	-0.00165	-0.02149
-0.03219	-0.03655	-0.01356	-0.01522	-0.00285	-0.01019	-0.04194	-0.02836	0.096124	-0.01394	0.15832	0.131248	0.150861
0.04554	0.051305	-0.07514	0.108944	0.092344	0.107559	0.089074	0.113357	-0.01418	-0.079	0.086662	-0.01463	-0.09184
0.01576	-0.05854	0.038816	0.083186	-0.114	0.135598	-0.18212	0.098183	-0.06436	0.142758	0.04873	-0.02479	0.028881
-0.00552	-0.19502	-0.03032	-0.02095	0.054241	-0.107	-0.07764	0.117132	-0.0658	-0.17346	0.040572	0.128222	0.138327
-0.21435	0.023902	-0.19221	-0.12713	-0.08879	-0.25136	0.034475	-0.00518	0.198483	-0.12109	0.0044	0.162907	-0.02828
-0.05619	-0.00877	0.028357	-0.00646	0.165227	-0.14686	0.079481	-0.04495	0.008549	0.100903	0.031193	-0.05616	0.043427
-0.06099	0.046865	-0.06352	0.032007	0.177848	0.031568	-0.1064	0.006074	0.1876	-0.01626	0.088372	-0.17619	0.085023
0.009404	0.110365	-0.19229	-0.08269	-0.01425	-0.08282	0.104462	-0.01704	-0.04021	0.142964	0.034091	0.135525	-0.07469
-0.15617	0.071024	-0.10475	0.083763	0.031136	-0.00812	0.092894	0.148395	-0.0773	0.062537	-0.04579	-0.07372	-0.26611
-0.11745	-0.10798	-0.14747	0.066916	0.130683	0.031418	0.002512	-0.05565	-0.05635	0.017693	0.067295	-0.13944	0.11581
0.199518	-0.00086	0.027276	-0.08408	0.018519	0.026989	0.118213	-0.08791	-0.0391	0.010244	0.084266	0.168628	0.044738
0.032276	0.017669	-0.02425	-0.1729	-0.05239	0.019578	-0.05235	-0.07902	-0.17074	0.077972	0.09437	-0.14452	-0.02829
-0.12773	0.107929	-0.03354	0.233486	0.165345	-0.01378	-0.15844	0.016436	0.185958	0.292423	-0.0304	0.205797	-0.03205
-0.14866	-0.1051	0.079722	-0.1021	-0.08846	-0.04621	0.032841	0.007588	0.050268	0.001169	-0.11569	0.107595	-0.35148
-0.13804	-0.01124	0.060234	0.055836	0.039061	0.021308	-0.20549	0.002029	0.176195	-0.15519	0.001461	-0.18505	-0.14311
0.066509	-0.01988	0.201288	-0.10765	0.077236	-0.05045	0.004772	-0.05033	-0.02	-0.11384	0.147165	0.170058	-0.05852
0.042942	-0.07533	-0.1078	-0.09076	-0.05282	0.126753	0.061573	0.061452	0.146478	-0.04519	0.009371	-0.03268	0.023922
-0.03334	0.008008	-0.05816	########	-0.03146	-0.06304	0.009543	-0.06383	-0.00789	0.010712	-0.01738	0.039698	0.004326
0.029659	0.034019	0.152043	0.042893	0.101227	0.14043	0.019169	0.140529	-0.01757	-0.1204	0.074048	-0.16532	-0.04802
0.04651	0.021572	0.120873	0.048831	0.025673	0.205573	-0.0239	0.017808	-0.04123	0.10213	0.001022	0.008286	0.027148
-0.07203	-0.07634	-0.16228	-0.03582	0.017374	0.024564	-0.21807	0.115247	0.081404	-0.11696	0.099066	0.008995	0.0338
-0.12231	0.226192	0.003627	0.097853	-0.229	0.187596	-0.02716	-0.00622	0.070757	-0.05789	-0.14422	0.11774	0.069802
0.099516	0.007467	-0.02934	0.100395	0.114154	-0.04425	-0.02827	-0.07069	0.005937	-0.15519	-0.03018	0.057914	0.116372
0.039954	0.185669	0.157991	0.05155	0.05719	-0.08875	-0.10107	0.207754	-0.18388	0.165871	0.155051	0.086943	-0.13201
0.059108	0.047375	0.020224	-0.13871	-0.00387	-0.13941	-0.08593	0.074816	-0.02992	0.154067	-0.05461	-0.05691	0.065144
0.028275	-0.11841	0.030077	-0.1713	0.198737	0.048677	-0.09641	0.027831	0.044663	-0.06157	-0.1303	0.034492	0.102848
0.123677	-0.07058	-0.00225	-0.01305	0.058942	-0.06252	-0.16279	0.017542	-0.22532	-0.14939	-0.01382	0.106581	-0.05472
0.091695	-0.01906	-0.10549	-0.04253	0.251667	-0.08609	0.095183	-0.19419	0.004674	0.105054	0.017729	0.036445	-0.06952
-0.07675	-0.08253	-0.14738	0.102098	0.081852	0.16483	0.064697	-0.13786	-0.0196	-0.07695	-0.13205	0.051967	-0.17446
0.039031	0.063153	-0.23166	0.281892	0.137125	-0.16089	-0.15282	0.109467	-0.15158	0.063432	0.068742	0.132216	-0.0109
-0.14179	0.025966	0.054704	-0.07527	-0.00378	-0.01592	0.074584	-0.03066	-0.14804	-0.02115	0.003756	0.042599	0.112456
-0.02253	0.161073	0.155918	0.103782	0.036291	-0.04542	0.055655	-0.22486	0.077306	-0.08361	0.079782	0.123204	0.03269
-0.08278	0.182038	-0.02504	0.135707	0.014609	0.023086	-0.02215	-0.1344	-0.10841	0.132784	-0.07822	0.021164	0.099503
0.027809	0.074356	-0.09789	-0.05819	0.125525	0.042603	-0.16641	-0.06246	0.095316	-0.08936	0.022186	0.036921	-0.22847
-0.02745	-0.14542	-0.02713	0.08818	0.261777	0.157479	0.149142	0.029322	-0.03394	-0.04705	0.141901	0.015355	-0.08806
0.240227	-0.12606	-0.13113	0.149612	-0.2104	-0.07702	0.352261	0.027865	0.113529	0.055825	0.046338	-0.04671	-0.16805
0.233271	0.022434	-0.02162	-0.19517	0.023452	0.052028	-0.04845	0.04212	0.250688	0.094395	0.021187	-0.02928	-0.12569
