# Supplementary material for: Identification of Salty Dietary Patterns of the Japanese Macroregion
Source: J Nutr Metab. 2021 Jul 22;2021:6675418. doi: 10.1155/2021/6675418 (PMC8321765; doi:10.1155/2021/6675418)
Supplement: Supplementary Materials — Supporting File S1: details of 109 foods and calculation units used for nutritional value (data for 2013). Supporting File S2: details of 109 foods and calculation units used for nutritional value (data for 2018). Figure S1: the distribution map of prefectures in Japan. [file 6675418.f1.zip › 6675418.f1/Supporting file S2.pdf]

（品目分類） 第11表 都市階級・地方・都道府県庁所在市別１世帯当たり年間の品目別支出金額，購入数量（二人以上の世帯）

(Commodity Classification) Table 11. Yearly Amount of Expenditures and Quantities per Household by City Group, District and City with Prefectural Government (Two-or-more-person Households)

平成30年 2018

都市階級 地方 都道府県庁所在市 City group District City with prefectural government	世帯数分布（抽出率調整） Distribution of households	集計世帯数 Num. of tabulated households	世帯人員（人） Num. of persons per household (persons)	有業人員（人） Num. of earners per household (persons)	世帯主の年齢（歳） Age of household heads (years old)	102～981 消費支出 Consumption expenditures 金額 Exp.	102～399・39A・39B・39X・39Y 食料 Food 金額 Exp.	1 米 Rice 1kg 数量 Quan.	2 食パン White bread 1g 数量 Quan.	3 生うどん・そ Non-dried "Udon"&"S 1g 数量 Quan.	4 乾うどん・そ Dried "Udon"&"S 1g 数量 Quan.	5 スパゲッティ Spaghetti 1g 数量 Quan.
全国 All Japan	10,000	7,638	2.98	1.35	59.3	3,447,782	952,170	65.75	19,724	10,035	3,552	2,936
札幌市 Sapporo-shi	157	94	2.99	1.28	58.2	3,372,653	893,082	86.31	17,540	8,367	3,690	3,966
青森市 Aomori-shi	23	94	3.01	1.33	60.1	3,017,508	890,844	81.76	17,404	7,211	3,486	2,897
盛岡市 Morioka-shi	23	94	3.09	1.45	58.9	3,440,518	963,319	80.43	17,177	9,640	6,458	3,686
仙台市 Sendai-shi	81	91	3.02	1.26	59.7	3,295,025	941,483	57.90	16,934	8,973	5,205	3,017
秋田市 Akita-shi	25	95	2.90	1.22	59.7	3,360,697	919,370	56.75	14,917	8,965	4,976	2,862
山形市 Yamagata-shi	19	95	3.03	1.55	57.5	3,665,659	958,712	67.13	13,574	11,280	5,777	3,260
福島市 Fukushima-shi	22	92	2.94	1.22	61.5	3,415,902	928,386	65.30	13,173	7,912	5,327	2,612
水戸市 Mito-shi	22	92	2.96	1.33	57.9	3,487,427	934,098	55.28	17,679	9,268	3,460	2,678
宇都宮市 Utsunomiya-shi	42	93	2.87	1.19	59.3	3,677,284	967,570	71.77	18,130	10,053	3,734	2,845
前橋市 Maebashi-shi	27	92	2.73	1.21	60.6	3,190,481	901,904	72.67	13,899	10,983	2,715	2,192
さいたま市 Saitama-shi	103	91	3.06	1.40	58.7	3,887,169	1,042,064	65.46	19,579	11,180	2,961	3,421
千葉市 Chiba-shi	81	93	3.11	1.22	60.3	3,845,361	1,078,992	58.56	19,919	9,764	4,307	3,454
東京都区部 Ku-areas of Tokyo	693	355	2.95	1.42	59.0	3,914,749	1,117,304	53.93	20,476	7,597	3,555	3,550
横浜市 Yokohama-shi	304	127	2.80	1.10	61.3	3,614,844	1,036,897	52.87	19,882	9,632	2,908	3,238
新潟市 Niigata-shi	60	92	3.10	1.49	59.7	3,493,899	966,479	69.50	19,620	9,091	7,008	3,041
富山市 Toyama-shi	32	94	3.20	1.60	56.7	3,893,242	1,061,144	77.22	22,126	10,599	4,304	2,961
金沢市 Kanazawa-shi	35	95	3.30	1.67	56.3	4,070,101	1,009,000	54.11	22,849	13,364	2,779	2,783
福井市 Fukui-shi	20	95	3.17	1.50	60.5	3,400,223	953,432	77.73	17,811	11,013	2,620	2,287
甲府市 Kofu-shi	15	93	2.88	1.43	61.2	3,276,907	930,343	56.11	17,731	9,713	3,142	2,408
長野市 Nagano-shi	31	93	3.01	1.47	60.2	3,460,956	926,326	55.30	18,573	9,777	6,299	3,476
岐阜市 Gifu-shi	32	93	3.16	1.57	59.6	3,865,952	997,596	69.77	22,081	9,134	3,193	2,563
静岡市 Shizuoka-shi	57	95	3.09	1.41	59.5	3,683,278	1,039,277	89.11	18,226	9,680	3,132	3,264
名古屋市 Nagoya-shi	177	105	3.11	1.51	56.3	3,433,756	963,590	68.12	25,181	11,280	3,336	3,823
津市 Tsu-shi	23	90	2.91	1.29	60.3	3,401,318	975,519	57.47	15,569	11,012	4,356	2,886
大津市 Otsu-shi	28	94	2.86	1.14	61.7	3,318,414	1,008,064	72.66	22,235	15,607	3,904	2,872
京都市 Kyoto-shi	113	90	2.85	1.29	62.7	3,376,807	1,004,649	57.39	22,799	12,619	4,399	2,958
大阪市 Osaka-shi	203	126	3.05	1.40	59.7	3,225,215	1,018,324	71.86	24,220	11,506	1,786	2,412
神戸市 Kobe-shi	124	82	2.84	1.19	59.6	3,371,982	961,367	66.17	21,748	11,247	2,734	2,584
奈良市 Nara-shi	30	95	2.84	0.98	61.7	3,688,893	1,030,566	58.91	22,453	11,881	2,996	2,465
和歌山市 Wakayama-shi	30	92	2.72	1.11	63.3	2,698,241	852,941	69.91	23,070	11,308	4,398	1,567
鳥取市 Tottori-shi	15	94	3.30	1.54	57.9	3,203,459	906,974	69.62	25,817	12,178	2,412	3,563
松江市 Matsue-shi	16	93	2.96	1.38	57.5	3,759,872	954,200	58.03	21,468	8,822	3,958	2,706
岡山市 Okayama-shi	55	91	3.04	1.37	57.3	3,490,979	893,087	49.48	21,299	12,569	6,248	2,501
広島市 Hiroshima-shi	95	89	2.99	1.26	58.7	3,387,687	957,488	59.88	21,549	13,608	2,513	2,843
山口市 Yamaguchi-shi	16	94	3.06	1.52	56.7	3,408,272	871,899	63.50	20,346	12,031	2,286	2,530
徳島市 Tokushima-shi	19	92	2.95	1.44	59.7	3,392,785	889,681	55.87	17,153	12,491	3,740	2,348
高松市 Takamatsu-shi	33	95	3.07	1.39	59.1	3,823,350	937,950	63.42	24,813	21,374	5,270	2,152
松山市 Matsuyama-shi	39	92	2.89	1.08	58.2	3,169,288	874,132	59.06	17,108	12,535	3,906	2,890
高知市 Kochi-shi	26	93	3.07	1.50	55.8	3,708,178	997,538	67.17	21,003	9,540	2,014	2,461
福岡市 Fukuoka-shi	112	90	2.87	1.30	57.1	3,871,411	960,329	48.55	15,890	6,220	2,117	2,656
佐賀市 Saga-shi	18	95	2.94	1.32	59.0	3,527,485	932,437	67.11	19,234	7,166	3,831	2,520
長崎市 Nagasaki-shi	35	93	2.72	1.11	63.5	3,257,955	905,588	64.67	19,055	8,185	2,645	2,622
熊本市 Kumamoto-shi	58	87	2.99	1.33	55.3	3,202,427	840,465	58.64	18,206	7,840	2,225	2,651
大分市 Oita-shi	39	93	2.79	1.19	59.7	3,596,129	912,877	66.85	18,991	8,378	3,270	2,445
宮崎市 Miyazaki-shi	33	92	2.84	1.27	59.7	3,005,428	830,026	55.21	16,145	7,122	2,150	2,565
鹿児島市 Kagoshima-shi	48	93	2.88	1.26	59.3	3,159,266	842,475	56.99	16,710	8,195	2,854	2,809
那覇市 Naha-shi	24	150	3.01	1.16	59.0	2,715,399	780,615	65.54	14,112	4,654	2,426	2,343
政令指定都市（都道府県庁所在市を除く） (c)												
川崎市 Kawasaki-shi	116	93	3.12	1.43	54.6	3,770,139	1,044,258	56.95	19,804	8,339	3,497	3,945
相模原市 Sagamihara-shi	59	78	2.99	1.35	61.0	3,391,993	985,913	73.87	21,388	10,766	4,252	4,122
浜松市 Hamamatsu-shi	63	85	2.99	1.38	57.8	3,792,856	944,577	69.99	17,984	6,948	2,652	2,694
堺市 Sakai-shi	69	91	3.06	1.19	59.9	3,460,294	981,113	68.77	21,793	12,970	3,092	2,592
北九州市 Kitakyushu-shi	78	89	2.76	1.09	62.7	3,186,288	895,219	66.00	18,810	8,008	2,617	1,915

都市階級 地方 都道府県庁所在市 City group District City with prefectural government	6 中華麺 Chinese noo 1g 数量 Quan.	7 小麦粉 Wheat flour 1g 数量 Quan.	8 もち "Mochi", ric 1g 数量 Quan.	9 まぐろ Tuna fish 1g 数量 Quan.	10 あじ Horse mack 1g 数量 Quan.	11 いわし Sardines 1g 数量 Quan.	12 かつお Bonito 1g 数量 Quan.	13 かれい Flounder 1g 数量 Quan.	14 さけ Salmon 1g 数量 Quan.	15 さば Mackerel 1g 数量 Quan.	16 さんま Saury 1g 数量 Quan.	17 たい Sea bream 1g 数量 Quan.	18 ぶり Yellowtail 1g 数量 Quan.	19 いか Cuttlefish 1g 数量 Quan.	20 たこ Octopus 1g 数量 Quan.
全国 All Japan	8,562	2,376	2,250	1,929	932	642	815	713	2,507	966	1,135	423	1,617	1,154	539
札幌市 Sapporo-shi	8,598	2,472	2,491	1,937	190	425	440	2,212	3,894	978	2,334	165	613	1,601	1,038
青森市 Aomori-shi	8,867	2,037	1,520	2,091	1,053	1,563	575	2,769	4,086	997	2,084	415	1,183	2,658	663
盛岡市 Morioka-shi	11,401	2,240	1,866	2,349	356	428	1,188	836	3,206	792	3,480	187	1,316	1,109	643
仙台市 Sendai-shi	9,752	2,662	2,803	2,436	406	474	1,764	1,158	2,527	501	1,829	74	1,144	1,162	598
秋田市 Akita-shi	9,472	1,431	1,004	2,284	648	997	910	2,040	2,939	1,024	1,736	450	1,540	1,699	540
山形市 Yamagata-shi	9,391	2,034	2,137	2,032	309	354	1,273	917	2,591	617	1,432	114	1,201	1,437	564
福島市 Fukushima-shi	8,773	1,395	1,681	1,987	435	305	1,273	549	2,247	431	1,244	52	1,060	1,240	303
水戸市 Mito-shi	8,897	1,787	2,360	2,674	619	517	1,699	671	3,583	1,214	2,015	244	1,755	1,338	410
宇都宮市 Utsunomiya-shi	8,687	2,147	2,607	2,794	369	413	751	376	3,078	849	1,273	146	1,293	1,047	381
前橋市 Maebashi-shi	8,950	2,331	2,165	2,660	765	492	618	443	3,747	619	1,180	159	974	1,003	434
さいたま市 Saitama-shi	9,597	2,329	3,352	2,671	718	564	627	295	2,687	953	1,061	168	1,356	1,017	390
千葉市 Chiba-shi	9,097	2,133	2,600	2,902	825	770	1,031	596	3,002	796	1,182	146	1,686	963	614
東京都区部 Ku-areas of Tokyo	8,434	1,826	2,735	2,554	813	471	831	428	2,316	673	976	269	1,421	926	484
横浜市 Yokohama-shi	8,301	2,445	2,487	2,418	1,017	671	616	416	2,680	938	1,236	266	1,586	1,123	524
新潟市 Niigata-shi	8,780	2,090	2,165	1,668	613	844	820	1,641	3,012	827	1,582	114	2,154	1,569	626
富山市 Toyama-shi	8,092	1,454	3,448	2,367	1,273	574	454	779	2,771	934	755	268	4,427	1,964	381
金沢市 Kanazawa-shi	9,882	2,250	2,647	1,275	1,204	721	625	1,165	2,591	843	881	422	3,252	1,924	368
福井市 Fukui-shi	7,369	1,748	1,938	1,254	569	534	481	1,286	2,366	859	731	220	2,417	1,355	788
甲府市 Kofu-shi	8,331	2,244	1,537	3,722	783	524	387	375	2,715	777	996	83	976	1,303	436
長野市 Nagano-shi	9,497	3,760	2,330	1,969	407	503	610	846	3,515	705	1,520	210	1,305	1,124	217
岐阜市 Gifu-shi	9,742	1,598	2,999	2,452	588	395	775	376	2,372	876	994	240	1,679	1,148	596
静岡市 Shizuoka-shi	9,334	1,945	2,302	5,423	967	388	1,080	248	2,219	1,064	1,031	241	1,107	1,329	564
名古屋市 Nagoya-shi	8,547	2,609	2,555	1,943	535	520	507	300	2,417	711	895	128	1,422	871	472
津市 Tsu-shi	6,590	2,053	2,152	2,285	1,115	981	1,071	275	2,756	970	1,328	315	2,545	1,127	508
大津市 Otsu-shi	9,459	2,762	2,684	1,129	1,296	909	1,117	1,190	2,923	1,235	1,663	1,279	2,978	1,197	534
京都市 Kyoto-shi	9,155	2,396	2,217	1,117	1,025	713	971	1,041	2,374	1,021	970	696	1,801	1,294	683
大阪市 Osaka-shi	8,367	2,430	2,309	1,624	675	794	1,057	899	2,087	840	960	880	1,941	1,363	753
神戸市 Kobe-shi	7,878	2,691	2,416	1,372	664	444	853	620	1,919	889	922	781	1,651	793	522
奈良市 Nara-shi	7,722	2,884	2,540	1,146	887	939	900	1,112	2,658	1,051	1,200	580	1,945	1,426	664
和歌山市 Wakayama-shi	7,883	2,451	1,551	1,503	972	749	1,188	538	2,126	1,313	1,089	814	2,233	1,174	594
鳥取市 Tottori-shi	7,272	2,860	1,996	591	1,264	1,640	623	3,575	2,017	1,428	1,235	307	2,802	1,882	475
松江市 Matsue-shi	9,503	1,641	2,077	444	1,893	1,154	523	1,372	2,176	1,580	801	575	2,400	1,583	321
岡山市 Okayama-shi	7,612	1,838	2,439	756	817	576	859	773	2,093	1,103	1,092	404	1,647	843	485
広島市 Hiroshima-shi	8,238	2,415	1,667	580	1,333	950	698	930	2,080	987	1,005	791	1,839	1,218	581
山口市 Yamaguchi-shi	8,001	2,292	1,449	668	1,933	787	930	1,275	2,160	1,305	818	774	2,206	1,319	575
徳島市 Tokushima-shi	7,179	2,657	2,467	1,537	731	364	1,383	286	2,381	874	963	574	2,167	1,064	492
高松市 Takamatsu-shi	7,291	2,398	1,853	700	1,113	522	1,004	778	2,369	972	1,253	670	2,487	887	720
松山市 Matsuyama-shi	7,558	2,723	1,579	889	1,554	521	1,529	383	2,362	945	1,340	1,100	2,058	1,076	574
高知市 Kochi-shi	5,764	1,559	2,042	2,404	1,056	767	4,091	347	1,863	950	906	542	2,777	1,444	548
福岡市 Fukuoka-shi	7,166	2,115	1,700	540	1,445	523	359	694	2,181	1,108	778	989	2,176	960	397
佐賀市 Saga-shi	7,792	2,160	1,239	551	2,156	923	519	959	2,773	1,215	944	1,458	2,137	1,025	256
長崎市 Nagasaki-shi	6,176	2,055	2,969	408	3,137	1,034	682	209	2,303	1,473	511	988	2,614	1,146	286
熊本市 Kumamoto-shi	5,308	1,887	912	664	1,049	685	382	374	1,655	822	615	1,316	1,490	928	385
大分市 Oita-shi	6,413	2,787	1,509	579	2,131	1,179	477	709	1,703	1,403	944	1,019	2,391	1,292	354
宮崎市 Miyazaki-shi	7,180	3,430	1,999	1,250	1,971	845	1,067	308	1,527	1,409	522	1,080	1,877	1,019	338
鹿児島市 Kagoshima-shi	7,001	2,073	1,354	724	1,325	728	685	301	1,942	1,717	620	773	1,965	900	289
那覇市 Naha-shi	10,119	1,961	1,477	2,642	151	159	538	151	2,445	831	884	316	582	611	275
政令指定都市（都道府県庁所在市を除く） (c)															
川崎市 Kawasaki-shi	10,053	3,081	2,191	3,294	617	552	652	424	2,619	764	1,236	346	1,440	1,051	466
相模原市 Sagamihara-shi	8,938	1,489	2,974	2,597	860	700	696	611	3,465	1,101	973	239	1,656	877	432
浜松市 Hamamatsu-shi	7,560	2,708	1,798	2,118	738	439	1,251	241	2,635	985	1,077	164	1,341	895	460
堺市 Sakai-shi	8,969	2,823	3,208	1,374	629	606	1,099	811	2,014	1,051	844	825	2,020	1,428	574
北九州市 Kitakyushu-shi	6,081	1,889	2,469	246	1,615	1,066	271	858	1,812	1,262	661	689	1,919	1,236	339

(a) Small cities B, towns & villages

(b) City with prefectural government

(c) Designated city under article 252-19 of the Local Autonomy Act excluding cities with prefectural governments

（注） この表には川崎市，相模原市，浜松市，堺市及び北九州市も掲載されている。

Note: Kawasaki-shi, Sagamihara-shi, Hamamatsu-shi, Sakai-shi and Kitakyushu-shi are also shown in this table.

21	22	23	24	25	26	27	28	29	30	31	32	33	34	35	36	37	38	39	40	41	42	43	44	45	46	47	48	49	50	51	52
え び	か に	あ さ り	し じ み	か き（貝）	ほ た て 貝	塩 さ け	た ら こ	しらす干し	干しあじ	かつお節・削	牛 肉	豚 肉	鶏 肉	合いびき肉	ハ ム	ソーセージ	ベーコン	牛 乳	粉ミルク	バ タ ー	チ ー ズ	卵	キャベツ	ほうれんそう	はくさい	ね ぎ	レ タ ス	ブロッコリー	も や し	さつまいも	じゃがいも
Shrimps & l	Crabs	Short-necke	Fresh water	Oysters	Scallops	Salted salm	Salted pollo	"Shirasu-boshi", dried young	Dried horse	Bonito fillets & fish flakes, d	Beef	Pork	Chicken	Mixed groun	Ham	Sausages	Bacon	Fresh milk	Powdered m	Butter	Cheese	Eggs	Cabbage	Spinach	Chinese cab	Welsh onion	Lettuce	Broccoli	Bean sprout	Sweet potat	White potat
1g	1g	1g	1g	1g	1g	1g	1g	1g	1g	1g	1g	1g	1g	1g	1g	1g	1g	1l	1g	1g	1g	1g	1g	1g	1g	1g	1g	1g	1g	1g	1g
数 量 Quan.	数 量 Quan.	数 量 Quan.	数 量 Quan.	数 量 Quan.	数 量 Quan.	数 量 Quan.	数 量 Quan.	数 量 Quan.	数 量 Quan.	数 量 Quan.	数 量 Quan.	数 量 Quan.	数 量 Quan.	数 量 Quan.	数 量 Quan.	数 量 Quan.	数 量 Quan.	数 量 Quan.	数 量 Quan.	数 量 Quan.	数 量 Quan.	数 量 Quan.	数 量 Quan.	数 量 Quan.	数 量 Quan.	数 量 Quan.	数 量 Quan.	数 量 Quan.	数 量 Quan.	数 量 Quan.	数 量 Quan.
1,347	358	705	286	467	393	1,278	665	398	575	227	6,717	21,518	16,865	2,038	2,592	5,301	1,546	76.24	287	503	3,488	31,933	17,020	3,054	7,787	4,415	6,282	4,085	6,743	2,618	9,470
1,808	416	554	360	208	1,036	1,933	917	187	143	157	4,958	25,065	18,210	1,100	2,311	5,825	2,203	68.70	667	732	4,098	30,225	15,736	2,884	9,099	4,433	4,825	3,832	7,106	2,956	13,439
1,219	1,909	562	772	151	4,100	2,707	1,648	227	300	116	6,528	23,463	14,759	970	2,829	6,546	1,874	62.48	165	357	3,204	36,643	16,985	3,591	6,241	5,221	6,012	4,116	9,234	2,065	8,432
991	193	546	403	379	906	2,719	654	236	511	140	4,068	23,174	16,821	1,162	2,690	5,987	1,595	79.71	361	424	4,051	33,935	19,425	4,154	6,856	6,603	4,724	5,035	9,652	2,988	8,852
1,002	154	762	461	517	416	2,099	659	369	277	152	5,708	23,066	13,191	1,038	2,735	5,799	2,139	86.77	116	543	3,839	32,279	14,760	3,516	11,849	5,001	4,885	4,196	9,431	4,330	8,386
1,679	266	553	744	312	655	2,560	1,581	261	393	148	4,755	24,263	16,832	859	2,672	6,152	1,974	71.38	160	362	2,962	36,045	20,342	4,535	6,620	6,509	5,684	5,006	10,423	2,663	8,331
985	212	594	259	500	518	2,090	1,036	225	325	226	7,743	19,051	14,618	926	2,103	5,233	2,044	73.50	393	362	3,622	33,193	15,953	3,957	5,096	5,670	4,697	5,026	8,713	2,191	7,850
782	160	812	462	385	362	2,519	803	456	380	151	3,678	22,098	11,749	635	3,111	4,682	1,609	68.22	246	364	3,203	31,796	14,758	3,628	5,635	3,953	5,215	4,106	7,489	2,634	7,452
779	145	552	809	317	308	2,009	865	645	537	262	4,551	21,131	13,809	1,233	3,149	5,575	1,426	77.09	609	445	3,230	30,697	17,968	3,291	7,071	5,169	6,150	4,576	5,783	2,626	8,238
1,152	454	838	295	246	259	1,243	715	705	612	184	5,013	20,221	13,280	1,067	2,706	4,412	1,238	79.21	593	514	3,490	30,010	17,646	2,967	5,993	5,070	5,771	4,892	6,945	2,626	7,921
1,107	287	958	207	365	278	1,173	864	458	703	207	3,355	18,048	10,708	952	3,466	4,652	1,320	62.23	262	380	3,542	26,963	16,327	2,900	8,500	4,083	9,126	4,745	6,831	2,650	8,580
1,060	184	768	314	634	496	1,449	797	444	804	184	5,983	26,137	16,873	2,065	2,947	5,036	1,656	84.37	274	480	4,220	30,330	18,910	3,494	8,328	5,584	7,681	5,100	7,442	2,559	10,629
1,394	438	800	443	677	707	1,331	735	428	875	266	6,789	22,651	16,793	1,930	2,379	5,162	1,561	95.77	412	590	4,125	29,979	18,388	4,324	9,092	6,005	9,418	5,527	7,309	2,650	11,273
1,055	205	809	287	483	473	1,286	687	508	784	180	7,345	22,469	16,196	1,874	2,791	4,629	1,623	77.46	270	674	4,206	26,432	18,252	3,355	7,922	5,600	8,344	5,595	7,025	2,260	10,134
1,045	154	844	341	569	434	1,132	641	576	975	232	6,195	22,343	15,279	1,705	2,981	4,681	1,729	84.35	252	703	4,347	27,172	17,150	3,485	6,808	4,884	7,077	4,988	6,577	2,695	10,318
966	1,450	1,129	463	318	509	3,521	1,259	291	418	165	4,271	27,027	15,973	1,078	2,653	5,253	1,783	94.02	291	327	3,528	32,881	16,858	4,036	7,255	6,295	7,693	5,132	9,947	2,728	10,631
1,992	909	675	409	340	416	1,913	643	385	480	143	5,348	21,150	14,460	2,847	3,227	6,223	1,620	82.08	49	408	4,086	38,311	18,701	4,385	7,831	4,818	6,598	4,605	8,698	2,154	9,712
1,608	657	534	248	453	260	1,236	644	331	333	150	7,551	21,753	14,527	2,678	3,150	6,619	1,640	78.37	118	536	3,634	31,883	15,798	2,864	7,904	3,938	5,671	4,221	8,561	1,847	8,644
1,042	1,072	515	161	482	250	1,322	537	512	464	172	7,442	17,851	13,815	2,461	3,048	5,355	1,242	65.69	166	509	2,829	29,797	13,068	3,648	5,934	3,706	3,727	2,947	7,086	2,455	10,186
1,127	370	921	492	379	499	1,568	501	716	970	177	4,878	21,490	12,425	1,118	2,971	5,123	1,558	65.92	591	439	2,850	24,068	17,288	3,257	6,862	3,829	7,565	4,384	5,528	1,835	8,749
1,005	621	903	381	412	406	1,765	1,088	495	842	173	3,119	20,863	13,675	1,145	2,313	5,364	1,536	91.14	551	447	3,881	31,841	16,448	3,386	7,482	3,144	7,143	4,742	7,395	2,757	7,462
1,328	383	921	431	441	298	878	591	430	588	197	6,882	19,179	13,569	2,136	3,347	4,540	1,424	71.92	298	522	3,283	32,644	13,630	2,941	9,171	4,947	6,450	4,093	6,436	2,399	9,735
1,254	469	1,189	232	427	391	1,488	525	1,191	1,255	410	5,246	24,005	17,102	1,616	3,023	5,192	1,986	70.29	523	726	3,898	29,311	19,372	3,228	8,559	5,251	6,622	3,829	6,539	2,402	12,083
1,416	438	667	237	412	275	831	534	422	468	180	5,553	21,165	16,815	2,777	2,643	6,081	1,337	78.61	557	561	3,999	32,911	18,013	2,768	8,104	5,331	7,247	4,638	6,933	2,428	10,279
1,588	203	872	203	626	330	1,981	421	537	499	233	8,254	17,937	16,496	2,296	2,363	6,283	1,424	79.67	934	533	3,022	34,394	17,065	2,822	7,607	4,260	5,392	3,689	5,498	3,333	9,013
1,736	366	596	140	513	240	1,688	839	592	572	184	9,523	20,652	18,777	2,976	3,300	5,009	1,670	77.30	344	693	3,605	34,894	20,395	4,173	11,384	4,274	7,692	5,128	7,551	3,190	11,130
1,617	1,250	732	198	394	411	939	794	586	540	297	8,714	17,825	18,336	2,110	3,071	4,314	1,283	76.60	354	610	3,869	31,521	17,839	3,884	8,218	4,523	6,786	4,949	6,320	3,092	11,777
1,789	597	519	205	708	355	1,586	723	425	369	272	10,125	20,975	18,250	2,899	3,012	5,347	1,342	82.73	229	423	3,302	34,956	17,651	3,051	11,949	4,467	6,468	4,576	5,823	3,121	10,537
1,620	672	291	94	619	234	925	446	272	189	245	7,939	19,536	17,612	2,467	2,765	4,917	1,238	87.50	201	662	3,656	30,355	15,989	2,951	9,603	3,548	5,010	3,781	5,900	1,745	8,213
1,505	221	633	216	561	311	1,437	480	581	381	279	10,288	18,142	17,191	2,429	2,660	5,355	1,365	83.22	216	618	3,725	33,088	17,727	3,546	9,832	4,306	6,458	5,105	6,080	2,712	11,640
1,978	417	536	176	598	185	1,089	457	1,115	575	323	9,691	19,906	17,848	2,097	2,327	4,336	1,252	75.02	235	500	2,386	33,851	16,574	2,347	9,001	3,661	5,266	3,637	5,483	2,704	11,168
1,835	2,299	740	424	761	230	1,335	438	236	568	146	7,687	20,349	21,097	3,772	2,211	6,342	1,443	82.09	258	344	2,983	41,932	16,748	3,386	8,639	4,091	5,609	4,258	8,668	1,867	8,883
1,250	1,298	685	1,702	258	187	869	335	247	583	149	6,387	21,457	19,389	2,877	2,367	5,375	1,433	85.40	291	519	3,233	32,255	18,785	3,502	7,776	4,350	5,926	4,389	7,007	3,018	10,770
1,375	65	416	149	590	144	1,218	426	315	183	191	6,719	19,698	16,776	2,517	1,676	4,435	1,324	75.75	189	403	2,939	30,123	16,514	2,628	8,976	3,380	5,052	3,944	6,324	2,840	8,019
1,482	264	677	223	1,624	236	666	553	176	177	157	9,330	21,216	18,284	2,228	2,524	5,518	1,382	83.77	206	570	2,898	33,826	18,466	2,928	8,849	4,234	5,299	4,198	6,396	2,227	9,735
1,817	184	628	123	504	288	906	744	158	607	115	8,519	20,683	18,584	2,541	2,420	6,398	1,888	77.89	437	447	3,183	36,400	16,928	2,548	10,342	3,106	5,459	3,591	7,713	2,312	9,442
1,236	131	651	128	271	151	1,148	208	237	194	290	7,660	16,124	14,738	2,517	1,722	4,683	1,081	75.65	401	467	2,771	29,888	15,398	2,304	7,345	2,745	4,442	2,661	5,539	3,693	9,269
1,608	473	601	126	470	214	984	337	209	262	248	8,876	20,329	18,972	3,545	3,673	5,006	1,465	85.83	215	334	2,942	36,323	17,666	2,980	9,051	3,745	4,819	2,837	6,319	2,967	10,522
1,533	543	528	151	335	540	949	359	456	450	191	9,388	19,831	17,402	2,692	2,283	5,348	1,395	62.34	326	375	2,653	34,009	15,749	2,516	8,295	2,965	5,837	2,774	5,587	2,707	8,633
1,615	270	845	161	434	276	1,198	312	1,040	837	310	7,859	19,929	18,831	3,372	1,863	5,565	1,181	54.21	365	436	2,651	36,290	13,312	3,251	9,528	3,024	5,312	3,202	5,872	2,517	8,350
1,260	368	726	40	347	284	563	1,453	205	275	209	7,515	20,814	21,561	2,866	2,538	4,473	1,604	63.56	416	555	3,628	31,333	18,583	2,588	7,421	3,467	5,836	4,152	6,089	2,439	9,247
1,609	1,131	980	134	343	225	602	552	140	493	199	9,412	19,818	19,531	3,222	2,676	5,233	1,709	70.30	243	519	3,272	32,783	18,886	3,057	9,530	3,321	6,342	3,470	7,869	3,301	10,652
1,412	394	1,050	114	906	124	602	469	114	566	226	6,674	16,047	16,304	2,988	2,453	5,021	1,742	68.83	344	457	3,411	30,181	16,974	2,783	8,576	2,735	5,749	4,854	5,114	2,913	11,714
1,234	256	912	84	216	229	816	568	168	169	149	7,951	18,677	20,250	2,683	1,937	5,319	1,512	61.78	385	401	2,887	27,916	13,806	1,861	5,276	2,699	4,850	2,787	6,449	2,141	8,517
1,320	438	1,115	152	163	328	915	749	134	696	164	8,858	18,952	20,455	2,979	2,466	4,951	1,453	72.90	245	442	2,493	33,180	19,362	2,451	9,357	3,169	5,717	3,965	6,067	2,709	11,446
1,249	205	816	150	139	309	479	498	63	962	176	7,794	19,359	19,245	2,667	1,831	4,717	1,374	71.12	209	430	2,711	30,599	17,291	2,363	8,580	2,765	4,963	3,218	5,502	2,431	9,515
1,344	144	1,019	42	116	171	355	493	133	469	379	6,748	20,779	19,973	2,830	1,976	5,134	1,161	59.16	826	383	2,704	31,034	20,957	1,994	8,426	3,589	6,579	3,603	5,923	3,254	9,416
533	115	501	161	136	246	284	147	172	30	912	6,965	18,710	12,603	2,045	1,963	4,696	2,332	47.28	886	255	3,756	31,920	12,467	2,779	4,545	2,072	5,787	3,671	4,821	1,671	7,189
1,067	162	876	431	424	383	1,178	897	641	643	154	7,346	25,479	18,563	2,432	3,123	5,206	1,750	90.03	649	700	4,841	30,923	19,598	3,872	8,626	5,769	8,230	5,956	7,281	3,266	11,189
1,343	181	910	296	319	384	1,255	950	620	1,094	238	6,386	26,513	17,766	2,046	3,497	6,410	1,656	87.58	675	513	4,392	31,256	19,508	3,994	8,200	5,720	9,092	5,806	7,502	2,902	11,013
1,270	181	1,041	59	436	243	1,010	521	776	710	254	4,247	22,583	13,759	1,838	2,701	5,059	1,512	76.66	159	488	3,799	31,391	16,545	2,814	8,580	4,452	6,162	3,931	5,992	2,614	8,746
1,745	503	468	158	362	417	1,211	561	423	271	408	9,323	20,904	19,389	2,734	2,623	5,745	1,396	70.69	276	478	2,955	35,188	15,339	3,463	11,449	3,629	5,898	3,387	5,977	2,486	11,566
1,971	532	790	102	350	265	777	1,067	250	626	137	8,391	17,199	19,027	2,040	2,566	5,580	1,382	60.72	186	279	2,705	30,842	19,320	2,719	10,806	3,845	5,649	3,168	5,919	2,768	9,237

Note: Kawasaki-shi, Sagamihara-shi, Hamamatsu-shi, Sakai-shi and Kitakyushu-shi are Note: Kawasaki-shi, Sagamihara-shi, Hamamatsu-shi, Sakai-sh Note: Kawasaki-shi, Sagamihara-shi, Hamamatsu-shi, Sakai-shi and Kitakyu

53 さといも Taros 1g	54 だいこん Radishes 1g	55 にんじん Carrots 1g	56 ご ぼ う Burdocks 1g	57 たまねぎ Onions 1g	58 れんこん Lotus roots 1g	59 たけのこ Bamboo sho 1g	60 さやまめ String beans 1g	61 かぼちゃ Pumpkins 1g	62 きゅうり Cucumbers 1g	63 な す Eggplants 1g	64 ト マ ト Tomatoes 1g	65 ピーマン Green peppe 1g	66 生しいたけ "Shiitake", Japanese m fresh 1g	67 干ししいたけ "Shiitake", Japanese mushrooms, dried 1g	68 わ か め "Wakame", 1g
数 量 Quan.	数 量 Quan.	数 量 Quan.	数 量 Quan.	数 量 Quan.	数 量 Quan.	数 量 Quan.	数 量 Quan.	数 量 Quan.	数 量 Quan.	数 量 Quan.	数 量 Quan.	数 量 Quan.	数 量 Quan.	数 量 Quan.	数 量 Quan.
1,502	11,698	8,037	1,556	15,997	1,350	753	1,785	3,840	7,715	4,140	11,888	2,925	1,586	47	865
600	16,140	7,483	1,599	21,041	764	768	1,449	3,947	6,694	4,023	9,945	2,721	1,819	58	447
461	11,889	8,393	2,289	17,964	758	725	1,932	3,254	6,748	5,130	13,008	2,771	1,833	47	1,731
1,447	16,392	9,998	2,484	18,069	1,019	625	2,527	4,850	8,345	4,470	13,452	2,970	2,029	56	1,868
1,718	14,548	8,497	2,098	15,585	1,523	1,425	2,062	5,059	9,034	5,204	12,443	3,168	1,544	44	1,602
722	14,779	8,454	1,939	17,717	821	635	4,051	4,998	9,127	6,469	14,174	3,184	2,198	35	1,632
2,240	13,706	7,851	2,007	14,180	915	1,279	1,800	3,906	8,684	4,692	8,796	2,365	1,512	41	970
1,583	11,558	7,963	1,607	13,248	838	954	1,675	3,293	8,656	4,247	11,182	1,934	1,722	15	1,020
1,309	13,224	8,225	1,456	16,359	1,343	217	1,174	3,874	8,544	3,723	12,658	3,176	1,582	44	1,360
1,930	13,213	9,509	1,761	14,091	1,015	1,242	1,799	3,724	8,256	4,515	12,731	2,668	1,794	18	1,025
1,889	10,807	7,805	1,510	12,626	823	764	1,750	3,755	10,466	2,827	16,245	2,626	1,863	57	1,201
2,405	13,847	9,012	1,665	18,045	1,333	1,240	2,540	3,868	9,929	5,192	15,685	3,445	1,495	39	907
1,498	14,041	9,498	1,452	17,065	1,455	765	2,581	4,490	8,331	4,683	16,483	3,723	1,704	29	1,141
1,574	13,619	8,324	1,371	16,264	1,400	709	2,763	4,106	8,845	4,995	14,686	3,517	1,467	32	884
1,696	12,912	8,976	1,482	16,673	1,451	786	2,527	4,605	8,375	5,273	13,442	3,478	1,349	74	1,044
2,630	14,256	9,540	1,536	16,605	2,626	1,182	4,053	4,819	8,706	4,930	15,133	3,574	2,213	52	1,198
1,926	11,671	7,537	1,525	14,729	1,383	859	1,370	4,758	8,522	4,633	12,272	2,969	1,617	56	840
993	10,086	7,927	1,087	14,035	1,833	858	1,345	3,331	7,211	4,274	11,044	2,720	1,851	47	823
2,141	10,604	7,272	1,215	13,917	892	1,018	1,190	3,240	7,309	4,281	11,807	2,169	2,162	39	1,099
1,645	12,250	7,823	1,507	13,755	1,418	827	1,301	4,207	8,478	3,594	11,011	2,506	1,486	45	1,036
1,189	13,468	7,955	1,530	12,744	1,820	626	1,647	4,003	9,910	3,232	9,876	2,728	1,357	87	1,324
2,419	10,506	6,891	1,583	14,376	1,550	887	2,155	4,808	7,125	4,447	10,963	2,680	1,482	70	665
2,040	13,400	8,922	1,369	16,329	1,878	883	2,082	3,754	7,513	4,096	12,297	2,777	1,705	39	1,306
1,889	12,081	8,591	1,337	17,413	1,800	821	2,171	4,545	7,054	4,473	11,489	3,020	1,613	57	675
1,398	12,041	7,957	1,547	15,390	1,345	767	1,300	4,775	6,596	3,861	14,386	2,642	1,698	67	1,009
1,845	14,342	9,325	1,495	18,909	1,331	1,153	1,936	5,584	8,132	5,284	15,603	3,162	1,896	32	706
1,381	14,109	8,005	1,398	18,043	1,421	1,588	1,945	4,533	8,801	5,895	13,930	3,582	1,666	100	835
1,443	12,440	7,709	1,272	18,927	1,324	619	1,869	4,346	6,740	4,144	11,930	3,572	1,595	29	712
985	10,032	7,093	1,206	13,773	1,318	785	1,912	3,409	5,744	3,789	14,116	3,455	1,929	35	462
1,772	14,068	8,322	1,502	16,920	1,704	934	1,957	4,137	8,265	6,052	13,158	3,509	2,211	36	612
1,258	11,159	6,618	1,378	16,173	1,001	548	1,546	3,982	5,960	3,420	9,345	2,539	1,598	57	638
1,438	9,788	8,672	1,209	15,245	546	656	1,515	3,452	6,361	3,328	8,951	2,347	1,962	33	694
1,607	11,063	9,186	1,486	17,679	521	517	1,581	4,251	7,106	3,770	11,800	2,868	1,999	33	881
832	10,127	8,126	1,375	16,294	1,429	423	1,303	3,424	5,941	2,927	10,052	2,459	1,387	17	385
1,428	9,831	8,380	1,634	15,955	2,036	573	1,629	3,913	7,582	4,204	8,851	2,808	1,643	24	458
1,589	10,275	7,454	1,941	15,358	2,071	442	1,482	4,165	6,157	4,256	9,473	2,183	1,512	45	637
1,018	8,791	6,817	978	15,682	952	568	1,750	3,147	5,778	2,860	10,506	2,152	1,903	61	1,123
1,362	10,397	7,638	1,632	15,807	1,969	732	1,248	4,520	6,116	3,783	8,065	2,958	1,572	23	906
2,152	9,351	7,082	1,476	14,660	1,897	420	1,786	4,581	6,825	3,651	11,597	2,215	1,304	50	560
737	10,857	7,585	1,372	15,155	732	864	1,394	3,360	7,588	3,719	10,552	2,633	1,174	32	778
1,980	11,871	7,548	1,792	16,506	1,824	939	1,648	3,569	7,739	4,752	12,292	3,595	1,419	57	741
1,753	11,411	8,682	2,249	18,882	3,106	963	1,227	4,888	10,275	4,795	13,255	2,931	1,579	53	791
1,957	9,992	7,773	1,571	16,241	1,777	714	1,571	5,067	9,308	4,700	14,229	3,067	1,599	94	897
1,781	8,999	6,504	1,431	14,938	1,212	473	1,094	3,147	9,003	3,696	10,966	2,502	1,220	61	697
2,344	10,104	8,005	2,084	17,816	1,453	700	1,377	3,547	7,834	4,291	11,507	2,974	1,927	124	746
2,156	10,717	6,545	1,679	15,164	610	420	823	3,325	10,980	3,283	11,483	2,587	1,456	54	578
2,042	11,740	8,804	1,651	15,211	536	720	1,555	3,759	8,891	2,833	10,135	2,787	1,442	64	600
514	9,639	10,510	1,343	15,055	262	129	1,377	2,743	4,828	2,354	9,140	3,655	675	58	302
1,720	15,170	9,632	1,287	17,507	1,494	515	2,538	4,695	9,742	4,491	13,432	3,507	1,322	71	832
1,685	14,207	9,693	1,737	19,087	1,592	678	2,085	4,328	8,820	4,988	14,106	3,472	1,452	32	1,007
1,505	10,333	8,638	1,365	13,658	1,569	662	1,214	4,013	5,255	3,490	11,754	2,638	1,559	22	994
1,551	10,869	7,441	1,456	18,890	1,729	1,157	2,196	4,375	6,817	4,365	11,294	2,948	1,653	55	672
1,924	11,753	7,399	1,860	18,205	1,939	547	1,323	3,674	8,162	4,766	12,265	3,145	1,504	68	850

69 こ ん ぶ Dried tangle 1g	70 豆 腐 Bean curd 1丁 each	71 梅 干 し "Umeboshi", pickled plum 1g	72 だいこん漬 Pickled radis 1g	73 はくさい漬 Pickled chinese cab 1g	74 こんぶつくだ Tangle prepared in soy sauce 1g	75 り ん ご Apples 1g	76 み か ん Mandarin or 1g	77 グレープフル Grapefruits 1g	78 オレンジ Oranges 1g	79 梨 Pears 1g	80 ぶ ど う Grapes 1g	81 柿 Persimmons 1g	82 桃 Peaches 1g	83 す い か Watermelons 1g	84 メ ロ ン Melons 1g
数 量 Quan.	数 量 Quan.	数 量 Quan.	数 量 Quan.	数 量 Quan.	数 量 Quan.	数 量 Quan.	数 量 Quan.	数 量 Quan.	数 量 Quan.	数 量 Quan.	数 量 Quan.	数 量 Quan.	数 量 Quan.	数 量 Quan.	数 量 Quan.
277	83.66	710	1,300	784	558	10,363	9,476	747	1,332	3,354	2,272	2,440	1,299	3,555	1,801
261	55.71	766	1,074	504	515	9,402	9,771	759	1,670	1,439	1,472	2,546	1,146	2,890	2,331
576	84.53	1,028	1,967	637	591	25,845	7,283	1,103	1,793	1,563	1,550	3,939	1,134	1,575	3,063
576	93.49	876	1,588	651	625	32,513	10,523	979	1,270	4,230	3,701	2,603	1,231	2,239	1,975
395	82.70	584	1,387	1,001	523	11,386	8,507	1,255	1,144	6,231	1,836	2,805	1,436	5,699	1,459
403	77.82	1,075	2,370	559	486	19,117	8,414	872	1,041	3,264	2,355	1,362	1,218	3,584	3,543
445	89.55	626	1,429	990	650	12,910	10,066	967	958	3,836	3,564	2,250	2,082	3,359	1,501
327	88.64	955	1,479	1,121	710	16,209	7,360	343	882	6,384	3,446	1,795	11,772	2,957	790
350	80.35	676	1,150	709	928	15,854	10,275	926	1,884	5,098	1,869	1,777	1,176	2,694	6,786
277	93.56	597	1,236	1,092	660	11,240	9,983	1,057	1,100	5,310	2,090	1,652	1,068	3,810	1,594
404	94.67	679	1,693	846	739	15,016	11,149	1,221	1,334	3,957	2,459	1,271	1,245	2,727	2,400
296	79.34	1,170	1,204	846	640	10,937	8,150	974	1,702	2,940	2,373	1,206	1,338	4,585	1,807
197	98.97	857	1,342	1,345	650	9,818	8,378	1,105	2,328	5,483	2,863	2,467	1,149	4,410	1,660
245	80.80	822	1,082	1,007	467	9,437	9,131	1,097	1,301	3,286	2,464	2,510	1,460	3,847	2,034
317	78.32	646	1,021	834	522	11,596	10,964	784	2,520	4,758	2,071	2,829	1,309	4,939	2,088
395	110.87	775	1,293	728	861	11,720	11,079	1,476	938	8,614	2,183	2,911	2,216	4,674	1,759
424	100.24	909	2,100	1,251	964	11,393	10,058	829	2,190	6,469	1,464	2,067	1,384	4,028	1,851
476	88.06	619	1,389	759	697	8,417	5,788	351	1,794	3,507	1,848	1,634	791	4,881	979
318	70.23	566	1,916	544	1,114	9,571	10,261	672	677	3,348	2,241	1,381	613	2,360	1,217
159	86.25	509	1,297	837	911	8,105	8,646	968	1,319	1,409	4,908	1,494	3,365	1,722	1,073
490	76.75	762	1,113	488	656	21,429	9,211	542	1,574	1,513	2,391	898	3,811	2,738	1,164
257	103.25	601	995	652	804	10,602	5,986	1,036	1,463	1,860	2,557	10,194	1,081	3,478	1,185
355	97.89	495	1,503	1,262	451	6,787	14,465	505	1,275	2,146	1,699	1,607	800	2,565	895
117	93.54	621	1,294	709	455	8,017	6,301	577	1,399	2,565	2,031	3,615	1,048	2,056	1,309
211	81.75	785	1,823	402	571	8,456	12,794	399	888	5,221	2,411	3,039	629	2,573	1,983
442	86.57	581	1,204	933	1,183	11,508	12,895	476	1,709	2,683	1,957	2,713	1,256	2,848	1,147
310	89.63	628	1,475	778	730	10,244	8,678	1,393	1,109	2,455	1,762	4,248	933	4,042	1,473
356	78.08	690	1,535	1,179	1,095	8,781	7,309	987	1,859	1,511	1,664	2,352	960	3,777	878
202	75.79	572	1,201	617	441	9,149	6,075	463	1,316	2,272	2,066	2,863	397	3,717	545
271	90.09	758	1,797	773	772	12,127	11,822	1,252	1,691	3,551	2,391	5,781	1,879	5,109	889
253	70.94	679	1,190	410	786	11,255	11,006	957	947	2,389	2,279	3,619	5,250	5,669	1,291
137	83.85	616	1,425	2,063	557	8,619	8,961	300	1,958	11,812	2,409	3,048	705	4,633	1,127
380	84.11	826	1,444	983	447	8,973	6,731	1,209	1,431	5,608	1,517	2,721	618	2,313	1,712
157	87.52	537	765	1,060	407	7,459	8,430	512	1,185	2,055	2,919	1,866	9,252	2,598	1,124
186	97.09	684	1,133	1,122	371	12,012	11,740	342	1,351	2,328	3,162	3,077	661	5,761	1,998
222	89.66	779	1,421	1,233	459	9,963	9,815	516	1,292	3,339	1,901	1,216	1,731	2,943	1,125
177	87.71	612	1,226	588	369	11,850	10,330	451	825	1,689	1,462	3,174	1,471	3,631	1,644
282	93.50	920	1,293	861	476	11,839	13,980	395	1,509	2,310	1,839	1,855	2,310	2,982	580
216	82.47	410	860	353	253	7,323	9,576	95	276	2,381	2,002	3,880	1,243	4,027	496
326	71.57	608	1,215	2,507	589	10,705	8,900	145	1,050	3,005	1,774	1,278	541	2,156	1,136
168	76.24	481	971	544	435	9,056	11,777	519	920	3,485	2,290	2,680	858	2,492	1,159
378	88.41	730	1,571	613	521	8,585	9,981	320	777	3,173	2,189	1,688	466	4,628	1,950
313	89.29	647	1,684	775	500	11,045	15,135	674	788	3,795	2,544	4,532	920	6,333	2,435
201	69.44	943	1,245	630	537	6,134	10,699	178	476	2,487	2,432	2,215	284	5,847	1,003
312	77.01	746	1,844	846	605	9,889	12,627	286	660	4,565	2,240	2,530	721	4,214	1,446
176	82.24	471	1,245	783	291	9,327	12,859	293	519	2,179	1,668	2,979	695	2,187	3,066
178	74.92	358	856	423	444	9,192	10,334	233	540	2,506	1,419	2,133	232	3,433	713
353	63.31	897	587	95	153	5,668	8,454	533	1,266	1,277	1,057	1,407	205	2,222	359
250	73.28	627	811	800	416	8,317	8,213	1,007	1,505	2,126	2,069	1,533	1,305	2,163	1,575
244	102.25	661	1,792	657	507	10,799	9,477	1,145	1,579	3,971	2,446	2,684	787	3,265	1,019
236	105.82	690	1,331	771	459	9,552	14,444	299	1,307	5,882	2,526	3,485	2,026	3,019	1,652
257	85.70	542	1,313	636	415	8,015	9,133	443	694	1,578	1,989	3,102	1,181	3,443	886
222	92.39	643	1,137	1,125	386	10,437	9,850	571	1,687	3,162	2,388	3,164	557	3,372	894

ushu-shi are also shown in this table.

この表には川崎市，相模原市，浜松Note: Kawasaki-shi, Sagamihara-shi, Hamamatsu-shi, Sakai-shi and Kitakyushu-shi are also shown in this table.

85 いちご Strawberries 1g	86 バナナ Bananas 1g	87 キウイフルーツ Kiwi fruits 1g	88 食用油 Edible oil 1g	89 マーガリン Margarine 1g	90 食塩 Salt 1g	91 しょう油 Soy sauce 1ml	92 みそ "Miso", soy 1g	93 砂糖 Sugar 1g	94 酢 Vinegar 1ml	95 ソース Worcester s 1ml	96 ケチャップ Tomato ketc 1g	97 マヨネーズ マヨネーズ風調味料 Mayonnaise & mayonnaise flavor seasoning 1g	98 ドレッシング Dressing 1ml	99 ジャム Jam 1g	100 カレールウ Instant curr 1g	101 緑茶 Green tea 1g	102 紅茶 Black tea 1g	103 コーヒー Coffee 1g	104 清酒 "Sake" 1ml	105 焼酎 "Shochu", distilled spir 1ml	106 ビール Beer 1l	107 ウイスキー Whisky 1ml	108 ワイン Wine 1ml	109 発泡酒・ビール風アルコール飲料 Low-malt beer & beer-flavored alcoholic beverages 1l
数量 Quan.	数量 Quan.	数量 Quan.	数量 Quan.	数量 Quan.	数量 Quan.	数量 Quan.	数量 Quan.	数量 Quan.	数量 Quan.	数量 Quan.	数量 Quan.	数量 Quan.	数量 Quan.	数量 Quan.	数量 Quan.	数量 Quan.	数量 Quan.	数量 Quan.	数量 Quan.	数量 Quan.	数量 Quan.	数量 Quan.	数量 Quan.	数量 Quan.
2,242	18,448	2,339	8,368	917	1,769	4,981	5,194	4,736	2,180	1,458	1,531	2,520	2,391	1,109	1,441	798	173	2,478	6,640	9,245	19.37	1,126	3,327	26.43
902	17,563	2,350	8,890	680	904	5,172	5,285	3,004	2,082	1,130	1,739	2,581	1,874	879	1,541	778	127	3,208	9,490	7,868	27.35	2,489	7,085	40.92
2,224	17,252	2,192	10,006	1,000	3,661	5,770	6,553	4,584	2,848	1,621	1,428	2,776	2,223	1,372	1,558	369	97	2,691	9,988	12,507	25.18	3,214	5,351	33.36
2,771	18,816	3,101	8,308	958	1,916	6,182	7,316	3,690	2,531	1,067	1,556	2,626	2,276	1,122	1,715	829	171	2,480	7,165	10,076	25.62	980	3,909	32.31
2,533	17,815	2,594	7,960	768	1,808	4,808	5,085	5,290	1,889	1,333	1,382	2,039	2,106	966	1,502	994	272	2,429	9,028	7,473	22.74	1,470	5,353	21.12
2,119	17,868	2,488	8,089	834	1,608	5,957	7,875	4,750	2,146	977	1,271	2,334	1,835	1,040	1,429	1,038	168	2,323	12,203	11,329	23.72	2,061	6,359	35.97
1,921	18,180	1,991	8,609	533	2,715	6,270	7,142	4,899	2,006	1,130	1,175	2,651	2,138	955	1,428	518	152	2,523	6,120	7,303	20.43	3,442	3,062	27.34
2,889	17,961	2,317	8,287	637	762	3,565	6,583	4,403	3,205	1,094	1,187	2,322	2,263	1,347	1,203	739	130	2,057	10,419	6,663	15.74	2,262	2,932	24.82
2,878	17,749	1,965	8,026	669	1,612	6,189	5,295	4,504	1,428	984	1,560	2,828	2,326	959	1,420	791	129	2,782	5,256	2,907	24.75	1,548	2,300	19.66
2,927	18,973	2,451	7,369	604	1,911	4,673	3,782	3,737	2,347	1,215	1,483	1,947	2,348	777	1,329	745	194	2,534	7,414	8,738	19.94	1,484	3,262	31.61
2,921	18,482	2,578	8,472	726	1,883	4,853	4,380	6,984	2,742	1,332	1,333	2,146	2,944	1,012	1,197	649	257	2,040	10,798	9,695	18.96	1,130	5,647	16.51
2,549	17,843	3,027	9,636	985	1,589	5,617	5,554	3,261	2,478	1,346	1,578	2,377	2,697	1,359	1,582	852	463	2,444	6,051	7,557	18.35	912	4,086	27.50
3,119	18,261	3,206	8,622	907	1,295	4,433	4,616	2,887	2,189	1,317	1,555	2,299	2,806	1,300	1,437	1,208	229	2,475	8,642	11,175	26.50	1,983	3,921	24.37
3,010	15,836	2,956	6,940	894	1,241	3,762	4,140	3,530	2,133	1,244	1,422	2,215	2,568	1,322	1,251	825	318	2,714	6,041	6,990	19.34	1,341	4,381	19.63
2,644	20,432	3,393	6,868	921	1,553	4,305	4,279	3,956	1,784	1,231	1,069	2,137	2,737	1,484	1,197	854	265	2,914	5,147	8,370	20.96	1,270	5,351	16.94
1,990	18,236	3,055	7,965	896	2,136	5,771	6,469	4,331	2,554	1,173	1,616	2,764	2,173	1,044	1,896	819	113	2,737	12,140	11,118	26.29	1,988	5,443	40.16
2,240	18,870	4,229	8,211	1,188	1,565	6,412	7,279	3,857	1,969	1,377	1,709	2,678	2,403	1,068	1,472	845	222	3,011	9,539	9,760	19.99	823	3,109	44.92
1,297	17,678	2,139	6,160	1,032	2,029	5,016	4,414	3,766	1,529	1,165	1,603	2,628	2,784	1,168	1,627	1,039	78	3,308	6,471	7,310	20.45	1,408	2,024	28.94
2,086	18,775	1,924	7,025	822	1,327	3,590	5,212	3,424	1,587	1,451	1,401	2,173	1,956	725	1,413	774	145	1,907	6,774	5,361	19.78	849	1,866	14.19
2,290	17,585	1,734	6,919	798	2,218	4,893	4,176	3,577	1,782	928	1,525	2,126	2,884	1,120	1,391	663	254	2,276	5,466	9,037	15.00	1,513	2,802	19.56
2,054	17,359	2,138	8,638	868	3,508	4,869	9,172	6,760	2,114	1,136	1,497	2,491	2,725	1,492	1,392	636	153	2,330	7,440	10,090	23.47	1,293	2,780	27.72
2,791	20,340	2,331	8,292	846	1,078	5,366	6,222	4,514	2,023	1,444	1,792	2,853	2,087	1,163	1,340	753	168	2,288	3,598	7,532	18.13	1,921	2,951	26.99
2,884	17,278	1,859	7,945	747	1,494	6,395	5,199	4,237	1,491	1,431	1,659	2,604	2,283	989	1,246	2,333	200	2,154	8,057	7,352	23.87	855	4,566	20.32
2,617	22,159	2,761	7,179	1,174	1,284	4,857	5,305	3,462	2,896	1,654	1,872	2,581	2,247	1,107	1,590	901	171	2,335	5,530	6,847	15.87	1,142	1,627	21.20
3,224	21,609	3,323	7,719	700	1,134	5,355	7,149	4,070	3,152	1,384	1,661	2,524	1,820	1,242	1,237	1,407	122	2,348	6,748	9,659	20.47	1,261	1,952	19.50
2,081	22,972	2,664	7,997	1,138	1,435	5,814	4,036	6,004	1,742	2,065	1,670	2,218	2,773	1,073	1,355	916	145	2,784	6,558	8,281	25.05	1,308	3,646	31.69
1,553	19,795	2,483	7,776	972	1,105	5,437	4,011	4,596	1,188	2,506	1,606	2,409	2,372	1,154	1,278	912	248	3,835	6,304	10,087	20.79	1,309	3,973	28.83
2,142	19,095	1,907	9,929	1,117	1,431	4,557	4,167	4,166	1,706	1,799	1,797	2,590	2,307	1,054	1,465	738	163	2,871	7,300	8,448	15.22	934	1,937	34.62
1,410	18,209	2,987	6,645	1,106	1,383	3,538	3,930	4,723	1,907	2,143	1,687	1,859	2,293	1,001	1,045	629	367	2,671	5,468	4,946	18.43	1,279	3,276	30.75
2,724	22,422	3,211	8,463	1,007	1,338	5,400	4,545	5,505	2,140	1,922	1,545	2,527	2,577	1,344	1,486	905	225	3,040	6,271	6,095	18.50	669	3,144	25.06
2,119	21,839	1,860	8,392	969	1,620	4,802	8,038	6,050	3,395	1,659	1,604	2,656	2,276	924	1,292	819	274	2,435	9,126	8,490	22.62	1,240	2,369	21.45
2,225	22,218	2,224	9,750	1,293	1,617	6,360	5,500	6,031	1,136	1,666	1,685	3,250	2,315	916	1,910	627	122	3,037	4,724	5,275	18.33	1,274	1,925	28.45
2,416	16,838	2,244	8,325	719	1,555	4,879	4,576	5,745	2,233	1,335	1,500	2,411	3,241	1,185	1,561	792	154	2,416	6,200	9,863	18.98	2,377	2,854	23.97
2,099	17,484	1,848	6,339	911	2,654	5,620	3,846	3,664	2,382	1,930	1,615	2,312	2,430	968	1,155	623	97	2,969	8,620	8,788	13.18	712	1,911	22.06
2,430	23,527	1,492	8,682	1,193	1,727	5,263	4,687	4,284	1,725	2,371	1,403	2,478	2,101	976	1,270	720	100	3,041	7,111	12,702	19.67	1,542	2,284	28.92
2,938	17,586	1,305	9,573	1,055	1,810	4,347	5,422	4,065	1,521	1,526	1,195	2,508	2,147	1,012	1,614	656	79	2,853	3,931	16,020	25.69	842	1,190	39.87
2,315	14,453	1,837	9,136	760	1,302	4,253	4,661	4,819	1,581	1,789	1,597	2,716	1,966	924	1,420	782	183	2,515	6,866	7,294	25.23	904	1,489	27.71
1,979	20,319	1,886	12,641	963	1,418	5,266	4,138	5,584	1,887	1,959	1,786	2,334	2,369	956	1,601	570	215	3,097	5,976	8,307	12.57	669	1,453	33.50
1,815	16,510	1,805	10,866	761	1,820	3,791	4,934	5,822	2,278	1,874	1,499	2,626	2,119	867	1,375	737	54	2,328	6,450	10,391	24.19	472	983	25.59
2,559	15,342	1,742	8,484	711	1,686	5,023	4,872	4,732	2,128	1,592	1,502	2,291	2,192	839	1,290	662	131	2,368	4,245	9,920	15.30	1,365	2,947	48.74
2,197	16,061	2,380	7,380	856	1,601	4,188	4,439	3,537	1,965	1,215	1,955	2,848	2,425	849	1,245	828	219	2,463	4,627	6,239	17.17	656	7,553	24.96
2,651	17,030	1,698	9,850	784	1,454	5,455	6,086	4,779	2,265	1,085	2,063	2,919	2,564	886	1,428	905	150	2,757	5,574	9,278	16.69	721	2,429	30.66
2,899	20,430	2,832	8,767	998	1,437	5,862	6,005	7,000	3,052	1,123	1,371	2,619	2,461	921	1,187	1,180	114	2,679	5,092	9,486	17.41	982	3,072	30.39
1,840	14,759	1,840	9,011	883	1,882	4,147	6,316	4,373	1,713	1,273	1,899	2,700	2,583	781	1,379	848	85	1,770	3,864	13,880	22.07	814	1,740	33.59
2,424	20,513	2,247	10,114	751	2,124	5,510	5,098	5,648	3,552	889	1,788	2,481	2,239	1,032	1,158	810	174	2,500	4,311	15,650	17.80	570	2,740	25.51
1,727	15,917	1,952	8,555	598	1,736	5,060	6,524	5,507	2,564	1,274	1,504	2,820	2,851	719	1,439	713	160	1,554	2,754	21,393	15.63	306	2,626	40.78
2,379	17,334	1,937	11,771	640	1,607	6,635	7,700	5,925	3,798	1,181	1,681	2,950	2,334	633	1,669	757	143	2,087	1,216	12,901	17.82	209	1,135	24.79
1,276	14,500	1,285	10,351	560	857	3,031	3,877	3,868	1,156	637	1,611	1,116	2,261	772	1,207	224	249	2,188	1,805	8,857	16.04	400	3,117	29.53
2,547	18,999	2,550	7,615	741	1,390	3,951	5,525	3,644	3,215	1,134	1,582	2,268	2,332	1,076	1,546	654	235	2,572	6,317	6,729	16.02	916	4,467	27.43
2,572	19,750	3,667	8,159	1,022	1,669	3,999	4,913	4,787	2,502	1,507	1,738	2,845	2,508	1,357	1,744	995	165	2,793	5,914	8,733	14.45	1,158	6,031	30.26
2,557	18,551	2,645	8,738	702	1,707	5,004	5,913	4,440	1,727	1,144	1,202	2,195	2,083	1,548	1,118	1,704	103	1,896	5,244	4,691	13.96	1,225	2,292	15.14
2,259	20,225	1,904	7,108	1,078	1,436	4,006	3,957	4,662	1,891	1,843	1,716	2,567	1,978	1,791	1,393	1,313	246	2,747	6,761	6,955	20.18	688	1,865	25.14
2,431	19,805	1,907	8,398	1,065	972	5,674	5,042	5,347	1,609	1,720	1,426	2,733	2,555	915	1,212	884	159	2,196	4,734	16,358	22.08	1,730	1,353	27.25

この表には川崎市，相模原市，浜松市，堺市及び北九州市も掲載されている。

Note: Kawasaki-shi, Sagamihara-shi, Hamamatsu-shi, Sakai-shi and Kitakyushu-shi are also shown in this table.

Nutrient calculations

		朝昼夕間食	献立名	食品番号	食品名	Weight 重量 (g)	食品群	廃棄率 (%)	Erergy エネルギー (kcal)	Erergy エネルギー (kJ)	Water 水分 (g)	Protein たんぱく質 (g)	アミノ酸組成たんぱく質 (g)	Fat 脂質 (g)	TG当量 (g)	Fatty acids 飽和脂肪酸 (g)	Fatty acids 一価不飽和脂肪酸 (g)	Fatty acids 多価不飽和脂肪酸 (g)	Cholesterol コレステロール (mg)	Carbohydrate 炭水化物 (g)	利用可能炭水化物 (g)	低分子量水溶性食物繊維 (g)
全国	All Japan	総 合 計	0.0	0.0	0.0	674416.0	0.0	0.0	1027991.7	4300286.5	469216.5	33757.5	28463.3	41468.0	38614.3	12243.3	16545.8	8279.5	194285.0	115937.2	108571.5	564.6
札幌市	Sapporo-shi	総 合 計	0.0	0.0	0.0	718521.5	0.0	0.0	1116193.9	4669587.9	496009.6	35276.0	29665.7	42512.4	39759.8	12696.6	16960.1	8549.9	194244.3	129805.6	122969.1	553.4
青森市	Aomori-shi	総 合 計	0.0	0.0	0.0	737718.5	0.0	0.0	1135340.9	4749758.6	509021.3	37090.5	31273.7	44481.5	41379.2	12563.3	17802.0	9402.4	221823.5	128085.6	119304.7	532.2
盛岡市	Morioka-shi	総 合 計	0.0	0.0	0.0	769366.5	0.0	0.0	1139388.2	4766704.1	536631.7	37160.7	31350.8	42562.0	39569.5	12548.0	16672.2	8806.2	202731.1	136873.5	126571.4	569.2
仙台市	Sendai-shi	総 合 計	0.0	0.0	0.0	694245.0	0.0	0.0	1008011.7	4216887.7	492008.1	33650.4	28346.1	40686.4	37873.6	12373.5	15993.3	8023.1	194085.1	113359.0	104547.4	523.4
秋田市	Akita-shi	総 合 計	0.0	0.0	0.0	720690.0	0.0	0.0	1022686.8	4278578.4	516768.4	35108.8	29458.1	41275.5	38359.3	12185.3	16331.0	8344.9	215491.0	110649.2	99496.2	490.3
山形市	Yamagata-shi	総 合 計	0.0	0.0	0.0	675686.5	0.0	0.0	1027403.7	4298490.9	468456.8	33258.8	28149.1	40568.8	37731.5	11725.6	16200.6	8326.9	196059.6	117757.9	109210.0	499.6
福島市	Fukushima-shi	総 合 計	0.0	0.0	0.0	642409.0	0.0	0.0	963650.2	4031562.1	449180.0	30892.4	26129.2	37572.1	35032.2	10905.6	14760.8	7992.8	183040.2	112043.4	103966.8	475.8
水戸市	Mito-shi	総 合 計	0.0	0.0	0.0	664416.5	0.0	0.0	958219.3	4008572.4	471987.7	32887.9	27687.3	39544.9	36780.4	11580.3	15590.2	8175.7	188140.2	107387.2	98828.9	517.4
宇都宮市	Utsunomiya-shi	総 合 計	0.0	0.0	0.0	682570.0	0.0	0.0	1000887.4	4187069.0	479105.6	32573.0	27474.6	37392.9	34758.7	11241.5	14583.6	7590.6	180382.1	119168.9	112000.1	547.1
前橋市	Maebashi-shi	総 合 計	0.0	0.0	0.0	648705.5	0.0	0.0	978789.3	4095006.9	448068.2	30497.3	25699.3	35676.1	33237.1	10084.3	13954.0	7914.9	161810.7	120103.6	113063.8	504.1
さいたま市	Saitama-shi	総 合 計	0.0	0.0	0.0	709667.0	0.0	0.0	1068043.5	4468002.4	497756.9	35141.4	29527.6	44567.0	41655.1	13254.0	17725.3	9030.2	192146.2	118174.3	109718.1	578.5
千葉市	Chiba-shi	総 合 計	0.0	0.0	0.0	735125.5	0.0	0.0	1047869.6	4383538.3	527753.3	35451.0	29764.1	43336.0	40365.2	13057.0	17079.8	8636.6	192400.2	113360.1	104373.8	630.8
東京都区部	Ku-areas of Tokyo	総 合 計	0.0	0.0	0.0	659830.0	0.0	0.0	953164.3	3987257.5	470814.5	32301.2	27123.7	39808.5	37098.1	12216.2	15874.5	7543.2	174142.0	104350.7	96693.2	582.1
横浜市	Yokohama-shi	総 合 計	0.0	0.0	0.0	676612.0	0.0	0.0	962442.6	4026071.5	484288.5	32648.1	27416.6	39547.4	36815.3	12223.7	15606.0	7530.9	177233.2	106868.6	98770.7	567.7
新潟市	Niigata-shi	総 合 計	0.0	0.0	0.0	790851.5	0.0	0.0	1120659.0	4688082.3	565270.8	37551.7	31673.6	43489.3	40517.8	13172.0	16997.1	8746.1	205647.3	126605.2	116601.9	644.7
富山市	Toyama-shi	総 合 計	0.0	0.0	0.0	759788.0	0.0	0.0	1120016.5	4685200.1	534094.9	37357.2	31533.2	42992.5	39826.2	12696.5	16836.9	8754.8	221677.0	129415.4	120151.0	627.6
金沢市	Kanazawa-shi	総 合 計	0.0	0.0	0.0	668579.0	0.0	0.0	986066.3	4124613.1	472245.8	33885.5	28593.9	40796.2	37869.3	12495.7	16281.7	7602.9	197492.4	107577.0	99838.3	594.8
福井市	Fukui-shi	総 合 計	0.0	0.0	0.0	607929.5	0.0	0.0	979995.2	4099512.0	409193.6	31563.0	26589.0	37149.3	34459.1	11009.2	14843.9	7262.0	180147.3	118508.9	112642.6	517.3
甲府市	Kofu-shi	総 合 計	0.0	0.0	0.0	606644.5	0.0	0.0	906694.2	3792940.4	424228.0	30183.6	25391.3	36431.7	34004.5	10841.4	14459.5	7397.4	157460.6	102345.0	95467.9	531.6
長野市	Nagano-shi	総 合 計	0.0	0.0	0.0	689421.5	0.0	0.0	1014701.9	4244660.7	482897.4	33297.5	28189.6	39949.5	37216.2	11805.7	15515.8	8426.4	190066.7	116533.4	106604.1	520.8
岐阜市	Gifu-shi	総 合 計	0.0	0.0	0.0	669655.5	0.0	0.0	1029025.0	4304554.3	462858.7	33272.3	28125.6	40171.1	37325.0	11635.3	15931.3	8314.4	191320.2	120570.8	113077.7	625.2
静岡市	Shizuoka-shi	総 合 計	0.0	0.0	0.0	701199.5	0.0	0.0	1115375.2	4666041.8	474542.9	37021.3	30841.9	41713.8	38872.3	12461.7	16438.1	8506.4	189451.8	133667.4	126443.3	617.2
名古屋市	Nagoya-shi	総 合 計	0.0	0.0	0.0	675407.0	0.0	0.0	1039425.7	4347879.3	466172.7	34537.9	29149.5	40815.1	37978.4	12372.6	16126.0	8007.0	195681.7	121312.7	114398.5	666.1
津市	Tsu-shi	総 合 計	0.0	0.0	0.0	666012.5	0.0	0.0	992814.1	4153426.8	468524.0	33792.2	28363.4	40817.0	37815.2	12064.7	16251.2	8021.8	204825.0	109409.4	100491.7	510.2
大津市	Otsu-shi	総 合 計	0.0	0.0	0.0	758935.5	0.0	0.0	1138507.2	4762751.5	529902.3	37675.7	31693.6	44318.8	41051.4	13176.7	17738.0	8516.2	215319.5	132193.0	124220.4	616.0
京都市	Kyoto-shi	総 合 計	0.0	0.0	0.0	716636.5	0.0	0.0	1022822.1	4278707.7	511966.5	34426.4	28887.0	40765.1	37797.5	12055.8	16266.4	7983.8	195999.9	115205.9	106923.7	636.5
大阪市	Osaka-shi	総 合 計	0.0	0.0	0.0	709259.0	0.0	0.0	1100733.1	4604468.6	492161.8	35831.8	30176.6	45815.6	42614.1	13290.1	18520.1	9127.9	213192.1	121765.3	114950.1	603.6
神戸市	Kobe-shi	総 合 計	0.0	0.0	0.0	656963.5	0.0	0.0	993987.0	4157743.5	458110.2	32860.7	27715.2	39326.9	36540.8	12179.9	15634.6	7282.5	187443.6	114456.0	108344.9	541.8
奈良市	Nara-shi	総 合 計	0.0	0.0	0.0	717805.5	0.0	0.0	1044528.9	4369498.6	509629.4	34707.4	29172.2	43301.4	40161.4	12745.4	17394.9	8430.8	203138.7	117098.3	108794.6	627.6
和歌山市	Wakayama-shi	総 合 計	0.0	0.0	0.0	683172.0	0.0	0.0	1073047.4	4488579.4	468266.4	34657.2	29131.1	42423.7	39375.0	12326.6	17127.6	8371.6	205199.3	123780.3	115802.9	569.5
鳥取市	Tottori-shi	総 合 計	0.0	0.0	0.0	722481.5	0.0	0.0	1125026.7	4705909.1	499568.7	38112.9	32200.8	47014.2	43637.5	13476.2	18894.0	9548.1	242220.0	124553.3	117416.8	634.4
松江市	Matsue-shi	総 合 計	0.0	0.0	0.0	679572.5	0.0	0.0	1021357.3	4272492.4	477506.9	34272.9	28879.5	42494.6	39533.5	12607.9	16908.1	8434.1	199715.8	111106.5	103533.7	598.6
岡山市	Okayama-shi	総 合 計	0.0	0.0	0.0	633005.0	0.0	0.0	924470.9	3867118.5	447652.9	31156.4	26348.8	37621.4	34973.9	11345.7	15030.0	7212.5	181496.7	102528.2	95877.0	558.2
広島市	Hiroshima-shi	総 合 計	0.0	0.0	0.0	708114.5	0.0	0.0	1051705.6	4399462.4	500766.7	34253.9	28918.6	43819.8	40743.8	12953.7	17597.0	8572.2	204677.1	114347.6	106768.7	610.6
山口市	Yamaguchi-shi	総 合 計	0.0	0.0	0.0	700310.0	0.0	0.0	1060861.6	4437695.6	492912.3	34711.5	29356.5	44829.7	41706.3	13030.1	18023.7	8998.5	216005.6	112455.6	104783.3	578.2
徳島市	Tokushima-shi	総 合 計	0.0	0.0	0.0	627944.5	0.0	0.0	951234.0	3979235.8	439650.3	30521.2	25756.0	39352.4	36591.8	11166.5	15735.7	8243.2	177823.4	105794.2	98567.1	535.9
高松市	Takamatsu-shi	総 合 計	0.0	0.0	0.0	714582.0	0.0	0.0	1128999.0	4722493.8	492384.9	36029.6	30451.4	47932.6	44667.1	13380.9	19250.9	10258.8	213828.7	124567.1	117437.0	639.2
松山市	Matsuyama-shi	総 合 計	0.0	0.0	0.0	634913.5	0.0	0.0	1017394.0	4256041.6	436603.2	32587.0	27533.9	44000.5	41011.0	12204.3	17913.9	9259.1	201525.4	108479.5	101850.9	522.6
高知市	Kochi-shi	総 合 計	0.0	0.0	0.0	646429.5	0.0	0.0	1008774.0	4219709.5	447144.4	34054.4	28614.4	41112.3	38165.2	11777.0	16640.8	8277.8	213454.3	110030.0	103136.0	527.2
福岡市	Fukuoka-shi	総 合 計	0.0	0.0	0.0	619645.0	0.0	0.0	910227.4	3807824.8	442444.5	31517.1	26480.5	40588.3	37774.4	11969.7	16464.1	7821.5	195945.0	92808.0	85243.0	500.4
佐賀市	Saga-shi	総 合 計	0.0	0.0	0.0	690877.5	0.0	0.0	1055498.6	4415568.4	483057.2	34608.0	29123.9	44753.3	41655.7	12726.3	18136.7	9126.7	201663.1	114856.9	106963.2	578.3
長崎市	Nagasaki-shi	総 合 計	0.0	0.0	0.0	683133.5	0.0	0.0	1007188.9	4213500.8	480405.4	32447.1	27187.4	39806.9	36942.8	11317.2	15840.6	8316.5	182628.6	116775.7	108467.9	590.9
熊本市	Kumamoto-shi	総 合 計	0.0	0.0	0.0	599308.0	0.0	0.0	948344.8	3967027.5	414220.3	30074.0	25281.8	40736.3	38063.6	11576.7	16647.8	8313.9	175463.0	100247.6	93114.2	500.9
大分市	Oita-shi	総 合 計	0.0	0.0	0.0	682065.5	0.0	0.0	1049149.8	4389034.3	474429.9	33761.5	28366.8	43517.8	40460.8	12310.9	17580.4	8956.1	201747.6	115373.1	108121.5	558.4
宮崎市	Miyazaki-shi	総 合 計	0.0	0.0	0.0	638991.0	0.0	0.0	972269.8	4067322.7	449180.5	31495.1	26553.1	40942.2	38131.8	11747.0	16556.3	8309.0	187123.2	101921.4	94090.2	519.8
鹿児島市	Kagoshima-shi	総 合 計	0.0	0.0	0.0	621418.0	0.0	0.0	991541.1	4148014.2	428425.9	31635.3	26628.7	44002.1	41155.4	12013.6	17842.9	9646.3	187838.5	104256.1	96314.8	515.5
那覇市	Naha-shi	総 合 計	0.0	0.0	0.0	542361.5	0.0	0.0	905611.0	3788734.1	366121.4	28223.9	23991.0	38296.0	35812.6	10766.2	15477.8	8181.7	177109.5	99521.9	94692.8	421.5
川崎市	Kawasaki-shi	総 合 計	0.0	0.0	0.0	698217.0	0.0	0.0	1028660.7	4303172.5	495271.1	35410.3	29853.4	43611.6	40659.0	13590.2	17323.0	8147.7	200059.9	110804.8	102656.7	582.8
相模原市	Sagamihara-shi	総 合 計	0.0	0.0	0.0	749359.5	0.0	0.0	1129888.1	4726654.1	523381.6	37481.5	31582.8	45332.6	42277.9	13786.5	17965.1	8867.8	201567.0	128551.5	120710.7	662.8
浜松市	Hamamatsu-shi	総 合 計	0.0	0.0	0.0	640773.0	0.0	0.0	995313.9	4163656.3	440290.3	33110.9	27836.8	39915.9	37208.8	11775.0	15600.5	8379.4	186811.7	115456.3	108352.6	576.6
堺市	Sakai-shi	総 合 計	0.0	0.0	0.0	681424.0	0.0	0.0	1058083.1	4426163.7	469900.5	35350.3	29677.7	42098.4	39051.7	12574.0	17006.7	7949.1	211168.0	121268.1	114608.6	610.7
北九州市	Kitakyushu-shi	総 合 計	0.0	0.0	0.0	660396.5	0.0	0.0	1002376.1	4193325.9	461911.6	32037.4	26932.1	40355.4	37495.3	11372.3	16374.7	8260.3	188989.1	111710.0	104466.3	551.5

Dietary fibers			Ash	Salt	Na	K	Ca	Mg	P	Fe	Zn	Cu	Mn	I	Se	Cr	Mo	Vitamin A				
高分子量 水溶性 食物繊維 (g)	不溶性 食物繊維 (g)	食物繊維 総量 (g)	灰分 (g)	食塩 相当量 (g)	ナトリウム (mg)	カリウム (mg)	カルシウム (mg)	マグネシウム (mg)	リン (mg)	鉄 (mg)	亜鉛 (mg)	銅 (mg)	マンガン (mg)	ヨウ素 (μg)	セレン (μg)	クロム (μg)	モリブデン (μg)	レチノール (μg)	α-カロテン (μg)	β-カロテン (μg)	β-クリプト キサンチン (μg)	β-カロテン 当量 (μg)
1657.7	4896.4	7562.0	8605.4	5162.0	2043644.4	1151313.2	237266.4	116902.5	482454.9	3613.0	3974.1	477.5	1748.5	729233.9	42537.2	3535.1	95061.2	127686.6	272698.8	1241954.4	192604.1	1590714.7
1778.2	5029.5	7646.8	7690.2	4181.1	1658685.2	1172098.3	223629.2	117427.8	505995.7	3583.3	4187.7	509.5	1877.6	650428.9	44398.5	3565.3	102761.9	143104.8	254103.3	1138679.0	198192.0	1479518.8
1664.8	5013.6	8090.7	11256.9	7606.0	3003496.5	1226843.5	237891.2	130949.5	529028.8	3895.8	4372.0	528.1	1651.4	1419666.6	50815.2	3998.5	107478.8	147166.4	284438.5	1273835.8	166129.3	1558449.5
1921.2	5862.3	9285.0	10005.1	6083.1	2408910.5	1320404.9	269894.2	137965.2	541047.7	4081.8	4351.8	552.8	1976.7	1437021.7	46330.2	4058.3	112636.5	154513.3	337754.4	1483006.9	215584.5	1882075.3
1702.4	5179.5	8174.3	9010.5	5381.1	2131524.6	1216266.5	261507.6	122825.1	496200.7	3664.6	3948.1	477.5	1849.3	1037924.2	43551.1	3612.1	92395.8	150121.3	287987.2	1364742.4	179925.9	1742845.4
1733.6	5491.3	8495.2	9255.9	5596.0	2217062.2	1246676.9	246909.1	125675.7	503903.7	3871.0	3973.9	481.9	1850.9	1052293.6	46723.7	3909.3	90846.6	151615.1	286384.4	1425819.3	172766.5	1801987.5
1588.4	4822.9	7450.3	9938.2	6467.4	2557268.7	1158547.2	237839.4	119787.6	482911.0	3625.7	3966.5	483.4	1613.0	1086357.4	42017.6	3442.2	99314.6	144230.3	266175.2	1230624.6	202120.5	1544339.0
1558.8	4731.0	7290.8	7287.8	4010.6	1592921.8	1098591.3	223583.3	112213.5	453508.4	3447.2	3673.4	463.8	1657.8	860221.0	38970.1	3322.1	93970.4	135352.3	269723.4	1199823.1	153639.8	1519101.7
1632.0	4827.8	7663.9	8744.9	5265.9	2085240.3	1178270.2	242976.7	118133.0	478008.9	3568.5	3720.0	450.7	1656.7	966609.9	43448.9	3586.4	86835.1	147597.9	278718.5	1280582.9	203970.5	1636085.5
1613.9	4858.7	7526.5	8596.2	5122.1	2028520.4	1150536.3	243941.3	119478.6	478527.9	3537.8	3876.3	486.0	1776.0	756937.0	41192.6	3362.2	101265.9	147219.3	321197.5	1343108.1	196172.6	1711947.8
1612.0	4789.9	7526.2	8521.7	5171.9	2047973.2	1125465.5	225830.6	117019.2	452103.8	3509.8	3683.7	482.7	1738.8	1034316.8	38508.4	3564.8	100486.6	128153.1	264974.6	1312859.4	214234.5	1649223.6
1835.9	5427.5	8314.0	8966.6	5265.7	2086214.3	1247648.3	257896.8	122940.8	509561.2	3792.8	4188.0	501.6	1889.1	782585.3	43783.0	3938.2	97010.6	151823.7	304759.3	1411531.1	164458.3	1774267.8
1882.3	5559.5	8597.7	8605.8	4758.1	1887764.3	1282839.7	278917.9	127788.9	518703.9	3890.1	4155.5	501.9	2015.2	611238.7	44703.9	3846.4	96165.7	158201.5	321362.0	1549583.4	176126.5	1970856.7
1746.3	5086.9	7848.0	7762.6	4312.7	1710590.3	1160399.3	242126.7	113806.9	467464.6	3472.9	3802.2	453.2	1702.0	656919.1	40308.6	3510.8	86305.3	139938.7	281629.5	1370766.6	185926.3	1727342.0
1767.1	5122.0	7982.5	8295.8	4747.3	1881076.7	1196537.7	252119.5	117453.1	478178.8	3480.8	3815.6	458.5	1714.0	824610.9	41431.6	3551.4	85278.4	143020.8	304692.5	1416660.4	220944.0	1805815.2
1878.1	5647.1	8791.3	10121.8	6100.6	2415868.8	1343463.7	280843.9	137143.2	548163.6	4052.6	4343.1	539.2	1921.1	1038046.5	48681.8	3950.2	109355.2	150369.0	322600.5	1527984.4	223868.1	1920081.2
1767.8	5422.5	8317.2	9425.1	5585.0	2211638.8	1278911.7	265267.8	132352.4	540210.2	4063.3	4403.2	541.7	1959.9	1069395.0	48605.6	3955.8	111337.1	162617.3	256594.5	1325303.4	205216.0	1679808.5
1584.1	4765.9	7440.7	8944.5	5533.5	2189492.5	1140573.6	240326.0	115799.4	479549.7	3506.8	3911.7	455.8	1771.6	1139596.0	44836.3	3554.4	89440.1	137778.7	268622.3	1188625.2	125851.4	1533396.3
1538.9	4585.1	7231.2	7674.3	4495.8	1780195.5	1063085.4	211078.8	111065.1	449943.9	3401.8	3904.5	477.8	1737.2	893726.8	39848.4	3330.2	97298.0	125245.1	247087.7	1110028.9	198285.2	1442815.0
1566.2	4535.4	7126.5	8605.5	5417.4	2143409.2	1071030.9	217536.9	109757.4	434433.9	3311.5	3509.9	435.3	1583.9	546728.4	38859.3	3321.2	85804.4	135057.2	265201.8	1252026.3	171534.8	1568711.1
1679.8	5056.8	7953.1	10908.6	7375.7	2912818.6	1178144.9	264318.2	119196.1	490839.5	3597.8	3789.2	461.1	1575.0	1215531.9	44688.7	3608.7	89430.0	149458.0	269391.2	1277083.4	182320.2	1598956.8
1660.9	5089.1	7742.3	7954.5	4502.2	1784660.5	1158024.3	234420.4	119336.2	478810.1	3735.7	3993.0	497.3	1822.8	696088.0	42191.3	3591.7	103588.6	145063.9	236867.9	1197816.2	173385.9	1513760.2
1814.7	5814.3	8874.8	8951.9	5189.2	2055938.1	1256967.8	254613.3	134045.5	529683.7	4255.7	4407.1	559.8	2836.7	918842.6	46218.6	3919.8	115242.3	170456.8	301397.5	1324093.5	272085.1	1926767.1
1780.5	5300.8	8054.6	8264.4	4728.9	1873330.7	1175712.6	246977.0	119491.0	494551.4	3742.9	4094.7	499.4	1885.8	379346.6	43407.4	3626.3	101547.7	149457.7	292379.7	1300582.3	145229.8	1652011.1
1642.4	5115.8	7748.3	8238.0	4732.9	1874895.9	1178939.6	244753.2	118268.1	484087.0	3775.5	3945.9	473.1	2002.8	614765.5	42418.4	3604.2	88507.7	156333.1	271175.2	1248476.4	252444.9	1704010.1
1930.2	5571.2	8595.1	9110.3	5196.7	2059413.8	1324186.2	255838.1	131370.6	532162.5	3992.8	4429.2	533.1	1978.4	1114869.2	47860.9	3943.0	104013.1	148276.0	317105.0	1516460.2	256160.4	1934733.1
1861.3	5353.1	8304.2	8391.6	4728.6	1874527.6	1232360.4	249277.4	123153.1	491595.6	3699.5	3988.1	487.3	1845.5	812498.6	44457.4	3899.9	92111.2	139208.2	272648.3	1349966.1	188671.0	1713562.9
1729.4	5012.7	7796.1	8420.5	4814.6	1906933.1	1202049.9	246768.2	120571.8	507732.4	3710.9	4311.1	498.1	1773.2	933289.0	45248.2	3589.9	98137.7	150378.0	262280.7	1250525.5	157727.2	1568954.1
1526.1	4581.2	6902.8	7649.8	4333.0	1716396.2	1090270.2	237028.5	109459.6	471257.4	3328.7	3931.8	460.9	1639.9	526887.4	40620.6	3246.9	91894.7	140550.8	241645.8	1121767.1	135789.5	1406957.9
1881.5	5431.8	8295.4	8561.2	4861.9	1926675.0	1248778.8	253842.2	122799.1	499791.5	3743.9	4100.0	493.8	1847.2	717153.3	43533.0	3802.2	93461.5	143754.2	284027.3	1345545.5	251020.6	1743525.8
1720.3	5118.0	7776.6	8604.1	5173.3	2047708.0	1159399.8	228072.9	116534.2	483496.9	3689.3	4081.2	491.2	1799.4	683625.1	43282.8	3551.1	94390.7	143235.2	226861.9	1091808.9	224875.6	1433938.4
1673.8	4969.6	7607.2	9049.5	5416.5	2142207.6	1208192.5	248592.4	124175.6	526999.4	3876.3	4460.9	517.2	1713.5	436568.2	52920.1	3587.3	101147.8	157735.0	294754.3	1253439.8	190317.9	1588652.3
1715.2	4947.7	7737.8	8462.7	4958.9	1964249.2	1174485.4	251094.6	116968.1	485510.8	3654.1	3966.5	469.6	1715.3	926217.7	43841.3	3612.7	90071.7	137405.4	310429.2	1337670.9	148023.4	1682940.9
1571.4	4421.8	6758.4	9152.1	5967.0	2357724.4	1060689.3	220739.0	105891.4	438031.6	3191.6	3582.1	422.2	1495.6	422733.9	38831.5	3165.4	83683.6	127095.4	275083.7	1140867.5	171051.1	1455579.4
1688.8	4886.5	7423.1	8624.9	5107.8	2021940.9	1177726.3	242530.6	119220.7	485083.5	3611.7	4186.9	488.0	1688.2	487786.4	42624.4	3581.7	94611.9	138714.4	285561.0	1242459.2	235034.9	1606278.9
1614.3	4662.7	7183.3	8550.0	5121.7	2027510.8	1134781.3	237058.9	115897.2	490386.0	3569.0	4056.3	469.6	1601.9	587143.6	43722.2	3425.1	94461.8	144328.9	252900.6	1134867.0	193891.4	1454444.3
1523.0	4389.5	6949.0	7612.3	4491.0	1778837.7	1028191.7	223343.7	107620.5	434655.5	3284.0	3540.2	427.2	1606.0	532074.3	37654.1	3317.6	86225.6	132382.9	231126.3	1032527.9	208475.2	1363898.9
1780.4	4906.5	7779.6	8880.6	5301.7	2099687.4	1179717.1	249187.3	121656.3	501474.6	3672.4	4163.7	487.7	1674.0	736760.1	44489.8	3972.9	96557.9	145324.7	259962.7	1173824.4	268573.3	1522492.1
1515.8	4397.8	6721.7	8010.4	4838.2	1914741.7	1060721.5	207579.4	107470.5	449048.1	3403.9	3779.0	442.4	1588.2	540630.7	40727.5	3165.4	86828.3	132385.2	240800.3	1143665.4	200651.3	1469627.7
1480.5	4389.5	6829.5	8142.6	4933.5	1951544.8	1068868.7	203655.6	111496.0	464608.8	3546.7	3915.3	454.8	1587.6	819156.0	43683.1	3217.4	89780.8	149802.9	256494.1	1138149.3	177478.7	1450980.3
1562.0	4563.1	6980.4	7670.5	4472.9	1770151.5	1081717.3	215245.2	105575.4	441457.6	3304.2	3640.2	417.7	1565.3	483795.9	39029.0	3249.4	77877.7	130754.5	255827.8	1163448.6	231483.1	1526922.1
1725.6	5115.2	7865.5	8419.1	4878.8	1931480.2	1199834.9	235328.7	120580.8	488343.0	3752.5	4120.3	492.7	1821.4	919851.4	42697.9	3624.0	97146.2	132976.1	293574.2	1345668.9	197921.8	1721273.0
1765.8	5253.3	8071.7	8282.3	4802.4	1901270.7	1182659.0	233116.9	119943.4	468172.1	3691.7	3948.4	491.1	1962.0	797152.6	39835.2	3626.9	95042.8	123697.1	265107.1	1311260.2	299144.2	1757204.6
1415.0	4243.8	6515.8	7878.7	4944.5	1954352.6	982556.1	198496.6	101068.7	418753.5	3186.3	3526.3	415.8	1558.2	553616.3	35013.4	3179.7	82182.6	119243.7	220704.0	1016911.8	207829.5	1350432.4
1723.2	4991.2	7687.7	8832.4	5395.9	2133551.7	1166228.8	225503.7	116675.0	473476.6	3593.2	3977.6	475.4	1747.2	792279.0	41378.0	3482.2	92894.2	133316.2	271989.5	1196510.5	245629.1	1569656.1
1543.3	4470.3	6815.5	7946.0	4787.9	1894911.2	1062556.2	215982.7	106453.1	441115.4	3344.5	3623.1	430.0	1527.0	466886.0	38664.9	3171.5	84872.8	131606.2	222562.3	1038165.0	250388.6	1375850.6
1577.3	4714.6	7112.4	8155.6	4998.6	1976981.1	1075403.4	207514.0	107216.1	438162.5	3485.9	3651.0	434.2	1585.7	488082.0	38504.6	3228.6	85377.0	127629.8	297360.5	1229141.0	203676.6	1589145.7
1282.6	3688.5	5618.8	5969.5	3232.7	1282682.3	909349.9	177862.8	91508.2	399548.6	3010.1	3396.1	391.4	1250.9	773957.2	37174.0	2716.8	82441.0	136176.6	352585.3	1276416.7	167571.2	1580754.9
1831.3	5243.6	8073.3	8347.2	4643.5	1840777.5	1238760.3	265410.4	119189.9	512934.6	3684.0	4123.3	471.8	1640.6	659949.8	45020.9	3613.0	88984.9	166682.5	325591.5	1486418.9	167750.1	1835860.8
1905.5	5610.8	8658.3	9108.1	5185.1	2054933.8	1300024.9	273888.0	131598.6	541990.2	4047.4	4416.8	536.9	2020.9	675588.2	46543.9	3945.6	107992.9	158592.3	328024.6	1515606.2	195057.8	1922138.8
1620.8	5161.3	7823.0	8372.9	4934.6	1953929.9	1144999.9	246299.5	119840.0	481132.7	3778.6	3945.6	491.8	2247.0	649199.6	40981.3	3436.0	101642.6	142471.5	292629.8	1266665.0	282054.7	1786818.0
1763.5	5217.7	7946.9	8136.5	4619.7	1830472.5	1179966.6	230241.8	119006.0	489982.3	3776.1	4154.0	500.2	2099.6	653894.6	44410.4	3571.9	97503.0	144501.5	253650.3	1217383.6	190894.5	1622181.2
1598.5	4839.6	7399.0	7551.7	4268.0	1691436.8	1109605.9	216229.9	114737.8	452700.1	3528.7	3836.8	474.0	1776.9	596423.1	38670.0	3382.7	95108.3	118960.8	252216.3	1159289.6	202593.6	1511487.4

	Vitamin D	Vitamin E				Vitamin K	Vitamin B									Vitamin C						
レチノール活性当量（μg）	ビタミンD（μg）	α－トコフェロール（mg）	β－トコフェロール（mg）	γ－トコフェロール（mg）	δ－トコフェロール（mg）	ビタミンK（μg）	ビタミンB_1（mg）	ビタミンB_2（mg）	ナイアシン（mg）	ナイアシン当量（mg）	ビタミンB_6（mg）	ビタミンB_{12}（μg）	葉酸（μg）	パントテン酸（mg）	ビオチン（μg）	ビタミンC（mg）	アルコール（g）	硝酸イオン（g）	テオブロミン（g）	カフェイン（g）	タンニン（g）	ポリフェノール（g）
259898.2	4306.2	5846.0	248.7	7753.0	1499.1	122994.5	494.0	525.1	8001.4	14896.5	669.1	2983.8	144926.3	2746.2	20651.4	54071.5	5328.8	58.0	0.0	44.2	209.5	0.7
266007.1	4882.8	5806.9	247.5	7635.9	1434.4	110818.7	515.4	523.5	8766.9	15927.0	716.9	3050.7	144436.3	2893.0	20741.2	53662.4	7109.6	59.1	0.0	48.5	227.4	0.8
276645.1	5984.3	6346.6	234.6	8805.8	1711.8	133040.0	531.3	560.0	9110.0	16622.9	724.5	3951.5	149556.2	2903.4	23448.6	54904.6	7817.5	58.6	0.0	33.9	152.8	1.0
310963.1	5181.6	6314.6	259.6	7926.2	1567.0	146169.5	544.5	572.3	8717.4	16320.6	754.0	3377.6	165005.4	3024.3	23154.9	62095.2	6072.5	64.5	0.0	44.9	213.4	0.9
294950.9	4507.6	5971.3	254.3	7265.9	1423.7	137073.1	504.1	547.6	7862.7	14750.9	673.3	3211.3	153355.4	2781.2	21645.9	57798.3	5460.6	61.4	0.0	51.2	244.2	0.6
301429.1	5257.1	6417.7	255.4	7507.7	1469.1	147201.2	524.7	566.4	8782.3	15904.2	712.2	3593.3	164373.5	2839.2	23278.5	61564.6	7616.7	66.5	0.0	48.3	234.7	1.1
272484.9	4342.0	5723.7	234.3	7806.2	1546.2	120295.3	480.0	521.7	7845.8	14674.3	654.2	2886.0	143416.0	2716.0	21088.0	52735.7	5720.1	55.5	0.0	37.5	172.4	0.6
261585.7	4158.2	5650.9	231.4	7549.1	1497.5	117876.9	471.9	487.0	7209.0	13576.5	617.3	2909.1	137063.8	2528.4	19921.6	50639.2	5375.3	52.7	0.0	38.0	182.4	1.0
283625.2	4891.1	5826.3	237.4	7519.3	1439.6	130840.9	490.1	525.9	8129.3	14823.0	670.3	3503.2	147447.0	2657.9	20819.3	56669.2	3844.8	59.8	0.0	45.3	214.4	0.7
289582.9	4192.8	5496.4	226.1	6883.5	1366.1	121374.6	487.5	514.4	7634.3	14334.3	657.3	2849.8	147184.2	2728.8	20245.3	54705.6	5665.8	57.3	0.0	44.0	206.9	0.6
265508.1	4145.0	5513.2	231.9	7731.6	1519.5	129861.9	472.4	468.5	7197.0	13461.1	636.0	2801.6	144969.2	2532.9	19158.9	55659.6	5715.1	62.9	0.0	39.5	184.0	0.7
299393.8	4218.7	6192.0	271.6	8517.1	1638.2	140451.6	535.1	551.4	8401.8	15554.7	702.3	3042.3	159083.4	2887.5	21141.9	59723.3	4930.0	66.3	0.0	53.6	247.2	1.2
322224.6	4473.0	6362.0	280.2	8076.2	1574.9	150406.0	529.5	577.8	8477.2	15726.5	716.4	3313.3	170066.2	2930.6	21593.5	64180.4	6477.9	71.8	0.0	55.2	268.9	0.9
283687.7	3795.1	5498.5	227.3	6815.7	1311.0	131523.0	489.7	507.5	7831.6	14402.8	648.8	2847.5	152426.6	2638.3	19267.0	60040.4	4650.4	65.6	0.0	51.0	237.2	0.8
293267.1	4091.0	5591.5	232.1	6760.8	1298.6	128707.0	496.4	524.6	7829.6	14471.8	668.2	3076.6	150758.2	2697.0	19821.8	60648.3	4848.2	60.5	0.0	51.8	242.2	0.6
310002.0	5294.5	6295.6	252.3	7883.5	1578.7	138282.3	576.3	607.1	9082.8	16753.2	751.8	3719.7	166365.4	3110.6	23719.1	63216.8	7723.2	64.7	0.0	45.1	214.7	0.8
302103.8	5023.3	6534.0	257.4	8090.4	1599.5	133056.8	544.5	595.4	8563.8	16222.6	723.1	3171.8	163111.0	3025.0	23860.8	59676.4	6499.0	63.8	0.0	51.2	239.7	0.9
265203.1	4433.7	5757.5	246.9	6679.6	1286.5	118092.0	496.3	538.5	7850.8	14738.7	638.6	2848.1	142237.2	2700.8	21013.1	50988.2	5030.5	53.3	0.0	53.9	259.4	0.6
245158.0	3999.5	5310.3	222.0	6598.4	1281.3	112095.0	462.1	482.4	7295.9	13774.2	624.7	2527.9	130507.6	2590.2	19124.5	47223.9	3792.5	47.5	0.0	38.0	183.3	0.6
265607.0	3863.9	5106.3	216.3	6777.2	1324.2	122140.2	464.4	458.5	7234.1	13356.8	607.7	2909.4	135624.3	2415.6	17846.9	51646.2	4770.1	60.8	0.0	41.7	193.8	0.5
282269.7	4706.2	5912.7	254.6	7981.6	1570.0	130546.3	486.2	547.2	7685.4	14504.4	637.3	3210.2	144890.0	2729.6	21186.5	53114.1	5808.9	61.1	0.0	38.6	181.1	0.8
270758.2	3969.6	5901.6	244.6	7941.2	1574.3	116872.2	488.0	517.6	7613.9	14460.9	657.2	2950.0	145811.3	2726.5	20931.2	57704.3	4819.7	54.7	0.0	41.4	196.5	0.6
331089.4	4155.2	6734.7	335.0	7662.2	1488.8	154220.8	550.2	544.8	9046.9	16597.9	735.1	3221.0	173589.1	2937.7	21669.3	61247.1	5079.3	63.9	0.0	77.6	400.7	0.5
286746.2	4019.2	5939.9	253.2	7306.0	1427.3	122595.2	510.3	538.6	7805.7	14884.9	682.4	2637.4	153722.3	2831.6	21097.9	57590.0	4196.2	60.9	0.0	45.3	217.7	0.6
297992.5	4870.5	6324.3	274.5	7305.1	1419.8	128461.8	484.6	549.6	7984.2	14900.6	687.4	3190.4	152469.3	2761.6	21647.4	58357.2	5101.7	56.5	0.0	55.6	278.5	0.8
309143.6	5130.2	6483.6	270.4	7665.7	1468.0	138595.7	546.9	584.6	9067.7	16740.5	772.7	3229.6	169997.5	3106.1	23039.0	64092.2	5660.1	72.6	0.0	48.7	232.5	0.6
281654.7	4210.6	6026.7	259.8	7443.7	1438.4	132316.9	491.3	547.3	8314.8	15306.3	687.4	2941.6	156592.7	2822.7	21486.4	60052.7	5744.2	64.4	0.0	60.4	280.1	0.6
280602.8	4468.1	6330.9	267.7	8682.7	1649.9	130001.0	512.3	557.2	8472.8	15791.8	703.1	2935.2	152247.0	2943.5	21740.9	56414.6	5234.7	63.9	0.0	45.8	214.4	0.7
257295.1	3792.2	5299.5	217.5	6469.8	1254.9	109434.8	469.3	518.9	7649.9	14399.1	635.3	2409.4	134742.0	2719.7	19533.8	50332.8	4466.1	54.0	0.0	47.5	215.6	0.6
288657.9	4603.8	6222.7	262.5	7949.2	1534.0	129528.2	500.3	559.2	8076.8	15153.0	708.2	2988.0	159026.0	2897.4	21898.8	64201.6	4387.1	64.9	0.0	52.9	248.8	0.8
262234.8	4372.5	6004.2	258.0	7799.9	1507.0	117281.1	483.7	527.8	8203.3	15261.3	688.8	2858.6	141994.5	2804.8	20976.4	53130.6	5336.1	54.5	0.0	47.2	221.8	0.7
289544.8	5464.5	6713.5	268.4	8983.3	1706.4	124547.5	520.7	612.8	8669.3	16443.3	721.8	3585.7	151021.5	3045.2	24528.9	53149.0	4273.6	55.8	0.0	43.5	201.2	0.6
277213.6	4383.5	5935.3	247.5	7770.9	1492.3	127029.6	494.4	556.9	7888.3	14884.9	669.1	3941.1	147433.6	2788.5	20949.3	54580.8	5655.3	59.4	0.0	43.0	204.5	0.8
247942.6	3899.7	5203.5	220.3	6475.3	1262.4	102573.4	448.7	488.5	7161.4	13498.8	607.1	2464.4	131208.9	2519.0	19241.2	48563.6	4809.5	53.1	0.0	42.1	195.6	0.5
272106.3	4217.7	5971.9	255.0	8057.3	1581.7	116099.9	502.3	546.8	7882.8	14886.6	686.3	3079.1	146040.7	2837.3	21403.0	55700.0	6259.0	56.4	0.0	45.0	211.0	0.7
264963.5	4510.5	6124.3	255.7	8510.9	1661.4	116629.2	497.3	546.2	8155.3	15275.7	665.0	2907.4	141638.6	2814.7	21644.8	51119.7	6875.9	56.9	0.0	41.3	193.8	0.8
245642.9	3883.0	5537.6	254.9	8065.6	1569.5	114917.0	432.2	481.2	7069.3	13342.8	596.3	2480.1	126377.4	2485.7	18994.9	47006.3	4966.8	47.2	0.0	44.4	209.8	0.6
271598.5	4422.0	6487.1	314.6	10185.6	1988.3	125555.8	517.4	559.6	8115.9	15479.1	684.8	2795.3	141094.5	2904.8	21830.7	52534.3	4779.2	55.6	0.0	45.4	206.1	0.9
254436.6	4258.6	6097.5	276.5	9063.8	1745.0	112567.0	464.2	494.5	7700.0	14376.2	635.2	2701.5	131194.1	2586.1	19911.4	48606.8	5222.0	52.4	0.0	38.1	183.2	0.4
270323.9	4475.5	5795.8	230.2	7523.1	1440.2	115345.4	466.3	499.9	8588.2	15525.1	662.4	3113.1	135713.2	2622.3	20566.8	46655.7	6004.4	53.4	0.0	38.9	183.4	0.6
257710.2	3883.8	5724.1	220.5	7178.3	1350.9	121037.0	464.8	496.1	8027.6	14450.1	630.0	2690.1	138283.8	2561.7	19647.9	54683.2	4541.2	57.5	0.0	46.1	217.9	0.5
276064.2	4428.5	6309.4	267.2	8797.6	1696.7	130167.9	501.9	536.6	8220.1	15281.9	690.5	3034.8	150360.7	2821.2	21246.7	55843.9	5074.1	62.4	0.0	48.3	230.6	0.7
269868.5	4128.3	6196.4	269.5	8140.3	1578.8	129169.7	480.1	516.6	7660.1	14283.4	676.4	3077.9	152263.1	2721.4	20596.1	62055.6	5228.3	55.9	0.0	52.9	259.7	0.6
231502.8	3460.7	5489.5	246.7	8054.7	1540.4	108431.7	433.0	454.0	7236.9	13373.9	587.9	2421.8	121988.4	2431.5	17810.6	45453.6	6068.8	44.2	0.0	36.8	181.5	0.9
263756.1	4236.7	6103.0	260.0	8571.4	1645.5	125746.8	487.4	527.6	8155.7	15045.7	689.7	3075.9	145861.4	2807.2	20909.9	56061.3	6029.4	59.1	0.0	44.7	211.9	0.7
245897.1	3678.3	5572.3	233.6	7891.0	1526.7	112550.4	452.0	487.6	7503.9	13949.4	622.0	2670.1	131683.7	2551.3	18955.7	49900.3	7468.1	54.2	0.0	34.1	164.7	0.5
259789.2	3731.5	6091.7	278.3	9681.5	1869.1	124345.8	461.6	485.4	7686.7	14119.7	634.9	2638.9	138406.5	2533.6	19449.3	51903.0	4809.5	60.8	0.0	39.1	187.2	0.4
267658.6	3494.7	5084.1	210.4	7948.4	1556.6	93986.4	411.0	424.1	6695.5	12556.9	543.6	2138.1	111838.6	2235.3	17270.2	41175.8	4358.6	45.7	0.0	30.8	133.1	0.9
319317.6	4279.9	5841.2	231.1	7128.8	1370.1	134001.2	530.8	563.5	8573.5	15797.5	704.3	3210.1	158622.9	2899.2	20945.5	61227.0	4705.6	69.7	0.0	43.5	200.9	0.6
318525.3	4686.9	6344.1	266.5	8015.8	1560.0	143613.1	576.4	580.1	8964.3	16640.7	747.2	3239.8	168296.8	3061.0	22239.5	64853.4	5354.9	71.6	0.0	51.1	245.3	0.7
291246.9	4060.0	6305.7	295.7	7964.1	1595.3	132813.7	504.0	522.4	7517.5	14337.4	652.9	2830.7	154142.3	2685.1	20832.6	57135.3	3486.5	56.1	0.0	58.1	299.2	0.7
279314.5	4252.2	6242.6	273.2	7094.7	1352.5	127122.7	505.9	540.9	8406.6	15638.2	695.6	2665.0	153063.3	2860.8	21526.9	55208.2	4547.3	59.1	0.0	60.4	293.9	0.5
244622.0	3911.5	5848.8	243.2	7962.6	1543.5	124225.4	465.4	491.2	7753.7	14299.1	656.7	2735.6	144675.6	2641.9	20000.8	54921.9	6719.9	61.6	0.0	43.4	209.3	0.6

酢酸 (g)	調理油 (g)	有機酸 (g)	重量変化率 (%)	備考欄 (メモ欄)	イソロイシン(mg)	ロイシン (mg)	リシン (リジン) (mg)	含硫アミノ酸			芳香族アミノ酸			トレオニン (スレオニン) (mg)	トリプトファン (mg)	バリン (mg)	ヒスチジン (mg)	アルギニン (mg)	アラニン (mg)	アスパラギン酸 (mg)	グルタミン酸 (mg)	グリシン (mg)
								メチオニン (mg)	シスチン (mg)	含硫 アミノ酸合計 (mg)	フェニル アラニン (mg)	チロシン (mg)	芳香族 アミノ酸合計 (mg)									
176.2	0.0	973.0	0.0	0.0	1428647.4	2569083.7	2116835.5	760282.8	509507.3	1271451.1	1508901.4	1195130.6	2726088.8	1333734.1	402995.4	1721735.5	1086987.6	1976484.4	1651215.9	2966554.0	6180684.8	1467681.7
162.1	0.0	946.9	0.0	0.0	1489391.7	2691800.3	2209771.5	806353.6	536531.6	1344308.0	1573245.0	1250004.4	2846307.3	1393561.4	420317.3	1809572.8	1129637.9	2112695.3	1755429.3	3106390.2	6431573.3	1581386.7
206.0	0.0	1021.5	0.0	0.0	1582242.3	2844427.4	2371114.5	857619.2	567461.6	1425805.8	1660302.7	1317967.2	3002862.9	1490451.1	444584.0	1907267.1	1176204.1	2248962.0	1886356.5	3352531.5	6619711.8	1720661.1
185.8	0.0	1168.6	0.0	0.0	1577965.7	2841786.3	2316766.7	838987.7	567235.6	1407699.1	1676996.6	1319815.5	3021466.7	1472732.8	447880.0	1912397.7	1204179.8	2199386.8	1830992.4	3308502.0	6842285.8	1633240.2
154.5	0.0	1018.4	0.0	0.0	1431806.5	2573312.6	2129945.6	757922.4	503021.3	1263110.1	1509377.0	1200298.4	2732472.3	1336653.5	404230.2	1728463.1	1091921.7	1955349.1	1640548.5	2974947.4	6183447.2	1463425.3
158.1	0.0	1047.8	0.0	0.0	1497361.1	2672255.9	2263686.2	799234.5	521207.8	1321265.1	1558827.5	1241565.4	2822923.7	1408314.9	419179.3	1798104.8	1137850.4	2074204.1	1757889.9	3150587.7	6279188.3	1560389.7
160.6	0.0	978.5	0.0	0.0	1424098.0	2560288.8	2093278.1	751474.0	510280.7	1263245.2	1507772.7	1191574.0	2721544.2	1326208.2	401847.6	1721392.6	1074400.6	1972197.9	1640533.7	2971413.9	6121481.6	1449463.4
208.2	0.0	1000.2	0.0	0.0	1320759.5	2377754.8	1947995.1	700644.7	474551.6	1176309.1	1400839.7	1111897.4	2537252.8	1235463.9	375310.2	1601082.5	992765.7	1845387.2	1531554.7	2800796.9	5631389.3	1359583.0
141.8	0.0	999.8	0.0	0.0	1399812.8	2507175.5	2101809.4	745678.8	488405.6	1235573.5	1464480.5	1162501.4	2647506.4	1312168.7	393077.5	1685694.4	1096659.2	1908340.7	1622206.4	2910236.5	5993624.9	1431883.1
176.1	0.0	982.4	0.0	0.0	1383226.3	2494747.4	2026118.9	732175.9	498897.0	1232853.8	1471113.8	1163982.7	2656947.3	1287561.7	393130.3	1678213.6	1051341.7	1918212.0	1587016.0	2880079.9	6017382.7	1403322.9
202.8	0.0	988.2	0.0	0.0	1291055.4	2329698.4	1883576.1	682598.6	468575.4	1152384.8	1379998.5	1084933.0	2485182.5	1203473.2	368177.1	1570715.6	977030.9	1814369.7	1497111.7	2725716.5	5617754.1	1324584.8
191.7	0.0	1088.2	0.0	0.0	1479670.7	2662930.7	2201555.2	782726.3	520705.4	1305526.7	1560564.7	1240279.5	2823082.6	1379726.0	416869.9	1787726.5	1132711.3	2035639.5	1706342.3	3068794.0	6447314.9	1526791.9
178.9	0.0	1115.7	0.0	0.0	1497284.0	2688981.1	2243233.9	789291.4	519147.6	1311246.7	1573175.3	1253565.3	2846479.5	1395632.7	422279.2	1800826.7	1155913.5	2039611.0	1707024.9	3104042.5	6493081.8	1525385.2
169.2	0.0	998.2	0.0	0.0	1358367.7	2441813.1	2037205.6	717237.7	472685.1	1192343.9	1428422.9	1138436.0	2585264.9	1267598.2	383029.4	1635355.4	1051398.4	1861190.5	1561770.4	2816701.4	5933239.1	1399592.3
154.6	0.0	1051.7	0.0	0.0	1378007.1	2476766.5	2068041.7	728177.2	477207.3	1207840.9	1446751.7	1152416.4	2617873.7	1284120.9	387599.0	1659053.5	1063157.1	1872314.8	1574726.6	2849911.9	5998216.8	1407936.3
189.2	0.0	1121.0	0.0	0.0	1599270.0	2873072.7	2381690.6	842372.8	561193.2	1405687.9	1686806.8	1337306.3	3049070.2	1493873.0	450457.5	1924052.8	1193993.3	2210808.6	1847927.5	3366538.6	6891545.1	1657309.8
172.1	0.0	1101.5	0.0	0.0	1583282.6	2845672.7	2330312.7	842358.6	569410.9	1413078.8	1677253.2	1328369.1	3032986.1	1473757.4	446568.7	1914433.0	1196261.5	2199338.8	1818132.4	3308612.2	6764746.0	1611011.2
151.4	0.0	932.2	0.0	0.0	1430011.9	2567672.4	2124548.5	755948.7	505560.3	1263465.8	1508349.5	1194212.2	2724244.4	1330527.2	400515.9	1715013.2	1081045.4	1950677.7	1633008.2	2942214.3	6264782.0	1467997.0
144.8	0.0	901.5	0.0	0.0	1326696.1	2398884.5	1942187.8	712598.3	485146.6	1199066.4	1413458.9	1115231.2	2550524.4	1239875.6	378088.6	1611710.3	998143.3	1875777.4	1547365.7	2778524.6	5723966.3	1373034.0
157.9	0.0	915.1	0.0	0.0	1273661.5	2289786.5	1900319.6	672269.2	448933.1	1122994.9	1344767.7	1060363.3	2423104.4	1192089.3	358942.9	1532988.6	989262.9	1768237.2	1477547.8	2658840.7	5570439.2	1336407.2
173.0	0.0	1027.5	0.0	0.0	1425154.9	2563520.9	2086113.5	747668.5	503408.5	1252982.2	1508739.4	1194289.4	2724289.6	1320283.1	401050.2	1717943.9	1058975.8	1914490.0	1608160.6	2923683.8	6256398.4	1428051.3
170.8	0.0	998.2	0.0	0.0	1409328.2	2536735.1	2051807.2	740490.3	513381.5	1255383.2	1503940.0	1184528.8	2711075.6	1311443.9	399866.6	1704185.5	1065535.2	1961266.3	1612562.4	2936971.5	6133866.7	1431122.7
147.5	0.0	977.0	0.0	0.0	1546892.2	2789974.0	2296084.3	827459.0	553561.8	1382585.1	1638575.4	1295647.2	2956035.0	1449447.3	439442.6	1877306.8	1233231.9	2190600.3	1814343.8	3255404.5	6631880.0	1616432.8
209.2	0.0	1026.4	0.0	0.0	1452464.3	2617027.5	2110339.6	762927.8	528328.2	1292993.2	1549926.6	1221699.1	2794121.9	1347617.7	412015.7	1755873.6	1092309.9	2000649.0	1651860.4	2992681.0	6437222.8	1471692.9
209.8	0.0	1027.8	0.0	0.0	1432333.4	2563878.6	2152993.9	765171.3	502671.0	1269649.6	1496778.1	1189657.9	2707389.4	1341349.6	402434.0	1721948.2	1111335.1	1966168.8	1650388.0	2979885.4	6036111.1	1455708.2
175.9	0.0	1109.0	0.0	0.0	1581343.8	2842467.8	2348717.6	846008.6	569018.2	1416584.0	1671418.3	1319266.3	3013433.9	1478054.9	446671.5	1907377.9	1212620.5	2192070.3	1834112.6	3284871.4	6893247.4	1627774.9
151.4	0.0	1055.1	0.0	0.0	1447271.1	2597834.8	2142369.2	763578.9	516102.2	1281333.4	1529060.0	1209450.0	2756505.7	1347363.3	407771.5	1738257.0	1098430.5	1981558.0	1653427.4	2989362.5	6373626.3	1470788.2
165.3	0.0	984.9	0.0	0.0	1509036.3	2715713.1	2241309.8	808322.2	541275.9	1351394.5	1591461.8	1264905.7	2879500.6	1410615.8	426420.8	1821081.7	1148202.2	2094723.1	1746160.4	3114646.6	6517892.8	1552238.5
169.3	0.0	952.6	0.0	0.0	1387434.3	2502688.2	2048621.6	738361.2	495472.2	1236084.5	1467716.0	1168379.8	2656318.3	1289103.9	392733.2	1675063.2	1054655.4	1904680.4	1580847.5	2837360.3	6056389.5	1405648.4
185.7	0.0	1108.2	0.0	0.0	1463109.9	2625610.7	2178743.3	774908.9	518588.2	1295675.7	1540961.2	1221330.9	2782479.9	1364537.9	411851.1	1759329.5	1109318.0	2004555.6	1672625.6	3036712.2	6351656.6	1482067.2
231.1	0.0	962.5	0.0	0.0	1460097.7	2626372.5	2160573.4	781896.6	528029.2	1311223.7	1543917.1	1219615.6	2785248.0	1366575.0	412474.0	1761338.0	1127420.5	2037406.0	1696004.2	3035681.3	6299664.4	1505855.5
140.5	0.0	985.2	0.0	0.0	1613988.6	2892118.5	2398186.2	868321.6	581063.1	1450246.9	1696283.8	1343974.5	3063854.9	1508946.2	453017.6	1937713.9	1208192.3	2229449.3	1863532.7	3340158.1	6885441.5	1646609.8
186.1	0.0	979.3	0.0	0.0	1447023.7	2595915.6	2168032.9	770697.2	508103.7	1280756.7	1520486.9	1208714.1	2748309.2	1353747.5	407247.9	1736880.7	1091750.1	1986493.0	1663689.7	2992267.4	6249844.6	1478053.8
191.5	0.0	926.5	0.0	0.0	1315037.4	2359592.0	1940909.9	690744.3	470640.0	1163011.6	1391435.4	1097792.8	2507904.1	1223078.9	369105.0	1577472.8	994153.2	1793114.9	1496498.5	2713244.6	5811954.8	1335195.9
165.0	0.0	1017.4	0.0	0.0	1451506.4	2605402.1	2154375.5	765665.7	518422.3	1286077.8	1531664.8	1211375.3	2764310.5	1353813.9	408376.9	1742451.9	1089563.9	2002209.9	1665204.4	3003684.9	6300596.2	1481090.5
142.7	0.0	934.7	0.0	0.0	1476338.5	2648705.6	2205610.0	788441.4	525822.5	1315930.3	1551638.2	1234578.4	2808256.7	1380345.9	415699.2	1774319.5	1111697.2	2045739.6	1703013.3	3068473.0	6289458.4	1509955.9
150.0	0.0	888.4	0.0	0.0	1289388.7	2317231.8	1902105.7	683086.4	462512.0	1147401.0	1365714.1	1078958.9	2463369.6	1201313.9	365352.1	1553162.0	992917.8	1769470.7	1469420.2	2665207.3	5601379.2	1298849.8
171.7	0.0	1002.0	0.0	0.0	1516884.4	2727281.9	2228196.6	803072.1	550963.9	1355586.9	1608999.8	1266499.0	2897918.6	1411413.7	428492.9	1822511.6	1147259.6	2076342.9	1726871.3	3109371.0	6682493.5	1537596.4
180.3	0.0	856.0	0.0	0.0	1376158.4	2468846.3	2057382.6	739066.6	496791.0	1236934.2	1450758.0	1149291.6	2621459.2	1291584.3	389148.3	1654461.6	1062165.5	1923203.0	1601789.6	2860680.9	5898552.3	1432668.4
172.3	0.0	833.2	0.0	0.0	1430158.2	2561260.5	2160348.5	781676.0	513989.4	1295960.8	1495592.5	1191617.4	2709518.8	1351773.3	404688.6	1724811.7	1178270.0	2009749.8	1691135.8	2989779.0	5953228.7	1490037.0
167.0	0.0	905.8	0.0	0.0	1333149.0	2378743.6	2034132.7	711907.7	460340.9	1173523.3	1380755.1	1110033.1	2507487.6	1250548.9	373134.2	1595608.9	1021164.6	1841043.1	1552243.0	2779876.7	5578201.1	1366978.5
182.4	0.0	973.4	0.0	0.0	1455866.6	2610835.2	2182164.0	777307.9	515830.6	1294739.3	1532401.2	1214117.7	2766115.6	1363821.9	410390.9	1751704.9	1102947.1	2043765.6	1695937.1	3066684.3	6207019.0	1505012.6
208.6	0.0	1026.7	0.0	0.0	1358995.6	2440732.4	2015838.6	721750.2	485369.0	1208700.3	1437143.1	1133587.4	2588984.2	1269551.5	384127.7	1640078.6	1031025.3	1894988.9	1571869.3	2860432.4	5821728.9	1380957.2
158.8	0.0	840.3	0.0	0.0	1265581.6	2270670.2	1894545.7	674566.4	447910.8	1124073.7	1328853.5	1054909.5	2400516.4	1184185.8	356751.5	1520758.1	963826.9	1772174.2	1475180.1	2634427.9	5396009.8	1311581.0
225.7	0.0	970.5	0.0	0.0	1420906.2	2549153.3	2127815.1	762193.9	507493.2	1271159.9	1491881.8	1181795.7	2693144.9	1333231.7	400913.3	1710018.6	1084027.3	1986285.4	1659094.5	2974378.8	6037292.3	1461953.9
195.3	0.0	882.4	0.0	0.0	1333463.5	2388331.1	2007254.6	710464.1	468568.8	1180692.1	1396786.9	1110110.2	2524437.8	1248900.7	375058.1	1599624.0	1033737.2	1848708.5	1545261.8	2770726.3	5664764.3	1366307.3
243.4	0.0	875.1	0.0	0.0	1335265.3	2387354.6	1998691.2	709474.4	472653.6	1182977.7	1399509.0	1105058.7	2522975.2	1252051.7	374240.0	1603807.0	1033606.8	1858707.7	1558185.7	2787630.5	5678388.2	1378328.4
108.3	0.0	720.5	0.0	0.0	1204130.5	2166487.5	1775975.5	650107.5	439163.1	1089616.2	1274768.0	1016102.6	2313066.3	1126453.7	342517.1	1467233.1	942839.6	1687954.3	1396852.4	2489442.9	5104635.9	1229781.0
211.2	0.0	1043.5	0.0	0.0	1498711.3	2692175.7	2254372.7	794375.4	517296.6	1314228.5	1568160.2	1255592.0	2844390.4	1397043.6	421217.3	1804838.0	1161592.2	2031392.2	1713548.4	3074686.5	6499245.6	1527110.5
193.7	0.0	1108.2	0.0	0.0	1584260.9	2854443.1	2361522.1	840038.2	557598.2	1400228.2	1672131.0	1329037.8	3024405.8	1478496.9	448384.7	1912522.8	1215212.4	2196510.7	1826640.8	3291554.3	6870452.4	1634033.3
143.9	0.0	962.2	0.0	0.0	1400735.7	2522301.1	2069515.1	740533.6	499276.6	1241475.4	1485566.4	1177453.7	2686321.6	1306449.9	397002.7	1694888.1	1067108.8	1952916.0	1611971.6	2936508.7	5977475.0	1429663.5
164.1	0.0	954.8	0.0	0.0	1481697.9	2663994.8	2197076.3	794214.5	538023.9	1333544.5	1567115.4	1241992.5	2831476.8	1387096.6	420337.0	1785754.4	1139538.9	2072592.1	1720059.3	3075156.8	6413445.0	1533239.5
158.2	0.0	952.4	0.0	0.0	1353467.2	2427548.8	2015456.9	719847.0	485469.8	1206739.5	1426671.0	1128115.3	2572729.0	1267999.1	382326.3	1627806.8	1014458.5	1916963.9	1584525.9	2854030.9	5758613.7	1402426.5

								飽和脂肪酸														
プロリン (mg)	セリン (mg)	ヒドロキシプロリン (mg)	アミノ酸合計 (mg)	アンモニア (mg)	脂肪酸総量 (g)	n-3系 多価不飽和脂肪酸(g)	n-6系 多価不飽和脂肪酸(g)	4:0 酪酸 (mg)	6:0 ヘキサン酸 (mg)	7:0 ヘプタン酸 (mg)	8:0 オクタン酸 (mg)	10:0 デカン酸 (mg)	12:0 ラウリン酸 (mg)	13:0 トリデカン酸 (mg)	14:0 ミリスチン酸 (mg)	15:0 ペンタデカン酸 (mg)	15:0 ant ペンタデカン酸 (mg)	16:0 パルミチン酸 (mg)	16:0 iso パルミチン酸 (mg)	17:0 ヘプタデカン酸 (mg)	17:0 ant ヘプタデカン酸 (mg)	18:0 ステアリン酸 (mg)
1951625.4	1658057.1	124687.1	32637231.1	651575.2	36666.7	1339.2	6788.7	136730.8	89264.9	762.4	55990.4	130821.4	216079.9	2287.2	825058.6	77423.6	20460.3	7222043.5	9680.3	103116.3	19503.9	2997540.3
2018089.7	1719486.4	137605.8	34240466.6	681966.6	37975.3	1424.2	6956.5	139713.0	90881.3	687.0	57139.1	133856.7	223566.5	2061.0	864936.5	77962.4	20838.5	7524906.4	9779.1	103029.0	19831.8	3152317.6
2048928.0	1841725.3	139218.0	36128117.7	709780.4	39538.4	1639.3	7571.1	113606.1	74231.4	624.8	47218.7	112787.6	198235.9	1874.4	808442.3	73739.8	17042.0	7537238.3	8007.9	105939.4	16285.3	3161515.0
2156147.1	1837842.5	131330.9	36171286.3	741453.6	37792.1	1558.8	7058.5	143898.3	93947.6	797.1	59105.0	137012.2	226965.8	2391.3	880828.9	79792.1	21599.8	7403311.6	10144.6	102840.4	20621.3	3080258.9
1976993.7	1665048.6	125550.5	32686950.3	670099.3	36141.4	1345.9	6497.1	153445.0	100068.1	867.7	61196.9	143702.0	222769.6	2603.1	879384.0	81510.0	22965.4	7238719.2	10881.5	104545.7	21917.4	3068034.1
1965924.4	1738886.1	135877.6	34040121.6	670697.9	36639.3	1440.9	6718.7	122238.4	79895.9	713.8	49897.2	118883.7	198262.9	2141.4	800728.0	72709.1	18322.4	7269936.6	8689.9	101829.6	17476.0	3070699.5
1937437.2	1665739.2	116641.5	32459033.3	656949.9	36031.4	1387.1	6774.0	130941.4	85393.4	735.0	52692.0	122965.9	193188.0	2205.0	796881.5	75839.4	19663.0	6969193.5	9242.1	101917.1	18760.4	2887651.7
1779005.2	1552234.3	113137.2	30198313.9	615231.2	33486.7	1327.2	6509.5	120750.2	78794.4	682.2	48790.8	115152.0	186473.4	2046.6	724173.1	65826.7	18111.1	6453875.3	8541.3	88187.6	17282.8	2744918.9
1890047.1	1617076.3	121629.6	31901902.7	640959.6	35071.3	1452.9	6539.0	134165.5	87632.5	770.9	55133.0	127957.0	210660.9	2312.7	808198.4	74865.3	20091.6	6803741.1	9526.0	97473.2	19095.8	2851680.7
1905422.0	1615094.8	110793.9	31661403.4	651049.7	33146.9	1259.2	6156.5	140897.4	91923.0	792.1	57319.1	132440.7	212014.1	2376.3	796375.1	74567.8	21083.2	6595093.3	9984.9	94333.4	20047.9	2739045.5
1757428.7	1508153.1	101366.2	29633733.4	614379.5	31718.4	1342.5	6411.4	117060.3	76315.7	622.3	47710.8	110698.2	181064.5	1866.9	679285.8	63003.5	17567.5	5940289.4	8213.1	80903.3	16791.9	2489748.7
2046347.6	1708201.4	134902.3	33901930.0	685548.3	39565.5	1473.6	7367.6	152441.6	99486.6	843.7	62144.7	145496.3	239228.9	2531.1	900269.4	83402.1	22863.7	7762938.1	10755.6	109878.5	21834.9	3283579.0
2069337.0	1720232.1	188840.3	34172499.2	687487.2	72134.8	1447.6	6996.3	202149.3	143681.2	34740.7	102093.0	157279.7	249466.0	36656.1	936634.6	88780.2	58982.4	7597874.9	45730.2	111280.4	57785.9	3165748.6
1886364.1	1559199.3	124128.7	31129938.6	624978.2	35230.7	1238.7	6129.3	149257.8	97205.4	774.6	60413.4	140917.4	227169.6	2323.8	866800.1	81603.1	22298.1	7137529.1	10494.7	106176.6	21280.8	2982474.5
1920157.9	1580370.8	123142.6	31499520.7	635130.5	34994.7	1268.9	6089.1	159560.9	103942.1	843.5	64368.8	149153.8	236432.0	2530.5	890428.7	83787.0	23837.4	7100423.9	11243.7	105129.8	22745.2	2970612.3
2186397.2	1851347.9	139795.8	36549841.0	746683.9	38680.4	1498.6	7074.4	153678.5	100541.7	940.2	62543.7	147011.7	239049.9	2820.6	915225.7	83002.4	23083.7	7735017.6	10967.5	110047.6	22001.7	3285821.0
2134759.4	1856481.2	126114.3	36058888.6	730391.0	37692.7	1502.7	7033.7	146332.1	95595.2	820.8	59962.1	139486.4	229704.5	2462.4	866306.9	81797.4	21971.0	7429360.2	10330.4	109214.4	21020.4	3078541.7
1993704.8	1658212.6	128791.4	32687765.4	662400.3	35836.5	1274.4	6141.1	141332.9	92254.4	783.7	57625.8	135790.0	222664.2	2351.1	865312.5	81596.9	21141.5	7308199.1	10003.6	111737.3	20179.3	3060006.4
1791226.5	1552584.0	110524.3	30395884.4	610734.4	32615.7	1197.2	5898.6	118188.0	77141.6	656.9	48226.3	113304.8	186219.9	1970.7	741654.9	70567.9	17642.1	6498886.3	8386.9	97454.5	16807.6	2671041.7
1753589.4	1464320.3	116468.8	29274276.1	596082.6	32412.2	1247.7	5971.1	117162.5	76578.9	659.2	49123.3	114609.8	199244.6	1977.6	733143.0	66912.5	17524.7	6382611.1	8307.0	91729.0	16645.1	2713335.0
2013737.2	1661426.8	114200.6	32483928.5	677188.0	35487.5	1430.1	6825.2	156883.9	102512.5	911.4	64336.6	146816.2	236756.8	2734.2	843185.3	78435.4	23530.5	6870365.5	11136.4	94302.1	22402.3	2874374.9
1930717.1	1656834.0	111104.3	32233745.8	659808.1	35416.6	1359.7	6763.2	130225.1	84986.2	719.2	53270.7	124621.9	205466.4	2157.6	783684.5	74721.6	19465.7	6859454.9	9219.5	99350.1	18545.2	2825210.7
2071809.0	1779787.8	131891.3	35510470.0	709071.4	37000.5	1435.4	6846.6	139523.6	90809.1	702.9	56910.2	133175.8	220222.9	2108.7	842666.7	77760.7	20801.5	7362187.8	9797.6	104144.8	19802.3	3070116.2
2042711.7	1704925.9	119512.5	33313402.0	688100.9	35924.0	1253.2	6553.6	145993.6	95354.6	786.1	61271.0	140779.6	242191.7	2358.3	842870.6	78762.5	21851.6	7234279.1	10288.1	101859.8	20813.6	2991921.3
1906571.3	1660700.5	122253.8	32479914.4	633415.9	35857.0	1390.7	6435.2	138071.0	90250.6	796.7	57736.6	130949.9	218350.1	2390.1	841810.5	80719.8	20630.3	7099120.9	9831.4	107595.4	19533.6	2901141.1
2162229.2	1834869.4	133962.7	36221163.4	726543.1	38853.1	1468.6	6856.2	144239.4	94134.8	773.0	59749.5	138213.3	233578.4	2319.0	916666.6	87448.7	21507.4	7769809.2	10201.6	117755.2	20474.9	3152311.3
2012661.5	1677843.6	116188.3	33097313.7	668421.4	35870.6	1311.7	6503.2	143543.8	93615.5	766.0	58889.5	135568.9	222172.1	2298.0	843164.1	82073.1	21453.7	7109853.4	10121.3	105846.6	20451.6	2870355.7
2048986.2	1757745.4	130464.4	34469897.4	679780.9	40345.7	1487.8	7441.1	140630.3	92008.0	827.3	58171.0	135937.6	228829.8	2481.9	889860.9	85642.0	21071.4	7857379.0	10010.9	116357.1	20082.8	3215123.8
1930305.1	1609276.3	116921.3	31685641.8	632575.9	34588.8	1149.3	5959.3	155966.9	101830.5	875.0	63686.9	146418.5	235670.5	2625.0	875863.0	83892.5	23281.8	7107690.0	11080.5	105522.1	22178.0	2898665.3
2009304.7	1699143.6	122348.2	33362072.7	667114.1	38080.5	1405.2	6844.0	150278.0	98065.8	832.2	61189.8	141580.8	228035.1	2496.6	897097.8	87614.0	22451.8	7494302.6	10649.1	114309.6	21401.8	3045954.5
1977187.7	1701587.5	123991.0	33397740.2	658358.1	37379.2	1391.2	6800.7	125218.9	81948.9	750.2	51784.6	121949.5	206459.0	2250.6	824216.4	79305.0	18679.3	7353359.3	8978.1	110085.8	17743.8	2981306.5
2161093.4	1892186.4	131623.4	36664645.0	718571.9	41179.5	1585.1	7748.1	134885.5	88436.2	820.9	56853.4	132274.9	230898.7	2462.7	870755.5	83391.9	20229.5	7981940.4	9635.6	114224.0	19266.7	3245204.7
1975154.5	1672170.1	126696.4	32985365.0	652116.2	37379.9	1391.4	6870.8	145863.5	95241.8	854.0	58757.9	137973.6	218523.9	2562.0	855392.2	81529.8	21810.3	7400624.6	10400.1	106900.7	20756.9	3054233.6
1848488.4	1533267.9	113239.3	30108282.0	611788.8	33089.4	1162.3	5888.1	128409.7	83968.6	757.5	52626.9	123230.2	202957.2	2272.5	775268.5	73271.3	19228.8	6671715.0	9150.3	97095.9	18322.0	2743512.0
1997023.9	1691768.1	127607.7	33132853.3	657909.5	38663.9	1364.5	7037.6	142189.6	93038.8	837.7	58943.3	136956.1	230399.3	2513.1	877113.6	83825.2	21218.7	7657248.5	10168.5	112866.9	20179.1	3148279.1
1986364.4	1721329.8	130512.7	33591664.8	653329.1	39527.7	1450.2	7365.4	134594.8	88053.0	778.9	56268.5	130526.8	223431.4	2336.7	853182.7	81370.5	20151.2	7720728.4	9568.2	112392.3	19172.6	3190181.2
1775448.1	1503315.9	104368.2	29408649.0	590150.2	34639.3	1372.9	6701.3	128704.9	84125.6	756.5	52794.0	121285.4	196912.0	2269.5	761818.2	74126.5	19237.8	6555899.6	9184.5	96613.4	18281.0	2676738.2
2122636.5	1775461.2	126164.6	34693284.1	699356.6	42196.7	1660.2	8406.3	138694.1	90817.8	858.3	56905.0	133704.3	221253.7	2574.9	868647.0	83992.5	20802.9	7903742.7	9929.3	112727.1	19812.1	3233545.5
1850107.9	1605438.3	125083.5	31463373.3	612428.6	38843.1	1528.8	7555.6	109116.4	71315.3	623.4	45140.2	107146.2	183786.4	1870.2	767592.1	73408.6	16334.3	7293360.9	7745.5	105974.4	15549.5	3004798.1
1843630.2	1663846.1	126783.7	32591782.5	615809.5	36000.3	1379.8	6682.6	101026.1	65928.5	542.1	41920.9	100854.2	176548.3	1626.3	731174.7	69276.1	15093.6	7047232.1	7130.6	102676.6	14365.9	2878832.3
1753708.8	1531496.0	124065.8	30221123.7	567082.4	35701.5	1275.0	6388.4	124300.0	81006.6	635.6	51332.4	119291.8	201629.7	1906.8	785129.4	74472.4	18580.4	7084699.3	8718.5	102780.3	17715.9	2915508.5
1934342.9	1683115.5	128196.8	33222495.6	645102.7	39366.0	1509.2	7442.6	128049.4	83523.8	703.0	52026.1	122701.2	200989.5	2109.0	828841.8	79973.2	19139.7	7547971.1	9059.4	110769.1	18244.5	3086555.4
1823763.8	1578054.2	109515.6	30981505.1	616190.0	34893.0	1403.4	6750.5	125957.4	82268.3	688.3	52401.5	120034.6	202263.5	2064.9	754872.1	73188.6	18861.6	6655251.0	8888.0	96478.0	17984.6	2702283.4
1693499.7	1459181.2	117623.5	28862546.9	548296.6	36005.7	1327.4	6830.4	111307.9	72745.3	617.8	46586.7	108967.1	189445.9	1853.4	733551.3	69764.9	16663.6	6895304.3	7871.5	97903.6	15867.7	2829279.1
1884015.1	1648716.4	123733.6	32373026.4	624386.4	38259.5	1487.1	7297.4	122081.3	79816.6	729.0	49790.5	117443.2	192626.4	2187.0	796754.1	77140.0	18240.2	7335278.0	8734.3	106557.4	17341.8	2972157.1
1774561.4	1539583.5	118734.5	30329097.3	580805.2	36071.0	1362.5	6779.8	121549.5	79357.8	711.2	48853.2	115611.1	183858.6	2133.6	761863.9	73640.0	18176.4	6969868.0	8665.1	101640.9	17304.8	2865704.0
1767673.8	1543650.1	121144.0	30409803.0	587481.0	38940.4	1561.2	7922.6	106445.4	69575.1	591.6	45200.6	105561.0	189649.9	1774.8	725575.2	68756.1	15937.1	7173706.8	7526.5	98607.3	15106.7	2971277.8
1603147.6	1415767.8	104257.8	27472327.8	542238.6	33966.4	1263.5	6738.8	98257.8	64024.9	472.8	41876.6	97063.0	175315.1	1418.4	663511.5	62001.8	14819.7	6412963.0	6774.7	89026.6	14137.5	2693707.5
2076405.9	1719572.8	137863.1	34202552.8	682599.8	38545.1	1329.8	6613.8	171115.1	111433.9	900.3	69270.5	160371.6	255331.6	2700.9	970900.7	91193.1	25600.2	7904175.7	12030.9	116942.0	24392.5	3314403.2
2172634.7	1823298.1	143647.6	36288284.5	726370.6	40174.0	1447.2	7221.3	159109.5	103895.9	875.8	66053.5	152983.4	258102.3	2627.4	946123.7	87431.7	23861.3	8076545.9	11218.4	116831.8	22730.3	3407172.9
1895992.0	1632868.8	117915.6	31964288.8	643062.0	35352.6	1367.7	6833.3	139508.5	90925.1	766.6	55779.4	131523.3	207038.2	2299.8	796248.8	73842.7	20898.1	6902085.7	9848.0	95374.0	19961.2	2905073.0
2011314.6	1729892.9	129552.8	33929831.3	668682.8	36963.8	1269.7	6487.4	124588.4	81474.6	706.9	52027.9	122221.2	211212.6	2120.7	825298.2	78414.1	18623.4	7485000.7	8850.3	111179.9	17731.7	3059165.1
1786563.6	1573055.1	116205.7	30873908.1	596008.8	35587.8	1348.1	6758.4	104916.8	68713.9	607.2	44340.6	103755.7	183554.6	1821.6	717915.9	69002.6	15748.9	6830671.0	7437.9	98862.5	15030.1	2774372.3

			一価不飽和脂肪酸																			
20:0 アラキジン酸 (mg)	22:0 ベヘン酸 (mg)	24:0 リグノセリン酸 (mg)	10:1 デセン酸 (mg)	14:1 ミリストレイン酸 (mg)	15:1 ペンタデセン酸 (mg)	16:1 パルミトレイン酸 (mg)	17:1 ヘプタデセン酸 (mg)	18:1 計 (mg)	18:1 *n*-9 オレイン酸 (mg)	18:1 *n*-7 シス-バクセン酸 (mg)	20:1 イコセン酸 (mg)	22:1 ドコセン酸 (mg)	24:1 テトラコセン酸 (mg)	16:2 ヘキサデカジエン酸 (mg)	16:3 ヘキサデカトリエン酸 (mg)	16:4 ヘキサデカテトラエン酸 (mg)	18:2 n-6 リノール酸 (mg)	18:3 *n*-3 α-リノレン酸 (mg)	18:3 *n*-6 γ-リノレン酸 (mg)	18:4 *n*-3 オクタデカテトラエン酸 (mg)	20:2 *n*-6 イコサジエン酸 (mg)	20:3 *n*-3 イコサトリエン酸 (mg)
104178.6	48900.5	22134.8	11607.1	100667.6	22.7	815294.3	84945.6	14902759.2	6095568.0	380083.3	297710.6	96858.4	19542.9	2559.9	2458.3	2736.7	6520667.0	1043089.8	1163.8	25402.8	50035.0	440.5
107938.6	48624.4	23033.7	11793.7	89590.1	46.7	824718.6	82308.1	15253849.5	6518604.2	424625.3	372749.0	168570.5	21191.8	3673.8	2944.0	3903.2	6679119.5	1056757.5	1446.0	36839.6	57013.6	857.9
116317.1	55387.3	24924.4	9715.2	92947.9	41.7	845211.5	86642.2	16060134.9	6822543.0	432336.7	387368.4	171241.0	25114.8	4545.3	3809.5	4852.0	7268736.4	1174126.4	2127.5	45103.6	56217.8	808.1
108962.4	50625.8	22639.9	12218.2	84254.0	69.6	793390.6	78668.2	14888384.0	6558627.9	419773.0	417363.7	230974.8	25276.6	4494.8	3525.4	4860.6	6765508.0	1078092.7	2280.1	57630.6	54897.6	1131.2
101051.8	46134.3	20716.6	12975.2	96569.1	36.6	781770.0	83391.9	14392801.9	6144138.0	396579.4	336093.9	143419.3	19889.9	3003.5	2588.2	3098.1	6222174.5	987848.2	1620.9	38059.4	53080.6	654.0
102629.1	46762.5	20656.9	10398.1	82255.8	34.7	812606.8	79818.9	14711166.8	6589971.6	423766.6	350154.2	149760.8	22303.4	3829.1	3350.4	3929.8	6420443.3	1014541.2	1850.7	40573.6	56623.3	653.5
102123.8	49013.9	21626.7	11098.6	105741.5	28.6	803795.0	86814.9	14622309.2	5675711.7	360246.7	319075.4	124026.0	20024.6	2671.9	2342.4	2725.7	6508777.0	1056814.4	1296.0	30707.2	47297.6	535.7
98894.5	47154.9	20814.6	10229.3	71059.9	24.9	666974.2	68215.3	13417919.1	5873262.7	377967.7	300799.8	115259.4	18986.1	2443.9	2128.0	2458.6	6251243.1	1014266.9	1203.9	28656.3	48299.0	461.9
102402.9	48034.7	21431.6	11390.8	82699.3	40.3	735957.8	75782.9	14003173.7	6082845.4	388683.2	350117.0	162603.3	23482.2	3669.8	2981.0	3603.9	6272494.5	1030587.6	1794.1	42099.4	49726.8	737.9
94873.7	44227.4	20090.5	11918.4	87361.4	25.5	705907.7	74103.6	13151476.6	5433218.4	348304.7	293197.7	116334.5	18924.1	2582.1	2231.8	2470.3	5905382.1	934832.5	1321.4	30080.4	45641.4	494.6
98158.7	48536.5	21709.0	9927.9	67267.0	23.6	599070.8	62372.3	12672454.1	5145452.9	326665.4	286774.2	112362.0	19716.1	2567.3	2197.0	2451.2	6178169.3	1030085.0	1368.5	29423.6	41740.8	495.3
114678.3	53648.3	24378.7	12922.6	100044.4	21.2	842775.6	87962.3	15992464.7	6609035.9	424222.6	331271.5	109702.8	21594.1	2612.8	2410.1	2490.0	7083723.0	1143938.9	1292.8	28102.1	55961.1	416.7
109092.0	51326.2	22966.7	14227.4	109937.8	23.6	842382.0	89630.2	15350614.9	6107867.2	388916.8	325149.4	117148.7	21788.4	2783.3	2661.9	2711.9	6719662.3	1090240.3	1403.1	31538.1	51792.0	473.3
96839.3	43540.9	19867.6	12609.5	108199.3	19.5	819097.0	88518.5	14238242.6	5859965.3	373605.0	292804.6	99766.6	18795.8	2357.9	2219.3	2265.7	5875669.5	932180.5	1170.8	26220.6	49840.0	382.8
97129.2	43623.2	19994.9	13457.3	102170.4	24.7	791012.9	84875.9	13993964.0	5849072.1	372808.2	302620.6	113449.7	19985.0	2775.0	2541.4	2691.2	5831215.8	928591.1	1393.0	30520.4	49911.7	467.8
110360.5	50209.2	22133.2	13061.8	87795.6	31.6	822575.1	83241.0	15329730.7	6984485.4	449499.8	359361.4	147713.2	23375.6	3520.5	3074.9	3672.6	6776689.3	1067900.4	1572.5	37794.0	58254.7	604.5
108521.3	50874.5	23056.1	12479.0	93165.5	15.1	806600.9	85312.2	15088579.8	6597720.3	404461.4	317007.5	105958.3	25505.4	2618.1	2578.7	2435.5	6739526.4	1075030.3	1240.6	31115.8	51543.0	342.4
97577.5	42887.1	19413.2	12041.2	107761.1	17.6	842751.4	92318.3	14537205.5	6412866.5	398719.5	295615.7	94798.7	21068.8	2540.3	2423.7	2532.8	5868595.7	918707.6	1199.4	27634.7	52043.7	379.9
90709.0	41846.6	19587.0	10035.5	99132.8	14.6	757566.3	81966.5	13277493.7	5371851.0	333453.2	264359.8	83367.9	18666.6	2168.0	2168.8	2146.1	5656797.1	891757.1	1153.0	25231.6	43838.3	329.9
94252.7	43304.4	19370.2	9963.1	79227.2	19.9	689776.6	72657.4	13053492.9	5625894.0	363273.5	291078.3	108959.4	19100.1	2563.7	2253.4	2317.8	5730989.3	922767.4	1325.0	28515.7	47692.7	398.9
105282.7	50545.2	22551.5	13294.7	78433.5	30.4	691126.2	70252.8	14043993.3	6009209.9	378590.7	321842.3	131082.0	21477.0	2907.0	2526.8	2957.9	6557593.6	1075064.7	1487.8	34044.1	48556.1	589.2
102946.1	49927.4	22309.2	11066.5	98644.7	19.9	759165.1	82061.5	14350842.9	5859874.8	361173.8	291448.7	97841.4	20074.4	2273.1	2143.6	2129.7	6505706.8	1057799.6	1120.9	25252.0	45089.4	388.5
105902.7	48375.0	22410.9	11771.4	90421.0	20.6	804317.8	82541.8	14797915.2	6316438.4	408899.9	326815.6	120145.9	21744.7	2870.1	2414.8	2403.3	6566862.4	1057501.3	1602.1	33133.0	53962.5	393.2
100951.6	46325.0	21138.2	12440.2	95380.7	17.9	782020.4	81413.4	14474126.1	6368769.2	391162.9	284207.4	88192.7	18095.4	2050.5	2029.6	2037.6	6286511.0	980764.2	988.4	22652.8	50213.6	358.4
98091.2	45790.5	20488.2	11685.8	111800.5	26.6	849997.1	90534.3	14482518.1	5736712.1	358795.3	318561.5	126402.2	22355.0	3184.7	2732.7	3173.3	6163723.8	992055.3	1467.8	34382.4	47745.2	498.3
106009.8	48828.8	22581.8	12205.9	124633.8	33.3	944693.6	100137.1	15747850.8	6075745.2	375043.2	345665.7	141073.0	24701.4	3872.6	3572.4	3963.7	6568372.1	1034163.1	1611.0	36694.4	51097.7	617.5
99402.5	47174.8	21343.7	12163.0	117868.5	19.4	848520.4	90621.1	14590351.9	5500801.6	334765.3	284576.1	93439.3	19716.6	2664.7	2677.1	2690.9	6245877.6	999860.2	1229.2	25469.1	44378.8	397.1
113920.3	54674.9	25099.1	11996.0	127846.5	19.2	935902.2	100550.4	16608284.8	6304422.1	387083.3	322402.5	101050.3	22122.2	2787.9	2694.4	2823.3	7158573.8	1152325.7	1255.0	26451.1	50814.1	380.8
92458.5	41830.4	19665.8	13184.5	114543.9	18.4	828129.5	88168.6	13965079.0	5588018.6	344724.5	271393.1	87179.2	17293.7	2348.4	2296.9	2336.6	5706409.1	872332.8	1002.7	22225.6	46737.8	355.9
105625.0	50483.3	22762.8	12737.9	131269.7	24.0	905753.5	99509.3	15559490.9	5785570.1	353095.8	312792.3	110192.1	21326.6	3005.1	2886.4	3094.3	6576219.6	1065498.7	1268.6	27982.9	46754.6	473.6
103739.2	49135.0	22631.0	10651.6	119321.9	21.8	894288.2	94674.1	15368228.2	5949354.4	364940.1	306972.4	103876.8	21786.6	2950.2	2680.5	2884.4	6528776.8	1045318.9	1289.6	27827.4	47523.4	412.6
117823.6	56604.3	25682.1	11569.7	110484.9	24.7	929995.9	93682.7	16892384.8	6901239.5	414090.3	337293.1	112149.1	24795.3	3505.7	3433.4	3743.1	7437429.9	1191726.7	1419.4	30794.0	53092.7	504.2
106418.8	49838.1	22525.2	12380.5	101732.1	16.0	847138.3	85767.5	15156394.7	6003814.0	377021.1	295128.2	86380.8	21312.3	2987.9	2986.0	2953.1	6589385.6	1059884.4	1326.5	25507.3	50955.6	344.0
90698.4	41557.6	18573.7	10931.5	97571.7	21.8	774691.8	80100.3	13424810.9	5633589.8	349119.9	273244.3	94946.7	17638.3	2527.2	2386.1	2571.1	5639337.3	879071.9	1006.3	23329.8	45835.0	413.5
107422.2	50959.6	22819.3	12061.8	121610.7	20.1	905884.9	96170.5	15827215.6	6246916.9	383780.5	299614.3	89118.0	19809.5	2747.1	2690.8	2824.3	6760006.5	1067103.4	1120.7	23522.6	50801.3	390.2
111842.8	53317.7	23845.6	11468.5	114610.7	16.4	906743.0	95072.8	16215080.6	6318765.0	388163.3	307103.7	87094.1	21732.6	2833.3	2686.9	2777.5	7076028.4	1125641.5	1166.7	23797.1	52292.7	350.2
101518.6	50749.1	22476.0	10902.2	103847.5	19.3	756866.9	81577.4	14128857.0	5239344.2	320697.1	284335.8	94895.0	20566.2	2321.3	2071.1	2261.5	6461428.5	1071400.9	1167.1	27028.3	40765.2	384.4
125342.5	63556.0	28196.1	11852.4	118447.5	25.1	909630.2	94735.7	17286687.7	6062787.6	371907.9	338385.6	108419.2	24236.7	2678.9	2561.1	2771.8	8116440.8	1330112.3	1246.5	28895.0	49729.8	471.2
112853.2	55981.0	24740.4	9304.4	112716.4	26.8	881765.2	92895.5	16083959.9	5901841.3	367578.6	325946.9	111456.7	22909.0	2966.2	2609.8	3005.8	7283068.6	1198003.1	1195.6	27943.8	48586.9	492.6
100584.6	46944.6	21403.9	8658.3	99866.8	18.1	856771.2	87008.9	14824860.7	5934710.8	370720.7	300654.9	99604.5	22247.1	2648.4	2525.1	2529.2	6402775.8	1012443.0	1247.7	28468.4	49191.9	376.1
97825.1	44995.7	20148.0	10504.9	104560.9	15.6	865942.2	87868.2	14730594.7	5920495.6	367805.8	279541.3	78619.3	19496.9	2503.0	2350.8	2505.4	6121547.1	980574.3	1076.4	22437.7	49002.6	322.6
113007.5	54975.2	24612.4	10871.2	119246.1	18.9	915390.4	96294.1	16242044.3	5977237.0	369400.6	312717.3	93153.7	22626.6	3195.3	3034.2	3188.3	7164380.4	1178126.4	1345.5	25934.0	49359.8	399.6
103164.8	50957.5	22983.8	10707.9	96760.5	10.2	765095.7	80112.3	14218645.4	5333116.9	320259.5	267045.3	72416.5	21796.8	2923.1	2839.2	2711.5	6500813.5	1079955.1	1288.1	23155.5	42371.1	246.2
103417.6	50100.1	22539.9	9493.4	103544.0	12.3	831509.2	84920.5	14979182.9	5612210.0	345560.8	274558.2	69251.4	18836.3	2323.9	2191.0	2389.7	6580766.0	1078563.8	1007.5	19153.6	46349.0	249.7
109730.7	53769.4	24355.9	10377.2	113000.7	18.9	895007.5	91267.1	15748089.8	5665353.3	350053.7	304787.3	93739.6	22867.0	3152.3	2965.2	3182.8	7022726.2	1144320.8	1348.5	26275.3	47755.3	351.4
103162.0	49804.9	22123.7	10304.9	104618.4	10.4	837765.9	86177.7	14878907.9	5628721.9	351398.9	275357.2	70277.7	20527.1	2613.0	2503.1	2442.0	6516197.1	1070918.0	1100.5	20098.7	46618.3	224.1
118452.2	59631.6	26245.6	9072.9	93631.8	12.4	827679.1	82503.4	16141604.3	5945474.5	372023.5	298820.8	73834.6	22233.2	2491.9	2364.3	2366.3	7653271.2	1284786.0	1086.0	20317.2	48674.8	264.2
100020.2	48872.8	21962.0	8398.5	90103.7	17.7	721576.5	77400.6	13977140.8	4935230.3	317036.5	275439.5	84354.2	16702.5	1850.2	1686.5	1673.4	6494593.4	1042516.4	877.2	18878.1	44548.4	353.7
103983.9	45729.3	20989.3	14436.2	116104.0	24.7	901057.7	95976.1	15478415.4	6436220.8	417284.8	327434.7	117135.5	20122.2	2714.1	2505.1	2616.3	6328385.8	986679.9	1316.0	29871.7	56346.4	465.8
113495.4	51167.5	23176.5	13518.0	105806.0	19.5	888389.7	93760.5	16176938.5	7064582.7	450358.5	333176.0	108317.4	21845.6	2779.3	2640.9	2636.8	6926509.7	1094196.6	1344.4	28847.3	59032.9	430.2
103948.7	49681.3	22007.5	11823.0	81418.0	21.5	717831.9	74019.9	14091464.5	5976516.9	382225.1	296230.1	101718.8	19454.3	2409.3	2176.9	2284.3	6566788.7	1065827.1	1229.6	27015.7	49856.1	424.3
98748.8	44573.7	20486.0	10625.7	117601.6	16.9	912584.6	95910.3	15198506.1	6287309.5	386457.3	291954.4	88507.8	19153.0	2537.7	2546.3	2537.0	6209042.6	959628.8	1116.8	24077.5	51160.9	344.1
102161.9	49761.7	22219.0	9000.4	104536.4	13.2	831502.5	85953.2	14766172.0	5635594.6	339465.8	272304.0	73137.5	20047.1	2600.6	2622.4	2647.5	6502497.3	1058664.2	1160.5	21727.0	44998.8	277.4

多価不飽和脂肪酸										未同定物質 (mg)	利用可能炭水化物									糖アルコール	
20:3 n-6 イコサトリエン酸 (mg)	20:4 n-3 イコサテトラエン酸 (mg)	20:4 n-6 アラキドン酸 (mg)	20:5 n-3 イコサペンタエン酸 (mg)	21:5 n-3 ヘンイコサペンタエン酸(mg)	22:2 ドコサジエン酸 (mg)	22:4 n-6 ドコサテトラエン酸 (mg)	22:5 n-3 ドコサペンタエン酸 (mg)	22:5 n-6 ドコサペンタエン酸 (mg)	22:6 n-3 ドコサヘキサエン酸 (mg)		でん粉 (g)	ぶどう糖 (g)	果糖 (g)	ガラクトース (g)	しょ糖 (g)	麦芽糖 (g)	乳糖 (g)	トレハロース (g)	利用可能炭水化物計 (g)	ソルビトール (g)	マンニトール (g)
22916.1	9193.9	103920.8	77910.6	2417.9	167.2	14092.7	30861.5	16562.5	149133.3	180492.9	76740.1	4245.0	4378.9	34.9	10373.7	685.3	3566.9	56.9	100093.8	130.5	193.6
23406.1	12485.1	104926.7	97010.3	3902.2	285.1	15865.1	35451.3	16827.4	179893.8	219604.4	92057.5	4272.7	4189.4	38.4	8372.5	663.2	3421.4	56.9	113087.8	96.1	195.5
23935.3	14598.9	119105.0	128261.0	4708.6	335.9	16523.3	43027.1	20563.7	227900.5	232725.2	85605.7	4560.1	5206.8	39.9	10954.4	620.7	2896.5	56.5	109974.9	182.6	250.3
23622.4	16414.7	114243.4	126128.9	4855.4	381.9	15753.7	42598.6	19095.7	231177.8	250504.1	90156.0	5032.5	6118.9	41.8	10779.5	744.2	3753.0	65.6	116726.3	261.7	297.4
23008.3	11701.3	106883.1	92319.3	3344.6	255.9	15091.4	34385.5	17514.9	176117.5	207492.0	71711.9	4429.3	4634.7	35.0	11010.0	678.7	3938.1	57.2	96508.0	180.9	254.7
23967.1	13332.2	118390.6	114656.8	4002.6	295.5	16466.4	40600.9	19795.1	211459.4	213538.7	67485.4	4682.9	4983.4	39.6	10753.7	637.0	3278.2	55.9	91945.7	175.2	262.6
22075.4	10626.2	103075.1	84954.8	2949.5	216.2	13638.2	31561.5	17606.7	167221.3	196508.7	77345.8	4121.9	4362.5	40.3	10577.4	677.2	3481.4	52.3	100668.4	157.1	234.2
20291.3	10171.0	98601.1	78884.4	2720.0	234.8	14094.4	30948.4	16818.1	159796.4	183370.8	72444.0	4158.4	4555.3	27.6	10911.0	592.2	3180.1	48.0	95916.4	242.1	192.2
21787.9	13567.6	104305.3	111292.8	3897.3	301.8	14339.2	39297.0	17477.4	209792.1	213942.1	67059.3	4296.3	4743.5	41.0	10656.6	650.1	3764.7	55.5	91292.3	190.1	213.1
20908.9	10191.3	97377.7	83305.1	2849.3	180.5	13357.6	31613.4	16323.8	163832.8	182809.7	80531.7	4221.3	4455.6	32.8	9369.5	659.0	3847.8	53.3	103178.7	165.1	193.3
17994.2	9741.3	88169.3	81228.4	2829.1	171.8	12180.8	29944.6	14936.4	157046.0	174065.4	79044.7	4260.6	4531.8	33.0	12824.5	590.8	2928.9	54.4	104282.5	165.1	225.0
24099.1	10167.7	106526.8	84821.1	2849.4	173.6	15661.7	33928.3	16542.0	167099.9	192755.7	78197.8	4483.2	4655.8	37.6	9059.2	711.0	3920.5	58.6	101137.0	130.9	198.1
24036.1	11006.4	105629.9	93235.5	3064.4	189.6	14591.8	35451.2	16596.0	179825.2	196636.4	72683.9	4676.0	4800.5	33.4	8843.9	712.7	4489.0	57.2	96308.4	162.7	194.9
22517.9	9404.4	95879.8	79086.2	2593.0	156.1	13740.7	31371.5	14560.7	155549.4	179862.1	67064.4	4319.9	4465.3	29.3	9010.7	666.9	3615.0	55.1	89243.1	124.1	191.8
22495.3	10430.9	98755.7	89312.8	2950.9	173.5	13846.9	33936.3	15164.0	170441.4	184876.4	66974.4	4451.3	4781.9	31.7	10352.6	669.9	3909.2	58.2	91241.0	155.9	221.4
24539.0	14040.5	113952.8	114524.3	3686.4	302.9	16460.6	42437.5	18031.9	215768.6	219313.6	81852.4	4842.6	4995.9	38.0	10636.3	746.1	4351.3	60.5	107536.0	228.0	257.4
23681.9	12725.7	119592.1	112341.7	2759.3	182.3	15353.2	43745.2	20121.0	223211.4	217536.1	86906.1	4547.4	4729.8	42.3	9910.1	744.0	3713.3	59.0	110665.5	190.6	255.7
23474.2	10667.3	107396.4	95085.9	2549.2	148.1	14690.6	37407.7	16785.1	180618.9	197134.8	70608.6	3954.5	4069.5	37.3	8932.1	774.9	3585.9	65.0	92035.7	124.0	229.7
20850.9	9122.8	96861.6	81255.2	2221.6	133.3	12651.0	31330.1	15522.7	155180.6	170541.0	83393.3	3845.7	3962.1	28.4	8676.3	632.5	3039.6	52.8	103628.6	131.1	195.5
19812.4	9946.8	88924.5	83091.8	2935.4	189.0	13337.7	31918.9	14132.1	165287.9	176124.3	67127.1	3993.3	3997.8	34.4	8936.8	630.7	3264.0	53.6	88040.5	100.8	176.5
21538.9	11425.3	103444.8	92808.8	3124.1	227.4	14071.1	34263.9	17086.1	176528.5	202084.4	71436.7	4157.5	4744.6	35.7	12926.6	738.4	4351.6	62.0	98469.5	171.3	245.7
21427.3	9132.9	100680.6	77455.7	2482.8	153.0	13318.8	30559.5	17206.7	155615.2	187731.5	80371.4	4495.5	4648.4	34.7	10520.3	704.6	3391.0	64.9	104241.5	113.2	215.8
23094.8	11055.2	107000.5	95670.8	3395.7	162.1	15365.6	36440.5	17375.3	195848.8	202777.5	94007.7	4258.8	4098.6	41.4	9725.3	668.1	3423.9	57.3	116291.6	90.6	210.4
22743.0	8381.9	104081.5	68896.8	2212.4	130.1	14437.5	28990.1	16982.3	139582.3	195988.1	82373.3	4485.5	4468.2	37.3	9336.9	791.0	3821.7	63.5	105384.0	103.8	175.8
23614.5	12494.9	109938.8	104514.1	3305.6	202.7	13872.6	38636.9	18680.9	203299.4	202772.2	68886.6	4402.9	4424.0	39.8	10286.7	640.6	4034.4	49.4	92769.4	146.3	205.7
25277.8	13605.3	115339.5	117714.7	3722.0	207.7	14594.2	42922.5	18854.6	217938.9	207426.1	87395.6	4944.4	5046.0	37.9	12582.3	818.3	3654.0	61.7	114551.4	137.4	287.3
22812.3	9327.2	102438.5	83587.2	2607.1	135.3	12786.7	31578.2	16587.5	157453.6	180254.4	74232.2	4666.0	4763.7	35.3	10489.1	785.1	3626.3	67.8	98674.4	119.3	222.4
25100.3	10357.8	111552.7	88141.1	2825.3	158.3	14644.4	34695.0	18366.1	171102.1	197371.8	82705.8	4425.3	4403.5	32.9	9683.9	729.6	3835.6	58.9	105892.7	101.0	228.5
23076.3	8744.8	99465.9	71999.8	2357.4	119.0	13257.7	29674.4	15846.7	140225.0	172193.5	77057.1	4082.4	4185.5	28.2	9705.3	691.1	4025.1	56.9	99842.5	101.4	167.6
24042.2	10701.6	105743.7	90255.4	2953.2	173.3	13429.3	34261.6	17538.5	171796.0	196333.6	73558.7	4929.1	5164.4	36.7	12183.0	724.4	3845.2	58.4	100511.6	146.4	208.9
23947.0	10411.5	108066.9	91560.6	2882.0	144.2	13828.2	34882.0	17974.6	176878.2	183269.7	81116.2	4490.6	4632.4	31.0	12238.6	727.4	3492.0	59.3	106797.0	136.2	193.0
26088.6	11576.3	127522.6	108886.6	3349.6	181.6	16064.9	40015.5	21662.9	197113.4	221461.6	82206.9	4506.4	4814.3	41.9	12033.2	787.9	3822.3	59.1	108282.7	244.0	154.5
23987.7	9708.0	109958.8	90397.5	2689.8	240.7	14503.0	35353.2	17070.5	165659.1	175928.2	71214.9	4179.1	4294.5	34.4	11070.1	695.5	3973.0	61.6	95543.1	155.2	233.7
21629.2	9011.3	96643.8	74284.6	2458.8	147.1	13074.4	29549.0	15680.9	143049.6	171073.8	66617.1	4155.7	4077.0	34.9	9263.5	760.7	3492.2	51.5	88455.5	118.8	150.9
24746.5	9060.1	108355.1	80398.9	2681.9	125.6	14489.6	31810.3	17463.9	148637.7	180807.4	73808.1	4614.1	4813.4	35.6	10609.2	734.9	3862.3	55.5	98543.3	116.8	174.3
24801.6	9529.0	114951.4	86913.2	2617.2	116.8	14981.6	34648.2	18791.8	165891.1	187685.1	74564.7	3902.1	4079.5	34.7	9504.1	683.8	3716.8	55.3	96554.0	126.3	188.5
20747.7	9414.3	94843.4	77629.5	2337.7	137.8	11883.1	30371.0	15734.9	152914.5	171649.2	68768.8	3873.1	4041.1	30.4	9885.4	660.1	3587.0	53.0	90913.5	108.6	153.8
24799.6	10252.8	113686.3	86306.8	2580.8	148.3	14357.4	34396.7	18497.5	165487.9	192252.8	82712.6	4372.1	4548.0	34.7	11645.9	913.2	3968.8	55.5	108251.5	125.1	213.3
23291.6	10236.2	106259.9	86742.8	2828.3	154.1	13974.0	33929.1	17767.1	167977.9	181649.8	71505.7	3961.0	3924.1	29.5	10795.4	680.0	2966.1	50.4	93916.1	93.5	195.5
23391.3	10498.9	113147.4	95047.2	2745.7	133.8	14613.1	36457.3	19298.5	193013.7	191679.4	74328.0	3797.7	3990.9	35.0	9544.8	622.9	2633.2	46.5	95010.4	120.4	186.8
23214.9	8492.1	104966.3	80146.7	2322.0	94.2	13750.3	31271.9	16149.4	149099.6	164116.1	58143.2	4088.7	4173.7	30.6	8641.9	536.4	3065.8	49.3	78747.1	118.7	158.0
24081.4	9919.9	108551.3	90082.8	2857.6	119.2	13913.3	35128.5	17305.6	165959.6	178437.7	75745.3	4313.9	4385.7	36.3	10147.4	634.2	3281.9	54.7	98615.7	115.3	246.3
20848.1	9173.1	99724.3	89038.8	2558.4	86.9	12028.4	33852.6	16152.6	165081.4	164079.5	73613.9	4540.4	4829.6	37.9	13209.6	606.3	3267.4	54.2	100181.3	134.8	236.3
21810.5	7586.0	94962.3	65649.9	2110.4	92.8	12738.0	27617.7	14502.9	125508.1	156883.0	65783.8	3713.7	3799.9	31.3	8922.8	576.4	2972.5	45.7	85851.3	89.3	151.9
23923.6	10027.1	109268.8	93358.6	2825.7	122.4	13629.3	35506.2	17528.7	173403.3	169678.2	75288.5	4320.1	4519.8	35.2	11491.4	616.5	3394.4	59.5	99745.2	143.0	197.9
22653.7	8258.0	101973.3	77389.9	2349.3	77.8	13267.7	31465.0	16246.0	150528.6	156060.0	64584.6	3822.4	3969.8	33.0	10528.9	547.2	3287.7	49.4	86839.0	97.6	152.9
22406.8	7870.8	102633.4	73261.3	2185.0	74.2	13838.3	30039.7	16216.5	141167.2	159713.4	66305.2	3903.3	4047.6	42.0	10863.3	590.3	3076.8	50.5	88895.6	103.1	166.0
19420.9	6778.9	93136.6	52290.7	2046.5	101.7	12972.4	22991.4	16741.3	116065.0	159358.0	69696.4	3131.9	3129.9	27.9	8014.2	492.9	2577.1	53.5	87132.3	66.8	168.1
25342.9	10605.9	109306.2	88069.6	3034.7	182.0	15761.9	34901.5	17007.3	173262.2	201177.9	71649.3	4357.0	4326.0	33.0	9190.4	696.5	4360.5	63.0	94688.0	100.5	207.5
25373.4	10723.3	111828.5	91951.6	2920.8	166.9	16379.9	36742.6	17094.7	179304.0	206799.8	85560.3	4725.5	4887.7	35.2	10950.0	768.1	4272.7	57.5	111269.7	140.4	197.7
21559.9	9243.6	101708.0	77369.9	2550.1	136.1	14377.8	30836.9	16684.1	153860.9	181298.0	76451.2	4154.8	4369.9	34.4	10721.2	581.4	3515.4	49.3	99891.6	162.8	188.1
24594.3	9391.4	111289.7	80765.3	2494.5	131.9	14731.8	32794.6	18269.2	159913.6	187947.4	82081.3	4354.0	4294.6	31.3	10700.5	741.8	3322.3	61.3	105594.4	88.6	204.1
21915.5	8228.2	100720.2	79807.2	2335.5	97.0	12730.3	30258.9	15992.3	146533.5	163266.5	73069.0	4360.6	4504.7	37.1	10926.5	580.7	2830.3	50.5	96378.4	119.1	174.4

Summary

Data:	X	dimension:	53	109											
	Y	dimension:	53	1											
Fit	method:	kernelpls													
Number	of	componen	considerec	52											
TRAINING	%	variance	explained												
	1comps	2comps	3comps	4comps	5comps	6comps	7comps	8comps	9comps	10comps	11comps	12comps	13comps	14comps	15comps
X	14.76	24.37	29.09	35.53	41.56	45.79	49.75	53.25	55.45	57.81	59.84	62.03	63.33	65.52	67.07
sio1	57.33	78.8	92.41	95.47	97.55	98.9	99.36	99.64	99.83	99.9	99.94	99.96	99.97	99.98	99.99
	16comps	17comps	18comps	19comps	20comps	21comps	22comps	23comps	24comps	25comps	26comps	27comps	28comps	29comps	
X	68.44	69.69	71.23	72.47	73.81	74.78	75.94	77.2	78.6	79.86	80.93	81.76	83.06	84.37	
sio1	100	100	100	100	100	100	100	100	100	100	100	100	100	100	
	30comps	31comps	32comps	33comps	34comps	35comps	36comps	37comps	38comps	39comps	40comps	41comps	42comps	43comps	
X	85.29	86.28	87.16	88.38	89.23	90.12	91.15	91.93	92.7	93.31	94.19	94.85	95.34	95.85	
sio1	100	100	100	100	100	100	100	100	100	100	100	100	100	100	
	44comps	45comps	46comps	47comps	48comps	49comps	50comps	51comps	52comps						
X	96.49	96.97	97.45	97.89	98.28	98.84	99.22	99.62	100						
sio1	100	100	100	100	100	100	100	100	100						
>															

Loadings

No.	intake	和名	英名	Factor1	Factor2	Factor3	Factor4	Factor5	Factor6	Factor7	Factor8	Factor9	Factor10	Factor11	Factor12	Factor13	Factor14	Factor15	Factor16	Factor17	Factor18	Factor19	Factor20	Factor21	Factor22	Factor23	Factor24	Factor25	Factor26	Factor27	Factor28	Factor29	Factor30	Factor31	Factor32	Factor33	Factor34	Factor35	Factor36
1	intake11	米	Rice	0.080565	-0.04677	-0.11218	0.078201	0.04217	-0.03951	-0.10459	-0.0831	0.305257	-0.07295	-0.09558	0.083011	-0.06293	0.171115	-0.17034	0.076408	-0.03493	0.121714	0.063082	-0.16601	0.297481	-0.34836	0.111412	-0.15309	0.275381	0.037649	-0.24991	0.131305	-0.0983	0.049641	0.062546	-0.1208	0.09083	0.155271	-0.01852	-0.02916
2	intake12	食 パ ン	White bread	-0.02549	0.004236	0.041325	0.312162	-0.27991	0.057231	0.1443	-0.04591	-0.00907	0.061959	-0.06152	0.10637	-0.07436	-0.0103	-0.12292	0.020388	-0.03634	0.065092	0.039663	-0.01769	0.140232	-0.02954	-0.04206	-0.07318	0.054974	0.067755	-0.00516	-0.07972	0.032944	0.026368	-0.0282	-0.03027	-0.07538	0.130339	-0.00218	-0.02213
3	intake13	生うどん・そば	“Udon”&“Soba	0.002404	0.059204	0.076091	0.194084	-0.22394	-0.00323	0.209852	-0.11193	0.131569	-0.19655	0.119481	-0.01747	0.12927	0.034367	-0.1268	-0.00194	0.091558	-0.04906	0.192707	-0.07286	-0.08073	0.010126	0.194468	-0.01524	-0.08113	-0.21785	0.184035	0.075664	-0.05452	-0.10977	0.173805	-0.03945	0.13137	-0.19694	0.006634	-0.00458
4	intake14	乾うどん・そば	“Udon”&“Soba	0.159382	-0.00541	0.103016	-0.06006	0.013905	-0.01335	0.123225	0.014237	-0.11132	-0.16469	0.340242	-0.21383	-0.02846	0.025043	-0.05074	0.020719	0.023519	0.017706	-0.00681	-0.18721	0.035878	0.154622	0.034611	-0.13048	0.104244	-0.03514	0.057762	-0.08354	0.090884	-0.16579	0.052769	0.003185	-0.14142	-0.06485	0.214416	-0.11709
5	intake15	スパゲッティ	Spaghetti	0.110203	-0.15254	0.103005	0.069759	-0.02395	-0.07793	-0.03973	0.153613	-0.11073	0.136492	0.019496	-0.02355	-0.01919	0.220852	-0.29273	0.036367	-0.11315	0.203825	-0.20578	0.075887	-0.20472	0.112512	0.048282	0.072375	-0.03607	-0.09632	0.044178	-0.00782	0.031815	0.069726	-0.09266	-0.01321	-0.02538	0.096773	-0.11586	0.083947
6	intake16	中 華 め ん	Chinese noodl	0.131063	-0.16914	0.134846	-0.00042	0.008559	-0.19698	0.07813	0.032777	-0.03814	-0.02634	0.019438	0.016024	0.070394	0.05595	0.014348	-0.11762	-0.0635	-0.07645	0.081572	0.033043	0.024844	-0.07808	0.183543	-0.13293	0.066512	0.124276	-0.22307	-0.07108	0.108868	0.140374	-0.12763	0.021092	0.114971	-0.13966	-0.01107	-0.1101
7	intake17	小 麦 粉	Wheat flour	-0.02842	0.08219	0.195356	0.111915	-0.12687	-0.14879	-0.11814	0.07956	0.024706	-0.10082	0.076554	-0.12892	-0.00928	0.069366	0.122267	-0.25922	0.06557	0.142067	-0.18834	0.178713	0.189867	-0.09051	-0.1547	-0.06221	-0.00868	-0.10853	0.243053	-0.14268	-0.05635	0.167764	-0.01005	-0.03974	0.082204	-0.18697	-0.11395	0.246278
8	intake18	も ち	“Mochi”, rice-	0.014011	-0.16001	0.127386	0.100972	-0.15114	0.081917	0.070334	-0.05251	0.114752	-0.03268	-0.13551	0.014071	0.058112	-0.01265	-0.10807	-0.07568	0.154327	0.010473	-0.02202	0.101372	-0.08975	-0.06192	0.035525	-0.10199	0.289254	-0.0878	-0.06075	-0.03696	0.154901	-0.13556	-0.14103	0.250671	-0.29431	-0.03732	-0.02658	0.049368
9	intake19	ま ぐ ろ	Tuna fish	0.06113	-0.1954	0.192606	-0.08821	0.163017	-0.02465	-0.06515	-0.11728	0.123858	-0.04675	-0.12634	0.13663	-0.07205	0.250654	-0.12522	0.062441	-0.06436	-0.04253	0.050391	########	0.102804	-0.0163	0.009045	0.024549	-0.06415	-0.06257	-0.01632	0.032587	-0.02819	0.058144	0.003475	-0.00199	-0.05229	0.026479	-0.08105	0.069896
10	intake110	あ じ	Horse mackere	-0.05524	0.158131	-0.1136	0.032042	-0.15316	0.211289	-0.21663	0.144719	-0.08176	0.071973	-0.15062	0.023427	0.040972	-0.02845	0.083867	-0.15891	0.074713	-0.0324	0.041667	-0.00139	-0.13655	0.130379	-0.11553	-0.01273	-0.0157	0.120248	-0.1277	0.057114	-0.06943	0.046264	0.034427	0.032513	-0.00387	0.010556	0.114548	-0.17463
11	intake111	い わ し	Sardines	0.083662	0.140076	-0.18078	0.142413	-0.13511	0.141195	-0.07825	0.081623	-0.11888	0.107644	-0.12907	0.168596	-0.20093	-0.01543	0.202171	-0.13237	-0.04168	0.08568	-0.0335	-0.01103	0.044712	0.022889	-0.04094	0.158256	-0.24405	0.083045	0.0283	-0.02348	-0.06352	0.113007	0.017487	-0.08005	-0.02288	-0.00062	-0.01431	0.03776
12	intake112	か つ お	Bonito	-0.01155	0.025955	0.000312	-0.01002	0.054599	0.0052	0.010172	-0.2087	0.134458	-0.12166	0.117671	-0.01439	-0.15805	0.325386	-0.16749	-0.04859	0.213224	-0.3378	0.157595	-0.03464	-0.02993	-0.00467	-0.18816	0.211888	-0.27998	0.205152	0.138439	-0.29488	0.187731	-0.02264	-0.06172	0.220274	-0.12761	0.168225	-0.1512	-0.0481
13	intake113	か れ い	Flounder	0.159116	0.061332	-0.21886	0.170797	-0.0455	-0.10648	0.076904	-0.02864	0.003789	0.163022	-0.02109	0.036437	-0.12794	0.011954	0.086258	-0.10877	-0.06571	0.156679	-0.16167	0.064816	0.058261	-0.00577	0.040398	0.04648	-0.06251	0.039876	0.021179	0.004341	0.068193	-0.12181	0.001371	-0.14029	0.13768	-0.10636	0.056644	0.063071
14	intake114	さ け	Salmon	0.18142	-0.10084	0.085522	-0.01991	0.065114	-0.13886	-0.02376	0.075799	0.109542	-0.11432	-0.03558	0.145757	-0.06531	-0.03348	0.075808	-0.01069	-0.02874	-0.04401	0.030851	0.120864	-0.06983	0.004769	0.043967	-0.19328	0.052246	-0.15661	0.158151	0.211427	-0.09385	-0.13032	0.072389	-0.03612	-0.02794	0.05423	-0.00939	-0.17471
15	intake115	さ ば	Mackerel	-0.05335	0.136456	-0.1423	0.113997	-0.08986	0.134555	-0.15175	0.224955	-0.09924	-0.00903	-0.01967	0.292766	-0.1913	0.098622	0.076567	-0.05825	-0.01275	0.017801	-0.03062	0.027897	0.033263	0.041772	-0.09402	0.038453	0.031967	0.11151	-0.11981	0.01208	0.090016	-0.19065	0.014735	0.050301	0.099198	-0.08848	0.103659	-0.05715
16	intake116	さ ん ま	Saury	0.187254	-0.05427	-0.00452	-0.00014	0.059406	-0.18658	0.024176	0.048704	0.042421	-0.25403	0.211842	0.012948	-0.076	0.05559	-0.05915	0.044123	0.098036	-0.13439	-0.1409	0.088393	0.11752	0.048833	-0.1346	-0.02997	0.016278	0.153856	-0.04877	-0.04857	-0.01408	0.052872	0.015056	-0.12104	0.084913	0.028235	-0.07591	0.002148
17	intake117	た い	Sea bream	-0.11312	0.157567	-0.11888	0.152731	-0.08456	0.048582	-0.20825	0.128899	-0.02359	0.032076	0.111113	-0.0888	0.090036	-0.17373	0.143408	-0.00498	0.04053	-0.15417	0.132465	-0.00717	0.058754	0.141605	-0.0705	0.039425	-0.0303	0.004325	-0.04139	-0.04946	0.006396	0.110921	-0.01822	-0.04466	0.137117	0.086872	-0.00902	-0.14945
18	intake118	ぶ り	Yellowtail	-0.01075	0.1137	-0.12582	0.147567	-0.19809	0.200071	0.014644	-0.09819	-0.04737	0.056409	-0.10374	0.009615	-0.13437	0.063426	-0.02724	0.035355	0.060458	-0.18937	0.156066	0.006399	-0.17432	0.19297	0.007564	-0.20206	0.317094	-0.18587	0.131243	-0.14873	0.037237	0.005579	0.031555	0.00579	-0.1364	-0.03878	-0.03577	-0.01983
19	intake119	い か	Cuttlefish	0.175079	0.076919	-0.17161	0.141669	0.021564	-0.00551	-0.02321	-0.18588	0.057562	0.062206	-0.22798	0.136445	-0.00666	0.027483	0.095314	-0.10223	-0.07786	0.09667	0.01408	0.024172	-0.11231	0.067969	0.014047	-0.06127	0.013834	0.085246	-0.01595	-0.03804	0.052051	-0.02355	-0.03024	0.023914	-0.09609	-0.02499	0.003461	0.039395
20	intake120	た こ	Octopus	0.052872	-0.0939	-0.11873	0.226026	0.014247	-0.0876	0.086866	-0.09973	0.298574	-0.27399	0.086373	-0.0783	0.089763	0.017639	-0.04705	0.082464	-0.0082	0.073263	-0.18744	-0.03525	0.061775	-0.08832	0.060569	0.130141	-0.19157	0.064636	0.054202	-0.06343	-0.06944	-0.0618	0.20468	-0.16006	-0.02203	0.059854	0.048411	0.07961
21	intake121	え び	Shrimps & lobs	-0.02391	0.080435	-0.16664	0.259982	-0.21814	0.046874	-0.05477	-0.05197	0.153769	-0.03261	-0.02667	-0.0426	-0.09774	0.034328	0.033003	0.100494	-0.11377	-0.02505	0.104351	-0.0248	0.074518	0.166821	-0.18346	0.060665	-0.04849	-0.16223	0.059166	0.172526	-0.0209	-0.17317	-0.02865	0.158309	-0.05411	0.056245	-0.0492	0.005601
22	intake122	か に	Crabs	0.103699	0.085387	-0.13345	0.162593	-0.06521	0.005625	-0.01316	-0.14493	-0.10196	0.200075	-0.13701	0.135642	-0.13924	-0.04764	0.103901	-0.06445	-0.05577	0.20063	-0.15806	-0.04826	0.137437	-0.16763	0.284699	-0.15771	0.064928	-0.02107	0.091199	-0.15614	0.077833	0.041504	-0.00359	-0.11697	0.076248	-0.00193	0.171255	-0.09454
23	intake123	あ さ り	Short-necked	0.006469	-0.00707	0.102513	-0.17511	-0.01536	0.232936	-0.31957	0.067733	-0.02098	0.136872	-0.03516	-0.00992	-0.07096	0.186934	-0.13756	-0.06246	-0.02801	0.18503	-0.08236	-0.04032	0.075747	-0.10321	0.166467	-0.04157	0.003062	0.078697	-0.0425	0.000957	-0.07632	-0.0636	0.12508	-0.00679	-0.09785	0.041708	0.002633	-0.06852
24	intake124	し じ み	Fresh water cl	0.143231	-0.05319	-0.07402	-0.06936	0.007762	-0.01965	0.149294	0.108395	-0.07602	0.028841	-0.18665	0.262276	-0.08753	0.002217	0.098269	-0.11373	0.034964	0.076645	-0.07152	-0.07692	-0.11575	0.094868	-0.07863	-0.01978	-0.07901	0.205063	0.054139	-0.25036	0.178512	-0.02688	-0.08524	0.097792	-0.01611	-0.15896	0.05951	-0.17239
25	intake125	か き（貝）	Oysters	-0.0132	-0.00863	0.050022	0.086078	-0.20252	0.117316	0.184322	-0.12315	-0.03072	-0.08082	0.029681	-0.11229	0.005492	0.064449	0.058134	-0.12879	0.057769	-0.00773	-0.08208	0.095059	-0.29047	-0.0825	-0.09889	0.321822	-0.09646	0.188298	-0.32701	0.231438	-0.17222	0.122874	0.128781	-0.18151	0.064871	0.186165	-0.30081	0.0383
26	intake126	ほ た て 貝	Scallops	0.161802	0.029547	-0.0464	0.108853	0.171423	-0.11143	-0.06446	-0.00404	0.014831	-0.08112	-0.17399	0.131152	0.076995	-0.14583	0.109414	-0.00277	-0.06637	0.091511	-0.03566	-0.06745	0.177334	-0.04764	0.074981	0.15028	-0.19425	0.063423	-0.06253	0.089656	-0.12956	0.191145	-0.13968	0.027157	-0.1425	0.104088	0.057238	-0.05695
27	intake127	塩 さ け	Salted salmon	0.228667	-0.05709	-0.03235	-0.03876	0.069311	-0.02537	0.055655	-0.13609	0.049298	-0.08467	0.118024	-0.09204	-0.07361	0.050975	-0.01629	0.070202	-0.04203	-0.04365	-0.03221	-0.05842	0.028613	0.042589	-0.08135	0.006041	0.090888	-0.05529	0.071484	-0.14232	-0.03321	-0.00561	0.142291	0.0168	-0.0475	0.065659	0.063969	0.055899
28	intake128	た ら こ	Salted pollock	0.183926	-0.03601	-0.02281	0.019688	0.075823	-0.02757	-0.16032	0.056167	0.005527	0.172982	0.048135	-0.09447	0.011872	-0.28005	0.152257	0.155362	-0.11875	0.0428	0.085753	0.057645	-0.12564	-0.08563	-0.04943	0.21801	-0.02823	-0.02663	-0.09524	0.100796	-0.002	-0.0967	0.041992	0.110462	-0.02114	-0.18703	-0.06706	0.046929
29	intake129	しらす干し	dried young sa	-0.01011	-0.10639	0.191534	0.003894	0.017951	0.078931	-0.07056	-0.26652	0.231457	-0.10096	0.011732	0.182355	-0.33426	0.211138	-0.00539	0.04264	-0.09047	-0.0189	0.126034	-0.11397	0.083231	0.090408	-0.1384	0.030163	-0.15507	0.0491	0.090845	-0.0323	0.119243	-0.00421	-0.04481	-0.1146	-0.03029	0.120327	-0.12394	-0.06201
30	intake130	干 し あ じ	Dried horse ma	0.026555	-0.05427	0.208892	-0.10049	-0.0912	0.141485	-0.26196	0.018453	0.118505	0.120891	-0.20447	0.198599	-0.15548	0.151297	-0.1516	-0.00548	-0.00164	0.006047	-0.06015	-0.13232	0.056029	0.048421	-0.10187	0.08875	-0.23084	0.059545	0.078997	-0.06016	0.010552	0.046474	0.056258	0.00584	-0.00625	0.006718	-0.01464	0.041777
31	intake131	かつお節・削り節	fish flakes, drie	-0.13758	-0.06967	0.029977	0.036842	0.184549	-0.10449	-0.01364	-0.04057	-0.14068	-0.02737	0.100624	0.135078	-0.29638	0.179699	0.077317	-0.13286	0.119823	-0.15425	0.115592	-0.12223	0.10581	-0.21404	0.30193	-0.16599	0.021573	0.004354	-0.09585	0.131651	-0.07797	0.017055	0.007243	0.053185	-0.02496	0.035916	0.046081	-0.00517
32	intake132	牛 肉	Beef	-0.12723	0.080707	-0.11484	0.265308	-0.11715	0.054999	-0.01619	-0.00879	-0.04273	-0.10866	0.020591	-0.09783	0.051865	-0.08758	0.10898	-0.02446	########	-0.08163	0.100939	-0.06833	0.005781	0.091501	0.153947	0.030589	-0.30124	0.182267	0.02758	-0.07759	-0.05453	0.112469	-0.03054	-0.09313	0.03188	0.095947	-0.06605	0.029076
33	intake133	豚 肉	Pork	0.142676	-0.15496	0.069913	0.059566	0.03125	-0.06789	-0.02523	0.092239	-0.09549	0.095169	-0.08069	-0.0944	0.020542	0.270588	-0.1407	0.102076	-0.21718	0.080703	-0.03032	-0.09656	0.128444	-0.01622	-0.22969	0.221179	0.061972	-0.02937	-0.07246	-0.00895	-0.00372	0.016095	-0.00781	0.035992	0.057539	-0.07229	-0.02788	-0.10951
34	intake134	鶏 肉	Chicken	-0.06391	0.078164	-0.14077	0.257726	-0.11684	0.061616	-0.16326	0.185854	-0.16106	0.085372	0.110198	-0.01765	-0.04771	0.048596	-0.09781	0.172043	0.021142	0.09288	-0.04677	-0.11442	0.04874	0.098207	-0.18442	0.241871	-0.11794	0.066446	-0.00366	-0.14604	0.043607	-0.04085	-0.00831	-0.01135	0.125477	-0.05356	-0.06573	0.04958
35	intake135	合いびき肉	Mixed ground r	-0.14901	0.110542	-0.0936	0.211731	-0.17603	0.106891	0.015591	0.071867	-0.08414	0.108081	-0.05978	0.055046	-0.09056	0.086432	-0.1086	0.068012	0.079573	-0.02599	0.114585	-0.07667	-0.01168	0.049388	0.008867	-0.11	0.102855	-0.04413	0.07602	-0.11629	0.041823	0.126776	-0.08343	-0.04568	0.003223	-0.00511	-0.02548	0.008412
36	intake136	ハ ム	Ham	0.080631	-0.15383	0.069598	0.005236	-0.17667	0.014856	-0.03542	-0.08823	0.15541	-0.04089	-0.1446	0.081549	0.14819	-0.12316	0.043599	0.085924	-0.076	-0.1207	0.212067	-0.1138	0.002054	-0.0305	0.33073	-0.136	0.22563	-0.04428	-0.17401	0.111757	-0.1187	0.007627	-0.0252	-0.02023	0.166156	-0.16879	0.001615	0.007202
37	intake137	ソーセージ	Sausages	0.13264	0.04527	-0.14245	0.117669	-0.03972	-0.04874	0.040071	0.039712	0.030372	0.05073	-0.21066	0.004464	-0.07873	0.286136	-0.12811	0.140711	-0.12529	-0.04436	0.002205	0.0872	-0.31327	0.155723	0.029361	-0.02069	0.009454	-0.1067	0.15881	0.091168	-0.26476	0.221492	-0.22659	0.108562	0.015221	-0.00044	-0.04556	0.065349
38	intake138	ベーコン	Bacon	0.092357	-0.11461	-0.05707	0.018377	0.174405	-0.11956	-0.06193	0.131636	-0.00502	0.168798	-0.06196	-0.2671	-0.02419	0.197908	0.016058	-0.17424	0.076132	0.016946	-0.019	0.009574	-0.04588	0.168604	0.049046	-0.17249	0.000641	0.196671	-0.32168	0.296049	-0.11603	0.011439	0.042777	0.000977	0.030617	-0.02704	0.059201	-0.00476
39	intake139	牛 乳	Fresh milk	0.101169	-0.09485	0.15264	0.1036	-0.23	0.086614	0.167759	0.058625	-0.11112	-0.0623	0.03406	-0.08469	0.094962	0.130018	-0.02013	-0.06913	0.083624	0.072545	-0.16769	0.057575	0.021492	0.089937	-0.12652	0.054416	0.116811	-0.13113	0.158525	-0.18509	-0.09653	0.03951	0.120126	0.087262	0.006548	-0.05043	-0.00155	-0.0086
40	intake140	粉ミルク	Powdered milk	-0.05819	-0.07039	0.076731	-0.05984	0.133395	-0.12186	-0.09479	0.199818	-0.10742	-0.09934	0.087979	0.173865	-0.28755	0.245542	-0.11715	0.143033	-0.19823	0.082127	0.055536	0.073562	-0.25434	0.090975	0.292565	-0.10881	-0.10627	0.003482	0.159866	-0.02418	-0.13161	-0.11552	0.215464	0.057626	-0.01643	0.109265	-0.09837	0.007739
41	intake141	バ タ ー	Butter	-0.03459	-0.2016	0.103085	0.160658	-0.07853	0.032419	-0.11631	-0.00889	0.132239	-0.10546	-0.00715	0.028778	0.064115	0.017368	0.015197	-0.13416	-0.0856	0.158414	-0.15705	0.122883	-0.16729	0.29266	-0.27959	0.055769	-0.01049	0.086732	-0.00963	-0.11254	0.041677	0.018223	-0.08093	-0.0938	0.134089	0.108206	-0.03126	-0.01241
42	intake142	チ ー ズ	Cheese	0.069695	-0.25689	0.173307	0.053577	-0.02632	-0.06605	-0.02333	0.088397	-0.06883	0.136784	-0.07018	-0.04967	0.058221	0.08636	-0.05067	-0.01957	0.131527	0.05324	-0.06226	0.039534	-0.07572	0.073491	0.029521	-0.10887	0.150778	-0.08863	-0.07993	0.105913	-0.01847	0.04148	-0.08608	0.016672	-0.01577	0.142579	0.037303	0.044874
43	intake143	卵	Eggs	0.083802	0.123406	-0.20419	0.181596	-0.11544	-0.02908	0.094141	-0.04528	-0.04258	0.041654	0.108504	-0.00338	-0.20246	0.279489	-0.08738	-0.03097	-0.02484	-0.14985	0.164711	-0.08134	0.092229	-0.11282	0.075302	-0.1099	0.001261	0.021605	-0.05226	0.031486	-0.01527	0.128269	-0.13107	0.135553	-0.09325	-0.06363	-0.15174	0.151027
44	intake144	キャベツ	Cabbage	0.081939	-0.03858	0.054165	0.075507	-0.15852	0.133182	-0.19657	0.239789	-0.21257	-0.08229	0.007588	0.125304	-0.08959	-0.02996	-0.06514	0.100207	-0.10795	-0.08655	0.121429	-0.06099	-0.05295	0.028959	-0.18479	0.207691	-0.03406	-0.06684	-0.06633	0.140667	0.02031	-0.11561	0.020077	-0.05205	0.20321	-0.20974	0.092943	0.014231
45	intake145	ほうれんそう	Spinach	0.185052	-0.16987	0.007823	0.034118	-0.07028	0.011532	0.038771	-0.09857	-0.03078	0.11656	0.032353	-0.00197	-0.06654	0.05803	0.055753	0.038354	0.14117	-0.11671	0.14123	-0.15952	-0.07102	0.101725	0.127249	-0.03969	-0.07224	0.081398	0.050723	-0.09335	0.020544	-0.01651	-0.03487	-0.10519	-0.02287	-0.12027	-0.00503	0.090609
46	intake146	はくさい	Chinese cabba	-0.04438	-0.00172	-0.00583	0.201326	-0.2652	0.035742	-0.07967	0.075953	0.238878	-0.10019	-0.00898	-0.1375	-0.03343	0.131406	0.056361	-0.07138	0.0036	-0.16091	0.029577	-0.02304	0.142955	-0.08934	-0.17179	0.237714	0.053479	0.205405	-0.08721	-0.1039	0.049545	-0.05023	-0.14898	0.180534	-0.01622	-0.0777	0.065131	-0.02342
47	intake147	ね ぎ	Welsh onions	0.187915	-0.2031	0.0402	0.018265	-0.02108	0.082872	0.0404	0.021203	-0.06345	-0.07016	0.005639	0.016546	-0.00722	0.033072	-0.1313	0.167934	-0.0737	0.017586	-0.00998	0.076943	0.051445	-0.09764	0.052246	0.141153	-0.11999	0.027146	-0.0538	-0.01119	0.041695	0.003962	-0.04218	-0.00331	0.026737	-0.01152	-0.01758	0.065277
48	intake148	レ タ ス	Lettuce	0.046433	-0.16229	0.225912	-0.01264	-0.13828	0.041329	-0.13769	0.127783	-0.04419	0.211394	-0.11084	0.119423	-0.16108	-0.03105	0.070228	0.018724	-0.17651	-0.03847	0.107724	-0.0536	0.063568	-0.15256	0.149186	0.084913	-0.11892	-0.14363	0.138973	0.048227	-0.07847	-0.07	0.061314	0.013353	-0.10937	0.112552	0.010042	-0.15559
49	intake149	ブロッコリー	Broccoli	0.135893	-0.20073	0.16474	0.002463	-0.09784	0.067831	0.014975	0.110212	-0.14565	0.134175	0.00042	0.044483	-0.10496	-0.05324	0.086746	-0.08489	-0.07673	-0.01361	0.101466	0.068792	-0.06253	0.016526	0.039903	0.052909	-0.04281	0.117348	-0.07283	########	0.039379	-0.0806	0.108121	-0.03242	-0.02454	0.119227	-0.12863	0.061213
50	intake150	も や し	Bean sprouts	0.231466	-0.03274	-0.07053	0.023302	0.00144	-0.00526	0.062085	3.00E-05	-0.04827	0.185861	0.009197	-0.21221	0.112222	0.083068	-0.0974	0.002004	-0.02411	0.000339	0.053804	0.066402	0.048458	0.01008	0.043495	-0.0179	0.053829	-0.08263	0.016935	-0.03249	0.091563	0.061302	-0.11947	-0.07667	0.118163	-0.01607	0.050895	-0.00279
51	intake151	さつまいも	Sweet potatoe	0.021035	-0.04425	-0.01457	-0.02026	-0.08617	0.06721	-0.08402	0.210213	0.080948	-0.36577	0.228108	-0.16024	-0.02292	0.127322	-0.01494	-0.12003	0.199343	-0.028	0.061454	-0.08125	-0.11435	0.107348	0.051979	0.05844	-0.0508	-0.1887	0.043691	-0.21504	0.309834	0.055116	-0.04834	0.01734	-0.06637	-0.08426	0.159295	-0.1459
52	intake152	じゃがいも	White potatoes	-0.01901	-0.12966	-0.06165	0.227122	-0.15757	0.103855	-0.22526	0.112702	0.082749	-0.15746	-0.00375	0.051148	0.059076	0.021336	-0.04546	-0.08888	0.003575	0.298347	-0.03149	-0.15344	0.023525	0.02394	-0.03366	-0.05223	0.087168	0.083153	-0.00266	-0.02194	-0.00713	-0.08588	0.166922	-0.13098	-0.04874	0.015705	0.034764	-0.08155
53	intake153	さ と い も	Taros	-0.01253	-0.01802	0.057786	-0.11738	-0.13312	0.267373	-0.15774	0.09065	0.049189	0.061251	-0.01062	-0.17279	0.226699	-0.00447	-0.09149	0.134352	-0.08944	0.079822	-0.11849	0.010745	0.020797	-0.33628	0.295509	-0.21055	0.066777	0.131739	0.015088	-0.12768	0.110258	0.029807	0.077103	-0.10166	0.060605	-0.08187	-0.01258	0.089342
54	intake154	だ い こ ん	Radishes	0.157652	-0.20835	0.075398	0.043703	0.006469	-0.04807	-0.13481	0.110291	-0.01297	-0.11163	0.095895	-0.07842	-0.06497	0.034857	-0.0558	0.077904	0.007364	-0.04747	-0.01074	-0.0491	-0.04086	0.119365	-0.06498	0.027862	-0.10761	0.12031	0.06128	-0.08066	0.144649	-0.19675	0.039974	-0.11866	0.040241	0.029739	-0.03999	0.144959
55	intake155	に ん じ ん	Carrots	0.086307	-0.16572	0.086471	-0.00969	0.014407	-0.0165	0.02166	0.153421	-0.2941	0.09843	0.032774	0.099204	-0.24855	0.280005	-0.03453	-0.08515	0.033988	-0.06419	0.13132	-0.03329	0.062906	-0.15057	0.103979	-0.06685	-0.07214	0.193118	-0.10107	0.035236	-0.05772	0.13956	0.026843	-0.25453	0.113685	-0.10584	0.147832	-0.1339
56	intake156	ご ぼ う	Burdocks	0.139482	0.053882	-0.07759	-0.05345	0.0689	0.031863	-0.15108	0.21913	-0.0501	-0.08822	0.049338	-0.14821	0.267891	-0.05518	-0.04632	0.031436	0.016365	-0.17605	0.041398	-0.08999	0.162704	-0.00452	-0.02374	-0.06034	-0.19243	0.422155	-0.25294	0.11738	-0.08315	0.064922	-0.11317	0.056162	0.100571	-0.07892	-0.01487	0.052361
57	intake157	た ま ね ぎ	Onions	0.045429	-0.11451	-0.14333	0.257905	-0.06453	-0.02924	-0.16159	0.230021	-0.0687	-0.1064	0.085437	0.067135	-0.02085	-0.08428	-0.07307	0.055064	0.033572	0.071184	0.077999	-0.11974	0.025582	0.080116	-0.20432	0.082635	-0.1101	0.148644	-0.15137	0.015088	0.104866	0.110609	0.057336	-0.01088	-0.04435	-0.06208	0.092644	-0.08575
58	intake158	れ ん こ ん	Lotus roots	0.006385	-0.00143	0.114228	0.106862	-0.17262	0.120029	-0.14097	-0.03196	-0.03925	0.015124	0.111173	-0.28136	0.204605	-0.01734	-0.04664	0.156443	-0.0549	-0.03557	0.004047	0.11196	-0.18099	-0.04606	-0.14049	-0.19617	0.081527	0.221518	-0.01842	0.15858	-0.28845	0.079707	-0.05701	0.005624	-0.0178	-0.01496	0.281538	-0.25368
59	intake159	た け の こ	Bamboo shoot	0.069258	-0.10903	0.042987	0.089246	-0.02668	0.153209	-0.07946	-0.21422	0.079626	0.010602	0.103704	-0.22428	0.261384	-0.05074	-0.02733	-0.12186	-0.03575	0.082173	0.070215	-0.08796	0.040241	-0.04758	0.305287	-0.23299	-0.13228	0.062491	0.025555	-0.15053	0.227263	-0.14427	-0.00541	0.109571	0.152886	-0.11632	-0.07854	0.056129
60	intake160	さ や ま め	String beans	0.132596	-0.1836	0.009245	0.036006	-0.00348	0.049965	-0.01486	0.019237	-0.11958	0.095884	0.230083	-0.15334	-0.04772	-0.04976	0.025298	-0.00929	-0.03535	0.057624	0.108458	0.059662	0.054705	-0.35476	-0.04769	0.207577	-0.12896	-0.07525	0.187365	-0.10093	-0.03633	0.085796	-0.04313	0.091988	-0.04587	0.073979	0.064951	-0.00508
61	intake161	か ぼ ち ゃ	Pumpkins	0.092413	-0.11177	0.017797	0.056938	-0.21396	0.12146	-0.07184	0.171132	0.090731	-0.1356	0.163743	-0.20557	-0.12329	0.059681	0.034554	0.018946	0.006177	-0.14893	0.141466	0.065136	-0.03839	0.164023	0.134153	-0.20968	-0.09486	0.109662	-0.00186	-0.00589	-0.00836	0.076338	-0.02649	0.080594	-0.05995	0.059091	0.074638	-0.01982
62	intake162	きゅうり	Cucumbers	0.085167	-0.0313	0.111369	-0.15617	-0.06239	0.101482	-0.28581	0.131906	-0.07821	0.120338	-0.06836	-0.10444	0.317562	-0.10403	0.019419	-0.02763	0.052111	-0.09225	0.106352	0.020384	-0.12501	0.073102	0.05776	0.064559	-0.0356	-0.10149	0.104029	-0.09339	0.248104	-0.17036	-0.03679	-0.18836	0.02266	0.104114	-0.13567	0.169971
63	intake163	な す	Eggplants	0.127037	-0.15885	-0.08403	0.127417	-0.08342	0.150928	-0.11682	-0.02714	-0.02359	0.067644	0.082013	-0.139	0.22263	-0.20528	0.06446	-0.02905	0.020461	-0.05468	0.102246	-0.0179	-0.11919	0.094938	-0.02426	-0.05715	-0.30281	0.188445	0.034743	0.083832	0.007303	0.048653	-0.16301	-0.02256	-0.01353	-0.02645	-0.03061	0.086492
64	intake164	ト マ ト	Tomatoes	0.076966	-0.19854	0.033614	-0.03619	-0.06593	0.166414	-0.17686	0.053599	0.006669	0.053323	-0.00296	0.059727	0.031418	-0.16862	0.215477	0.011453	0.036124	-0.18832	0.048408	0.038968	0.072856	-0.03742	-0.05544	0.130897	0.010533	-0.26087	0.22519	-0.12317	-0.0719	0.118491	-0.02675	-0.00559	0.019383	0.089058	0.08215	-0.13782
65	intake165	ピ ー マ ン	Green peppers	0.008366	-0.2164	0.063983	0.179315	-0.02787	0.043102	-0.09108	0.146538	-0.26627	0.087785	-0.0065	-0.01835	-0.10761	-0.14372	0.186128	0.016062	0.159542	-0.23961	0.000941	-0.03803	0.036292	-0.16646	0.005558	0.056675	0.064346	0.053054	-0.01847	-0.02569	-0.08344	-0.0122	0.017996	0.04822	-0.06577	-0.02369	-0.00548	0.064923
66	intake166	生しいたけ	fresh	0.139027	-0.02124	-0.15808	0.037056	-0.12176	0.123466	0.025464	-0.11429	0.075583	-0.15805	0.074667	0.08646	0.050313	-0.11242	0.084684	-0.07157	-0.07216	0.142108	-0.08279	0.051999	0.035767	-0.16973	-0.17185	-0.03128	0.168308	-0.10512	0.298768	-0.17701	-0.05319	-0.06846	0.058479	-0.09724	0.274834	-0.19377	0.10677	-0.03965
67	intake167	干ししいたけ	dried	-0.02334	0.049044	0.018385	0.019187	0.017187	0.021534	-0.30932	0.14602	-0.03817	-0.00475	0.136367	0.054937	0.084023	-0.15236	0.169163	-0.15352	0.069932	0.155204	-0.08179	0.052711	-0.20977	0.0123	0.234111	-0.2794	0.239801	0.010766	-0.02097	-0.15073	-0.06865	0.168264	0.091637	0.126104	-0.27419	0.038493	-0.14148	0.191663
68	intake168	わ か め	“Wakame”, sea	0.203899	-0.03875	0.046533	-0.12682	0.101831	0.059892	-0.07029	-0.04587	0.055858	-0.12281	0.006193	0.052499	-0.05896	-0.04016	-0.06272	0.019214	0.267811	-0.06749	0.008965	0.029852	-0.02753	0.017528	0.085686	0.087516	-0.03055	-0.14425	0.071112	-0.09083	-0.04425	0.240413	-0.13115	-0.00402	0.09802	-0.13396	0.019766	-0.06665
69	intake169	こ ん ぶ	Dried tangle	0.184792	0.002464	0.01776	0.011577	0.103983	-0.06095	-0.06396	-0.03629	0.069259	-0.00318	-0.04406	-0.01982	-0.0132	-0.04516	0.087391	-0.1997	0.252348	-0.22495	0.209595	-0.13567	-0.08527	-0.00456	0.128785	-0.24173	0.290788	0.124169	-0.16567	-0.08511	0.021071	0.132433	-0.07864	-0.04149	0.075685	0.100826	0.047311	-0.07377
70	intake170	豆 腐	Bean curd	0.085772	-0.01593	0.098875	-0.06455	-0.19641	0.19352	0.01642	-0.12549	-0.01799	-0.0092	-0.00213	-0.023	0.081649	0.059566	-0.15347	0.146811	-0.00776	-0.06439	-0.02906	0.097799	0.172725	-0.25221	0.251467	-0.07597	-0.06423	0.06275	-0.12611	0.216103	-0.13569	0.030694	0.012212	0.191761	-0.12618	-0.18991	0.324462	-0.26569
71	intake171	梅 干 し	pickled plums	0.121085	-0.05934	-0.12058	-0.03511	0.052357	-0.03095	0.117559	0.075735	-0.12525	0.10572	0.001611	0.074314	0.181455	-0.14203	-0.10858	-0.14317	0.209368	-0.02475	0.133957	-0.29156	0.194779	0.104384	-0.16028	-0.03227	0.061485	-0.13517	0.091157	0.211354	-0.31578	0.085087	0.022989	-0.01571	-0.10829	0.157208	-0.21412	-0.02295
72	intake172	だいこん漬	Pickled radishe	0.145463	0.0073	-0.17111	0.000276	-0.01964	0.210701	-0.04709	-0.07472	0.136826	-0.02094	-0.07572	0.098069	0.095332	-0.05089	0.03751	-0.08107	-0.04404	-0.06693	0.125721	-0.19188	-0.0499	0.117852	0.038859	-0.10225	0.041867	-0.24514	0.193816	0.100713	-0.19137	0.160909	-0.02213	0.037212	0.104422	-0.07639	-0.02777	0.122189
73	intake173	はくさい漬	chinese cabba	0.025302	0.029014	-0.0274	0.031777	-0.13406	0.097299	0.060763	-0.20587	0.069738	0.270745	-0.16464	0.071708	-0.07559	0.284045	-0.2601	-0.0245	0.201718	-0.07193	0.079812	-0.05362	0.051523	-0.04052	-0.21056	0.310702	-0.24563	0.28463	-0.10656	-0.13016	0.163825	-0.14596	0.121988	-0.05219	-0.06893	0.058253	-0.06922	0.077966
74	intake174	こんぶつくだ煮	in soy sauce	0.097581	-0.0404	0.021428	0.016922	-0.09586	0.09432	0.044529	-0.14619	0.267296	-0.0145	-0.03358	0.101447	-0.05946	-0.17693	0.084113	0.119643	-0.10826	-0.17619	0.100557	-0.07264	-0.0129	0.063188	0.164479	-0.28067	0.213233	0.017427	-0.00795	-0.24672	0.111573	-0.06278	0.337509	-0.2527	0.073984	0.190965	-0.18495	0.037529
75	intake175	り ん ご	Apples	0.205692	0.047488	0.022846	-0.08843	0.029388	-0.1072	-0.02271	0.003986	-0.01625	-0.17212	0.124581	0.106817	0.020522	-0.1869	-0.02012	0.013669	0.220547	-0.13498	0.046868	-0.04124	0.043673	-0.09668	-0.08211	0.087096	0.071747	0.069604	-0.05395	0.009652	-0.07301	0.14635	-0.12436	0.008135	0.021971	0.107092	-0.12131	0.06609
76	intake176	み か ん	Mandarin oran	-0.03388	0.060722	0.016379	-0.02994	-0.00126	0.203156	-0.23713	0.042537	0.08498	-0.19592	0.136638	-0.05317	-0.21073	0.112181	-0.0896	-0.09778	0.267398	-0.03151	-0.07581	0.037981	0.097142	0.075466	-0.02494	-0.01885	0.0955	-0.06963	-0.07581	0.28961	-0.23127	-0.11516	0.275646	-0.24062	0.075018	-0.17989	0.075405	0.10289
77	intake177	グレープフルーツ	Grapefruits	0.135355	-0.19085	0.064028	0.016523	0.003923	0.06182	0.068182	0.058462	0.003703	-0.08351	-0.0857	0.044958	0.067619	-0.20212	0.177269	-0.07275	0.034169	-0.00333	-0.02465	0.009628	0.121482	-0.16867	0.255479	-0.03434	-0.13175	0.073552	0.041155	-0.14555	0.131944	-0.18176	0.130104	-0.01058	-0.1078	0.090317	0.086989	-0.05454
78	intake178	オ レ ン ジ	Oranges	0.091978	-0.12866	0.086366	0.119376	-0.15421	-0.08817	0.131319	-0.00649	0.00645	0.115698	-0.26704	0.233375	-0.11934	0.031293	0.052822	0.034129	0.23407	-0.0798	-0.10146	0.028669	-0.02542	0.03263	-0.14451	-0.03526	0.009483	0.016809	-0.1468	0.233312	-0.03245	-0.02277	0.099945	0.001035	-0.10315	0.070368	-0.02884	0.083243
79	intake179	な し	Pears	0.098393	-0.02061	-0.1202	-0.12805	-0.13855	0.170806	0.100961	-0.09545	-0.16578	0.199503	-0.00307	-0.0017	-0.22654	0.285368	-0.02904	-0.09974	0.053666	0.005046	-0.25905	0.118266	0.155706	-0.00474	0.045583	-0.0963	0.093282	-0.0866	0.040023	-0.1027	0.050236	-0.04074	0.117416	-0.01901	0.063904	-0.06883	0.005028	0.03035
80	intake180	ぶ ど う	Grapes	0.057669	0.008359	0.135344	-0.19909	-0.06687	0.073566	0.093869	-0.09782	-0.02622	-0.01382	0.089103	-0.115	0.07144	-0.15334	0.020611	0.288732	-0.11308	0.097709	-0.0933	-0.05407	0.037765	0.196727	-0.08079	0.035587	-0.28039	0.400914	-0.17009	0.076584	-0.18992	-0.02845	0.142608	-0.12667	0.153071	0.011941	-0.25779	0.033794
81	intake181	か き(果物)	Persimmons	-0.02045	-0.02061	-0.07405	0.053546	-0.12365	0.086598	-0.00168	-0.00961	0.090986	-0.16112	0.099973	0.051327	0.010781	-0.19013	0.128611	0.050074	-0.08075	0.097008	-0.32039	0.143689	0.203595	-0.21892	0.317272	-0.22309	-0.14892	0.334852	-0.1602	0.049157	0.070685	0.059259	-0.25833	0.260638	-0.34063	0.164549	-0.0919	-0.02627
82	intake182	も も	Peaches	0.028242	0.042204	0.091766	-0.1878	-0.04182	-0.0668	0.188011	-0.15562	-0.00636	-0.0425	0.228046	-0.0708	0.060153	-0.02169	0.0678	-0.0056	-0.18348	0.152551	0.09019	-0.31834	0.13561	0.254739	-0.32692	0.088101	0.080558	0.058739	-0.23455	0.107741	0.186196	-0.11103	0.085465	-0.02258	-0.13812	0.075102	0.167482	-0.0805
83	intake183	す い か	Watermelons	-0.01753	0.002281	-0.05022	0.076238	-0.12502	0.201909	0.013537	-0.03996	-0.11989	0.032603	0.10627	-0.25079	0.229404	0.014099	0.147296	-0.30472	0.138063	0.014731	-0.17077	0.177398	0.047345	0.009292	0.051268	-0.00935	0.0569	-0.13803	-0.0533	0.169158	0.002386	-0.04167	0.001818	-0.10974	-0.00587	0.375584	-0.32843	-0.17124
84	intake184	メ ロ ン	Melons	0.109852	-0.02344	-0.05781	-0.08431	0.062831	0.035644	-0.0682	0.08635	-0.02382	-0.1435	-0.03588	0.318217	0.041791	-0.11988	-0.00653	-0.10035	0.063223	-0.19595	-0.02592	0.423846	-0.14607	0.151074	-0.25674	0.118188	0.047336	-0.0672	-0.07934	0.012037	0.075125	-0.07245	0.095416	0.026656	-0.05126	-0.02888	-0.03171	0.091812
85	intake185	い ち ご	Strawberries	0.037255	-0.0654	0.059667	-0.20512	-0.12237	0.249228	-0.03092	0.042534	0.04197	-0.09616	0.011844	0.190692	-0.06129	0.0888	-0.17924	0.012362	0.040418	-0.03222	0.140241	0.050898	-0.01011	-0.05978	-0.07454	0.160291	-0.20394	0.213223	-0.04191	0.008857	-0.16356	0.188155	-0.16903	-0.00529	-0.04144	0.173544	0.030001	-0.03085
86	intake186	バ ナ ナ	Bananas	0.038681	-0.02554	0.000463	0.108397	-0.30324	0.122236	0.056012	-0.02671	0.053633	-0.19556	0.120419	0.056215	-0.15391	0.028099	0.149791	-0.04567	-0.16037	0.116419	0.055415	0.034131	0.009364	-0.09228	0.10259	-0.11322	-0.05385	0.161286	-0.19652	0.208056	-0.11975	0.09235	0.099928	-0.12906	0.010582	-0.05859	-0.20324	0.288769
87	intake187	キウイフルーツ	Kiwi fruits	0.099711	-0.18688	0.042288	0.004216	-0.1188	0.179819	0.00281	0.04022	-0.07022	0.009102	-0.03178	-0.00248	-0.03336	-0.01294	0.071205	0.054109	0.032826	-0.06094	-0.15226	-0.06567	0.078993	0.10593	-0.05013	-0.12012	0.214814	-0.3484	0.200361	0.045996	-0.05858	0.013122	-0.04682	0.164636	-0.1698	0.147779	-0.05649	0.1622
88	intake188	食 用 油	Edible oil	-0.01553	0.142845	-0.11276	0.019186	0.001155	-0.07808	-0.06157	0.222676	-0.06647	-0.09481	0.029284	0.026651	0.033029	0.187888	-0.15325	-0.10125	0.170723	-0.08962	0.027779	-0.2848	0.183556	-0.33909	0.342746	-0.04829	-0.09239	-0.0617	0.051588	0.198888	-0.11881	-0.10477	0.052373	-0.01886	0.034433	0.094707	-0.14554	-0.0947
89	intake189	マーガリン	Margarine	0.038384	0.017093	-0.01375	0.265707	-0.28126	0.035183	0.099642	-0.06849	-0.00715	0.146819	-0.0256	-0.00177	-0.02771	-0.17255	0.00012	0.099985	-0.00653	-0.00937	-0.00591	0.016729	0.018425	-0.09719	-0.04727	0.098796	0.102026	-0.1649	-0.12533	0.17067	-0.15941	0.233566	-0.06542	-0.04107	-0.00648	0.143357	-0.06738	0.042209
90	intake190	食 塩	Salt	0.142961	0.197249	0.227672	0.057712	0.156155	0.020497	0.051731	0.050937	0.037429	0.101875	-0.04065	-0.01929	0.047587	-0.04586	0.099942	-0.03137	-0.05067	0.034486	-0.02176	0.047408	0.004355	-0.01748	-0.05935	0.051803	-0.04743	0.072642	0.013532	-0.03916	-0.03943	0.049119	0.000239	0.000899	-0.00611	-0.02103	0.030554	-0.03677
91	intake191	しょう油	Soy sauce	0.133181	0.099148	-0.02079	0.076308	-0.03347	0.180063	-0.10415	0.050052	-0.03267	-0.10229	0.060797	0.174335	-0.06755	0.143127	-0.12751	0.060916	0.058356	-0.09861	-0.05653	0.095544	-0.01166	-0.06495	0.019773	-0.11382	0.062959	0.113035	-0.26852	0.199433	0.210969	-0.3208	0.062609	-0.00529	0.021322	-0.01389	0.011534	0.148855
92	intake192	み そ	“Miso”, soybe	0.12452	0.124352	-0.0261	-0.13249	0.067241	0.037687	-0.11035	0.088363	-0.04087	-0.07779	0.123025	-0.08818	-0.12705	0.112064	-0.03109	0.093312	-0.07237	0.097385	-0.03985	-0.06824	-0.02703	0.095383	0.003818	-0.08615	0.21313	-0.17907	0.025752	0.068029	0.026287	0.020065	-0.1604	0.096924	-0.10297	0.359372	-0.27296	0.200384
93	intake193	砂 糖	Sugar	0.002975	0.188142	0.018638	-0.06108	-0.13327	0.054464	-0.11071	0.098139	0.10865	-0.04179	0.088665	-0.00196	-0.28197	0.001776	0.261331	-0.31618	0.195296	-0.03308	0.05484	-0.08067	0.029325	-0.22832	0.222198	-0.03192	0.026537	-0.12556	0.037938	0.01226	0.114641	-0.21962	-0.135	0.134368	0.090208	0.041034	-0.06398	0.005276
94	intake194	酢	Vinegar	0.03628	0.038305	0.025431	-0.13119	0.005024	0.126624	-0.10146	0.212982	-0.12461	-0.26636	0.03351	-0.03144	0.017056	0.055513	0.129194	-0.01481	-0.34614	0.219111	0.249166	-0.21886	0.129114	-0.09961	-0.19237	0.100835	0.137347	-0.0631	0.038812	-0.12387	0.026835	0.011542	-0.04098	0.078902	-0.15128	0.24627	-0.14745	0.049926
95	intake195	ソ ー ス	Worcester sau	-0.04611	0.046007	0.005548	0.259662	-0.20986	0.002418	0.098675	-0.17044	0.112126	-0.13278	0.144212	0.02922	0.127617	-0.11148	0.05529	0.011532	0.016019	-0.05114	-0.0414	-0.09364	-0.02741	-0.1531	0.145698	0.30734	-0.25144	-0.13529	0.029598	0.053219	0.078792	2.23E-06	-0.04871	0.003515	-0.03799	0.001501	0.132589	-0.03186
96	intake196	ケチャップ	Tomato ketchu	-0.07115	0.009978	-0.02782	0.223884	-0.02513	0.007927	-0.09182	0.116006	-0.11305	-0.00048	0.0636	0.055675	-0.00079	-0.06965	-0.14873	0.341303	-0.22256	-0.02967	-0.02439	-0.08434	0.185647	-0.01254	0.060222	-0.29572	0.341095	-0.12328	0.055659	-0.11342	0.107536	0.005082	-0.01594	-0.0296	0.006691	-0.03862	-0.15554	-0.14628
97	intake197	マヨネーズ ・	seasoning	0.073262	0.147008	-0.09211	0.077384	-0.1394	0.102782	-0.17773	0.041133	-0.05647	-0.05199	0.015365	0.079403	0.015451	-0.00377	-0.20354	0.30656	-0.1217	0.172034	-0.22712	0.114001	-0.11785	0.00529	0.060968	-0.00093	0.034811	-0.12787	-0.09616	0.012028	0.221045	-0.06151	-0.08587	0.170267	-0.02833	0.16262	-0.07236	-0.0476
98	intake198	ドレッシング	Dressing	-0.00486	0.007474	0.200189	-0.05921	-0.14117	0.027183	-0.0538	0.133341	-0.10602	0.225056	-0.3415	0.125978	0.021592	-0.20595	0.152967	-0.05148	0.192927	-0.07191	-0.03264	-0.14532	0.100295	0.144951	-0.11185	-0.08104	-0.06047	-0.01182	0.068747	-0.05687	0.186399	-0.11123	0.081396	-0.12243	0.125322	0.023896	0.08096	-0.18445
99	intake199	ジ ャ ム	Jam	0.088422	-0.10465	0.160452	0.022506	-0.12103	-0.00481	0.03292	-0.08034	0.02769	0.046627	0.054773	0.111355	-0.03091	-0.07938	0.055843	-0.13333	0.010454	0.163394	-0.04152	-0.1886	0.07203	0.218966	-0.07162	-0.00628	-0.05886	0.080121	0.011752	0.044304	-0.32278	0.248605	-0.27979	0.401217	-0.11282	-0.04933	0.0385	0.022077
100	intake1100	カレールウ	Instant curry r	0.133105	0.023514	-0.06391	0.110649	-0.09299	-0.07164	0.052769	0.145255	-0.16013	0.025785	-0.12536	-0.03795	0.016397	0.240436	-0.26922	0.169213	-0.06605	0.110288	-0.03068	0.067576	0.048344	-0.15369	0.27005	-0.15443	-0.04675	-0.1923	0.251621	-0.18407	0.112938	-0.02954	0.024494	-0.07766	0.08604	0.050073	-0.0063	-0.05018
101	intake1101	緑 茶	Green tea	-0.007	-0.10132	0.030938	0.017849	-0.02474	0.236231	-0.21527	-0.11663	0.154161	-0.08334	0.087213	0.030299	-0.1694	0.265547	-0.12858	0.01814	0.009074	0.095787	-0.15642	0.015141	-0.03232	0.213554	-0.10186	0.070786	0.109802	-0.11969	-0.0767	0.174795	-0.1287	0.155673	-0.20361	0.100191	0.099886	-0.19067	0.117981	-0.17423
102	intake1102	紅 茶	Black tea	-0.03983	-0.19036	0.145057	0.043147	0.008249	0.029329	0.00854	-0.01308	-0.09574	-0.02733	-0.06758	0.012493	0.185758	-0.17731	0.08405	-0.14134	0.161701	-0.04804	0.049449	-0.18534	0.321279	-0.15253	-0.06324	0.048975	0.064627	-0.11664	0.141592	-0.0377	0.067394	-0.08206	-0.08727	0.140107	0.167886	-0.00741	-0.24496	0.248175
103	intake1103	コーヒー	Coffee	0.055612	-0.05072	-0.00662	0.310262	-0.16057	-0.05451	0.083955	-0.09022	-0.14648	-0.02483	0.092718	0.068133	0.153898	-0.0309	0.010364	-0.18881	0.002826	-0.05671	0.036338	0.06979	-0.20075	0.197953	0.076811	-0.09808	0.026482	0.116069	-0.11455	0.091681	0.001167	-0.24595	0.197216	0.058159	-0.04611	0.069319	0.123968	-0.07198
104	intake1104	清 酒	“Sake”	0.177736	-0.07413	-0.02571	-0.01276	0.006872	0.006917	0.070556	-0.16414	0.121291	-0.11348	0.099467	-0.14259	0.024607	-0.02874	0.15092	-0.09284	-0.10808	0.073604	0.095575	-0.10651	0.04553	-0.03975	-0.25843	0.168689	0.140069	-0.34943	0.085978	0.095787	0.058043	-0.05709	0.116896	-0.10594	-0.10115	-0.06496	0.116864	-0.18195
105	intake1105	焼ちゅう	distilled spirits	0.003747	0.163032	-0.07461	-0.03752	-0.00191	0.014766	-0.22374	0.202577	-0.05007	0.068219	-0.12137	-0.10855	0.238475	-0.04612	-0.01236	-0.05677	-0.0332	-0.11271	-0.00969	-0.07611	0.13378	-0.01492	-0.00367	0.263296	-0.23245	-0.00173	0.040608	-0.02164	-0.12147	0.077686	0.222562	-0.03444	-0.27607	-0.06235	0.123492	0.057467
106	intake1106	ビール	Beer	0.125259	-0.01938	-0.0896	0.055684	0.049207	-0.09184	-0.13166	-0.01025	0.140118	-0.07942	0.149266	-0.02324	-0.00855	-0.0241	0.018168	-0.08883	0.147756	-0.01405	-0.24107	0.289441	-0.14925	-0.00126	-0.02172	0.080631	-0.00593	-0.131	0.211734	-0.09448	-0.09545	0.022568	0.066362	-0.10132	-0.04159	0.151779	-0.02936	-0.26417
107	intake1107	ウイスキー	Whisky	0.1669	-0.04476	-0.07856	-0.04097	0.072018	-0.05094	0.107971	-0.10523	0.061522	-0.01063	-0.11372	-0.02535	-0.08094	-0.12798	0.092016	-0.01742	0.019316	0.24113	-0.05231	0.032398	-0.12207	0.050033	0.045623	0.075549	-0.28543	0.333317	-0.14246	0.012912	0.065403	-0.20893	0.113906	0.273293	-0.04378	0.074951	-0.10042	-0.12106
108	intake1108	ワイン	Wine	0.115646	-0.20054	0.001424	0.033459	0.128057	-0.01726	-0.15562	0.066662	0.012644	0.116145	-0.01007	-0.09491	-0.02064	-0.08891	0.16118	-0.05031	0.000172	0.016778	-0.09253	-0.03033	0.04282	-0.0507	-0.1599	0.132546	0.007436	-0.08018	-0.09115	0.100663	0.136696	-0.13504	0.113845	0.077886	0.017159	-0.12703	-0.09242	0.00878
109	intake1109	発 泡 酒 ・	alcoholic bever	0.051602	0.072743	-0.16299	0.152493	0.005806	-0.10548	-0.09145	0.022855	-0.05501	0.172861	-0.06333	-0.14705	0.187449	0.135253	-0.18312	0.078071	0.103352	-0.21018	0.225296	-0.01409	0.137186	-0.03647	-0.15112	-0.00214	0.057457	-0.0143	0.088279	-0.08491	0.014405	-0.22581	0.237448	0.163223	-0.26178	0.185113	-0.09258	0.018133

Factor37	Factor38	Factor39	Factor40	Factor41	Factor42	Factor43	Factor44	Factor45	Factor46	Factor47	Factor48	Factor49	Factor50	Factor51	Factor52
-0.16216	0.200693	-0.07992	-0.02844	0.122669	-0.00106	-0.05296	-0.0123	-0.08455	-0.05603	0.069948	0.014697	-0.09205	-0.19706	0.18906	0.07373
0.059242	-0.12833	0.019367	-0.03964	-0.02109	-0.12742	0.142814	0.017554	-0.12565	0.1324	0.119107	0.039645	-0.11749	0.047643	0.048403	-0.01314
0.088173	0.076237	-0.01873	-0.14321	-0.05552	0.094847	0.153184	-0.11741	0.050851	0.116019	-0.04944	-0.04409	0.086655	0.081587	0.042676	0.03613
0.017193	0.046302	-0.04852	-0.1112	0.078931	-0.0258	0.013178	0.000152	-0.06359	0.078048	-0.10132	0.072995	-0.00615	-0.00349	0.230215	0.102049
-0.16576	0.17992	-0.20401	0.07914	0.068629	0.014275	0.068959	-0.13424	-0.009	0.012208	0.078453	0.017794	0.044521	-0.0651	0.030726	0.106793
0.019671	0.002694	0.122871	-0.24383	0.206254	-0.12942	0.065124	0.07406	0.145646	-0.03873	-0.11255	-0.04447	0.062713	-0.0057	-0.03081	0.029102
-0.0881	-0.08785	-0.13066	0.073098	0.054477	0.046284	-0.06986	0.023669	0.092439	-0.16919	0.070684	0.021462	0.014681	-0.02871	-0.12718	0.028316
-0.14156	0.188655	-0.00641	-0.03436	0.032077	0.369059	-0.1162	0.058577	-0.0143	-0.12648	0.095077	-0.1186	0.177977	-0.11041	0.20469	-0.09168
0.147037	-0.05438	-0.02006	-0.10336	0.123484	0.013031	0.058344	0.024865	-0.15916	-0.03787	-0.17265	0.14064	0.127715	0.022755	-0.037	0.038741
-0.11092	-0.00574	-0.10268	0.004397	0.030224	0.005987	0.150459	-0.10588	0.044089	0.090086	-0.13635	0.04967	0.145813	-0.07639	0.036568	-0.06359
0.051251	0.024619	-0.10068	0.052757	-0.03897	0.13055	-0.01571	0.084164	-0.08508	-0.02537	-0.01293	-0.1285	-0.00723	0.106553	0.050811	-0.04867
-0.00092	-0.03167	-0.14814	0.080937	-0.13767	-0.10882	0.060732	-0.12396	0.069144	0.04989	0.122423	-0.15689	0.049715	-0.00298	0.059504	0.067003
0.013211	-0.04477	-0.02326	0.123696	-0.05762	0.02258	-0.01336	0.082536	-0.03853	-0.0067	-0.09097	-0.03021	0.076992	0.067133	0.070396	-0.04788
0.018177	0.112116	-0.07241	0.27115	-0.07788	-0.00181	-0.07503	0.003259	0.077144	0.014207	-0.02576	-0.08195	-0.0009	-0.02191	0.128513	-0.02882
-0.14857	-0.04554	0.227587	-0.11029	0.063699	0.012697	0.028217	-0.09958	0.047142	0.006162	0.008441	-0.0667	0.025868	0.009165	0.176984	-0.2095
-0.02879	0.130264	-0.07351	0.041621	-0.07949	0.108203	0.089215	-0.05138	-0.06825	0.138233	-0.0419	-0.08052	0.030251	-0.05195	-0.06809	0.067812
0.125338	0.062281	-0.06593	-0.01111	0.06784	-0.02462	0.042239	-0.15024	0.065161	-0.15595	0.084828	0.06411	0.008258	-0.05841	-0.03549	0.005559
-0.07279	0.076117	0.147576	-0.03679	-0.03514	-0.0377	-0.02497	-0.1917	0.148408	0.020774	-0.20217	0.032823	0.053334	0.043436	-0.03226	-0.02205
0.07587	-0.03322	0.10885	-0.03902	-0.01897	0.080268	-0.0368	-0.05078	-0.00159	-0.04895	0.005674	-0.00992	0.109262	-0.05	-0.23553	-0.023
-0.01788	-0.08179	0.044198	0.083238	-0.01978	-0.12361	0.122506	-0.03954	0.166633	0.153274	0.016784	-0.05761	0.090074	0.034788	-0.05146	0.075902
-0.08175	0.029113	0.142826	-0.0577	0.167273	0.012868	-0.06438	0.163159	-0.06356	0.173366	-0.13363	0.084327	-0.10249	-0.04451	-0.06299	0.104385
0.050879	-0.04484	-0.1893	0.177401	0.146042	0.057088	-0.03089	0.016232	0.018199	0.150352	-0.15048	0.149369	-0.089	-0.1238	0.151681	-0.07819
0.18361	-0.02232	-0.0696	0.163942	-0.07434	-0.09655	0.004556	0.050854	-0.17224	0.026147	-0.04857	-0.05361	-0.12762	0.215676	-0.16036	-0.07334
0.336804	-0.05249	-0.01446	-0.14829	0.166351	-0.13668	0.094374	-0.09043	-0.03895	0.034941	0.00887	0.149599	-0.0627	-0.19119	-0.07206	-0.13199
0.078891	0.029784	-0.12326	0.064293	0.205354	-0.08557	-0.01474	0.085564	0.09805	0.00865	-0.10316	-0.07084	0.054722	-0.01837	0.051007	-0.20441
-0.07304	0.049	-0.17041	0.126003	-0.10896	0.166496	-0.07515	-0.17159	0.085923	0.014393	-0.00785	0.11942	0.089214	0.058381	-0.043	-0.0801
0.107499	-0.04378	-0.05769	-0.0465	-0.0568	0.231124	-0.03878	-0.05142	-0.08058	-0.12535	0.058225	-0.09595	0.103741	-0.02924	-0.06932	-0.07379
-0.00511	-0.11658	0.068068	0.096436	-0.17418	0.118613	0.004528	0.012832	0.178185	-0.13667	-0.0093	0.108204	-0.12687	0.038614	0.025361	0.002687
0.077242	-0.14073	0.149585	-0.06652	-0.08128	-0.02309	0.140355	-0.06123	-0.04477	-0.00657	-0.06814	0.004796	-0.2144	-0.19257	0.057782	-0.02873
-0.07806	-0.08422	0.199917	-0.05449	0.111497	0.002669	0.181472	-0.05081	-0.04859	0.059955	-0.1163	-0.00732	0.019977	0.042176	0.298698	0.044687
0.058462	-0.0269	0.117704	-0.13387	0.061311	-0.0908	-0.11727	0.143369	0.067207	-0.07346	0.171897	-0.02576	0.178321	0.015233	0.111302	-0.00353
0.066983	0.00248	0.074379	-0.04394	-0.04067	0.103134	-0.02602	-0.03259	0.119599	-0.3001	-0.14256	-0.03944	0.043263	-0.01367	0.07281	0.243164
0.171223	-0.14479	0.145798	0.014209	-0.06024	-0.00722	0.062108	-0.26974	0.027069	0.052928	0.104997	-0.15639	-0.03906	0.007235	0.030843	-0.15725
0.04409	0.056977	-0.00547	-0.05229	-0.0423	-0.03207	0.076788	-0.01065	0.103936	0.013898	-0.00607	0.018893	-0.04602	-0.10922	0.004669	0.050249
-0.02261	0.027979	-0.11626	0.100411	-0.01599	-0.05553	0.049935	-0.08931	0.118961	0.036857	-0.11381	-0.03056	0.111175	0.075041	-0.04479	0.03918
0.173324	-0.13824	-0.01521	0.100518	-0.17981	0.006477	0.277958	-0.20506	-0.04114	0.082359	-0.04484	0.055195	-0.11117	0.068469	0.025492	-0.00974
-0.03051	0.014656	-0.03927	-0.07809	-0.11871	0.002232	-0.07183	0.156348	-0.18267	0.037648	0.159711	-0.17238	0.034205	-0.10185	0.034923	-0.00924
0.080445	-0.13323	-0.08896	-0.14716	-0.04527	0.053528	0.038949	-0.02573	-0.01383	-0.0715	-0.13875	0.027774	0.199811	0.003096	0.04549	-0.01708
0.066176	-0.10443	0.015373	0.016455	0.09164	-0.06809	0.079305	0.020104	0.044149	-0.22678	-0.03047	0.209041	-0.16835	0.087984	0.138392	0.014582
-0.02524	0.13723	-0.09448	-0.03072	-0.04785	0.14958	-0.0227	0.18312	-0.06258	-0.07075	-0.05138	0.125814	-0.03024	-0.04507	0.039805	-0.23241
-0.02136	0.170395	-0.09377	-0.01547	-0.01443	-0.21835	0.071993	-0.12341	0.085249	-0.08513	-0.1618	0.195645	-0.10181	0.222787	-0.05011	-0.06235
-0.20625	0.049834	-0.13187	0.078384	-0.04404	-0.09805	0.060679	-0.01508	0.137374	-0.09107	-0.06541	0.048607	-0.08693	-0.06687	-0.11236	0.065559
-0.01865	-0.00924	-0.02465	0.029501	-0.01237	-0.01136	0.026178	-0.01054	0.216281	-0.0966	-0.13967	0.150047	-0.03314	-0.10646	0.012372	0.010207
-0.1161	-0.00176	0.100978	-0.10941	0.107165	0.055909	-0.08847	-0.09233	-0.08307	-0.06543	-0.09954	0.200302	-0.08686	0.016538	-0.21545	0.091852
-0.07612	0.018713	-0.01392	-0.01405	0.216001	-0.15138	-0.10321	-0.13444	0.060484	-0.07473	0.022882	0.030811	0.137503	-0.03087	0.150131	0.017814
0.04209	-0.11396	-0.02385	0.056986	-0.06345	0.106134	-0.23552	0.244482	-0.10372	0.009701	-0.00929	-0.05696	0.070323	0.069484	-0.03638	0.015765
0.024743	-0.05199	0.050241	-0.09393	0.073458	-0.00129	0.043449	-0.06942	0.054556	0.045311	0.005328	-0.00853	-0.1143	0.019237	-0.0387	0.215421
0.121647	-0.03323	-0.032	0.03696	-0.04227	-0.04255	-0.18769	-0.02294	0.076076	0.046831	-0.04064	-0.0611	0.116677	0.018614	-0.05869	0.14753
-0.18472	0.019823	-0.01693	0.01709	0.029923	-0.07184	0.098537	0.02958	0.04381	-0.15881	0.07197	-0.01646	0.061105	-0.07001	-0.1158	0.127017
-0.03245	0.031469	0.042578	-0.06483	-0.03573	-0.12218	-0.03497	0.019302	-0.01463	0.058718	-0.14477	-0.02055	-0.02077	0.150958	0.017655	0.078517
0.04293	-0.05166	-0.00427	0.286703	-0.11847	0.05859	-0.15904	0.157472	-0.18002	-0.06187	-0.0737	-0.01941	-0.12951	0.115831	-0.09139	-0.07961
0.041269	-0.09551	0.061547	-0.06017	0.041459	-0.16719	-0.01639	-0.02977	0.097761	0.020097	0.099936	-0.07897	0.162813	-0.05062	0.041516	0.039429
0.028859	-0.02389	0.092421	-0.14559	-0.02243	0.230666	-0.04647	-0.21513	0.208684	-0.10501	-0.01276	0.036939	0.009122	-0.22066	0.01148	0.03244
-0.11023	-0.03477	0.050147	-0.0408	0.004747	0.074521	-0.01697	0.121697	-0.00437	-0.05988	0.098183	0.013897	-0.06879	-0.01599	-0.02893	-0.03835
-0.11941	0.174201	-0.00752	0.026996	-0.01219	0.059793	-0.08244	0.011365	-0.04764	-0.02146	0.086918	-0.09159	-0.09561	0.103874	-0.01391	-0.05628
-0.10799	0.152215	0.050289	0.08196	-0.12511	0.01206	-0.07929	0.10857	-0.04803	0.085237	-0.08713	0.157613	-0.05591	-0.11279	0.175616	-0.00725
0.064738	-0.00922	0.030239	0.027389	-0.00693	0.123546	-0.16981	0.057048	-0.13563	-0.05262	0.185495	-0.20757	0.054679	-0.12707	0.117661	0.076051
0.28281	-0.21815	-0.05517	0.123101	-0.07864	0.02741	-0.03106	0.054142	-0.02611	0.025745	-0.09396	0.038659	-0.0827	-0.06057	0.156196	-0.01839
-0.14014	0.139036	-0.10133	0.085325	-0.08191	0.161698	-0.25619	0.090939	0.095516	0.126577	0.079246	0.009944	-0.09123	0.097452	-0.01497	-0.23692
0.133254	-0.06502	0.180709	-0.15079	0.142045	-0.00916	0.148246	-0.12309	-0.05108	0.107501	-0.04686	-0.05353	0.160112	0.128627	-0.08247	-0.00309
0.123945	-0.06362	-0.05743	0.009366	0.230354	-0.05401	0.022084	-0.1406	-0.01635	0.285533	-0.05977	0.113546	0.06575	-0.07113	-0.09772	-0.13625
0.134257	0.017811	-0.11374	0.037452	0.077188	-0.06149	-0.03432	0.08315	0.014931	-0.03286	0.179796	-0.10527	0.067869	-0.04368	0.093147	-0.02896
-0.09242	-0.08615	0.056648	-0.02581	-0.07511	0.076371	0.113114	-0.08025	-0.02226	-0.09127	0.125408	0.057409	-0.07092	0.074288	0.007556	-0.06605
-0.10148	0.114081	-0.26468	0.037756	0.045077	0.261086	-0.0504	-0.00967	0.075358	0.035847	-0.06692	-0.19673	0.043552	0.030554	0.044142	-0.00277
0.055342	-0.12336	-0.04143	0.03819	-0.05189	-0.00762	0.022089	0.081847	-0.01133	0.009393	0.190058	0.031588	-0.05885	0.057758	-0.00176	0.016788
-0.12114	0.103354	-0.01073	-0.08239	0.066328	-0.11059	0.053577	0.079714	-0.04684	-0.1348	0.052679	-0.0656	0.068164	0.208508	-0.106	0.041848
-0.10386	-0.07631	0.045949	-0.06224	-0.05624	-0.04393	-0.01767	0.102579	-0.25727	0.296074	-0.20792	0.016131	-0.01616	-0.04147	-0.06273	0.170901
0.030351	-0.11145	0.026339	0.06866	-0.06797	-0.00677	0.073598	0.101816	-0.17905	0.115499	-0.01657	0.127902	0.040281	0.07364	0.011766	-0.06007
0.041665	0.10432	0.051546	-0.06797	0.036752	0.005715	0.090598	0.008165	0.087327	-0.03433	0.048933	-0.12418	-0.09017	0.04973	-0.07472	0.127826
-0.04608	0.07696	-0.0534	-0.05648	0.072149	-0.23331	-0.00955	0.009226	0.048487	-0.12858	0.043421	-0.01918	-0.02431	0.013998	-0.03623	0.04607
0.199325	-0.14313	-0.09676	-0.08449	0.012269	0.083293	-0.03238	0.052428	0.096049	-0.0599	-0.09605	-0.13139	-0.17153	0.01907	-0.06411	-0.06211
-0.12232	-0.00922	-0.05797	0.05899	0.084106	-0.2613	0.07515	0.208417	-0.14305	-0.13692	0.117942	0.034032	0.096101	-0.03476	0.033926	0.134365
-0.04112	0.086385	-0.13232	0.089575	-0.08703	0.032072	-0.06271	0.137152	-0.07688	0.045927	-0.05509	0.129083	0.034515	0.001043	-0.10316	0.113719
0.020325	-0.15845	0.101696	0.029321	-0.03448	0.112852	-0.19049	0.002247	0.076578	-0.14856	0.148033	-0.0061	-0.0142	0.168788	-0.08658	-0.09633
-0.18032	0.119831	0.073281	0.045535	-0.02917	-0.15557	0.127129	0.010783	-0.00381	0.025138	0.02769	0.02679	0.012368	0.036801	-0.02136	-0.11187
-0.01772	0.061488	-0.03384	0.055465	-0.10761	-0.10421	0.107189	-0.19237	0.195498	-0.14455	0.051471	-0.06483	0.067784	-0.12565	-0.0197	-0.12101
-0.02392	-0.16418	0.12601	-0.03089	-0.02422	-0.18746	0.070049	0.233662	-0.04355	-0.06668	-0.06965	-0.00888	-0.01068	-0.30463	0.001773	-0.03976
0.049976	-0.03683	0.133825	0.020555	-0.09206	0.127056	-0.11954	0.040471	-0.00267	0.00791	-0.07472	0.163375	-0.08476	0.278193	-0.0698	0.080339
-0.04149	0.037852	-0.02205	0.128193	-0.24385	0.132927	-0.05536	-0.00697	0.050688	0.063536	-0.0244	0.072562	-0.07382	-0.15884	0.0715	0.099166
-0.08728	0.056361	-0.03733	0.005411	0.141978	-0.06226	-0.0594	0.079327	-0.19064	0.097133	-0.01875	-0.19294	0.280654	-0.11873	-0.03821	0.033403
0.01153	0.041119	-0.0431	-0.02847	0.04434	-0.14563	0.259163	-0.11497	0.025861	-0.1142	-0.0256	-0.04993	0.054415	0.189982	-0.10886	-0.06488
-0.0378	-0.12894	0.004804	-0.0189	-0.15066	0.137241	0.006286	0.008652	0.04732	-0.12834	0.021753	0.064886	0.158548	0.027656	0.036088	-0.09314
0.185779	-0.06564	0.239423	-0.02512	-0.04361	0.08917	0.000677	0.039796	-0.20984	0.036963	0.248874	-0.02111	-0.02131	-0.01313	0.023062	0.097913
0.221062	-0.23833	-0.17079	0.088249	0.029098	-0.07921	-0.04799	-0.14623	0.140641	0.008961	0.13412	-0.08025	0.030701	-0.13298	0.246258	0.048226
0.087947	0.048504	0.025637	0.123002	-0.20981	0.089242	-0.10521	0.195005	0.074806	-0.07477	-0.22891	0.261424	0.093802	-0.06903	-0.00817	-0.08003
-0.03901	0.117826	-0.02918	-0.08819	-0.07523	0.018119	-0.00253	0.016852	-0.02487	0.045325	0.130375	-0.00335	-0.14733	0.032595	0.120843	-0.02552
-0.26126	0.259454	-0.06971	0.066004	-0.07534	0.007527	0.041566	-0.16085	-0.00248	0.027162	0.019316	0.011193	-0.01408	-0.02241	-0.02903	0.014429
0.118891	-0.20005	-0.00743	0.176876	-0.01381	0.157168	-0.05423	-0.17379	0.099862	-0.01474	-0.01655	0.145742	0.020478	0.101521	-0.16208	0.106752
-0.09244	-0.01529	0.017248	-0.11718	-0.04977	-0.02299	-0.06292	0.057168	-0.19462	0.101856	0.034734	0.026083	0.082441	-0.10201	0.026392	-0.21559
-0.03288	0.060626	0.024225	0.090645	-0.0409	0.006558	-0.00696	-0.03194	0.010097	-0.04034	0.045489	0.012038	0.029035	-0.0731	0.013955	0.026837
-0.01946	-0.02991	-0.10754	-0.1774	0.139123	0.113466	-0.15781	0.075519	-0.06681	0.134355	-0.05308	-0.15705	0.008974	0.191152	-0.23708	0.006215
-0.00291	-0.18445	0.120373	-0.14176	0.223376	-0.18242	-0.04805	0.021476	0.168662	-0.04857	-0.10434	0.056434	-0.15292	0.094268	0.076156	-0.11305
0.082482	0.04437	-0.11518	-0.05192	-0.04062	-0.10991	0.187165	-0.04381	-0.15916	-0.06834	0.099922	-0.03439	0.0552	-0.10278	0.086958	0.057887
-0.13768	0.147408	-0.12219	0.0228	-0.15479	0.062846	0.038588	-0.07746	0.10385	0.123302	0.025902	0.015588	0.039391	0.062959	-0.01369	0.101096
-0.06658	0.135704	-0.19079	-0.06426	0.021272	-0.07699	-0.01733	-0.06883	-0.00284	-0.07509	-0.04832	0.014505	-0.15582	-0.11435	0.03657	-0.16215
0.310974	0.090815	-0.28383	0.098337	-0.03233	-0.03328	-0.04429	0.244547	-0.13213	-0.03316	-0.05421	0.118755	0.049543	0.054871	0.025321	0.029363
0.150119	-0.08369	0.103775	0.065368	-0.08585	-0.01105	0.022257	0.043503	0.123741	-0.16998	0.008648	-0.0774	0.164852	-0.08049	0.037157	0.106852
0.078517	0.132619	-0.03591	-0.19326	-0.01753	-0.01068	0.035117	0.084022	0.104234	0.038751	0.113065	-0.07273	0.03651	0.023504	-0.08106	-0.00991
0.127756	0.005018	0.159464	0.064542	-0.04046	0.071958	-0.17435	-0.04701	0.174946	-0.0742	0.232768	-0.23623	0.061423	-0.05822	-0.07067	-0.03502
0.023411	-0.14814	0.158214	-0.04646	-0.1375	-0.07608	0.050825	0.053161	0.018545	-0.03391	0.118001	-0.21022	0.172567	-0.01968	0.017156	-0.17231
0.078267	-0.05655	-0.00377	-0.1699	0.14646	0.060945	0.025797	0.166465	0.072569	0.002617	0.193251	-0.03993	-0.01266	0.060264	-0.12408	0.009009
0.005062	-0.10085	0.068842	-0.10848	0.01906	0.084594	-0.19702	0.03443	-0.12439	0.139036	-0.36859	0.103383	0.102975	-0.14129	0.1284	-0.11521
-0.0577	0.012631	0.069988	0.172762	-0.13221	0.011096	0.080796	0.039299	-0.11483	0.096949	-0.09353	0.008124	0.047571	-0.08083	-0.05846	0.090362
0.198523	0.029437	-0.05237	0.015724	0.08045	-0.03513	-0.03222	0.114693	0.008873	0.047027	-0.05671	0.074918	0.059475	-0.1549	-0.03449	0.075675
-0.07316	0.121218	0.136052	-0.29211	0.139704	-0.06962	-0.01059	0.140389	-0.02787	0.052817	0.054232	-0.00237	-0.13085	0.107609	0.048051	-0.03194
0.039037	0.060033	0.221596	-0.40569	-0.01059	0.193781	-0.2378	-0.01193	0.059716	0.024466	-0.17897	0.238733	-0.002	-0.03866	0.048247	-0.02427
0.086469	0.072785	-0.17772	-0.08485	0.085107	0.039401	-0.12148	-0.07309	0.054205	-0.04793	0.034901	0.0746	-0.21018	0.1283	0.073105	0.152073
0.025676	0.170412	-0.02527	0.185023	-0.19453	-0.28219	0.236291	-0.02178	0.033403	0.051896	-0.17983	-0.00399	0.125271	-0.00482	0.031526	-0.11822
-0.07908	0.129361	-0.0613	-0.01481	0.184622	-0.15097	0.195597	-0.0338	-0.05221	-0.16232	0.092681	0.027573	-0.04208	0.004614	-0.04384	-0.21882

Scores

No.	和名	英名	Factor1	Factor2	Factor3	Factor4	Factor5	Factor6	Factor7	Factor8	Factor9	Factor10	Factor11	Factor12	Factor13	Factor14	Factor15	Factor16	Factor17	Factor18	Factor19	Factor20	Factor21	Factor22	Factor23	Factor24	Factor25	Factor26
	全国	All Japan	-0.21522	0.067576	0.557788	0.224447	0.277325	-0.08545	0.4391	0.312158	0.204125	0.039316	-0.19293	0.022104	0.119996	-0.12632	-0.2647	-0.22183	-0.25903	-0.12881	-0.55772	-0.29334	-0.47404	-0.92379	-1.26311	-1.49368	-1.95052	-2.41057
1	札幌市	Sapporo-shi	1.139281	-4.45538	-4.28687	2.669946	1.801002	-5.79428	-1.4162	1.84317	2.398201	-1.42889	-0.31694	-0.59875	0.313445	0.534973	-0.78533	-0.42259	-0.66332	2.095069	-1.69868	0.181535	-0.29791	0.769602	-1.11829	-0.29486	0.822151	0.212059
2	青森市	Aomori-shi	11.77852	4.553139	-1.15638	3.302017	4.79113	-1.6133	-0.63788	-0.38483	0.002219	-0.73743	-2.37387	2.012828	0.218425	-1.45483	1.383911	-0.29001	-0.4172	0.861126	0.354462	-0.34157	0.925843	-0.43662	0.538405	0.828079	-0.81499	0.143937
3	盛岡市	Morioka-shi	8.492937	-0.50067	0.082021	-1.58915	-0.22252	-2.24511	-0.37201	0.977721	-1.2778	-2.30206	1.704865	0.227949	0.153228	-0.18605	-1.77766	1.775243	1.54644	-1.44094	-0.70971	-0.10162	0.962183	0.077529	-0.24594	-0.18644	0.769338	0.71086
4	仙台市	Sendai-shi	3.046064	-2.6324	0.111263	-0.4384	1.204172	1.036077	1.586904	0.982743	1.470605	-1.17927	-0.49688	-4.03611	0.495055	1.641748	0.629352	-2.00258	0.68935	-1.53768	-1.3058	0.569784	0.28866	0.284544	0.666586	0.38484	-0.17626	0.091158
5	秋田市	Akita-shi	7.902669	-1.88582	-4.788	-0.99307	2.393973	0.872763	0.259978	0.93379	-0.01716	1.319024	2.333927	-0.31233	-0.43961	-0.59603	0.789948	-0.16006	-0.57517	-0.94035	1.842254	0.43785	-0.40179	0.259692	-0.95929	0.992871	-0.87593	-0.82673
6	山形市	Yamagata-shi	4.574612	2.060652	1.401559	-0.49371	3.845457	1.187178	1.91884	-0.55242	0.017475	-0.14375	0.256725	-2.28059	0.238827	0.085585	-0.27087	0.509263	0.978235	1.066651	0.329233	0.270489	-1.10483	0.11271	1.202264	-0.79405	-1.24988	0.997812
7	福島市	Fukushima-shi	1.597282	-1.14213	-2.49477	-8.32603	-2.31598	-2.84553	0.504763	-3.86665	-0.98413	0.274311	1.227914	-0.48972	0.8487	0.214124	-0.07364	-0.3904	-0.99288	1.007717	0.687032	-2.76358	-0.05491	0.660856	-0.39708	0.368027	0.319832	0.04267
8	水戸市	Mito-shi	2.238729	-0.65539	0.195511	-1.98385	0.670049	-0.86514	0.422767	0.193798	-0.30424	-1.35599	0.585285	4.224742	-0.17076	-0.02905	-0.17662	0.715792	0.421693	-2.52543	-0.41565	2.670336	-1.28445	0.510662	-0.2805	0.028166	0.880948	0.415327
9	宇都宮市	Utsunomiya-sh	0.231287	-1.73347	1.301584	-1.54565	1.673901	1.436004	0.793363	-0.88821	-0.21577	-0.33775	-0.21459	0.503251	0.603598	0.126347	-0.96462	-0.80449	-0.73566	0.148225	1.712254	2.321188	1.395496	-0.48341	-0.07389	-0.59247	-0.61143	0.136502
10	前橋市	Maebashi-shi	1.204069	-0.35416	2.004146	-4.77262	-1.07239	-0.51552	-0.79324	0.384726	2.976597	0.350149	-0.93879	1.07105	0.36719	-0.81054	1.745656	-0.92169	0.089635	-0.58801	0.995514	0.350766	0.436128	-1.71057	0.60477	0.785808	0.554794	-1.17886
11	さいたま市	Saitama-shi	1.019656	-3.7361	2.146769	0.854452	0.372367	1.575474	0.575265	1.490027	-0.95398	0.842935	-0.44324	0.780799	2.209468	-0.03689	-1.24722	-0.53672	0.61098	0.132814	0.195471	-1.41904	0.38015	-1.00528	-0.67901	0.123368	0.145261	-0.35261
12	千葉市	Chiba-shi	1.439755	-4.88979	0.756213	-0.35199	-1.64325	0.848563	0.426088	0.402722	-0.84772	0.46105	-0.59275	0.38094	0.003363	0.785861	0.647863	0.889808	1.102194	-0.14305	-1.01125	-0.16066	0.607976	0.348034	-0.07528	1.115515	-1.03515	-0.11773
13	東京都区部	Ku-areas of T	-1.59639	-5.148	1.322762	-0.49479	0.348352	0.995087	0.238066	-0.17598	-1.12879	0.597572	-1.09563	-1.06901	-1.32166	-3.34768	-3.04463	-2.11094	-0.92678	-0.38518	-0.38963	0.205459	0.309296	0.802134	0.114706	1.737978	0.135446	0.447532
14	横浜市	Yokohama-shi	-0.58419	-4.00406	2.173607	-0.3215	-0.84421	0.284948	-0.46995	-0.00542	-0.34702	0.754877	-0.1969	0.579043	0.291956	0.105996	1.476351	-0.81012	1.869129	0.733519	-0.91199	0.485705	-0.56582	0.602879	-0.69417	-0.39773	-0.94367	-0.04442
15	新潟市	Niigata-shi	7.917817	-1.25267	-0.46954	0.187026	0.386833	1.997427	-0.76969	-1.25678	-2.12589	0.974901	1.271928	-2.65352	-0.55326	0.530135	0.324361	0.636151	-0.58212	0.53182	-0.62411	0.731568	0.633438	-2.07354	0.14887	-0.40939	0.917098	-0.00289
16	富山市	Toyama-shi	3.960438	-0.15	-1.33745	1.105769	-1.28121	2.339419	0.717907	-1.35861	-0.03858	1.157102	-2.07119	0.076256	0.601044	0.704166	-0.51612	0.576355	-0.00857	-1.97075	0.350681	-0.71958	0.049694	0.799531	0.076789	-1.32711	2.175092	-1.18807
17	金沢市	Kanazawa-shi	1.048044	1.888656	1.115963	2.187326	-0.72022	-1.35537	0.584013	-3.13914	-1.48416	0.554773	-2.96419	-0.8739	0.567467	0.168036	0.289826	-0.52466	-0.86146	-0.19912	0.55883	1.607151	-1.25714	1.230986	0.012725	-0.96146	1.25306	-0.33131
18	福井市	Fukui-shi	-1.92629	-0.63418	-2.90616	-0.95053	1.968506	1.543281	1.737237	-1.99796	2.804947	-0.31865	-0.81369	0.099268	0.296091	-0.9351	0.155691	0.69805	-0.06141	1.090525	-0.0809	-0.65771	-1.42104	-0.35164	0.856765	-0.31508	0.945322	-0.13308
19	甲府市	Kofu-shi	-0.48949	0.610152	3.980406	-2.37861	0.713218	-0.47177	-0.00915	-1.28731	0.367818	-0.13228	-2.39582	0.228033	-0.13379	-0.82327	0.657262	1.525371	-1.52452	0.153034	-0.16033	-0.34999	0.404474	1.125807	0.030388	-0.94988	-1.42566	0.91812
20	長野市	Nagano-shi	5.34839	5.725878	7.447376	-0.91352	-0.19798	-3.55712	-0.84936	1.004923	0.793505	1.560735	1.493269	-0.5254	-1.09726	-0.62229	-0.02526	-1.08539	0.671284	0.575942	-0.74064	-0.91332	-1.41223	0.089072	-0.2896	-0.49208	1.133599	-0.11185
21	岐阜市	Gifu-shi	-1.66299	-1.79383	-0.58038	-1.45352	-1.58355	0.496763	1.017804	0.372299	2.572595	0.209073	0.595014	0.841938	0.793798	-0.93652	-0.28054	1.265328	-0.91543	0.175219	-0.8413	1.020017	0.788085	-1.06241	1.427183	-1.26225	0.143235	1.436311
22	静岡市	Shizuoka-shi	-0.64243	-2.77049	2.434788	1.514658	3.074872	2.293518	-4.14819	-2.59296	1.817356	-0.06522	-0.03404	1.309476	-0.34925	2.555594	-1.56234	0.121105	0.158094	0.792299	-0.83344	-0.98243	-0.32237	-0.61501	-0.12361	0.626777	0.494627	0.054162
23	名古屋市	Nagoya-shi	-1.61112	-1.96561	1.141338	0.733091	-1.75372	-0.24805	1.406499	1.182143	-0.21116	-0.20975	-0.08176	0.145808	-0.57533	-0.7696	-2.11915	0.844539	-1.8702	1.613512	0.805874	0.769073	-0.70945	-1.39699	-0.79067	-1.27427	-0.42656	-0.23788
24	津市	Tsu-shi	-0.86304	-0.44431	-1.56285	-1.23274	0.712049	2.968642	0.211581	0.069372	-0.39573	-2.94406	0.604798	0.304667	-1.34152	1.086844	0.294783	0.26054	-1.29833	-0.45073	-0.57482	-0.2465	-0.9903	1.829963	0.508414	-0.31436	-0.37123	-0.70401
25	大津市	Otsu-shi	1.312312	-1.13267	0.571817	2.524966	-3.85037	-0.81268	-1.19058	1.206434	2.789298	0.687911	1.393427	-0.53788	-1.92907	-0.88548	0.416488	-0.36843	0.103959	-1.64398	1.538149	0.255829	0.378025	0.791219	0.180333	-0.53698	-0.20694	-0.01213
26	京都市	Kyoto-shi	-0.66172	-2.47897	0.423296	3.303016	-1.27012	-0.61988	-2.61747	-2.71109	-1.06167	0.372879	2.683624	1.653283	1.271902	-1.4321	0.460791	-1.39193	-0.00976	-0.49171	-0.54312	-0.80292	-0.37441	0.960324	2.059207	0.00042	-1.06826	-0.27837
27	大阪市	Osaka-shi	-1.46551	-0.80385	-0.74039	3.220275	-1.39284	-0.66144	1.344632	0.586117	1.45701	-0.42505	-1.11271	-0.49435	-0.60129	-0.60625	-0.02252	0.534846	-0.36571	-1.53061	0.464817	-1.2918	-0.1175	-0.83223	0.194313	0.862596	0.317797	-0.1611
28	神戸市	Kobe-shi	-4.75009	-1.66798	0.392602	2.255145	-0.03126	-1.07283	1.851382	-1.73234	-0.28753	0.310543	0.038295	-0.22921	0.882798	-0.35045	2.595847	0.793711	0.075337	-0.67402	-0.85602	0.204741	0.530302	-0.67978	-0.82257	0.936181	1.299967	-0.19116
29	奈良市	Nara-shi	-0.2435	-1.87224	-0.17272	1.822631	-2.32065	1.632589	0.419337	-0.5529	-0.28571	-1.24601	0.685085	0.807625	0.56779	-0.00364	1.807103	-0.33934	0.348872	-0.4747	-0.93837	-0.65538	0.169843	-0.6178	-0.74255	-1.48979	-0.96995	0.795024
30	和歌山市	Wakayama-shi	-2.02539	2.590756	0.420902	1.132195	0.050572	0.77328	0.238605	-1.21248	0.141697	-3.06208	0.44971	-0.38579	-1.59561	-0.46631	1.639234	-0.56092	-0.83786	1.010624	0.697664	-0.54613	1.817526	0.403871	-0.77318	0.042572	0.243942	-0.33937
31	鳥取市	Tottori-shi	2.426533	4.218069	-3.03006	1.358074	-4.38481	-0.743	1.860275	-1.53821	-1.50472	3.038094	0.039342	1.608661	-1.82928	1.813409	-0.72144	-0.50233	0.017017	1.497206	-1.79675	1.157314	1.117272	-0.28185	0.717238	0.077339	-0.3456	-0.353
32	松江市	Matsue-shi	0.935976	-0.04564	-2.51021	-0.92987	-1.06482	1.272815	2.800636	3.157262	-0.72957	0.382204	-2.22089	1.922243	-0.76131	-0.12697	0.671158	-1.20249	0.932212	1.277134	-0.52648	-1.75277	-0.70927	0.359781	-0.3166	-0.32852	-0.13649	0.813595
33	岡山市	Okayama-shi	-2.40785	3.445923	3.090635	1.19459	1.487862	1.189758	5.079513	0.906288	0.067943	0.542086	2.393433	0.530257	0.556151	0.05191	0.619998	0.8733	-0.84894	0.007866	0.033173	-0.32088	0.308263	1.015664	-1.24554	1.153783	0.433382	0.277472
34	広島市	Hiroshima-shi	-1.08107	2.288734	0.095228	1.30425	-2.55228	0.088621	1.458305	-1.1891	-1.71477	-1.29382	-0.0464	-0.73612	0.971615	0.143753	-0.26739	-1.06257	-0.84596	-0.44736	-0.60291	0.913499	-1.56517	-0.96052	-0.07216	2.149742	-0.36063	0.89475
35	山口市	Yamaguchi-shi	-0.47039	3.197215	-1.95874	0.061993	-1.93333	-1.66641	0.565987	1.885067	2.193172	1.885944	0.061084	-0.26898	1.393946	1.339425	-1.19757	-0.61477	-0.43592	-0.47604	0.864048	1.138169	-0.37432	0.365713	-0.22776	0.492742	-0.79519	0.631919
36	徳島市	Tokushima-shi	-4.09026	1.764603	-0.75387	-0.5921	0.917777	-1.0214	0.618738	-1.43304	-0.24333	-2.22532	1.123371	0.649587	0.22029	-0.93875	-1.63673	-0.509	1.998906	1.288455	0.012081	1.310036	-0.47435	-0.51926	-0.13358	0.055834	-0.13544	-1.57146
37	高松市	Takamatsu-sh	-1.46235	3.006727	0.488067	2.163847	-2.1132	-0.82279	2.321926	0.511928	-0.5073	-2.36819	-0.17541	-0.84606	0.369134	-0.13955	-1.84052	0.493577	2.137968	-0.10434	1.360514	-1.86009	0.980108	0.011696	0.840702	-0.4471	0.141531	-0.28537
38	松山市	Matsuyama-sh	-3.3056	3.711377	-0.20266	-0.4186	-0.76939	-2.09477	-0.72681	0.102048	0.85763	-0.56686	1.053119	-1.49127	0.187382	0.959758	0.731833	0.189565	-1.19249	-1.32007	-1.24023	-0.27212	-0.83774	-0.76508	0.307901	-0.22995	-1.0088	-0.19987
39	高知市	Kochi-shi	-3.09187	2.303872	-0.74585	1.061965	2.358361	0.459018	-0.86464	-3.23033	1.128505	1.278317	-0.66433	0.488263	-0.92423	1.929201	-1.36896	0.420109	1.238441	-1.62671	1.823312	-1.06099	-0.53851	-0.41724	-1.23457	0.757423	-1.08695	1.090825
40	福岡市	Fukuoka-shi	-3.76894	-0.93586	-0.48328	1.155217	2.172317	0.982002	-1.91593	0.204274	-1.59249	1.428391	0.595948	-1.31445	-0.72591	-2.10774	0.535402	2.117367	-0.19463	-0.29231	-0.87907	0.159087	-0.75094	-0.19527	-0.78639	0.005359	0.345255	0.102419
41	佐賀市	Saga-shi	-0.63119	1.768632	-1.47627	0.282759	-1.16036	0.621655	-3.29883	0.802186	-1.69732	0.186709	0.192299	-0.84162	1.024694	0.062203	-0.18503	0.14235	-0.26686	-0.63047	1.114458	0.022642	0.389713	0.67734	-0.63608	-2.02605	-0.15286	0.563648
42	長崎市	Nagasaki-shi	-2.06579	1.256958	-1.19487	-1.17816	-1.16112	4.509611	-1.50282	1.409327	-0.08521	-0.36066	-0.19338	-0.51359	-0.51723	-0.40796	1.039764	-1.45572	1.780888	1.228819	1.083965	1.154371	-1.06322	0.053588	0.150452	-0.36741	0.894474	0.278185
43	熊本市	Kumamoto-shi	-4.38551	3.405004	-1.43166	-0.4469	3.113494	1.231777	0.063222	1.423769	0.824303	2.791951	0.924142	-0.55379	1.26375	-0.95529	-0.37214	1.677987	0.999201	0.894467	-0.82195	0.491947	0.865997	1.670225	0.690474	0.380396	-0.02241	-0.95697
44	大分市	Oita-shi	-1.2217	3.571821	-0.98893	0.28105	0.672339	3.148473	-2.15934	2.15796	-0.07534	0.511352	1.187157	1.016892	0.799171	-0.31614	0.074056	-1.9247	-1.11477	0.267621	-0.25608	-1.41092	0.35487	-0.36914	-0.62087	-0.43827	1.104757	1.113421
45	宮崎市	Miyazaki-shi	-3.59936	4.657084	0.05167	-2.08208	-0.30964	-0.82575	-3.38234	1.204693	-0.68713	-0.61489	-2.56474	-0.6427	0.900981	-0.0818	-0.46299	-0.50768	-0.23849	-1.01683	-1.12762	0.790151	1.557148	0.488766	-0.5451	0.684452	-0.36105	-0.48135
46	鹿児島市	Kagoshima-shi	-3.67869	3.766802	0.063045	-1.00513	1.161415	0.903721	-1.56827	3.253499	-2.01854	-1.25407	0.383771	0.809469	-0.32866	1.503964	-0.10569	0.564769	-1.39287	-0.00841	0.211729	-0.38838	-0.47304	-0.843	1.454785	0.977302	0.405431	-0.50775
47	那覇市	Naha-shi	-8.73891	-3.18197	-1.14746	-0.97364	5.735779	-5.22307	1.502214	0.969691	-2.58079	1.519456	-0.13308	0.278431	-1.32609	0.666403	0.822105	-1.39358	0.535076	-1.59301	0.634205	-0.49504	0.53676	-0.87455	1.042563	-1.45507	0.311744	0.359752
48	川崎市	Kawasaki-shi	-0.40749	-4.46806	2.402381	0.140636	-1.25554	-2.2394	-1.20439	2.0004	-1.47146	-0.24586	-0.49534	-0.38991	0.355862	2.051024	1.576382	1.240841	0.183622	2.328146	2.436443	1.04045	-0.22081	0.586001	0.175507	1.022148	0.297782	0.099963
49	相模原市	Sagamihara-sh	2.021401	-3.84096	1.599621	0.719186	-1.40712	0.676283	-0.2717	2.359324	-0.09475	0.755267	-0.83288	0.33109	-0.37637	0.818717	-0.59711	1.446678	-1.12512	-0.17342	0.063951	-1.46235	-0.39063	0.427188	1.162217	0.564594	-0.31419	-0.72183
50	浜松市	Hamamatsu-sh	-1.80082	-0.37176	1.439662	-2.30182	0.600995	1.694959	0.449561	-1.01654	1.514553	0.142091	0.338427	0.066135	-1.43446	1.219559	-0.27048	-0.1251	0.368035	0.414047	-1.14364	0.655022	1.649731	0.480308	-0.37815	-0.03335	0.286567	0.1908
51	堺市	Sakai-shi	-2.77187	-0.67406	-0.19473	2.954869	-0.96169	-0.99183	-0.77802	-1.75916	0.718958	-0.12174	1.58193	0.080973	0.280501	0.10875	0.178545	-0.12987	-0.05514	1.421881	1.561827	0.456515	0.810066	0.414197	0.50438	0.352064	0.464765	0.291419
52	北九州市	Kitakyushu-sh	-1.91875	1.76283	-2.59792	-1.54741	-2.93053	-0.66784	-2.46771	-0.40839	-0.14877	0.010654	-1.5355	-0.966	-0.83567	-1.71488	0.593549	2.057243	1.760028	0.200306	-0.14371	-0.10161	-0.46083	-0.79491	-0.14238	0.911214	-0.39115	0.590102

Factor27	Factor28	Factor29	Factor30	Factor31	Factor32	Factor33	Factor34	Factor35	Factor36	Factor37	Factor38	Factor39	Factor40	Factor41	Factor42	Factor43	Factor44	Factor45	Factor46	Factor47	Factor48	Factor49	Factor50	Factor51	Factor52
-2.70126	-2.03124	-1.66738	-1.14793	-0.89292	-0.48187	-0.23725	-0.12044	-0.1042	0.090719	0.017776	-0.08374	0.098949	-0.03604	-0.03424	-0.14207	0.056188	-0.01134	0.069563	0.103429	0.125362	-0.23996	-0.07823	-0.14087	0.153703	0.172883
0.102137	0.839639	0.409486	-2.07944	1.288996	-0.04076	-0.4679	0.26846	-0.39058	-0.02133	-0.09966	0.650549	-0.18961	0.780353	0.198054	0.088035	-0.23531	0.106305	-0.04953	0.572793	0.302723	-0.35271	-0.0389	0.030273	-0.13468	0.050886
-0.41899	0.645067	-0.66937	1.132956	-1.03697	0.191478	-0.98604	0.489425	0.569207	-0.53436	-0.239	0.181272	-0.89689	0.775877	-0.91601	0.398054	-0.24417	-0.74876	0.246727	-0.3056	-0.10198	0.401646	0.071554	0.309014	-0.04094	-0.49875
-0.57696	-0.96828	-0.54148	1.561553	-0.41311	-0.78072	1.131873	0.557515	-0.22143	0.162364	-1.56757	1.430904	0.529196	-0.56886	0.404347	-0.47439	0.708718	0.608081	-0.27145	0.873045	-0.08376	-0.57626	0.434794	-0.57867	-0.57361	0.096796
-0.33553	-0.7661	1.7127	0.612902	-1.49985	0.103494	0.103129	-0.82162	-0.26191	0.02678	0.26583	-0.78284	-0.54529	0.260851	-1.61145	-0.03666	-0.62693	0.901156	-1.28574	0.447998	0.071442	0.566519	0.001621	0.013283	0.129439	-0.60068
0.828155	1.273132	0.069893	-0.20693	-0.83261	0.35733	0.56214	-1.13297	-0.43171	0.913147	0.565611	-1.06132	0.727783	-0.55634	1.780235	-0.53054	0.95107	0.037102	-0.62983	0.436521	0.087547	-0.01925	0.302294	0.816204	-0.23276	0.167992
-0.56078	0.430072	0.858903	-1.07411	0.874249	1.193429	1.344413	0.405079	-0.48317	1.418485	-0.58734	0.399851	-0.22945	-0.87398	0.600905	0.026739	-0.66491	-0.43562	0.908924	-1.01198	-0.00833	0.007308	0.214821	-0.49126	0.242009	1.861764
-0.88844	0.114985	0.561526	0.162833	-0.0108	0.4132	0.033254	0.452742	-0.08138	-0.33994	0.016716	0.012976	-0.54756	0.00897	-0.95794	0.515577	0.094909	-0.39631	0.259956	-0.82083	0.624494	0.59741	0.199555	0.34	-0.16861	-0.33035
-0.26639	-0.43359	0.422153	-0.82793	0.298047	0.073257	0.350215	-0.11839	-0.23737	-0.13605	1.269096	-1.99912	-0.46666	-0.07507	-1.12388	0.433038	-0.294	-0.67827	0.113562	-0.33299	0.757301	0.214251	-0.03484	-0.61944	0.910522	0.296214
0.557609	-0.2356	0.384487	-0.95177	0.497591	-0.82684	0.179027	-0.65856	-0.19686	-0.6537	-0.70183	2.005404	0.865998	1.010205	0.450877	1.250981	0.862188	0.634537	-0.22589	-0.38899	0.558837	1.42163	-1.7755	-0.44305	0.106154	-0.45595
0.427424	0.869288	0.178686	-0.55995	0.820163	-0.02776	0.998184	0.044065	0.332621	0.131079	0.684582	1.344622	-0.67491	0.427427	-0.86273	-0.74222	-0.16423	1.183681	0.544898	0.977465	-0.65212	-0.84357	0.425547	-0.6201	-0.49666	1.005758
0.430556	0.81373	0.734328	0.27998	-0.13001	0.375166	0.783661	0.045216	-1.98733	0.044616	-0.12843	-1.05254	-0.00134	-0.43221	0.403292	1.805837	-0.76872	-0.35631	0.567573	0.078162	-0.72267	-1.5915	-0.11329	-0.28819	0.197097	-1.07888
0.766661	-0.29462	-1.10726	0.033921	0.588442	-0.63848	-1.41735	-0.09327	-0.99866	-2.23659	-0.4244	-0.07364	-0.16968	-0.3319	0.68264	0.013454	-1.27064	1.032781	1.449245	0.575272	0.410749	1.309665	1.051694	1.217055	0.533417	1.054605
0.352298	-0.61173	-0.88781	-0.9756	-1.37756	-0.51976	-0.0651	1.367424	0.813226	0.422838	0.28146	-0.16428	0.088021	0.128805	-0.23946	0.11638	0.158624	-0.68118	0.050195	0.000172	-0.59367	-0.05188	0.623591	-0.22308	-0.08429	0.718471
-0.26887	0.793863	0.510526	1.66418	1.106972	0.349568	0.058737	1.040759	1.248051	1.964223	0.812073	0.941784	1.986217	0.577805	-0.78093	0.304309	0.32204	-1.56346	-0.10516	1.226892	0.202827	0.333365	-0.19482	0.71119	0.278415	0.026407
0.711059	-1.53619	-0.55716	-0.54618	2.31006	0.068912	-1.11291	0.313014	1.734669	-0.85198	1.024626	-0.64596	-0.02536	-0.43724	-0.33656	-0.05947	-0.02065	-0.52421	0.149131	-0.0064	0.248976	-0.90541	-0.20441	-0.03256	0.243613	-0.68042
-0.07016	1.040353	0.315628	-0.55053	-0.13654	-0.02486	-2.44885	0.207111	0.077466	1.139396	-0.39466	0.505104	0.543153	-0.2615	0.703677	-0.61498	-1.18368	-0.92078	-0.1259	0.009016	-0.48752	1.177212	0.04611	-0.98026	-0.70047	-0.00107
0.036356	-0.02274	0.052891	0.333683	-0.57501	0.318096	0.636361	-0.46402	-0.36619	-1.65175	-0.24543	0.117648	0.731757	-1.08638	-0.72125	0.390842	1.048806	0.761537	0.293689	0.436882	0.344453	-1.29101	-0.07994	1.31929	-0.66792	0.910519
0.765388	-1.26603	-0.15089	0.876134	-0.25851	-2.95428	1.113498	-0.51938	0.319228	0.854247	-1.1069	-0.6862	0.634713	0.775327	0.3603	-0.28578	-0.21178	-0.29814	0.681376	0.142714	0.041579	-0.28041	-0.01903	0.3854	1.443024	-0.4409
0.383094	-0.23241	-0.76408	-0.54174	1.558841	-0.20562	1.018027	-0.70134	-0.31945	0.018589	0.180551	-0.95869	0.103681	-0.00937	0.778509	-0.18521	-0.64836	-0.13859	-1.89379	0.805886	-0.09806	-0.04051	1.586684	-0.26053	-0.8134	-1.08248
0.950334	-0.26153	-0.56839	-0.27512	-0.8219	0.074579	-0.8243	0.169488	-0.73789	0.485979	0.399317	-0.23165	0.313027	0.547359	0.924249	-0.04588	0.018672	1.028367	0.126658	-0.4104	0.122724	0.682019	-0.76584	0.296929	0.47684	-0.6952
-1.02513	-0.33946	0.584673	-0.12145	-1.22311	1.24782	-2.22322	0.451081	-0.936	-0.09664	1.136973	0.082636	0.459658	-0.09003	0.802931	-0.30975	0.970322	-0.11342	0.173626	-0.00644	-0.97065	-0.06919	0.05571	1.382342	0.044995	-0.23779
-0.83315	1.146544	0.190409	0.22294	-0.39236	-0.65176	1.341938	-0.3542	1.23002	-0.60025	0.925726	-0.08558	-0.31739	-1.94066	0.552418	0.15036	0.899748	0.496825	-0.30886	-0.53081	-0.45137	0.853033	0.460617	0.112005	-0.18568	0.058367
0.72327	0.914532	0.937648	1.965966	-0.35791	0.461418	-0.46126	0.200089	0.345124	0.921095	-0.57941	-0.45112	-1.8767	-1.19436	-0.62921	-0.56338	-0.46273	0.359979	-0.1301	1.049836	0.955768	0.675089	-0.51692	-0.05031	-0.01754	0.264413
0.722608	-0.18525	-1.90842	0.855539	0.564616	1.010113	-0.71039	0.153286	-0.01506	1.429232	0.703552	1.273055	-1.01462	0.137311	0.055747	1.127761	0.01075	1.187382	0.559766	-0.0325	-1.1677	-0.52119	-0.72792	0.124006	0.050109	-0.49463
-0.49257	-0.34832	0.788461	0.127045	0.815453	-1.00047	1.073119	-0.45555	-0.19466	-0.65107	-0.40906	1.421014	-0.89835	-1.42597	-0.2597	0.547685	-0.89031	-1.70263	0.244081	-0.97119	-0.05892	-0.26748	0.005337	0.99558	-0.18547	-0.70042
-0.12844	-0.51682	1.216569	-0.42605	0.830259	0.232303	-0.63233	-0.30983	0.536933	-0.08133	-1.35508	-1.07277	-0.59349	0.054564	0.479089	-0.60898	0.21241	0.643549	-0.00696	0.921351	-0.03731	-0.12833	-1.32259	-0.43762	-0.3126	-0.0882
-0.74291	-0.80066	0.580459	1.416021	1.68047	0.457069	0.560435	1.083198	0.186876	0.408402	0.719463	-1.32015	0.648962	1.693163	1.249664	0.92484	0.354077	1.322447	-0.00307	-1.07798	1.011768	0.329283	-0.02708	-0.11797	-1.3019	0.623325
0.487161	-0.99624	-1.30374	-0.58653	-0.53499	1.860573	2.395796	0.873884	0.020828	0.561942	-0.94787	0.66818	-1.38056	-0.47043	0.860291	-0.058	0.316312	-0.72363	-0.93552	-0.0279	-0.27451	0.816125	-0.60068	0.356223	0.769139	-0.00709
1.655682	0.916544	-0.48249	-1.16879	-1.42689	-1.18119	-0.17859	0.731589	0.283486	1.150775	-0.24063	-0.00227	0.217549	-0.28287	-1.27299	-0.44897	0.425821	0.804995	0.161297	-1.98779	0.68566	-0.44805	0.722199	0.011053	-1.0973	-0.12615
-0.25172	0.412769	1.200198	0.065757	0.523032	-0.26526	-0.03801	1.468419	-1.65775	-0.72325	0.15144	-1.32131	1.514347	-1.55659	-0.48604	-0.6701	0.164646	0.21348	0.293565	0.344836	-0.60944	0.230408	-0.62217	-0.73077	1.037562	0.187132
-0.07626	0.148053	0.908931	-0.14547	-0.03604	-0.67735	1.393622	-0.05429	-0.91236	1.34436	-0.04375	-0.02546	-0.35496	0.901067	-0.11457	0.534272	0.21083	0.459559	-0.48998	0.133347	-0.64889	0.020223	0.930413	-0.22142	0.245551	0.199003
0.369924	-1.80345	1.252255	-0.12211	-0.40627	0.790512	0.888607	-0.85704	1.014292	-1.82997	0.884105	0.792077	0.055064	-1.11021	0.936555	-0.68314	0.306343	-0.13451	0.407883	0.074901	-0.04733	0.068408	-0.9742	-0.85611	-0.54034	-0.37683
-0.91576	0.701304	1.612616	-0.51713	0.157872	-0.8669	-1.63442	-0.36945	2.321617	-0.38759	-0.55539	0.499496	-0.31581	0.32584	0.098689	0.45834	-0.04299	0.712998	-0.15819	0.222861	-0.33602	-0.72252	0.790757	0.220228	0.298538	0.2079
-1.08704	1.632891	-1.11828	0.456463	0.754509	-1.31817	-0.0321	-0.42831	-1.25004	-0.09374	1.205006	1.27529	-0.03378	0.03898	0.849303	-1.33145	-0.62384	-0.4688	-0.02073	-0.27751	0.153077	-0.19185	-0.80371	-0.15536	-0.11551	-0.72859
0.14514	0.043753	-2.17666	0.257659	0.895623	0.873099	0.587152	0.782192	1.272999	-0.35158	-1.28594	-1.36019	1.085969	-1.04381	-1.13269	-0.28	0.04185	0.66633	0.709674	-0.30499	-1.08795	0.617969	-0.00094	-0.42734	0.098151	-0.78283
1.050722	-0.45867	0.767897	0.773944	0.549987	1.07571	-0.62832	-0.33991	0.250048	-1.53868	-0.22096	0.15561	0.535445	0.475204	0.121344	-0.48567	-0.92917	-0.33641	-1.30667	-1.57164	-0.97472	-0.01573	1.007242	0.562116	-0.57958	0.046592
0.344723	1.010563	-1.22952	-1.32243	0.571043	-0.09839	0.553096	-1.38783	0.460761	0.404113	1.240568	-0.80115	-0.6221	0.498551	-0.84402	-0.02542	1.390908	-0.71701	0.379237	0.995392	0.002459	0.606226	-0.04421	0.705882	0.202012	-0.0157
1.335616	0.361533	0.232163	0.627177	-1.10688	-0.50264	-0.73027	0.037223	0.102848	-1.85564	0.004516	0.085319	0.076327	0.209313	0.73862	1.239058	0.931588	-2.14506	0.268651	0.778637	-0.17329	0.195293	0.610108	-1.39198	-0.25367	0.934545
0.732224	-1.58571	0.876456	-0.50097	-0.03048	0.525781	-1.52824	1.004821	-1.05819	0.248242	-0.07468	0.766775	-0.50659	0.929533	-0.67426	-0.66285	0.140623	-0.08646	-0.124	0.886101	0.09777	-0.75242	-0.28953	0.228297	0.074051	-0.18079
-0.4665	0.360082	0.96596	0.081156	0.145338	0.675537	0.947244	-1.63065	-0.8794	-0.33107	-0.24553	0.375457	0.280688	1.371626	-1.77247	-0.33849	0.506002	0.349395	1.595613	-0.09543	-0.81172	0.948297	0.609928	-0.49201	-0.64069	-0.83452
-0.11308	0.554453	0.500741	0.332473	-0.96654	-0.49221	2.024759	1.159049	1.145514	-1.29797	1.353047	-0.10242	-0.57437	2.159255	0.978998	-0.44494	-1.19011	0.666279	-0.09134	-0.05734	-0.67061	-0.03243	-0.28328	0.314541	1.103413	0.527871
-1.04331	0.890413	-0.672	-0.26943	0.275568	0.229901	-0.8797	1.201862	-0.46713	-0.89803	-1.40527	-0.39441	-1.18527	0.541402	0.839773	0.356772	1.188774	0.099588	-0.34989	0.506515	0.794579	-0.00211	1.723113	-0.39789	0.105016	-0.88456
0.376798	-0.04667	-0.53486	0.488754	0.090086	-1.14114	0.069007	1.407132	-0.97019	-0.51996	1.606514	0.374649	-0.6261	-1.0592	-0.71642	-0.14663	0.20555	0.073478	-0.51841	-0.20002	1.001466	-0.13531	-0.98768	-0.35124	-0.77667	-0.05711
-0.08325	-1.21358	-1.33203	0.699248	1.182458	-0.07208	-0.5095	-1.60412	-1.07184	0.619555	-0.36914	0.106829	0.172194	0.055074	-1.08702	0.220668	-0.24435	-0.40588	-1.17238	-0.01075	-0.04506	0.290809	0.01263	0.432768	-0.08106	1.917676
0.315648	-0.64396	1.56161	0.398044	0.587754	0.263554	-0.8477	-0.94471	0.557885	2.066959	-0.35878	-0.07782	-0.55636	-0.76832	1.220285	-0.49649	0.850771	-0.13784	1.128955	-0.98809	0.646097	-0.37951	0.566785	0.307431	0.91402	-0.30165
0.009587	-0.3626	0.891511	-1.57191	-0.92456	0.167535	1.258633	1.095998	0.343935	0.446627	-0.77461	0.193226	1.230488	-0.15403	-0.33948	0.078964	-1.16121	-0.39881	0.129269	0.817761	0.671952	0.268096	0.04407	1.317278	-0.96372	-0.58687
-0.42159	1.12815	-0.89656	0.693091	0.381896	-0.62043	-0.82036	-0.18382	0.136426	0.077901	-0.07613	-0.0022	-0.00835	-0.77758	0.244987	-0.11454	-0.1706	0.444411	-0.11229	-0.31391	-0.10954	-0.23647	0.249148	-0.03614	0.329105	-0.15801
-0.16335	-1.576	0.045211	0.219324	-0.66572	-1.33191	-1.06516	-0.26758	-0.31323	1.053249	0.071321	-1.02482	-0.61301	0.601809	0.011161	-0.28669	0.51622	-0.72051	-0.06689	-0.73997	-1.11918	-0.02614	-0.459	-0.38204	-0.8705	0.545287
0.144871	1.01394	-0.14446	0.068825	0.074717	1.073224	-0.10399	-0.16134	-0.32311	-1.18859	-0.70554	0.887647	0.740714	0.963341	-0.96149	-0.88458	0.93926	-0.23939	-1.26903	-1.267	0.653058	-0.77434	0.068529	-0.42808	2.006852	0.188548
-0.34036	1.394546	-1.34281	0.43335	-0.82372	0.932036	0.508097	-0.92342	0.37943	-0.63159	-1.17955	-1.44473	-0.02007	1.676561	0.49551	-0.64707	-0.62694	-1.11153	0.782109	-0.13896	0.639713	-1.02278	-1.7883	0.08756	-1.07607	0.266387
-0.05999	-0.97421	-1.09113	0.394122	-1.72551	1.43552	-0.09081	-1.24669	0.320092	0.721008	1.248703	0.931383	1.0133	-0.26736	0.229825	0.266859	-1.27318	0.421828	0.611477	0.389506	1.338359	-0.34904	1.613266	-1.25569	0.47047	-0.32049
-0.39326	1.061449	-0.1782	-0.74553	-0.81728	-0.10934	-1.23997	-0.8614	0.863503	0.376731	-0.44601	-0.25239	0.591438	-1.11525	-0.17746	1.355527	-0.85518	0.978786	-1.25511	0.0701	-0.20842	-0.3629	-1.67512	-0.18595	0.448405	0.417565

Coefficients

	sio1.1.fact	sio1.2.fact	sio1.3.fact	sio1.4.fact	sio1.5.fact	sio1.6.fact	sio1.7.fact	sio1.8.fact	sio1.9.fact	sio1.10.fac	sio1.11.fac	sio1.12.fac	sio1.13.fac	sio1.14.fac	sio1.15.fac	sio1.16.fac	sio1.17.fac	sio1.18.fac	sio1.19.fac	sio1.20.fac	sio1.21.fac	sio1.22.fac	sio1.23.fac	sio1.24.fac	sio1.25.fac	sio1.26.fac	sio1.27.fac	sio1.28.fac	sio1.29.fac	sio1.30.fac	sio1.31.fac	sio1.32.fac	sio1.33.fac	sio1.34.fac	sio1.35.fac	sio1.36.fac	sio1.37.fac	sio1.38.fac	sio1.39.fac
intake11	5.715504	-3.20424	-18.8843	-17.6791	-17.0153	-19.2274	-21.1705	-20.2843	-13.9261	-13.2695	-13.4058	-12.8486	-12.2878	-11.5542	-11.5026	-10.9964	-10.6312	-10.2982	-10.1334	-10.1636	-10.1159	-10.2421	-10.3345	-10.453	-10.525	-10.7566	-11.193	-11.3364	-11.4385	-11.4879	-11.4984	-11.507	-11.501	-11.4933	-11.4916	-11.4918	-11.4924	-11.4918	-11.4918
intake12	1.149201	10.05949	30.50791	44.54114	43.96305	48.5006	53.21314	54.84635	55.8009	56.20688	55.58014	55.17183	52.86706	50.93767	48.39766	47.40614	47.06333	47.12862	47.24443	47.30945	47.40334	47.40989	47.38383	47.3501	47.34684	47.34681	47.29941	47.2519	47.23171	47.21333	47.19553	47.18288	47.17897	47.1825	47.18339	47.18324	47.18301	47.18232	47.18217
intake13	9.040226	27.03129	52.24545	61.76558	59.92473	62.40604	68.34622	70.68944	74.33092	74.4443	76.28197	77.39975	79.58905	80.47173	80.84326	81.37446	82.02151	82.3297	82.69431	82.76402	82.80133	82.87898	83.02274	83.13974	83.24513	83.41877	83.82161	84.01146	84.11023	84.18558	84.24866	84.27225	84.28025	84.27597	84.27525	84.27572	84.27697	84.27756	84.27746
intake14	27.54922	43.33575	64.71369	67.35852	70.73744	73.927	77.11893	77.37149	74.68017	73.43986	74.90147	73.63284	71.82556	70.77298	69.47924	68.9657	68.72404	68.56848	68.36442	68.18917	68.15846	68.18238	68.15755	68.08051	68.00846	67.84696	67.61889	67.47096	67.36996	67.23849	67.18956	67.16057	67.14723	67.14332	67.1458	67.14516	67.14518	67.14526	67.14508
intake15	10.50194	3.825155	14.20318	17.62146	16.65762	13.70008	14.19245	17.35327	18.35043	20.35471	21.16338	20.82406	19.52111	18.34114	15.26323	13.81496	12.95393	12.79341	12.44498	12.34191	12.22352	12.24733	12.30433	12.36815	12.40413	12.43314	12.52152	12.56819	12.60808	12.63812	12.6289	12.62276	12.62089	12.62165	12.61943	12.61926	12.61833	12.61864	12.61834
intake16	11.71342	2.17448	8.31279	4.128573	-4.24907	-16.5082	-18.1088	-18.8233	-20.0685	-20.3573	-19.9747	-19.4781	-18.5178	-18.235	-18.5431	-19.4575	-20.1126	-20.3652	-20.3687	-20.3562	-20.3678	-20.4108	-20.4503	-20.5667	-20.7114	-20.983	-21.4747	-21.6606	-21.7195	-21.74	-21.7697	-21.7769	-21.7774	-21.786	-21.7916	-21.7945	-21.7952	-21.7953	-21.7952
intake17	7.652349	30.70138	64.31241	65.18688	52.08508	35.40705	27.66735	24.54753	21.91814	19.74259	18.70839	17.65601	17.19133	17.45443	17.77253	17.09025	17.21107	17.42502	17.44398	17.60488	17.71425	17.65518	17.51106	17.36265	17.23026	17.10093	17.07979	17.0237	17.00662	17.04343	17.05001	17.04619	17.04459	17.03535	17.03121	17.0322	17.03104	17.03012	17.02974
intake18	-3.17147	-13.2968	-0.31519	5.283642	5.440613	10.25545	13.33534	14.49513	16.07919	14.48422	11.83379	10.67655	9.050587	7.503863	5.378979	4.512299	4.65009	4.710941	4.736628	4.768027	4.698511	4.639174	4.589542	4.529909	4.512264	4.329045	4.020862	3.870665	3.778472	3.637428	3.561813	3.540445	3.518688	3.507882	3.503181	3.502235	3.501636	3.502379	3.502542
intake19	3.299761	-5.6027	13.88557	18.09672	26.29015	28.14966	25.60425	22.75111	22.92551	22.08207	21.36561	22.41058	24.10106	25.90717	26.97354	27.56665	27.6169	27.63141	27.72465	27.77173	27.81953	27.79221	27.71113	27.58249	27.36691	27.02126	26.60576	26.44416	26.34779	26.29274	26.2689	26.25587	26.25069	26.24961	26.24905	26.25051	26.25205	26.25218	26.25212
intake110	-2.06459	7.867924	-1.5276	-4.41025	-9.13506	-6.39212	-10.1841	-10.6177	-13.2016	-14.5409	-16.8549	-17.5035	-17.9994	-18.4292	-18.8354	-19.6156	-19.7961	-19.9213	-19.9934	-20.0778	-20.2123	-20.2733	-20.4073	-20.5527	-20.72	-20.9447	-21.301	-21.4328	-21.5173	-21.5531	-21.5569	-21.5549	-21.5551	-21.5558	-21.5571	-21.5607	-21.5633	-21.5642	-21.5646
intake111	17.20509	31.14004	22.36799	26.57897	26.71537	31.62517	31.37961	31.67948	29.74306	29.79511	28.95179	29.28095	28.44733	28.67894	29.67741	29.532	29.4792	29.58443	29.60666	29.6271	29.67064	29.69389	29.70389	29.7218	29.65725	29.64286	29.63475	29.62169	29.61483	29.64087	29.64385	29.633	29.62968	29.62903	29.62944	29.63039	29.63119	29.63141	29.63127
intake112	0.312115	3.919327	6.849967	8.117949	10.21213	9.782121	6.937457	2.01646	1.209989	0.216178	0.743785	0.694116	0.345996	1.117771	0.727427	0.464291	0.508043	0.07748	0.030412	-0.02373	-0.06931	-0.08774	-0.09718	-0.02054	0.044542	0.325016	0.691736	0.784712	0.907044	0.998451	1.040211	1.077862	1.080696	1.083366	1.078983	1.076668	1.075346	1.074526	1.074002
intake113	21.40497	24.61261	3.906735	5.294594	1.606613	-3.39553	-2.27222	-1.02925	1.595646	4.453028	5.648202	5.823253	4.884429	4.567191	4.356749	3.745172	3.465847	3.544665	3.465897	3.533787	3.638572	3.720068	3.839797	3.976227	4.120067	4.355168	4.643575	4.75404	4.80676	4.794102	4.780843	4.765862	4.768602	4.767839	4.770128	4.771604	4.772045	4.772002	4.772048
intake114	23.35808	24.85941	35.03212	35.29721	34.69577	29.60179	29.80471	32.74853	36.1857	36.11846	36.4958	37.51773	38.15411	38.66324	39.55266	39.84675	39.90243	39.93677	40.0648	40.1712	40.17908	40.18294	40.18359	40.16322	40.22966	40.40328	40.81158	41.02163	41.09128	41.10748	41.12182	41.12011	41.11801	41.11679	41.11374	41.11109	41.11103	41.11161	41.11179
intake115	-1.88161	7.943478	3.030196	9.525568	14.40314	23.38143	26.21942	32.53604	35.72989	38.26949	41.34711	44.42107	46.96682	49.21816	51.69808	52.57174	52.95877	53.12144	53.20381	53.26925	53.32749	53.35672	53.35887	53.38076	53.40647	53.41856	53.32532	53.28328	53.25067	53.17524	53.15946	53.16512	53.16986	53.16868	53.16891	53.16735	53.16553	53.16512	53.16551
intake116	23.44684	23.63306	19.9886	15.98172	10.08721	-0.84528	-2.99641	-3.39421	-4.043	-5.83699	-4.65591	-4.40871	-4.77418	-4.83446	-5.12038	-5.07634	-5.08982	-5.38764	-5.61005	-5.57056	-5.47631	-5.43848	-5.44432	-5.42598	-5.35799	-5.21165	-5.10166	-5.0724	-5.05068	-5.02285	-5.01803	-5.02633	-5.02412	-5.02388	-5.02556	-5.02581	-5.02557	-5.02499	-5.02503
intake117	-9.24709	0.761167	-3.31957	0.989228	-0.41213	-1.28892	-4.4096	-2.67483	-0.73636	1.447647	3.784207	4.414627	5.799281	6.351851	7.838981	8.543976	8.902665	8.950005	9.206736	9.340806	9.490032	9.602948	9.67164	9.740222	9.792473	9.854502	9.916139	9.956021	10.01403	10.09936	10.13499	10.15606	10.16957	10.17732	10.17902	10.17853	10.17978	10.18035	10.18032
intake118	2.80763	11.34616	5.083744	8.559603	6.574844	11.94644	10.83652	6.566883	1.881599	-0.55046	-3.63182	-5.26984	-7.94103	-8.99242	-10.0761	-10.4274	-10.6007	-10.8367	-10.7728	-10.7775	-10.8287	-10.7781	-10.7544	-10.7531	-10.6591	-10.6503	-10.6155	-10.6582	-10.6747	-10.6911	-10.7027	-10.7203	-10.7336	-10.7424	-10.7474	-10.7492	-10.7499	-10.7495	-10.7492
intake119	27.22603	38.31705	29.69528	34.6918	36.15903	33.66693	28.54888	21.64047	17.82807	15.93502	13.3198	13.53272	14.20027	14.89728	15.74841	15.62777	15.53271	15.67784	15.76258	15.78268	15.75047	15.77141	15.79567	15.81751	15.87512	15.98938	16.09579	16.12818	16.15166	16.14978	16.13934	16.13113	16.12437	16.12203	16.12248	16.12364	16.12478	16.12512	16.1254
intake120	0.621701	-11.673	-21.6108	-10.3219	-3.27446	-0.20972	3.975164	7.089824	13.44383	12.55154	12.9578	13.05169	14.16955	14.78786	15.30907	15.79212	15.84761	15.75644	15.42446	15.28587	15.21776	15.14469	15.08015	14.99345	14.79284	14.52355	14.15716	13.94869	13.80121	13.69726	13.68143	13.65628	13.65233	13.65589	13.65972	13.66161	13.6618	13.6616	13.66175
intake121	-2.9091	-2.75945	-18.4603	-15.8633	-24.2266	-28.4123	-30.9247	-31.8597	-29.9025	-30.3645	-31.3238	-32.0894	-33.019	-32.873	-32.315	-31.8294	-31.8827	-31.8149	-31.6248	-31.5491	-31.464	-31.429	-31.5103	-31.604	-31.7593	-32.0047	-32.2068	-32.2621	-32.3401	-32.4477	-32.4795	-32.4743	-32.4755	-32.4752	-32.4765	-32.4771	-32.4775	-32.4772	-32.4768
intake122	17.6993	27.50714	20.41341	23.06632	18.94817	13.35559	7.241216	-0.52706	-6.6867	-7.16016	-8.7218	-8.99931	-10.6141	-11.3359	-11.606	-11.9881	-12.1093	-11.9582	-12.0702	-12.0833	-11.9936	-11.9479	-11.7965	-11.6801	-11.5009	-11.2372	-10.8876	-10.7734	-10.6708	-10.5868	-10.5608	-10.5571	-10.5502	-10.5439	-10.5381	-10.5375	-10.5372	-10.5375	-10.5377
intake123	2.351924	5.532136	13.40941	7.534163	6.85126	11.06038	4.933672	3.345121	2.954781	4.159143	4.173551	3.862823	3.063096	2.844161	1.62409	0.928401	0.797509	0.977594	0.951839	0.937876	0.970718	0.973964	1.025747	1.045475	1.052412	1.038726	0.939582	0.868521	0.798204	0.745449	0.744156	0.736842	0.73122	0.731087	0.73134	0.731596	0.733266	0.733623	0.733643
intake124	14.59005	7.848273	-7.13721	-11.5072	-11.1277	-7.53319	-0.68799	4.691779	6.44483	6.936964	6.661151	8.234863	9.325055	10.14609	11.18326	11.19893	11.28041	11.27451	11.07064	10.90635	10.76682	10.72722	10.66686	10.62169	10.60225	10.68017	10.71942	10.66993	10.68488	10.67366	10.65809	10.65867	10.6545	10.64696	10.64495	10.64398	10.64644	10.64683	10.64678
intake125	-0.38778	2.203652	10.87214	13.28184	9.990108	14.10538	16.91731	14.25367	10.43118	7.602103	5.642908	4.242776	3.207028	2.926029	2.499227	1.707369	1.362615	1.096841	0.799797	0.654981	0.400276	0.299421	0.287065	0.382193	0.428779	0.486083	0.404657	0.454864	0.46612	0.528551	0.562234	0.556916	0.559574	0.5614	0.555216	0.554422	0.554957	0.555172	0.555095
intake126	27.80753	43.50433	56.61294	68.87864	79.70167	79.5603	76.51168	73.52164	69.98633	66.62187	63.70002	63.74082	64.20701	64.1749	64.80785	65.05368	65.12514	65.29769	65.37191	65.42874	65.58705	65.67053	65.78732	65.90234	65.93796	66.01098	66.07375	66.11796	66.1226	66.15627	66.13792	66.12696	66.11953	66.12104	66.12164	66.12058	66.11951	66.11919	66.11881
intake127	29.2275	30.68003	26.80272	25.37938	26.28824	24.95204	23.42608	19.58901	17.45999	15.92868	15.50843	14.42707	12.85551	12.28136	11.64353	11.38846	10.95419	10.6573	10.41891	10.29984	10.25	10.21844	10.15241	10.09223	10.02931	9.877486	9.673579	9.536138	9.466797	9.450656	9.476967	9.491776	9.496573	9.505102	9.51204	9.5153	9.517058	9.517231	9.517071
intake128	25.34646	30.29736	33.0946	36.39723	40.07801	39.67357	37.4302	38.93327	42.10705	45.53654	47.30537	47.09956	46.49438	45.82909	46.64041	47.56788	47.7876	48.02346	48.24716	48.30883	48.24886	48.21357	48.22673	48.29761	48.31764	48.2857	48.20962	48.20672	48.18968	48.15744	48.15725	48.16154	48.15547	48.14294	48.1361	48.13429	48.13312	48.13235	48.1324
intake129	-0.84797	0.003464	26.65426	33.60911	39.03341	42.32048	38.44797	33.03153	34.03251	33.52949	33.88257	34.56648	33.66698	34.53921	35.37729	35.77647	35.745	35.80678	35.96484	35.9754	36.04911	36.09632	36.09816	36.12487	36.16719	36.3641	36.69849	36.84911	36.96106	37.01524	37.01178	36.99295	36.9873	36.98554	36.9804	36.97813	36.97749	36.9768	36.97692
intake130	6.003957	11.54455	33.38193	29.13046	23.36166	21.6636	15.47157	14.08261	15.84503	16.38442	14.79013	14.65012	12.54837	11.26274	8.846544	7.540394	6.77759	6.376419	5.99026	5.769484	5.675987	5.613646	5.502658	5.393646	5.222779	5.100639	5.018524	4.983122	4.987248	5.020103	5.044445	5.054354	5.056259	5.056934	5.056841	5.056834	5.056057	5.055797	5.056256
intake131	-22.8765	-34.6419	-32.3585	-26.0127	-18.182	-19.879	-23.343	-28.081	-33.6929	-34.655	-33.7647	-33.1029	-33.7914	-32.9268	-32.0119	-32.0871	-31.9847	-32.163	-32.1869	-32.2788	-32.3102	-32.3848	-32.3963	-32.4893	-32.5941	-32.7564	-32.9725	-33.0216	-33.067	-33.0849	-33.0831	-33.0741	-33.0713	-33.0667	-33.0631	-33.0617	-33.0607	-33.0604	-33.0602
intake132	-14.8861	-12.8619	-15.6175	-5.12445	-3.48668	-1.78858	-3.61861	-6.77689	-11.5495	-15.0942	-17.4432	-18.8419	-19.7831	-20.2901	-20.232	-20.3681	-20.4831	-20.5666	-20.4913	-20.4843	-20.4285	-20.3504	-20.269	-20.2466	-20.3131	-20.295	-20.3055	-20.3461	-20.3714	-20.3655	-20.3755	-20.3863	-20.385	-20.3809	-20.3798	-20.3785	-20.3773	-20.3769	-20.3767
intake133	14.42537	7.487196	15.02711	19.49098	21.55839	19.98327	19.56407	20.23852	18.87751	18.8747	17.92353	17.54018	18.51518	19.80878	20.10837	20.16536	19.56852	19.44276	19.30329	19.22532	19.25331	19.24626	19.22304	19.2841	19.32421	19.29213	19.19088	19.14866	19.12801	19.12141	19.12167	19.12674	19.12798	19.12342	19.11979	19.11799	19.11846	19.11815	19.11842
intake134	-6.42101	-3.23062	-8.39977	1.805092	3.977029	6.835162	6.091393	9.010137	9.461406	12.18158	15.11462	16.2776	17.75364	19.04049	20.49721	22.09563	22.94225	23.28028	23.3171	23.26179	23.26433	23.26349	23.19787	23.15918	23.00584	22.7352	22.30105	22.05376	21.90896	21.78989	21.74719	21.73617	21.73866	21.73691	21.73581	21.7366	21.73753	21.73802	21.73802
intake135	-15.7521	-9.91882	-9.29357	0.12737	2.554956	9.94162	13.87788	17.27862	18.60814	20.21318	20.68737	21.08755	21.11277	21.51791	21.74868	22.45765	23.09054	23.34927	23.58685	23.61858	23.64939	23.6878	23.72302	23.75366	23.84727	23.97751	24.16913	24.22847	24.29281	24.35884	24.36157	24.35579	24.35399	24.35219	24.35066	24.35016	24.34976	24.34964	24.34943
intake136	1.276024	-16.7989	-28.11	-39.7133	-56.9641	-68.5236	-73.7663	-77.2044	-76.9353	-78.0281	-79.2696	-78.6009	-76.8237	-76.2523	-75.2618	-74.6403	-74.629	-74.6385	-74.4069	-74.3785	-74.307	-74.2141	-74.0321	-73.9157	-73.7872	-73.7892	-73.9321	-73.9858	-74.0591	-74.1126	-74.1343	-74.1382	-74.1341	-74.1389	-74.1399	-74.1396	-74.1386	-74.1389	-74.139
intake137	18.97253	23.97078	13.03356	15.20859	13.96353	12.38574	13.70657	15.21002	15.88735	14.92645	12.36542	11.8553	12.24714	13.65667	14.39317	14.92566	14.64411	14.4605	14.35473	14.3057	14.15672	14.18236	14.23548	14.29903	14.39446	14.55213	14.84877	14.95224	14.95362	14.99472	14.9786	14.98759	14.99015	14.99063	14.99032	14.9908	14.99054	14.99034	14.99004
intake138	5.446462	-7.05457	-16.2053	-11.9641	-3.93521	-3.0601	-1.50247	2.754489	5.890908	7.419409	6.199535	4.231141	3.188083	3.521369	3.452896	2.852042	2.856922	2.877435	2.875041	2.886336	2.899531	2.947043	2.938356	2.866517	2.811647	2.770647	2.604936	2.639669	2.633275	2.6454	2.661204	2.670251	2.674093	2.675857	2.677869	2.678186	2.677968	2.676968	2.676382
intake139	15.32445	20.89347	45.44688	51.95345	50.8628	57.36523	64.07426	67.58354	67.21355	66.91816	67.51715	68.1514	70.83833	72.91412	74.76877	75.54882	76.1689	76.37558	76.32314	76.37101	76.4291	76.47647	76.48882	76.52921	76.57615	76.5518	76.53587	76.44994	76.40282	76.42018	76.46037	76.4867	76.49179	76.49157	76.49141	76.49134	76.49145	76.49106	76.49109
intake140	-11.2215	-19.3794	-16.0574	-16.9093	-15.345	-19.703	-21.1529	-18.9762	-19.955	-20.2288	-18.8151	-17.6788	-17.9176	-16.9176	-16.3973	-15.8308	-15.96	-15.7811	-15.5942	-15.5136	-15.5339	-15.4247	-15.2251	-15.0732	-14.8995	-14.5716	-14.0935	-13.9312	-13.8626	-13.7916	-13.7127	-13.6726	-13.6628	-13.6564	-13.656	-13.6556	-13.6551	-13.6545	-13.6546
intake141	-13.6498	-32.8645	-25.879	-18.6589	-18.1631	-18.2454	-20.7289	-21.1453	-19.5882	-20.473	-20.8685	-20.792	-20.6776	-21.0632	-21.9997	-23.174	-23.8243	-23.9157	-24.137	-24.1741	-24.2798	-24.3003	-24.4644	-24.6245	-24.8297	-25.1403	-25.6073	-25.8549	-26.0012	-26.1224	-26.1845	-26.2075	-26.2033	-26.1974	-26.1955	-26.1947	-26.194	-26.1931	-26.1933
intake142	-0.51517	-19.4692	-8.53511	-5.89922	-7.28927	-10.2669	-10.1399	-8.10436	-6.94093	-4.9382	-4.1965	-3.5347	-1.39076	0.282283	1.90767	2.997032	3.959846	4.32108	4.432939	4.481137	4.47425	4.491996	4.489573	4.459462	4.456705	4.377448	4.276246	4.275232	4.269055	4.265598	4.252836	4.254483	4.257857	4.265702	4.269113	4.269416	4.267608	4.267108	4.266744
intake143	14.85336	23.91159	8.376067	10.46229	6.106296	3.467278	4.551524	4.323024	4.453327	5.961176	7.716011	7.966791	7.405988	8.032026	7.76181	7.267518	6.797049	6.499568	6.497276	6.427276	6.380808	6.280813	6.157809	5.980273	5.781963	5.508056	5.161331	5.036569	4.969894	4.940514	4.908457	4.902288	4.893017	4.884372	4.8789	4.879083	4.878891	4.878767	4.878714
intake144	11.61686	14.49146	22.24853	22.80841	17.27884	16.24094	11.43631	10.04396	3.077058	-0.57204	-2.69514	-3.54297	-6.28426	-8.3117	-10.6886	-11.7327	-12.6569	-13.054	-13.1732	-13.2886	-13.3911	-13.4466	-13.5241	-13.5431	-13.6155	-13.7589	-13.923	-13.9482	-13.9812	-14.0389	-14.0548	-14.0584	-14.0527	-14.0575	-14.0576	-14.0581	-14.0593	-14.0595	-14.0593
intake145	15.99433	1.40575	-7.8539	-10.9906	-16.3022	-19.2673	-20.2984	-22.18	-21.8983	-19.2029	-16.7198	-15.2382	-12.6845	-10.1956	-6.98686	-5.06344	-3.91765	-3.67711	-3.49084	-3.54913	-3.57834	-3.53923	-3.4976	-3.50619	-3.55414	-3.61647	-3.74996	-3.87118	-3.9648	-4.07005	-4.13585	-4.18211	-4.19669	-4.20711	-4.21061	-4.21087	-4.21155	-4.21164	-4.21162
intake146	-6.66661	-8.9958	-9.77215	-8.47358	-18.7095	-22.4868	-22.0324	-17.3661	-10.5277	-10.3011	-10.8927	-11.7793	-11.9638	-11.3069	-10.7573	-11.0293	-11.3656	-11.6661	-11.7053	-11.6492	-11.485	-11.367	-11.1503	-10.7776	-10.314	-9.65269	-8.9917	-8.75918	-8.62149	-8.54853	-8.53139	-8.50333	-8.49898	-8.49938	-8.49839	-8.49859	-8.49892	-8.49959	-8.49975
intake147	16.80835	3.160365	3.155491	6.53336	11.06059	17.78764	20.2166	20.54818	18.44046	16.73206	15.79929	15.46513	14.94225	14.60683	14.00438	14.37985	14.40221	14.48979	14.59358	14.69573	14.76	14.76487	14.7969	14.82845	14.77882	14.68737	14.53415	14.47288	14.44467	14.41679	14.39906	14.39385	14.39419	14.39453	14.39532	14.39643	14.39682	14.39673	14.39678
intake148	2.707916	-3.63964	15.17724	12.53327	4.648259	0.682596	-1.54908	0.544931	2.514257	5.016912	5.145852	5.104956	3.12552	1.956643	0.885882	-0.02665	-0.99527	-1.30638	-1.36108	-1.41856	-1.44442	-1.49868	-1.50724	-1.5453	-1.67431	-1.8781	-2.03909	-2.12763	-2.22031	-2.30436	-2.32831	-2.34194	-2.34903	-2.34874	-2.35025	-2.35217	-2.35195	-2.35211	-2.35223
intake149	12.82768	4.342282	19.43684	21.656	21.82595	26.14923	29.22765	32.32602	32.45062	34.13286	34.77256	34.62643	33.03537	31.99781	31.1257	30.26212	29.80666	29.80341	30.01289	30.15066	30.2421	30.35218	30.5302	30.74989	31.01281	31.43235	31.90341	32.11565	32.25401	32.34981	32.4041	32.42026	32.42328	32.42548	32.4224	32.42115	32.41905	32.41852	32.41841
intake150	30.86891	34.98143	31.11094	33.04559	35.70671	38.89016	42.21733	44.64486	46.89485	49.66794	50.51746	49.87686	50.41038	50.68916	50.4231	50.39842	50.48671	50.66384	50.92985	51.11354	51.2727	51.38085	51.5158	51.65826	51.83571	52.06303	52.38291	52.53529	52.65591	52.73963	52.74775	52.75062	52.75797	52.76165	52.76444	52.76515	52.76525	52.76542	52.76544
intake151	-1.2808	-9.31765	-18.4946	-22.7446	-26.5428	-25.012	-23.1241	-17.85	-15.5317	-18.3053	-18.0873	-18.8087	-18.7915	-18.0876	-17.525	-17.3069	-16.6322	-16.4689	-16.4074	-16.4854	-16.5825	-16.6055	-16.6636	-16.7724	-16.9902	-17.3829	-17.8017	-17.9632	-17.952	-17.94	-17.9489	-17.9566	-17.9628	-17.9668	-17.9663	-17.9678	-17.9681	-17.9682	-17.968
intake152	-12.4198	-33.1048	-46.8262	-42.3913	-46.0616	-46.8181	-51.0335	-50.6299	-50.0006	-51.5824	-51.9806	-51.5089	-50.5328	-50.1308	-49.9871	-49.876	-49.3143	-48.7119	-48.5364	-48.5618	-48.5246	-48.4702	-48.3871	-48.2646	-48.0495	-47.7138	-47.3348	-47.1938	-47.119	-47.0758	-47.0443	-47.0533	-47.0579	-47.0597	-47.061	-47.0626	-47.0632	-47.0638	-47.0638
intake153	-2.31441	-3.86172	-2.05057	-6.47081	-6.95572	3.38269	5.304616	9.787905	14.48257	16.7151	17.69296	17.82165	19.98925	20.84203	21.41679	22.04754	22.03577	22.03234	21.86421	21.80353	21.75462	21.69772	21.81409	21.93967	22.21512	22.72843	23.37582	23.65314	23.87573	24.06081	24.13889	24.15924	24.16596	24.16663	24.16832	24.17017	24.17103	24.17123	24.17135
intake154	12.06157	-3.58715	-4.09339	-4.7087	-8.36015	-14.7957	-19.5533	-20.8753	-23.3277	-25.6215	-26.7469	-28.0927	-30.1115	-31.0959	-32.2065	-32.467	-32.706	-32.9078	-33.0557	-33.1329	-33.1554	-33.1173	-33.0935	-33.0517	-32.9998	-32.837	-32.644	-32.6005	-32.5765	-32.6296	-32.6523	-32.6726	-32.6742	-32.6731	-32.6723	-32.6709	-32.6716	-32.6719	-32.6718
intake155	4.884467	-7.22017	-3.49138	-2.61492	-1.37797	-0.68526	0.374815	1.205093	-3.17914	-2.51218	-1.3401	-0.472	-0.23168	1.250153	2.249506	2.435546	2.653503	2.746158	2.949666	2.996268	3.024206	2.995561	3.009039	3.01048	3.036955	3.164193	3.258305	3.314734	3.348956	3.400855	3.401591	3.373854	3.371718	3.367884	3.367883	3.365996	3.365181	3.365779	3.365899
intake156	22.15186	31.93762	28.77869	28.9077	33.17835	36.68206	36.95433	40.94854	41.65751	40.96908	40.93607	40.72037	42.27681	42.34217	42.00708	41.73773	41.32682	40.90396	40.71772	40.6313	40.65881	40.63075	40.55341	40.44593	40.32572	40.31079	40.11742	40.0641	40.00927	39.97745	39.9537	39.95719	39.96158	39.96038	39.96034	39.96098	39.96116	39.96205	39.96236
intake157	-3.85064	-23.4403	-43.8046	-37.4513	-38.3312	-41.0253	-43.0417	-40.2881	-40.5956	-41.3466	-40.8384	-40.6753	-41.5674	-42.6018	-43.6357	-43.5913	-43.2754	-43.0156	-42.8498	-42.8629	-42.8353	-42.804	-42.8023	-42.7359	-42.6317	-42.3606	-42.0238	-41.7942	-41.5813	-41.3749	-41.2881	-41.2593	-41.2555	-41.2549	-41.253	-41.2531	-41.2525	-41.2523	-41.2521
intake158	5.365468	14.77039	34.7915	38.35066	33.13099	31.15998	25.30218	20.71814	17.19028	16.51205	16.46459	15.46535	16.63477	17.25833	17.98883	18.7972	18.86719	18.7997	18.73009	18.6691	18.44673	18.26938	18.04232	17.82771	17.72243	17.69922	17.62091	17.56608	17.44494	17.37155	17.33436	17.32279	17.32179	17.32581	17.33219	17.33173	17.333	17.3323	17.33213
intake159	6.567645	2.304867	11.64543	20.17901	26.81407	33.663	30.31047	24.75406	23.23479	23.1317	23.35422	22.47208	22.94428	22.23337	20.90361	19.77783	19.3418	19.34661	19.36143	19.28881	19.23572	19.16486	19.06997	18.8235	18.5023	18.10051	17.60904	17.38745	17.30738	17.21673	17.1987	17.21217	17.21919	17.21489	17.21121	17.21036	17.20934	17.20956	17.20941
intake160	8.617598	-7.68719	-12.9992	-10.3711	-7.01597	-2.83796	-1.71215	-0.87376	-0.731	2.027517	4.974972	4.668292	3.708874	3.125877	2.798653	2.74504	2.878504	3.149306	3.437058	3.534257	3.51129	3.381925	3.331386	3.357713	3.358341	3.420475	3.612304	3.663405	3.708364	3.777108	3.80803	3.834859	3.842444	3.851967	3.858837	3.861684	3.863906	3.864526	3.865007
intake161	6.367767	-4.25141	-9.99347	-12.6473	-19.3678	-16.8101	-13.8724	-7.60415	-2.76605	-2.80701	-2.57184	-4.20933	-6.29363	-6.72234	-6.85772	-6.84584	-6.85609	-6.88914	-6.62973	-6.46534	-6.34106	-6.2127	-6.10784	-6.07281	-5.99565	-5.76268	-5.44272	-5.28424	-5.16562	-5.04453	-4.99306	-4.96163	-4.95435	-4.94601	-4.93999	-4.93788	-4.93631	-4.93616	-4.93613
intake162	11.31196	12.73022	15.43298	3.140564	-8.32985	-15.7118	-24.1007	-26.3388	-28.3387	-27.5073	-27.2628	-26.5997	-23.26	-22.0469	-20.7333	-20.1387	-19.7449	-19.6475	-19.4492	-19.3798	-19.3659	-19.2919	-19.1814	-19.0633	-18.9616	-18.8355	-18.6201	-18.5477	-18.4826	-18.5219	-18.5618	-18.597	-18.6011	-18.5977	-18.596	-18.5924	-18.5899	-18.5893	-18.5893
intake163	6.690674	-12.1533	-28.0214	-24.4445	-22.836	-17.8317	-19.772	-21.1098	-21.3353	-20.0088	-18.8108	-18.9245	-18.1538	-18.7735	-19.0811	-19.3489	-19.4568	-19.5391	-19.5212	-19.589	-19.7172	-19.7905	-19.9334	-20.1243	-20.3608	-20.4811	-20.5695	-20.5885	-20.6181	-20.6678	-20.7252	-20.7538	-20.7622	-20.7674	-20.7701	-20.7707	-20.7721	-20.7729	-20.773
intake164	-1.51378	-24.3902	-35.6631	-39.4056	-40.8836	-36.7794	-38.4611	-37.1288	-34.7083	-32.302	-30.1023	-28.4147	-25.9285	-24.383	-21.5427	-20.3185	-19.9334	-20.0346	-19.9713	-19.9043	-19.846	-19.8464	-19.8605	-19.8619	-19.9256	-20.1074	-20.2185	-20.3192	-20.3774	-20.3824	-20.3866	-20.3848	-20.3815	-20.3771	-20.3761	-20.3782	-20.3797	-20.38	-20.3806
intake165	-8.61369	-28.4917	-23.3334	-12.7732	-7.42758	-4.37817	-6.4632	-8.15321	-14.9055	-16.5705	-18.1423	-19.2772	-21.198	-21.9045	-21.3619	-21.0653	-20.966	-21.3926	-21.6612	-21.7986	-21.8947	-21.9929	-22.0467	-22.0719	-22.0984	-22.1827	-22.3713	-22.4954	-22.6002	-22.6781	-22.7039	-22.7131	-22.7188	-22.7215	-22.7218	-22.7212	-22.7212	-22.7219	-22.7221
intake166	13.05307	4.227024	-22.0705	-27.9293	-34.1548	-33.6399	-34.6684	-37.3844	-38.3247	-39.6489	-39.0558	-38.276	-37.6637	-37.8543	-37.8283	-38.193	-38.4028	-38.3165	-38.3806	-38.3978	-38.4611	-38.5754	-38.6837	-38.7195	-38.6508	-38.5381	-38.3048	-38.3112	-38.3391	-38.3675	-38.3643	-38.3602	-38.3487	-38.3507	-38.3499	-38.3506	-38.3515	-38.3513	-38.3514
intake167	0.56977	7.735498	14.15812	15.06914	13.95136	10.24278	3.735982	4.058571	5.349141	8.045876	11.9999	14.33036	17.45603	18.92478	21.17036	21.87002	22.60226	23.00607	23.07176	23.06603	22.95659	22.94373	22.97644	22.94535	22.9983	23.00438	22.93838	22.87508	22.86841	22.9386	22.9799	22.99125	22.97907	22.9737	22.96956	22.96988	22.96858	22.96782	22.96768
intake168	29.46632	37.76949	46.66937	46.18567	51.30009	54.85678	52.8292	50.38912	48.55803	46.21747	44.96113	44.55318	43.53134	43.02793	42.82927	43.536	44.6408	44.93837	45.13594	45.23253	45.29164	45.34921	45.42955	45.49411	45.49396	45.41796	45.36277	45.32419	45.3257	45.38125	45.3774	45.37702	45.37807	45.37162	45.36804	45.3659	45.3649	45.36415	45.36412
intake169	29.82379	43.76827	57.20127	62.50513	67.65573	66.68756	64.19719	62.42382	62.06546	61.05071	59.59183	58.66804	57.3159	56.45121	55.74334	54.94338	55.09997	54.89221	54.94368	54.84194	54.76613	54.76785	54.82256	54.86464	55.03932	55.22818	55.31024	55.34141	55.39301	55.46528	55.48232	55.49452	55.50471	55.5145	55.51964	55.52094	55.52245	55.52347	55.52374
intake170	13.4473	19.1055	27.42821	21.00313	12.38319	13.42411	11.21559	6.624917	3.135739	1.952516	1.444418	1.485787	2.34353	2.746645	2.707741	3.316833	3.522903	3.559138	3.671456	3.833098	3.990844	4.018517	4.140193	4.230807	4.337805	4.543335	4.799335	4.980395	5.064569	5.154491	5.203026	5.234833	5.233183	5.22861	5.230875	5.2279	5.226676	5.226536	5.226314
intake171	9.818288	-1.05964	-21.6445	-24.1201	-21.4423	-17.81	-12.2287	-7.96406	-6.3605	-3.91589	-2.15115	-1.17113	-0.68906	-2.13641	-4.34045	-5.44126	-5.27663	-5.24762	-5.21363	-5.35443	-5.31597	-5.32026	-5.42023	-5.5281	-5.63794	-5.82255	-5.97395	-6.04077	-6.17362	-6.24884	-6.28238	-6.30577	-6.31647	-6.32	-6.32712	-6.329	-6.32884	-6.32971	-6.33018
intake172	17.55795	16.35227	-0.51503	2.099517	10.26892	24.01504	27.47376	29.76539	34.16041	35.29816	35.86117	36.87996	38.06378	38.05907	37.754	36.99749	36.39675	36.0965	35.96927	35.79203	35.72204	35.74895	35.77892	35.79601	35.85979	35.97646	36.33193	36.51066	36.60059	36.73042	36.78591	36.81865	36.82996	36.83148	36.83235	36.83336	36.83245	36.83209	36.83196
intake173	4.398974	6.957496	3.742307	1.774862	-2.78962	-1.58449	-1.35409	-3.34108	-0.7116	2.886065	3.645156	4.478769	5.340885	6.330506	6.000408	6.266959	6.980443	7.164544	7.337643	7.367524	7.401269	7.39918	7.398789	7.488929	7.515674	7.642789	7.666444	7.651107	7.673632	7.656948	7.665396	7.657826	7.653485	7.65354	7.652879	7.653623	7.653746	7.653999	7.653779
intake174	13.18958	15.28535	20.41654	22.56606	23.95913	30.4077	34.41515	37.35377	45.07097	47.18474	48.02377	48.30809	47.01881	45.70325	44.92061	44.53113	43.70455	43.18074	42.99247	42.87655	42.82357	42.81566	42.79153	42.68445	42.63052	42.47992	42.21115	42.04792	42.00958	42.01192	42.05841	42.05146	42.05567	42.06057	42.05774	42.05705	42.05634	42.05556	42.05567
intake175	32.84797	47.65136	50.98871	42.64615	33.42475	21.64359	16.271	12.29144	8.395423	6.086614	6.372922	6.722193	5.979109	4.688282	3.730851	3.797502	4.324057	4.313414	4.36208	4.369245	4.407342	4.430324	4.518812	4.69494	4.946149	5.310858	5.71469	5.887967	5.989008	6.091352	6.109796	6.12136	6.126116	6.129589	6.128055	6.127872	6.126939	6.127383	6.127666
intake176	0.466909	10.12701	20.20887	24.29995	30.76675	39.25252	35.8338	34.59335	33.46965	30.48108	29.02914	27.15345	23.42374	21.83027	19.91598	19.30288	19.8426	19.97059	20.03433	20.15008	20.31008	20.42182	20.52292	20.6311	20.77067	20.91381	21.09132	21.20991	21.19764	21.18215	21.2053	21.18466	21.18168	21.17677	21.17867	21.18069	21.18149	21.18195	21.18195
intake177	11.24778	-0.35142	5.86119	12.43858	21.04332	31.1318	36.88882	40.67708	41.7159	40.24226	38.7527	38.58405	38.57412	38.18918	38.73814	38.74896	38.86138	38.8954	38.91449	38.95368	39.01302	38.99427	39.02147	38.97586	38.85592	38.69881	38.46156	38.30968	38.23283	38.1359	38.11923	38.10669	38.10079	38.10304	38.10459	38.10368	38.10242	38.10143	38.10148
intake178	8.022591	0.921356	6.592379	6.685709	-1.90023	-8.29767	-6.33854	-4.92546	-3.41331	-2.41944	-3.11191	-1.47329	0.56112	2.700411	5.663316	7.607821	8.93528	9.127259	9.078818	9.054728	8.996532	8.938642	8.82527	8.726741	8.657098	8.609599	8.586794	8.681384	8.736828	8.789847	8.826969	8.837582	8.83715	8.841358	8.844291	8.846654	8.848023	8.848482	8.848876
intake179	5.741359	-7.70239	-40.5573	-56.5513	-67.9825	-67.9792	-68.1413	-71.628	-75.5732	-74.3432	-73.9497	-74.1222	-74.9222	-74.1044	-73.9066	-74.2957	-74.4855	-74.7084	-75.0224	-74.9845	-74.8806	-74.8548	-74.8598	-74.9073	-74.9552	-75.0869	-75.2433	-75.3285	-75.3532	-75.3581	-75.3361	-75.327	-75.323	-75.3238	-75.3237	-75.3234	-75.3235	-75.3234	-75.3234
intake180	11.03455	18.94168	29.08273	18.64423	11.29355	10.11303	10.17287	7.712237	5.815025	5.583104	6.103638	5.911389	6.61962	7.085018	8.624589	10.41009	10.89851	11.11528	11.08136	11.05346	11.07822	11.10443	11.02297	10.89176	10.66406	10.4583	10.00993	9.789125	9.595604	9.46182	9.428513	9.403915	9.40211	9.3942	9.383777	9.380714	9.379104	9.37888	9.378818
intake181	-7.49733	-17.6798	-33.1946	-36.0201	-40.6155	-39.4139	-38.5305	-37.4366	-35.567	-36.0502	-35.0696	-34.5724	-34.5944	-35.055	-34.7272	-34.6283	-34.9038	-35.0414	-35.367	-35.3066	-35.1804	-35.1675	-35.0802	-35.0678	-35.0142	-34.7687	-34.5619	-34.4454	-34.3699	-34.3408	-34.3712	-34.3715	-34.3872	-34.3894	-34.3931	-34.3944	-34.3946	-34.3946	-34.3947
intake182	6.601574	12.9392	14.63937	0.568767	-11.6819	-20.2933	-20.1195	-22.6914	-22.9531	-21.1879	-17.6043	-16.3405	-14.2853	-13.1634	-11.8847	-11.5979	-11.7808	-11.6015	-11.5392	-11.6603	-11.5644	-11.4479	-11.4089	-11.2874	-11.1037	-10.8477	-10.5816	-10.3692	-10.1941	-10.0903	-10.0406	-10.0297	-10.0311	-10.026	-10.0216	-10.0221	-10.0235	-10.0245	-10.0248
intake183	-2.991	-4.64593	-7.20091	-2.65516	0.780946	10.74387	12.50129	11.39218	8.912092	9.267396	10.24379	10.12781	12.7078	14.2104	15.73507	15.4056	15.69128	15.77028	15.75751	15.92032	16.05205	16.13028	16.21614	16.28973	16.36152	16.40137	16.49714	16.59733	16.64116	16.64906	16.64313	16.6302	16.63007	16.63539	16.6272	16.62374	16.62439	16.62468	16.62527
intake184	12.21871	9.165632	-1.67198	-5.4286	-4.01912	-2.28083	-2.70191	-1.86459	-2.68112	-3.91074	-3.42957	-1.51919	-0.82786	-1.67003	-2.92854	-3.94559	-4.36072	-4.64014	-4.53774	-4.24209	-4.19066	-4.16928	-4.24952	-4.30035	-4.39048	-4.59763	-4.87637	-4.96617	-4.99785	-5.02507	-5.01683	-5.01404	-5.01614	-5.01703	-5.01623	-5.01444	-5.01332	-5.01438	-5.0149
intake185	0.573671	-7.81038	-14.3848	-25.0199	-28.5581	-19.5175	-16.0445	-12.5367	-9.58658	-9.01218	-7.69854	-6.31175	-5.96704	-6.09799	-7.11331	-7.2756	-7.06949	-6.87973	-6.57225	-6.45179	-6.42673	-6.44769	-6.47262	-6.47002	-6.52843	-6.53375	-6.62967	-6.70419	-6.78454	-6.81473	-6.85694	-6.8696	-6.86927	-6.85989	-6.85453	-6.8525	-6.8506	-6.84973	-6.84947
intake186	3.020991	-0.69176	-6.29955	-10.8981	-24.1302	-26.4909	-26.1751	-26.5746	-26.7767	-28.2018	-27.4713	-26.989	-26.9335	-26.0883	-24.6245	-24.3319	-24.3927	-24.1361	-23.9162	-23.8307	-23.8115	-23.8362	-23.8453	-23.8866	-23.9048	-23.849	-23.8262	-23.7365	-23.6963	-23.6302	-23.6015	-23.6095	-23.6127	-23.6172	-23.6195	-23.6163	-23.6151	-23.6143	-23.6144
intake187	3.868748	-13.768	-18.3569	-18.3562	-17.5849	-9.24752	-6.59137	-5.2675	-6.13052	-6.56623	-7.09454	-7.19241	-7.03945	-6.5425	-5.55497	-5.05681	-5.07065	-5.35928	-5.71959	-5.84447	-5.83302	-5.80346	-5.81159	-5.82695	-5.78145	-5.80059	-5.63745	-5.56032	-5.52652	-5.49214	-5.47505	-5.45341	-5.45337	-5.44651	-5.44337	-5.44106	-5.44139	-5.44034	-5.44028
intake188	2.959919	13.03961	3.953264	1.245976	-2.4502	-7.04112	-6.90045	-3.03947	-2.61845	-3.19681	-2.84864	-2.33885	-1.46843	-1.03018	-1.83558	-2.4916	-2.57258	-2.88815	-3.20698	-3.49497	-3.58137	-3.68833	-3.6585	-3.67052	-3.7068	-3.71592	-3.64001	-3.58083	-3.60512	-3.64922	-3.65707	-3.66164	-3.66195	-3.66392	-3.67031	-3.67338	-3.6743	-3.67539	-3.67541
intake189	8.040056	14.73457	21.97298	28.43484	21.03191	19.28029	20.69762	20.52856	21.28801	22.74315	22.59612	21.77696	19.45386	17.45572	15.83784	15.49528	15.27494	15.16267	15.08057	15.03776	14.97299	14.8834	14.78539	14.69681	14.55176	14.20865	13.78212	13.6674	13.58877	13.59333	13.57971	13.57182	13.57207	13.57626	13.57592	13.57564	13.57445	13.57393	13.5737
intake190	50.34113	117.258	204.572	239.3316	274.385	301.1297	314.2356	324.7838	333.9911	338.5903	341.1399	342.4065	344.5165	345.794	347.5082	348.0806	348.2527	348.3793	348.4532	348.5139	348.5435	348.5522	348.5642	348.6042	348.6524	348.7605	348.8759	348.9062	348.9234	348.9526	348.9633	348.9667	348.9668	348.9663	348.9664	348.9661	348.9661	348.9665	348.9667
intake191	29.27695	55.3467	77.61761	91.16933	104.1218	118.7984	121.4717	123.9301	125.1036	125.8291	128.121	130.2247	132.205	133.5688	134.3008	134.8731	135.0712	134.9324	134.8204	134.8166	134.7543	134.6689	134.5581	134.4153	134.2853	134.0979	133.7781	133.7343	133.71	133.5969	133.5679	133.559	133.5592	133.5612	133.5644	133.5668	133.567	133.5667	133.5662
intake192	25.30575	45.42483	49.96157	46.17597	47.08693	47.69758	45.70187	45.6594	44.27491	43.33221	43.48815	42.80458	41.92434	42.28497	42.73962	43.28863	43.42488	43.57732	43.57833	43.55747	43.59514	43.68457	43.80052	43.93976	44.17373	44.43706	44.83306	45.03325	45.15404	45.22917	45.23884	45.25715	45.26229	45.27514	45.27573	45.27798	45.2779	45.27717	45.27725
intake193	12.10616	36.26134	45.68842	39.43957	30.64998	27.55324	26.78465	30.14904	34.54371	35.60482	36.35958	35.69291	33.12995	32.59122	32.86473	31.99284	32.13736	32.09681	32.03755	31.91236	31.78135	31.62742	31.51028	31.32124	31.04238	30.54442	29.93029	29.64684	29.45081	29.22143	29.12795	29.11668	29.1193	29.12108	29.1208	29.12128	29.12217	29.12237	29.12204
intake194	7.968472	15.05596	18.55047	14.9759	16.39413	21.39342	21.04568	21.8816	16.68994	11.62143	9.350068	8.86889	9.650553	10.80766	12.32902	12.51442	12.09613	12.48046	12.85032	12.81871	12.81791	12.74017	12.62972	12.57154	12.51182	12.32936	12.06041	11.89601	11.80328	11.73252	11.70071	11.69256	11.68656	11.69077	11.68861	11.68808	11.68712	11.68728	11.68696
intake195	-1.5235	7.179203	19.57191	28.54871	24.5831	22.85847	23.27269	21.4486	23.05742	23.87385	26.61597	28.29134	30.76758	31.6077	32.68624	33.06119	33.02982	32.81179	32.55886	32.39547	32.30375	32.27304	32.35921	32.47468	32.45581	32.4227	32.47916	32.54872	32.6107	32.64612	32.65027	32.65216	32.65243	32.65569	32.65988	32.66049	32.66036	32.66057	32.66007
intake196	-7.48081	-4.6122	5.496904	19.78866	27.49366	31.72501	31.64744	32.90642	31.67278	31.87748	32.66066	32.88309	32.12422	31.06074	29.80422	30.07973	29.38119	29.07132	28.88968	28.83868	28.93014	28.96437	28.99103	28.98768	29.12274	29.23305	29.38608	29.43133	29.48823	29.51966	29.5206	29.51187	29.50644	29.49788	29.49003	29.48775	29.48989	29.49052	29.49004
intake197	17.28081	34.04177	30.69883	28.07602	19.58852	14.59468	7.535114	3.210395	-1.22854	-3.00735	-3.48596	-3.23944	-3.29356	-3.64756	-4.29601	-3.44432	-3.3769	-3.28216	-3.50789	-3.5504	-3.65424	-3.70496	-3.75612	-3.8357	-3.95825	-4.20063	-4.46627	-4.50517	-4.46344	-4.4473	-4.43565	-4.40543	-4.39674	-4.38779	-4.38576	-4.38495	-4.38346	-4.38321	-4.38289
intake198	5.025893	16.61927	40.32624	35.66959	26.21827	21.17299	19.3305	19.7954	17.81585	17.33264	13.86718	13.35061	12.63948	11.99838	12.43456	12.73063	13.23539	13.18981	13.05567	12.94914	12.97339	12.99697	12.94268	12.87527	12.83272	12.8667	13.00677	13.09084	13.18873	13.23705	13.26745	13.27411	13.28307	13.28799	13.29021	13.28927	13.29017	13.29084	13.29067
intake199	11.21381	11.59438	28.28537	27.38727	20.71326	16.55734	15.68186	14.67857	16.05375	18.14208	20.30796	21.44173	21.52952	21.04964	20.56561	19.92226	19.86227	20.00118	19.92126	19.80662	19.83688	19.89486	19.86692	19.80396	19.69482	19.53714	19.27917	19.12204	18.96408	18.91405	18.87908	18.91136	18.91343	18.91529	18.91902	18.92155	18.92407	18.92516	18.9258
intake1100	19.47786	25.39694	21.2978	20.92604	14.30605	8.198534	7.959413	8.095422	3.643473	1.168225	-1.60423	-2.50503	-2.49993	-1.96929	-2.4747	-1.90634	-1.63981	-1.36018	-1.22207	-1.14182	-1.12983	-1.1912	-1.23887	-1.39294	-1.60287	-1.9074	-2.14198	-2.29235	-2.35145	-2.40366	-2.42173	-2.43197	-2.42935	-2.42757	-2.42824	-2.42938	-2.43018	-2.43095	-2.43085
intake1101	-5.59259	-15.3066	-12.8198	-7.65738	-0.67082	9.293372	5.942948	3.584347	5.467094	5.982828	7.393819	8.036362	8.425858	9.670138	10.21355	10.62652	10.89601	11.03257	10.95519	10.99065	11.08688	11.23923	11.38418	11.57317	11.79446	12.02707	12.32791	12.50794	12.59017	12.67361	12.67784	12.69406	12.69846	12.69237	12.69033	12.68763	12.68703	12.68651	12.68632
intake1102	-12.7215	-28.6808	-16.7604	-11.0857	-6.94638	-4.40214	-5.4715	-8.45327	-13.3612	-15.8287	-17.6211	-17.8197	-17.2068	-17.6858	-17.8121	-18.0918	-17.7272	-17.639	-17.5369	-17.5126	-17.2958	-17.209	-17.0552	-16.8179	-16.4815	-15.9706	-15.2208	-14.9029	-14.6975	-14.5617	-14.5004	-14.4465	-14.4271	-14.4226	-14.4235	-14.4208	-14.4203	-14.4207	-14.4207
intake1103	7.204709	7.756802	15.53068	25.62313	21.40576	15.48325	12.45562	6.872249	0.797967	-0.04674	1.161421	2.304322	3.799903	3.652805	2.771936	1.454431	0.836259	0.595297	0.555187	0.573264	0.548751	0.647165	0.757683	0.84954	0.986371	1.201176	1.396664	1.499685	1.54091	1.541616	1.58966	1.617719	1.62491	1.633145	1.638627	1.639172	1.639146	1.639419	1.639769
intake1104	20.54691	17.20781	11.26754	9.479824	8.616017	8.047215	7.436741	4.376482	4.050409	2.688351	2.324999	1.445667	1.180638	1.312009	1.89791	1.581316	1.268545	1.314722	1.367726	1.29149	1.232792	1.151423	1.031612	0.987395	0.931575	0.736196	0.652383	0.668744	0.686757	0.683847	0.68572	0.663246	0.652106	0.646635	0.646189	0.645011	0.646592	0.647156	0.647196
intake1105	7.89161	23.17344	19.07442	14.15025	9.156623	4.134308	-0.80601	-0.11442	-1.31619	-2.30739	-4.50258	-5.55785	-5.42797	-6.25838	-7.67415	-8.91283	-9.81934	-10.3268	-10.5873	-10.6625	-10.5889	-10.531	-10.4387	-10.3149	-10.2957	-10.2518	-10.1806	-10.1619	-10.1523	-10.0912	-10.0445	-10.0508	-10.0657	-10.0698	-10.067	-10.0654	-10.0648	-10.0639	-10.0637
intake1106	14.32781	11.66035	-0.59662	-2.14531	-5.46553	-13.0919	-17.5433	-18.0219	-14.8613	-13.5147	-11.2852	-10.7461	-10.5173	-10.5658	-10.627	-10.8032	-10.5842	-10.6655	-10.8648	-10.7723	-10.8202	-10.8368	-10.8429	-10.8321	-10.8475	-10.9016	-10.8989	-10.9712	-11.0437	-11.0909	-11.1098	-11.1329	-11.1396	-11.1407	-11.146	-11.1505	-11.1514	-11.1512	-11.1511
intake1107	18.79655	14.6364	1.259925	-2.17441	-2.36243	-4.26823	-4.28411	-7.05714	-9.29412	-12.1347	-15.7463	-17.7689	-20.6702	-22.1125	-22.612	-22.4651	-21.8877	-21.3229	-21.0985	-21.0176	-21.0081	-20.9496	-20.8562	-20.752	-20.6684	-20.3751	-20.1031	-19.9647	-19.866	-19.8042	-19.7283	-19.6655	-19.6539	-19.6503	-19.653	-19.6549	-19.6547	-19.6547	-19.6552
intake1108	5.315411	-13.4347	-18.7306	-13.4911	-5.59833	-3.0221	-4.5439	-3.06632	-0.92225	0.799752	1.046329	0.436313	-0.19422	-0.3635	0.208495	0.131925	-0.02065	-0.17029	-0.39878	-0.499	-0.54865	-0.60074	-0.65383	-0.64219	-0.62821	-0.6134	-0.53877	-0.42038	-0.31056	-0.24242	-0.18788	-0.1605	-0.15743	-0.16326	-0.16705	-0.16724	-0.1661	-0.16493	-0.16471
intake1109	7.669305	10.20723	-5.17496	-4.1901	-8.60883	-17.6121	-22.6082	-24.7778	-25.8325	-24.2628	-23.9632	-23.749	-21.1755	-19.6106	-18.742	-17.7704	-17.109	-16.9784	-16.586	-16.4379	-16.3234	-16.3291	-16.3911	-16.4338	-16.4559	-16.4962	-16.5431	-16.6106	-16.6595	-16.7128	-16.68	-16.6607	-16.6668	-16.6636	-16.6646	-16.6647	-16.6648	-16.6642	-16.6642

sio1.40.fac	sio1.41.fac	sio1.42.fac	sio1.43.fac	sio1.44.fac	sio1.45.fac	sio1.46.fac	sio1.47.fac	sio1.48.fac	sio1.49.fac	sio1.50.fac	sio1.51.fac	sio1.52.factors
−11.4918	−11.4918	−11.4918	−11.4918	−11.4918	−11.4918	−11.4918	−11.4918	−11.4918	−11.4918	−11.4918	−11.4918	−11.4918
47.18209	47.18205	47.18204	47.18205	47.18205	47.18205	47.18205	47.18205	47.18205	47.18205	47.18205	47.18205	47.18205
84.27733	84.27729	84.27732	84.27735	84.27735	84.27735	84.27735	84.27735	84.27735	84.27735	84.27735	84.27735	84.27735
67.14499	67.145	67.14499	67.14499	67.14499	67.14499	67.14499	67.14499	67.14499	67.14499	67.14499	67.14499	67.14499
12.61839	12.61844	12.61846	12.61846	12.61846	12.61846	12.61846	12.61846	12.61846	12.61846	12.61846	12.61846	12.61846
−21.7953	−21.7952	−21.7952	−21.7952	−21.7952	−21.7952	−21.7952	−21.7952	−21.7952	−21.7952	−21.7952	−21.7952	−21.7952
17.02977	17.02981	17.02982	17.02981	17.02982	17.02982	17.02982	17.02982	17.02982	17.02982	17.02982	17.02982	17.02982
3.502595	3.502674	3.502768	3.502782	3.502785	3.502785	3.502784	3.502784	3.502784	3.502784	3.502784	3.502784	3.502784
26.25209	26.25214	26.25216	26.25217	26.25217	26.25216	26.25216	26.25216	26.25216	26.25216	26.25216	26.25216	26.25216
−21.5647	−21.5646	−21.5646	−21.5646	−21.5646	−21.5646	−21.5646	−21.5646	−21.5646	−21.5646	−21.5646	−21.5646	−21.5646
29.63129	29.6313	29.63134	29.63135	29.63135	29.63135	29.63135	29.63135	29.63135	29.63135	29.63135	29.63135	29.63135
1.073916	1.073807	1.073765	1.073756	1.073752	1.073753	1.073753	1.073753	1.073753	1.073753	1.073753	1.073753	1.073753
4.772136	4.772139	4.772147	4.772151	4.772154	4.772153	4.772153	4.772153	4.772153	4.772153	4.772153	4.772153	4.772153
41.112	41.11203	41.11202	41.11201	41.11201	41.11201	41.11201	41.11201	41.11201	41.11201	41.11201	41.11201	41.11201
53.16551	53.16554	53.16555	53.16555	53.16555	53.16555	53.16555	53.16555	53.16555	53.16555	53.16555	53.16555	53.16555
−5.02501	−5.02503	−5.02499	−5.02497	−5.02497	−5.02497	−5.02497	−5.02497	−5.02497	−5.02497	−5.02497	−5.02497	−5.02497
10.18033	10.18036	10.18036	10.18036	10.18035	10.18035	10.18035	10.18035	10.18035	10.18035	10.18035	10.18035	10.18035
−10.7492	−10.7492	−10.7492	−10.7492	−10.7492	−10.7492	−10.7492	−10.7492	−10.7492	−10.7492	−10.7492	−10.7492	−10.7492
16.12541	16.12541	16.12542	16.12542	16.12542	16.12542	16.12542	16.12542	16.12542	16.12542	16.12542	16.12542	16.12542
13.66183	13.66184	13.66183	13.66184	13.66185	13.66186	13.66186	13.66186	13.66186	13.66186	13.66186	13.66186	13.66186
−32.4767	−32.4766	−32.4766	−32.4766	−32.4766	−32.4766	−32.4766	−32.4766	−32.4766	−32.4766	−32.4766	−32.4766	−32.4766
−10.5375	−10.5374	−10.5373	−10.5373	−10.5373	−10.5373	−10.5373	−10.5373	−10.5373	−10.5373	−10.5373	−10.5373	−10.5373
0.733736	0.733712	0.733682	0.733673	0.73367	0.733666	0.733665	0.733665	0.733665	0.733664	0.733665	0.733665	0.733665
10.64671	10.64675	10.64673	10.64674	10.64673	10.64673	10.64673	10.64673	10.64673	10.64673	10.64673	10.64673	10.64673
0.555215	0.555343	0.555348	0.555353	0.555359	0.555363	0.555363	0.555363	0.555363	0.555363	0.555363	0.555363	0.555363
66.11882	66.11878	66.1188	66.11879	66.11878	66.11878	66.11878	66.11878	66.11878	66.11878	66.11878	66.11878	66.11878
9.517008	9.516989	9.517033	9.517036	9.517032	9.517028	9.517026	9.517026	9.517026	9.517026	9.517026	9.517026	9.517026
48.13244	48.13238	48.13241	48.13242	48.13243	48.13243	48.13243	48.13243	48.13243	48.13243	48.13243	48.13243	48.13243
36.97686	36.9768	36.9768	36.97682	36.97682	36.97681	36.97681	36.97681	36.97681	36.97681	36.97681	36.97681	36.97681
5.056344	5.05644	5.056481	5.056513	5.056514	5.056513	5.056513	5.056513	5.056513	5.056513	5.056513	5.056513	5.056513
−33.0602	−33.0602	−33.0603	−33.0603	−33.0603	−33.0603	−33.0603	−33.0603	−33.0603	−33.0603	−33.0603	−33.0603	−33.0603
−20.3767	−20.3767	−20.3767	−20.3767	−20.3767	−20.3767	−20.3767	−20.3767	−20.3767	−20.3767	−20.3767	−20.3767	−20.3767
19.11845	19.11842	19.11841	19.1184	19.11839	19.11839	19.11839	19.11839	19.11839	19.11839	19.11839	19.11839	19.11839
21.73796	21.73792	21.73792	21.73794	21.73794	21.73794	21.73794	21.73794	21.73794	21.73794	21.73794	21.73794	21.73794
24.34947	24.34947	24.34946	24.34946	24.34946	24.34947	24.34947	24.34947	24.34947	24.34947	24.34947	24.34947	24.34947
−74.139	−74.1391	−74.139	−74.139	−74.139	−74.139	−74.139	−74.139	−74.139	−74.139	−74.139	−74.139	−74.139
14.98988	14.98976	14.98974	14.98973	14.98973	14.98973	14.98973	14.98973	14.98973	14.98973	14.98973	14.98973	14.98973
2.676159	2.676077	2.676081	2.676084	2.676083	2.676081	2.67608	2.67608	2.67608	2.67608	2.67608	2.67608	2.67608
76.49114	76.4912	76.49121	76.49122	76.49122	76.49122	76.49122	76.49122	76.49122	76.49122	76.49122	76.49122	76.49122
−13.6547	−13.6547	−13.6547	−13.6546	−13.6546	−13.6546	−13.6546	−13.6546	−13.6546	−13.6546	−13.6546	−13.6546	−13.6546
−26.1933	−26.1934	−26.1935	−26.1935	−26.1935	−26.1935	−26.1935	−26.1935	−26.1935	−26.1935	−26.1935	−26.1935	−26.1935
4.266725	4.266689	4.266669	4.266673	4.266674	4.266677	4.266677	4.266676	4.266676	4.266676	4.266676	4.266676	4.266676
4.878727	4.878728	4.878734	4.878743	4.878747	4.878752	4.878752	4.878751	4.878752	4.878752	4.878751	4.878751	4.878751
−14.0594	−14.0593	−14.0593	−14.0593	−14.0593	−14.0593	−14.0593	−14.0593	−14.0593	−14.0593	−14.0593	−14.0593	−14.0593
−4.21157	−4.21149	−4.21154	−4.21157	−4.21158	−4.21158	−4.21158	−4.21158	−4.21158	−4.21158	−4.21158	−4.21158	−4.21158
−8.49975	−8.49978	−8.49978	−8.4998	−8.4998	−8.4998	−8.4998	−8.4998	−8.4998	−8.4998	−8.4998	−8.4998	−8.4998
14.39674	14.39677	14.39678	14.39679	14.39678	14.39679	14.39679	14.39679	14.39679	14.39679	14.39679	14.39679	14.39679
−2.35226	−2.35231	−2.35236	−2.35239	−2.3524	−2.3524	−2.3524	−2.3524	−2.3524	−2.3524	−2.3524	−2.3524	−2.3524
32.41842	32.41844	32.41844	32.41845	32.41846	32.41846	32.41845	32.41846	32.41846	32.41846	32.41846	32.41846	32.41846
52.76535	52.76529	52.76524	52.76522	52.76522	52.76522	52.76522	52.76522	52.76522	52.76522	52.76522	52.76522	52.76522
−17.9678	−17.9678	−17.9678	−17.9678	−17.9678	−17.9678	−17.9678	−17.9678	−17.9678	−17.9678	−17.9678	−17.9678	−17.9678
−47.0639	−47.0639	−47.064	−47.064	−47.064	−47.064	−47.064	−47.064	−47.064	−47.064	−47.064	−47.064	−47.064
24.17126	24.17125	24.17129	24.17129	24.17129	24.17129	24.17129	24.17129	24.17129	24.17129	24.17129	24.17129	24.17129
−32.6718	−32.6718	−32.6718	−32.6718	−32.6718	−32.6718	−32.6718	−32.6718	−32.6718	−32.6718	−32.6718	−32.6718	−32.6718
3.365935	3.365942	3.365945	3.365935	3.365933	3.365931	3.365931	3.365931	3.365931	3.365931	3.365931	3.365931	3.365931
39.96242	39.96237	39.96236	39.96236	39.96236	39.96236	39.96236	39.96236	39.96236	39.96236	39.96236	39.96236	39.96236
−41.2521	−41.2521	−41.252	−41.2521	−41.2521	−41.2521	−41.2521	−41.2521	−41.2521	−41.2521	−41.2521	−41.2521	−41.2521
17.33217	17.33215	17.33215	17.33215	17.33215	17.33215	17.33215	17.33215	17.33215	17.33215	17.33215	17.33215	17.33215
17.20943	17.2094	17.20941	17.20939	17.20939	17.2094	17.2094	17.2094	17.2094	17.2094	17.2094	17.2094	17.2094
3.865026	3.865108	3.865135	3.865155	3.865152	3.865151	3.865151	3.865151	3.865151	3.865151	3.865151	3.865151	3.865151
−4.93603	−4.9359	−4.93589	−4.93589	−4.9359	−4.9359	−4.93589	−4.93589	−4.93589	−4.93589	−4.93589	−4.93589	−4.93589
−18.5893	−18.5893	−18.5893	−18.5893	−18.5893	−18.5893	−18.5893	−18.5893	−18.5893	−18.5893	−18.5893	−18.5893	−18.5893
−20.773	−20.7731	−20.7731	−20.773	−20.773	−20.773	−20.773	−20.773	−20.773	−20.773	−20.773	−20.773	−20.773
−20.3806	−20.3805	−20.3805	−20.3804	−20.3804	−20.3804	−20.3804	−20.3804	−20.3804	−20.3804	−20.3804	−20.3804	−20.3804
−22.7221	−22.7222	−22.7222	−22.7221	−22.7221	−22.7221	−22.7221	−22.7221	−22.7221	−22.7221	−22.7221	−22.7221	−22.7221
−38.3515	−38.3515	−38.3515	−38.3515	−38.3515	−38.3515	−38.3515	−38.3515	−38.3515	−38.3515	−38.3515	−38.3515	−38.3515
22.96757	22.96751	22.96748	22.96747	22.96747	22.96747	22.96747	22.96747	22.96747	22.96747	22.96747	22.96747	22.96747
45.36415	45.36413	45.36414	45.36415	45.36416	45.36415	45.36415	45.36415	45.36415	45.36415	45.36415	45.36415	45.36415
55.52376	55.52379	55.52381	55.52383	55.52384	55.52384	55.52384	55.52384	55.52384	55.52384	55.52384	55.52384	55.52384
5.226232	5.226206	5.22614	5.22612	5.226118	5.226118	5.226117	5.226117	5.226117	5.226117	5.226117	5.226117	5.226117
−6.33031	−6.33033	−6.33031	−6.33031	−6.33031	−6.3303	−6.3303	−6.3303	−6.3303	−6.3303	−6.3303	−6.3303	−6.3303
36.832	36.83202	36.83198	36.83198	36.83199	36.83198	36.83198	36.83198	36.83198	36.83198	36.83198	36.83198	36.83198
7.653783	7.653745	7.653744	7.653741	7.653746	7.653745	7.653746	7.653746	7.653746	7.653746	7.653746	7.653746	7.653746
42.0557	42.05569	42.05569	42.05567	42.05566	42.05566	42.05566	42.05566	42.05566	42.05566	42.05566	42.05566	42.05566
6.127724	6.127717	6.127696	6.127706	6.127708	6.127709	6.127709	6.12771	6.12771	6.12771	6.12771	6.12771	6.12771
21.18193	21.18187	21.18184	21.18184	21.18183	21.18183	21.18183	21.18183	21.18183	21.18183	21.18183	21.18183	21.18183
38.10144	38.1014	38.10137	38.10138	38.10139	38.10139	38.10139	38.10139	38.10139	38.10139	38.10139	38.10139	38.10139
8.848924	8.848903	8.848917	8.848908	8.848909	8.848909	8.848909	8.848909	8.848909	8.848909	8.848909	8.848909	8.848909
−75.3234	−75.3235	−75.3235	−75.3235	−75.3235	−75.3235	−75.3235	−75.3235	−75.3235	−75.3235	−75.3235	−75.3235	−75.3235
9.37886	9.378928	9.378914	9.378903	9.378902	9.378897	9.378897	9.378897	9.378897	9.378897	9.378897	9.378897	9.378897
−34.3947	−34.3947	−34.3947	−34.3947	−34.3947	−34.3947	−34.3947	−34.3947	−34.3947	−34.3947	−34.3947	−34.3947	−34.3947
−10.0249	−10.025	−10.025	−10.025	−10.025	−10.025	−10.025	−10.025	−10.025	−10.025	−10.025	−10.025	−10.025
16.62534	16.62535	16.62537	16.62538	16.62537	16.62537	16.62537	16.62537	16.62537	16.62537	16.62537	16.62537	16.62537
−5.01491	−5.01492	−5.01496	−5.01498	−5.01498	−5.01498	−5.01498	−5.01498	−5.01498	−5.01498	−5.01498	−5.01498	−5.01498
−6.84941	−6.84948	−6.84947	−6.84947	−6.84947	−6.84946	−6.84946	−6.84946	−6.84946	−6.84946	−6.84946	−6.84946	−6.84946
−23.6145	−23.6146	−23.6146	−23.6146	−23.6146	−23.6146	−23.6146	−23.6146	−23.6146	−23.6146	−23.6146	−23.6146	−23.6146
−5.44025	−5.44028	−5.44029	−5.44029	−5.4403	−5.4403	−5.4403	−5.4403	−5.4403	−5.4403	−5.4403	−5.4403	−5.4403
−3.67526	−3.6752	−3.67517	−3.67517	−3.67518	−3.67518	−3.67518	−3.67518	−3.67518	−3.67518	−3.67518	−3.67518	−3.67518
13.57354	13.57345	13.57342	13.57341	13.5734	13.5734	13.5734	13.5734	13.5734	13.5734	13.5734	13.5734	13.5734
348.9668	348.9668	348.9668	348.9668	348.9668	348.9668	348.9668	348.9668	348.9668	348.9668	348.9668	348.9668	348.9668
133.5661	133.5661	133.5661	133.5661	133.5661	133.5661	133.5661	133.5661	133.5661	133.5661	133.5661	133.5661	133.5661
45.27722	45.27729	45.27725	45.27724	45.27724	45.27725	45.27725	45.27725	45.27725	45.27725	45.27725	45.27725	45.27725
29.12193	29.12187	29.12185	29.12186	29.12186	29.12185	29.12185	29.12185	29.12185	29.12185	29.12185	29.12185	29.12185
11.68687	11.68678	11.68679	11.6868	11.6868	11.6868	11.6868	11.6868	11.6868	11.6868	11.6868	11.6868	11.6868
32.65993	32.65988	32.65985	32.65983	32.65982	32.65982	32.65982	32.65982	32.65982	32.65982	32.65982	32.65982	32.65982
29.49002	29.49	29.48999	29.49	29.49	29.49	29.49	29.49	29.49	29.49	29.49	29.49	29.49
−4.38282	−4.38283	−4.38283	−4.38282	−4.38282	−4.38282	−4.38282	−4.38282	−4.38282	−4.38282	−4.38282	−4.38282	−4.38282
13.29048	13.29042	13.29042	13.29043	13.29044	13.29044	13.29044	13.29045	13.29045	13.29045	13.29045	13.29045	13.29045
18.92594	18.92595	18.92595	18.92593	18.92593	18.92593	18.92593	18.92593	18.92593	18.92593	18.92593	18.92593	18.92593
−2.43094	−2.43104	−2.43106	−2.43106	−2.43106	−2.43106	−2.43106	−2.43106	−2.43106	−2.43106	−2.43106	−2.43106	−2.43106
12.68623	12.68629	12.68634	12.68636	12.68637	12.68638	12.68638	12.68638	12.68638	12.68638	12.68638	12.68638	12.68638
−14.4208	−14.4208	−14.4208	−14.4208	−14.4208	−14.4208	−14.4208	−14.4208	−14.4208	−14.4208	−14.4208	−14.4208	−14.4208
1.639917	1.639907	1.639921	1.639935	1.639937	1.639935	1.639935	1.639935	1.639935	1.639935	1.639935	1.639935	1.639935
0.647247	0.647298	0.6473	0.647303	0.647308	0.647311	0.647311	0.647311	0.647311	0.647311	0.647311	0.647311	0.647311
−10.0638	−10.0638	−10.0638	−10.0638	−10.0638	−10.0638	−10.0638	−10.0638	−10.0638	−10.0638	−10.0638	−10.0638	−10.0638
−11.1515	−11.1515	−11.1515	−11.1516	−11.1516	−11.1516	−11.1516	−11.1516	−11.1516	−11.1516	−11.1516	−11.1516	−11.1516
−19.6553	−19.6553	−19.6553	−19.6553	−19.6553	−19.6553	−19.6553	−19.6553	−19.6553	−19.6553	−19.6553	−19.6553	−19.6553
−0.16462	−0.16471	−0.16477	−0.16475	−0.16475	−0.16475	−0.16475	−0.16475	−0.16475	−0.16475	−0.16475	−0.16475	−0.16475
−16.6641	−16.664	−16.664	−16.664	−16.664	−16.664	−16.664	−16.664	−16.664	−16.664	−16.664	−16.664	−16.664

Loadingweights

	Factor1	Factor2	Factor3	Factor4	Factor5	Factor6	Factor7	Factor8	Factor9	Factor10	Factor11	Factor12	Factor13
intake11	0.037484	-0.09134	-0.07634	0.07575	-0.00298	-0.05967	-0.03994	0.085994	0.244206	-0.10837	-0.04554	0.080496	-0.00338
intake12	0.007537	0.070027	0.112675	0.150773	-0.19601	0.110892	0.106281	-0.05057	-0.00673	0.004168	-0.07431	-0.02057	-0.17043
intake13	0.059289	0.120608	0.105162	0.061432	-0.16111	0.083044	0.170865	-0.05185	0.086769	-0.07952	0.150469	0.04985	0.090384
intake14	0.180677	0.04515	0.086593	-0.0347	0.030798	0.022328	0.070664	-0.06991	-0.12152	-0.01811	0.188467	-0.24416	-0.04073
intake15	0.068875	-0.08762	0.111185	0.017286	-0.06373	-0.05258	0.050204	0.119616	-0.0491	0.109402	-0.03483	-0.0874	-0.08572
intake16	0.076821	-0.11501	0.092717	-0.08903	-0.10761	-0.15355	0.086022	0.010496	-0.03218	0.010577	0.047469	0.045094	0.039032
intake17	0.050187	0.166671	0.144684	-0.10708	-0.26597	-0.18385	-0.06944	0.064772	-0.02136	-0.08176	0.024502	-0.08374	0.060672
intake18	-0.0208	-0.07381	0.147631	0.042781	-0.07067	0.106349	0.048389	-0.02919	0.033682	-0.1439	-0.14301	-0.01206	-0.03508
intake19	0.021641	-0.08372	0.191258	-0.00285	0.103679	-0.07843	-0.10652	-0.05502	0.08992	-0.06024	-0.01734	0.175339	0.051973
intake110	-0.01354	0.08841	-0.11941	-0.01226	-0.05381	0.131325	-0.15838	0.077473	-0.09712	-0.02726	-0.1276	0.037041	0.018279
intake111	0.112837	0.061857	-0.13396	0.09894	-0.0528	0.108794	-0.06417	0.018715	-0.09085	0.049757	-0.07443	0.087892	-0.10836
intake112	0.002047	0.028823	0.004912	0.009722	0.023976	-0.04048	-0.09046	-0.13385	0.108101	-0.04679	0.096267	-0.03443	-0.02692
intake113	0.140381	-0.03972	-0.17307	0.096775	-0.0899	-0.05869	0.094652	0.023604	0.075448	0.1272	-0.04606	-0.04018	-0.10287
intake114	0.15319	-0.05985	0.070188	-0.0324	-0.01517	-0.10612	0.064843	0.117848	0.060728	-0.08665	0.035578	0.114466	-0.04201
intake115	-0.01234	0.086957	-0.08477	0.121574	0.009202	0.130941	-0.00716	0.192309	-0.04715	0.09247	0.13051	0.241589	-0.06871
intake116	0.153772	-0.07099	-0.02864	-0.05095	-0.06172	-0.1601	0.05245	0.037605	-0.01603	-0.10375	0.19323	-0.02994	-0.05759
intake117	-0.06065	0.111253	-0.07932	0.083607	-0.08395	0.000805	-0.09463	0.15112	0.032093	0.098846	0.085852	-0.04064	0.064663
intake118	0.018413	0.061827	-0.08884	0.078144	-0.08432	0.150383	-0.09841	-0.15036	-0.07535	-0.04966	-0.13639	-0.05251	-0.08342
intake119	0.178557	0.007374	-0.1191	0.110961	-0.0373	-0.0778	-0.14318	-0.15956	0.038013	-0.0347	-0.1246	0.166304	0.04009
intake120	0.004077	-0.10345	-0.01636	0.216331	-0.01177	-0.03439	0.105373	0.024615	0.179574	-0.21123	0.080695	-0.00913	0.092867
intake121	-0.01908	0.010234	-0.12023	0.098086	-0.19663	0.02843	-0.03653	0.024266	0.110106	-0.0775	-0.05772	-0.04996	-0.00988
intake122	0.116078	0.026246	-0.10129	0.067977	-0.11491	-0.06569	-0.14126	-0.17036	-0.03673	0.115792	-0.10837	0.04608	-0.12025
intake123	0.015425	0.018987	0.044622	-0.12233	0.0641	0.105021	-0.25335	0.088076	0.029379	0.089397	-0.06104	-0.04163	-0.04258
intake124	0.095686	-0.1008	-0.08155	-0.01591	0.064913	0.075539	0.188521	0.052172	-0.0812	-0.0092	-0.04891	0.221585	-0.05463
intake125	-0.00254	0.0226	0.053483	0.007314	-0.09566	0.141237	0.047377	-0.18214	-0.08518	-0.09668	-0.0204	-0.08056	0.0426
intake126	0.182371	0.04361	0.024085	0.148943	0.04869	-0.16222	-0.10059	-0.04805	-0.06356	-0.13914	-0.07461	0.159877	0.038568
intake127	0.191684	-0.07841	-0.03652	-0.0088	0.036393	-0.04351	-0.03593	-0.12181	0.020624	-0.0509	0.043422	-0.12001	-0.03755
intake128	0.16623	-0.03752	-0.00258	0.042742	0.027999	-0.06321	-0.0706	0.119329	0.091219	0.152109	-0.02684	-0.12061	-0.03509
intake129	-0.00556	0.009654	0.198742	0.015232	0.01377	-0.00553	-0.16727	-0.12863	0.199136	-0.05737	0.056045	0.071286	-0.14913
intake130	0.039376	0.027183	0.139495	-0.14665	-0.05606	0.046446	-0.18823	0.098059	0.114967	-0.00628	-0.16351	0.065892	-0.17818
intake131	-0.15003	-0.0264	0.074111	0.093262	0.068523	-0.15336	-0.09679	-0.11059	-0.10113	0.070216	0.125472	0.039974	-0.12769
intake132	-0.09763	0.062761	-0.03073	0.177735	-0.10636	0.014255	-0.0807	-0.08579	-0.1112	-0.12154	-0.01656	-0.05977	0.051104
intake133	0.094606	-0.10192	0.090844	0.04423	-0.01863	-0.06592	0.003894	0.038738	-0.07727	0.032352	-0.08077	-0.00013	0.126578
intake134	-0.04211	0.046208	-0.05473	0.181819	-0.09219	0.032585	-0.0575	0.140662	-0.06527	0.170046	0.108874	-0.00213	0.020834
intake135	-0.10331	0.096897	-0.02337	0.148412	-0.0769	0.131022	0.047795	0.042831	-0.04193	0.074915	-0.04264	0.027562	-0.0369
intake136	0.008369	-0.15321	0.001064	-0.14482	-0.18225	-0.00738	-0.04404	-0.01147	0.110859	-0.07908	-0.04911	0.153608	0.096749
intake137	0.124428	-0.01741	-0.10735	0.074178	-0.05282	-0.01731	0.062256	0.029506	-0.01474	-0.08007	-0.16819	0.068331	0.085751
intake138	0.03572	-0.12008	-0.00937	0.100787	0.100089	-0.09823	0.042247	0.13855	0.009985	0.026627	-0.1828	-0.19441	0.097604
intake139	0.100503	-0.00141	0.160031	0.015617	-0.10686	0.162764	0.150823	-0.02253	-0.1172	-0.01079	0.066229	0.051751	0.18319
intake140	-0.07359	-0.03266	0.064622	-0.02559	0.041598	-0.12133	0.001049	0.127464	-0.10449	0.0052	0.134421	0.074711	-0.13313
intake141	-0.08952	-0.11645	0.145821	0.090307	-0.08544	-0.00914	-0.08232	0.045204	0.078128	-0.09605	0.012103	0.030975	0.00295
intake142	-0.00338	-0.15493	0.174618	0.002771	-0.06171	-0.04677	0.038185	0.081821	-0.0095	0.105325	-0.04045	0.047829	0.130913
intake143	0.097413	0.028859	-0.16192	0.089321	-0.11207	0.004452	0.066417	-0.03687	0.012139	0.097125	0.071324	-0.05981	-0.07577
intake144	0.076187	-0.01219	0.04518	-0.01899	-0.11477	0.057829	-0.14924	0.062946	-0.2554	-0.07602	0.008056	0.000753	-0.16723
intake145	0.104896	-0.16995	-0.00013	-0.01681	-0.06186	0.011137	-0.00078	-0.05261	0.066376	0.172458	0.071873	0.063577	0.088008
intake146	-0.04372	0.001387	0.005322	0.023564	-0.2159	0.065164	0.058272	0.183456	0.155256	-0.14843	-0.06204	-0.08535	0.070024
intake147	0.110235	-0.1647	0.065772	0.054038	0.043448	0.085285	0.00478	-0.04737	-0.09904	-0.06318	0.008978	0.005372	-0.015
intake148	0.017759	-0.0608	0.173829	-0.11006	-0.11832	0.026369	-0.02963	0.143718	0.023012	0.119291	-0.11843	-0.0122	-0.17672
intake149	0.084128	-0.10975	0.155805	-0.01888	-0.02592	0.095065	0.053941	0.051824	-0.08432	0.108855	-0.03256	-0.05305	-0.13095
intake150	0.202449	-0.06152	-0.04929	0.044881	0.026208	0.032738	0.075267	0.017533	0.025277	0.130559	-0.07111	-0.12919	0.111469
intake151	-0.0084	-0.06241	-0.0311	-0.03491	-0.0178	0.090357	0.045845	0.172715	-0.05415	-0.23982	0.161952	-0.10642	0.07225
intake152	-0.08145	-0.1324	-0.0047	0.120348	-0.12968	0.036858	-0.13269	0.123111	0.015032	-0.1202	0.047908	0.083105	0.042907
intake153	-0.01518	-0.00562	0.021246	-0.07721	0.048786	0.240429	-0.05337	0.138822	0.069569	0.036176	-0.03224	-0.03478	0.185301
intake154	0.079104	-0.16654	0.071604	-0.00802	-0.06281	-0.09158	-0.08617	0.064701	-0.06584	-0.09386	0.022852	-0.11751	-0.05247
intake155	0.032034	-0.11507	0.086749	0.000586	0.012483	-0.00254	0.027645	0.00796	-0.21008	0.149158	0.065226	0.052205	-0.0631
intake156	0.145279	0.012291	-0.07123	0.013441	0.081244	0.016315	-0.03079	0.159974	-0.08543	-0.06272	0.032786	-0.02663	0.163239
intake157	-0.02525	-0.14986	-0.06054	0.174954	-0.10075	-0.04787	-0.0369	0.165841	-0.09269	-0.04257	0.082064	-0.00543	-0.09743
intake158	0.035189	0.06107	0.107037	-0.01519	-0.14824	0.032218	-0.17392	-0.04382	-0.01714	0.039257	0.03103	-0.12893	0.204665
intake159	0.043073	-0.05552	0.091649	0.102829	0.016497	0.057063	-0.19043	-0.14759	0.096228	0.02947	0.02426	-0.1278	0.129535
intake160	0.056517	-0.1613	0.038189	0.061164	0.030555	0.044985	-0.00986	0.006643	-0.01819	0.179971	0.108118	-0.1962	-0.05755
intake161	0.041762	-0.10739	0.0075	-0.02176	-0.09558	0.15647	0.069341	0.187773	0.024033	-0.11839	0.022123	-0.22782	-0.02988
intake162	0.074188	-0.02328	0.013733	-0.20632	-0.06091	0.001958	-0.19712	0.11796	-0.02014	0.103086	-0.02218	0.074286	0.239966
intake163	0.04388	-0.17631	-0.02991	0.114359	-0.01586	0.089297	-0.12207	-0.00697	0.029122	0.093571	0.033337	-0.0783	0.081495
intake164	-0.00993	-0.18423	0.024506	-0.01925	0.020573	0.114337	-0.10314	0.098044	0.064187	0.102099	0.062715	0.105657	0.061667
intake165	-0.05649	-0.13751	0.135111	0.150305	-0.03523	-0.00973	-0.10464	-0.01804	-0.23768	0.050744	-0.04763	-0.06616	-0.06419
intake166	0.085606	-0.11326	-0.1576	0.001024	-0.04376	0.103096	-0.04034	-0.08752	0.038651	-0.06556	0.118928	0.071202	-0.02049
intake167	0.003737	0.057413	0.014332	-0.00856	-0.0337	-0.06727	-0.17588	0.177484	0.04544	0.148421	0.196941	0.097446	0.057074
intake168	0.19325	-0.02258	0.027703	-0.03979	0.105699	0.005112	-0.1085	-0.05081	-0.00714	-0.11182	0.014136	0.012778	-0.05333
intake169	0.195594	0.022903	0.035004	0.036438	0.030194	-0.09753	-0.07246	-0.0113	0.036082	-0.05889	-0.07164	-0.04436	-0.03295
intake170	0.088192	0.00513	0.036063	-0.13273	-0.08281	0.15015	-0.0859	-0.13608	-0.0153	0.004761	0.017952	0.03231	0.074262
intake171	0.064392	-0.1202	-0.10423	0.034553	0.084606	0.042624	0.145718	0.037451	-0.05529	0.124178	0.023733	0.035587	-0.052
intake172	0.115151	-0.06427	-0.12257	0.102567	0.124235	0.190165	-0.04067	0.00854	0.120241	-0.02944	-0.01093	0.104235	0.008279
intake173	0.02885	0.007521	-0.03681	-0.01988	-0.06274	0.094266	-0.00601	-0.0888	0.169061	0.176303	-0.12143	0.069508	-0.00295
intake174	0.086502	-0.02349	0.02895	0.015894	-0.00125	0.125054	0.06087	0.021734	0.242517	-0.04398	-0.03791	-0.00697	-0.14557
intake175	0.215428	0.020641	-0.04598	-0.14544	-0.06924	-0.13036	-0.04587	-0.0308	-0.05024	-0.06034	0.143728	0.030801	-0.10206
intake176	0.003062	0.07833	0.030156	0.029113	0.071726	0.096474	-0.21129	0.034358	-0.01181	-0.17181	0.031	-0.16994	-0.15678
intake177	0.073767	-0.13058	0.103217	0.082811	0.080509	0.101227	0.078049	0.013124	-0.06548	-0.1228	-0.05053	0.056587	0.015614
intake178	0.052615	-0.08346	0.077406	-0.01893	-0.16798	-0.01821	0.138569	0.009643	0.023304	0.029918	-0.11029	0.252156	0.025217
intake179	0.037654	-0.12878	-0.18524	-0.13744	-0.01141	0.168043	-0.00547	-0.14155	-0.06658	0.176084	-0.03011	-0.0435	-0.05613
intake180	0.072368	0.031165	0.039059	-0.20346	-0.00531	0.08137	0.015457	-0.10429	-0.00935	0.029941	0.056273	-0.05281	0.083498
intake181	-0.04917	-0.0609	-0.06899	0.010695	-0.05204	0.09464	0.015927	0.02342	0.047708	-0.07682	0.108397	0.013553	-0.05072
intake182	0.043295	0.031916	-0.01762	-0.23115	-0.05265	-0.01432	0.103945	-0.11181	0.063275	0.123609	0.213586	-0.02326	0.063831
intake183	-0.01962	-0.00441	-0.01147	0.0819	0.006878	0.174341	-0.0546	-0.09062	-0.07317	0.082915	0.064692	-0.06689	0.246922
intake184	0.080134	-0.06301	-0.06777	-0.02104	0.07684	0.018517	-0.03392	0.045588	-0.05887	-0.06222	0.104508	0.225838	-0.12403
intake185	0.003762	-0.07101	-0.00961	-0.1464	0.07132	0.256005	0.013423	0.058977	0.023747	-0.03235	0.082053	0.112946	-0.10439
intake186	0.019813	-0.04	-0.02478	-0.05334	-0.19644	0.14116	0.037481	-0.02465	0.00298	-0.08991	0.135835	0.0248	-0.04218
intake187	0.025373	-0.15761	0.050116	0.016541	0.014969	0.176814	-0.00595	-0.01165	-0.07492	-0.00833	-0.02242	0.015064	0.023553
intake188	0.019412	0.074083	-0.11776	-0.01057	-0.03614	-0.04929	0.057025	0.157726	-0.0938	-0.04852	0.059522	0.048644	0.029529
intake189	0.052729	0.030414	0.022814	0.077269	-0.22886	0.069249	0.067472	-0.04279	0.03712	0.078579	-0.08774	-0.09997	-0.13186
intake190	0.330154	0.396889	0.341907	0.241397	0.223091	0.088471	0.134629	0.110253	0.085665	0.085622	-0.0209	0.031778	0.068569
intake191	0.192008	0.124726	0.043805	0.136508	0.073114	0.140881	-0.0776	0.035303	-0.0213	0.020175	0.157464	0.155508	-0.02528
intake192	0.165963	0.087869	-0.06248	-0.07688	0.06754	0.000395	-0.07386	0.048529	-0.05753	-0.02957	0.062006	-0.09816	-0.0134
intake193	0.079396	0.162029	-0.04472	-0.13389	-0.08843	0.05927	0.009518	0.159902	0.089199	-0.03453	0.009344	-0.1276	-0.16869
intake194	0.05226	0.033881	-0.00758	-0.06975	0.074613	0.091978	-0.06862	0.043681	-0.2445	-0.21283	0.068831	0.056821	0.118508
intake195	-0.00999	0.07658	0.052361	0.098924	-0.19522	0.019351	0.033537	-0.08663	0.121034	0.015811	0.191061	0.075365	0.061957
intake196	-0.04906	0.046829	0.063111	0.192148	-0.03854	-0.01773	-0.05081	0.054542	-0.08877	0.043103	0.056042	-0.01216	-0.09108
intake197	0.113333	0.08496	-0.10626	-0.0299	-0.1303	0.012019	-0.17976	-0.0027	-0.0633	-0.01213	0.051254	0.057735	-0.02909
intake198	0.032961	0.080193	0.124539	-0.15986	-0.12224	0.025021	-0.00428	0.06586	-0.09746	0.015205	-0.26983	0.115311	-0.01432
intake199	0.073544	-0.03154	0.125205	-0.07448	-0.11779	0.004282	0.018003	-0.01984	0.087379	0.105952	0.07628	0.034598	-0.10306
intake1100	0.127742	-0.01137	-0.05974	0.008819	-0.12368	-0.04055	0.061573	0.011708	-0.19287	-0.05811	-0.10788	0.028124	0.088714
intake1101	-0.03668	-0.06292	0.065769	0.073602	0.067714	0.1222	-0.22585	-0.01407	0.148118	-0.01073	0.093369	0.009902	-0.02739
intake1102	-0.08343	-0.09244	0.167685	0.047816	0.005671	-0.00341	-0.06484	-0.09759	-0.12205	-0.0467	-0.0249	0.06866	0.075413
intake1103	0.047251	-0.01773	0.056507	0.133402	-0.2148	-0.07168	-0.03402	-0.1569	-0.0963	0.08907	0.146453	0.086443	0.024584
intake1104	0.134753	-0.09113	-0.02911	-0.00719	0.006763	-0.00014	-0.01398	-0.11244	0.074671	-0.08275	0.039512	-0.09645	0.061948
intake1105	0.051756	0.101788	-0.10489	-0.06399	-0.03214	-0.03996	-0.10839	0.153413	-0.071	-0.03716	-0.1355	-0.02272	0.115241
intake1106	0.093967	-0.06635	-0.08043	0.019381	-0.04409	-0.12332	-0.06233	0.092211	0.147977	0.01395	0.12005	-0.047	-0.03191
intake1107	0.123274	-0.0925	-0.08175	-0.00674	0.04157	-0.04024	0.021182	-0.11543	-0.01473	-0.13536	-0.16038	-0.07506	-0.06675
intake1108	0.03486	-0.17128	0.050103	0.102867	0.084298	-0.05784	-0.08037	0.100076	0.048257	0.063216	-0.06806	-0.09328	0.002192
intake1109	0.050298	-0.00276	-0.12932	0.071155	-0.09879	-0.13825	-0.06491	0.03531	0.017987	0.129567	-0.05567	0.012327	0.213983

	Factor14	Factor15	Factor16	Factor17	Factor18	Factor19	Factor20	Factor21	Factor22	Factor23	Factor24	Factor25	Factor26	Factor27
intake11	0.066608	-0.10447	0.102806	0.041439	0.117709	-0.00605	-0.11212	0.09026	-0.29142	0.062382	-0.04088	0.112083	-0.15603	-0.16744
intake12	-0.10744	-0.0971	0.040299	0.031256	0.104187	0.059106	0.031532	0.082448	-0.08127	-0.05667	-0.01218	0.060924	0.005685	-0.05366
intake13	-0.04349	-0.07783	0.076435	0.123031	0.048513	0.147519	-0.07328	-0.00071	0.112533	0.112201	-0.06859	-0.05329	0.026604	0.211328
intake14	-0.01372	-0.03875	0.018712	-0.00315	-0.04111	-0.08892	-0.13316	0.090552	0.07689	-0.08517	-0.09986	0.030591	-0.07038	-0.03047
intake15	-0.07441	-0.29515	-0.00378	-0.06302	0.07727	-0.19133	0.023433	-0.08787	0.16434	0.056786	0.007089	-0.06521	-0.02785	0.059194
intake16	-0.03508	-0.09099	-0.16441	-0.07344	-0.01532	0.092412	0.01758	-0.0259	-0.07137	0.007353	-0.14689	-0.01395	-0.07688	-0.1739
intake17	0.078241	0.008872	-0.17698	0.129105	0.097935	-0.06672	0.197241	0.031037	-0.22337	-0.14557	0.00761	0.069739	0.074931	0.1586
intake18	-0.10423	-0.09155	0.02578	0.159268	0.007616	-0.00432	0.028699	-0.12174	-0.04498	0.018557	-0.01415	0.087744	-0.19255	-0.09055
intake19	0.138706	-0.1119	0.020783	-0.06539	-0.00159	0.061894	0.018656	0.031315	-0.10054	-0.0923	-0.08449	-0.10891	-0.04277	0.017116
intake110	-0.02538	0.003071	-0.1261	0.05151	-0.03577	-0.00508	-0.07581	-0.12468	0.016692	-0.12456	-0.00753	0.005201	0.019972	-0.08669
intake111	0.103535	0.118916	-0.12994	0.003821	0.070136	-0.0235	0.016225	0.045649	0.001318	-0.02363	0.014425	-0.14366	0.095916	0.011126
intake112	0.14666	-0.17866	-0.01742	0.048934	-0.25324	0.127845	-0.04825	-0.0228	0.010033	0.016104	0.170297	-0.04154	0.227828	0.019603
intake113	0.028038	0.016077	-0.10953	-0.0012	0.099451	-0.08652	0.121869	0.095572	0.052473	0.063814	0.019522	-0.02693	0.033997	-0.00508
intake114	0.026052	0.059514	-0.02543	-0.02315	0.008621	0.079564	0.079001	-0.07013	-0.00042	-0.00568	-0.04139	0.151707	0.095037	0.217543
intake115	0.137099	0.038462	-0.05947	-0.00191	0.016707	-0.00165	0.046979	0.031965	-0.00182	-0.04777	0.038561	0.000108	-0.03044	-0.12271
intake116	0.020599	-0.03498	0.037721	-0.01005	-0.1666	-0.0487	0.149535	0.10242	-0.02124	-0.07677	0.048209	0.078088	0.059061	-0.08195
intake117	-0.02838	0.145294	0.002944	0.012446	-0.04329	0.16764	0.057046	0.107577	0.068662	-0.07992	-0.00786	-0.04723	-0.01617	-0.01772
intake118	0.056983	-0.00644	0.032462	-0.00454	-0.10019	0.134825	-0.03445	-0.06843	0.14893	-0.04825	-0.04653	0.155349	-0.15455	0.027077
intake119	0.052291	0.024799	-0.11006	-0.01229	0.101074	0.006657	-0.01204	-0.06066	0.072641	0.005119	-0.00744	0.053765	0.038154	-0.04071
intake120	0.003472	-0.01416	0.051336	-0.04886	-0.06267	-0.20551	-0.02931	0.009955	-0.07288	0.016918	-0.03639	-0.16634	0.02411	-0.03503
intake121	0.098264	0.063912	0.04824	-0.08203	0.048931	0.111848	0.012159	0.061911	-0.01773	-0.2022	-0.01563	-0.0762	-0.02648	0.117351
intake122	0.021243	0.068857	-0.05469	0.015321	0.109576	-0.13766	0.033085	0.136265	-0.00165	0.181858	-0.08574	0.071889	0.006652	0.023965
intake123	0.031743	-0.15513	-0.02742	0.055001	0.127951	-0.08629	-0.00638	0.056847	-0.02658	0.083963	-0.06878	-0.02718	-0.0289	-0.09301
intake124	0.036791	0.034561	-0.09943	0.022448	-0.01929	-0.14504	-0.11923	-0.07087	0.063124	-0.03478	0.036554	0.056273	0.129261	-0.06553
intake125	0.041502	-0.02294	-0.12653	0.003554	-0.08357	-0.11465	-0.05283	-0.24773	0.060105	0.156244	0.212701	-0.10899	-0.01198	-0.17314
intake126	-0.04298	0.102808	-0.01031	-0.01183	0.08407	-0.01125	0.03959	0.179301	0.002766	0.055228	-0.01647	-0.16655	0.026462	-0.03195
intake127	0.04033	-0.01064	0.008823	-0.09635	-0.08373	-0.06059	-0.04603	0.020756	-0.01105	-0.05877	0.01883	0.012774	-0.07464	-0.01673
intake128	-0.05252	0.227439	0.11734	-0.05969	0.091037	0.072926	-0.0208	-0.13141	-0.00811	0.084931	0.112018	-0.10587	-0.07418	-0.04111
intake129	0.207062	-0.00407	0.002058	-0.0637	0.041262	0.09095	-0.0569	0.095608	0.017406	-0.07998	0.0487	0.018516	0.165862	0.10094
intake130	-0.02539	-0.17662	-0.03905	-0.0527	-0.0787	-0.12813	-0.11024	0.036975	-0.0268	-0.08241	0.016225	-0.07244	0.151351	0.079364
intake131	0.188666	0.008964	-0.10668	0.041098	-0.12135	0.049748	-0.10678	0.025871	-0.11242	0.111334	-0.1589	0.00708	-0.01385	-0.01573
intake132	-0.00085	0.086694	-0.03478	-0.0162	-0.02491	0.085748	-0.02464	0.073196	0.09481	0.003625	-0.12532	-0.15573	0.139037	-0.03737
intake133	0.118594	-0.15193	-0.01754	-0.18777	0.045332	-0.05347	-0.03754	0.098864	-0.0416	-0.02781	0.168307	-0.05281	-0.10968	-0.06943
intake134	0.076657	0.02805	0.196432	0.038285	0.026424	-0.10047	-0.0871	0.045778	-0.00417	-0.11216	0.060242	-0.18142	-0.06066	-0.10988
intake135	0.060013	-0.02641	0.128284	0.094614	0.023185	0.074347	-0.06526	0.019121	0.043316	-0.00665	-0.01294	0.096946	-0.00565	0.033266
intake136	-0.05753	0.065603	0.034341	-0.08097	-0.00766	0.170888	-0.06678	0.078765	0.107883	0.151615	-0.14933	-0.01331	-0.22831	-0.15909
intake137	0.183962	-0.10213	0.040544	-0.15724	-0.04924	-0.00739	-0.01556	-0.17214	0.19848	0.046846	0.014577	0.035226	0.024626	0.11353
intake138	0.136212	-0.06167	-0.12131	0.083075	0.010702	-0.00944	0.015506	0.009938	0.078493	-0.09873	-0.1232	0.049238	0.046435	-0.12988
intake139	0.098677	-0.03133	-0.01747	0.081098	-0.00389	-0.11556	0.084546	0.045179	0.033312	-0.06204	0.053758	-0.00066	-0.11224	0.016329
intake140	0.172706	-0.07281	0.06921	-0.11589	0.126933	0.067739	0.019789	-0.09008	0.231008	0.153425	-0.116	-0.00719	0.094675	0.078835
intake141	-0.06841	-0.08574	-0.15754	-0.0367	0.075372	-0.12555	0.051094	-0.12026	0.06614	-0.24818	0.026182	-0.02955	-0.01822	-0.09073
intake142	0.081301	-0.00506	0.071189	0.142471	0.016869	-0.05499	0.011795	-0.04647	0.041133	-0.03545	-0.05417	0.054638	-0.09186	-0.00279
intake143	0.141695	-0.13774	-0.07861	-0.07478	-0.07699	0.110163	-0.08846	-0.01194	-0.1465	-0.0369	-0.09354	0.016343	0.014411	-0.00622
intake144	-0.08683	-0.05685	0.012937	-0.137	-0.04477	0.063168	-0.09449	-0.05611	-0.00445	-0.0366	0.123547	-0.08405	-0.04776	0.016491
intake145	0.172846	0.114772	0.092112	0.084388	-0.08752	0.044125	-0.15748	0.003408	0.104675	0.003233	-0.10339	-0.06363	0.00823	-0.06325
intake146	0.115707	-0.01569	-0.11246	-0.06448	-0.10494	0.084603	0.089238	0.188083	0.063465	0.167424	0.282802	0.045036	-0.00807	-0.18454
intake147	-0.0087	-0.04176	0.139745	-0.04425	0.045395	0.042042	0.084366	0.012435	-0.05486	0.046866	-0.00449	-0.14547	-0.02434	-0.04451
intake148	-0.01749	0.013557	-0.08845	-0.16824	0.012752	0.077432	-0.04913	0.007499	-0.07885	0.080754	-0.05705	-0.1418	-0.02187	0.105261
intake149	-0.02908	0.024151	-0.09769	-0.0201	0.087281	0.152531	0.082814	0.023489	0.120967	0.114429	0.062132	0.009213	0.049706	-0.05848
intake150	-0.00084	-0.08388	0.021104	0.029982	0.083384	0.12555	0.116354	0.083676	0.049529	0.043223	-0.00023	0.01765	-0.03457	0.041547
intake151	0.106438	-0.02088	-0.00926	0.173896	-0.03922	-0.01697	-0.12718	-0.07695	0.0526	-0.05998	-0.09334	-0.15161	-0.09632	0.079862
intake152	-0.01808	-0.0394	0.009445	0.154348	0.232404	-0.09969	-0.11061	0.071743	0.067812	0.048067	0.068134	0.120222	0.031583	-0.04458
intake153	-0.0463	-0.04182	0.077521	-0.08921	0.00035	-0.12015	-0.00268	-0.02249	-0.06088	0.30174	0.005194	0.215496	0.142102	0.008959
intake154	0.013978	-0.02087	0.054514	-0.03672	-0.06795	-0.03096	-0.03279	0.027311	0.095873	-0.02574	0.032715	0.004847	0.107458	-0.01111
intake155	0.207409	-0.07257	-0.05936	0.040477	0.010002	0.112165	-0.03106	0.003732	-0.08322	0.073795	-0.02516	0.041635	0.108714	-0.07297
intake156	-0.11705	-0.06184	-0.02422	-0.08737	-0.15989	0.024428	-0.02752	0.104637	-0.08166	-0.08452	-0.05067	0.009659	0.193101	-0.19801
intake157	-0.08565	-0.00136	0.111919	0.089251	0.085824	0.022133	-0.0906	0.048803	0.032657	-0.052	0.126992	0.044306	0.147538	-0.00096
intake158	6.71E-05	0.017401	0.099956	-0.08867	-0.05205	-0.02491	-0.04696	-0.26622	-0.11986	-0.08086	0.049715	0.245597	0.156771	-0.05597
intake159	-0.14746	-0.09669	-0.10825	0.021375	0.088057	0.008896	-0.09944	-0.01924	-0.08366	-0.03952	-0.28747	-0.05441	0.074403	0.010297
intake160	-0.011	0.038749	0.020994	0.047539	0.127772	0.106051	-0.0039	-0.10648	-0.22668	0.140331	0.156751	-0.05077	0.074712	0.129638
intake161	0.104471	0.044772	0.015947	-0.00471	-0.01678	0.199799	0.094603	0.049362	0.123411	-0.0445	-0.14894	0.060671	0.14861	0.03367
intake162	-0.08678	0.017236	-0.00341	0.038019	-0.02172	0.106627	0.000446	-0.0334	0.128835	0.061064	0.002755	-0.06173	-0.02497	0.06615
intake163	-0.15785	0.047407	-0.02662	0.003818	-0.02565	0.043883	-0.09465	-0.12856	-0.01318	-0.11846	-0.07853	-0.02136	0.268934	0.069581
intake164	0.033832	0.202369	-0.02046	-0.05009	-0.13289	0.083801	0.0574	0.030877	-0.05904	-0.02369	0.026472	-0.1043	-0.10973	0.130659
intake165	0.048555	0.192199	0.009475	-0.01034	-0.26186	-0.03363	-0.05607	-0.0302	-0.09352	0.079919	0.061994	0.005313	-0.05641	-0.09463
intake166	-0.07918	0.03322	-0.08032	-0.01374	0.090049	-0.0787	0.006622	-0.07601	-0.1572	0.013724	0.154708	0.185773	0.016688	0.105304
intake167	-0.03014	0.122167	-0.07335	0.125847	0.086188	-0.10434	-0.03658	-0.14957	0.084661	0.079282	-0.12908	0.150149	-0.08566	-0.08336
intake168	0.006292	0.046434	0.170357	0.237262	-0.04709	0.030844	0.035483	0.009432	0.05198	0.037747	-0.03997	-0.12734	-0.09248	0.044752
intake169	-0.0221	0.023058	-0.10041	0.155876	-0.1487	0.115267	-0.15298	-0.029	0.079147	0.091715	-0.03091	0.210582	-0.07664	-0.17359
intake170	-0.00826	-0.0678	0.133711	-0.02056	-0.01973	0.067506	0.156611	0.098518	-0.10436	0.161985	-0.0746	0.001369	0.062683	########
intake171	-0.2611	-0.11902	-0.01629	0.199163	-0.01573	0.013643	-0.19512	0.161541	-0.04674	-0.16558	-0.00442	0.027824	-0.03216	0.089051
intake172	-0.09736	-0.04646	-0.13105	-0.07846	-0.05307	0.020955	-0.16991	0.036814	0.121946	0.004485	-0.02866	0.07351	0.030236	0.238059
intake173	0.081238	-0.20273	0.089537	0.179011	-0.035	0.055828	-0.0389	0.02466	-0.03778	0.003	0.178042	-0.13251	0.108087	-0.15262
intake174	-0.0963	0.080598	-0.00549	-0.19643	-0.13591	0.060895	-0.06432	0.013935	0.037737	-0.02788	-0.16037	0.120156	-0.08894	-0.09195
intake175	-0.1371	0.049775	0.109081	0.149776	-0.10909	0.039151	-0.01251	0.048116	0.006249	0.11277	0.162474	0.075291	0.003387	-0.05724
intake176	0.060343	-0.05182	0.058973	0.246066	-0.03288	-0.00208	0.119587	0.1367	0.055633	-0.02173	0.002679	0.021506	-0.0707	-0.00093
intake177	-0.05816	0.143895	-0.05209	0.032439	-0.00267	0.001002	0.041598	0.053554	-0.09553	0.080139	-0.14618	-0.11171	0.019148	-0.04703
intake178	0.161676	0.130331	0.12097	0.136322	-0.15067	-0.10715	-0.00922	-0.06347	-0.05351	-0.09438	0.041791	0.07696	0.064475	0.041207
intake179	0.190587	-0.09474	-0.10255	-0.00441	-0.08952	-0.14298	0.188246	0.117226	-0.05412	-0.05409	-0.0831	0.01318	-0.07654	0.008695
intake180	0.013486	0.166765	0.228107	-0.09517	0.027608	-0.10598	-0.02056	0.056123	0.025819	-0.18725	-0.08876	-0.1242	0.149236	-0.21757
intake181	-0.06878	0.1213	-0.01141	-0.09652	-0.0243	-0.1834	0.222163	0.131454	-0.10146	0.128701	-0.15721	0.065807	0.205179	-0.1121
intake182	0.004113	0.025792	-0.06556	-0.09413	0.137724	-0.02242	-0.18262	0.22735	0.129019	-0.13774	0.157717	0.069535	-0.01053	-0.05988
intake183	0.019592	0.005491	-0.22132	0.130919	-0.01101	-0.03892	0.213827	0.061022	0.019236	0.010895	-0.03366	-0.02428	-0.07757	0.052264
intake184	-0.18546	-0.06555	-0.09213	0.01291	-0.07755	0.178998	0.332334	-0.1533	-0.01015	-0.17665	0.06677	-0.05136	-0.0943	-0.02343
intake185	-0.0482	-0.13695	0.066007	0.084211	0.067503	0.150759	0.017057	-0.05669	-0.0655	-0.00627	0.056919	-0.10325	0.096204	-0.10116
intake186	0.124965	0.096828	-0.08266	-0.05806	0.15769	0.062395	0.011321	-0.03821	-0.06691	0.027805	-0.06235	0.050816	0.100008	-0.05297
intake187	0.06365	0.076556	0.008352	-0.07183	-0.16132	-0.15175	0.000831	0.111403	0.045581	-0.06612	-0.01333	0.106665	-0.10334	0.211853
intake188	-0.00391	-0.19173	-0.06005	0.064665	-0.16348	-0.11167	-0.22615	0.098236	-0.11999	0.240058	-0.08561	-0.03728	0.05266	0.098864
intake189	-0.11648	0.056044	0.087282	-0.01994	-0.02068	-0.0171	-0.01816	-0.05844	-0.1081	-0.01196	0.029439	-0.06928	-0.16368	0.001052
intake190	0.023467	0.069299	-0.04782	-0.02583	0.038297	0.005763	0.044629	-0.00466	-0.01267	0.005265	0.05387	0.002065	0.04729	-0.02192
intake191	0.047275	-0.09581	0.049473	-0.01796	-0.11764	-0.02877	0.04502	-0.08464	-0.10262	-0.04128	-0.0509	0.062849	-0.00011	-0.09781
intake192	0.127109	0.015039	0.071995	-0.03346	0.059966	-0.05657	-0.02711	0.068898	0.134902	0.043299	0.032914	0.118926	-0.09001	0.076989
intake193	0.126692	0.124866	-0.21298	0.162003	-0.05132	-0.02757	-0.13364	-0.08873	-0.16603	0.068254	-0.12834	-0.09631	-0.11739	0.007069
intake194	0.113467	0.057931	-0.11122	-0.15135	0.300258	0.12268	-0.20513	0.023003	-0.14923	-0.05436	0.115061	0.014209	-0.11766	-0.04717
intake195	-0.07344	0.038033	-0.02693	-0.06038	-0.11777	-0.10073	-0.09622	-0.00432	0.032472	0.20332	0.048039	-0.259	-0.00723	0.110709
intake196	-0.10098	-0.03132	0.183243	-0.24812	-0.0394	-0.01472	0.015692	0.167572	-0.02542	-0.01412	-0.06198	0.233474	-0.10283	0.017679
intake197	-0.04982	-0.04603	0.245831	-0.09533	0.040639	-0.19865	0.046182	-0.11361	0.00597	0.000745	-0.05021	-0.04922	-0.08029	0.041131
intake198	-0.04017	0.165717	0.019899	0.112044	-0.12467	-0.07976	-0.07643	0.115398	0.02124	-0.13554	-0.01976	0.061213	0.116268	0.110729
intake199	-0.08069	-0.00131	-0.0892	0.069281	0.090676	-0.10994	-0.11096	0.130058	0.081608	-0.15049	-0.06575	-0.05941	-0.00052	-0.06972
intake1100	0.080881	-0.15949	0.171253	0.003203	0.106749	-0.00535	0.04108	-0.04439	-0.13041	0.025512	-0.20387	-0.04939	-0.00252	0.164066
intake1101	0.158836	-0.10667	0.034196	0.025204	0.024864	-0.10722	0.079791	0.108298	0.197755	-0.01731	0.070489	-0.0003	-0.1052	0.012524
intake1102	-0.12341	0.053872	-0.0471	0.147945	-0.0212	0.040565	-0.01441	0.286332	-0.04915	0.113266	0.147152	0.098064	0.031949	0.128452
intake1103	-0.14463	-0.11368	-0.1936	-0.00753	-0.01596	0.061602	0.040973	-0.04827	0.214437	0.018061	-0.04898	0.049039	0.021553	-0.08171
intake1104	0.041763	0.070475	-0.12555	-0.05135	0.087446	0.020927	-0.12106	-0.02438	-0.09832	-0.06418	0.161951	-0.00673	-0.14027	0.180814
intake1105	-0.13783	-0.09167	-0.12379	-0.10521	-0.11099	0.00259	0.019923	0.160863	0.038089	0.05808	0.051477	-0.21157	0.019951	0.018744
intake1106	-0.02612	-0.00202	-0.03151	0.089983	-0.08905	-0.11339	0.207064	-0.13799	0.015835	0.01873	0.033726	-0.04685	-0.0391	0.079448
intake1107	0.015876	0.1438	0.08082	0.154222	0.207946	-0.05017	0.003473	-0.04845	0.103526	0.05861	0.010827	-0.06465	0.210956	-0.10578
intake1108	0.025539	0.114405	-0.073	-0.03562	-0.05518	-0.10879	-0.02636	0.00665	-0.05087	-0.00019	0.133148	0.000601	-0.00653	0.063664
intake1109	0.029677	-0.10553	0.121092	0.067535	-0.05521	0.23429	0.014586	0.048027	-0.12539	-0.09743	0.044765	0.046854	-0.01013	0.003605

	Factor28	Factor29	Factor30	Factor31	Factor32	Factor33	Factor34	Factor35	Factor36	Factor37	Factor38	Factor39
intake11	0.099384	-0.03917	0.078718	0.043123	-0.03261	0.149318	0.117834	-0.05507	-0.05793	-0.0583	0.175526	-0.06695
intake12	-0.05844	0.026118	-0.00909	-0.05258	-0.04093	-0.01806	0.115478	-0.02186	-0.03119	-0.01835	-0.13114	-0.00748
intake13	0.032888	-0.05249	0.002699	0.166799	-0.01176	0.046887	-0.17021	0.039327	0.051806	0.114254	0.044077	-0.08555
intake14	-0.10631	-0.02795	-0.15821	0.011244	-0.06972	-0.12343	0.036231	0.148692	-0.10415	0.02623	0.015271	-0.08254
intake15	0.018094	0.031798	########	-0.10344	-0.0181	-0.00828	0.034455	-0.09167	0.038324	-0.09244	0.12391	-0.14899
intake16	0.059261	0.159949	0.068008	-0.10732	0.034089	0.022005	-0.1873	-0.07008	-0.09351	0.033611	0.02356	0.055505
intake17	-0.10177	0.050214	0.141883	-0.03838	-0.04757	-0.01326	-0.19234	-0.00789	0.16807	-0.15846	-0.1189	-0.0826
intake18	-0.03591	0.001289	-0.20452	-0.10226	0.065085	-0.31423	-0.04013	-0.00414	0.035568	-0.02796	0.191982	0.00885
intake19	0.04029	0.009453	0.050113	-0.01191	-0.02583	-0.04037	0.02402	-0.00362	0.122701	0.106991	-0.06768	-0.03537
intake110	0.049423	-0.00944	0.079874	0.049846	0.025887	-0.01122	-0.01481	-0.03731	-0.24064	-0.13376	-0.03858	-0.08737
intake111	-0.02069	0.003422	0.08913	-0.03541	-0.08881	-0.01483	0.016217	0.024766	0.061919	0.04895	-0.00389	-0.07583
intake112	-0.1432	0.186146	-0.00211	0.030451	0.154736	-0.11097	0.033532	-0.19814	-0.07439	-0.05325	-0.08844	-0.15101
intake113	-0.03164	-0.04416	-0.14959	-0.04119	-0.07145	0.116553	-0.04256	0.093846	0.058951	-0.00835	-0.03643	0.022167
intake114	0.071568	-0.17163	-0.10356	0.039693	-0.05489	-0.03179	-0.00774	-0.09116	-0.12957	0.091461	0.123849	0.031209
intake115	-0.0035	-0.01912	-0.1453	0.067263	0.088189	0.064151	-0.07061	0.026291	-0.1226	-0.13261	0.026966	0.192863
intake116	-0.03998	0.010535	0.032768	-0.02982	-0.07533	0.077391	-0.01516	-0.06383	0.019136	0.034422	0.106825	-0.06235
intake117	0.028526	0.09571	0.11891	0.011848	0.050476	0.161078	0.048274	-0.05678	-0.07568	0.149481	0.040802	-0.05713
intake118	-0.12552	0.028484	-0.01165	-0.02556	-0.09588	-0.17215	-0.07203	-0.0489	-0.02082	-0.002	0.119637	0.115767
intake119	-0.02984	0.010059	-0.05591	-0.04799	-0.02979	-0.09094	0.01038	0.052037	0.076974	0.076142	0.000459	0.089595
intake120	-0.10753	-0.05412	0.020404	0.121919	-0.13894	0.035756	0.116423	0.083217	0.055155	-0.04955	-0.05352	0.075193
intake121	0.070113	-0.12568	-0.13951	0.049925	0.13191	-0.0447	0.018958	-0.05485	-0.00896	-0.0295	0.088313	0.157477
intake122	-0.08102	0.092196	0.019123	-0.03319	-0.04969	0.113908	0.075873	0.114448	-0.09002	0.009164	-0.0705	-0.06824
intake123	-0.06087	-0.07587	0.000602	0.095213	-0.05014	-0.0734	0.049266	0.011118	0.013447	0.166078	-0.02963	-0.01945
intake124	-0.1442	0.130274	-0.06422	-0.05538	0.050135	-0.08069	-0.13011	0.042436	-0.02706	0.294479	-0.07153	-0.05066
intake125	0.185424	-0.05647	0.154108	0.046322	-0.13844	0.072935	0.016247	-0.24996	0.080578	0.085662	0.011443	-0.04879
intake126	0.036844	-0.06481	0.086204	-0.15563	-0.02679	-0.09135	0.103045	-0.00153	-0.09313	-0.07331	-0.00046	-0.13156
intake127	-0.1063	0.044213	0.103085	0.161198	0.031741	0.025299	0.146675	0.119181	0.087489	0.064007	-0.0735	-0.07905
intake128	0.065225	-0.04365	-0.05546	0.061174	0.032205	-0.1325	-0.22436	-0.0549	0.019263	-0.05605	-0.08609	0.081095
intake129	0.012164	0.054569	-0.08611	-0.12145	-0.12867	-0.02383	0.013015	-0.15787	-0.05376	0.016716	-0.10229	0.102258
intake130	0.000443	0.074372	0.08497	0.057091	0.001399	-0.00752	-0.00255	-0.01364	0.001593	-0.08142	-0.00568	0.208936
intake131	0.096539	-0.04309	0.046436	0.043573	0.060993	0.013221	0.076925	0.060329	0.022577	0.05622	-0.00379	0.06148
intake132	-0.07827	-0.00083	0.071501	-0.06076	-0.05074	0.071777	0.080381	-0.0229	0.068382	0.079641	0.021392	0.050308
intake133	0.003652	0.015463	0.025547	0.014018	0.036639	0.001094	-0.11372	-0.06095	-0.05241	0.115711	-0.09381	0.135608
intake134	-0.12799	0.022141	-0.02858	0.018195	0.044493	0.094559	-0.06229	-0.01285	0.083798	0.06933	0.042655	-0.0381
intake135	-0.05152	0.079491	0.050151	-0.11364	-0.05072	-0.00854	-0.0237	-0.02734	-0.00295	-0.02302	-0.00069	-0.07626
intake136	0.017974	-0.11509	0.004807	-0.00418	0.035293	0.094003	-0.14536	0.034467	0.052057	0.090884	-0.13932	-0.00288
intake137	-0.05456	-0.17884	0.114393	-0.15883	0.113746	0.008777	-0.01298	-0.01845	0.042964	-0.04535	-0.02508	-0.10571
intake138	0.231118	-0.07968	0.048389	0.0548	0.020184	0.032521	0.003835	0.045413	-0.02185	-0.03463	-0.19448	-0.16294
intake139	-0.17135	0.016861	0.150965	0.165295	0.075833	-0.01935	-0.05218	-0.00258	-0.00164	0.014104	-0.088	0.043695
intake140	-0.09764	-0.09016	0.05519	0.253177	0.063316	0.009634	0.052511	-0.08349	0.023576	0.032089	0.096885	-0.10732
intake141	-0.09772	0.018178	-0.03129	-0.07342	0.012607	0.180164	0.092828	-0.02262	0.013682	0.05287	0.125456	-0.11954
intake142	0.092955	-0.0159	0.003416	-0.05645	0.049738	0.055988	0.144561	0.002916	-0.05449	-0.20133	0.008328	-0.11041
intake143	0.055479	0.029443	0.059537	-0.10193	0.048912	-0.1467	-0.10768	-0.0648	0.137761	-0.02688	-0.0139	-0.01239
intake144	0.099796	-0.05016	-0.09382	0.032313	0.020543	0.122911	-0.16178	0.070564	-0.03546	-0.10069	0.026053	0.073987
intake145	-0.13734	-0.05398	-0.09923	-0.12267	-0.14741	-0.07149	-0.09795	0.032825	0.059989	-0.06204	0.023789	0.0135
intake146	-0.11729	-0.01643	-0.08784	-0.05578	0.156473	-0.04074	-0.0494	0.04163	-0.03724	-0.02801	-0.11846	-0.01197
intake147	0.011193	0.027468	-0.01894	-0.03397	0.013788	0.028956	0.004471	0.023521	0.065138	-0.00028	-0.04229	0.025795
intake148	-0.04062	-0.10904	-0.0407	0.043454	-0.02999	-0.07338	0.072502	-0.05892	-0.10927	0.093838	-0.047	-0.03662
intake149	0.017296	0.021336	-0.02402	0.083903	-0.04066	-0.01396	0.021316	-0.14403	-0.02442	-0.1735	0.018968	-0.00227
intake150	0.029658	0.076267	-0.02036	-0.12112	-0.00276	0.125155	0.014088	0.044368	-0.01034	-0.0153	0.028974	-0.00664
intake151	0.043586	0.317387	0.010055	-0.06683	-0.03105	-0.08192	-0.03134	0.077853	-0.12905	0.034138	-0.01486	0.097884
intake152	-0.05051	-0.03507	-0.0372	0.072208	-0.15901	-0.04746	0.002571	-0.01932	-0.08571	-0.00842	-0.08397	0.030698
intake153	-0.00738	0.147631	0.049758	0.029589	-0.07977	0.037058	-0.04744	0.050653	0.100198	0.021996	-0.0116	0.032683
intake154	-0.08723	-0.00807	-0.20333	-0.00975	-0.08349	0.059548	0.038897	0.013473	0.084726	-0.12204	-0.01996	0.039389
intake155	0.03387	-0.00168	0.074614	-0.09632	-0.20677	0.080862	-0.06613	0.058422	-0.14168	-0.01576	0.175164	0.002563
intake156	0.066185	-0.06283	0.027058	-0.05615	0.095722	0.066982	-0.06767	0.016545	0.049777	-0.00524	0.173649	0.057018
intake157	0.181249	0.203912	0.131868	0.031529	-0.04333	-0.05494	-0.02132	0.059951	-0.05181	0.068774	0.00682	0.042682
intake158	-0.04525	-0.25014	0.05101	-0.04256	0.024252	0.031541	0.099402	0.168234	-0.17954	0.150203	-0.2241	-0.01585
intake159	-0.01839	0.162168	-0.08667	0.08543	0.152504	0.072692	-0.16157	-0.06656	0.018979	-0.07527	0.109629	-0.07823
intake160	-0.06956	0.038499	0.099624	0.020508	0.106831	0.025133	0.14304	0.101594	0.058065	0.127937	-0.00899	0.149054
intake161	0.04281	0.05976	0.09069	0.021286	0.080217	-0.00064	0.119486	0.088847	0.022515	0.085779	-0.0645	-0.00234
intake162	-0.04564	0.058597	-0.25231	-0.12153	-0.14227	0.078043	0.111581	0.010984	0.232391	0.126472	-0.01316	-0.08238
intake163	0.04198	-0.05136	-0.0781	-0.18799	-0.04193	-0.0328	-0.03884	-0.01822	0.019642	-0.13545	-0.07271	0.035747
intake164	-0.11391	0.011359	0.110848	-0.01134	0.025878	0.053285	0.068304	-0.03053	-0.17856	-0.08255	0.031999	-0.21835
intake165	-0.09177	-0.0811	0.003125	0.022723	0.007935	-0.06821	-0.00491	0.027614	0.052442	-0.02529	-0.13627	-0.03433
intake166	-0.23313	-0.06887	-0.02087	0.070582	0.02032	0.199044	-0.15269	0.060429	-0.07343	-0.06845	0.089047	-0.03806
intake167	-0.07518	0.092716	0.214834	0.069067	-0.03789	-0.27767	-0.00702	-0.06696	0.1181	-0.14905	-0.07637	-0.00015
intake168	-0.03176	0.072488	0.155426	-0.12604	0.008576	0.021323	-0.15452	-0.03025	-0.07926	-0.02554	-0.09445	0.045212
intake169	-0.00955	0.092722	0.095395	-0.05493	0.03981	0.137654	0.124849	0.035339	-0.01897	0.111031	0.117227	0.034335
intake170	0.151898	-0.07879	0.075746	0.066815	0.091672	-0.16947	-0.08721	0.151084	-0.27474	-0.01834	0.046879	-0.08002
intake171	-0.00254	-0.26249	0.070957	-0.02095	-0.07378	-0.09831	0.020118	-0.20167	0.019727	0.086476	-0.19071	-0.12657
intake172	0.053313	-0.05817	0.177337	0.024363	0.078054	0.069153	-0.07106	0.007839	0.056432	-0.13323	-0.01844	-0.02452
intake173	-0.0555	0.091626	-0.09612	0.073909	-0.08072	-0.0483	0.041564	-0.02455	0.070779	-0.01456	0.044884	-0.1104
intake174	-0.10121	0.178563	0.089189	0.225387	-0.18824	0.109146	0.070841	-0.17671	0.013052	-0.04959	-0.11816	0.107165
intake175	-0.00398	-0.01672	0.074937	-0.10591	0.030977	0.038677	0.033656	-0.10803	0.021041	-0.09128	0.150474	0.081513
intake176	0.090231	-0.24468	-0.01785	0.144326	-0.22047	0.034114	-0.08241	0.14341	0.107762	0.009871	0.046637	-0.03951
intake177	-0.10626	0.048208	-0.11148	0.104225	-0.04345	-0.05565	0.105055	0.021681	-0.10349	-0.09917	-0.12718	0.098406
intake178	0.226543	-0.00831	0.032151	0.08145	-0.03105	-0.05433	0.098373	0.041197	0.110987	0.056214	0.010543	0.126013
intake179	-0.03775	0.079705	0.039234	0.118609	0.002003	0.035574	-0.05708	0.017286	0.019424	-0.02214	0.032702	-0.0137
intake180	-0.05722	-0.1642	0.034236	0.092965	-0.08334	0.073362	-0.16059	-0.25381	0.006309	-0.05569	0.053396	-0.00789
intake181	0.057963	0.010807	-0.07972	-0.20612	0.087657	-0.29289	0.096187	-0.10057	-0.01373	0.025404	0.023446	-0.04701
intake182	0.210468	0.126066	-0.08006	0.045933	-0.06637	-0.07415	0.128877	0.079107	-0.14004	-0.12063	-0.13999	-0.02939
intake183	0.127204	-0.05149	-0.07172	-0.04457	-0.07789	0.053939	0.120494	-0.37526	-0.0742	0.19662	0.018321	0.223344
intake184	0.067376	0.067912	-0.0096	0.093201	-0.00372	-0.05143	-0.00035	0.041976	0.116765	0.050557	-0.28815	-0.13255
intake185	-0.0714	-0.09849	0.086623	-0.15058	0.030972	0.061394	0.207173	0.049471	0.030854	0.125014	0.062643	0.037611
intake186	0.172971	-0.04306	0.102114	0.01448	-0.14346	-0.02439	-0.07045	-0.01744	0.294422	0.011454	0.08529	-0.08655
intake187	0.013849	-0.03945	0.025464	0.018304	0.109329	-0.09365	0.15343	0.008313	0.102686	-0.12058	0.237733	-0.05778
intake188	0.056968	-0.17416	-0.0737	0.046085	-0.01056	0.014051	-0.04106	-0.19973	-0.08586	0.017906	-0.17066	0.078168
intake189	0.152292	-0.02255	0.182213	-0.07616	-0.01803	0.039001	0.091637	-0.07608	-0.01379	-0.11346	-0.03552	-0.05381
intake190	-0.04271	-0.00436	0.046688	-0.00361	-0.00645	-0.01245	-0.01277	0.012147	-0.02917	0.015413	0.08162	0.055846
intake191	0.205712	0.007706	-0.27062	0.074414	0.019819	0.042522	0.042711	0.083271	0.113676	-0.07128	-0.08757	-0.15338
intake192	0.061741	-0.00772	-0.04527	-0.0969	0.106614	0.016406	0.240506	-0.17486	0.155444	-0.09105	-0.14897	0.094374
intake193	-0.0372	-0.06069	-0.23344	-0.02049	0.192252	0.098008	0.015716	-0.03725	0.042359	0.075135	-0.01242	-0.15106
intake194	-0.10361	0.024867	-0.00262	-0.021	0.033539	-0.07681	0.150051	-0.14155	0.009356	-0.0822	0.09377	-0.14268
intake195	0.097739	0.054635	-0.03216	-0.0477	0.001698	-0.00308	0.070342	0.10127	-0.04963	-0.03601	0.051674	-0.22353
intake196	-0.04577	0.083027	-0.03263	-0.05593	-0.06714	-0.06356	-0.14154	-0.15141	0.006545	0.309651	-0.00223	-0.24752
intake197	0.165431	0.188256	-0.04365	0.026487	0.188629	0.03109	0.119714	-0.06312	0.014643	0.126116	-0.04056	0.11472
intake198	0.050588	0.13187	-0.0726	0.057291	-0.04047	0.138776	0.027106	0.004721	-0.12081	0.128934	0.085204	-0.12613
intake199	-0.09817	-0.17484	0.196959	-0.07659	0.341134	-0.10173	0.02235	0.105446	0.106084	0.170211	0.071749	0.177511
intake1100	-0.1055	0.096421	-0.02199	0.011197	-0.02232	0.093693	0.01542	-0.05098	-0.07079	-0.04176	-0.11015	0.101063
intake1101	0.107519	-0.08256	0.061423	-0.13978	0.107156	0.011792	-0.17748	0.019398	-0.15622	0.036503	-0.07058	-0.03733
intake1102	-0.01583	0.026829	-0.05401	0.041609	0.216379	0.129142	-0.07806	-0.10393	0.223474	-0.05005	-0.09314	0.020512
intake1103	0.03958	-0.06394	-0.08668	0.236214	0.065473	0.012383	0.117841	0.07138	-0.08333	-0.02301	0.058633	0.122369
intake1104	0.114278	0.022692	-0.04707	0.014865	-0.1713	-0.11066	-0.01915	0.067391	-0.0784	0.20982	0.019091	-0.02752
intake1105	-0.02635	-0.00578	0.154025	0.113217	-0.18358	-0.25252	0.047432	0.161495	0.060221	0.005581	0.133068	0.031523
intake1106	-0.15941	-0.07968	0.020992	-0.00234	-0.11534	-0.02373	0.03597	-0.17036	-0.22344	0.082508	0.073465	0.035731
intake1107	0.044203	0.0384	-0.03595	0.256534	0.239454	-0.0573	-0.02723	-0.15032	-0.07907	0.085076	-0.00235	-0.19988
intake1108	0.186547	0.105396	-0.04167	0.138478	0.041354	-0.06185	-0.15919	-0.0473	0.071487	0.127054	0.171327	0.002436
intake1109	-0.10203	-0.02101	-0.04716	0.264951	0.046174	-0.19819	0.128128	-0.08383	0.013863	-0.00865	0.119025	-0.0275

Factor40	Factor41	Factor42	Factor43	Factor44	Factor45	Factor46	Factor47	Factor48	Factor49	Factor50	Factor51	Factor52
0.028076	0.108039	−0.04651	−0.07577	−0.05772	−0.09846	−0.03992	0.059423	−0.01921	−0.11523	−0.13349	0.194908	0.073627
−0.05808	−0.03525	−0.045	0.137385	−0.01373	−0.06783	0.165921	0.123651	0.008293	−0.10654	0.063093	0.047372	−0.01299
−0.14457	−0.00259	0.168288	0.122431	−0.07779	0.085906	0.100586	−0.05693	−0.01366	0.103401	0.096445	0.045557	0.036265
−0.07361	0.071867	−0.02246	0.005565	−0.01926	−0.04208	0.061717	−0.06024	0.074979	0.006742	0.074233	0.238317	0.101999
0.119034	0.076258	0.024255	0.016638	−0.13234	0.004124	0.037667	0.093915	0.028223	0.035438	−0.05231	0.039217	0.106894
−0.14575	0.187491	−0.05965	0.116311	0.129471	0.120129	−0.07322	−0.12723	−0.02678	0.060111	−0.01499	−0.02848	0.029283
0.103984	0.05904	0.01451	−0.05297	0.04272	0.041302	−0.14673	0.082866	0.022234	0.002622	−0.06945	−0.12492	0.028422
0.033016	0.128783	0.307454	−0.1027	0.034154	−0.05295	−0.11089	0.057504	−0.06858	0.169986	−0.04602	0.197429	−0.09141
−0.03312	0.134273	0.034301	0.035457	−0.05789	−0.17941	−0.05812	−0.07467	0.178821	0.129747	0.011699	−0.0339	0.039091
0.033123	0.05491	0.078484	0.120858	−0.07487	0.067219	0.06637	−0.08748	0.0892	0.134333	−0.06612	0.031504	−0.06376
0.053755	0.001908	0.129973	−0.00096	0.037308	−0.10158	−0.04735	−0.08109	−0.1244	0.013932	0.121859	0.046929	−0.04888
−0.0062	−0.16656	−0.09185	0.02828	−0.08208	0.090782	0.062086	0.044989	−0.14134	0.052869	0.018164	0.06483	0.067054
0.098279	−0.04859	0.028714	0.010227	0.059651	−0.04961	−0.03181	−0.09262	−0.00301	0.092418	0.088845	0.066573	−0.04813
0.224187	−0.08977	−0.03779	−0.05999	0.038065	0.075457	−0.00484	−0.07026	−0.08123	0.002442	0.019263	0.12623	−0.02874
−0.07513	0.067213	0.011171	−0.00254	−0.07781	0.047191	0.000141	−0.02221	−0.05594	0.036539	0.061456	0.160328	−0.20969
0.024141	−0.03341	0.146478	0.063807	−0.06427	−0.02794	0.115686	−0.08317	−0.07532	0.017678	−0.07241	−0.06273	0.067526
0.019037	0.057618	−0.0325	−0.01313	−0.14006	0.022086	−0.1236	0.119329	0.062972	−0.00387	−0.06985	−0.03506	0.005378
−0.06882	−0.06122	−0.08292	−0.07538	−0.12752	0.139144	−0.02658	−0.17468	0.05017	0.05895	0.032342	−0.03402	−0.02205
−0.04166	−0.00505	0.04426	−0.06003	−0.05876	−0.0173	−0.04508	0.014289	0.015723	0.087138	−0.12742	−0.23737	−0.02317
0.067059	−0.03093	−0.03543	0.147008	0.061973	0.22008	0.153357	0.000304	−0.03008	0.093542	0.019972	−0.04543	0.075953
0.0317	0.170892	0.011505	−0.00227	0.157102	−0.01313	0.144712	−0.1057	0.050981	−0.11332	−0.06235	−0.0547	0.104297
0.261906	0.161532	0.049248	−0.01307	0.045087	0.062555	0.127273	−0.08513	0.119278	−0.10225	−0.07636	0.145482	−0.07804
0.108501	−0.10598	−0.10057	−0.00671	−0.02849	−0.172	0.000665	−0.094	−0.08291	−0.09958	0.161476	−0.16618	−0.07335
−0.07832	0.133741	−0.10368	0.055024	−0.09953	−0.01973	0.055169	0.074617	0.120004	−0.10057	−0.21811	−0.08255	−0.13211
0.161119	0.185086	−0.06444	0.035226	0.12637	0.088466	−0.0275	−0.13335	−0.0551	0.053503	−0.00702	0.034781	−0.20429
0.084048	−0.0802	0.091429	−0.12514	−0.12646	0.097849	0.03422	0.07314	0.147834	0.096555	0.042275	−0.04938	−0.08032
−0.04622	0.000535	0.182292	−0.08141	−0.10783	−0.12229	−0.11969	0.020879	−0.06817	0.094407	−0.05376	−0.07519	−0.07393
0.028185	−0.13046	0.138973	0.033941	0.074396	0.133469	−0.12831	0.030839	0.07327	−0.11871	0.046954	0.025573	0.00267
−0.1024	−0.06859	0.040345	0.105757	−0.08751	−0.05698	−0.03502	−0.10496	−0.06721	−0.2447	−0.17447	0.055508	−0.02863
0.019515	0.141465	0.095278	0.154385	−0.06851	−0.03839	0.029281	−0.11315	0.005751	0.044417	0.140757	0.302262	0.044865
−0.12165	0.023361	−0.12065	−0.04976	0.170752	0.059365	−0.0225	0.187972	0.029339	0.187253	0.051442	0.111021	−0.00354
−0.05208	−0.01557	0.07982	−0.03887	−0.0325	0.000202	−0.34259	−0.15674	−0.02588	0.046107	0.01638	0.092128	0.243206
−0.02205	−0.0693	−0.02881	−0.036	−0.24816	0.04678	0.056556	0.013385	−0.16721	−0.03676	0.013228	0.018375	−0.15697
−0.07058	−0.03497	0.023303	0.092309	0.039259	0.108202	0.012239	−0.00612	########	−0.0645	−0.1064	0.008661	0.050253
0.086538	−0.02652	−0.03348	0.036752	−0.03334	0.121328	0.006791	−0.11091	0.00529	0.121844	0.061445	−0.04169	0.039073
0.026676	−0.14115	0.122905	0.194093	−0.21213	−0.01531	0.074123	−0.03038	0.026391	−0.09788	0.076536	0.024734	−0.00954
−0.14375	−0.12551	−0.02162	−0.03976	0.081114	−0.1631	0.056147	0.068242	−0.16695	0.018456	−0.0907	0.034185	−0.0093
−0.16005	−0.02464	0.065611	0.020143	−0.04757	−0.04733	−0.09613	−0.09083	0.087466	0.202848	0.017492	0.044142	−0.01697
0.061279	0.085681	−0.01894	0.081937	0.006657	−0.02915	−0.21033	0.060657	0.166322	−0.14517	0.133503	0.139564	0.014756
−0.02778	0.005607	0.169966	0.033985	0.143384	−0.08614	−0.0676	0.011606	0.114967	−0.03686	−0.03811	0.021348	−0.23236
−0.05577	−0.07703	−0.19902	0.032214	−0.10062	0.049414	−0.10282	−0.06529	0.176152	−0.06624	0.204837	−0.05504	−0.06208
0.046419	−0.0611	−0.05424	0.073021	0.031218	0.100379	−0.10615	−0.05565	0.017825	−0.10461	−0.10181	−0.10712	0.06595
0.026507	−0.00572	0.021137	0.054183	0.070834	0.176413	−0.1144	−0.06566	0.135075	−0.05088	−0.10217	0.013158	0.009903
−0.0584	0.09751	−0.0307	−0.14437	−0.1414	−0.10637	−0.06685	−0.00524	0.172119	−0.09577	−0.05135	−0.20817	0.091727
0.059337	0.140272	−0.24076	−0.14901	−0.11583	0.040339	−0.0578	0.062452	0.072223	0.140726	0.018562	0.151555	0.017918
0.025698	−0.05981	0.011575	−0.15764	0.196999	−0.10294	0.002236	−0.02754	−0.0333	0.080399	0.05803	−0.03512	0.015826
−0.05289	0.078437	0.015829	0.028531	−0.03773	0.0687	0.040586	−0.01743	−0.04154	−0.11218	0.012205	−0.02156	0.215786
−0.01	−0.08977	−0.15104	−0.18086	0.017275	0.08718	0.031862	−0.05522	−0.0266	0.11725	0.003301	−0.04695	0.147764
0.031714	0.027954	−0.00626	0.109332	0.027303	−0.00494	−0.13987	0.06988	−0.00382	0.042963	−0.10448	−0.10569	0.127355
−0.10648	−0.07963	−0.13954	−0.02895	0.01524	−0.00881	0.016716	−0.15494	−0.01856	0.006794	0.158749	0.02389	0.07849
0.221009	−0.12557	−0.02258	−0.13532	0.060006	−0.2113	−0.08976	−0.10286	−0.05323	−0.11493	0.083965	−0.09771	−0.07959
−0.06674	−0.01257	−0.17178	−0.00764	0.022119	0.112504	0.042302	0.08191	−0.0329	0.156553	−0.03605	0.044662	0.039604
−0.12925	0.031241	0.170624	−0.10009	−0.13562	0.172363	−0.10422	0.002902	0.028583	−0.0284	−0.21608	0.014057	0.032444
−0.02328	0.0335	0.091414	0.028162	0.114151	−0.01636	−0.03441	0.093967	−0.0077	−0.07338	−0.02642	−0.03195	−0.03806
0.021813	−0.00991	0.007259	−0.08758	−0.01301	−0.05284	−0.01493	0.024062	−0.11473	−0.07861	0.097885	−0.01836	−0.05603
0.014557	−0.12884	−0.01188	−0.03991	0.099625	−0.01939	0.08216	−0.01135	0.138315	−0.06558	−0.0557	0.175039	−0.00726
0.02692	−0.0009	0.019181	−0.17398	−0.01056	−0.14658	−0.03143	0.078157	−0.19592	0.039621	−0.08672	0.123711	0.076167
0.085065	−0.07271	0.018854	−0.01426	0.042473	−0.0253	0.002324	−0.0864	0.013808	−0.08445	−0.0101	0.154742	−0.01831
0.049981	−0.06756	0.045609	−0.19353	0.158501	0.146471	0.146206	0.072406	−0.01249	−0.07622	0.086433	−0.03378	−0.23689
−0.06849	0.15733	0.048595	0.096281	−0.13144	−0.0181	0.094616	−0.04753	−0.00122	0.177763	0.101654	−0.0827	−0.00298
0.119194	0.209938	−0.06491	−0.01817	−0.10181	0.084109	0.28826	0.010059	0.127451	0.047253	−0.10653	−0.10853	−0.13607
0.067859	0.058123	−0.06061	0.001471	0.090537	0.016015	0.00311	0.132672	−0.08601	0.06543	−0.01405	0.090846	−0.02897
−0.04522	−0.03711	0.1208	0.074067	−0.09876	−0.04014	−0.05128	0.147529	0.040375	−0.05789	0.075046	0.002325	−0.06585
0.100246	0.11945	0.236453	−0.04106	0.023603	0.072128	−0.00927	−0.16642	−0.18162	0.051345	0.044884	0.043936	−0.00259
0.01536	−0.04364	0.026217	0.056415	0.086822	0.010787	0.063474	0.19949	0.017215	−0.04884	0.057624	−0.00041	0.016999
−0.05913	0.044477	−0.06947	0.068546	0.03786	−0.09074	−0.12595	0.032625	−0.0366	0.098554	0.175021	−0.10268	0.041849
−0.09975	−0.0717	−0.04915	−0.00871	0.022646	−0.17329	0.240962	−0.20329	0.008439	−0.02614	−0.0575	−0.04916	0.170823
0.040834	−0.05319	0.046984	0.089611	0.040503	−0.13292	0.13234	0.062126	0.143635	0.053463	0.075919	0.006989	−0.06014
−0.03724	0.058739	0.069901	0.107003	0.041496	0.072259	−0.04324	−0.03284	−0.14925	−0.0852	0.028669	−0.06458	0.127685
−0.05759	−0.00213	−0.23615	−0.00473	0.012189	0.006423	−0.12069	0.029087	−0.02616	−0.02372	0.003376	−0.03257	0.046097
−0.0645	0.038219	0.082503	−0.00132	0.07857	0.056676	−0.11298	−0.19579	−0.18204	−0.17213	−0.00345	−0.06905	−0.06221
0.072368	0.025572	−0.18609	0.12537	0.127023	−0.17646	−0.09585	0.151515	0.061278	0.09259	−0.02022	0.04459	0.134256
0.047424	−0.08057	0.020535	−0.01923	0.109959	−0.05895	0.051448	0.020365	0.137724	0.029366	−0.02965	−0.09412	0.113768
0.011834	−0.03343	0.003343	−0.18256	0.020063	0.038624	−0.1089	0.146268	−0.00322	0.00977	0.13806	−0.09421	−0.09616
0.01781	−0.053	−0.07574	0.133073	0.015035	0.009218	0.037368	0.045116	0.031805	0.017044	0.026934	−0.03025	−0.11192
−0.01225	−0.12945	−0.06942	0.057996	−0.12442	0.147305	−0.13828	0.023095	−0.05179	0.044307	−0.13521	−0.02932	−0.12105
−0.05972	−0.05511	−0.09821	0.148789	0.199162	−0.07479	−0.08966	−0.08478	−0.02761	−0.06365	−0.30507	−0.00137	−0.03953
−0.0169	−0.0716	0.065045	−0.10338	0.040873	0.000873	0.010168	0.008327	0.151589	−0.04005	0.257514	−0.0634	0.080493
0.018073	−0.2105	0.106035	−0.04483	0.026616	0.072806	0.063465	−0.00026	0.044045	−0.09691	−0.13296	0.079373	0.099109
0.06371	0.11144	−0.09709	−0.05806	0.003397	−0.16461	0.074672	−0.08285	−0.11701	0.258024	−0.13033	−0.03556	0.033361
−0.00846	0.038248	−0.01937	0.21048	−0.12314	−0.01771	−0.12502	−0.0399	−0.02611	0.080945	0.152798	−0.11401	−0.06485
−0.07399	−0.1053	0.144214	0.011625	0.013504	0.01052	−0.10559	0.083901	0.113433	0.164974	0.037012	0.028688	−0.09317
−0.03479	−0.01849	0.079885	−0.01548	−0.04087	−0.17487	0.100341	0.233785	−0.02754	−0.02184	−0.00307	0.030842	0.097947
0.082742	−0.01053	−0.12598	−0.07797	−0.07583	0.152613	0.034353	0.093663	−0.07384	0.021773	−0.05142	0.250077	0.048081
0.025907	−0.1856	0.07696	−0.02047	0.214326	0.041887	−0.09446	−0.07263	0.285257	0.080993	−0.07377	−0.01453	−0.08
−0.12414	−0.06872	0.020709	0.004317	0.017323	0.001019	0.074273	0.106783	−0.04306	−0.13494	0.071348	0.118822	−0.02545
0.025819	−0.07681	−0.00469	−0.02037	−0.15666	0.009096	0.033206	0.022296	0.00544	−0.01955	−0.0315	−0.02787	0.014674
0.185199	0.015909	0.094488	−0.10449	−0.12713	0.101152	0.003701	0.068037	0.154396	0.029406	0.051419	−0.15362	0.106566
−0.15374	−0.06989	−0.06397	−0.06831	−0.01363	−0.15349	0.117998	0.059545	0.045286	0.065254	−0.09898	0.00928	−0.21544
0.068415	−0.04249	−0.00506	−0.01936	−0.03137	0.001223	−0.02546	0.054865	0.017115	0.017253	−0.06786	0.016066	0.026577
−0.09917	0.149545	0.033134	−0.13392	0.060439	−0.03269	0.097896	−0.13449	−0.14858	0.028767	0.113993	−0.23658	0.006249
−0.05625	0.163448	−0.19053	−0.01352	0.087333	0.142775	−0.07428	−0.09483	0.017369	−0.13275	0.116169	0.067151	−0.11337
−0.07764	−0.04916	−0.02714	0.137991	−0.12438	−0.17467	−0.0445	0.087939	−0.02187	0.042538	−0.07292	0.091546	0.057762
−0.04434	−0.12833	0.084123	0.035471	−0.00789	0.150838	0.134825	0.042504	0.030302	0.050002	0.061112	−0.00566	0.101028
−0.07083	−0.01257	−0.10759	−0.05101	−0.08519	−0.03548	−0.09366	−0.06848	−0.0368	−0.17433	−0.10662	0.023697	−0.16206
0.078564	−0.03779	−0.01736	0.026543	0.179167	−0.14174	−0.02757	0.020627	0.136598	0.060637	0.063893	0.02766	0.029455
0.023679	−0.07969	0.019585	0.051063	0.072862	0.063648	−0.17243	−0.00901	−0.03224	0.15346	−0.06561	0.04563	0.106675
−0.19519	−0.00369	0.044012	0.091168	0.141774	0.125205	0.060172	0.079017	−0.06215	0.035956	−0.00319	−0.08184	−0.00983
0.039047	−0.04873	−0.02632	−0.16383	0.026596	0.159567	−0.04413	0.110946	−0.22235	0.047156	−0.08217	−0.07342	−0.03466
−0.12365	−0.14755	−0.03194	0.073588	0.057577	0.009573	−0.02574	0.03013	−0.16038	0.169346	−0.01855	0.003454	−0.17251
−0.0726	0.185995	0.125693	0.107938	0.207763	0.089533	0.048674	0.169895	−0.04263	−0.00918	0.020032	−0.12336	0.009104
−0.10457	0.007482	−0.03681	−0.20239	−0.01359	−0.10411	0.058193	−0.29821	0.128454	0.085197	−0.10239	0.119268	−0.11497
0.113331	−0.1136	0.059147	0.080104	−0.00175	−0.08899	0.074138	−0.08414	0.017138	0.030633	−0.09755	−0.05128	0.090418
0.053763	0.072712	−0.0246	0.017546	0.125881	0.024254	0.044134	−0.01067	0.084031	0.030967	−0.16419	−0.02847	0.075776
−0.22616	0.126067	−0.04335	0.043793	0.137563	−0.00613	0.062377	0.035264	−0.03462	−0.10959	0.122445	0.04549	−0.03224
−0.40213	0.00679	0.055265	−0.23092	0.017408	0.063599	0.011141	−0.04915	0.236943	−0.00608	−0.02355	0.046323	−0.02422
−0.04793	0.070569	−0.04622	−0.14273	−0.05377	0.041872	−0.03539	0.046256	0.020726	−0.18308	0.15608	0.085178	0.151986
0.059948	−0.23908	−0.14165	0.234284	−0.00508	0.036207	0.008046	−0.16175	0.032992	0.12569	0.002409	0.022149	−0.11805
0.073134	0.168104	−0.05251	0.164129	−0.07959	−0.09928	−0.13505	0.100574	0.014406	−0.04474	−0.01536	−0.06123	−0.21888
