# Supplementary figures and images for: Identification of Salty Dietary Patterns of the Japanese Macroregion
Source: J Nutr Metab. 2021 Jul 22;2021:6675418. doi: 10.1155/2021/6675418 (PMC8321765; doi:10.1155/2021/6675418)

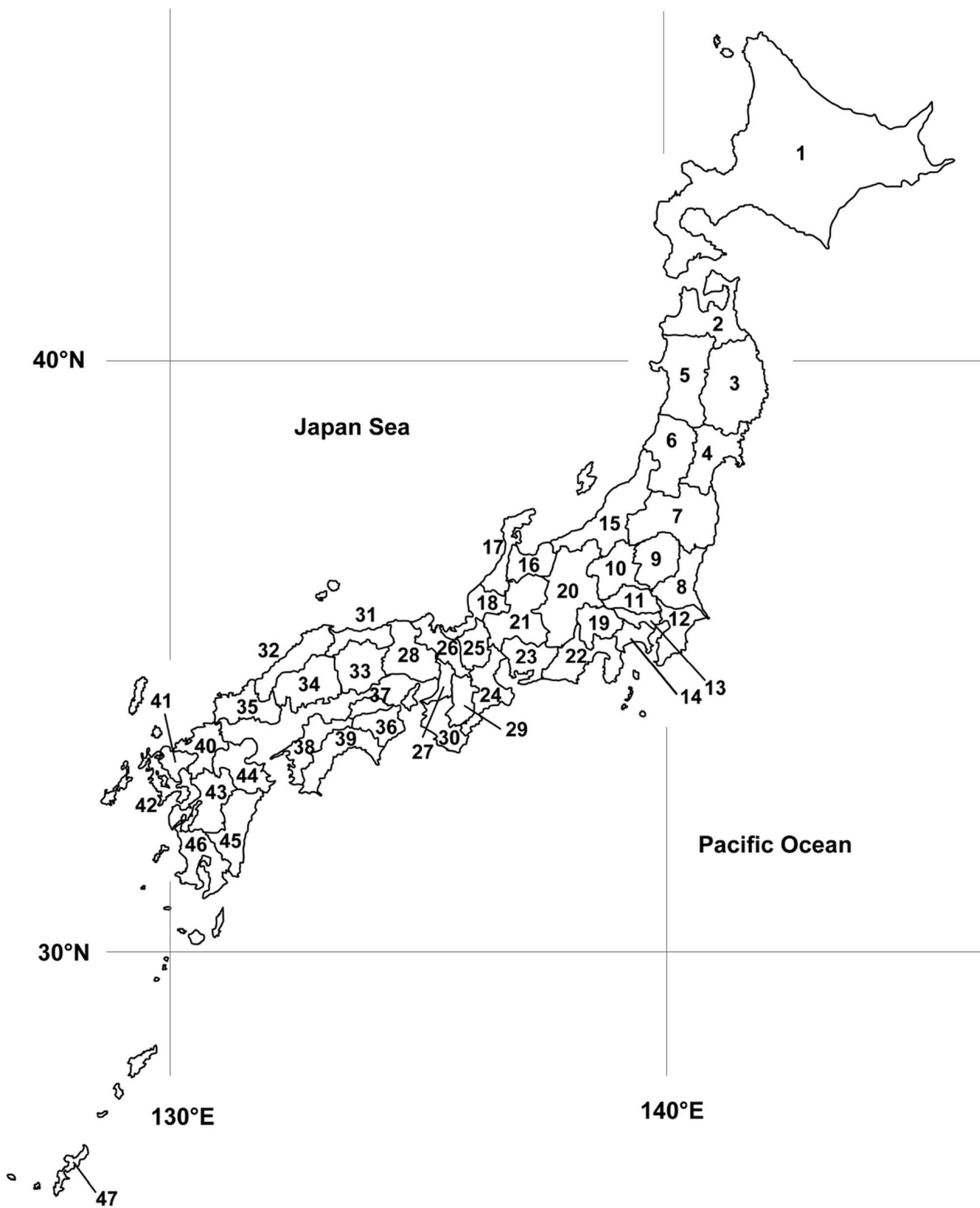

Supplement: Supplementary Materials — Supporting File S1: details of 109 foods and calculation units used for nutritional value (data for 2013). Supporting File S2: details of 109 foods and calculation units used for nutritional value (data for 2018). Figure S1: the distribution map of prefectures in Japan. [file 6675418.f1.zip › 6675418.f1/Figure S1.pdf]
